# The novel cyclophilin inhibitor C105SR reduces hepatic ischaemia–reperfusion injury via mitoprotection

**DOI:** 10.1016/j.jhepr.2023.100876

**Published:** 2023-08-16

**Authors:** Amel Kheyar, Nazim Ahnou, Abdelhakim Ahmed-Belkacem, Anne Hulin, Claire Pressiat, Bijan Ghaleh, Jean-François Guichou, Didier Morin, Jean-Michel Pawlotsky, Fatima Teixeira-Clerc

**Affiliations:** 1 Équipe “Virus, Hépatologie, Cancer”, INSERM U955, IMRB, Université Paris-Est, Créteil, France; 2 Laboratoire de Pharmacologie, DMU de Biologie et Pathologie, Hôpitaux Universitaires Henri Mondor, AP-HP, Créteil, France; 3 Équipe “Pharmacologie et Technologies pour les Maladies Cardiovasculaires”, INSERM U955, IMRB, Université Paris-Est, Créteil, France; 4 Centre de Biologie Structurale (CBS), Université de Montpellier, CNRS, INSERM, Montpellier, France; 5 Département Prévention, Diagnostic et Traitement des Infections, DMU de Biologie et Pathologie, Hôpitaux Universitaires Henri Mondor, AP-HP, Créteil, France

**Keywords:** Cellular protection, Peptidyl-prolyl *cis*-*trans* isomerase activity, Liver necrosis, Mitochondrial calcium retention capacity, Mitochondrial permeability transition pore, Mitochondrial swelling

## Abstract

**Background & Aims:**

Mitochondrial permeability transition pore (mPTP) opening is critical for mediating cell death during hepatic ischaemia–reperfusion injury (IRI). Blocking mPTP opening by inhibiting cyclophilin D (CypD) is a promising pharmacological approach for the treatment of IRI. Here, we show that diastereoisomers of a new class of small-molecule cyclophilin inhibitors (SMCypIs) have properties that make them attractive candidates for the development of therapeutic agents against liver IRI.

**Methods:**

Derivatives of the parent SMCypI were synthesised and evaluated for their ability to inhibit CypD peptidyl-prolyl *cis*-*trans* isomerase (PPIase) activity and for their mitoprotective properties, evaluated by measuring mitochondrial swelling and calcium retention capacity in liver mitochondria. The ability of the selected compounds to inhibit mPTP opening was evaluated in cells subjected to hypoxia/reoxygenation using a calcein/cobalt assay. Their ability to inhibit cell death was evaluated in cells subjected to hypoxia/reoxygenation by measuring lactate dehydrogenase (LDH) release, propidium iodide staining, and cell viability. The compound performing best *in vitro* was selected for *in vivo* efficacy evaluation in a mouse model of hepatic IRI.

**Results:**

The two compounds that showed the strongest inhibition of CypD PPIase activity and mPTP opening, C105 and C110, were selected. Their SR diastereoisomers carried the activity of the racemic mixture and exhibited mitoprotective properties superior to those of the known macrocyclic cyclophilin inhibitors cyclosporin A and alisporivir. C105SR was more potent than C110SR in inhibiting mPTP opening and prevented cell death in a model of hypoxia/reoxygenation. Finally, C105SR substantially protected against hepatic IRI *in vivo* by reducing hepatocyte necrosis and apoptosis.

**Conclusions:**

We identified a novel cyclophilin inhibitor with strong mitoprotective properties both *in vitro* and *in vivo* that represents a promising candidate for cellular protection in hepatic IRI.

**Impact and Implications:**

Hepatic ischaemia–reperfusion injury (IRI) is one of the main causes of morbidity and mortality during or after liver surgery. However, no effective therapies are available to prevent or treat this devastating syndrome. An attractive strategy to prevent hepatic IRI aims at reducing cell death by targeting mitochondrial permeability transition pore opening, a phenomenon regulated by cyclophilin D. Here, we identified a new small-molecule cyclophilin inhibitor, and demonstrated the enhanced mitoprotective and hepatoprotective properties of one of its diastereoisomers both *in vitro* and *in vivo*, making it an attractive lead compound for subsequent clinical development.

## Highlights

• C105SR is a novel cyclophilin inhibitor belonging to the family of small-molecule cyclophilin inhibitors (SMCypIs).
• C105SR inhibits cyclophilin D peptidyl-prolyl *cis-trans* isomerase activity.
• C105SR inhibits mitochondrial swelling and increases calcium retention capacity of isolated liver mitochondria.
• C105SR inhibits mPTP opening and prevents cell death in an *in vitro* model of hypoxia/reoxygenation.
• C105SR protects against hepatic ischaemia–reperfusion injury *in vivo* by reducing hepatocyte necrosis and apoptosis.

## Graphical abstract

**Figure:**
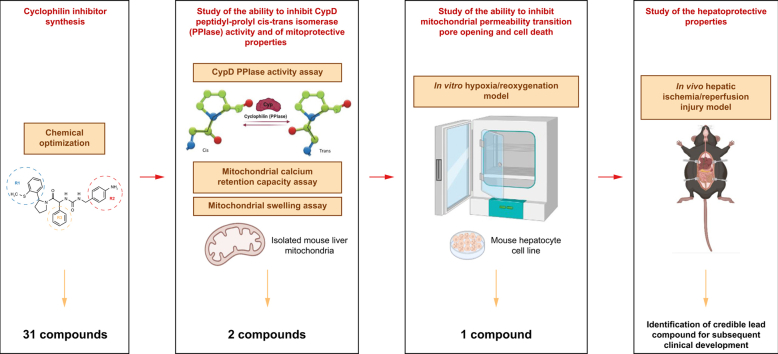

## Introduction

Hepatic ischaemia–reperfusion injury (IRI) is a severe complication of various clinical conditions, including haemorrhagic shock, liver resection, and liver transplantation. Hepatic IRI is a leading cause of early allograft dysfunction and a major risk factor for acute and chronic rejection, particularly when liver grafts with expanded criteria are used, including those from marginal, deceased, and non-beating heart donors.[1], [2], [3] Hepatic IRI can lead to multi-organ dysfunction or systemic inflammatory response syndrome, both of which carry high risks of mortality. No specific treatment is available to reduce hepatic IRI, and current management is based on supportive care.

IRI occurs as the result of a biphasic phenomenon in which cellular damage caused by hypoxia is paradoxically exacerbated by the restoration of oxygen delivery. Mitochondrial dysfunction plays an important role in the pathogenesis of IRI.^4^ In particular, mitochondrial permeability transition (mPT) is thought to be a critical mediator of the damage that accompanies reperfusion of organs following prolonged ischaemia. mPT is defined as a sudden increase in the permeability of the inner mitochondrial membrane to solutes with a molecular mass less than 1.5 kDa. mPT is mediated by the opening of the mPT pore (mPTP), a high-conductance channel involved in Ca^2+^ homeostasis. Persistent mPTP opening during IRI results in loss of membrane potential, uncoupling of oxidative phosphorylation, ATP depletion, and mitochondrial swelling, ultimately leading to necrotic cell death. In addition, mPTP releases cytochrome C from mitochondria, which in turn triggers apoptosis. Although the molecular composition of the mPTP is still debated, it is recognised that mPTP opening is regulated by cyclophilin D (CypD).^5^^,^^6^

CypD is a member of a family of highly homologous peptidyl-prolyl *cis*-*trans* isomerases (PPIases) that catalyse the interconversion of two energetically preferred conformers (*cis* and *trans*) of the planar peptide bond preceding an internal proline residue. Cyclophilins carry chaperone activity that regulates protein folding. The PPIase domain catalyses cyclophilin isomerase activity. It contains two binding pockets: the catalytic site of PPIase (the S1 pocket) and the so-called “gatekeeper pocket” (the S2 pocket), whose functional role remains unknown.^7^ Cyclophilins share a common cyclophilin-like domain (CLD) and a domain of approximately 109 amino acids, flanked by domains specific for each cyclophilin responsible for its subcellular compartmentalisation and functional specialisation. CypD is located in the mitochondrial matrix and acts as a key regulator of mPTP opening.^5^^,^^6^ Persistent CypD-dependent mPTP opening induces necrotic cell death, which plays a key role in IRI.^8^ Conversely, CypD-deficient cells are highly resistant to cell death induced by cytosolic calcium overload and hydrogen peroxide-mediated stress induced by reactive oxygen species.^9^ Thus, inhibition of mPTP opening by compounds that target CypD represents an attractive strategy for cellular protection in the context of hepatic IRI.

Cyclosporin A (CsA) and sanglifehrin A (SfA) are two structurally distinct natural Cyp inhibitors that potently inhibit mPTP opening through CypD inhibition, but their immunosuppressive properties limit their therapeutic use as Cyp inhibitors.^10^^,^^11^ Non-immunosuppressive derivatives of these molecules have been generated by chemical modifications. Among them, NIM811, a CsA analogue, can inhibit mPTP opening and protect against cell death after liver transplantation.^12^^,^^13^ Alisporivir (Debio-025, ALV), another CsA analogue, was developed for the treatment of hepatitis C virus infection, but its clinical development was halted due to adverse events unrelated to Cyp inhibition.^14^ Other CsA or SfA derivatives have been proposed that have potential utility for the treatment of liver diseases.^15^^,^^16^ However, no compound targeting cyclophilins has been clinically approved to date. Thus, novel potent cyclophilin inhibitors are required to make mPTP opening inhibition credible as a hepatoprotective strategy.

We have recently developed a new family of non-peptidic, small-molecule cyclophilin inhibitors (SMCypIs), which is chemically distinct from all currently known cyclophilin inhibitors.^17^ We show that one of the SMCypIs derivatives, compound C31, exerts mitoprotective effects *in vitro* and protects cells in an *in vivo* murine model of liver IRI.^18^ Here, we chemically improved C31, identified a new lead SMCypI compound, and demonstrated the enhanced mitoprotective and hepatoprotective properties of one of its diastereoisomers both *in vitro* and *in vivo*, making it an attractive lead compound for subsequent clinical development.

## Materials and methods

### Drugs

Unless otherwise mentioned, all reagents were purchased from Sigma-Aldrich. Calcein-AM and Calcium green 5N were purchased from Invitrogen. DMSO was used as the vehicle control of cyclophilin inhibitors for all *in vitro* and *in vivo* experiments, at the same dilutions as used in making cyclophilin inhibitor working solutions.

### SMCypI synthesis

Chemical reagents were obtained from ThermoFisher, Acros Organics, Spirochem, ACB Blocks, and Enamine, and were used without further purification. Compounds C31 and C32 were synthesised as previously described.^15^ The synthesis of the other compounds is detailed in Supporting Materials and Methods.

### Animals

Male C57BL/6J mice (8–12 weeks-old, n = 45) were purchased from Janvier (Le Genest-St-Isle, France). All animals were housed in an air-conditioned room with nycthemeral rhythm (12-h light/dark cycles) and were granted free access to water and standard rodent chow. All animal procedures in this study were conducted in accordance with the directives of the European Parliament (2010/63/EU-848 EEC) and approved by the Animal Ethics Committee ANSES/ENVA/Université Paris-Est-Créteil.

### Hepatic IRI *in vivo* model

Mice (10–12 weeks-old) were anesthetised with isoflurane and subcutaneously implanted with Alzet® osmotic pump containing C105SR (50 mg/kg) or vehicle 24 h before the surgical procedure. The mice were anesthetised with isoflurane and subjected to partial hepatic ischaemia by clamping the hepatic artery and portal vein for 60 min, followed by 6 h of reperfusion induced by removal of the clamp. The sham-operated group underwent laparotomy without vascular occlusion.

### Statistical analysis

All results are expressed as mean ± standard error of the mean (SEM) from at least 3 independent experiments. Statistical analyses were performed with GraphPad Prism v.9.1.2 Software using the Mann-Whitney *U* test or the one-way ANOVA analysis followed by a Tukey’s or a Dunnett's post-test if ANOVA produced a significant value of F (*p* <0.05). Differences were considered significant when *p* <0.05.

## Results

### Chemical optimisation and validation of novel CypD inhibitors with enhanced mitoprotective properties

**Fig. 1:**
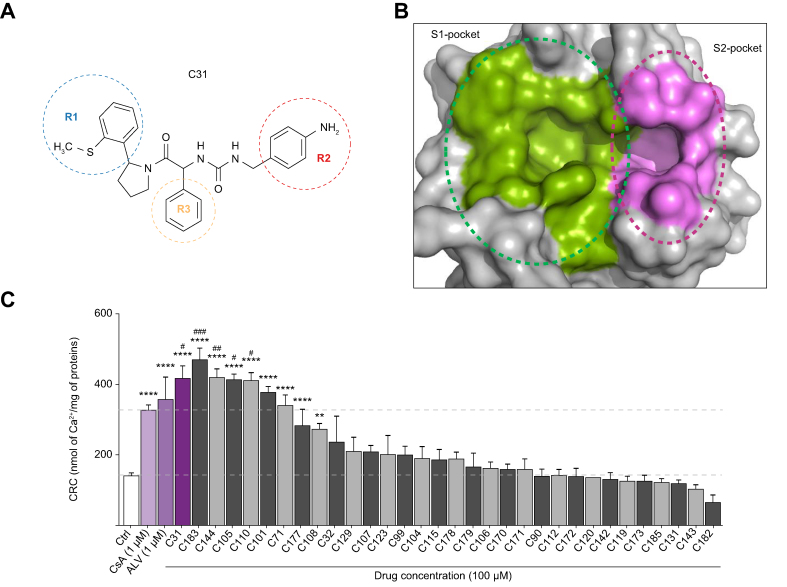
Concentration-dependent enhancement of calcium retention capacity of isolated mouse liver mitochondria by SMCypI C31 derivatives. (A) Chemical structure of compound C31. (B) Surface representation of CypD showing the PPIase catalytic site (S1 pocket) and the gatekeeper pocket (S2 pocket). (C) CRC of mouse liver mitochondria in the absence (Ctrl) or in the presence of CsA (1 μM), ALV (1 μM), C31 (100 μM), or C31 derivatives (100 μM), ranked by decreasing level of CRC, expressed as nmol of calcium per mg of mitochondrial proteins. Data are shown as mean ± SEM. One way ANOVA analysis followed by Tukey’s or Dunnett's post-test if ANOVA produced a significant value of F. ∗∗*p* <0.001 *vs*. Ctrl; ∗∗∗∗*p* <0.0001 *vs*. Ctrl; ^#^*p* <0.05 *vs*. CsA; ^##^*p* <0.01 *vs*. CsA; ^###^*p* <0.0001 *vs*. CsA. ALV, alisporivir; CsA, cyclosporin A; CypD, cyclophilin D; CRC, calcium retention capacity; PPIase, peptidyl-prolyl *cis*-*trans* isomerase; SMCypIs, small-molecule cyclophilin inhibitors.

To improve the potency of our previously reported SMCypI C31, chemical modifications of its three functional regions, including R1 that binds to the S1 pocket, R2 that binds to the S2 pocket, and R3 that interacts with residues between the two pockets (Fig. 1A and B), were performed. A library of compounds was generated in which: (i) the phenyl-pyrolidine group (R1 position) was modified to remove the thio-methyl group using benzothiophene ring; (ii) the aniline group (R2 position) was modified to remove the aniline motif using, for example, different heterocycles containing an amine moiety; (iii) the phenyl group (R3 position) was substituted to allow interaction with CypD residues R97 and H168. A total of 31 derivatives with single, double, or triple chemical modifications of the R1, R2, and/or R3 groups were selected to reflect these changes (Fig. S1).

We first assessed the capacity of the newly generated compounds to inhibit CypD PPIase activity using a standard chymotrypsin-coupled assay. The assay relies on the ability of α-chymotrypsin to release *p*-nitroanilide from peptides such as N-succinyl-Ala-Ala-Pro-Phe *p*-nitroanilide only if the prolyl amide bond is in the *trans* conformation. The release of *p*-nitroanilide is enhanced by the PPIase activity of CypD after isomerisation of the *cis* conformer into *trans*. The IC_50_ values in the PPIase assay ranged from 0.10 ± 0.02 to 1.26 ± 0.37 μM (Table 1), *vs*. 0.02 ± 0.003 μM and 0.03 ± 0.005 μM, for CsA and ALV, two potent macrocyclic cyclophilin inhibitors, respectively (Table 1).

**Table 1:** IC_50_ values for inhibition of CypD PPIase activity by C31 derivatives

Compound	CypD PPIase activity IC_50_ (μM)
CsA	0.02 ± 0003
ALV	0.03 ± 0.005
C31	0.20 ± 0.10
C32	1.18 ± 0.43
C71	0.79 ± 0.21
C90	0.32 ± 0.16
C99	0.63 ± 0.49
C101	0.40 ± 0.19
C104	0.22 ± 0.06
C105	0.35 ± 0.16
C106	1.26 ± 0.11
C107	0.17 ± 0.05
C108	0.48 ± 0.23
C110	0.10 ± 0.02
C112	0.48 ± 0.04
C115	0.50 ± 0.32
C119	0.15 ± 0.03
C120	0.15 ± 0.05
C123	1.26 ± 0.37
C129	0.26 ± 0.07
C131	0.48 ± 0.12
C142	0.42 ± 0.10
C143	0.72 ± 0.21
C144	0.31 ± 0.16
C170	1.05 ± 0.72
C171	0.34 ± 0.13
C172	0.21 ± 0.07
C173	0.65 ± 0.14
C177	0.49 ± 0.32
C178	0.40 ± 0.19
C179	0.28 ± 0.06
C182	0.16 ± 0.03
C183	0.26 ± 0.13
C185	0.59 ± 0.19

ALV, alisporivir; CsA, cyclosporin A; CypD, cyclophilin D; PPIase, peptidyl-prolyl *cis*-*trans* isomerase.

We next evaluated the ability of the newly generated SMCypIs to increase mitochondrial calcium retention capacity (CRC) in isolated mouse liver mitochondria. In basal conditions, 160 ± 8 nmol Ca^2+^/mg of mitochondrial proteins were required to induce mPTP opening. In the presence of CsA or ALV at 1 μM, the amount of Ca^2+^ required to open mPTP was raised to 328 ± 10 and 373 ± 59 nmol Ca^2+^/mg of mitochondrial proteins, respectively (Fig. 1C). Because we previously observed that the maximal effect of C31 was achieved at a concentration of 100 μM,^16^ we first evaluated the ability of the new derivatives to increase the mitochondrial CRC at this concentration. As shown in Fig. 1C, eight compounds increased the mitochondrial CRC. Among them, four compounds displayed a greater maximal mitochondrial CRC than CsA, including C183 (473 ± 38 nmol Ca^2+^/mg of mitochondrial proteins), C144 (422 ± 44 nmol Ca^2+^/mg of mitochondrial proteins), C105 (407 ± 13 nmol Ca^2+^/mg of mitochondrial proteins), and C110 (400 ± 27 nmol Ca^2+^/mg of mitochondrial proteins) (Fig. 1C). These four compounds induced a concentration-dependent increase in the CRC with EC_50_ values of 10.21 ± 0.06 μM for C183, 11.76 ± 0.01 μM for C144, 2.37 ± 0.11 μM for C105, and 2.83 ± 0.09 μM for C110, *vs*. 0.23 ± 0.02 μM and 0.08 ± 0.02 μM for CsA and ALV, respectively (Table 2).

**Table 2:** IC_50_ or EC_50_ values of CsA, ALV, and different SMCypIs derivatives from C31 for inhibition of CypD PPIase activity in an enzyme assay, and for mitochondrial swelling and calcium retention capacity in isolated mouse liver mitochondria

Compound	CypD PPIase activity IC_50_ (μM)	Mitochondrial swelling IC_50_ (μM)	Calcium retention capacity EC_50_ (μM)
CsA	0.02 ± 0.003	0.05 ± 0.01	0.23 ± 0.02
ALV	0.03 ± 0.005	0.05 ± 0.02	0.08 ± 0.02
C31	0.20 ± 0.10	1.30 ± 0.05	9.09 ± 0.21
C183	1.03 ± 0.13	2.56 ± 0.02	10.21 ± 0.06
C144	0.64 ± 0.15	11.57 ± 0.01	11.76 ± 0.01
C105	0.57 ± 0.12	0.69 ± 0.05	2.37 ± 0.11
C110	0.11 ± 0.01	0.37 ± 0.006	2.83 ± 0.09
C105SR	0.005 ± 0.001	0.009 ± 0.001	0.05 ± 0.02
C110SR	0.008 ± 0.002	0.04 ± 0.005	0.14 ± 0.02

ALV, alisporivir; CsA, cyclosporin A; CypD, cyclophilin D; PPIase, peptidyl-prolyl *cis*-*trans* isomerase.

The four compounds also inhibited Ca^2+^-induced swelling of energised isolated mouse liver mitochondria in a concentration-dependent manner (Fig. S2) with IC_50_ values of 2.56 ± 0.02 μM for C183, 11.57 ± 0.01 μM for C144, 0.69 ± 0.05 μM for C105, and 0.37 ± 0.006 μM for C110, *vs*. 0.05 ± 0.01 μM and 0.05 ± 0.02 μM for CsA and ALV, respectively (Table 2). Among the tested compounds, C105 and C110 were found to be the most potent cyclophilin inhibitors with the lowest IC_50_ values. C105 and C110 shared similar substitutions of the R1 (benzothiophene) and R3 (3-bromo-6-methoxy-phenyl) moieties, while C105 had an additional modification of the R2 group (tetrahydroquinoline), as compared with the parent SMCypI compound C31 (Fig. S1 and Fig. 1A).

### Improvement of the mitoprotective properties of the SMCypIs by stereoselectivity

**Fig. 2:**
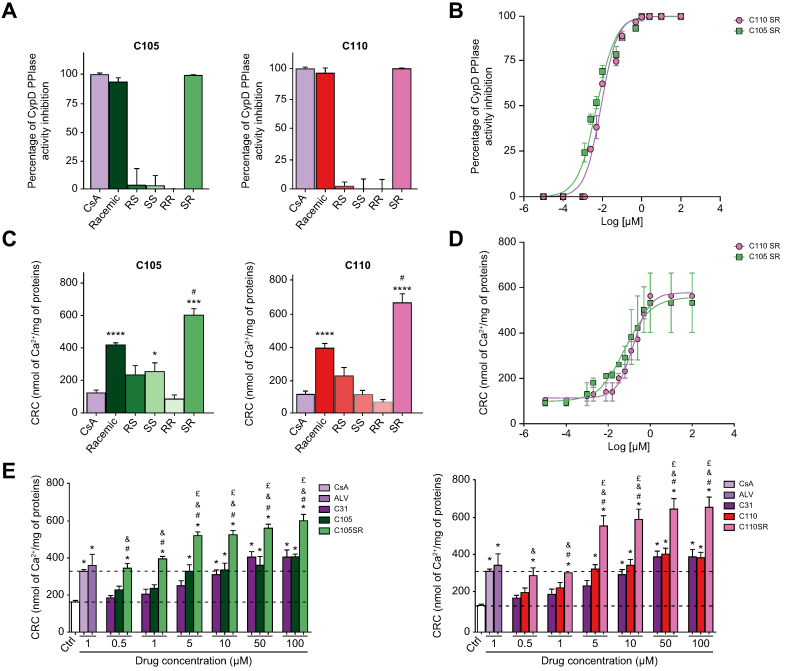
Biological activity of the racemic mixtures and diastereoisomers of compounds C105 and C110. (A) Inhibition of CypD PPIase activity by C105 (left) and C110 (right) racemic mixtures and their diastereoisomers at 10 μM expressed as percent of the complete inhibition of CypD PPIase activity induced by CsA. (B) Concentration-response curves of CypD PPIase activity inhibition by diastereoisomers C105SR and C110SR. (C) Mitochondrial CRC of mouse liver mitochondria in the absence (Ctrl) or in the presence of C105 (left) and C110 (right) racemic mixture and their diastereoisomers at 100 μM. Data are shown as mean ± SEM. One way ANOVA analysis followed by Tukey’s or Dunnett's post-test if ANOVA produced a significant value of F. ∗*p* <0.05 *vs*. Ctrl;; ∗∗∗*p* <0.001 *vs*. Ctrl; ∗∗∗∗*p* <0.0001 *vs*. Ctrl; ^#^*p* <0.05 *vs*. racemic mixture. (D) Concentration-response curves of mitochondrial CRC of compounds C105SR and C110SR. (E) Mitochondrial CRC in the absence or in the presence of 1 μM CsA, or 1 μM ALV or increasing concentrations of C31, C105, and C105SR (left) or C31, C110, and C110SR (right). Data are shown as mean ± SEM. One way ANOVA analysis followed by Tukey’s or Dunnett's post-test if ANOVA produced a significant value of F. ∗*p* <0.05 *vs*. Ctrl; ^#^*p* <0.05 *vs*. racemic mixture; ^&^*p* <0.05 *vs*. C31; ^£^*p* <0.05 *vs*. CsA. CRC, calcium retention capacity; CsA, cyclosporin A; CypD, cyclophilin D; PPIase, peptidyl-prolyl *cis*-*trans* isomerase.

Both C105 and C110 display two asymmetric carbons. We synthesised their four diastereoisomers (SS, SR, RS, RR) (Fig. S3) and evaluated their mitoprotective properties by assessing their ability to inhibit CypD PPIase activity and mPTP opening. Only C105SR and C110SR inhibited CypD PPIase activity (Fig. 2A) and this effect was concentration-dependent (IC_50_ values: 0.005 ± 0.001 μM and 0.008 ± 0.002 μM, respectively) (Table 2 and Fig. 2B). Apart from compound C105SS, which exerted a weak effect, C105SR and C110SR were the only diastereoisomers capable to induce greater than control mitochondrial CRC at 100 μM (Fig. 2C), an effect that was concentration-dependent (EC_50_ values: 0.05 ± 0.02 μM and 0.14 ± 0.02 μM, respectively) (Table 2 and Fig. 2D). Interestingly, C105SR and C110SR showed greater maximal mitochondrial CRC than their respective racemic mixtures (595 ± 38 *vs*. 407 ± 13 nmol Ca^2+^/mg of mitochondrial proteins for C105SR and C105, respectively and 670 ± 52 *vs*. 400 ± 27 nmol Ca^2+^/mg of mitochondrial proteins for C110SR and C110, respectively) (Fig. 2E). At the 5 μM concentration and above, both C105SR and C110SR exerted a stronger effect than the maximum effect achieved by CsA (Fig. 2E). At lower concentrations (0.5 and 1 μM), their protective effects were comparable to those of CsA and ALV (Fig. 2E).

Similarly, only compounds C105SR and C110SR were capable to inhibit Ca^2+^-induced mitochondrial swelling (IC_50_ values: 0.009 ± 0.001 μM and 0.04 ± 0.005 μM, respectively, Fig. S4). C105SR was 10-times more potent than C110SR, suggesting the importance of the substitution of the R2 group that differentiates these two compounds (Table 2). The EC_50_ values of C105SR were better than those of CsA and ALV (Table 2). Together, these results demonstrate that only the SR diastereoisomers of compounds C105 and C110 carry their biological activity, with greater mitoprotective properties than the parent compound C31, their racemic mixtures, and the macrocyclic Cyp inhibitors, CsA and ALV.

### Effects of the new SMCypIs on mPTP opening *in vitro*

We next evaluated the ability of compounds C105SR and C110SR to inhibit mPTP opening in cells. The hepatocyte cell line AML-12 was subjected to 4 h of hypoxia followed by 1 h of reoxygenation and mPTP opening was monitored using the CoCl_2_-calcein-acetoxymethyl ester (AM) fluorescence-quenching method.^19^ Calcein-AM is a membrane permeable fluorophore which passively diffuses in all subcellular compartments including mitochondria. In the cell, the AM group of the fluorophore is cleaved by ubiquitous intracellular esterases, generating a hydrophilic product that is retained within the cell. The cells are then loaded with the divalent cobalt cation (Co^2+^), which quenches calcein fluorescence in all subcellular compartments except the mitochondrial matrix, because the inner mitochondrial membrane is impermeable to cobalt. However, when mPTP is open, cobalt enters mitochondria and quenches mitochondrial calcein fluorescence.

**Fig. 3:**
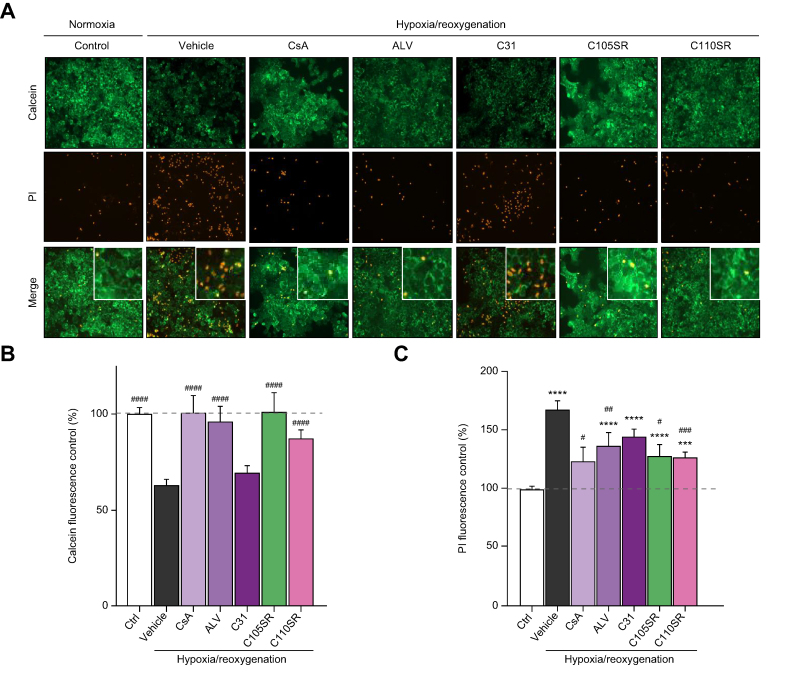
*In vitro* inhibition of mPTP opening and reduction of necrosis by C105SR and C110SR in a model of hepatic hypoxia/reoxygenation. Cells were pretreated with 1 μM calcein and 1 mM CoCl_2_ for 30 min and 10 min, respectively, then subjected to 4 h of hypoxia (1% O_2_) followed by 1 h of reoxygenation (21% O_2_) in the presence of 3 μM propidium iodide (PI). CsA and ALV were used as references. CsA, ALV, C31, C105SR, and C110SR were added at 1 μM for the entire duration of hypoxia/reoxygenation. (A) Representative images of calcein (green) and PI (red) labelling in cells exposed to normoxia (control) or hypoxia/reoxygenation in the absence (vehicle) or in the presence of CsA, ALV, C31, C105SR, or C110SR (original magnification, 400 × ). (B) Calcein fluorescence in cells exposed to normoxia (Ctrl) or hypoxia/reoxygenation in the absence (vehicle) or in the presence of CsA, ALV, C31, C105SR, or C110SR. Data are shown as mean ± SEM. One way ANOVA analysis followed by Tukey’s or Dunnett's post-test if ANOVA produced a significant value of F. ^####^*p* <0.001 *vs*. vehicle. (C) PI fluorescence in cells exposed to normoxia (Ctrl) or hypoxia/reoxygenation in the absence (vehicle) or in the presence of CsA, ALV, C31, C105SR or C110SR. Data are shown as mean ± SEM. One way ANOVA analysis followed by a Tukey’s or a Dunnett's post-test if ANOVA produced a significant value of F. ∗∗∗*p* <0.001 *vs*. Ctrl; ∗∗∗∗*p* <0.0001 *vs*. Ctrl; ^#^*p* <0.05 *vs*. vehicle; ^##^*p* <0.01 *vs*. vehicle; ^###^*p* <0.001 *vs*. vehicle. ALV, alisporivir; CsA, cyclosporin A; mPTP, mitochondrial permeability transition pore.

Cells subjected to hypoxia/reoxygenation exhibited lower calcein fluorescence than normoxic cells, indicating that mPTP opening occurred (Fig. 3A and 3B). This effect was substantially prevented by CsA, ALV, C105SR, and C110SR, but not by C105 and C110 racemic mixtures (Fig. S5), nor by C31 at 1 μM (Fig. 3A and 3B). These results indicate that compounds C105SR and C110SR inhibit mPTP opening at low concentrations in an *in vitro* hypoxia/reoxygenation model.

### Hepatoprotective properties of the new SMCypIs *in vitro*

To assess whether the new SMCypIs reduced mPTP opening-induced cell death, propidium iodide fluorescence was measured in cells that had been co-loaded with calcein-AM. As expected, the loss of calcein fluorescence in cells subjected to hypoxia/reoxygenation was associated with enhanced propidium iodide fluorescence (Fig. 3A and 3C). In contrast, high calcein fluorescence in cells treated with 1 μM CsA, ALV, C105SR or C110SR was associated with low propidium iodide fluorescence (Fig. 3A and 3C). These results suggest that C105SR and C110SR reduce mPTP opening-induced cell death.

**Fig. 4:**
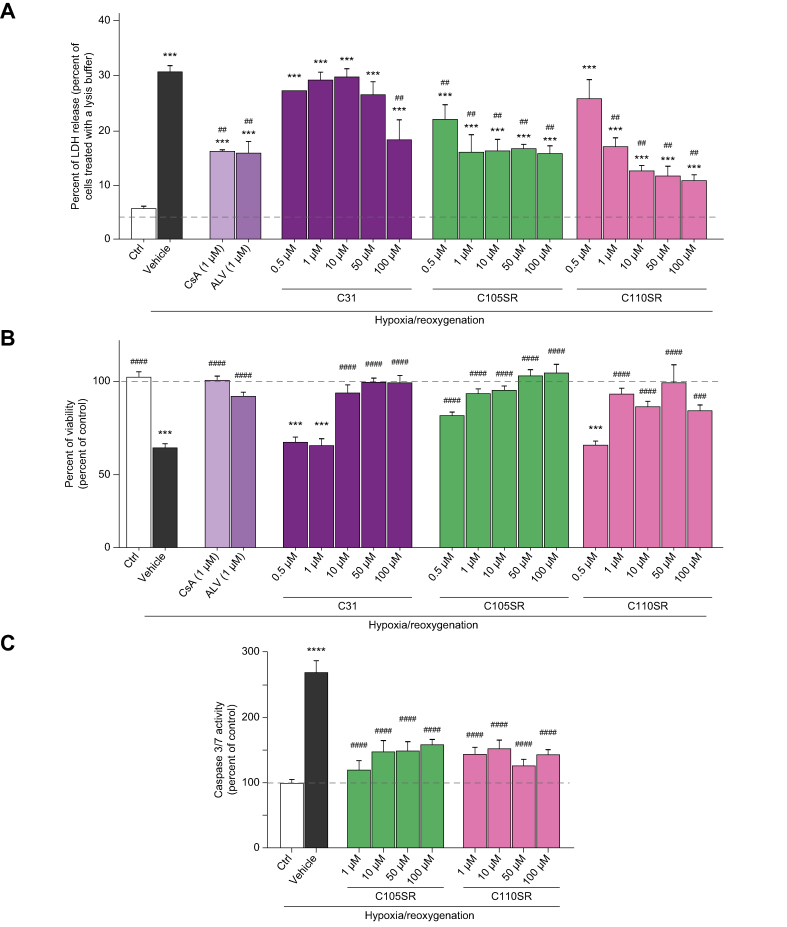
*In vitro* reduction of LDH release and increase in cell viability induced by C105SR and C110SR in a model of hepatic hypoxia/reoxygenation. Cells were subjected to 4 h of hypoxia (1% O_2_) followed by 2 h of reoxygenation (21% O_2_). CsA and ALV were used as references. CsA and ALV were added at 1 μM while C31, C105SR, and C110SR were added at increasing concentrations during the hypoxic and reoxygenation phases. (A) LDH release from cells exposed to normoxia (Ctrl) or hypoxia/reoxygenation in the absence (vehicle) or in the presence of CsA, ALV, or increasing concentrations of C31, C105SR, or C110SR expressed as percentage of LDH release in cells treated with a lysis buffer. Data are shown as mean ± SEM. One way ANOVA analysis followed by Tukey’s or Dunnett's post-test if ANOVA produced a significant value of F. ∗∗∗*p* <0.001 *vs*. Ctrl; ^##^*p* <0.01 *vs*. Vehicle. (B) Cell viability measured by MTT assay in cells exposed to normoxia (Ctrl) or hypoxia/reoxygenation in the absence (vehicle) or in the presence of CsA, ALV, or increasing concentrations of C31, C105SR, or C110SR expressed as percentage of control. Data are shown as mean ± SEM. One-way ANOVA analysis followed by Tukey’s or Dunnett's post-test if ANOVA produced a significant value of F.∗∗∗*p* <0.001 *vs*. Ctrl; ^###^*p* <0.001 *vs*. Vehicle; ^####^*p* <0.0001 *vs*. Vehicle. (C) Caspase 3/7 activity expressed as percentage of control (Ctrl) in cells subjected to 4 h of hypoxia and 2 h of reoxygenation treated with vehicle or increasing concentrations of C105SR or C110SR. Data are shown as mean ± SEM. One way ANOVA analysis followed by Tukey’s or Dunnett's post-test if ANOVA produced a significant value of F ∗∗∗∗*p* <0.0001 *vs*. Ctrl; ^####^*p* <0.0001 *vs*. Vehicle. ALV, alisporivir; CsA, cyclosporin A; LDH, lactate dehydrogenase.

To confirm these results, LDH release was measured in culture media, while cell viability was assessed by means of an MTT assay after 4 h of hypoxia and 2 h of reoxygenation. As expected, cells subjected to hypoxia/reoxygenation were characterised by greater LDH release and reduced cell viability as compared to untreated controls (Fig. 4A and 4B). C105SR and C110SR, applied during hypoxia and reoxygenation, protected against cell death, as shown by the reduced LDH release and the increased cell viability (Fig. 4A and 4B). Both compounds were more efficient than C31, with protective effects comparable to those of CsA and ALV (Fig. 4A and 4B). C105SR showed potent activity inhibiting LDH release and increased cell viability by approximatively 75% at 0.5 μM, whereas C110SR was not effective at this concentration (Fig. 4A and 4B). Similar results were obtained when C105SR and C110SR were applied only during hypoxia (pharmacological preconditioning, Fig. S6) or only during reoxygenation (pharmacological postconditioning, Fig. S7). Together, these results demonstrate that C105SR is the most effective compound in reducing hypoxia/reoxygenation-induced cell death.

Necrosis is the major cause of cell death during hepatic IRI. However, mPTP opening also leads to apoptosis resulting from mitochondrial swelling, rupture of the outer mitochondrial membrane and subsequent release of cytochrome C into the cytosol.^20^ We investigated whether C105SR and C110SR protected against mPTP opening-induced apoptotic cell death. Cells subjected to hypoxia/reoxygenation showed enhanced apoptosis characterised by enhanced caspase 3/7 activity (Fig. 4C). C105SR and C110SR, applied during hypoxia and reoxygenation, protected against hypoxia/reoxygenation-induced cell apoptosis (Fig. 4C).

### Molecular modelling of the interaction of C105SR with CypD

**Fig. 5:**
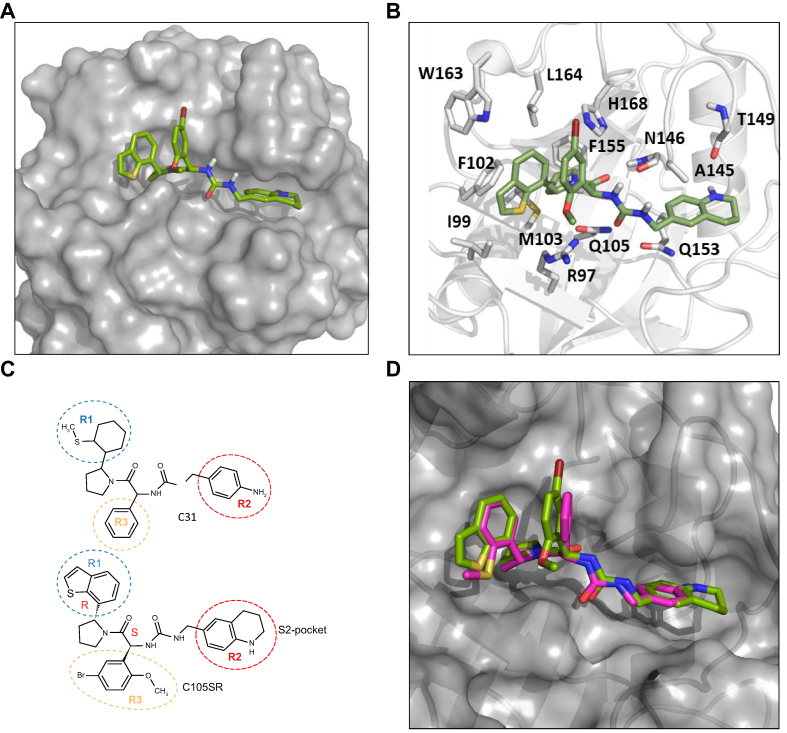
Docking of C31 and C105SR on CypD. CypD, C31, and C105SR are shown in grey, pink, and green, respectively. (A) Large view of C105SR docked on CypD. (B) Zoom view of C105SR docked on CypD showing amino acid interactions. (C) Chemical structures of C31 and C105SR, showing their similar backbones and the differences in R1, R2, and R3 functional regions. (D) Superposition of docking poses of C31 and C105SR on CypD. CypD, cyclophilin D.

The crystal structures of CypD in complex with the ligands were used to dock C31 and the different C105 diastereoisomers using the @TOME-2 server. Comparative docking of ligands co-crystallised with the structure templates confirmed proper modelling of the interactions predicted in the binding sites. The resulting complexes predicted the interaction of C105SR with CypD (Fig. 5A and 5B), whereas the other C105 diastereoisomers were not predicted to properly interact with CypD in keeping with our results showing activity only for diastereoisomer C105SR. The predicted binding mode of C105SR (Fig. 5A and 5B) showed binding to both the PPIase catalytic site and the gatekeeper pocket, with key interactions maintained between CypD and C105SR, including hydrogen bonds with R97, Q105, N146, and T149 and van der Waals interactions with I99, F102, M103, A145, Q153, W163, and L164. C105SR displayed another hydrogen bond between R97 and the methoxy-phenyl moiety (R3), an increase of van der Waals interaction between I99, F102, W163, L164, H168 and the benzothiophene (R1) and the bromo-phenyl (R3) moieties. The binding mode appeared to be similar for C31 and C105SR (Fig. 5C and 5D). Together, these docking experiments validate C105SR as the most effective CypD inhibitor in this study.

### *In vivo* hepatoprotective properties of the new SMCypIs

**Fig. 6:**
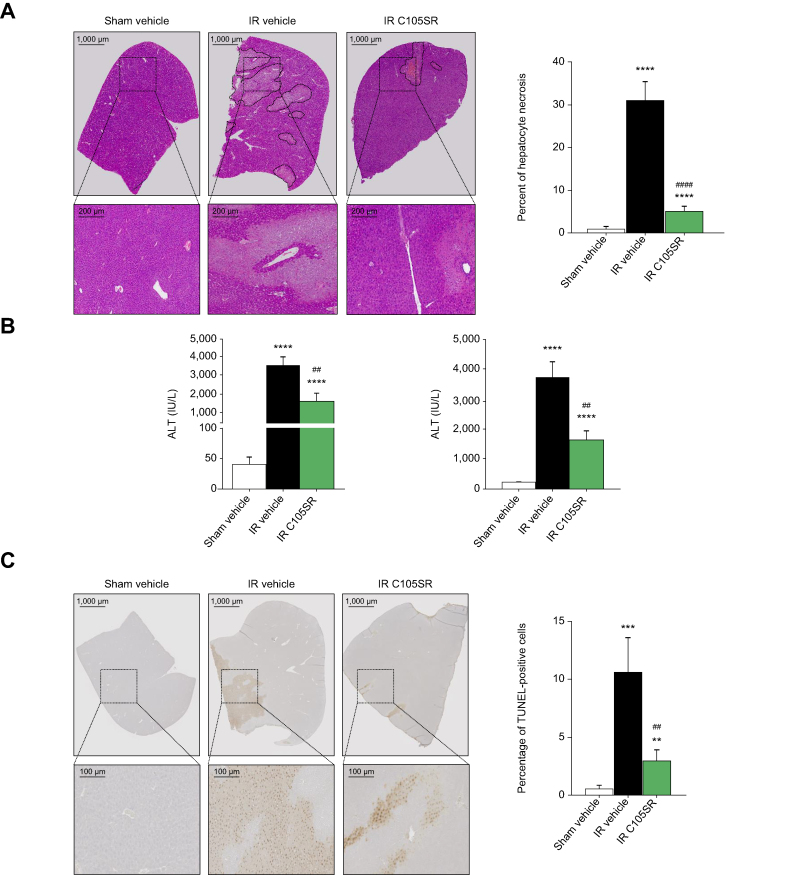
*In vivo* protective effects of C105SR (50 mg/kg) against hepatic ischaemia–reperfusion injury. (A) Representative haematoxylin and eosin staining images (magnification, 200 × ) of liver lobes (left) and percent of hepatocyte necrosis (right) in mice subjected to laparotomy without (sham vehicle) or with ischaemia–reperfusion (IR) in the absence (vehicle) or in the presence of C105SR. n = 8 for sham vehicle; n = 24 for IR vehicle; n = 13 for IR C105SR. Data are shown as mean ± SEM. Mann–Whitney *U* test ∗∗∗∗*p* <0.0001 *vs*. sham vehicle; ^####^*p* <0.0001 *vs*. IR vehicle. (B) Serum ALT and AST levels in mice subjected to laparotomy without (sham vehicle) or with ischaemia–reperfusion (IR) in the absence (vehicle) or in the presence of C105SR. n = 8 for sham vehicle; n = 24 for IR vehicle; n = 13 for IR C105SR. Data are shown as mean ± SEM. Mann–Whitney *U* test. ∗∗∗∗*p* <0.0001 *vs*. sham vehicle; ^##^*p* <0.01 *vs*. IR vehicle (C) Representative TUNEL staining (left) and number of TUNEL-positive cells (right) in livers of mice subjected to laparotomy without (sham vehicle) or with ischaemia–reperfusion (IR) in the absence (vehicle) or in the presence of C105SR. n = 4 for sham vehicle; n = 8 for IR vehicle; n = 8 for IR C105SR. Data are shown as mean ± SEM. Mann–Whitney *U* test ∗∗*p* <0.01 *vs*. sham vehicle; ∗∗∗*p* <0.001 *vs*. sham vehicle; ^##^*p* <0.01 *vs*. IR vehicle. ALT, alanine aminotransferase; AST, aspartate aminotransferase; IR, ischaemia–reperfusion.

The protective effects of the most potent SMCypI derivative, C105SR, were measured in a mouse model of liver IRI. Briefly, 70% of the liver was subjected to 1 h of ischaemia followed by 6 h of reperfusion, as recently described.^16^ C105SR was administered using osmotic pumps at a dose of 50 mg/kg. A tissue distribution study showed that plasmatic and hepatic concentrations of C105SR were around 30 nM (Fig. S8). IRI resulted in a significant increase in hepatocyte necrosis and serum alanine aminotransferase (ALT) and aspartate aminotransferase (AST) levels, as compared with sham-operated mice. As shown in Fig. 6A (histology) and 6B (ALT and AST levels), C105SR significantly protected mouse livers against the effects of ischaemia and reperfusion. Moreover, as shown in Fig. 6C, the number of TUNEL-positive apoptotic cells was significantly reduced in C105SR-treated mice with IRI, as compared with mice treated with the vehicle. Together, these results demonstrate that C105SR bears protective properties against hepatic IRI.

## Discussion

Hepatic IRI induces compromised liver function and leads to frequent graft rejection and represents one of the most challenging liver disorders faced by hepatologists and liver surgeons. Therefore, there is an urgent need to develop effective treatments against hepatic IRI. In this respect, opening of the mPTP has emerged as a critical pathophysiological event mediating cell death during IRI, making it a promising therapeutic target for hepatoprotection. The lack of a clear model of the molecular structure of the mPTP has focused attention on the development of inhibitors of the best characterised mPTP modulator, CypD. However, current Cyp inhibitors are limited by their immunosuppressive properties or off-target effects. Therefore, novel mPTP inhibitors are required to translate this promising therapeutic strategy into clinical practice.

We previously developed a new family of SMCypIs, which exerted mitoprotective and hepatoprotective effects both *in vitro* and *in vivo* in an experimental mouse model of hepatic IRI.^16^ Here, we used structure-guided optimisation to improve the mitoprotective and hepatoprotective properties of our initial best compound C31. We identified a new SMCypI diastereoisomer, C105SR, that was 144- and 182-fold more potent than C31 in inhibiting mitochondrial swelling and increasing mitochondrial CRC, respectively. In addition, C105SR inhibited mPTP opening and cell death at a very low concentration (0.5 μM), whereas C31 was not effective at this concentration.

Molecular modelling of the interaction of C105SR with CypD showed the capability of the compound to interact with the two CypD pockets. The three-dimensional structure predictions identified seven hydrogen bonds and a large number of van der Waals contacts, which could explain the high potency of C105SR against CypD PPIase activity. Docking studies were in keeping with the observation that C105SR was the active diastereoisomer. The use of a single enantiomer of a chiral drug has potential clinical advantages, such as an improved therapeutic index and pharmacological profile, simplified pharmacokinetics, and reduced drug interactions.

Our study showed that C105SR protects against hepatic IRI *in vivo* at a dose of 50 mg/kg, i.e. a lower dose than that used in our previous study (150 mg/kg).^16^ The better efficacy of C105SR at a lower dose could be at least partly explained by the mode of administration. Indeed, in our initial study, C31 was administered by infusion 1 min before and during the first 8 min of reperfusion, whereas in the present study, C105SR was administered using ALZET osmotic pumps implanted 24 h before surgery to achieve a continuous delivery of the compound. C105SR had the capacity to protect cells exposed to hypoxia/reoxygenation injury against cell death when applied during the ischemic and the reoxygenation period. Such pharmacologic preconditioning is transposable to clinical situations in which IRI can be anticipated, such as surgical resection and transplantation. However, it has been well established that mPTP opening occurs mainly at the onset of reperfusion.^21^ We therefore investigated whether C105SR could also protect against death in cells exposed to hypoxia/reoxygenation when applied only during the reoxygenation period. Our data showed similar hepatoprotective effects of C105SR when the compound was applied during the hypoxia period, during the reoxygenation period, or during both (Fig. S3 and S4 and Fig. 4).

An important finding of the present study was that C105SR was more potent than CsA and ALV, two prototypical cyclophilin inhibitors. Indeed, C105SR inhibited CypD PPIase activity and mitochondrial swelling and increased mitochondrial CRC, with EC_50_ values in the nanomolar range. CsA was identified several years ago as a potent inhibitor of Cyp PPIase activity and of mPTP opening.^22^ However, its strong immunosuppressive properties have been a major obstacle to its clinical use in the context of IRI. ALV is a non-immunosuppressive analogue of CsA, developed for the treatment of hepatitis C virus infection, that also protects against IRI.^23^ However, its development was halted in a Phase III clinical trial due to severe side effects, unrelated to its anti-Cyp activity.^24^ CsA derivatives devoid of immunosuppressive properties are macromolecules resulting in poor cell permeability and complex multistep synthesis. These molecules do not bind to the “gatekeeper” pocket of the active site of Cyps. In contrast, C105SR belongs to a new family of CsA-unrelated, non-peptidic, small-molecule Cyp inhibitors, with a molar mass below 700 kDa and easy synthesis. At the structural level, using *in silico* docking, we showed in this study that C105SR binds to the two pockets of CypD, including the PPIase catalytic site and the ‘gatekeeper’ pocket. The ‘gatekeeper’ pocket has been associated with substrate specificity,^25^ suggesting that the mode of binding of our compounds could lead to the synthesis of CypD-specific inhibitors in the future.

In conclusion, we identified a novel SMCypI diastereoisomer, C105SR, with the capacity to potently inhibit mPTP opening and prevent cell death *in vitro* in a model of hypoxia/reoxygenation. This compound also exhibited remarkable activity against hepatic IRI in an *in vivo* murine model. The results of this study provide a solid basis for considering C105SR as a promising candidate drug for hepatocellular protection during IRI. This compound also represents a promising candidate drug for the treatment of other liver and non-hepatic diseases that involve mitochondrial CypD-related mechanisms of cell death.

## Financial support

This work was supported by the 10.13039/501100001677Inserm, the 10.13039/501100009411Université Paris-Est Créteil, a grant of the 10.13039/501100006005Agence de la Biomédecine (21GREFFE014) and a FEDER grant (48748).

## Authors’ contributions

Performed experiments and procedures: AK, NA, AH. Analysis and interpretation of the data: All authors. Concept and design of the study, funding acquisition, study supervision, preparation of original draft: FTC. Review and approval of the final manuscript: All authors.

## Data availability statement

The data that support the findings of this study are available from the corresponding authors, upon reasonable request.

## Conflicts of interest

The authors have no conflict of interest to disclose.

Please refer to the accompanying ICMJE disclosure forms for further details.
