# Supplementary material for: The novel cyclophilin inhibitor C105SR reduces hepatic ischaemia–reperfusion injury via mitoprotection
Source: JHEP Rep. 2023 Aug 16;5(11):100876. doi: 10.1016/j.jhepr.2023.100876 (PMC10582583; doi:10.1016/j.jhepr.2023.100876)
Supplement: Multimedia component 1 [file mmc1.pdf]

The novel cyclophilin inhibitor C105SR reduces hepatic ischaemia-reperfusion injury via mitoprotection

Amel Kheyar, Nazim Ahnou, Abdelhakim Ahmed-Belkacem, Anne Hulin, Claire Pressiat, Bijan Ghaleh, Jean-François Guichou, Didier Morin, Jean-Michel Pawlotsky, Fatima Teixeira-Clerc

Table of contents

Supplementary materials and methods……………………………………………………………………………………………2

Supplementary figures……113

Supplementary materials and methods

Peptidyl-prolyl cis-trans isomerase (PPIase) activity assay. CypD PPIase activity and its inhibition by SMCypIs were measured at 20°C using standard chymotrypsin-coupled assay. The assay buffer (25 mM Hepes, 100 mM NaCl, pH 7.8) and CypD (1.9 µM stock solution) were pre-cooled to 4°C in the presence of SMCypIs, CsA or ALV. Then, 5 µL of 50 mg/mL chymotrypsin in 1 mM HCl was added. The reaction was started by adding 20 µL of 3.2 mM peptide substrate (N-Succinyl-Ala-Ala-Cis-Pro-Phe-p-nitroanilide, AAPF) in lithium chloride/trifluoroethanol (LiCl/TFE) solution with rapid inversion. P-nitroanilide absorbance was measured at 390 nm for 60 sec and absorbance was measured at 1-sec intervals by a spectrophotometer. CsA and ALV were used as positive controls of CypD PPIase activity inhibition in all measurements. For SMCypI PPIase inhibitory activity assessments, 5 µL of different concentrations in DMSO of the tested compound were added to the CypD solution in the assay buffer. The percent inhibition of CypD PPIase activity was calculated from the slopes and the half-maximal inhibitory concentration (IC_{50}) values were obtained from percent inhibition curves using Sigmaplot software (Systat, San Jose, California).

Isolation of mouse liver mitochondria. Mouse livers were cut and homogenized in ice-cold homogenization buffer (220 nM mannitol, 70 mM sucrose, 10 mM HEPES, 4 mM ethylene glycol-bis (β-aminoethyl-ether)-N,N,N',N'-tetraacetic acid (EGTA), pH 7.4 at 4°C) using a Potter-Elvehjem glass homogenizer in a final volume of 10 mL of buffer per g of tissue. The homogenate was centrifuged at 1,000 g for 5 min at 4°C. The supernatant was then transferred to a clean tube and centrifuged at 9,000 g for 10 min at 4°C. The mitochondrial pellet was resuspended in a final volume of 600 µL of homogenization buffer without EGTA. Protein concentration was determined using the advanced protein assay reagent (Sigma).

Calcium retention capacity (CRC) assay. Isolated liver mitochondria (1 mg/mL) energized with 5 mM glutamate/malate were incubated in a buffer allowing respiration of mitochondria (100 mM KCl, 50 mM sucrose, 10 mM HEPES, 5 mM KH_2PO_4, pH 7.4 at 30°C) supplemented with 1 µM calcium green 5N fluorescent probe. Mitochondria were pulsed with sequential additions of $CaCl_2$ (20 µM) until mPTP opening along with rapid calcium release happened. The calcium concentration in the extra-mitochondrial medium was monitored with a Jasco FP-6300 spectrofluorimeter (Jasco, Bouguenais, France) at 506 nm excitation and 532 nm emission wavelengths.

Mitochondrial swelling assay. Mitochondrial swelling was assessed by measuring changes in absorbance at 540 nm using a Jasco V-530 spectrophotometer (Bouguenais, France) equipped with magnetic stirring and thermostatic control (30°C). Isolated liver mitochondria (0.5 mg/mL) were energized with 5 mM of pyruvate/malate. Mitochondria were incubated for 30 sec in the same buffer as CRC assay before the induction of swelling with 100 µM $CaCl_2$. SMCypIs at various concentrations were added before the induction of mitochondrial swelling. CsA and ALV (1 µM) were used as positive controls of mitochondrial swelling inhibition.

Hypoxia/reoxygenation (H/R) in vitro model. AML-12 mouse hepatocyte line was obtained from American Type Culture Collection (ATCC) and maintained in DMEM/F12 culture medium at 37°C with 5% CO_2. HepaRG human hepatocyte line was obtained from American Type Culture Collection (ATCC) and maintained in William'S E culture medium at 37°C with 5% CO_2.When indicated, cells were incubated in phosphate buffered saline at 37°C in a hypoxic chamber filled with 94% N_2, 1% O_2, 5% CO_2 for 4 h to mimic ischemic conditions. Then, cells were transferred to a CO_2 incubator at 37°C in regular DMEM/F12 culture medium for 1 or 2 h under normoxic conditions (74% N_2, 21% O_2, 5% CO_2). SMCypIs or vehicle (DMSO) were added during the hypoxia period, during the reoxygenation period, or during both. CsA and ALV were used as positive controls for hepatic protection against H/R injury.

Monitoring of mPTP opening. mPTP opening was monitored in AML-12 cells using the $CoCl_2$-calcein AM fluorescence-quenching assay. Cells were loaded with 1 µM calcein-AM (green) for 20 min. Then, 1 mM $CoCl_2$ was added and cells were incubated for another 10 min. Cells were washed and subjected to 4 h of hypoxia followed by 1 h of reoxygenation. Calcein fluorescence was measured using a spectrofluorimeter at excitation and emission wavelengths of 485 nm and 535 nm, respectively. Images were acquired using a standard inverted fluorescence microscope (Zeiss Axio, Oberkochen, Germany). SMCypIs, CsA or ALV were added at the concentration of 1 µM in culture media during the entire H/R period. Images shown are representative images of 12-18 images.

Propidium iodide staining. Propidium iodide (PI) was used to identify non-viable cells as it is only permeant to cells with compromised plasma membrane integrity.[1] PI (3 µM) was added to culture media upon the reoxygenation period. Fluorescence was measured using an Infinite 200 Pro fluorescent plate reader (Tecan, Mannedorf, Switzerland) at excitation and emission wavelengths of 533 nm and 617 nm, respectively. Images were acquired using a standard inverted fluorescence microscope (Zeiss Axio, Oberkochen, Germany). Images shown are representative images of 12-18 images.

Cell viability assays. Lactate dehydrogenase (LDH) release in culture medium was assessed using the commercially non-radioactive CytoTox-96 kit (Promega, Madison, Wisconsin) following the manufacturer's instructions. Briefly, cell culture media were collected and the LDH activity was determined by measuring absorbance at 491 nm with a spectrometer (Multiskan skyHigh). For 3-(4,5-dimethylthiazol-2-yl)-2,5-diphenyltetrazolium bromide (MTT) assay, cells were incubated with a solution of MTT (0.5 mg/mL) for 2 h at 37°C. The medium was then removed and 50 µL DMSO was added to each well. Optical density of purple formazan product was measured at 550 nm. Cell viability was expressed as a percentage of the untreated control.

Mouse serum analysis. Blood was collected at 6 h of reperfusion. Alanine aminotransferase (ALT) and aspartate aminotransferase (AST) activities were measured on an automated analyzer in the Biochemistry Department of Henri Mondor Hospital.

Histological Analysis. Hematoxylin and eosin staining was performed on 4-µm thick formalin-fixed paraffin-embedded tissue sections. Slides were scanned at x20 magnification using a virtual slide scanning system (Axio scan7/ZEN slidescan). Necrosis areas were quantified with QuPath.

TUNEL Assay. Terminal deoxynucleotidyl-transferase (TdT) dUTP Nick-End Labeling (TUNEL) was performed on formalin-fixed paraffin-embedded tissue sections using a commercial kit (Abcam, Cambridge, United Kingdom) following the manufacturer's instructions. The number of TUNEL-positive cells were quantified using QuPath on slides scanned at x20 magnification using a virtual slide scanning system (Axio scan7/ZEN slidescan).

Caspase 3/7 activity assay. Caspase 3/7 activity was assessed using the commercially Caspase-Glo® 3/7 assay (Promega, Madison, Wisconsin) following the manufacturer's instructions. Briefly, Caspase-Glo® 3/7 Reagent was incubated with the cells and the caspase3/7 activity was determined by measuring luminescence with a luminometer (Berthold). Caspase3/7 activity was expressed as a percentage of the untreated control.

In silico modeling and docking. The CypD sequence (P30405) was recovered from UniProt (UniProt, http://www.uniprot.org/uniprot/). The search for ligand-CypD 3D crystal complexes was performed using the @TOME-2 server (https://atome.cbs.cnrs.fr/ATOME_V3/index.html).[2] Ligand files were generated with MarvinSketch 6.2.2 for SMILES and Frog2 server for mol2. Docking

simulation was performed using @TOME-2 server with no constraint. The images were generated using PyMOL and MarvinSketch.

Determination of plasmatic and hepatic concentration of C105SR. Mice (10-12 weeks-old, n=6) were anesthetized with isoflurane and subcutaneously implanted with Alzet® osmotic pump containing C105SR (50 mg/kg) or vehicle. 24h after, the blood and the liver were collected. C105SR in plasma and liver homogenates was extracted with cold acetonitrile containing 0.1% of acetic acid and centrifugation at 13,000 x g for 10 min at 4 °C. C105SR dosage was carried out by tandem mass spectrometric detection with a TSQ QUANTIS® mass spectrometer (ThermoFisher Scientific, Villebon-sur-Yvette, France), with an electrospray ionization source (ESI) which was set in positive electro-spray ionization (ESI+). The acquired data were processed using Trace Finder® software version 4.1 (Thermo Fisher Scientific, Villebon-sur-Yvette, France). Optimization of the MS conditions has been performed by single direct infusion of reference standards of the analyte. Two ion transitions were used per analyte (633.17/146.14 and 444.85).

References

1. Brana C, Benham C, Sundstrom L. A method for characterising cell death *in vitro* by combining propidium iodide staining with immunohistochemistry. Brain Res Brain Res Protoc. 2002 Oct;10(2):109-14.

2. Pons JL, Labesse G. @TOME-2: a new pipeline for comparative modeling of protein-ligand complexes. Nucleic Acids Res. 2009;37(Web Server issue):W485-91.

Synthesis and the characterization of the compounds

<u>Equipment and analytical methods used for the syntheses of examples:</u>

Microwaves irradiation: Apparatus: CEM Discover with Synergy Software.

Method: 10 mL or 30 mL sealed tube, power up to 50W, high stirring.

Flash chromatography:

Apparatus: Biotage SP with auto-collector and UV detection (2 wavelengths).

Normal phase columns: 10, 25 or 120 g Biotage external dry load cartridge kit, packed with Sigma-Aldrich 40-63 μm silica gel.

Reverse phase column: 30 g Biotage SNAP Cartridges, KP-C18-HS.

Chiral column: Daicel ChiralFlash IG 100 x 30 mm 20 μM.

Liquid Chromatography:HPLC

Apparatus: Waters alliance 2695 HPLC system with autosampler and Waters 2996 diode array detector.

Reverse phase conditions:

Column: Macherey-Nagel Nucleoshell RP18 plus (5 μm, 4 mm x 100 mm).

Column temperature: 40°C.

Solvents: A (H_2O 99.9%, H_2CO_2 0.1%); B (MeCN 99.9%, H_2CO_2 0.1%).

Flow rate: 1mL/min.

Gradient (A/B v/v): 95/5 (t=0min), 95/5 (t=1min), 0/100 (t=7min), 0/100 (t=10min).

Chiral phase conditions:

Column: Daicel ChiralPak IG (Amylose-based) 20 μm, 4.6 mm x 250 mm.

Column temperature: 25°C.

Solvents: EtOH containing 0.1% TEA 90%/DCM 10%.

Flow rate: 1mL/min.

Run time: 30min

Mass Spectrometer:

Apparatus: Waters Micromass ZQ (simple quad).

Mass detection method: Electrospray positive mode (ESI+), mass range: 50-800 uma.

Detection: 210-400nm range.

NMR Spectrometer:

Apparatus: Bruker 400 MHz.

<u>Example 1: Synthesis of 1-(4-aminobenzyl)-3-(1-(2,5-dimethoxyphenyl)-2-(2-(2-(methylthio)phenyl)pyrrolidin-1-yl)-2-oxoethyl)urea: C90</u>

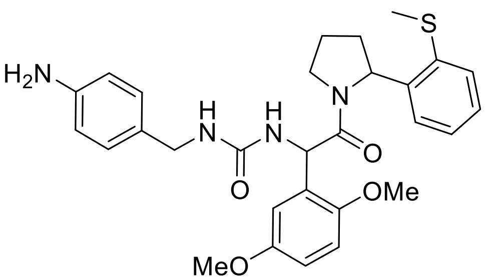

Step 1: methyl 2-amino-2-(2,5-dimethoxyphenyl)acetate

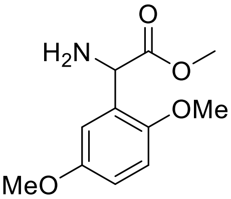

250 mg (1.18 mmol, 1 eq) of 2-amino-2-(2,5-dimethoxyphenyl)acetic acid are dissolved in 3 mL of dry MeOH under Argon. The solution is cooled to 0°C and 90 µL (1.42 mmol, 1.2 eq) of $SOCl_2$ are added. The mixture is stirred 5 min at this temperature and then 2h at reflux. Reaction is diluted with EtOAc and washed 3 times with a saturated $NaHCO_3$ solution. Aqueous layer is extracted 3 times with EtOAc. Combined organic layers are dried over Na_2SO_4, filtered and evaporated unde reduced pressure to give 162 mg of a yellow oil.

Yield : 61%

MH+ : 226.3 (M+1)

Step 2: methyl 2-((tert-butoxycarbonyl)amino)-2-(2,5-dimethoxyphenyl)acetate

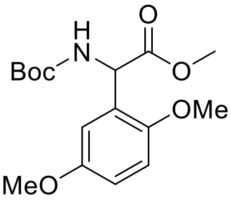

162 mg (0.72 mmol, 1 eq) of methyl 2-amino-2-(2,5-dimethoxyphenyl)acetate (described in previous step) are dissolved in 3 mL of dry THF under Argon. 164 mg (0.76 mmol, 1.05 eq) of Boc_2O are added and the solution is stirred 1h at room temperature. Reaction is diluted with EtOAc and washed 2 times with a saturated $NaHCO_3$ solution. Organic layer is dried over Na_2SO_4, filtered and evaporated unde reduced pressure. Crude product is purified by flash chromatography using a silica gel column and an Hexane/EtOAc mixture as eluent. 215 mg of the title compound are obtained as a pale yellow oil.

Yield : 92%

MH+ : 326.5 (M+1)

Step 3: 2-((tert-butoxycarbonyl)amino)-2-(2,5-dimethoxyphenyl)acetic acid

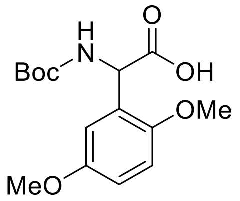

215 mg (0.66 mmol, 1 eq) of methyl 2-((tert-butoxycarbonyl)amino)-2-(2,5-dimethoxyphenyl)acetate (described in previous step) are dissolved in 6 mL of H_2O/MeOH solution (1/1, v/v). 58 mg (1.45 mmol, 2.2 eq) of NaOH are added and the solution is heated 1h at 70°C. Reaction is diluted with water and pH is adjusted to 9 with NaOH 2N. Aqueous layer is extracted 3 times with EtOAc, then aqueous layer pH is acidified with HCl 2N to reached a value of 2. Acidic aqueous layer is extracted 3 times with EtOAc and this organic layer is dried over Na_2SO_4, filtered and evaporated unde reduced pressure to give 168 mg of a yellow oil.

Yield : 82%

MH+ : 312.5 (M+1)

Step 4: tert-butyl (1-(2,5-dimethoxyphenyl)-2-(2-(2-(methylthio)phenyl)pyrrolidin-1-yl)-2-oxoethyl)carbamate

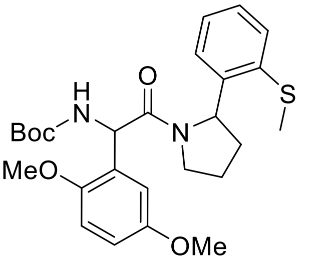

84 mg (0.27 mmol, 1 eq) of 2-((tert-butoxycarbonyl)amino)-2-(2,5-dimethoxyphenyl)acetic acid (described in previous step), 52 mg (0.27 mmol, 1 eq) of 2-(2-(methylthio)phenyl)pyrrolidine and 280 µL (1.62 mmol, 6 eq) DIEA are dissolved in 2 mL of dry DCM under Argon. 62 mg (0.32 mmol, 1.2 eq) of EDC and 44 mg (0.32 mmol, 1.2 eq) of HOBt are added and the solution is stirred overnight at room temperature. Reaction is diluted with EtOAc and washed one time with water, 2 times with a saturated $NaHCO_3$ solution and one time with brine. Organic layer is dried over Na_2SO_4, filtered and evaporated unde reduced pressure. Crude product is purified by flash chromatography using a silica gel column and an DCM/MeOH mixture as eluent. 140 mg of the title compound is obtained as a pale yellow oil.

Yield : quant.

MH+ : 487.7 (M+1)

Step 5: 2-amino-2-(2,5-dimethoxyphenyl)-1-(2-(2-(methylthio)phenyl)pyrrolidin-1-yl)ethan-1-one

131 mg (0.27 mmol, 1 eq) of tert-butyl (1-(2,5-dimethoxyphenyl)-2-(2-(2-(methylthio)phenyl)pyrrolidin-1-yl)-2-oxoethyl)carbamate (described in previous step) is dissolved in 1.5 mL of dry DCM under Argon. 1.5 mL of TFA is added and the solution is stirred 1h at room temperature. Reaction is evaporated under reduced pressure, diluted with a saturated $NaHCO_3$ solution and extracted 3 times with DCM. Combined organic layers are dried over Na_2SO_4, filtered and evaporated unde reduced pressure. Crude product is purified by flash chromatography using a silica gel column and an DCM/MeOH mixture as eluent. 63 mg of the title compound is obtained as a pale yellow oil.

Yield : 60%

MH+ : 387.5 (M+1)

Step 6: tert-butyl (4-((3-(1-(2,5-dimethoxyphenyl)-2-(2-(2-(methylthio)phenyl)pyrrolidin-1-yl)-2-oxoethyl)ureido)methyl)phenyl)carbamate

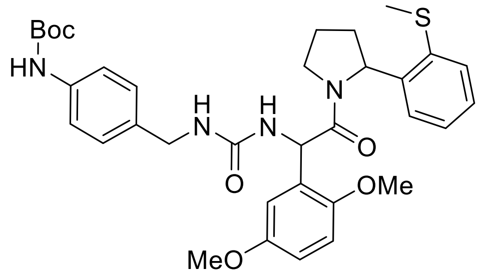

71 mg (0.32 mmol, 1 eq) of tert-butyl (4-(aminomethyl)phenyl)carbamate is dissolved in 2 mL of dry DCM under Argon. 55 µL (0.32 mmol, 1 eq) of DIEA and 52 mg (0.32 mmol, 1 eq) of CDI are added and the solution is stirred 15min at room temperature. 124 mg (0.32 mmol, 1 eq) of 2-amino-2-(2,5-dimethoxyphenyl)-1-(2-(2-(methylthio)phenyl)pyrrolidin-1-yl)ethan-1-one (described in previous step) dissolved in 2 mL of dry DCM is added. The solution is heated at 40°C overnight. Reaction is diluted with EtOAc and washed 3 times with water. Organic layer is dried over Na_2SO_4, filtered and evaporated unde reduced pressure. Crude product is purified by flash chromatography using a silica gel column and an DCM/MeOH mixture as eluent. 150 mg of the title compound is obtained as a pale yellow oil.

Yield : 74%

MH+ : 635.6 (M+1)

Step 7: 1-(4-aminobenzyl)-3-(1-(2,5-dimethoxyphenyl)-2-(2-(2-(methylthio)phenyl)pyrrolidin-1-yl)-2-oxoethyl)urea C90

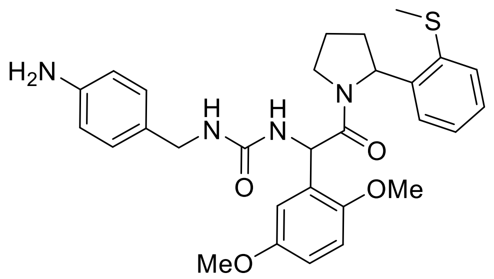

150 mg (0.23 mmol, 1 eq) of tert-butyl (4-((3-(1-(2,5-dimethoxyphenyl)-2-(2-(2-(methylthio)phenyl)pyrrolidin-1-yl)-2-oxoethyl)ureido)methyl)phenyl)carbamate (described in the previous step) is dissolved in 2 mL of dry DCM under Argon. Then 1 mL of TFA is added and the solution is stirred 1h at room temperature. Reaction is evaporated under reduced pressure, diluted with a saturated $NaHCO_3$ solution and extracted 3 times with DCM. Combined organic layers are dried over Na_2SO_4, filtered and evaporated unde reduced pressure. Crude product is purified by flash chromatography using a C18 column and an H_2O/MeOH mixture as eluent. 70 mg of the title compound is obtained as a pale yellow powder.

Yield : 55%

MH+ : 535.6 (M+1)

Example 2: Synthesis of 1-(1-(2,5-dimethoxyphenyl)-2-(2-(2-(methylthio)phenyl)pyrrolidin-1-yl)-2-oxoethyl)-3-((1,2,3,4-tetrahydroquinolin-6-yl)methyl)urea: C173

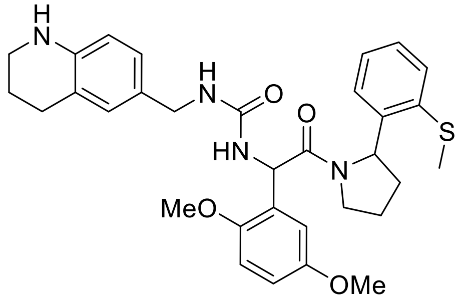

Step 1: tert-butyl 6-((3-(1-(2,5-dimethoxyphenyl)-2-(2-(2-(methylthio)phenyl)pyrrolidin-1-yl)-2-oxoethyl)ureido)methyl)-3,4-dihydroquinoline-1(2H)-carboxylate

The compound is obtained by the procedure described in Example 1, Step 6, starting from 74 mg (0.19 mmol) of 2-amino-2-(2,5-dimethoxyphenyl)-1-(2-(2-(methylthio)phenyl)pyrrolidin-1-yl)ethan-1-one (described in Example 1, Step 5) and tert-butyl 6-(aminomethyl)-3,4-dihydroquinoline-1(2H)-carboxylate instead of tert-butyl (4-(aminomethyl)phenyl)carbamate. The title compound was directly engaged in next step.

Yield: 52%

MH+ : 675.6 (M+1)

Step 2: 1-(1-(2,5-dimethoxyphenyl)-2-(2-(2-(methylthio)phenyl)pyrrolidin-1-yl)-2-oxoethyl)-3-((1,2,3,4-tetrahydroquinolin-6-yl)methyl)urea

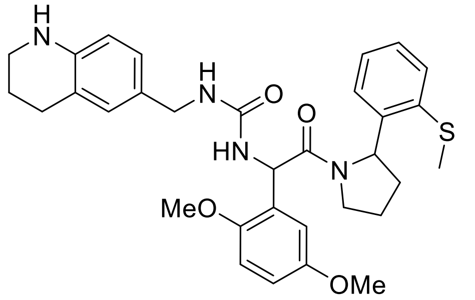

The compound is obtained by the procedure described in Example 1, Step 7, starting from crude of tert-butyl 6-((3-(1-(2,5-dimethoxyphenyl)-2-(2-(2-(methylthio)phenyl)pyrrolidin-1-yl)-2-oxoethyl)ureido)methyl)-3,4-dihydroquinoline-1(2H)-carboxylate (described in the previous step). 6 mg of the title compound are obtained as a white powder.

Yield: 10%

MH+ : 575.5 (M+1)

<u>Example 3: Synthesis of 1-((1H-indol-4-yl)methyl)-3-(1-(2,5-dimethoxyphenyl)-2-(2-(2-(methylthio)phenyl)pyrrolidin-1-yl)-2-oxoethyl)urea: C170</u>

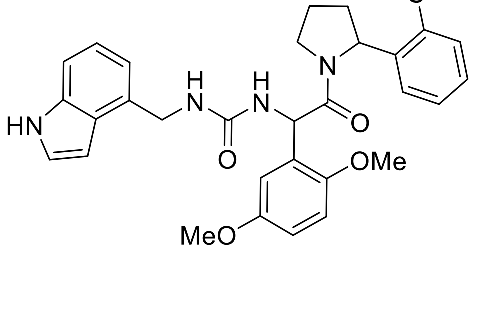

Step 1: 1-((1H-indol-4-yl)methyl)-3-(1-(2,5-dimethoxyphenyl)-2-(2-(2-(methylthio)phenyl)pyrrolidin-1-yl)-2-oxoethyl)urea

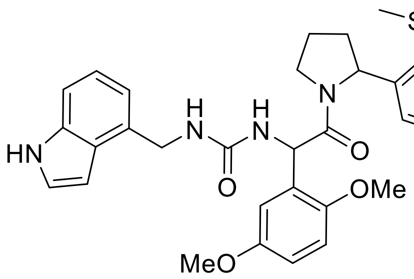

The compound is obtained by the procedure described in Example 1, Step 6, starting from 132 mg (0.34 mmol) of 2-amino-2-(2,5-dimethoxyphenyl)-1-(2-(2-(methylthio)phenyl)pyrrolidin-1-yl)ethan-1-one (described in Example 1, Step 5) and (1H-indol-4-yl)methanamine instead of tert-butyl (4-(aminomethyl)phenyl)carbamate. 32 mg of the title compound are obtained as a white powder.

Yield: 17%

MH+ : 559.3 (M+1)

Example 4: Synthesis of 1-((1H-indazol-4-yl)methyl)-3-(1-(2,5-dimethoxyphenyl)-2-(2-(2-(methylthio)phenyl)pyrrolidin-1-yl)-2-oxoethyl)urea: C171

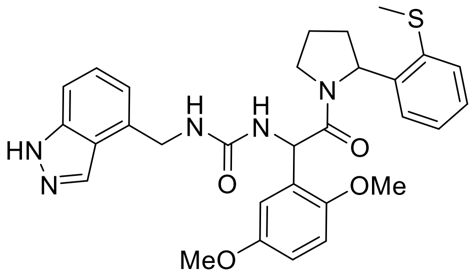

Step 1: 1-((1H-indazol-4-yl)methyl)-3-(1-(2,5-dimethoxyphenyl)-2-(2-(2-(methylthio)phenyl)pyrrolidin-1-yl)-2-oxoethyl)urea

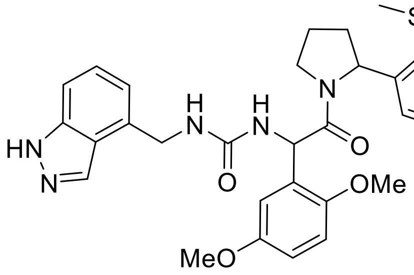

The compound is obtained by the procedure described in Example 1, Step 6, starting from 130 mg (0.34 mmol) of 2-amino-2-(2,5-dimethoxyphenyl)-1-(2-(2-(methylthio)phenyl)pyrrolidin-1-yl)ethan-1-one (described in Example 1, Step 5) and (1H-indazol-4-yl)methanamine instead of tert-butyl (4-(aminomethyl)phenyl)carbamate. 5 mg of the title compound are obtained as a white powder.

Yield: 3%

MH+ : 560.3 (M+1)

<u>Example 5: Synthesis of 1-(4-aminobenzyl)-3-(2-(2-(benzo[b]thiophen-7-yl)pyrrolidin-1-yl)-1-(2,5-dimethoxyphenyl)-2-oxoethyl)urea:F834 C108</u>

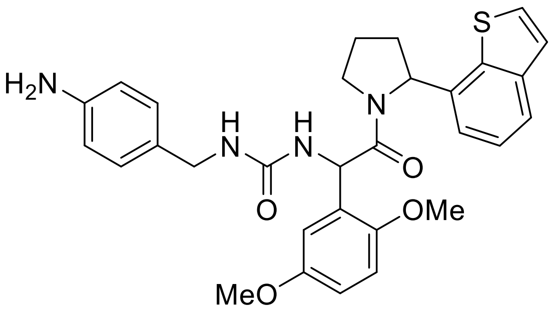

Step 1: tert-butyl (2-(2-(benzo[b]thiophen-7-yl)pyrrolidin-1-yl)-1-(2,5-dimethoxyphenyl)-2-oxoethyl)carbamate

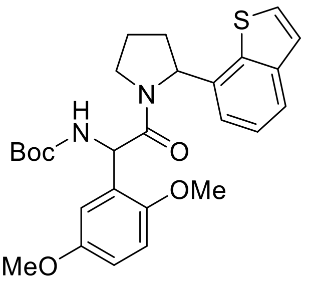

The compound is obtained by the procedure described in Example 1, Step 4, starting from 540 mg (1.73 mmol) of 2-((tert-butoxycarbonyl)amino)-2-(2,5-dimethoxyphenyl)acetic acid (described in Example 1, Step 3) and 2-(benzo[b]thiophen-7-yl)pyrrolidine instead of 2-(2-(methylthio)phenyl)pyrrolidine. 737 mg of the title compound are obtained as a white oil.

Yield: 86%

MH+ : 497.6 (M+1)

Step 2: 2-amino-1-(2-(benzo[b]thiophen-7-yl)pyrrolidin-1-yl)-2-(2,5-dimethoxyphenyl)ethan-1-one

The compound is obtained by the procedure described in Example 1, Step 5, starting from 700 mg (1.41 mmol) of tert-butyl (2-(2-(benzo[b]thiophen-7-yl)pyrrolidin-1-yl)-1-(2,5-

dimethoxyphenyl)-2-oxoethyl)carbamate (described in previous step). 348 mg of the title compound are obtained as a white powder.

Yield: 62%

MH+ : 397.5 (M+1)

Step 3: tert-butyl (4-((3-(2-(2-(benzo[b]thiophen-7-yl)pyrrolidin-1-yl)-1-(2,5-dimethoxyphenyl)-2-oxoethyl)ureido)methyl)phenyl)carbamate

The compound is obtained by the procedure described in Example 1, Step 6, starting from 170 mg (0.428 mmol) of 2-amino-1-(2-(benzo[b]thiophen-7-yl)pyrrolidin-1-yl)-2-(2,5-dimethoxyphenyl)ethan-1-one (described in previous step). 52 mg of the title compound are obtained as a white powder.

Yield: 19%

MH+ : 645.6 (M+1)

Step 4: 1-(4-aminobenzyl)-3-(2-(2-(benzo[b]thiophen-7-yl)pyrrolidin-1-yl)-1-(2,5-dimethoxyphenyl)-2-oxoethyl)urea

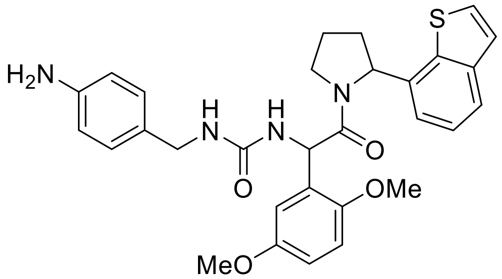

The compound is obtained by the procedure described in Example 1, Step 7, starting from 52 mg (0.08 mmol) of tert-butyl (4-((3-(2-(2-(benzo[b]thiophen-7-yl)pyrrolidin-1-yl)-1-(2,5-dimethoxyphenyl)-2-oxoethyl)ureido)methyl)phenyl)carbamate (described in previous step). 32 mg of the title compound are obtained as a white powder.

Yield: 73%

MH+ : 545.6 (M+1)

<u>Example 6: Synthesis of 1-(4-aminobenzyl)-3-(2-(2-(benzo[b]thiophen-7-yl)pyrrolidin-1-yl)-1-(5-bromo-2,4-dimethoxyphenyl)-2-oxoethyl)urea: C112</u>

Step 1: methyl 2-amino-2-(5-bromo-2,4-dimethoxyphenyl)acetate

The compound is obtained by the procedure described in Example 1, Step 1, starting from 500 mg (1.7 mmol) of 2-amino-2-(5-bromo-2,4-dimethoxyphenyl)acetic acid instead of 2-amino-2-(2,5-dimethoxyphenyl)acetic acid. 335 mg of the title compound are obtained as a pale yellow oil.

Yield: 64%.

MH+ : 304.3-306.3 (M+1)

Step 2: methyl 2-(5-bromo-2,4-dimethoxyphenyl)-2-((tert-butoxycarbonyl)amino)acetate

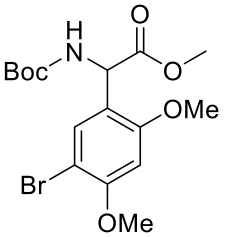

The compound is obtained by the procedure described in Example 1, Step 2, starting from 335 mg (1.1 mmol) of methyl 2-amino-2-(5-bromo-2,4-dimethoxyphenyl)acetate (described in the previous step). 432 mg of the title compound are obtained as a yellow solid foam.

Yield: 97%.

MH+ : 404.4-406.5 (M+1)

Step 3: 2-(5-bromo-2,4-dimethoxyphenyl)-2-((tert-butoxycarbonyl)amino)acetic acid

The compound is obtained by the procedure described in Example 1, Step 3, starting from 400 mg (0.99 mmol) of methyl 2-(5-bromo-2,4-dimethoxyphenyl)-2-((tert-butoxycarbonyl)amino)acetate (described in the previous step). 437 mg of the title compound are obtained as a white solid foam.

Yield: quant.

MH+ : 390.4-392.4 (M+1)

Step 4: tert-butyl (2-(2-(benzo[b]thiophen-7-yl)pyrrolidin-1-yl)-1-(5-bromo-2,4-dimethoxyphenyl)-2-oxoethyl)carbamate

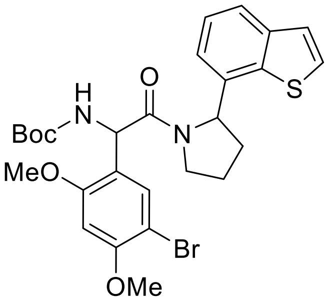

The compound is obtained by the procedure described in Example 1, Step 4, starting from 208 mg (0.53 mmol) of 2-(5-bromo-2,4-dimethoxyphenyl)-2-((tert-butoxycarbonyl)amino)acetic acid (described in the previous step) and 2-(benzo[b]thiophen-7-yl)pyrrolidine instead of 2-(2-(methylthio)phenyl)pyrrolidine. 226 mg of the title compound are obtained as a white solid foam.

Yield: 74%

MH+ : 575.5-577.5 (M+1)

Step 5: 2-amino-1-(2-(benzo[b]thiophen-7-yl)pyrrolidin-1-yl)-2-(5-bromo-2,4-dimethoxyphenyl)ethan-1-one

The compound is obtained by the procedure described in Example 1, Step 5, starting from 226 mg (0.39 mmol) of tert-butyl (2-(2-(benzo[b]thiophen-7-yl)pyrrolidin-1-yl)-1-(5-bromo-2,4-dimethoxyphenyl)-2-oxoethyl)carbamate (described in the previous step). 129 mg of the title compound are obtained as a white solid foam.

Yield: quant.

MH+ : 475.4-477.4 (M+1)

Step 6: tert-butyl (4-((3-(2-(2-(benzo[b]thiophen-7-yl)pyrrolidin-1-yl)-1-(5-bromo-2,4-dimethoxyphenyl)-2-oxoethyl)ureido)methyl)phenyl)carbamate

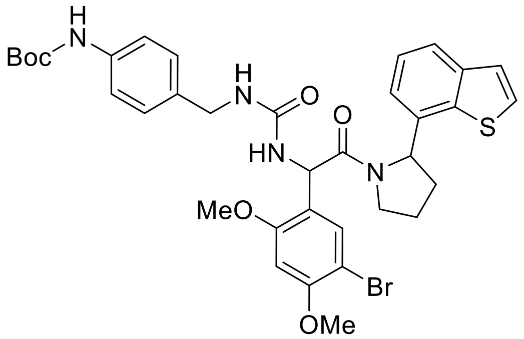

The compound is obtained by the procedure described in Example 1, Step 6, starting from 129 mg (0.27 mmol) of 2-amino-1-(2-(benzo[b]thiophen-7-yl)pyrrolidin-1-yl)-2-(5-bromo-2,4-dimethoxyphenyl)ethan-1-one (described in the previous step). 164 mg of the title compound are obtained as a white solid.

Yield: 84%

MH+ : 723.5-725.5 (M+1)

Step 7: 1-(4-aminobenzyl)-3-(2-(2-(benzo[b]thiophen-7-yl)pyrrolidin-1-yl)-1-(5-bromo-2,4-dimethoxyphenyl)-2-oxoethyl)urea

The compound is obtained by the procedure described in Example 1, Step 7, starting from 164 mg (0.23 mmol) of tert-butyl (4-((3-(2-(2-(benzo[b]thiophen-7-yl)pyrrolidin-1-yl)-1-(5-bromo-2,4-dimethoxyphenyl)-2-oxoethyl)ureido)methyl)phenyl)carbamate (described in the previous step). 47 mg of the title compound are obtained as an off white solid.

Yield: 33%

MH+ : 623.6-625.5 (M+1)

<u>Example 7: Synthesis of 1-(4-aminobenzyl)-3-(2-(2-(benzo[b]thiophen-7-yl)pyrrolidin-1-yl)-1-cyclohexyl-2-oxoethyl)urea: C107</u>

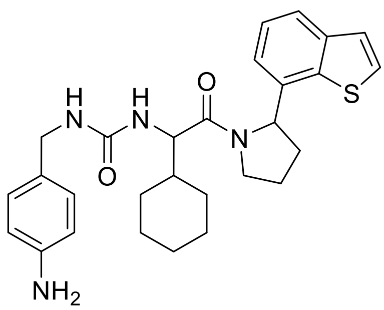

Step 1: tert-butyl (2-(2-(benzo[b]thiophen-7-yl)pyrrolidin-1-yl)-1-cyclohexyl-2-oxoethyl)carbamate

The compound is obtained by the procedure described in Example 1, Step 4, starting from 100 mg (0.39 mmol) of 2-((tert-butoxycarbonyl)amino)-2-cyclohexylacetic acid instead of 2-((tert-butoxycarbonyl)amino)-2-(2,5-dimethoxyphenyl)acetic acid and 2-(benzo[b]thiophen-7-yl)pyrrolidine instead of 2-(2-(methylthio)phenyl)pyrrolidine. 177 mg of the title compound are obtained as a colorless oil.

Yield: quant.

MH+ : 443.7 (M+1)

Step 2: 2-amino-1-(2-(benzo[b]thiophen-7-yl)pyrrolidin-1-yl)-2-cyclohexylethan-1-one

The compound is obtained by the procedure described in Example 1, Step 5, starting from 172 mg (0.39 mmol) of tert-butyl (2-(2-(benzo[b]thiophen-7-yl)pyrrolidin-1-yl)-1-cyclohexyl-2-oxoethyl)carbamate (described in the previous step). 89 mg of the title compound are obtained as a yellow oil.

Yield: 67%

MH+ : 343.8 (M+1)

Step 3: tert-butyl (4-((3-(2-(2-(benzo[b]thiophen-7-yl)pyrrolidin-1-yl)-1-cyclohexyl-2-oxoethyl)ureido)methyl)phenyl)carbamate

The compound is obtained by the procedure described in Example 1, Step 6, starting from 89 mg (0.26 mmol) of 2-amino-1-(2-(benzo[b]thiophen-7-yl)pyrrolidin-1-yl)-2-cyclohexylethan-1-one (described in the previous step). 157 mg of the title compound are obtained as a white solid.

Yield: quant.

MH+ : 591.7 (M+1)

Step 4: 1-(4-aminobenzyl)-3-(2-(2-(benzo[b]thiophen-7-yl)pyrrolidin-1-yl)-1-cyclohexyl-2-oxoethyl)urea

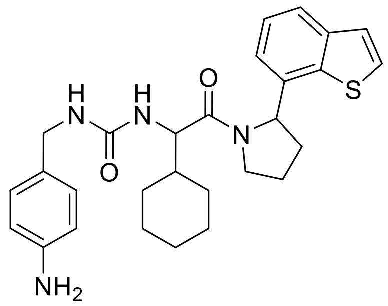

The compound is obtained by the procedure described in Example 1, Step 7, starting from 157 mg (0.26 mmol) of tert-butyl (4-((3-(2-(2-(benzo[b]thiophen-7-yl)pyrrolidin-1-yl)-1-cyclohexyl-2-oxoethyl)ureido)methyl)phenyl)carbamate (described in the previous step). 88 mg of the title compound are obtained as a slightly yellow solid.

Yield: 69%

MH+ : 491.6 (M+1)

Example 8: Synthesis of 1-(2-(2-(benzo[b]thiophen-7-yl)pyrrolidin-1-yl)-1-cyclohexyl-2-oxoethyl)-3-((1,2,3,4-tetrahydroquinolin-6-yl)methyl)urea: C106

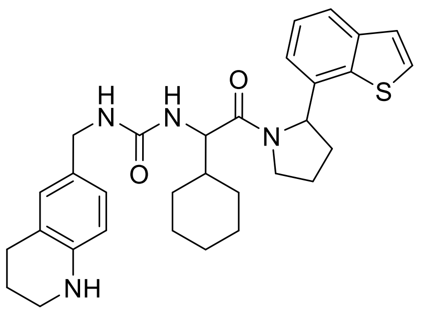

Step 1: tert-butyl 6-((3-(2-(2-(benzo[b]thiophen-7-yl)pyrrolidin-1-yl)-1-cyclohexyl-2-oxoethyl)ureido)methyl)-3,4-dihydroquinoline-1(2H)-carboxylate

The compound is obtained by the procedure described in Example 1, Step 6, starting from 459 mg (1.75 mmol) of 2-amino-1-(2-(benzo[b]thiophen-7-yl)pyrrolidin-1-yl)-2-cyclohexylethan-1-one (described in Example 8, Step 2). 1.05 g of the title compound are obtained as a colorless gel.

Yield: 95%

MH+ : 631.8 (M+1)

Step 2: 1-(2-(2-(benzo[b]thiophen-7-yl)pyrrolidin-1-yl)-1-cyclohexyl-2-oxoethyl)-3-((1,2,3,4-tetrahydroquinolin-6-yl)methyl)urea

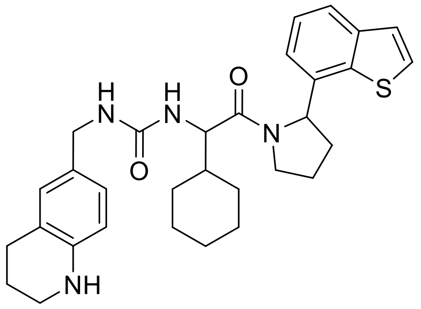

The compound is obtained by the procedure described in Example 1, Step 7, starting from 1.05 g (1.66 mmol) of tert-butyl 6-((3-(2-(2-(benzo[b]thiophen-7-yl)pyrrolidin-1-yl)-1-cyclohexyl-2-oxoethyl)ureido)methyl)-3,4-dihydroquinoline-1(2H)-carboxylate (described in the previous step). 515 mg of the title compound are obtained as a white powder.

Yield: 58%

MH+ : 531.8 (M+1)

Example 9: Synthesis of 1-(4-aminobenzyl)-3-(1-cyclohexyl-2-(2-(2-(methylthio)phenyl)pyrrolidin-1-yl)-2-oxoethyl)urea: C71

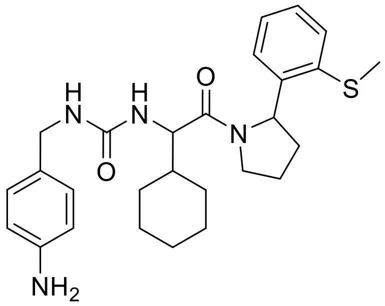

Step 1: tert-butyl (1-cyclohexyl-2-(2-(2-(methylthio)phenyl)pyrrolidin-1-yl)-2-oxoethyl)carbamate

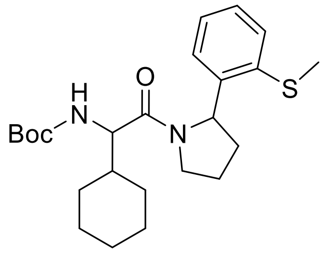

The compound is obtained by the procedure described in Example 1, Step 4, starting from 148 mg (0.58 mmol) of 2-((tert-butoxycarbonyl)amino)-2-cyclohexylacetic acid instead of 2-((tert-butoxycarbonyl)amino)-2-(2,5-dimethoxyphenyl)acetic acid. 125 mg of the title compound are obtained as a white powder.

Yield: 51%

MH+ : 432.5 (M+1)

Step 2: 2-amino-2-cyclohexyl-1-(2-(2-(methylthio)phenyl)pyrrolidin-1-yl)ethan-1-one

The compound is obtained by the procedure described in Example 1, Step 5, starting from 121 mg (0.28 mmol) of tert-butyl (1-cyclohexyl-2-(2-(2-(methylthio)phenyl)pyrrolidin-1-yl)-2-oxoethyl)carbamate (described in the previous step). 86 mg of the title compound are obtained as a white powder.

Yield: 92%

MH+ : 332.5 (M+1)

Step 3: tert-butyl (4-((3-(1-cyclohexyl-2-(2-(2-(methylthio)phenyl)pyrrolidin-1-yl)-2-oxoethyl)ureido)methyl)phenyl)carbamate

The compound is obtained by the procedure described in Example 1, Step 6, starting from 79 mg (0.195 mmol) of 2-amino-2-cyclohexyl-1-(2-(2-(methylthio)phenyl)pyrrolidin-1-yl)ethan-1-one (described in the previous step). 91 mg of the title compound are obtained as a white powder.

Yield: 80%

MH+ : 581.8 (M+1)

Step 4: 1-(4-aminobenzyl)-3-(1-cyclohexyl-2-(2-(2-(methylthio)phenyl)pyrrolidin-1-yl)-2-oxoethyl)urea

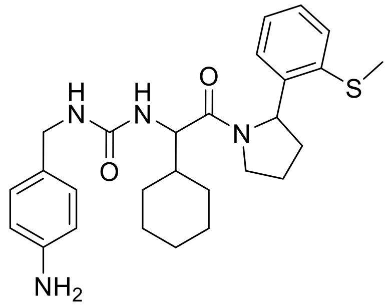

The compound is obtained by the procedure described in Example 1, Step 7, starting from 90 mg (0.155 mmol) of tert-butyl (4-((3-(1-cyclohexyl-2-(2-(2-(methylthio)phenyl)pyrrolidin-1-yl)-2-oxoethyl)ureido)methyl)phenyl)carbamate (described in the previous step). 38 mg of the title compound are obtained as a white powder.

Yield: 51%

MH+ : 481.6 (M+1)

Example 10: Synthesis of 1-(indolin-4-ylmethyl)-3-(2-(2-(2-(methylthio)phenyl)pyrrolidin-1-yl)-2-oxo-1-phenylethyl)urea: C144

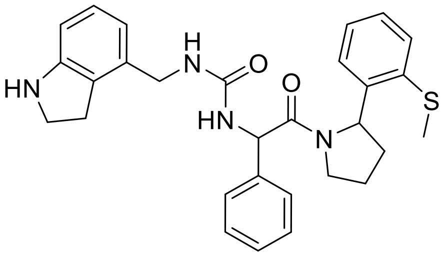

Step 1: tert-butyl (2-(2-(2-(methylthio)phenyl)pyrrolidin-1-yl)-2-oxo-1-phenylethyl)carbamate

The compound is obtained by the procedure described in Example 1, Step 4, starting from 200 mg (0.79 mmol) of 2-((tert-butoxycarbonyl)amino)-2-phenylacetic acid instead of 2-((tert-butoxycarbonyl)amino)-2-(2,5-dimethoxyphenyl)acetic acid. 264 mg of the title compound are obtained as a yellow gel.

Yield: 78%

MH+ : 427.5 (M+1)

Step 2: 2-amino-1-(2-(2-(methylthio)phenyl)pyrrolidin-1-yl)-2-phenylethan-1-one

The compound is obtained by the procedure described in Example 1, Step 5, starting from 264 mg (0.62 mmol) of tert-butyl (2-(2-(2-(methylthio)phenyl)pyrrolidin-1-yl)-2-oxo-1-phenylethyl)carbamate (described in the previous step). 250 mg of the title compound are obtained as a pale yellow gel.

Yield: quant.

MH+ : 327.6 (M+1)

Step 3: tert-butyl 4-((3-(2-(2-(2-(methylthio)phenyl)pyrrolidin-1-yl)-2-oxo-1-phenylethyl)ureido)methyl)indoline-1-carboxylate

The compound is obtained by the procedure described in Example 1, Step 6, starting from 51 mg (0.12 mmol) of 2-amino-1-(2-(2-(methylthio)phenyl)pyrrolidin-1-yl)-2-phenylethan-1-one (described in the previous step) and tert-butyl 4-(aminomethyl)indoline-1-carboxylate instead of tert-butyl (4-(aminomethyl)phenyl)carbamate. 67 mg of the title compound are obtained as a yellow solid.

Yield: 96%

MH+ : 601.8 (M+1)

Step 4: 1-(indolin-4-ylmethyl)-3-(2-(2-(2-(methylthio)phenyl)pyrrolidin-1-yl)-2-oxo-1-phenylethyl)urea

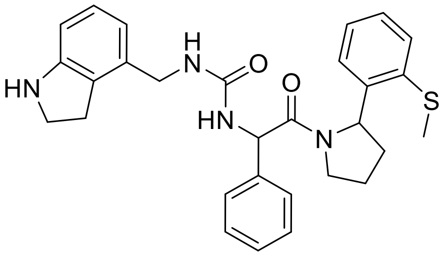

The compound is obtained by the procedure described in Example 1, Step 7, starting from 67 mg (0.11 mmol) of tert-butyl 4-((3-(2-(2-(2-(methylthio)phenyl)pyrrolidin-1-yl)-2-oxo-1-phenylethyl)ureido)methyl)indoline-1-carboxylate (described in the previous step). 40 mg of the title compound are obtained as a slightly yellow solid.

Yield: 71%

MH+ : 501.7 (M+1)

<u>Example 11: Synthesis of 1-((2,3-dihydrobenzo[b][1,4]dioxin-5-yl)methyl)-3-(2-(2-(2-(methylthio)phenyl)pyrrolidin-1-yl)-2-oxo-1-phenylethyl)urea: C129</u>

Step 1: 1-((2,3-dihydrobenzo[b][1,4]dioxin-5-yl)methyl)-3-(2-(2-(2-(methylthio)phenyl)pyrrolidin-1-yl)-2-oxo-1-phenylethyl)urea

The compound is obtained by the procedure described in Example 1, Step 6, starting from 200 mg (0.61 mmol) of 2-amino-1-(2-(2-(methylthio)phenyl)pyrrolidin-1-yl)-2-phenylethan-1-one (described in Example 11, Step 2) and (2,3-dihydrobenzo[b][1,4]dioxin-5-yl)methanamine instead of tert-butyl (4-(aminomethyl)phenyl)carbamate. 41 mg of the title compound are obtained as a white powder.

Yield: 13%

MH+ : 518.2 (M+1)

Example 12: Synthesis of 1-((7-fluoro-2,3-dihydrobenzo[b][1,4]dioxin-5-yl)methyl)-3-(2-(2-(2-(methylthio)phenyl)pyrrolidin-1-yl)-2-oxo-1-phenylethyl)urea: C131

Step 1: 1-((7-fluoro-2,3-dihydrobenzo[b][1,4]dioxin-5-yl)methyl)-3-(2-(2-(2-(methylthio)phenyl)pyrrolidin-1-yl)-2-oxo-1-phenylethyl)urea

The compound is obtained by the procedure described in Example 1, Step 6, starting from 200 mg (0.61 mmol) of 2-amino-1-(2-(2-(methylthio)phenyl)pyrrolidin-1-yl)-2-phenylethan-1-one (described in Example 11, Step 2) and (7-fluoro-2,3-dihydrobenzo[b][1,4]dioxin-5-yl)methanamine instead of tert-butyl (4-(aminomethyl)phenyl)carbamate. 32 mg of the title compound are obtained as a white powder.

Yield: 10%

MH+ : 536.2 (M+1)

Example 13: Synthesis of 1-(benzo[b]thiophen-7-ylmethyl)-3-(2-(2-(2-(methylthio)phenyl)pyrrolidin-1-yl)-2-oxo-1-phenylethyl)urea: C142

Step 1: 1-(benzo[b]thiophen-7-ylmethyl)-3-(2-(2-(2-(methylthio)phenyl)pyrrolidin-1-yl)-2-oxo-1-phenylethyl)urea

The compound is obtained by the procedure described in Example 1, Step 6, starting from 200 mg (0.61 mmol) of 2-amino-1-(2-(2-(methylthio)phenyl)pyrrolidin-1-yl)-2-phenylethan-1-one (described in Example 11, Step 2) and benzo[b]thiophen-7-ylmethanamine instead of tert-butyl (4-(aminomethyl)phenyl)carbamate. 127 mg of the title compound are obtained as a white powder.

Yield: 40%

MH+ : 516.7 (M+1)

Example 14: Synthesis of 1-((2,2-difluorobenzo[d][1,3]dioxol-4-yl)methyl)-3-(2-(2-(2-(methylthio)phenyl)pyrrolidin-1-yl)-2-oxo-1-phenylethyl)urea: C143

Step 1: 1-((2,2-difluorobenzo[d][1,3]dioxol-4-yl)methyl)-3-(2-(2-(2-(methylthio)phenyl)pyrrolidin-1-yl)-2-oxo-1-phenylethyl)urea

The compound is obtained by the procedure described in Example 1, Step 6, starting from 200 mg (0.61 mmol) of 2-amino-1-(2-(2-(methylthio)phenyl)pyrrolidin-1-yl)-2-phenylethan-1-one (described in Example 11, Step 2) and (2,2-difluorobenzo[d][1,3]dioxol-4-yl)methanamine instead of tert-butyl (4-(aminomethyl)phenyl)carbamate. 36 mg of the title compound are obtained as a white powder.

Yield: 11%

MH+ : 540.5 (M+1)

Example 15: Synthesis of 1-(2-(2-(2-(methylthio)phenyl)pyrrolidin-1-yl)-2-oxo-1-phenylethyl)-3-((1,2,3,4-tetrahydroquinolin-6-yl)methyl)urea: C104

Step 1: tert-butyl 6-((3-(2-(2-(2-(methylthio)phenyl)pyrrolidin-1-yl)-2-oxo-1-phenylethyl)ureido)methyl)-3,4-dihydroquinoline-1(2H)-carboxylate

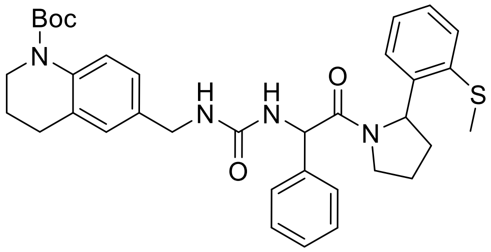

The compound is obtained by the procedure described in Example 1, Step 6, starting from 112 mg (0.34 mmol) of 2-amino-1-(2-(2-(methylthio)phenyl)pyrrolidin-1-yl)-2-phenylethan-1-one (described in Example 11, Step 2) and tert-butyl 6-(aminomethyl)-3,4-dihydroquinoline-1(2H)-carboxylate instead of tert-butyl (4-(aminomethyl)phenyl)carbamate. 165 mg of the title compound are obtained as a yellow oil.

Yield: 78%

MH+ : 615.5 (M+1)

Step 2: 1-(2-(2-(2-(methylthio)phenyl)pyrrolidin-1-yl)-2-oxo-1-phenylethyl)-3-((1,2,3,4-tetrahydroquinolin-6-yl)methyl)urea

The compound is obtained by the procedure described in Example 1, Step 7, starting from 165 mg (0.27 mmol) of tert-butyl 6-((3-(2-(2-(2-(methylthio)phenyl)pyrrolidin-1-yl)-2-oxo-1-phenylethyl)ureido)methyl)-3,4-dihydroquinoline-1(2H)-carboxylate (described in the previous step). 107 mg of the title compound are obtained as a slightly white powder.

Yield: 78%

MH+ : 515.6 (M+1)

<u>Example 16: Synthesis of 1-((1H-indol-4-yl)methyl)-3-(2-(2-(2-(methylthio)phenyl)pyrrolidin-1-yl)-2-oxo-1-phenylethyl)urea: C172</u>

Step 1: 1-((1H-indol-4-yl)methyl)-3-(2-(2-(2-(methylthio)phenyl)pyrrolidin-1-yl)-2-oxo-1-phenylethyl)urea

The compound is obtained by the procedure described in Example 1, Step 6, starting from 111 mg (0.34 mmol) of 2-amino-1-(2-(2-(methylthio)phenyl)pyrrolidin-1-yl)-2-phenylethan-1-one (described in Example 11, Step 2) and (1H-indol-4-yl)methanamine instead of tert-butyl (4-(aminomethyl)phenyl)carbamate. 11 mg of the title compound are obtained as a white powder.

Yield: 7%

MH+ : 499.5 (M+1)

<u>Example 17: Synthesis of 1-(5-aminoisoindolin-2-yl)-3-(2-(2-(2-(methylthio)phenyl)pyrrolidin-1-yl)-2-oxo-1-phenylethyl)urea: C185</u>

Step 1: tert-butyl (2-(3-(2-(2-(2-(methylthio)phenyl)pyrrolidin-1-yl)-2-oxo-1-phenylethyl)ureido)isoindolin-5-yl)carbamate

The compound is obtained by the procedure described in Example 1, Step 6, starting from 70 mg (0.21 mmol) of 2-amino-1-(2-(2-(methylthio)phenyl)pyrrolidin-1-yl)-2-phenylethan-1-one (described in Example 11, Step 2) and tert-butyl isoindolin-5-ylcarbamate instead of tert-butyl (4-(aminomethyl)phenyl)carbamate. 71 mg of the title compound are obtained as a slightly pink solid.

Yield: 57%

MH+ : 587.5 (M+1)

Step 2: 1-(5-aminoisoindolin-2-yl)-3-(2-(2-(2-(methylthio)phenyl)pyrrolidin-1-yl)-2-oxo-1-phenylethyl)urea

The compound is obtained by the procedure described in Example 1, Step 7, starting from 71 mg (0.12 mmol) of tert-butyl (2-(3-(2-(2-(2-(methylthio)phenyl)pyrrolidin-1-yl)-2-oxo-1-phenylethyl)ureido)isoindolin-5-yl)carbamate (described in the previous step). 2 mg of the title compound are obtained as a white solid.

Yield: 3%

MH+ : 487.5 (M+1)

<u>Example 18: Synthesis of 1-((1H-indazol-4-yl)methyl)-3-(2-(2-(2-(methylthio)phenyl)pyrrolidin-1-yl)-2-oxo-1-phenylethyl)urea: C178</u>

Step 1: 1-((1H-indazol-4-yl)methyl)-3-(2-(2-(2-(methylthio)phenyl)pyrrolidin-1-yl)-2-oxo-1-phenylethyl)urea

The compound is obtained by the procedure described in Example 1, Step 6, starting from 110 mg (0.34 mmol) of 2-amino-1-(2-(2-(methylthio)phenyl)pyrrolidin-1-yl)-2-phenylethan-1-one (described in Example 11, Step 2) and (1H-indazol-4-yl)methanamine instead of tert-butyl (4-(aminomethyl)phenyl)carbamate. 6 mg of the title compound are obtained as a white powder.

Yield: 4%

MH+ : 500.6 (M+1)

Example 19: Synthesis of 1-((8-hydroxyquinolin-5-yl)methyl)-3-(2-(2-(2-(methylthio)phenyl)pyrrolidin-1-yl)-2-oxo-1-phenylethyl)urea: C99

Step 1: 1-((8-hydroxyquinolin-5-yl)methyl)-3-(2-(2-(2-(methylthio)phenyl)pyrrolidin-1-yl)-2-oxo-1-phenylethyl)urea

The compound is obtained by the procedure described in Example 1, Step 6, starting from 265 mg (0.81 mmol) of 2-amino-1-(2-(2-(methylthio)phenyl)pyrrolidin-1-yl)-2-phenylethan-1-one (described in Example 11, Step 2) and 5-(aminomethyl)quinolin-8-ol instead of tert-butyl (4-(aminomethyl)phenyl)carbamate. 14 mg of the title compound are obtained as a white powder.

Yield: 3%

MH+ : 527.6 (M+1)

<u>Example 20: Synthesis of 1-(1-(4-amino-3-fluorophenyl)ethyl)-3-(2-(2-(2-(methylthio)phenyl)pyrrolidin-1-yl)-2-oxo-1-phenylethyl)urea: C101</u>

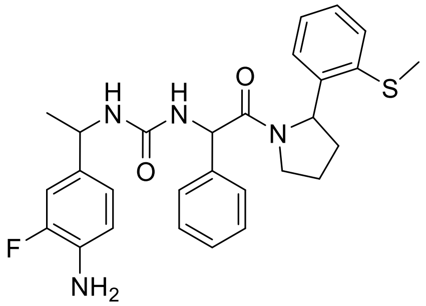

Step 1: 1-(1-(4-amino-3-fluorophenyl)ethyl)-3-(2-(2-(2-(methylthio)phenyl)pyrrolidin-1-yl)-2-oxo-1-phenylethyl)urea

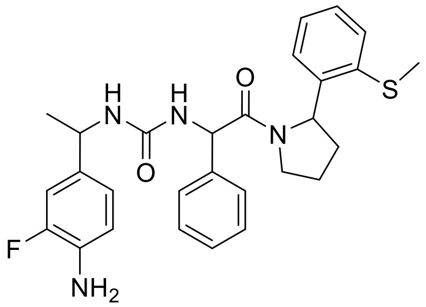

The compound is obtained by the procedure described in Example 1, Step 6, starting from 200 mg (0.61 mmol) of 2-amino-1-(2-(2-(methylthio)phenyl)pyrrolidin-1-yl)-2-phenylethan-1-one (described in Example 11, Step 2) and 4-(1-aminoethyl)-2-fluoroaniline instead of tert-butyl (4-(aminomethyl)phenyl)carbamate. 28 mg of the title compound are obtained as a white powder.

Yield: 9%

MH+ : 507.7 (M+1)

<u>Example 21: Synthesis of 1-((2-azaspiro[3.3]heptan-6-yl)methyl)-3-(2-(2-(2-(methylthio)phenyl)pyrrolidin-1-yl)-2-oxo-1-phenylethyl)urea: C119</u>

Step 1: tert-butyl (4-((3-(2-(2-(2-(methylthio)phenyl)pyrrolidin-1-yl)-2-oxo-1-phenylethyl)ureido)methyl)2-azaspiro[3.3]heptan-6yl)carbamate

The compound is obtained by the procedure described in Example 1, Step 6, starting from 200 mg (0.61 mmol) of 2-amino-1-(2-(2-(methylthio)phenyl)pyrrolidin-1-yl)-2-phenylethan-1-one (described in Example 11, Step 2). 119 mg of the title compound are obtained as a colorless gel.

Yield: 34%

MH+ : 579.8 (M+1)

Step 2: 1-((2-azaspiro[3.3]heptan-6-yl)methyl)-3-(2-(2-(2-(methylthio)phenyl)pyrrolidin-1-yl)-2-oxo-1-phenylethyl)urea

The compound is obtained by the procedure described in Example 1, Step 6, starting from 119 mg (0.206 mmol) of 2-amino-1-(2-(2-(methylthio)phenyl)pyrrolidin-1-yl)-2-phenylethan-1-one (described in Example 11, Step 2) and (2-azaspiro[3.3]heptan-6-yl)methanamine instead of tert-butyl (4-(aminomethyl)phenyl)carbamate. 76 mg of the title compound are obtained as a white powder.

Yield: 78%

MH+ : 479.7 (M+1)

Example 22: Synthesis of 1-(((1r,4r)-4-aminocyclohexyl)methyl)-3-(2-(2-(2-(methylthio)phenyl)pyrrolidin-1-yl)-2-oxo-1-phenylethyl)urea: C120

Step 1: tert-butyl (4-((3-(2-(2-(2-(methylthio)phenyl)pyrrolidin-1-yl)-2-oxo-1-phenylethyl)ureido)methyl)-trans-cyclohexyl)carbamate

The compound is obtained by the procedure described in Example 1, Step 6, starting from 200 mg (0.61 mmol) of 2-amino-1-(2-(2-(methylthio)phenyl)pyrrolidin-1-yl)-2-phenylethan-1-one (described in Example 11, Step 2). 320 mg of the title compound are obtained as a colorless gel.

Yield: 82%

MH+ : 597.8 (M+1)

Step 2: 1-(((1r,4r)-4-aminocyclohexyl)methyl)-3-(2-(2-(2-(methylthio)phenyl)pyrrolidin-1-yl)-2-oxo-1-phenylethyl)urea

The compound is obtained by the procedure described in Example 1, Step 6, starting from 300 mg (0.50 mmol) of 2-amino-1-(2-(2-(methylthio)phenyl)pyrrolidin-1-yl)-2-phenylethan-1-one (described in Example 11, Step 2) and (1r,4r)-4-(aminomethyl)cyclohexan-1-amine instead of tert-butyl (4-(aminomethyl)phenyl)carbamate. 86 mg of the title compound are obtained as a white powder.

Yield: 35%

MH+ : 497.7 (M+1)

Example 23: Synthesis of N-ethyl-5-((3-(2-(2-(2-(methylthio)phenyl)pyrrolidin-1-yl)-2-oxo-1-phenylethyl)ureido)methyl)-3,4-dihydroquinoline-1(2H)-carboxamide:F903 C177

Step 1: tert-butyl 5-((3-(2-(2-(2-(methylthio)phenyl)pyrrolidin-1-yl)-2-oxo-1-phenylethyl)ureido)methyl)-3,4-dihydroquinoline-1(2H)-carboxylate

The compound is obtained by the procedure described in Example 1, Step 6, starting from 62 mg (0.19 mmol) of 2-amino-1-(2-(2-(methylthio)phenyl)pyrrolidin-1-yl)-2-phenylethan-1-one (described in Example 11, Step 2) and tert-butyl 5-(aminomethyl)-3,4-dihydroquinoline-1(2H)-carboxylate instead of tert-butyl (4-(aminomethyl)phenyl)carbamate. 66 mg of the title compound are obtained as a white solid.

Yield: 56%

MH+ : 615.0 (M+1)

Step 2: 1-(2-(2-(2-(methylthio)phenyl)pyrrolidin-1-yl)-2-oxo-1-phenylethyl)-3-((1,2,3,4-tetrahydroquinolin-5-yl)methyl)urea

The compound is obtained by the procedure described in Example 1, Step 7, starting from 66 mg (0.11 mmol) of tert-butyl 5-((3-(2-(2-(2-(methylthio)phenyl)pyrrolidin-1-yl)-2-oxo-1-phenylethyl)ureido)methyl)-3,4-dihydroquinoline-1(2H)-carboxylate (described in the previous step). 18 mg of the title compound are obtained as a white solid.

Yield: 33%

MH+ : 515.4 (M+1)

Step 3: N-ethyl-5-((3-(2-(2-(2-(methylthio)phenyl)pyrrolidin-1-yl)-2-oxo-1-phenylethyl)ureido)methyl)-3,4-dihydroquinoline-1(2H)-carboxamide

15 mg (0.03 mmol, 1 eq) of 1-(2-(2-(2-(methylthio)phenyl)pyrrolidin-1-yl)-2-oxo-1-phenylethyl)-3-((1,2,3,4-tetrahydroquinolin-5-yl)methyl)urea (described in previous step), is dissolved in 500 µL of dry DCM under Argon. 3 µL (0.03 mmol, 1 eq) of ethyl isocyanate is added and the solution is stirred overnight at room temperature. Reaction is dried under reduced pressure. 17 mg of the title compound is obtained as an off white powder.

Yield : quant.

MH+ : 586.5 (M+1)

Example 24: Synthesis of 1-((1-acetyl-1,2,3,4-tetrahydroquinolin-5-yl)methyl)-3-(2-(2-(2-(methylthio)phenyl)pyrrolidin-1-yl)-2-oxo-1-phenylethyl)urea: C179

Step 1: 1-((1-acetyl-1,2,3,4-tetrahydroquinolin-5-yl)methyl)-3-(2-(2-(2-(methylthio)phenyl)pyrrolidin-1-yl)-2-oxo-1-phenylethyl)urea

15 mg (0.03 mmol, 1 eq) of 1-(2-(2-(2-(methylthio)phenyl)pyrrolidin-1-yl)-2-oxo-1-phenylethyl)-3-((1,2,3,4-tetrahydroquinolin-5-yl)methyl)urea (described in Example 25, Step 2), is dissolved in 500 µL of dry DCM under Argon. The solution is cooled to 0°C, then 4.7 µL (0.06 mmol, 2 eq) of pyridine and 2.5 µL (0.04 mmol, 1.2 eq) are added. The mixture is stirred 24h at room temperature. Reaction is diluted with a saturated NH4Cl solution and extracted 2 times with EtOAc. Combined organic layers are dried over Na_2SO_4, filtered and evaporated unde reduced pressure. Crude compound is triturated in Hexane, the solid is filtered and dried under vacuum to give 1.2 mg of a white powder.

Yield : 8%

MH+ : 557.5 (M+1)

<u>Example 25: Synthesis of 1-(4-((2-fluorobenzyl)amino)benzyl)-3-(2-(2-(2-(methylthio)phenyl)pyrrolidin-1-yl)-2-oxo-1-phenylethyl)urea: C182</u>

Step 1: 1-(4-((2-fluorobenzyl)amino)benzyl)-3-(2-(2-(2-(methylthio)phenyl)pyrrolidin-1-yl)-2-oxo-1-phenylethyl)urea

100 mg (0.21 mmol, 1 eq) of 1-(4-aminobenzyl)-3-(2-(2-(2-(methylthio)phenyl)pyrrolidin-1-yl)-2-oxo-1-phenylethyl)urea (described in Example 20, Step 2) and 22µL (0.21 mmol, 1 eq) of 2-fluorobenzaldehyde are dissolved in 4 mL of dry MeOH under Argon. 12µL (0.21 mmol, 1 eq) of AcOH are added and the solution is stirred 10min at room temperature. 8 mg (0.21 mmol, 1 eq) of $NaBH_4$ are added and reaction is stirred overnight at room temperature. Reaction is diluted with water and extracted 2 times with DCM. Combined organic layers are washed with brine, dried over Na_2SO_4, filtered and evaporated unde reduced pressure. Crude product is purified by flash chromatography using a silica gel column and an Hexane/EtOAc mixture as eluent. 14 mg of the title compound is obtained as a pale colorless oil.

Yield : 11%

MH+ : 583.5 (M+1)

Example 26: Synthesis of 1-(2-(2-(2-(methylthio)phenyl)pyrrolidin-1-yl)-2-oxo-1-phenylethyl)-3-(4-((piperidin-3-ylmethyl)amino)benzyl)urea: C183

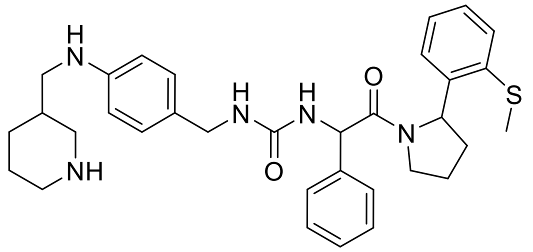

Step 1: tert-butyl 3-(((4-((3-(2-(2-(2-(methylthio)phenyl)pyrrolidin-1-yl)-2-oxo-1-phenylethyl)ureido)methyl)phenyl)amino)methyl)piperidine-1-carboxylate

The compound is obtained by the procedure described in Example 27, Step 1, starting from 35 mg (0.07 mmol) of 1-(4-aminobenzyl)-3-(2-(2-(2-(methylthio)phenyl)pyrrolidin-1-yl)-2-oxo-1-phenylethyl)urea (described in Example 20, Step 2) and tert-butyl 3-formylpiperidine-1-carboxylate instead of 2-fluorobenzaldehyde. 30 mg of the title compound are obtained as a white powder.

Yield: 60%

MH+ : 672.7 (M+1)

Step 2: 1-(2-(2-(2-(methylthio)phenyl)pyrrolidin-1-yl)-2-oxo-1-phenylethyl)-3-(4-((piperidin-3-ylmethyl)amino)benzyl)urea

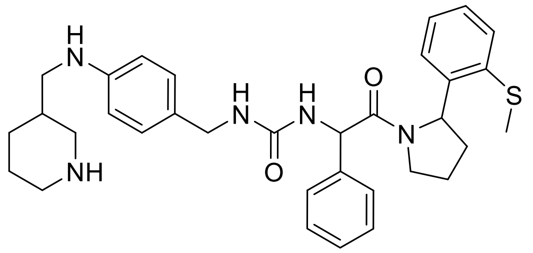

The compound is obtained by the procedure described in Example 1, Step 7, starting from 30 mg (0.04 mmol) of tert-butyl 3-(((4-((3-(2-(2-(2-(methylthio)phenyl)pyrrolidin-1-yl)-2-oxo-1-phenylethyl)ureido)methyl)phenyl)amino)methyl)piperidine-1-carboxylate (described in the previous step). 8 mg of the title compound are obtained as a pale yellow powder.

Yield: 31%

MH+ : 572.7 (M+1)

Example 27: Synthesis of 1-((5-aminopyridin-2-yl)methyl)-3-(1-(5-bromo-2-methoxyphenyl)-2-(2-(2-(methylthio)phenyl)pyrrolidin-1-yl)-2-oxoethyl)urea: C115

Step 1: tert-butyl (1-(5-bromo-2-methoxyphenyl)-2-(2-(2-(methylthio)phenyl)pyrrolidin-1-yl)-2-oxoethyl)carbamate

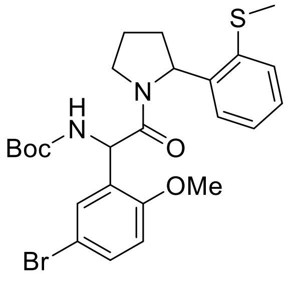

The compound is obtained by the procedure described in Example 1, Step 4, starting from 400 mg (1.12 mmol) of 2-(5-bromo-2-methoxyphenyl)-2-((tert-butoxycarbonyl)amino)acetic acid instead of 2-((tert-butoxycarbonyl)amino)-2-(2,5-dimethoxyphenyl)acetic acid. 478 mg of the title compound are obtained as a colorless oil.

Yield: 80%

MH+ : 534.6-536.6 (M+1)

Step 2: 2-amino-2-(5-bromo-2-methoxyphenyl)-1-(2-(2-(methylthio)phenyl)pyrrolidin-1-yl)ethan-1-one

The compound is obtained by the procedure described in Example 1, Step 5, starting from 200 mg (0.37 mmol) of tert-butyl (1-(5-bromo-2-methoxyphenyl)-2-(2-(2-(methylthio)phenyl)pyrrolidin-1-yl)-2-oxoethyl)carbamate (described in the previous step). 117 mg of the title compound are obtained as a slightly green oil.

Yield: 72%

MH+ : 434.5-436.5 (M+1)

Step 3: tert-butyl (6-((3-(1-(5-bromo-2-methoxyphenyl)-2-(2-(2-(methylthio)phenyl)pyrrolidin-1-yl)-2-oxoethyl)ureido)methyl)pyridin-3-yl)carbamate

The compound is obtained by the procedure described in Example 1, Step 6, starting from 81 mg (0.19 mmol) of 2-amino-2-(5-bromo-2-methoxyphenyl)-1-(2-(2-(methylthio)phenyl)pyrrolidin-1-yl)ethan-1-one (described in the previous step) and tert-butyl (6-(aminomethyl)pyridin-3-yl)carbamate instead of tert-butyl (4-(aminomethyl)phenyl)carbamate. 64 mg of the title compound are obtained as a colorless oil.

Yield: 56%

MH+ : 684.6-686.5 (M+1)

Step 4: 1-((5-aminopyridin-2-yl)methyl)-3-(1-(5-bromo-2-methoxyphenyl)-2-(2-(2-(methylthio)phenyl)pyrrolidin-1-yl)-2-oxoethyl)urea

The compound is obtained by the procedure described in Example 1, Step 7, starting from 64 mg (0.093 mmol) of tert-butyl (6-((3-(1-(5-bromo-2-methoxyphenyl)-2-(2-(2-(methylthio)phenyl)pyrrolidin-1-yl)-2-oxoethyl)ureido)methyl)pyridin-3-yl)carbamate (described in the previous step).157 mg of the title compound are obtained as a white solid.

Yield: 27%

MH+ : 584.6-586.5 (M+1)

<u>Example 28: Synthesis of 1-(1-(4-amino-3-fluorophenyl)ethyl)-3-(1-(5-bromo-2-methoxyphenyl)-2-(2-(2-(methylthio)phenyl)pyrrolidin-1-yl)-2-oxoethyl)urea: C123</u>

Step 1: 1-(1-(4-amino-3-fluorophenyl)ethyl)-3-(1-(5-bromo-2-methoxyphenyl)-2-(2-(2-(methylthio)phenyl)pyrrolidin-1-yl)-2-oxoethyl)urea

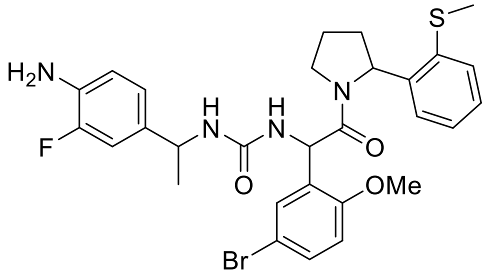

The compound is obtained by the procedure described in Example 1, Step 6, starting from 166 mg (0.38 mmol) of 2-amino-2-(5-bromo-2-methoxyphenyl)-1-(2-(2-(methylthio)phenyl)pyrrolidin-1-yl)ethan-1-one (described in Example 29, Step 2) and 4-(1-aminoethyl)-2-fluoroaniline instead of tert-butyl (4-(aminomethyl)phenyl)carbamate. 50 mg of the title compound are obtained as a white powder.

Yield: 22%

MH+ : 615.6-617.5 (M+1)

<u>Example 29: Synthesis of 1-(4-aminobenzyl)-3-(2-(2-(benzo[b]thiophen-7-yl)pyrrolidin-1-yl)-1-(5-bromo-2-methoxyphenyl)-2-oxoethyl)urea: C110</u>

Step 1: tert-butyl (2-(2-(benzo[b]thiophen-7-yl)pyrrolidin-1-yl)-1-(5-bromo-2-methoxyphenyl)-2-oxoethyl)carbamate

The compound is obtained by the procedure described in Example 1, Step 4, starting from 200 mg (0.56 mmol) of 2-(5-bromo-2-methoxyphenyl)-2-((tert-butoxycarbonyl)amino)acetic acid instead of 2-((tert-butoxycarbonyl)amino)-2-(2,5-dimethoxyphenyl)acetic acid and 2-(benzo[b]thiophen-7-yl)pyrrolidine instead of 2-(2-(methylthio)phenyl)pyrrolidine. 301 mg of the title compound are obtained as a colorless oil.

Yield: 99%

MH+ : 545.5-547.5 (M+1)

Step 2: 2-amino-1-(2-(benzo[b]thiophen-7-yl)pyrrolidin-1-yl)-2-(5-bromo-2-methoxyphenyl)ethan-1-one

The compound is obtained by the procedure described in Example 1, Step 5, starting from 301 mg (0.55 mmol) of tert-butyl (2-(2-(benzo[b]thiophen-7-yl)pyrrolidin-1-yl)-1-(5-bromo-2-methoxyphenyl)-2-oxoethyl)carbamate (described in the previous step). 176 mg of the title compound are obtained as a slightly green oil.

Yield: 72%

MH+ : 445.5-447.4 (M+1)

Step 3: tert-butyl (4-((3-(2-(2-(benzo[b]thiophen-7-yl)pyrrolidin-1-yl)-1-(5-bromo-2-methoxyphenyl)-2-oxoethyl)ureido)methyl)phenyl)carbamate

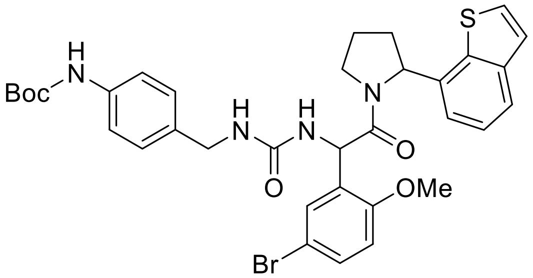

The compound is obtained by the procedure described in Example 1, Step 6, starting from 87 mg (0.19 mmol) of 2-amino-1-(2-(benzo[b]thiophen-7-yl)pyrrolidin-1-yl)-2-(5-bromo-2-methoxyphenyl)ethan-1-one (described in the previous step). 100 mg of the title compound are obtained as a colorless oil.

Yield: 74%

MH+ : 693.5-695.5 (M+1)

Step 4: 1-(4-aminobenzyl)-3-(2-(2-(benzo[b]thiophen-7-yl)pyrrolidin-1-yl)-1-(5-bromo-2-methoxyphenyl)-2-oxoethyl)urea

The compound is obtained by the procedure described in Example 1, Step 7, starting from 100 mg (0.14 mmol) of tert-butyl (4-((3-(2-(2-(benzo[b]thiophen-7-yl)pyrrolidin-1-yl)-1-(5-bromo-2-methoxyphenyl)-2-oxoethyl)ureido)methyl)phenyl)carbamate (described in the previous step). 57 mg of the title compound are obtained as a white solid.

Yield: 67%

MH+ : 593.5-595.5 (M+1)

<u>Example 30: Synthesis of 1-(4-aminobenzyl)-3-((R)-2-((S)-2-(benzo[b]thiophen-7-yl)pyrrolidin-1-yl)-1-(5-bromo-2-methoxyphenyl)-2-oxoethyl)urea: C110RS</u>

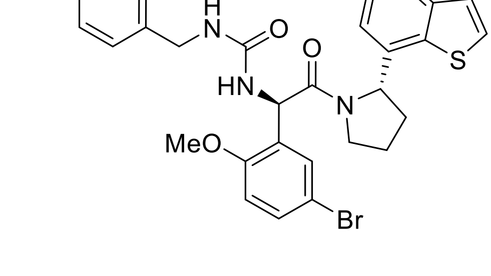

Step 1: (R)-2-(5-bromo-2-methoxyphenyl)-2-((tert-butoxycarbonyl)amino)acetic acid

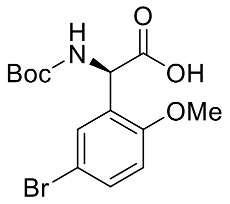

290 mg (0.98 mmol, 1 eq) of (R)-2-amino-2-(5-bromo-2-methoxyphenyl)acetic acid HCl are dissolved in 10 mL of a THF/H_2O mixture (1/1, v/v). The solution is cooled to 0°C and 125 mg (3.13 mmol, 3.2 eq) of NaOH are added. The mixture is stirred 10 min at this temperature, then 213 mg (0.98 mmol, 1 eq) of Boc_2O are added and reaction is stirred 40min at 0°C. Reaction is diluted with H_2O and extracted 3 times with DCM. Aqueous layer is cooled to 0°C and acidified with HCl 37% until pH reached a value of 2. Aqueous layer is extracted 3 times with DCM and this organic layer is dried over Na_2SO_4, filtered and evaporated unde reduced pressure to give 411 mg of a colorless gel.

Yield : quant.

MH+ : 360.4-362.4 (M+1)

Step 2: tert-butyl (R)-(2-(2-(benzo[b]thiophen-7-yl)pyrrolidin-1-yl)-1-(5-bromo-2-methoxyphenyl)-2-oxoethyl)carbamate

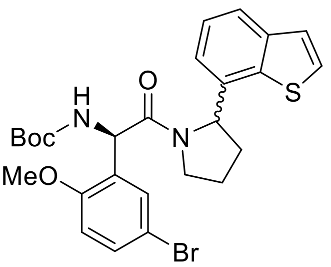

The compound is obtained by the procedure described in Example 1, Step 4, starting from 352 mg (0.98 mmol) of (R)-2-(5-bromo-2-methoxyphenyl)-2-((tert-butoxycarbonyl)amino)acetic acid (described in the previous step) and 2-(benzo[b]thiophen-7-yl)pyrrolidine instead of 2-(2-(methylthio)phenyl)pyrrolidine. 507 mg of the title compound are obtained as a yellow gel.

Yield: 95%

MH+ : 545.5-547.5 (M+1)

Step 3: (R)-2-amino-1-(2-(benzo[b]thiophen-7-yl)pyrrolidin-1-yl)-2-(5-bromo-2-methoxyphenyl)ethan-1-one

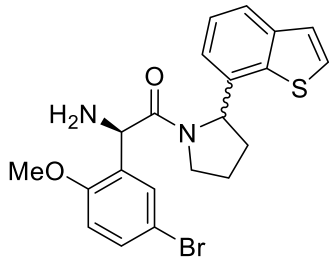

The compound is obtained by the procedure described in Example 1, Step 5, starting from 507 mg (0.93 mmol) of tert-butyl tert-butyl (R)-(2-(2-(benzo[b]thiophen-7-yl)pyrrolidin-1-yl)-1-(5-bromo-2-methoxyphenyl)-2-oxoethyl)carbamate (described in the previous step). 280 mg of the title compound are obtained as a yellow gel.

Yield: 68%

MH+ : 445.5-447.4 (M+1)

Step 4: tert-butyl (R)-(4-((3-(2-(2-(benzo[b]thiophen-7-yl)pyrrolidin-1-yl)-1-(5-bromo-2-methoxyphenyl)-2-oxoethyl)ureido)methyl)phenyl)carbamate

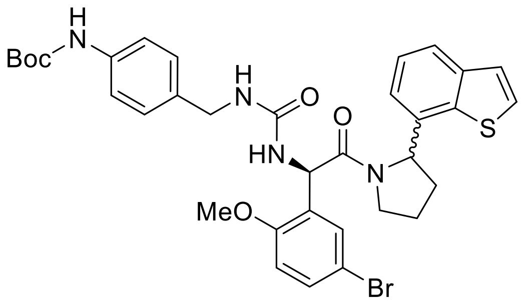

The compound is obtained by the procedure described in Example 1, Step 6, starting from 150 mg (0.34 mmol) of (R)-2-amino-1-(2-(benzo[b]thiophen-7-yl)pyrrolidin-1-yl)-2-(5-bromo-2-methoxyphenyl)ethan-1-one (described in the previous step). 201 mg of the title compound are obtained as a white solid.

Yield: 86%

MH+ : 693.5-695.5 (M+1)

Step 5: (R)-1-(4-aminobenzyl)-3-(2-(2-(benzo[b]thiophen-7-yl)pyrrolidin-1-yl)-1-(5-bromo-2-methoxyphenyl)-2-oxoethyl)urea

The compound is obtained by the procedure described in Example 1, Step 7, starting from 201 mg (0.29 mmol) of tert-butyl (R)-(4-((3-(2-(2-(benzo[b]thiophen-7-yl)pyrrolidin-1-yl)-1-(5-bromo-2-methoxyphenyl)-2-oxoethyl)ureido)methyl)phenyl)carbamate (described in the previous step). 125 mg of the title compound are obtained as a white solid.

Yield: 73%

MH+ : 593.5-595.5 (M+1)

Step 6: 1-(4-aminobenzyl)-3-((R)-2-((S)-2-(benzo[b]thiophen-7-yl)pyrrolidin-1-yl)-1-(5-bromo-2-methoxyphenyl)-2-oxoethyl)urea

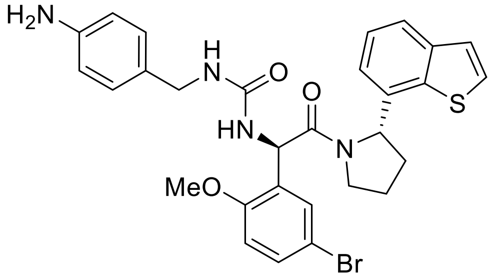

(R)-1-(4-aminobenzyl)-3-(2-(2-(benzo[b]thiophen-7-yl)pyrrolidin-1-yl)-1-(5-bromo-2-methoxyphenyl)-2-oxoethyl)urea (described in previous step) is separated by flash chromatography using a Chiralflash IG column and an EtOH/DCM mixture as the mobile phase. First fraction to be eluted is 1-(4-aminobenzyl)-3-((R)-2-((S)-2-(benzo[b]thiophen-7-yl)pyrrolidin-1-yl)-1-(5-bromo-2-methoxyphenyl)-2-oxoethyl)urea with ee > 98%. Product is purified by flash chromatography using a C18 column and an H_2O/MeOH mixture as eluent. 33 mg of the title compound is obtained as a white solid.

MH+ : 593.5-595.5 (M+1)

<u>Example 31: Synthesis of 1-(4-aminobenzyl)-3-((R)-2-((R)-2-(benzo[b]thiophen-7-yl)pyrrolidin-1-yl)-1-(5-bromo-2-methoxyphenyl)-2-oxoethyl)urea: C110RR</u>

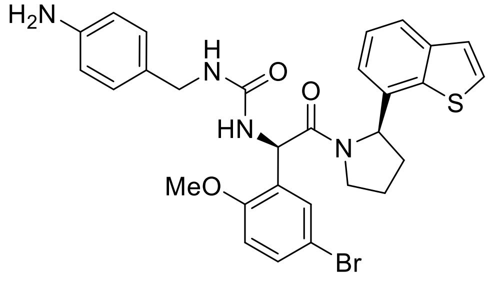

Step 1: 1-(4-aminobenzyl)-3-((R)-2-((R)-2-(benzo[b]thiophen-7-yl)pyrrolidin-1-yl)-1-(5-bromo-2-methoxyphenyl)-2-oxoethyl)urea

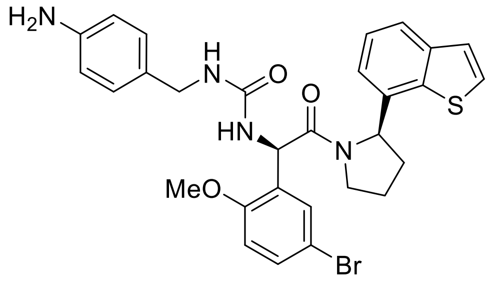

(R)-1-(4-aminobenzyl)-3-(2-(2-(benzo[b]thiophen-7-yl)pyrrolidin-1-yl)-1-(5-bromo-2-methoxyphenyl)-2-oxoethyl)urea (described in Example 32, Step 5) is separated by flash chromatography using a Chiralflash IG column and an EtOH/DCM mixture as the mobile phase. Second fraction to be eluted is 1-(4-aminobenzyl)-3-((R)-2-((R)-2-(benzo[b]thiophen-7-yl)pyrrolidin-1-yl)-1-(5-bromo-2-methoxyphenyl)-2-oxoethyl)urea with ee > 96%. Product is purified by flash chromatography using a C18 column and an H_2O/MeOH mixture as eluent. 48 mg of the title compound is obtained as a white solid.

MH+ : 593.5-595.5 (M+1)

Example 32: Synthesis of 1-(4-aminobenzyl)-3-((S)-2-((R)-2-(benzo[b]thiophen-7-yl)pyrrolidin-1-yl)-1-(5-bromo-2-methoxyphenyl)-2-oxoethyl)urea: C110SR

Step 1: (S)-2-(5-bromo-2-methoxyphenyl)-2-((tert-butoxycarbonyl)amino)acetic acid

The compound is obtained by the procedure described in Example 32, Step 1, starting from 265 mg (0.89 mmol) of (S)-2-amino-2-(5-bromo-2-methoxyphenyl)acetic acid HCl instead of (R)-2-amino-2-(5-bromo-2-methoxyphenyl)acetic acid HCl. 363 mg of the title compound are obtained as a colorless gel.

Yield: quant.

MH+ : 360.4-362.4 (M+1)

Step 2: tert-butyl (S)-(2-(2-(benzo[b]thiophen-7-yl)pyrrolidin-1-yl)-1-(5-bromo-2-methoxyphenyl)-2-oxoethyl)carbamate

The compound is obtained by the procedure described in Example 1, Step 4, starting from 322 mg (0.89 mmol) of (S)-2-(5-bromo-2-methoxyphenyl)-2-((tert-butoxycarbonyl)amino)acetic acid (described in the previous step) and 2-(benzo[b]thiophen-7-yl)pyrrolidine instead of 2-(2-(methylthio)phenyl)pyrrolidine. 501 mg of the title compound are obtained as a yellow gel.

Yield: quant.

MH+ : 545.5-547.5 (M+1)

Step 3: (S)-2-amino-1-(2-(benzo[b]thiophen-7-yl)pyrrolidin-1-yl)-2-(5-bromo-2-methoxyphenyl)ethan-1-one

The compound is obtained by the procedure described in Example 1, Step 5, starting from 501 mg (0.92 mmol) of tert-butyl (S)-(2-(2-(benzo[b]thiophen-7-yl)pyrrolidin-1-yl)-1-(5-bromo-2-methoxyphenyl)-2-oxoethyl)carbamate (described in the previous step). 270 mg of the title compound are obtained as a yellow gel.

Yield: 66%

MH+ : 445.5-447.4 (M+1)

Step 4: tert-butyl (S)-(4-((3-(2-(2-(benzo[b]thiophen-7-yl)pyrrolidin-1-yl)-1-(5-bromo-2-methoxyphenyl)-2-oxoethyl)ureido)methyl)phenyl)carbamate

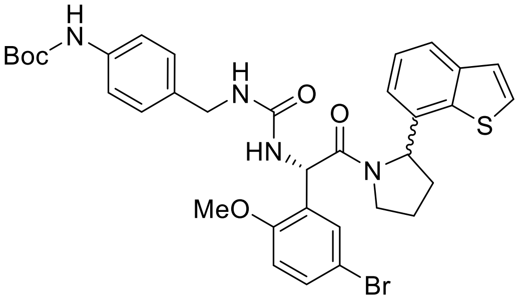

The compound is obtained by the procedure described in Example 1, Step 6, starting from 140 mg (0.31 mmol) of (S)-2-amino-1-(2-(benzo[b]thiophen-7-yl)pyrrolidin-1-yl)-2-(5-bromo-2-methoxyphenyl)ethan-1-one (described in the previous step). 186 mg of the title compound are obtained as a white solid.

Yield: 85%

MH+ : 693.5-695.5 (M+1)

Step 5: (S)-1-(4-aminobenzyl)-3-(2-(2-(benzo[b]thiophen-7-yl)pyrrolidin-1-yl)-1-(5-bromo-2-methoxyphenyl)-2-oxoethyl)urea

The compound is obtained by the procedure described in Example 1, Step 7, starting from 186 mg (0.29 mmol) of tert-butyl (S)-(4-((3-(2-(2-(benzo[b]thiophen-7-yl)pyrrolidin-1-yl)-1-(5-bromo-2-methoxyphenyl)-2-oxoethyl)ureido)methyl)phenyl)carbamate (described in the previous step). 109 mg of the title compound are obtained as a white solid.

Yield: 69%

MH+ : 593.5-595.5 (M+1)

Step 6: 1-(4-aminobenzyl)-3-((S)-2-((R)-2-(benzo[b]thiophen-7-yl)pyrrolidin-1-yl)-1-(5-bromo-2-methoxyphenyl)-2-oxoethyl)urea

(S)-1-(4-aminobenzyl)-3-(2-(2-(benzo[b]thiophen-7-yl)pyrrolidin-1-yl)-1-(5-bromo-2-methoxyphenyl)-2-oxoethyl)urea (described in previous step) is separated by flash chromatography using a Chiralflash IG column and an EtOH/DCM mixture as the mobile phase. First fraction to be eluted is 1-(4-aminobenzyl)-3-((S)-2-((R)-2-(benzo[b]thiophen-7-yl)pyrrolidin-1-yl)-1-(5-bromo-2-methoxyphenyl)-2-oxoethyl)urea with ee > 99%. Product is purified by flash chromatography using a C18 column and an H_2O/MeOH mixture as eluent. 20 mg of the title compound is obtained as a white solid.

MH+ : 593.5-595.5 (M+1)

Example 33: Synthesis of 1-(4-aminobenzyl)-3-((S)-2-((S)-2-(benzo[b]thiophen-7-yl)pyrrolidin-1-yl)-1-(5-bromo-2-methoxyphenyl)-2-oxoethyl)urea: C110SS

Step 1: 1-(4-aminobenzyl)-3-((S)-2-((S)-2-(benzo[b]thiophen-7-yl)pyrrolidin-1-yl)-1-(5-bromo-2-methoxyphenyl)-2-oxoethyl)urea

(S)-1-(4-aminobenzyl)-3-(2-(2-(benzo[b]thiophen-7-yl)pyrrolidin-1-yl)-1-(5-bromo-2-methoxyphenyl)-2-oxoethyl)urea (described in Example 34, Step 5) is separated by flash chromatography using a Chiralflash IG column and an EtOH/DCM mixture as the mobile phase. Second fraction to be eluted is 1-(4-aminobenzyl)-3-((S)-2-((S)-2-(benzo[b]thiophen-7-yl)pyrrolidin-1-yl)-1-(5-bromo-2-methoxyphenyl)-2-oxoethyl)urea with ee > 99%. Product is purified by flash chromatography using a C18 column and an H_2O/MeOH mixture as eluent. 32 mg of the title compound is obtained as a white solid.

MH+ : 593.5-595.5 (M+1)

Example 34: Synthesis of 1-(2-(2-(benzo[b]thiophen-7-yl)pyrrolidin-1-yl)-1-(5-bromo-2-methoxyphenyl)-2-oxoethyl)-3-((1,2,3,4-tetrahydroquinolin-6-yl)methyl)urea: C105

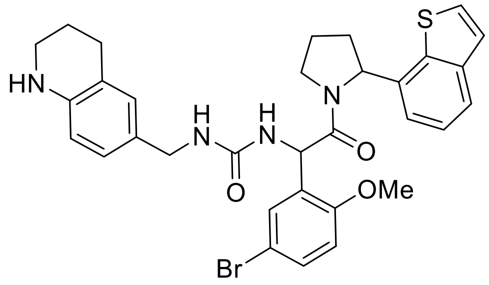

Step 1: tert-butyl 6-((3-(2-(2-(benzo[b]thiophen-7-yl)pyrrolidin-1-yl)-1-(5-bromo-2-methoxyphenyl)-2-oxoethyl)ureido)methyl)-3,4-dihydroquinoline-1(2H)-carboxylate

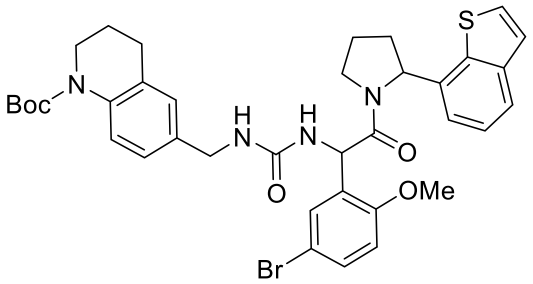

The compound is obtained by the procedure described in Example 1, Step 6, starting from 77 mg (0.17 mmol) of 2-amino-1-(2-(benzo[b]thiophen-7-yl)pyrrolidin-1-yl)-2-(5-bromo-2-methoxyphenyl)ethan-1-one (described in Example 31, Step 2) and tert-butyl 6-(aminomethyl)-3,4-dihydroquinoline-1(2H)-carboxylate instead of tert-butyl (4-(aminomethyl)phenyl)carbamate. 89 mg of the title compound are obtained as a colorless oil.

Yield: 70%

MH+ : 733.5-735.6 (M+1)

Step 2: 1-(2-(2-(benzo[b]thiophen-7-yl)pyrrolidin-1-yl)-1-(5-bromo-2-methoxyphenyl)-2-oxoethyl)-3-((1,2,3,4-tetrahydroquinolin-6-yl)methyl)urea

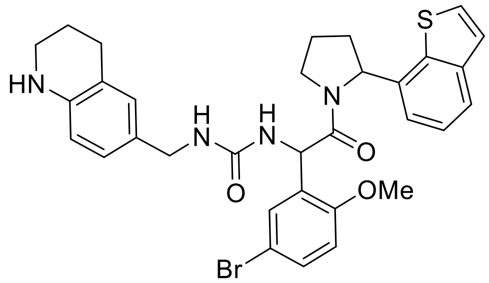

The compound is obtained by the procedure described in Example 1, Step 7, starting from 89 mg (0.12 mmol) of tert-butyl 6-((3-(2-(2-(benzo[b]thiophen-7-yl)pyrrolidin-1-yl)-1-(5-bromo-2-methoxyphenyl)-2-oxoethyl)ureido)methyl)-3,4-dihydroquinoline-1(2H)-carboxylate (described in the previous step). 59 mg of the title compound are obtained as a slightly yellow solid.

Yield: 77%

MH+ : 633.5-635.6 (M+1)

Example 35: Synthesis of 1-((R)-2-((S)-2-(benzo[b]thiophen-7-yl)pyrrolidin-1-yl)-1-(5-bromo-2-methoxyphenyl)-2-oxoethyl)-3-((1,2,3,4-tetrahydroquinolin-6-yl)methyl)urea: C105RS

Step 1: tert-butyl (R)-6-((3-(2-(2-(benzo[b]thiophen-7-yl)pyrrolidin-1-yl)-1-(5-bromo-2-methoxyphenyl)-2-oxoethyl)ureido)methyl)-3,4-dihydroquinoline-1(2H)-carboxylate

The compound is obtained by the procedure described in Example 1, Step 6, starting from 130 mg (0.29 mmol) of (R)-2-amino-1-(2-(benzo[b]thiophen-7-yl)pyrrolidin-1-yl)-2-(5-bromo-2-methoxyphenyl)ethan-1-one (described in Example 32, Step 3). 151 mg of the title compound are obtained as a white solid.

Yield: 71%

MH+ : 733.5-735.6 (M+1)

Step 2: (R)-1-(2-(2-(benzo[b]thiophen-7-yl)pyrrolidin-1-yl)-1-(5-bromo-2-methoxyphenyl)-2-oxoethyl)-3-((1,2,3,4-tetrahydroquinolin-6-yl)methyl)urea

The compound is obtained by the procedure described in Example 1, Step 6, starting from 151 mg (0.21 mmol) of tert-butyl (R)-6-((3-(2-(2-(benzo[b]thiophen-7-yl)pyrrolidin-1-yl)-1-(5-bromo-2-methoxyphenyl)-2-oxoethyl)ureido)methyl)-3,4-dihydroquinoline-1(2H)-carboxylate (described in the previous step). 107 mg of the title compound are obtained as a white solid.

Yield: 82%

MH+ : 633.5-635.6 (M+1)

Step 3: 1-((R)-2-((S)-2-(benzo[b]thiophen-7-yl)pyrrolidin-1-yl)-1-(5-bromo-2-methoxyphenyl)-2-oxoethyl)-3-((1,2,3,4-tetrahydroquinolin-6-yl)methyl)urea

(R)-1-(2-(2-(benzo[b]thiophen-7-yl)pyrrolidin-1-yl)-1-(5-bromo-2-methoxyphenyl)-2-oxoethyl)-3-((1,2,3,4-tetrahydroquinolin-6-yl)methyl)urea (described in previous step) is separated by flash chromatography using a Chiralflash IG column and an EtOH/DCM mixture as the mobile phase. First fraction to be eluted is 1-((R)-2-((S)-2-(benzo[b]thiophen-7-yl)pyrrolidin-1-yl)-1-(5-bromo-2-methoxyphenyl)-2-oxoethyl)-3-((1,2,3,4-tetrahydroquinolin-6-yl)methyl)urea with ee > 99%. Product is purified by flash chromatography using a C18 column and an H_2O/MeOH mixture as eluent. 25 mg of the title compound is obtained as a white solid.

MH+ : 633.5-635.6 (M+1)

<u>Example 36: Synthesis of 1-((R)-2-((R)-2-(benzo[b]thiophen-7-yl)pyrrolidin-1-yl)-1-(5-bromo-2-methoxyphenyl)-2-oxoethyl)-3-((1,2,3,4-tetrahydroquinolin-6-yl)methyl)urea: C105RR</u>

Step 1: 1-((R)-2-((R)-2-(benzo[b]thiophen-7-yl)pyrrolidin-1-yl)-1-(5-bromo-2-methoxyphenyl)-2-oxoethyl)-3-((1,2,3,4-tetrahydroquinolin-6-yl)methyl)urea

(R)-1-(2-(2-(benzo[b]thiophen-7-yl)pyrrolidin-1-yl)-1-(5-bromo-2-methoxyphenyl)-2-oxoethyl)-3-((1,2,3,4-tetrahydroquinolin-6-yl)methyl)urea (described in Example 37, Step 2) is separated by flash chromatography using a Chiralflash IG column and an EtOH/DCM mixture as the mobile phase. Second fraction to be eluted is 1-((R)-2-((R)-2-(benzo[b]thiophen-7-yl)pyrrolidin-1-yl)-1-(5-bromo-2-methoxyphenyl)-2-oxoethyl)-3-((1,2,3,4-tetrahydroquinolin-6-yl)methyl)urea with ee > 96%. Product is purified by flash chromatography using a C18 column and an H_2O/MeOH mixture as eluent. 33 mg of the title compound is obtained as a white solid.

MH+ : 633.5-635.6 (M+1)

Example 37: Synthesis of 1-((S)-2-((R)-2-(benzo[b]thiophen-7-yl)pyrrolidin-1-yl)-1-(5-bromo-2-methoxyphenyl)-2-oxoethyl)-3-((1,2,3,4-tetrahydroquinolin-6-yl)methyl)urea: C105SR

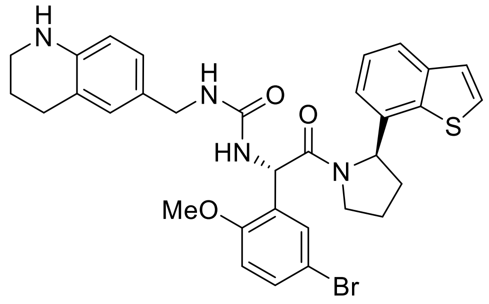

Step 1: tert-butyl (S)-6-((3-(2-(2-(benzo[b]thiophen-7-yl)pyrrolidin-1-yl)-1-(5-bromo-2-methoxyphenyl)-2-oxoethyl)ureido)methyl)-3,4-dihydroquinoline-1(2H)-carboxylate

The compound is obtained by the procedure described in Example 1, Step 6, starting from 124 mg (0.28 mmol) of (S)-2-amino-1-(2-(benzo[b]thiophen-7-yl)pyrrolidin-1-yl)-2-(5-bromo-2-methoxyphenyl)ethan-1-one (described in Example 34, Step 3). 162 mg of the title compound are obtained as a colorless gel.

Yield: 79%

MH+ : 733.5-735.6 (M+1)

Step 2: (S)-1-(2-(2-(benzo[b]thiophen-7-yl)pyrrolidin-1-yl)-1-(5-bromo-2-methoxyphenyl)-2-oxoethyl)-3-((1,2,3,4-tetrahydroquinolin-6-yl)methyl)urea

The compound is obtained by the procedure described in Example 1, Step 6, starting from 162 mg (0.22 mmol) of tert-butyl (S)-6-((3-(2-(2-(benzo[b]thiophen-7-yl)pyrrolidin-1-yl)-1-(5-bromo-2-methoxyphenyl)-2-oxoethyl)ureido)methyl)-3,4-dihydroquinoline-1(2H)-carboxylate (described in the previous step). 106 mg of the title compound are obtained as a white solid.

Yield: 76%

MH+ : 633.5-635.6 (M+1)

Step 3: 1-((S)-2-((R)-2-(benzo[b]thiophen-7-yl)pyrrolidin-1-yl)-1-(5-bromo-2-methoxyphenyl)-2-oxoethyl)-3-((1,2,3,4-tetrahydroquinolin-6-yl)methyl)urea

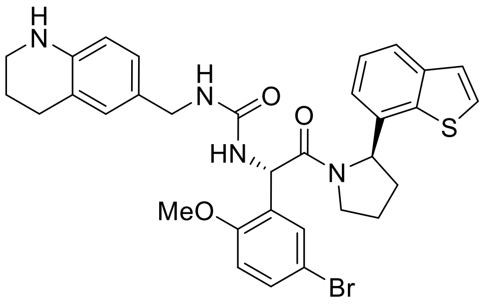

(S)-1-(2-(2-(benzo[b]thiophen-7-yl)pyrrolidin-1-yl)-1-(5-bromo-2-methoxyphenyl)-2-oxoethyl)-3-((1,2,3,4-tetrahydroquinolin-6-yl)methyl)urea (described in previous step) is separated

by flash chromatography using a Chiralflash IG column and an EtOH/DCM mixture as the mobile phase. First fraction to be eluted is 1-((S)-2-((R)-2-(benzo[b]thiophen-7-yl)pyrrolidin-1-yl)-1-(5-bromo-2-methoxyphenyl)-2-oxoethyl)-3-((1,2,3,4-tetrahydroquinolin-6-yl)methyl)urea with ee > 98%. Product is purified by flash chromatography using a C18 column and an H_2O/MeOH mixture as eluent. 15 mg of the title compound is obtained as a pale yellow solid.

MH+ : 633.5-635.6 (M+1)

Example 38: Synthesis of 1-((S)-2-((S)-2-(benzo[b]thiophen-7-yl)pyrrolidin-1-yl)-1-(5-bromo-2-methoxyphenyl)-2-oxoethyl)-3-((1,2,3,4-tetrahydroquinolin-6-yl)methyl)urea: C105SS

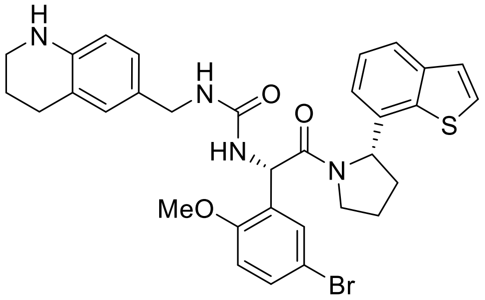

Step 1: 1-((S)-2-((S)-2-(benzo[b]thiophen-7-yl)pyrrolidin-1-yl)-1-(5-bromo-2-methoxyphenyl)-2-oxoethyl)-3-((1,2,3,4-tetrahydroquinolin-6-yl)methyl)urea

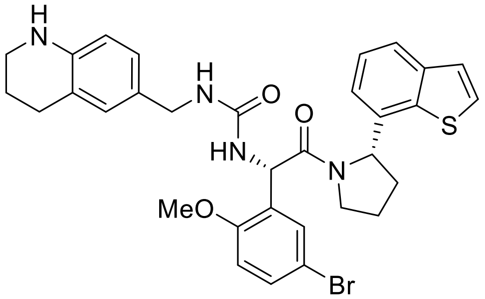

(S)-1-(2-(2-(benzo[b]thiophen-7-yl)pyrrolidin-1-yl)-1-(5-bromo-2-methoxyphenyl)-2-oxoethyl)-3-((1,2,3,4-tetrahydroquinolin-6-yl)methyl)urea (described in Example 39, Step 2) is separated by flash chromatography using a Chiralflash IG column and an EtOH/DCM mixture as the mobile phase. Second fraction to be eluted is 1-((S)-2-((S)-2-(benzo[b]thiophen-7-yl)pyrrolidin-1-yl)-1-(5-bromo-2-methoxyphenyl)-2-oxoethyl)-3-((1,2,3,4-tetrahydroquinolin-6-yl)methyl)urea with ee > 98%. Product is purified by flash chromatography using a C18 column and an H_2O/MeOH mixture as eluent. 29 mg of the title compound is obtained as a white solid.

MH+ : 633.5-635.6 (M+1)

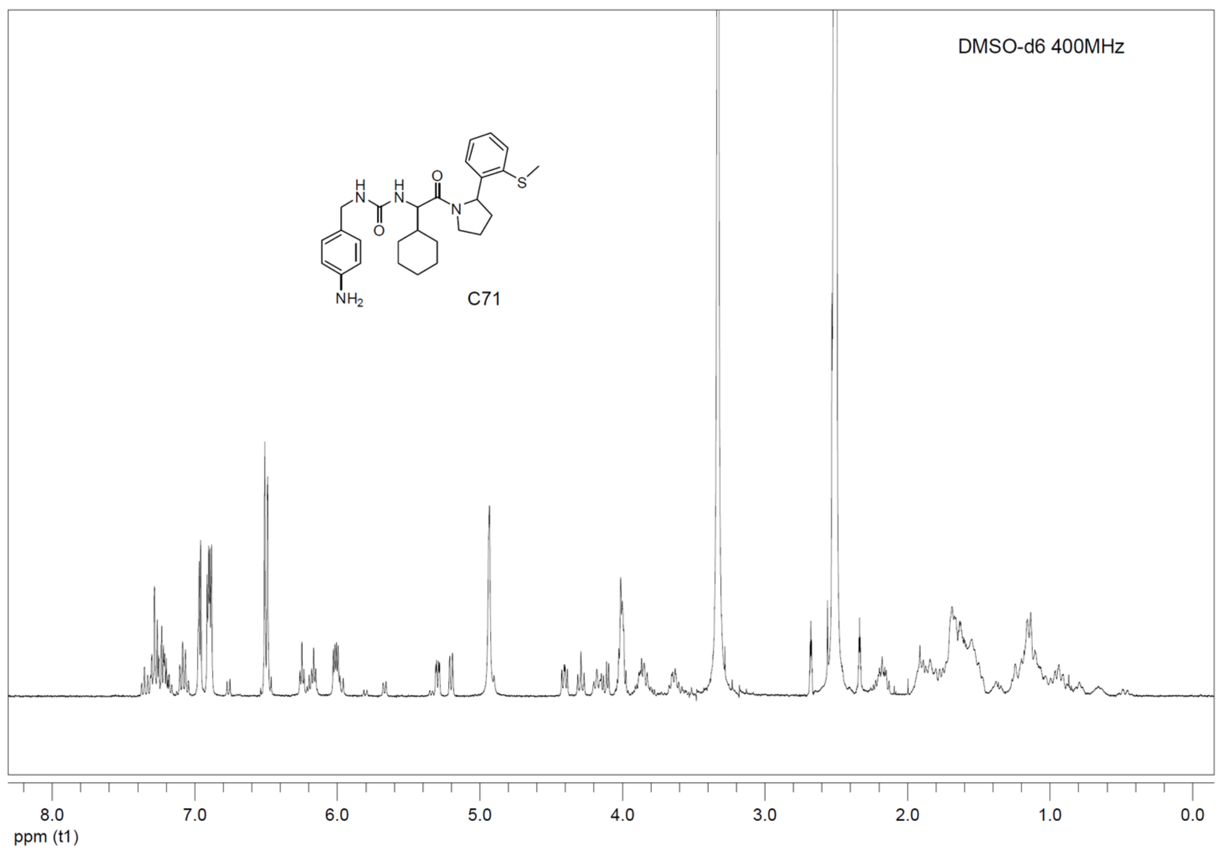

^{1}H NMR (400 MHz, DMSO-d6) spectrum of compound C71.

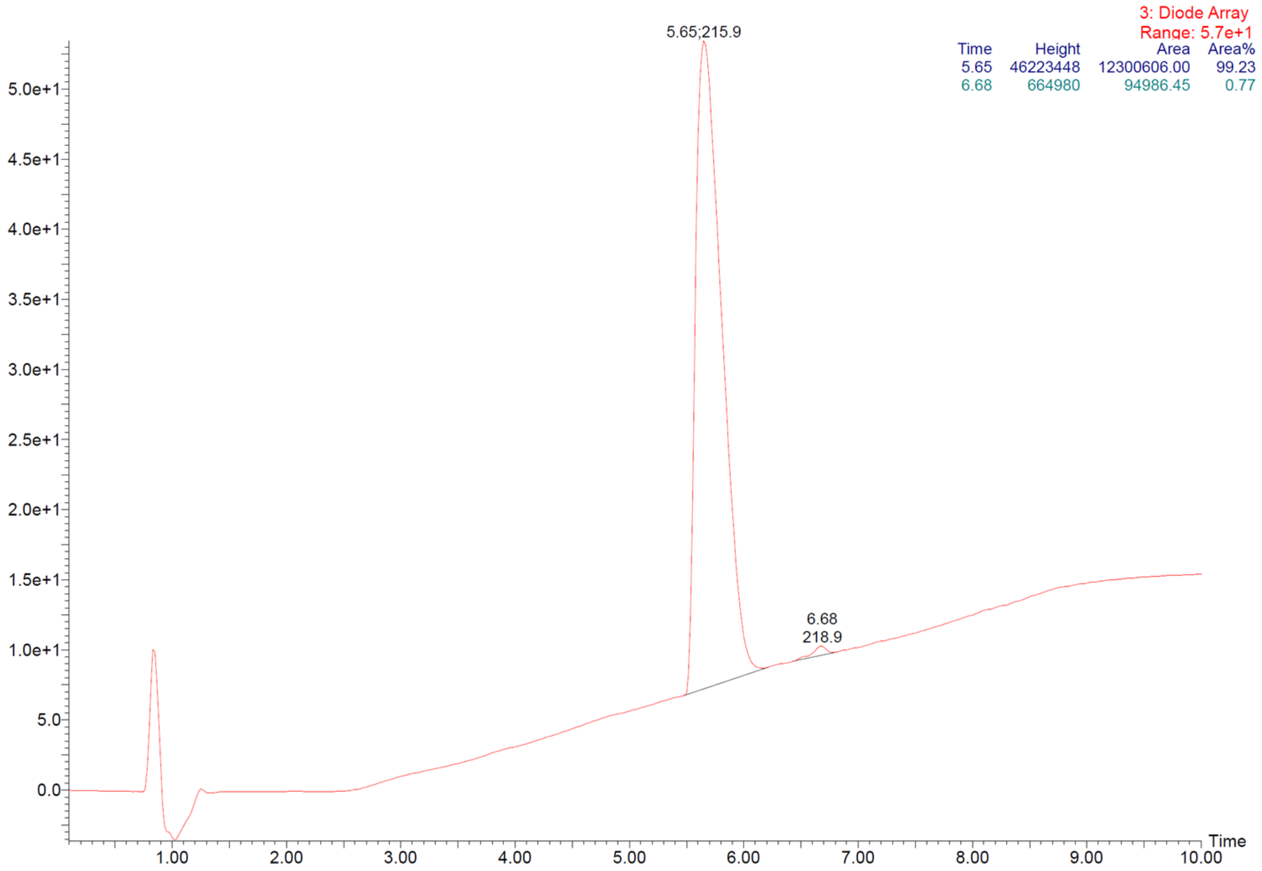

HPLC Chromatogram of compound C71.

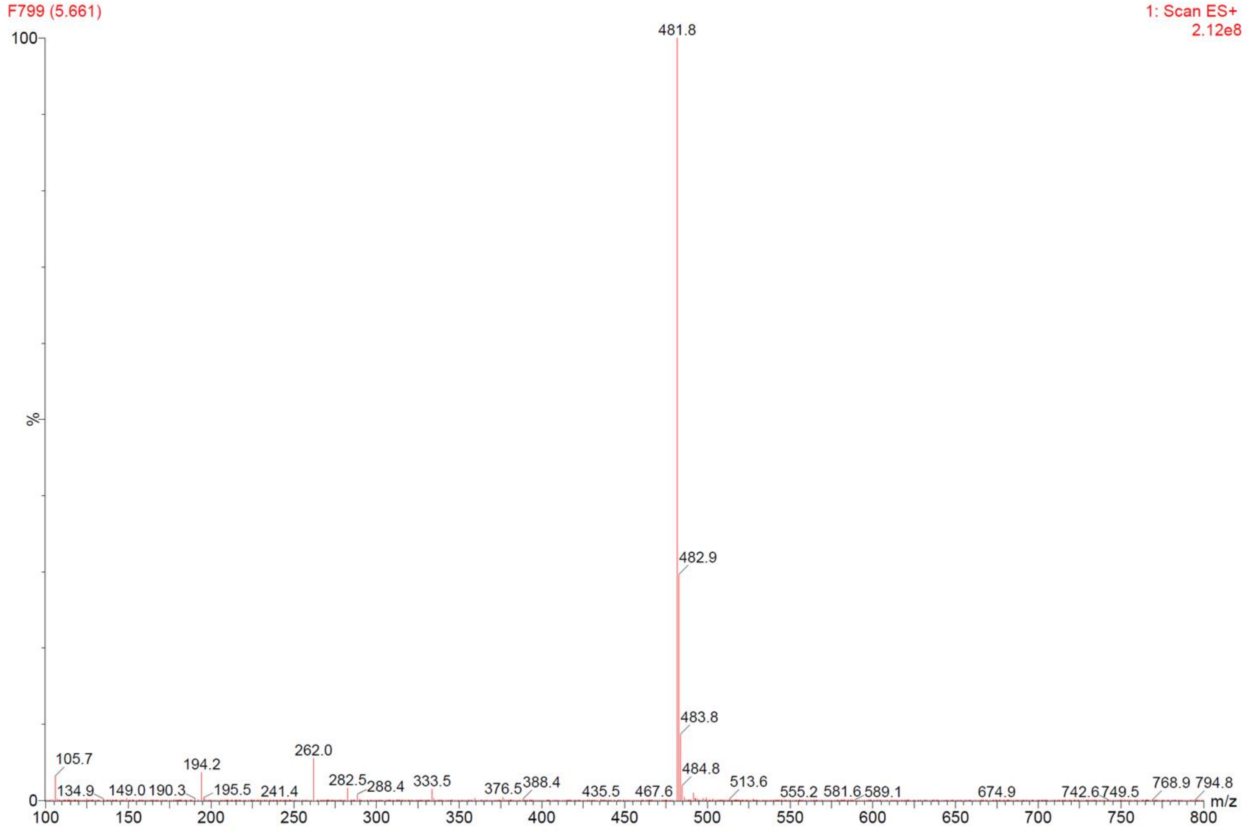

Mass spectrum of compound C71

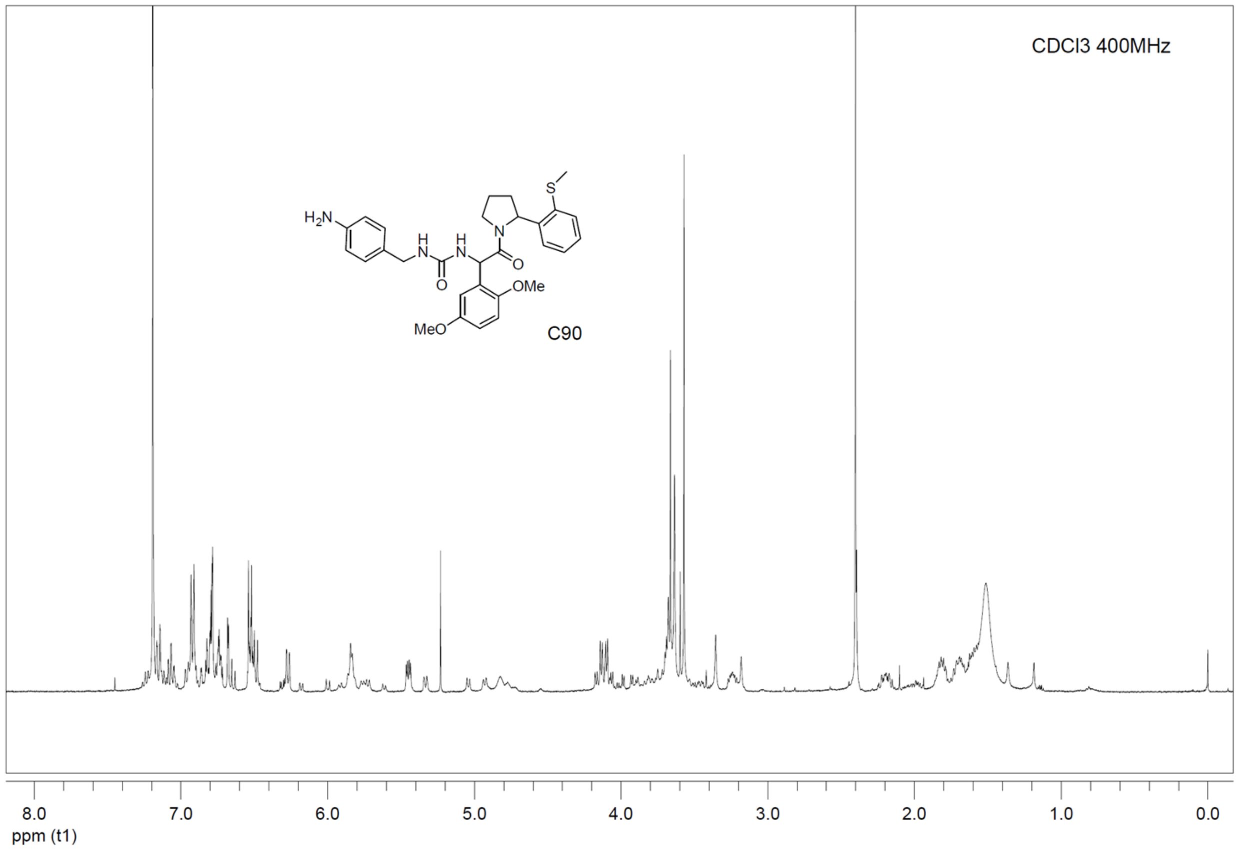

^{1}H NMR (400 MHz, $CDCl_3$) spectrum of compound C90.

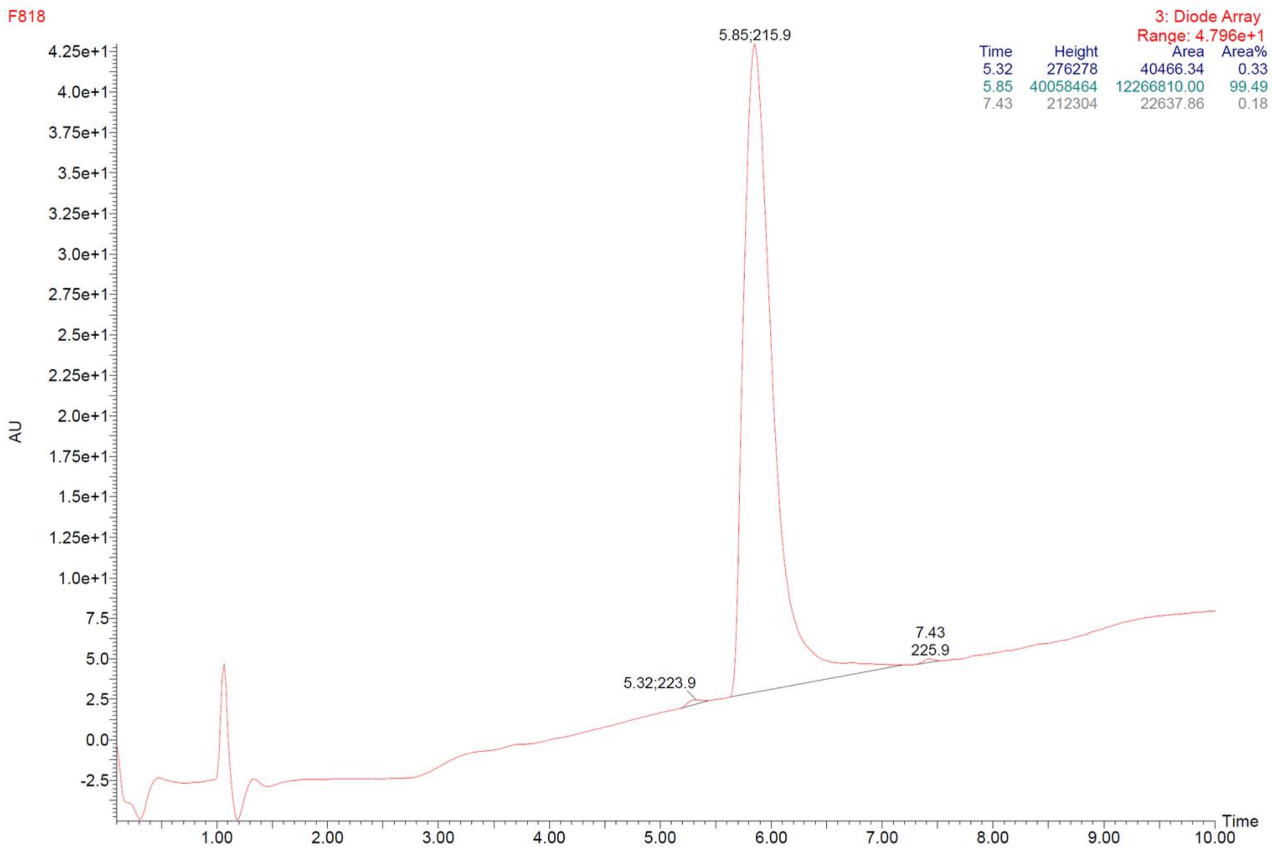

HPLC Chromatogram of compound C90.

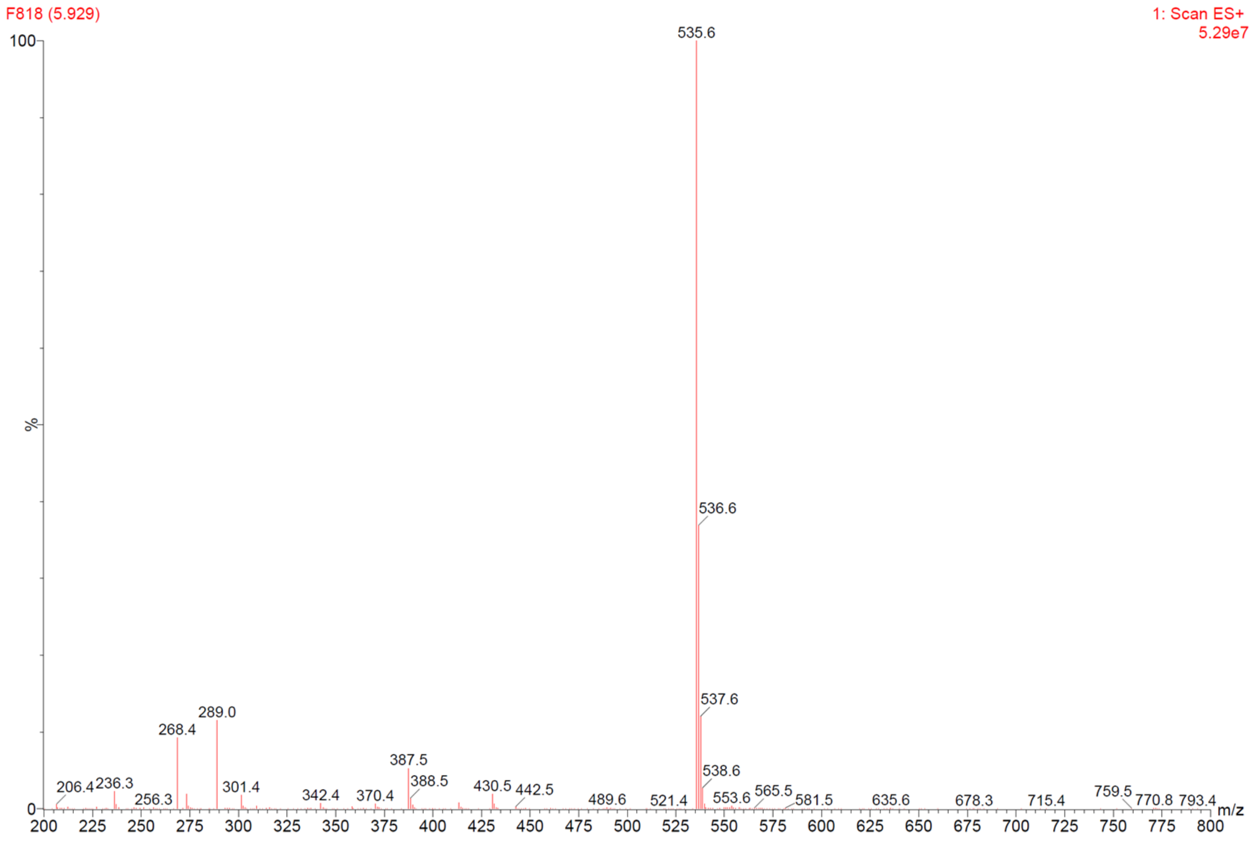

Mass spectrum of compound C90.

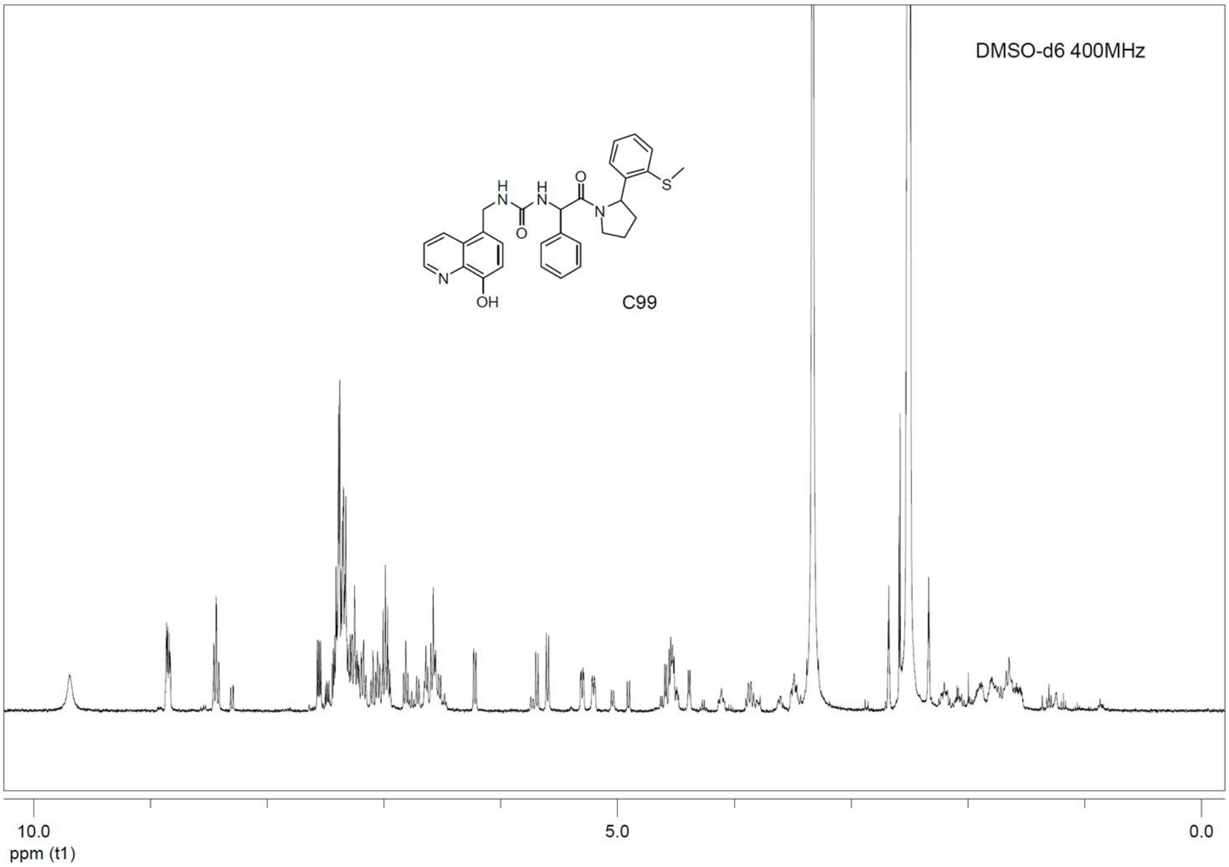

^{1}H NMR (400 MHz, DMSO-d6) spectrum of compound C99.

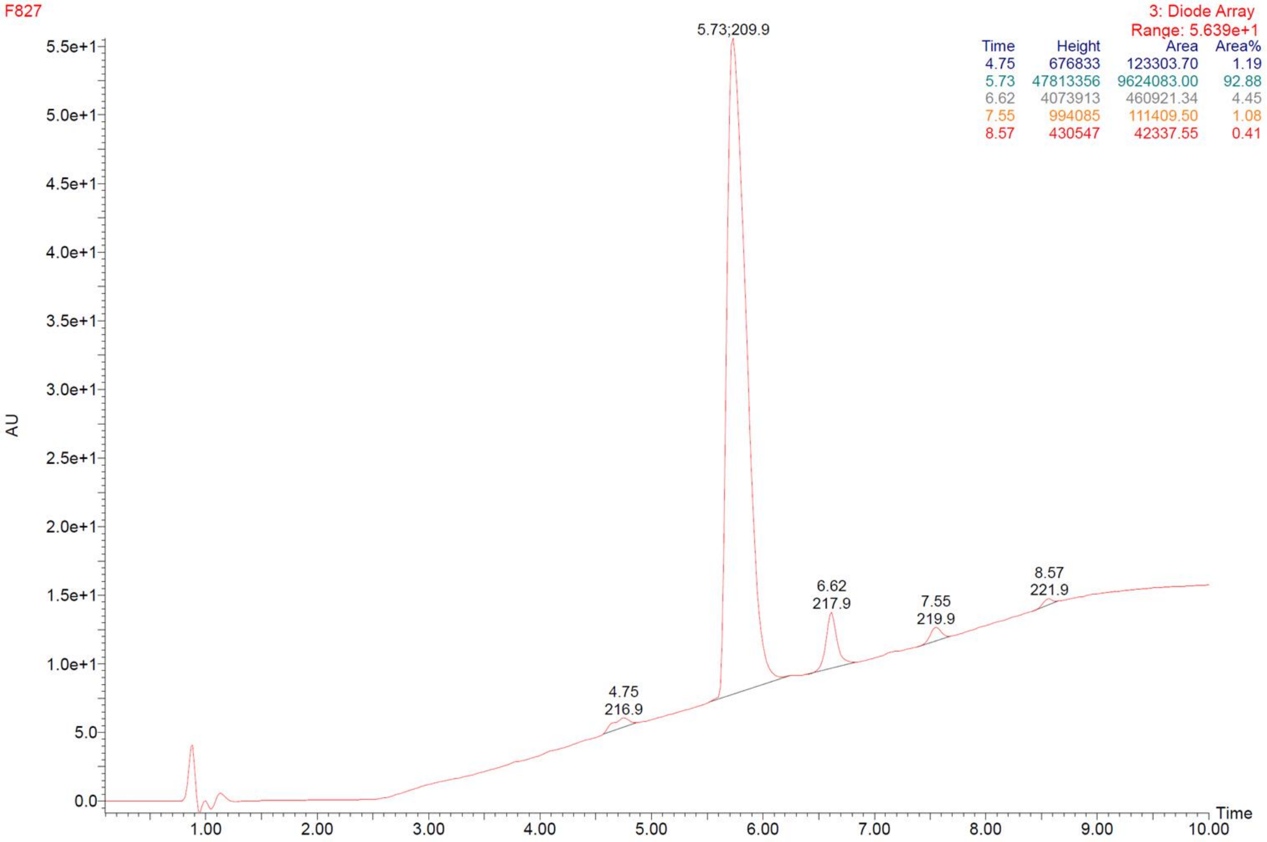

Time	Height	Area	Area%
4.75	676833	123303.70	1.19
5.73	47813356	9624083.00	92.88
6.62	4073913	460921.34	4.45
7.55	994085	111409.50	1.08
8.57	430547	42337.55	0.41

HPLC Chromatogram of compound C99.

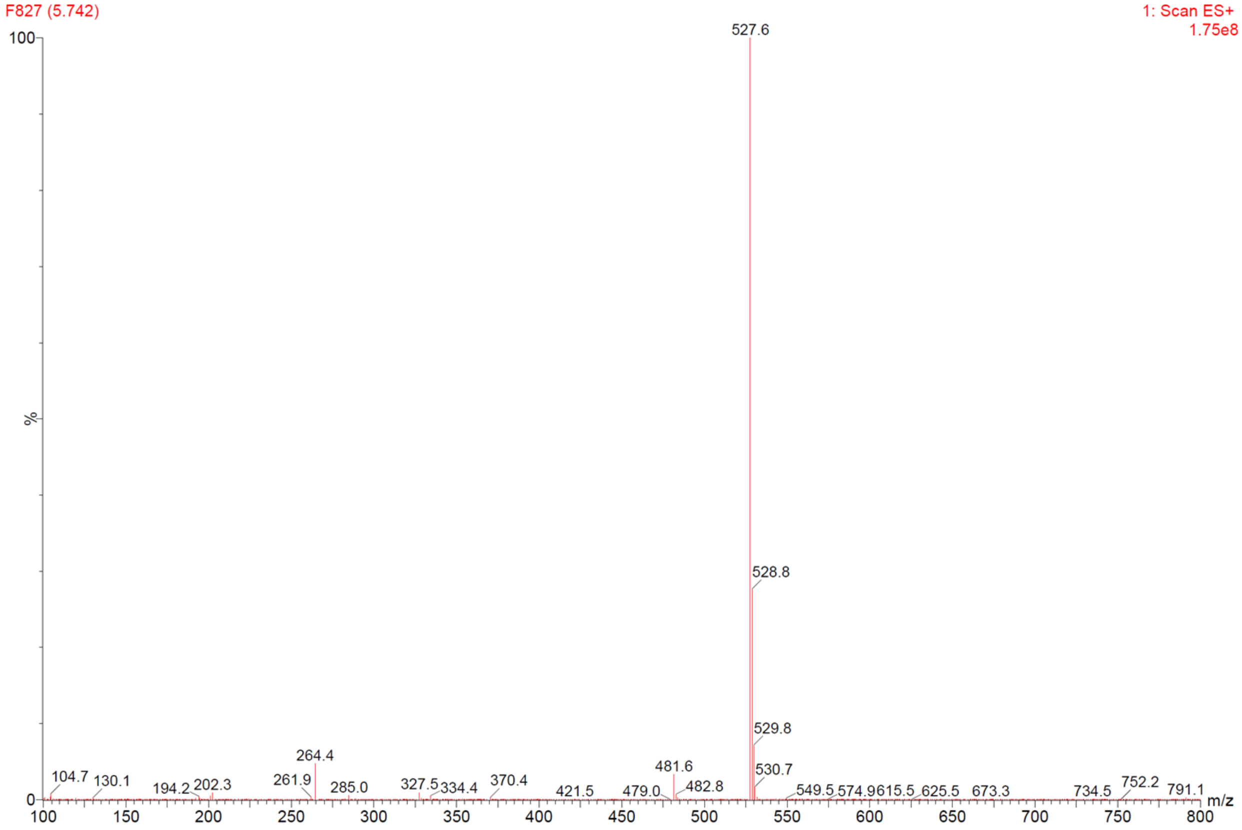

Mass spectrum of compound C99.

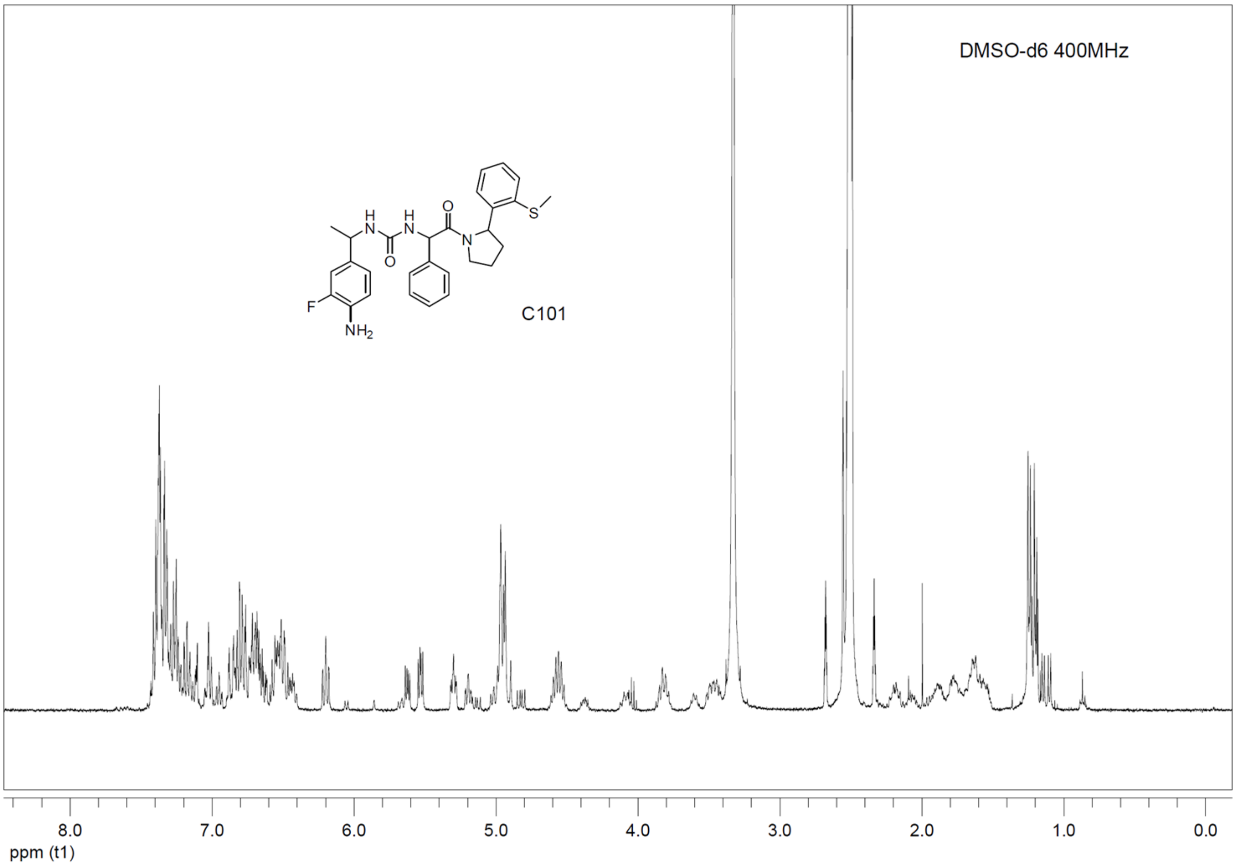

^{1}H NMR (400 MHz, DMSO-d6) spectrum of compound C101.

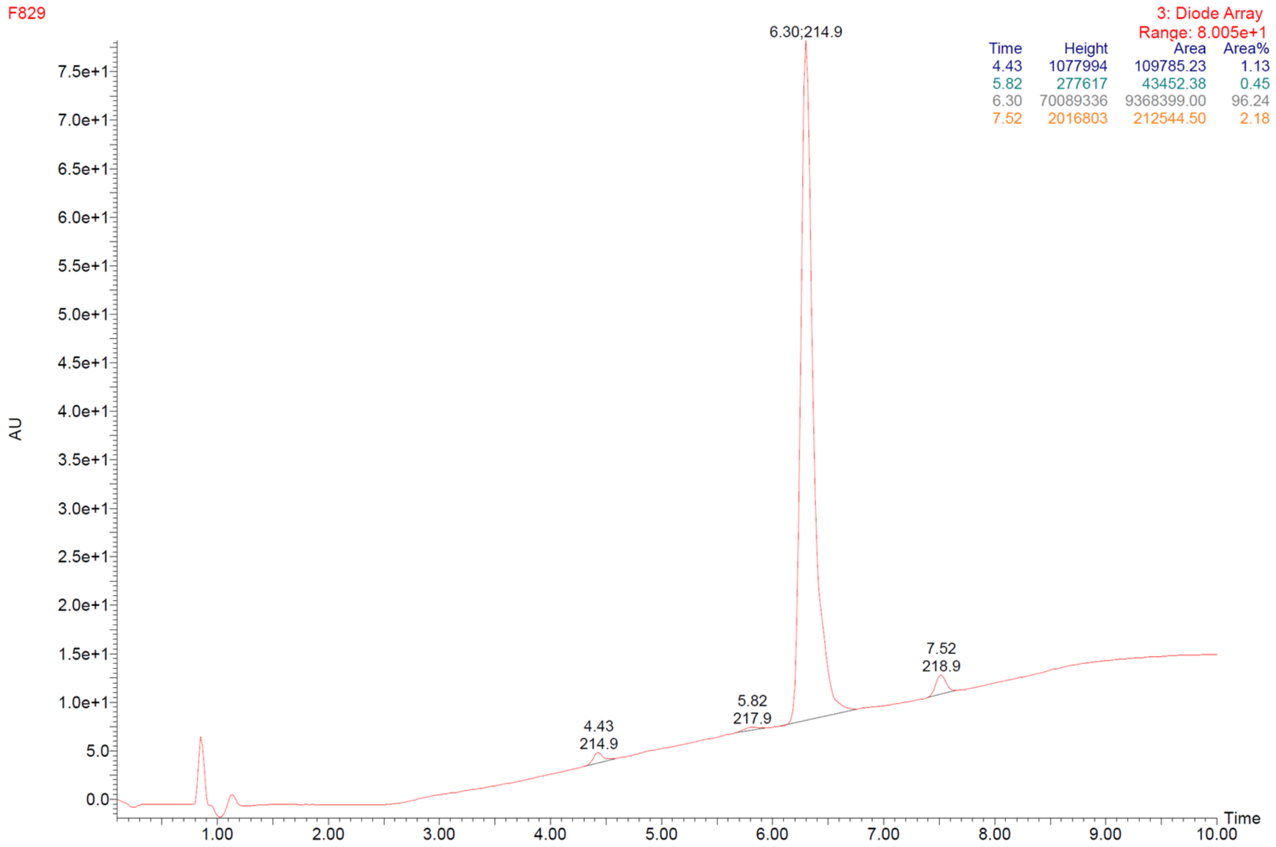

HPLC Chromatogram of compound C101.

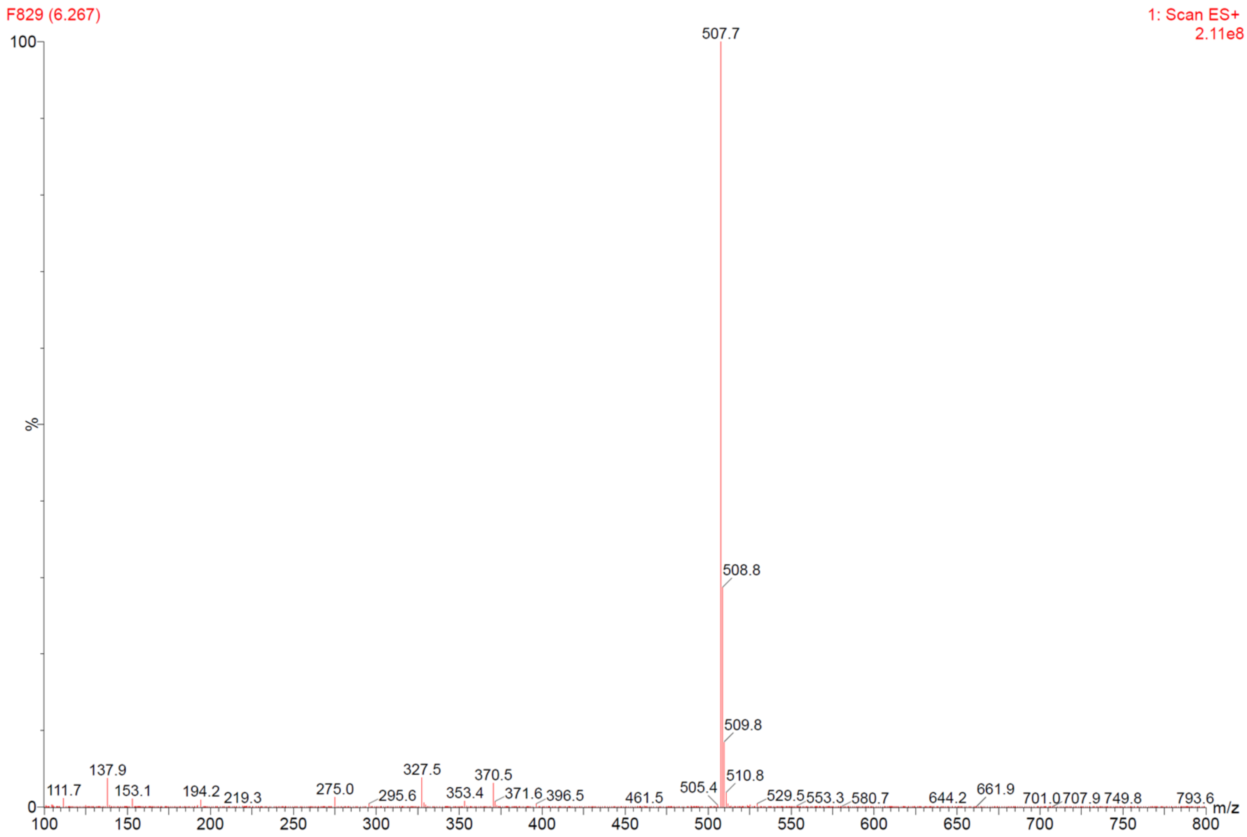

Mass spectrum of compound C101.

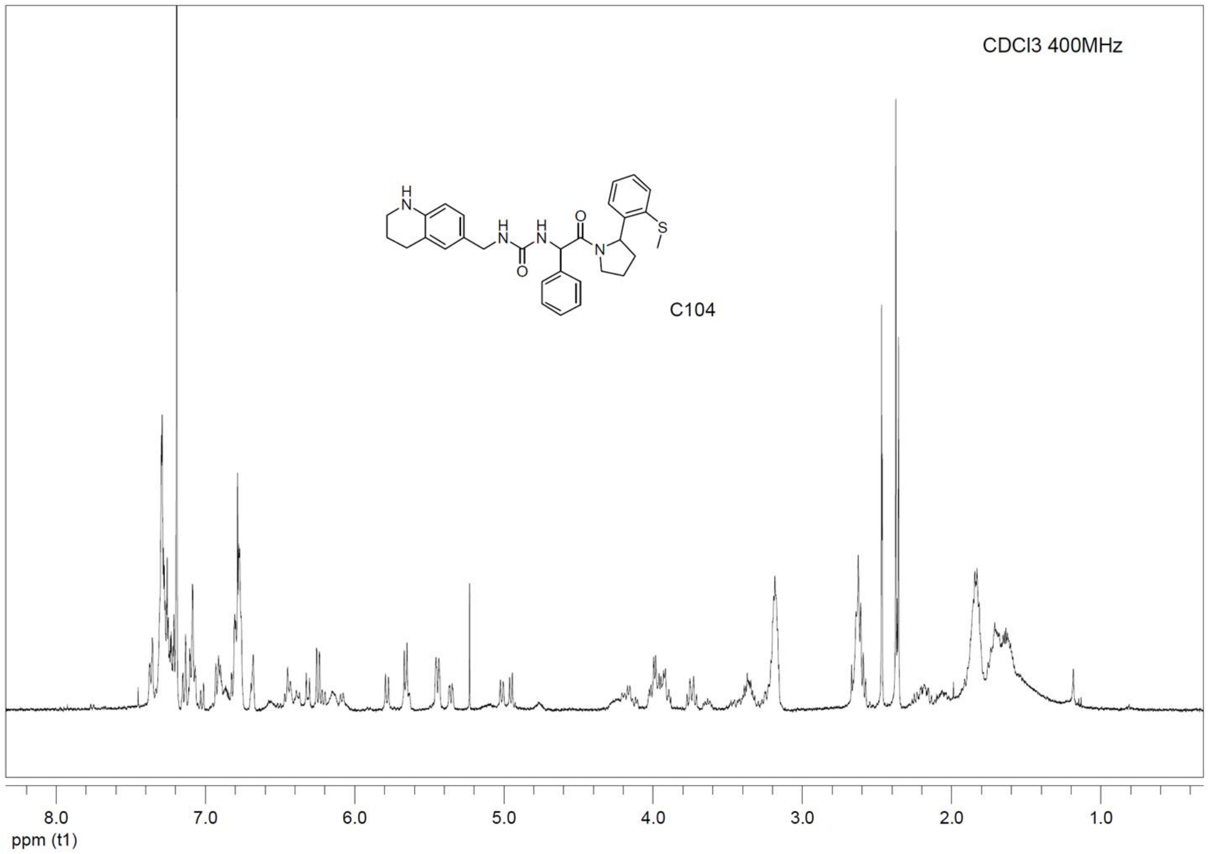

^{1}H NMR (400 MHz, $CDCl_3$) spectrum of compound C104.

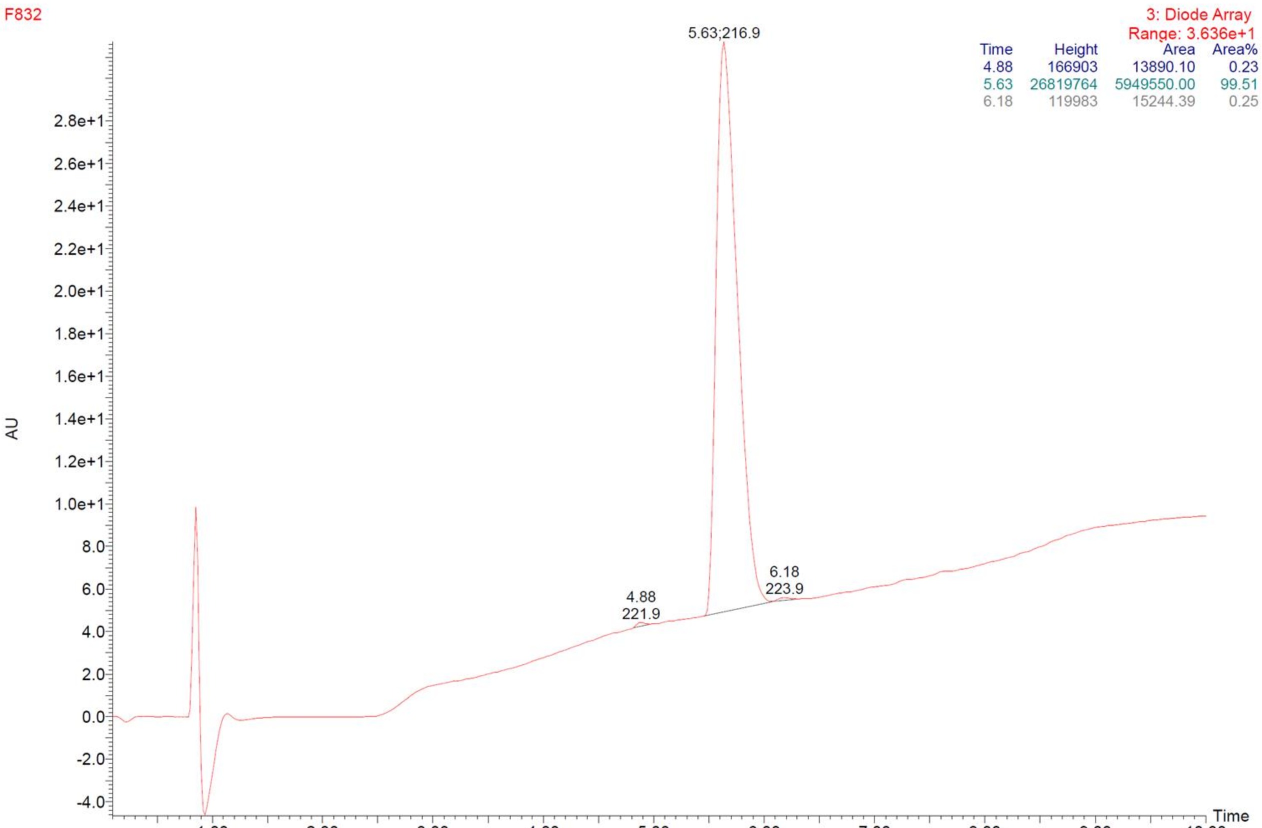

HPLC Chromatogram of compound C104.

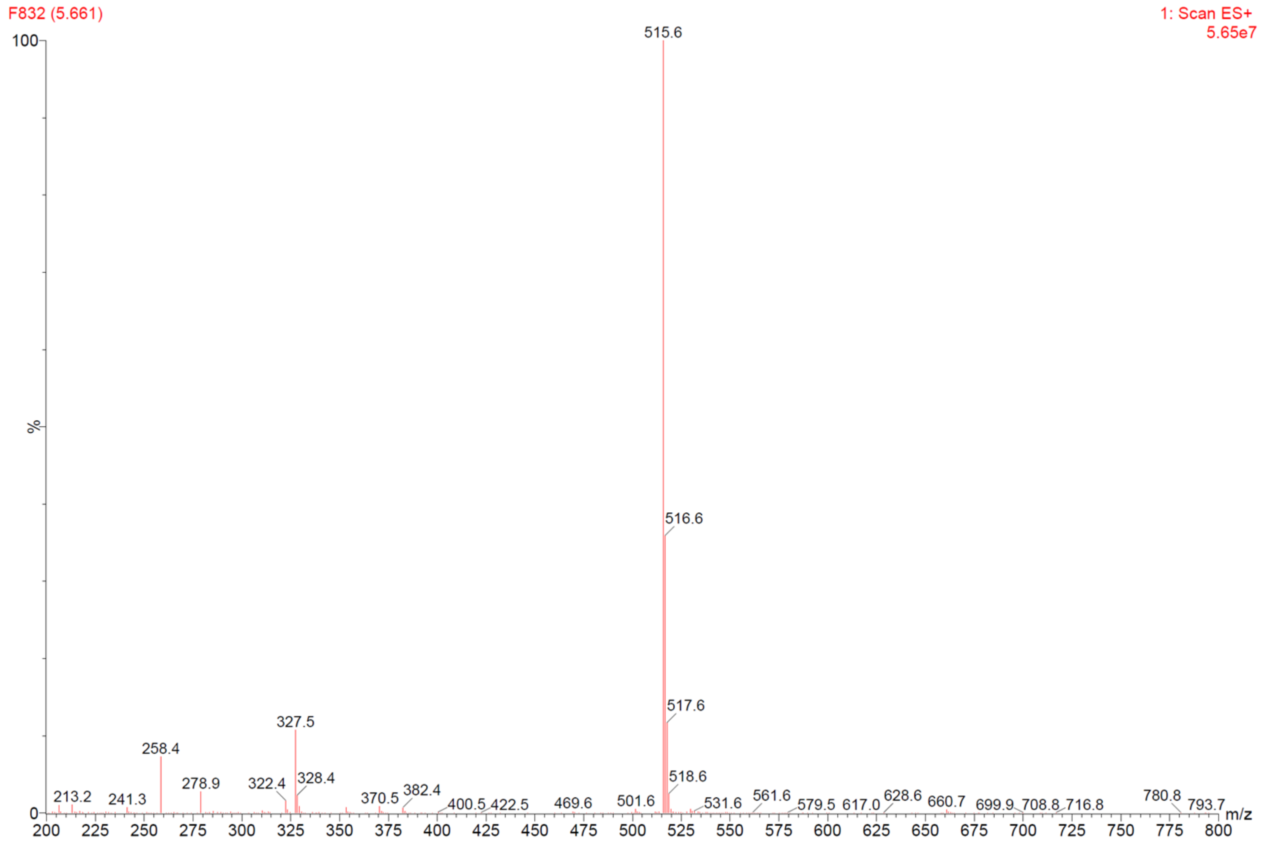

Mass spectrum of compound C104.

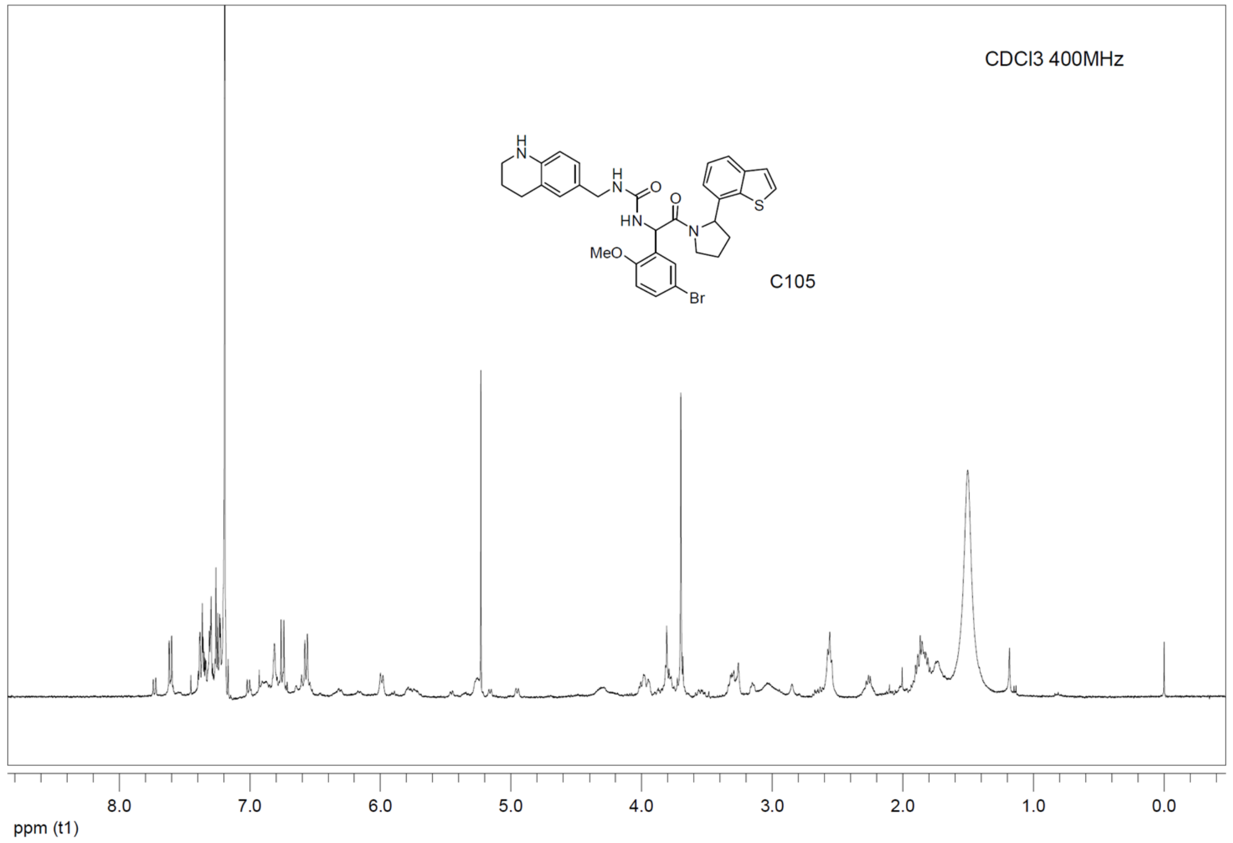

^{1}H NMR (400 MHz, $CDCl_3$) spectrum of compound C105.

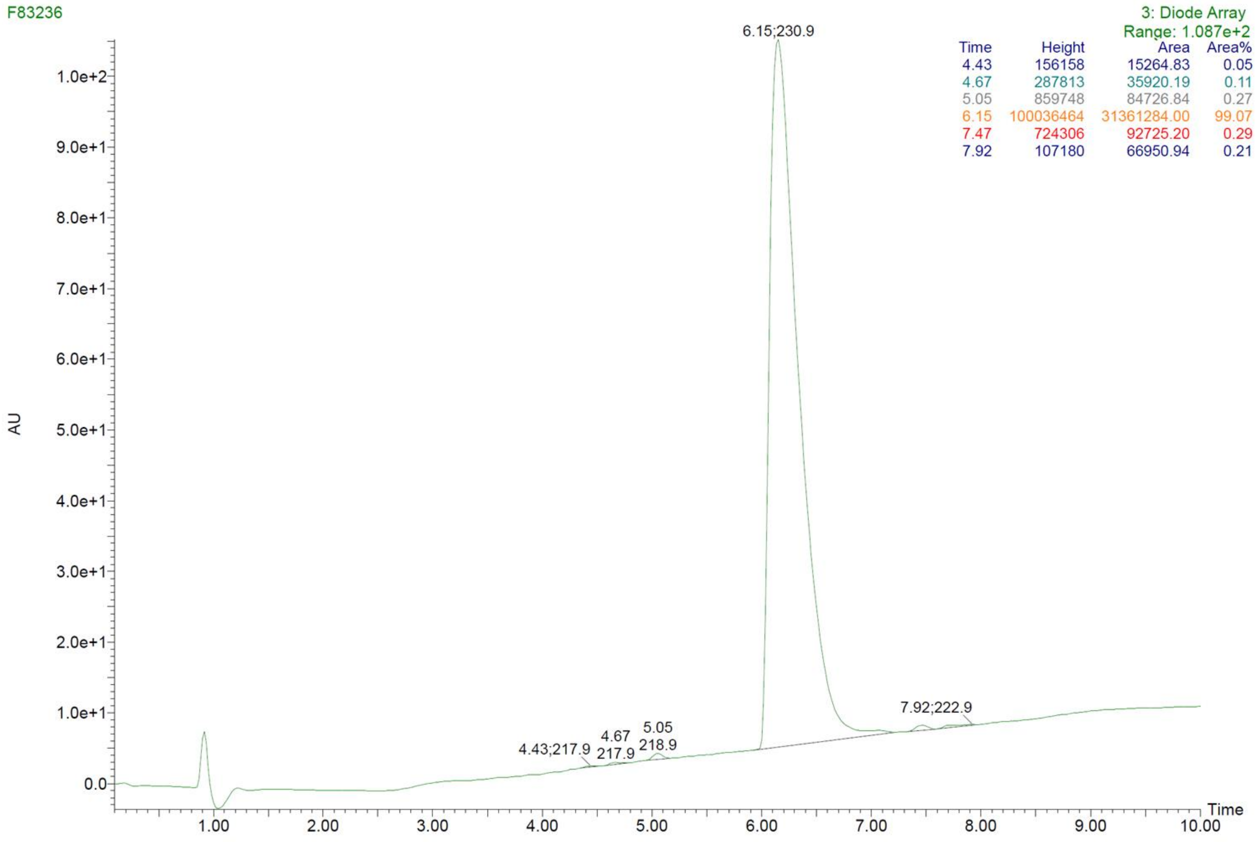

HPLC Chromatogram of compound C105.

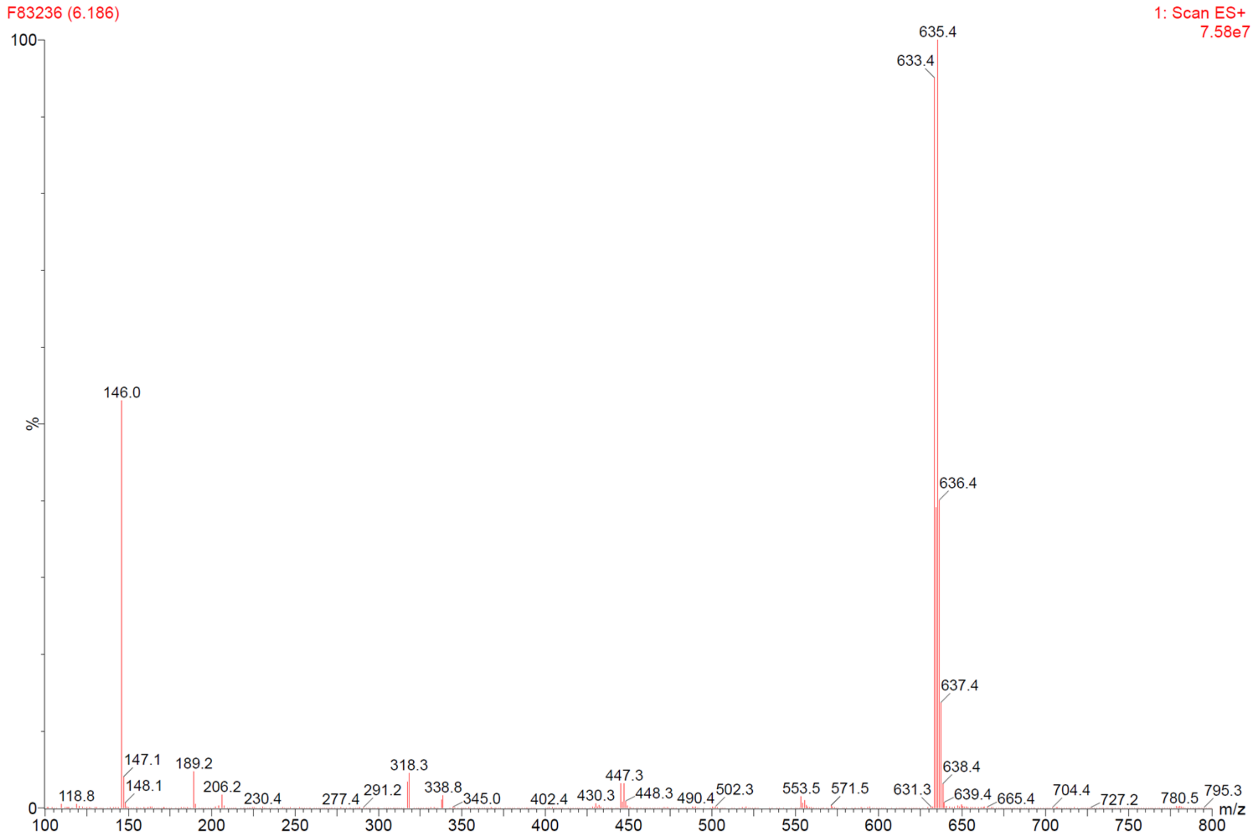

Mass spectrum of compound C105.

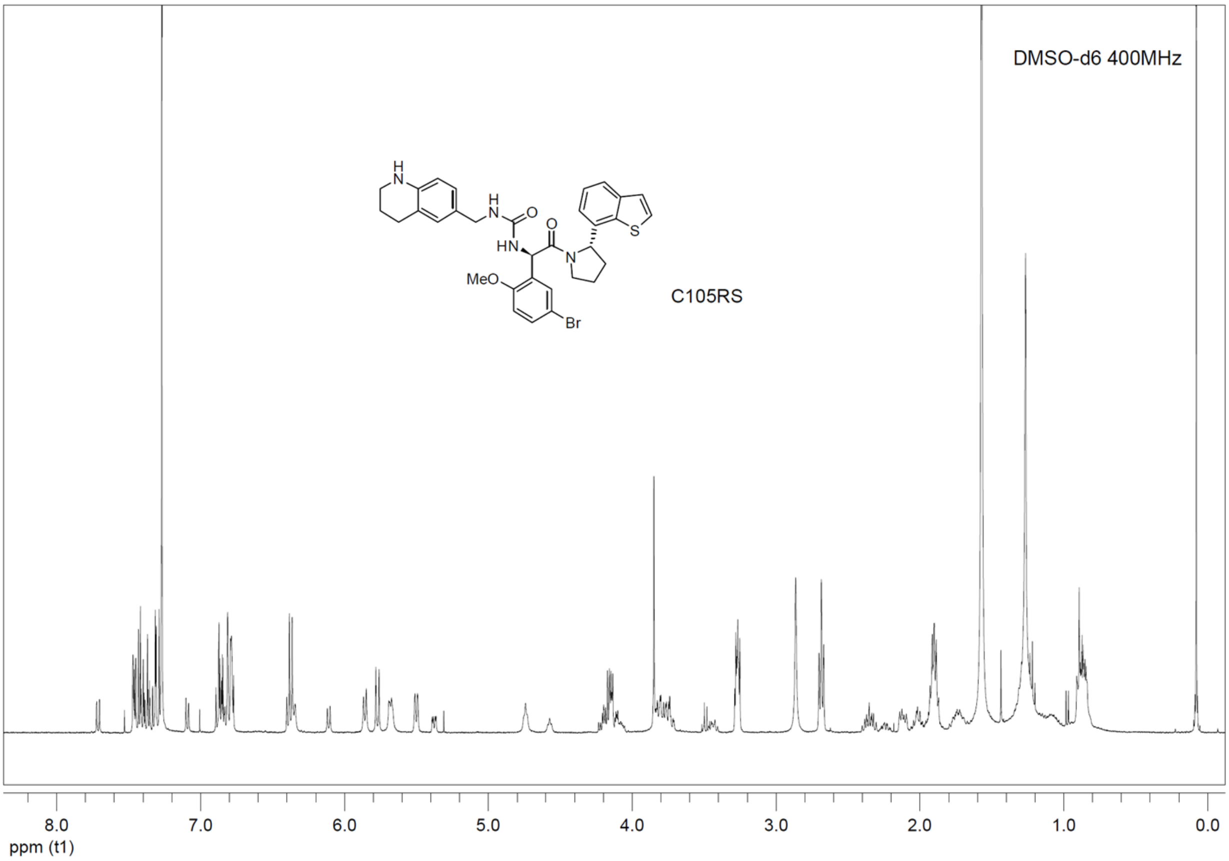

^{1}H NMR (400 MHz, DMSO-d6) spectrum of compound C105RS.

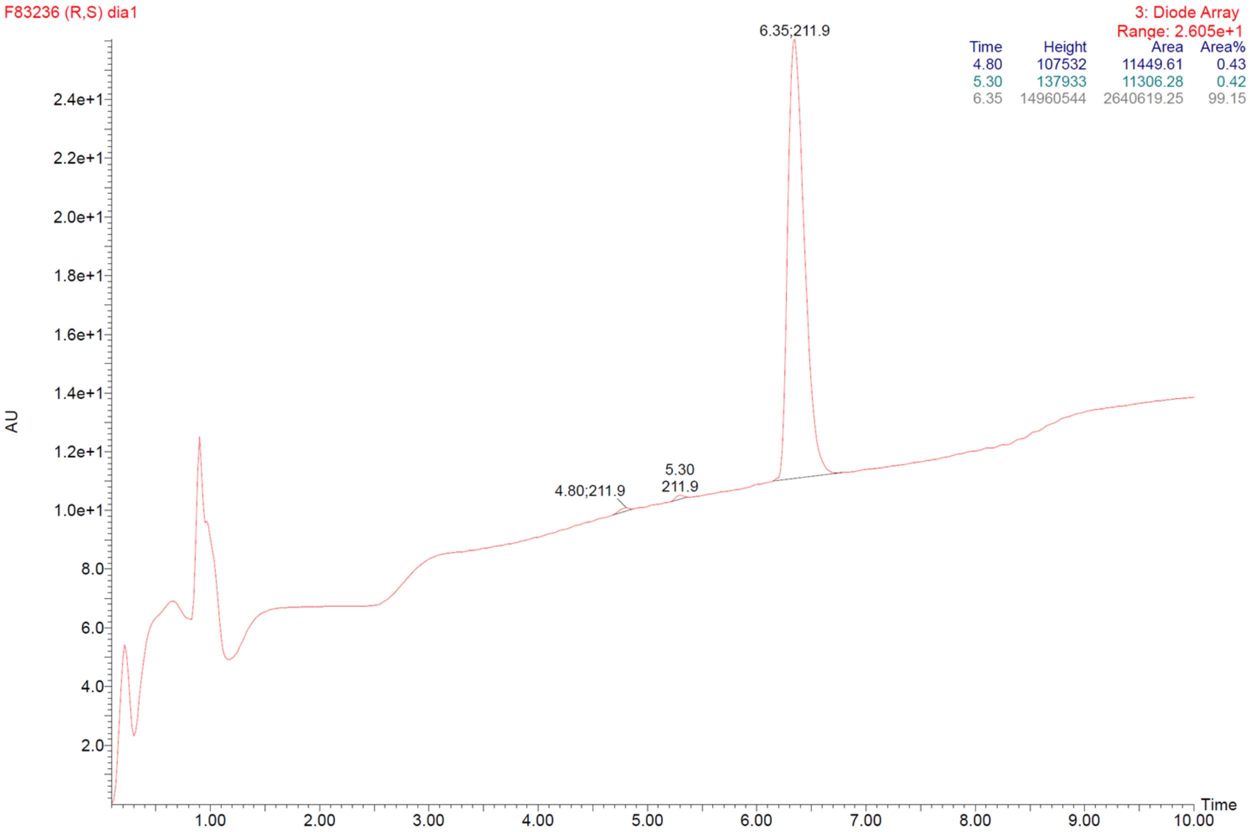

Time	Height	Area	Area%
4.80	107532	11449.61	0.43
5.30	137933	11306.28	0.42
6.35	14960544	2640619.25	99.15

HPLC Chromatogram of compound C105RS.

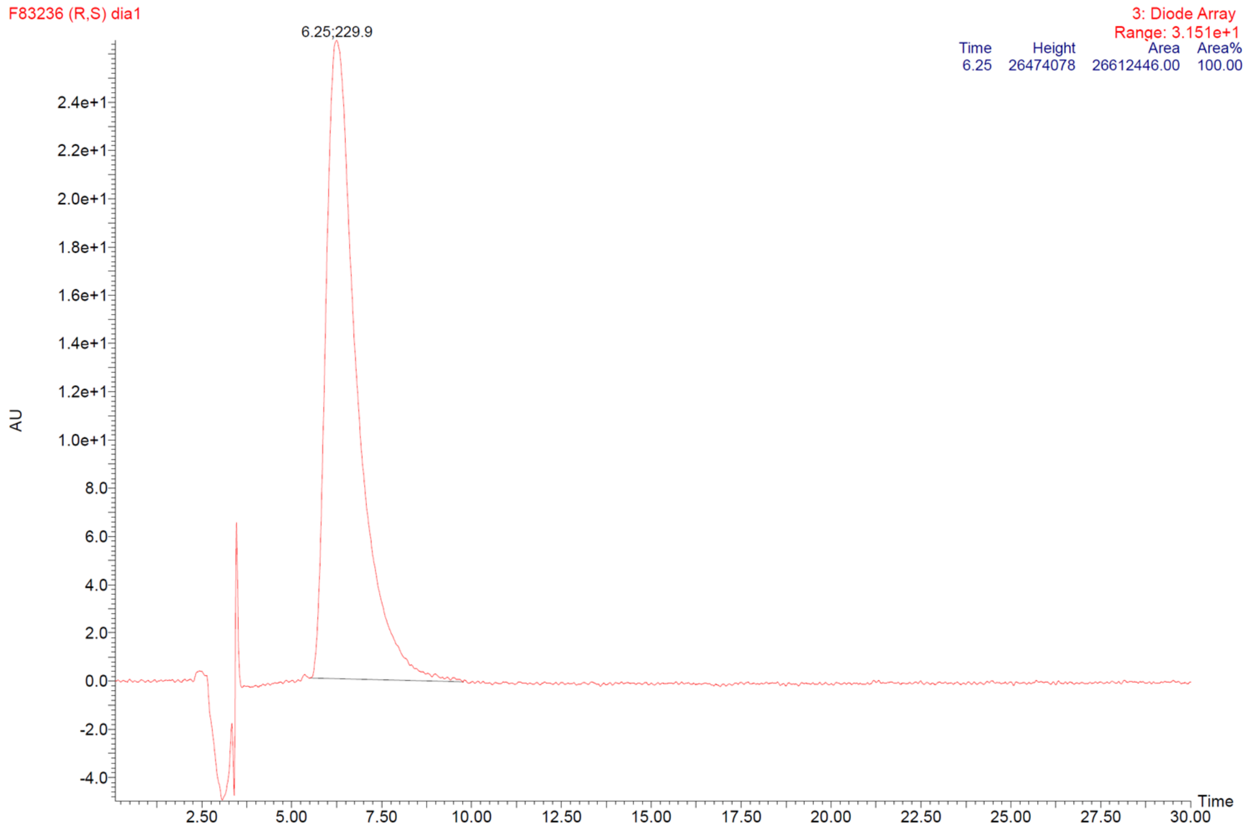

Chiral HPLC Chromatogram of compound C105RS.

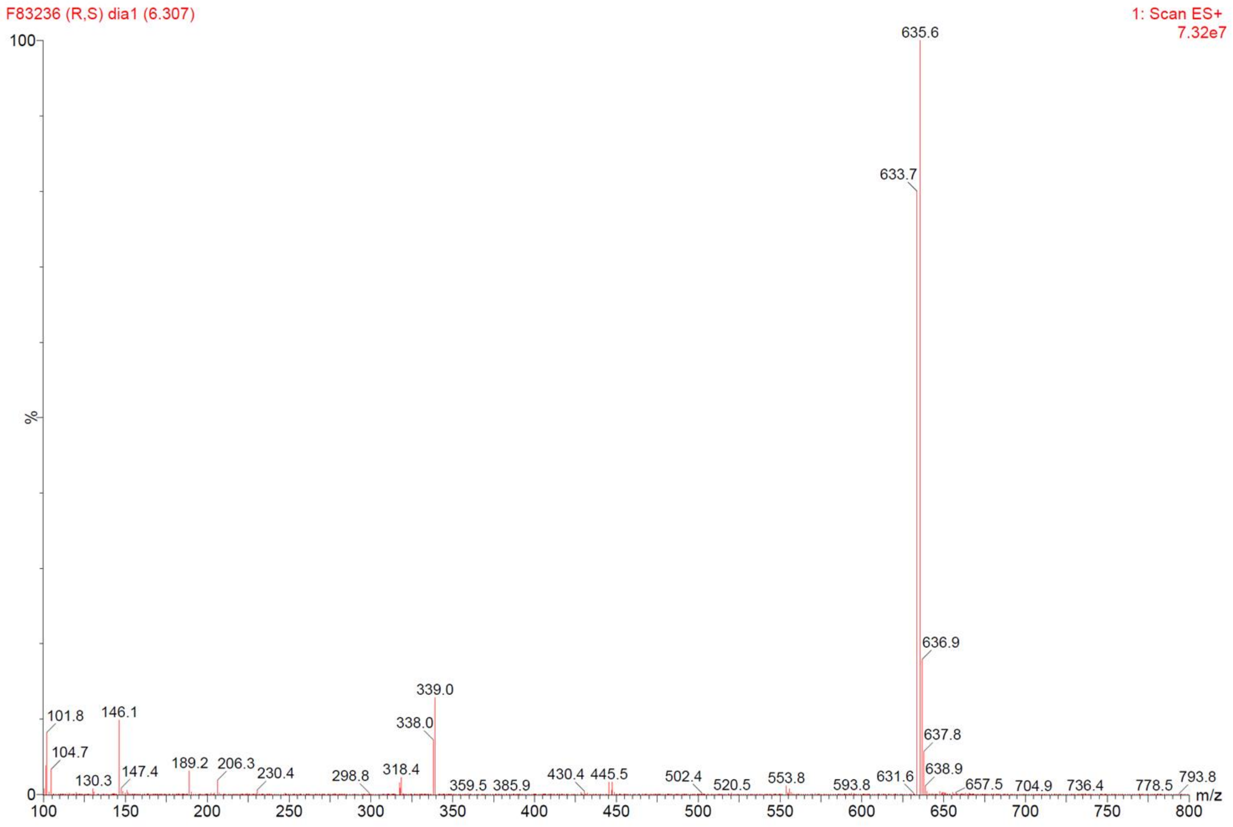

Mass spectrum of compound C105RS.

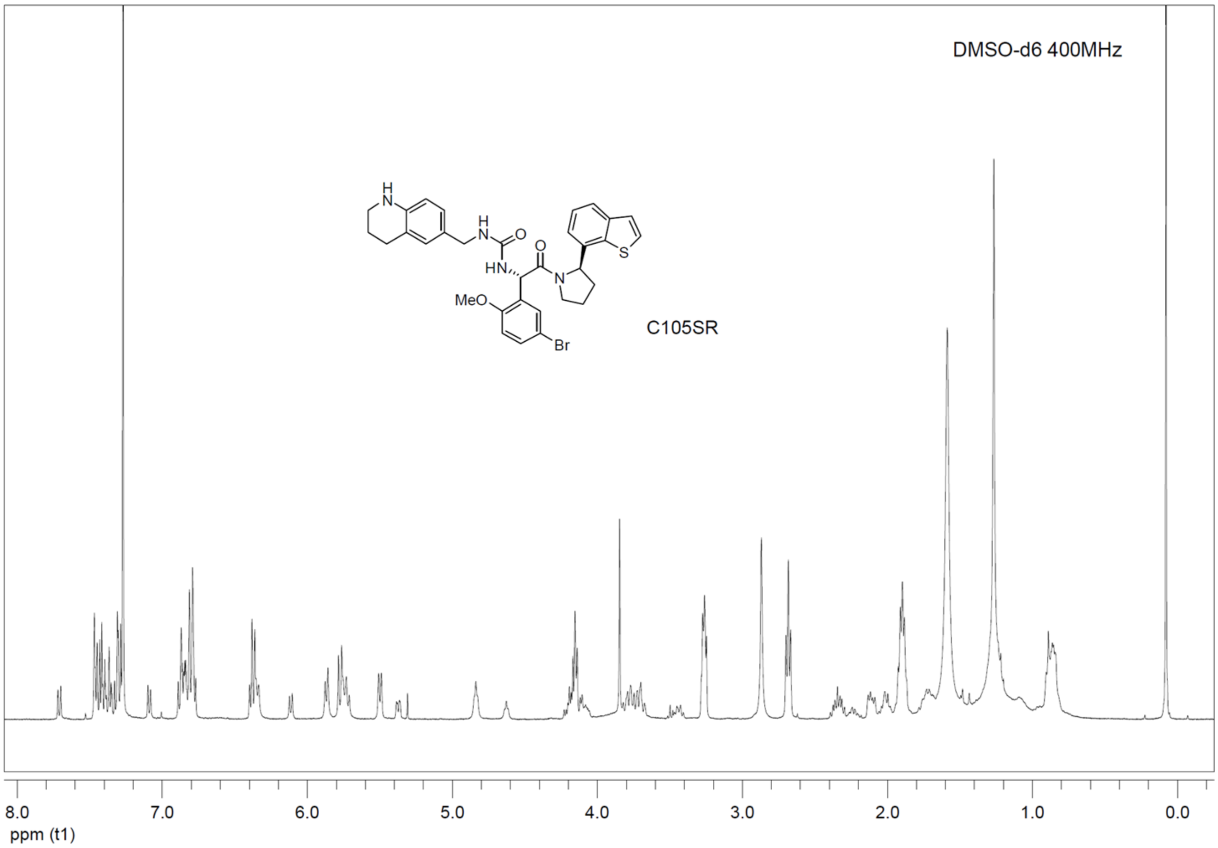

^{1}H NMR (400 MHz, DMSO-d6) spectrum of compound C105SR.

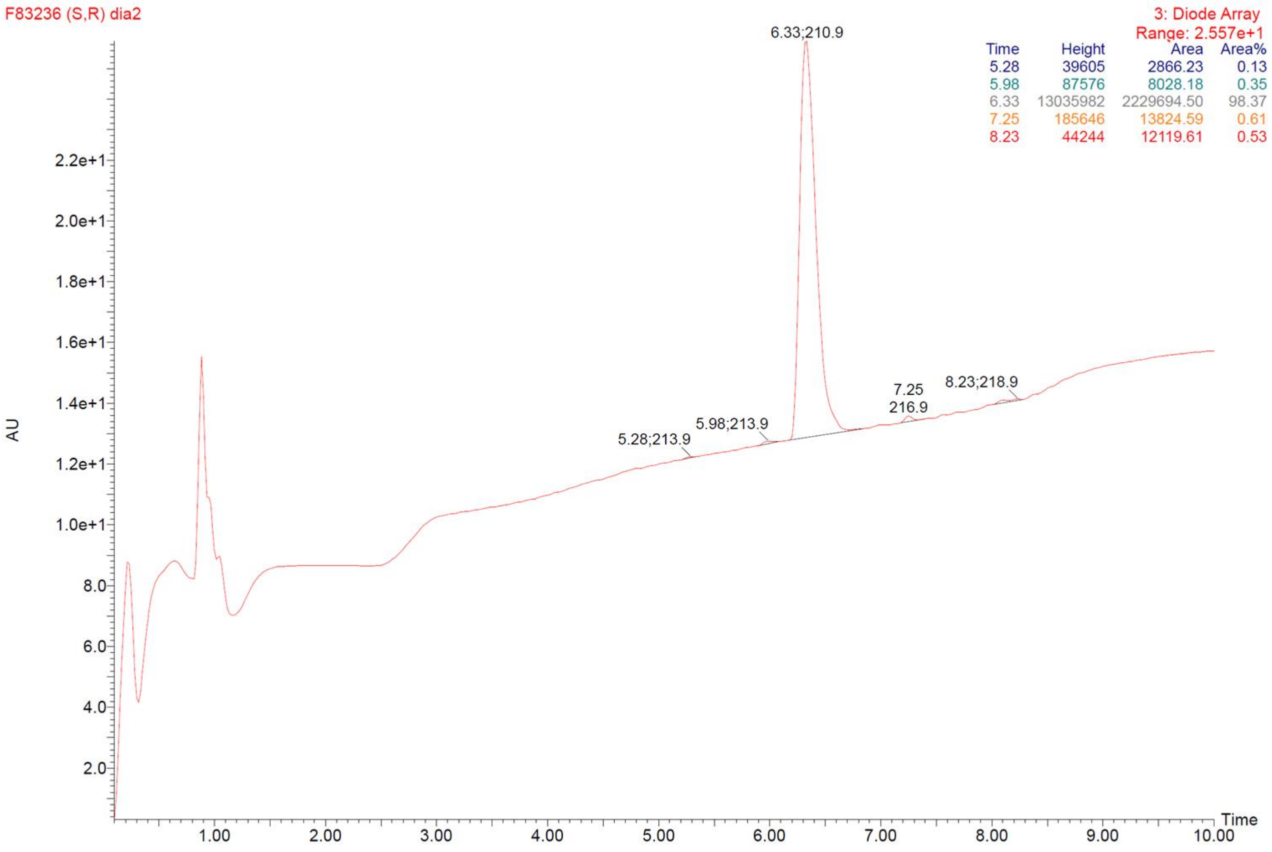

HPLC Chromatogram of compound C105SR.

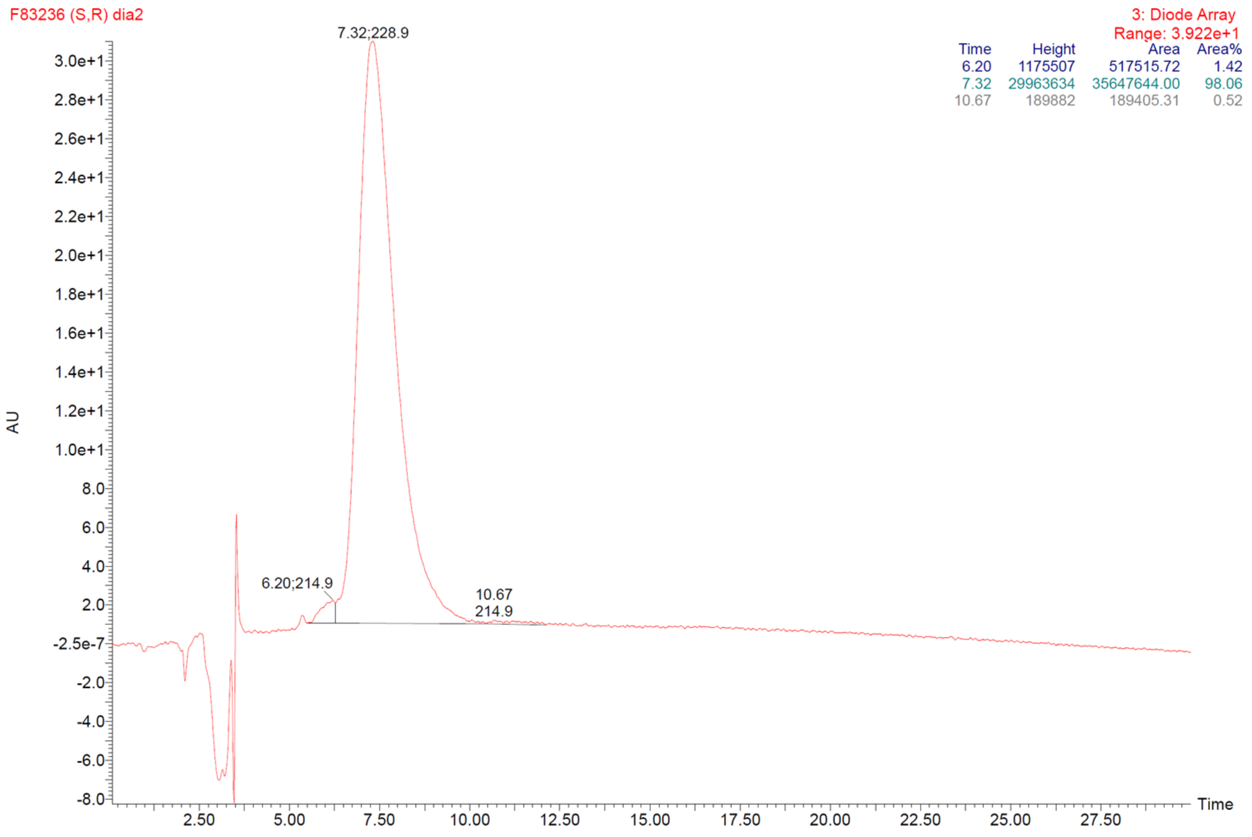

Chiral HPLC Chromatogram of compound C105SR.

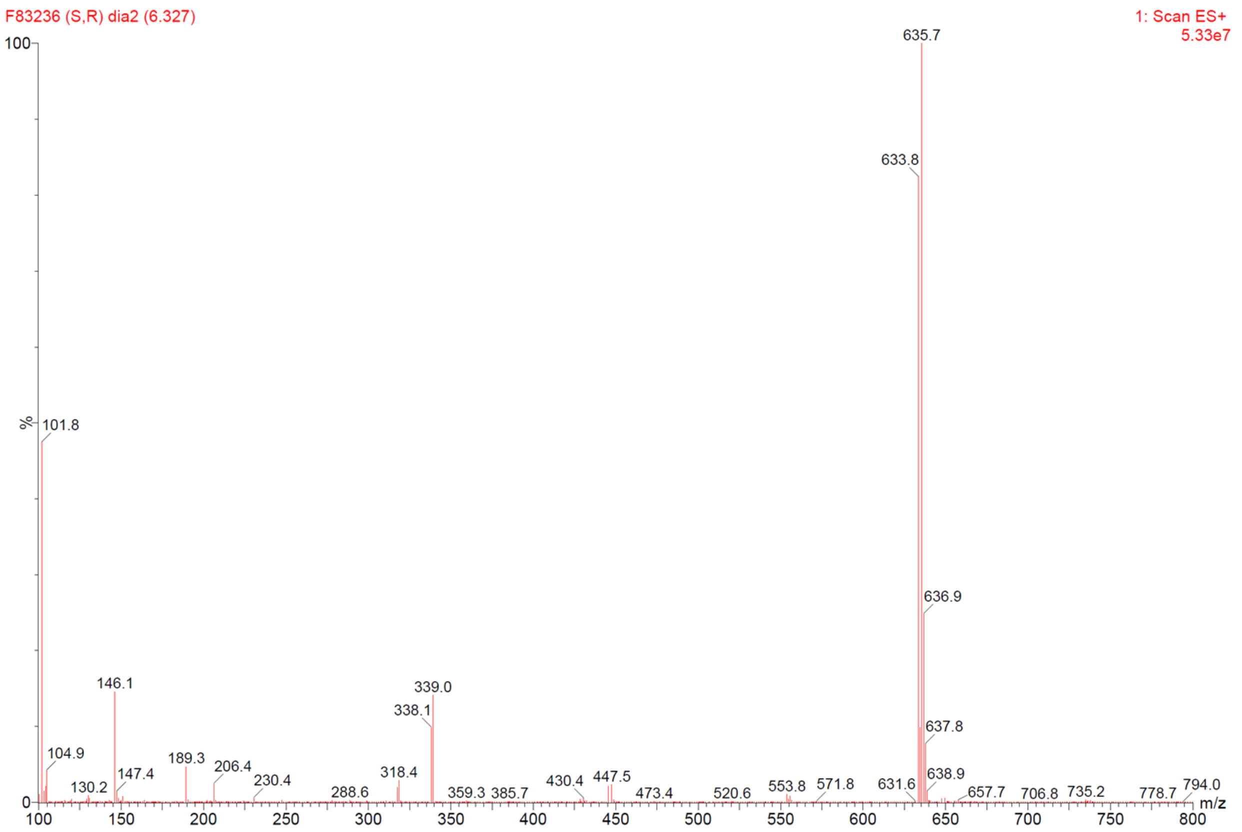

Mass spectrum of compound C105SR.

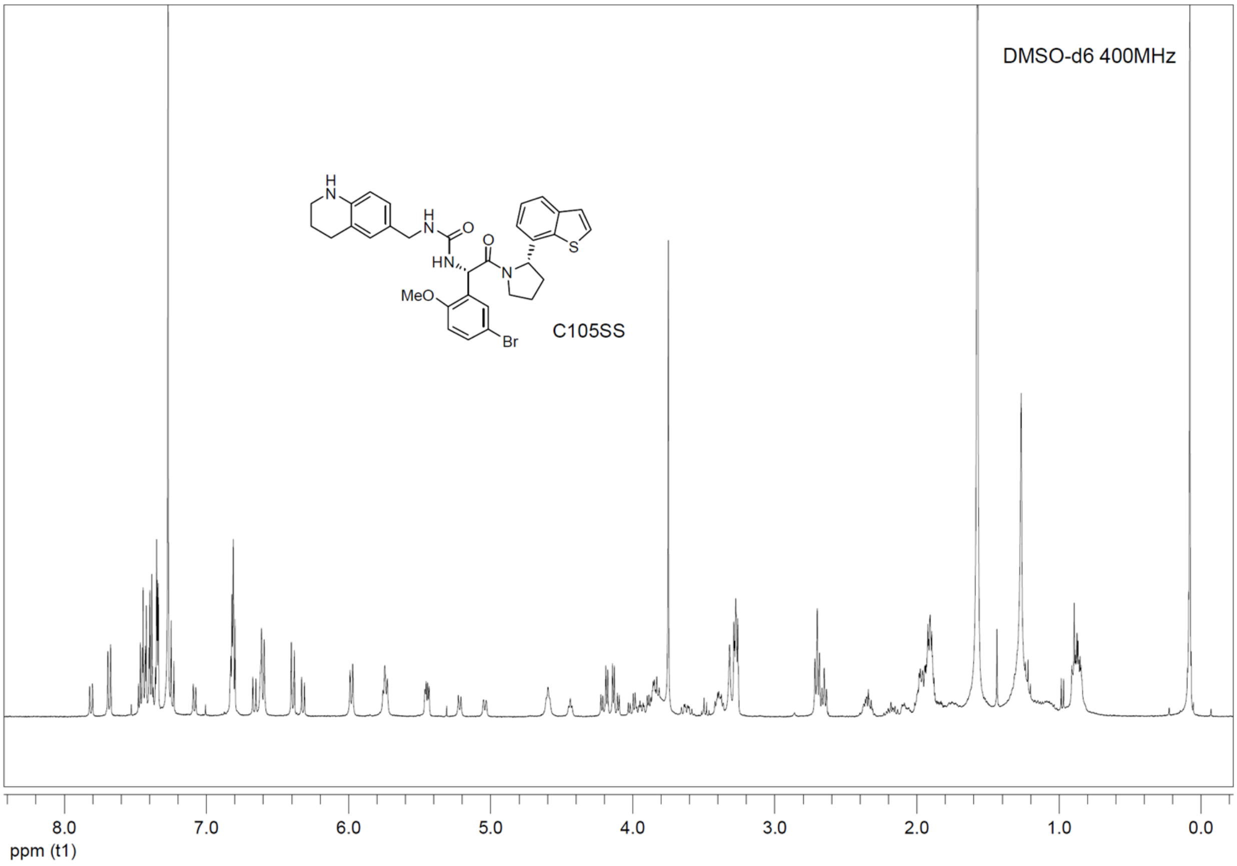

^{1}H NMR (400 MHz, DMSO-d6) spectrum of compound C105SS.

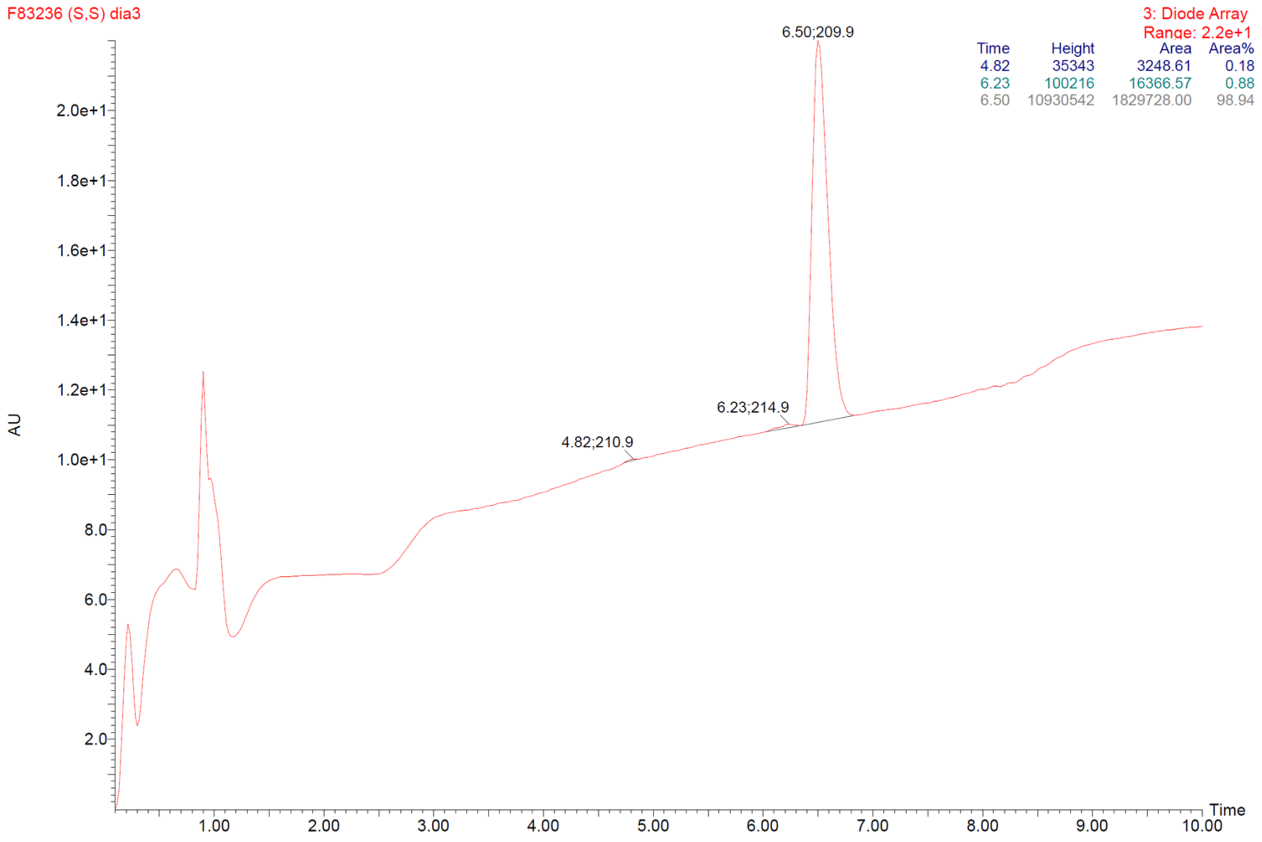

HPLC Chromatogram of compound C105SS.

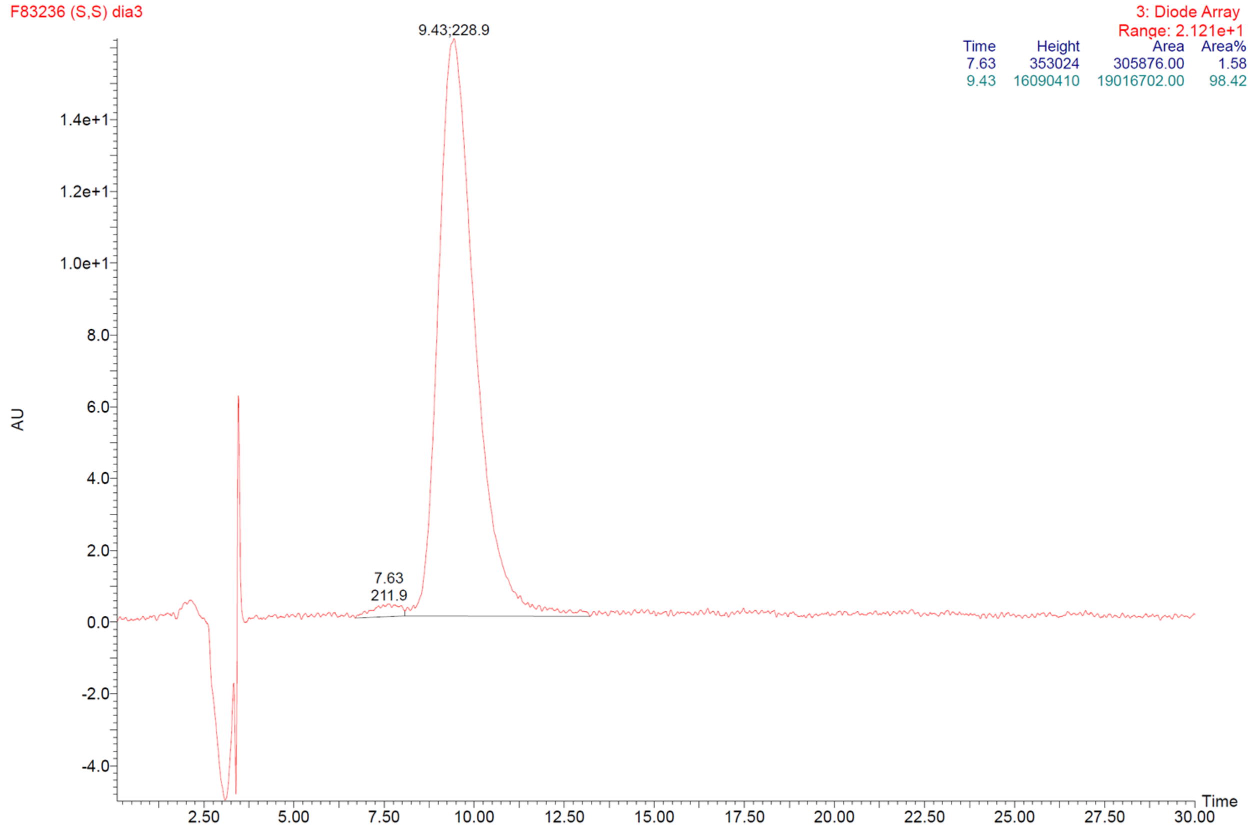

Chiral HPLC Chromatogram of compound C105SS.

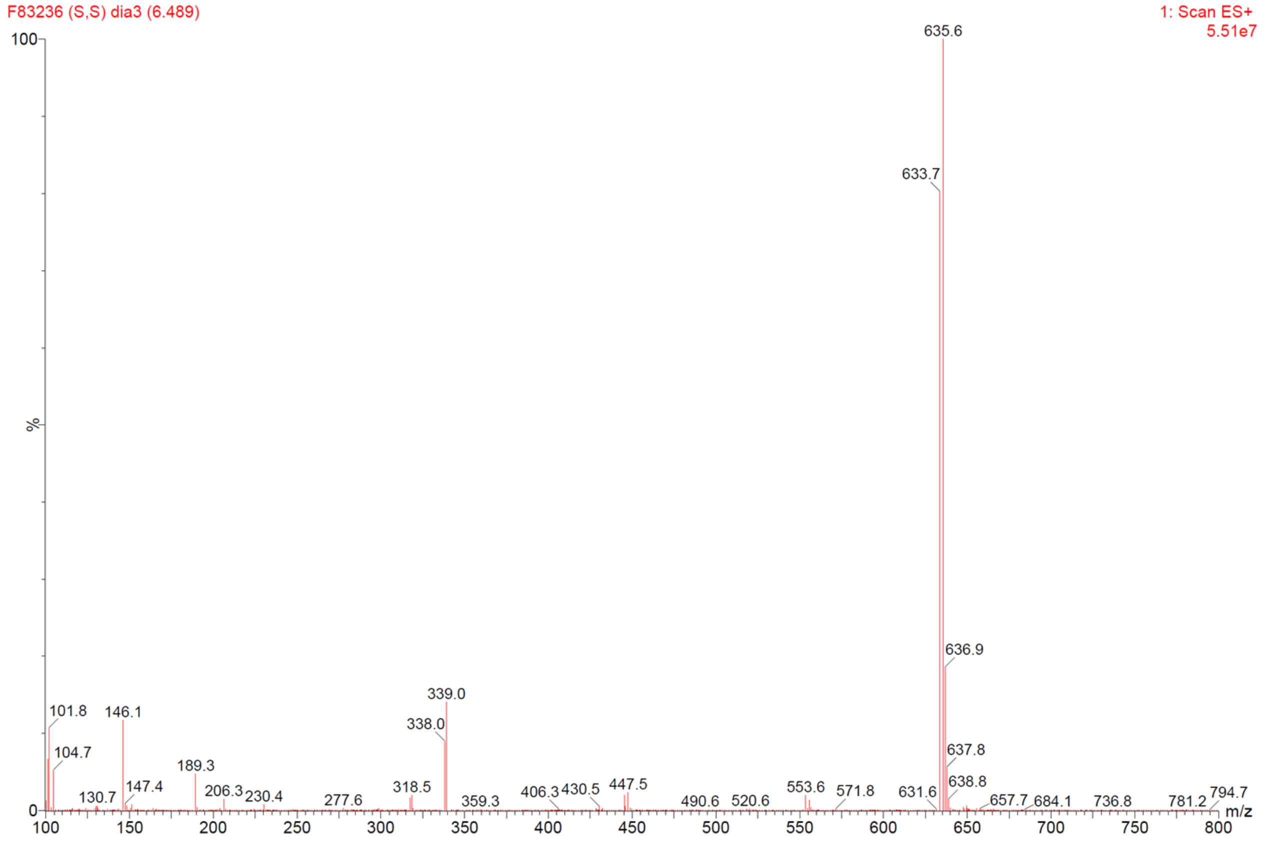

Mass spectrum of compound C105SS.

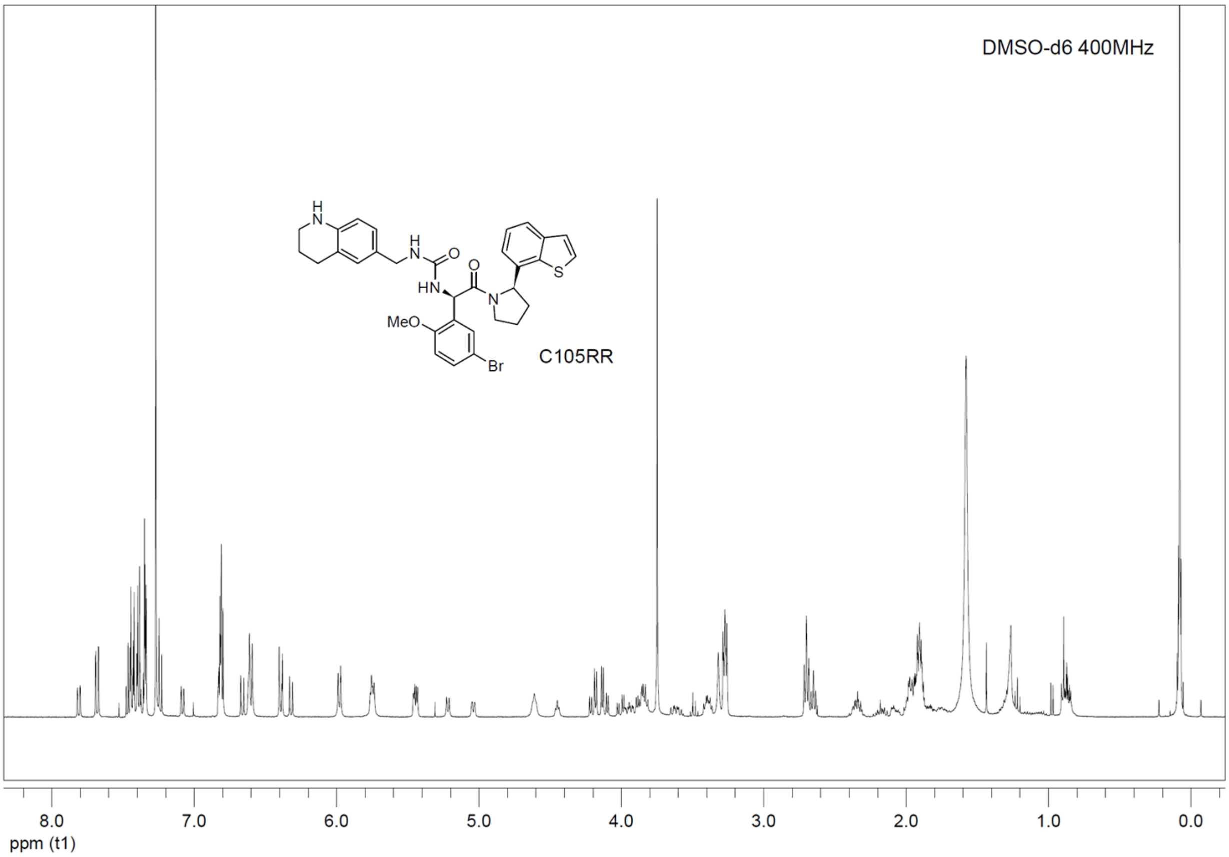

^{1}H NMR (400 MHz, DMSO-d6) spectrum of compound C105RR.

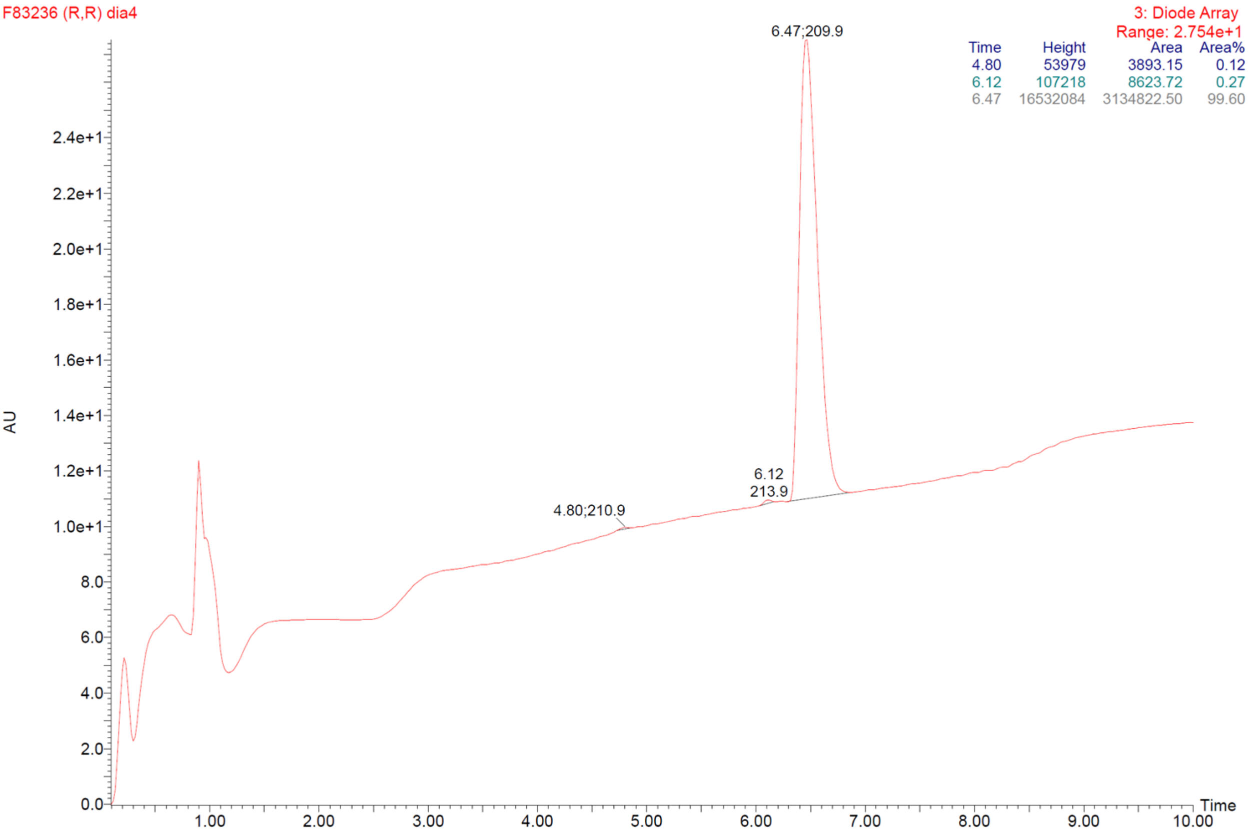

Time	Height	Area	Area%
4.80	53979	3893.15	0.12
6.12	107218	8623.72	0.27
6.47	16532084	3134822.50	99.60

HPLC Chromatogram of compound C105RR.

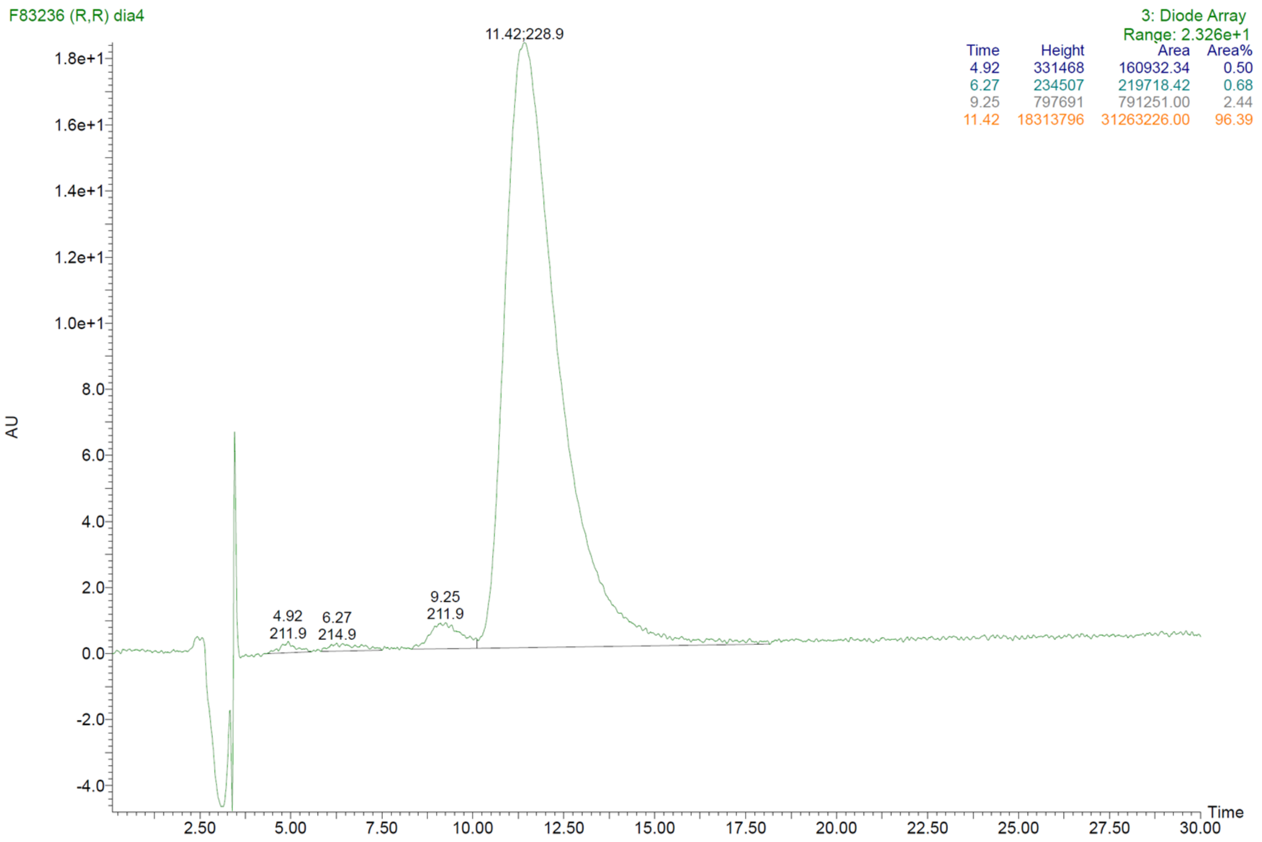

Chiral HPLC Chromatogram of compound C105RR.

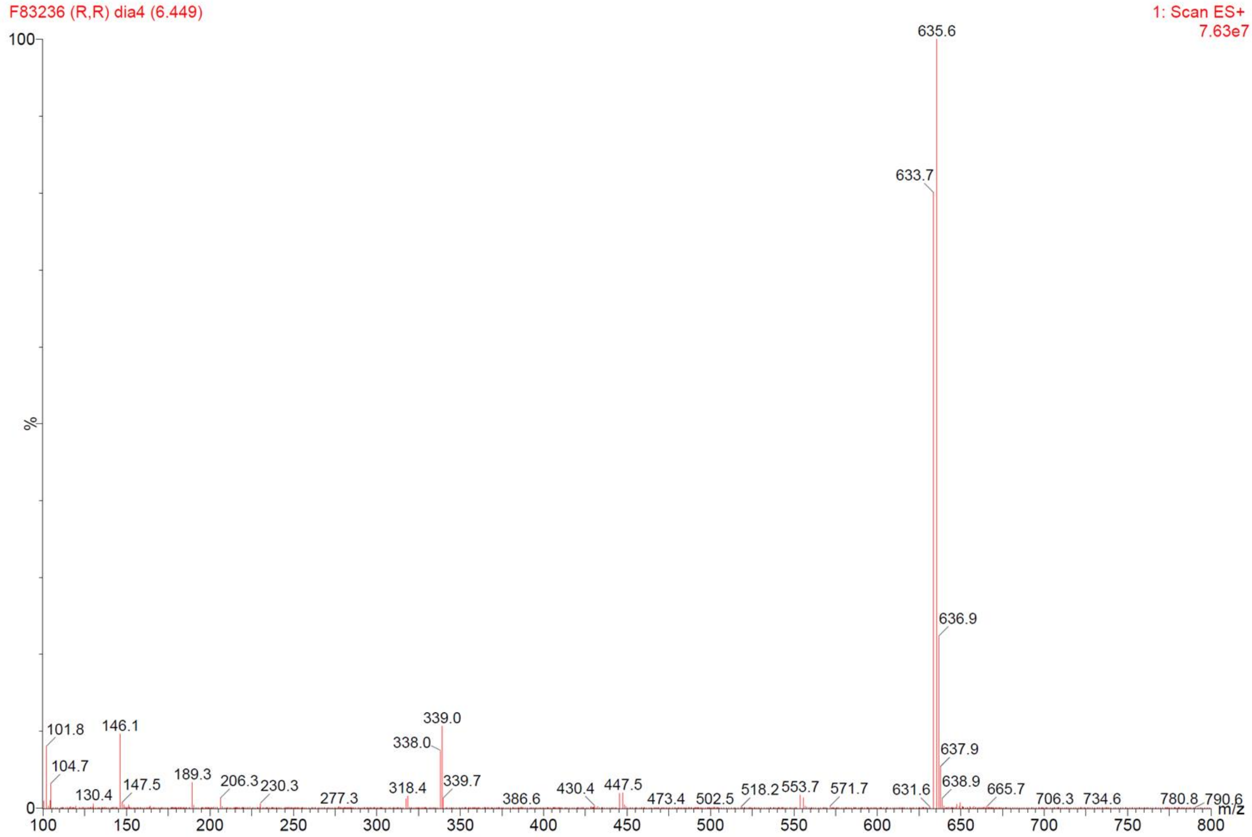

Mass spectrum of compound C105RR.

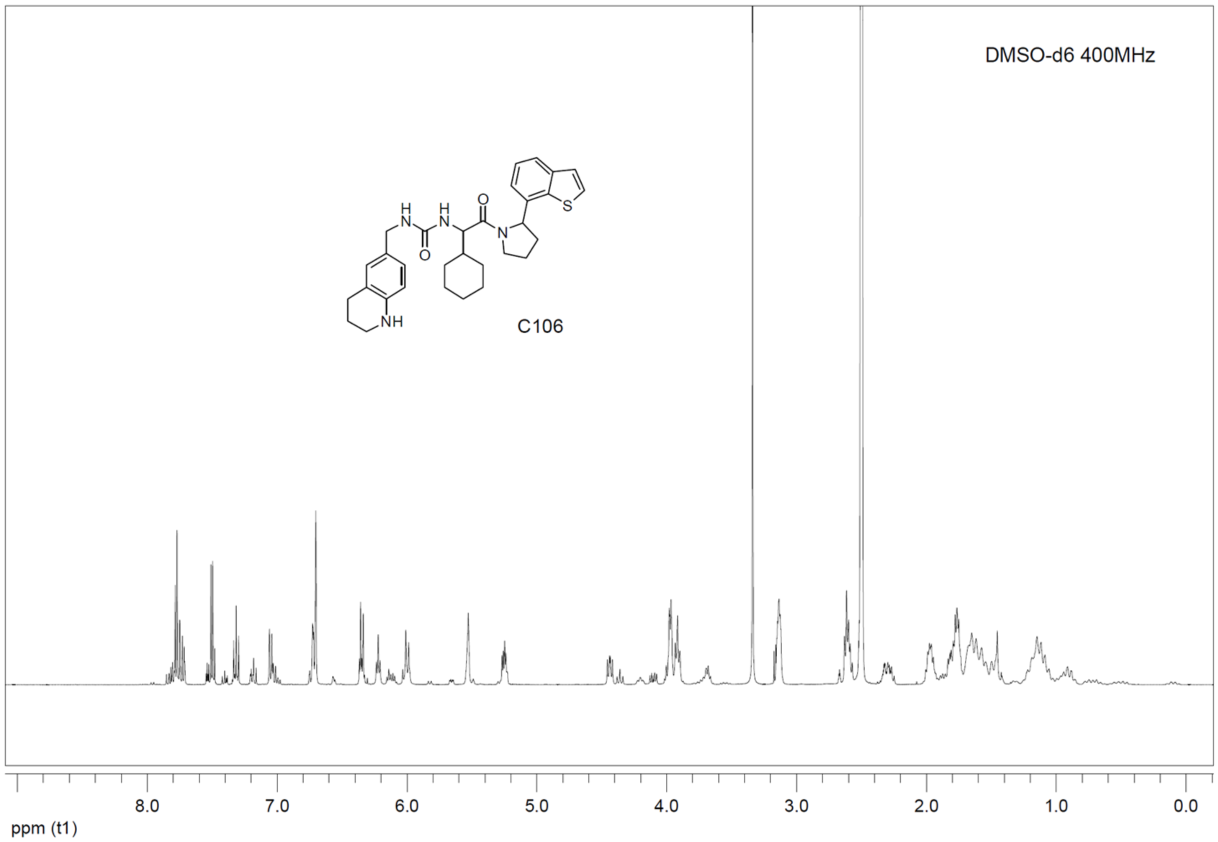

^{1}H NMR (400 MHz, DMSO-d6) spectrum of compound C106.

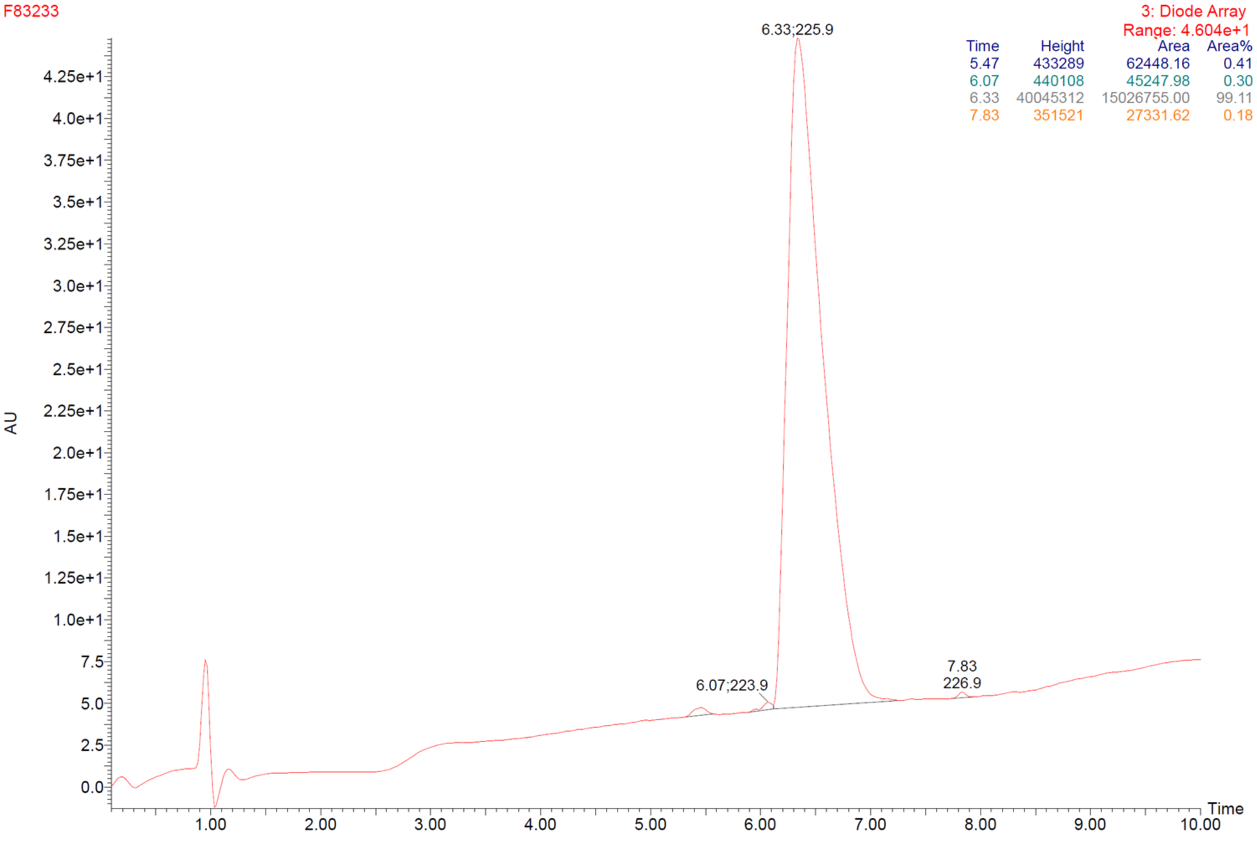

HPLC Chromatogram of compound C106.

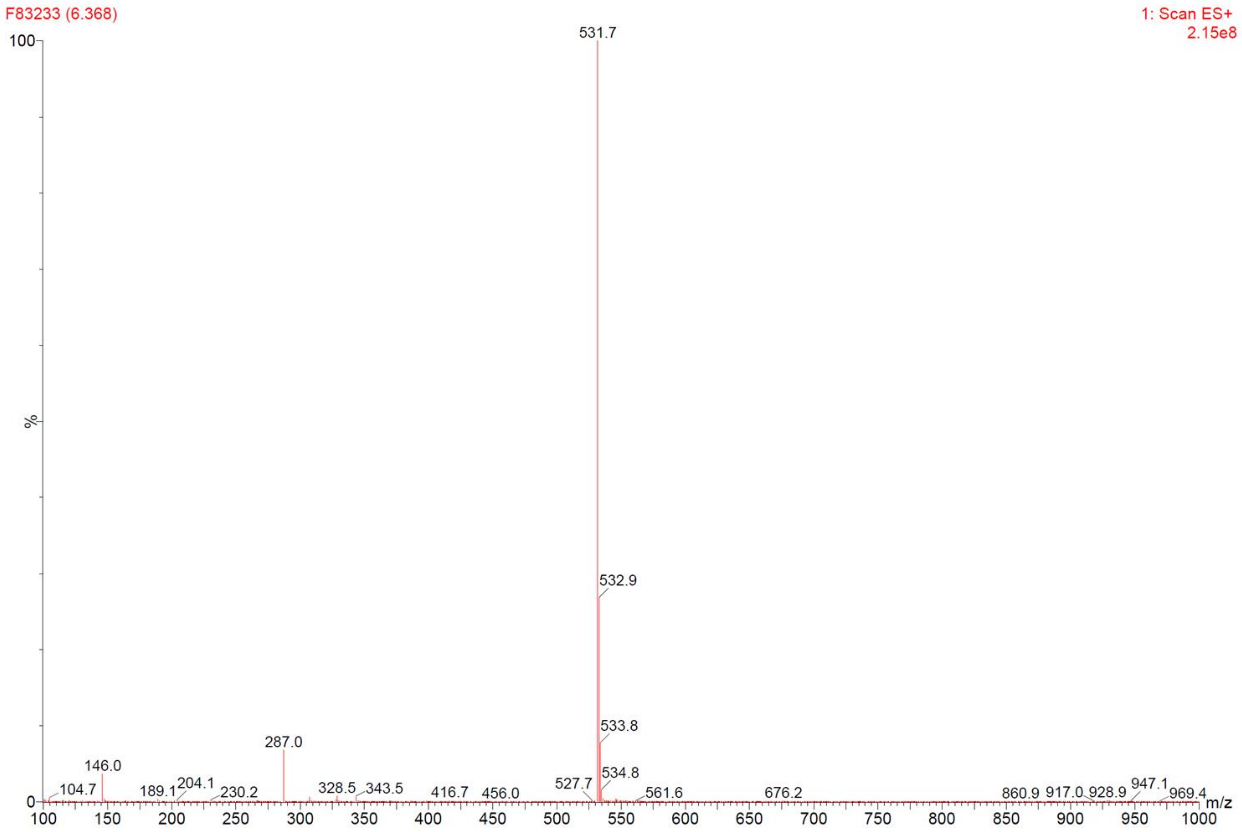

Mass spectrum of compound C106.

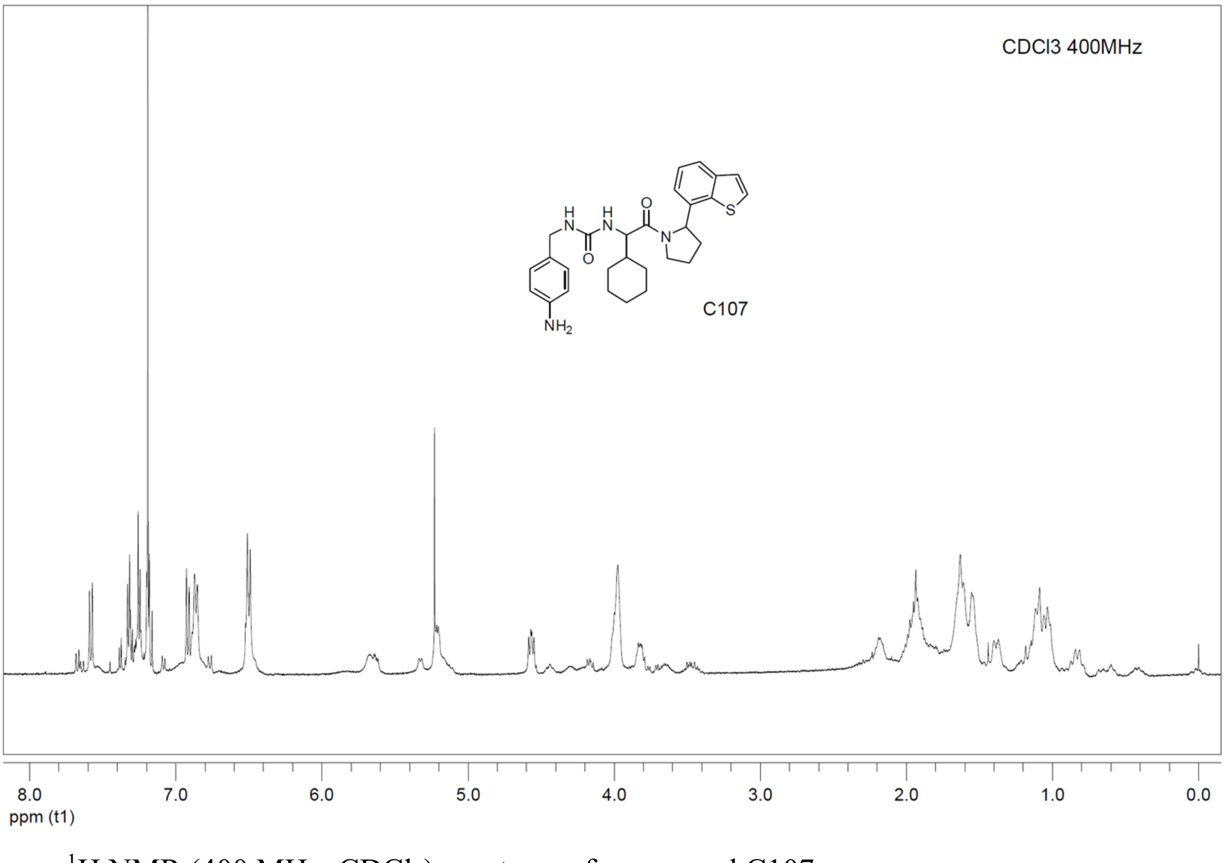

^{1}H NMR (400 MHz, $CDCl_3$) spectrum of compound C107.

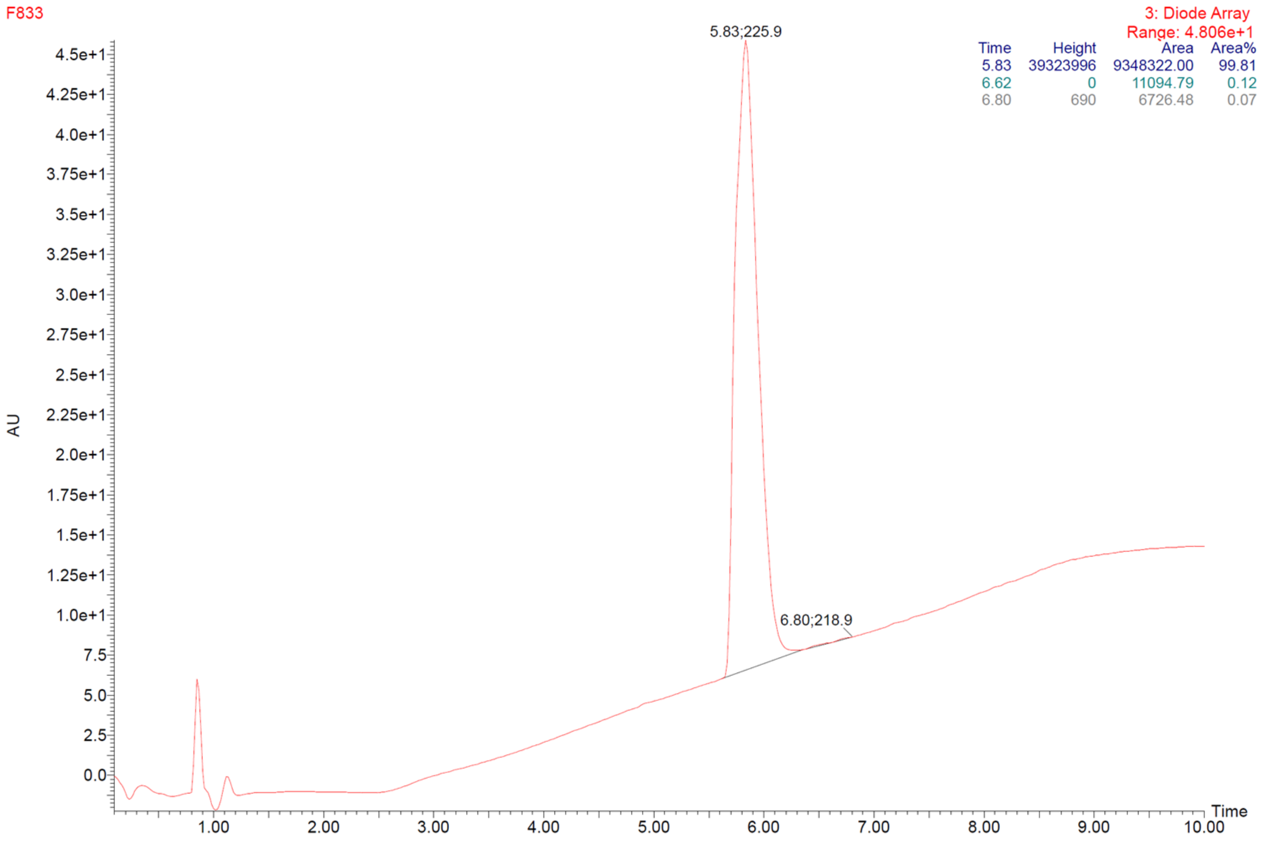

HPLC Chromatogram of compound C107.

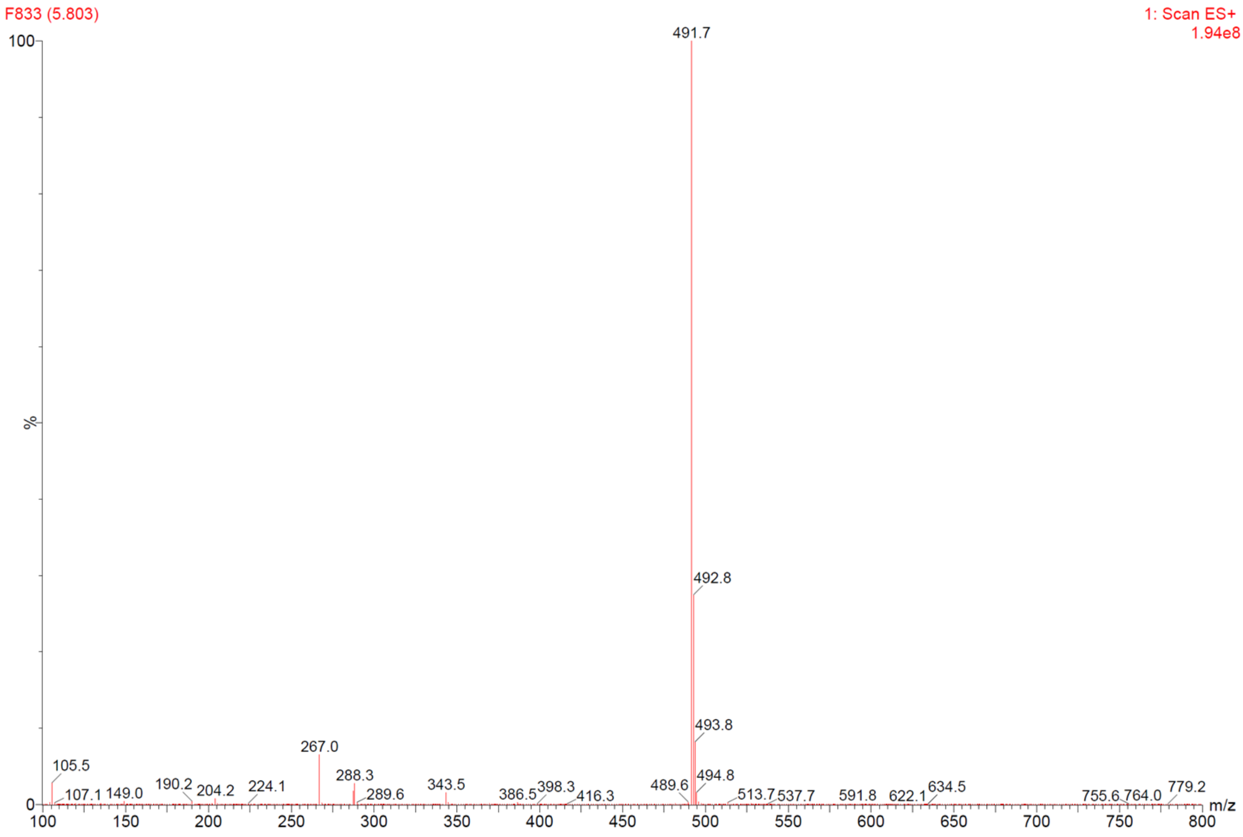

Mass spectrum of compound C107.

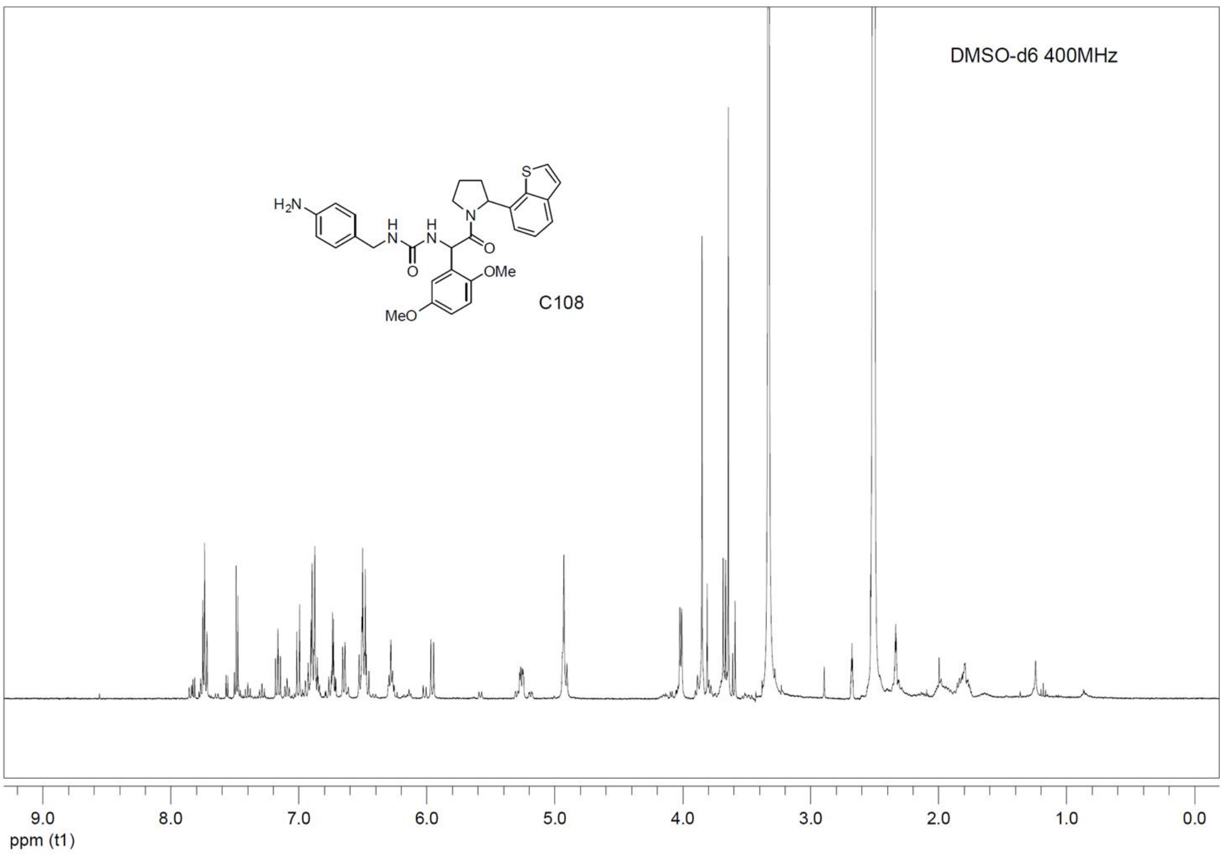

^{1}H NMR (400 MHz, DMSO-d6) spectrum of compound C108.

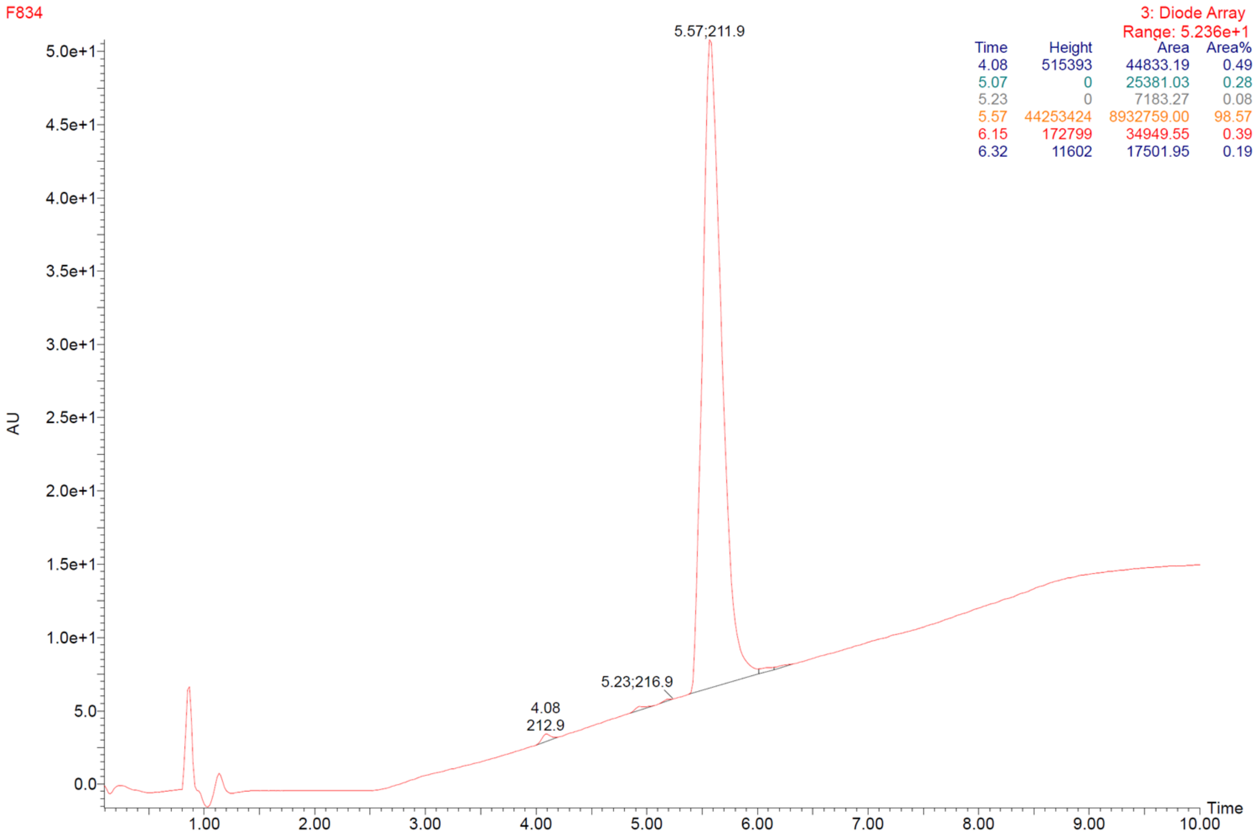

HPLC Chromatogram of compound C108.

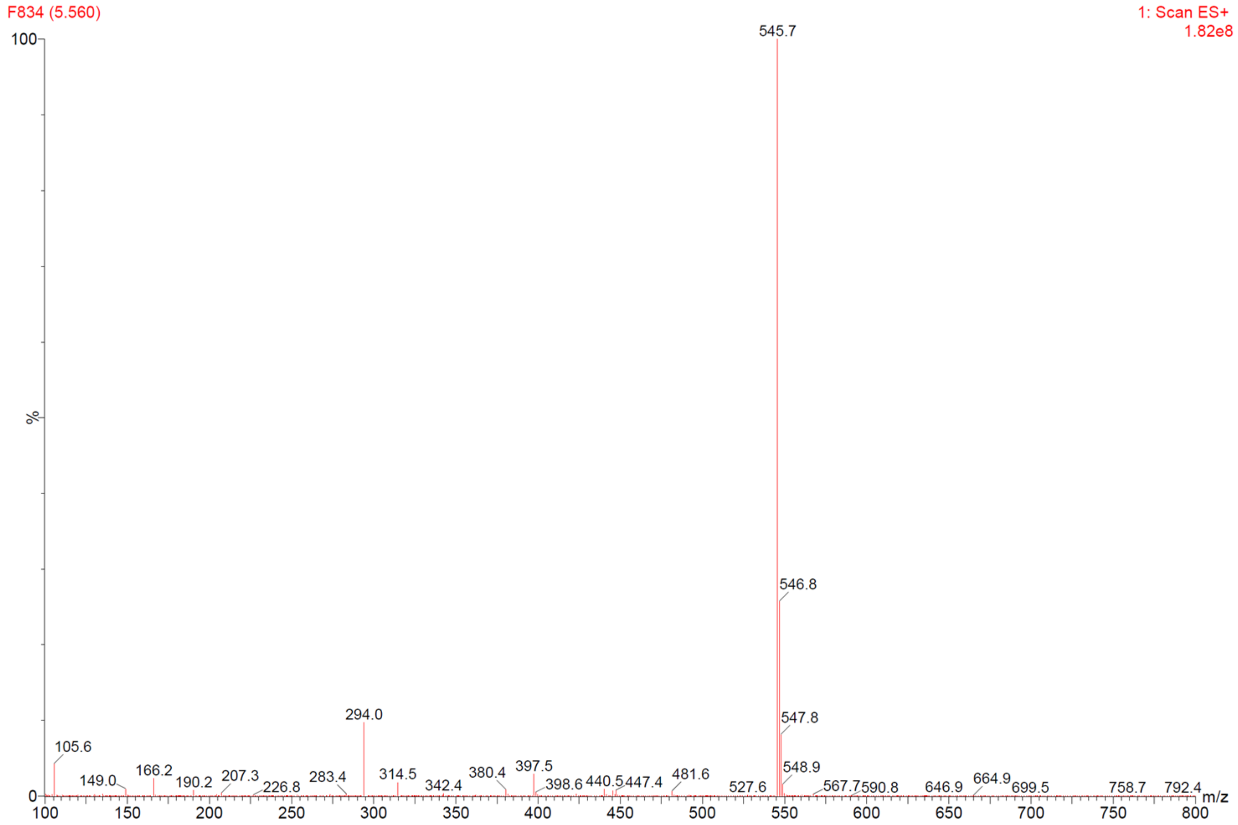

Mass spectrum of compound C108.

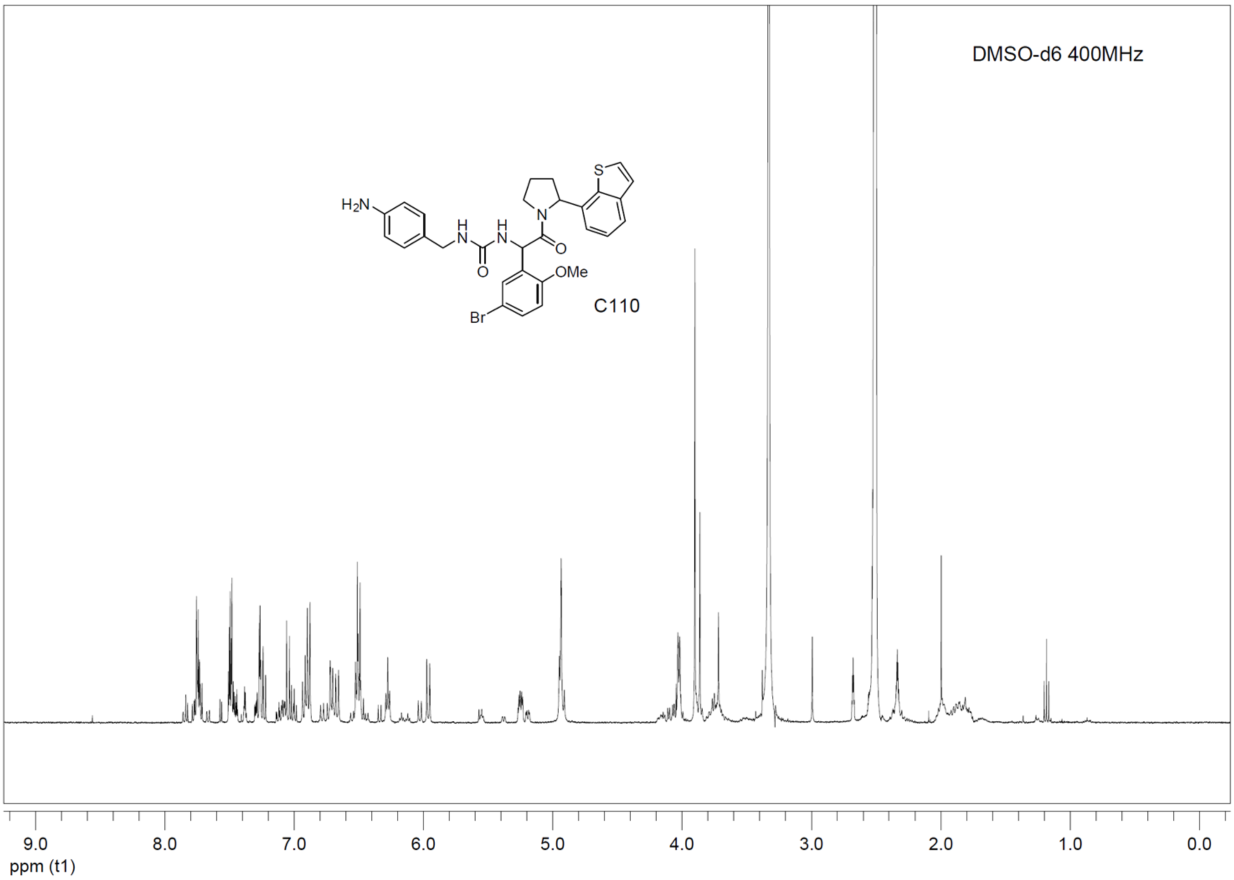

^{1}H NMR (400 MHz, DMSO-d6) spectrum of compound C110.

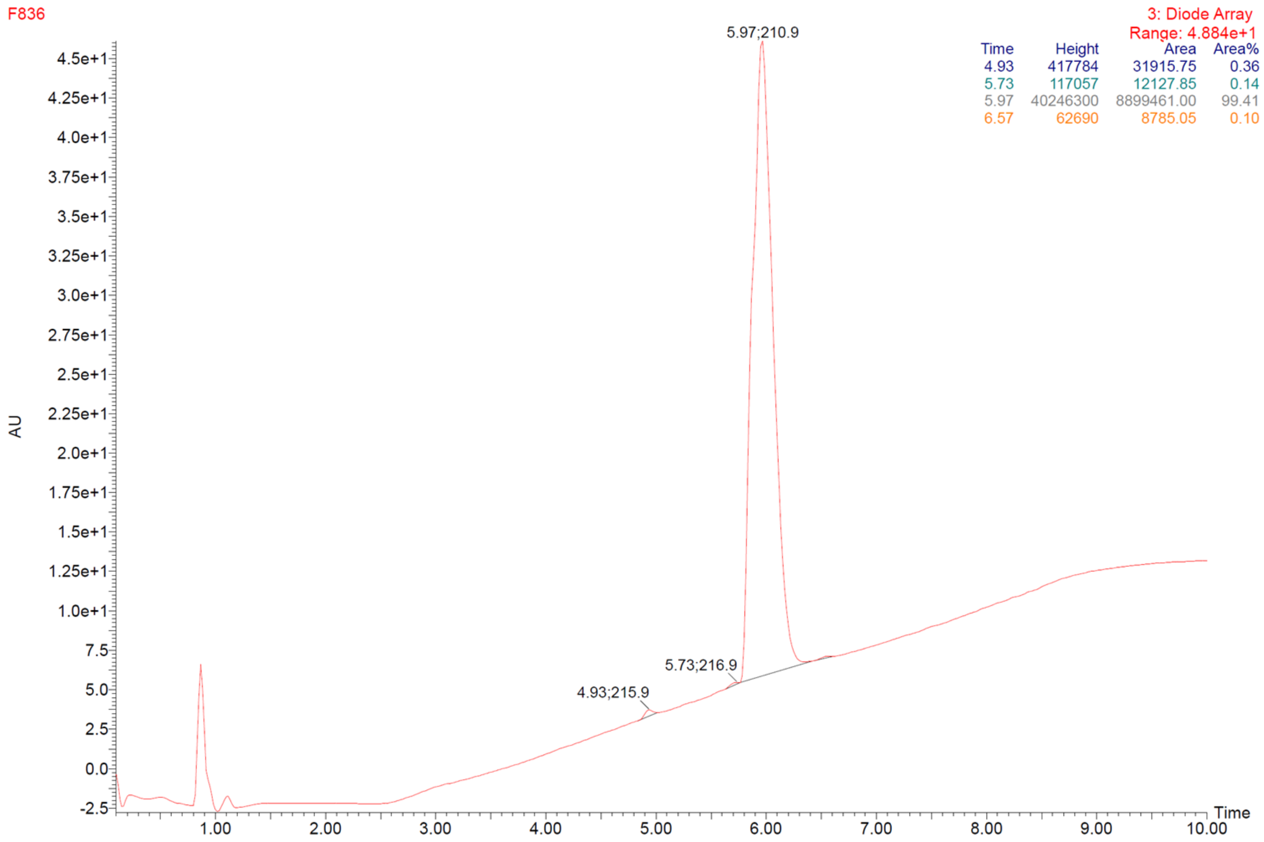

HPLC Chromatogram of compound C110.

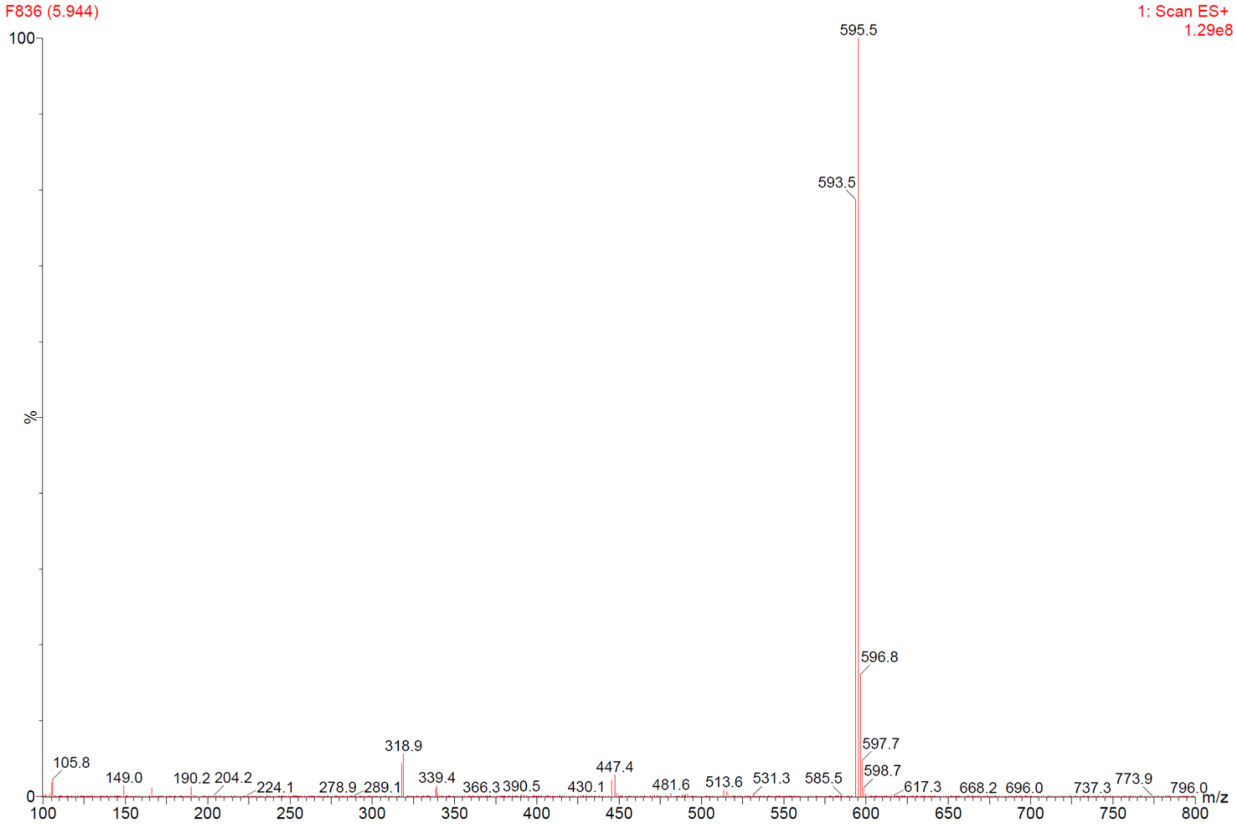

Mass spectrum of compound C110.

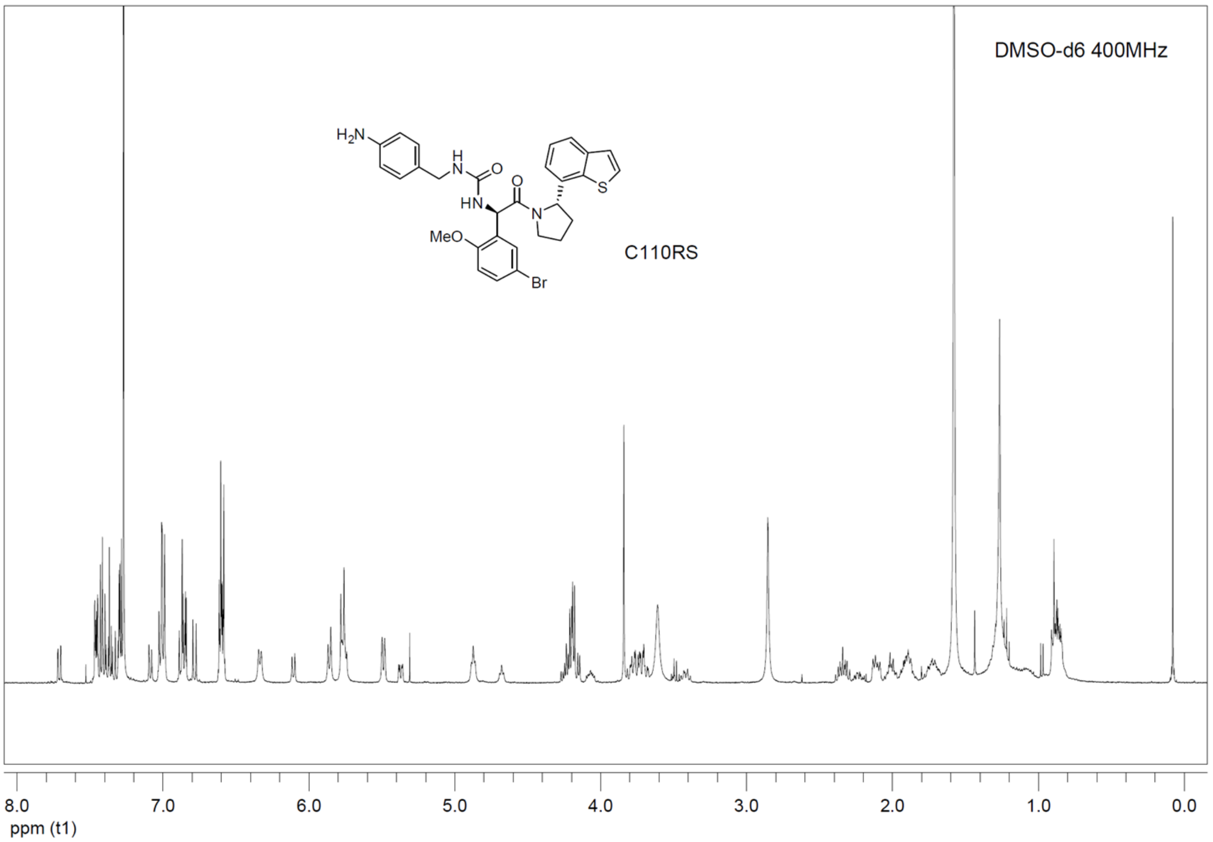

^{1}H NMR (400 MHz, DMSO-d6) spectrum of compound C110RS

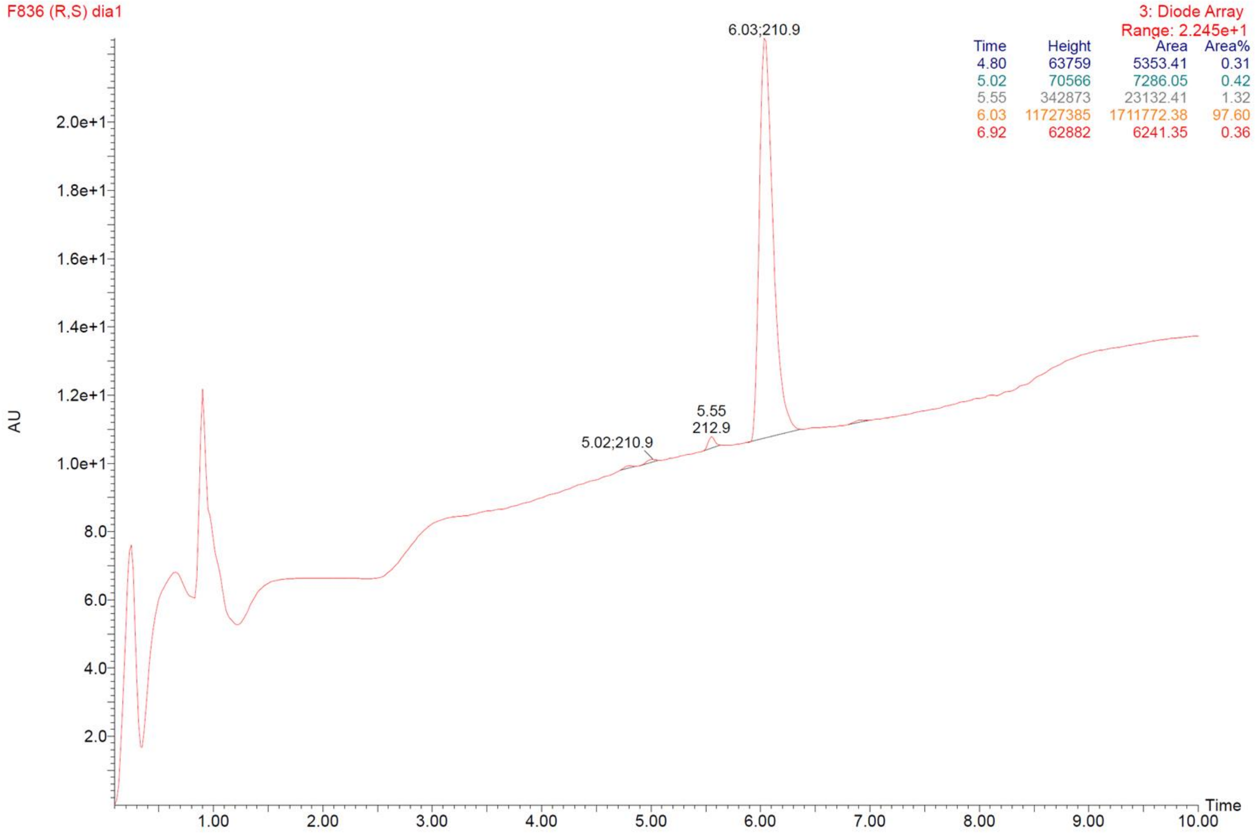

HPLC Chromatogram of compound C110RS.

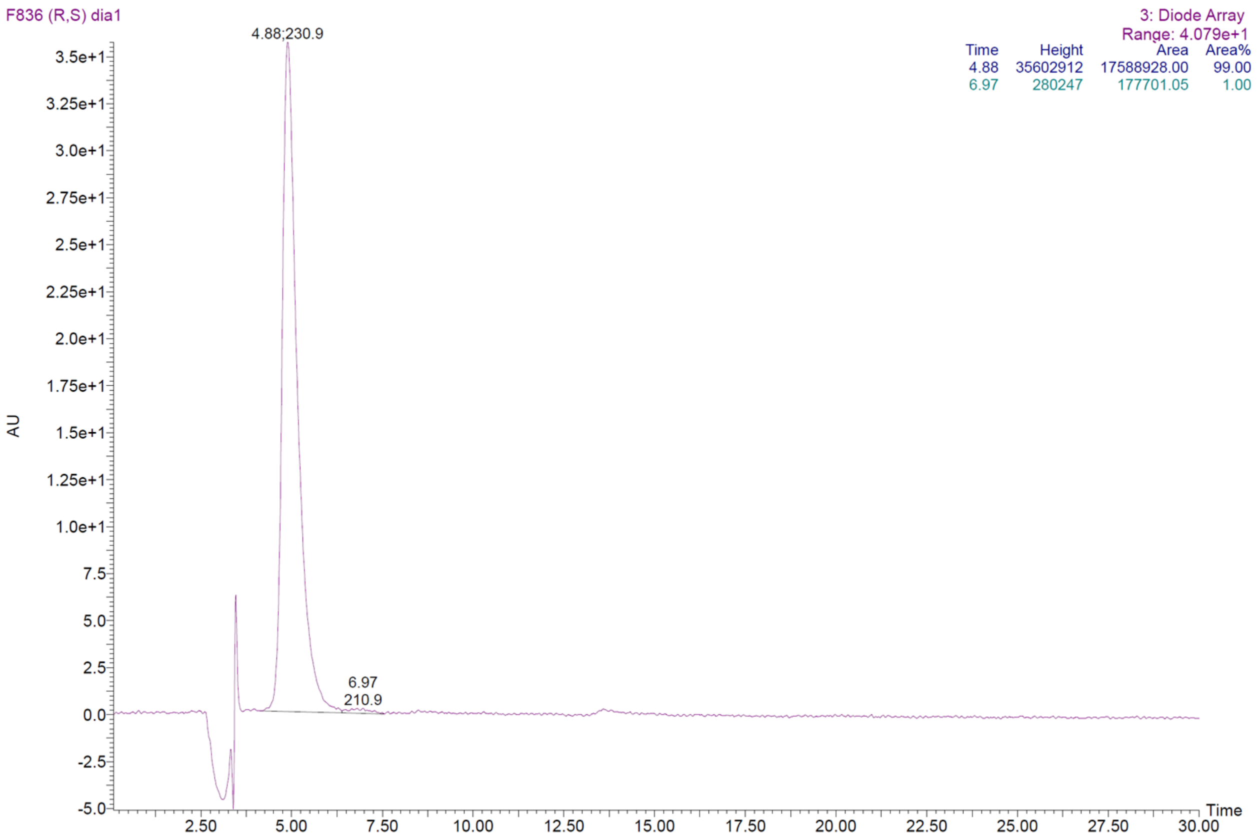

Chiral HPLC Chromatogram of compound C110RS.

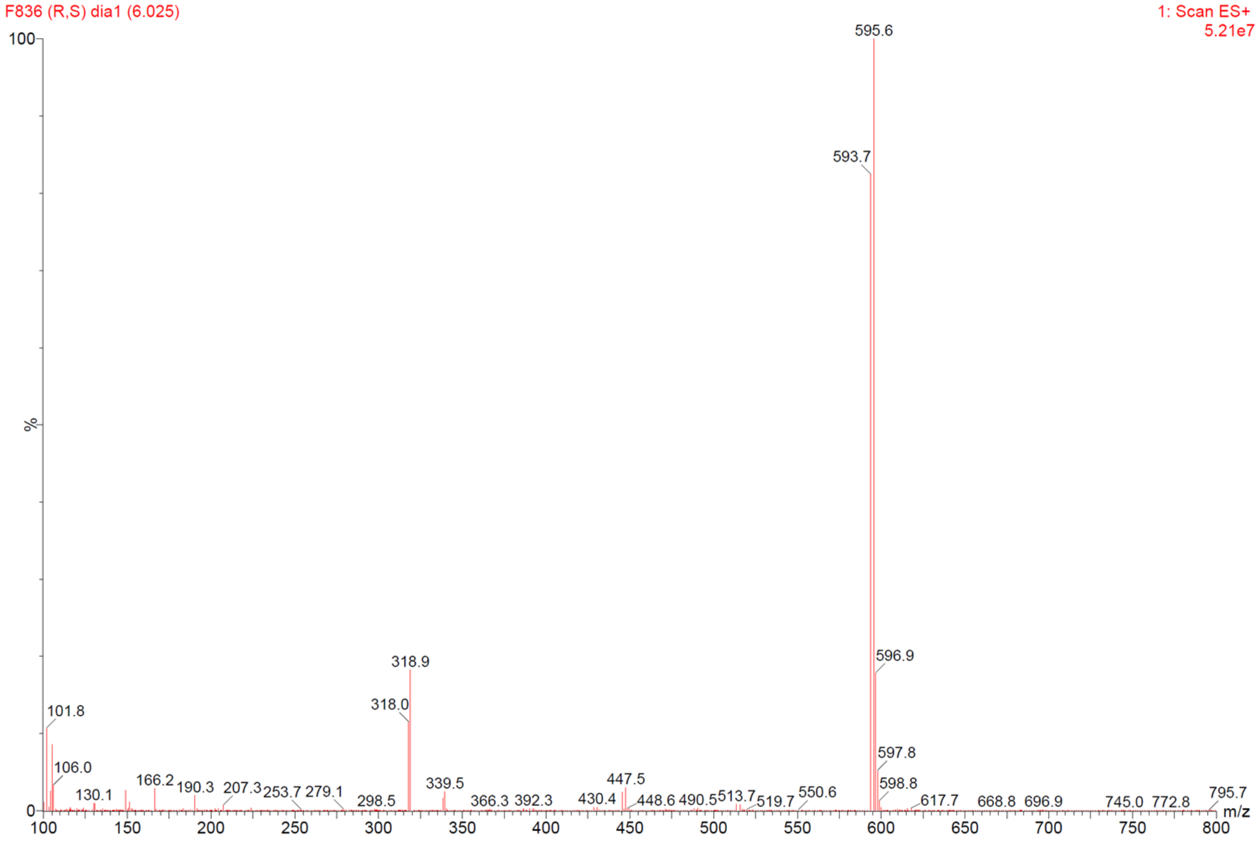

Mass spectrum of compound C110RS.

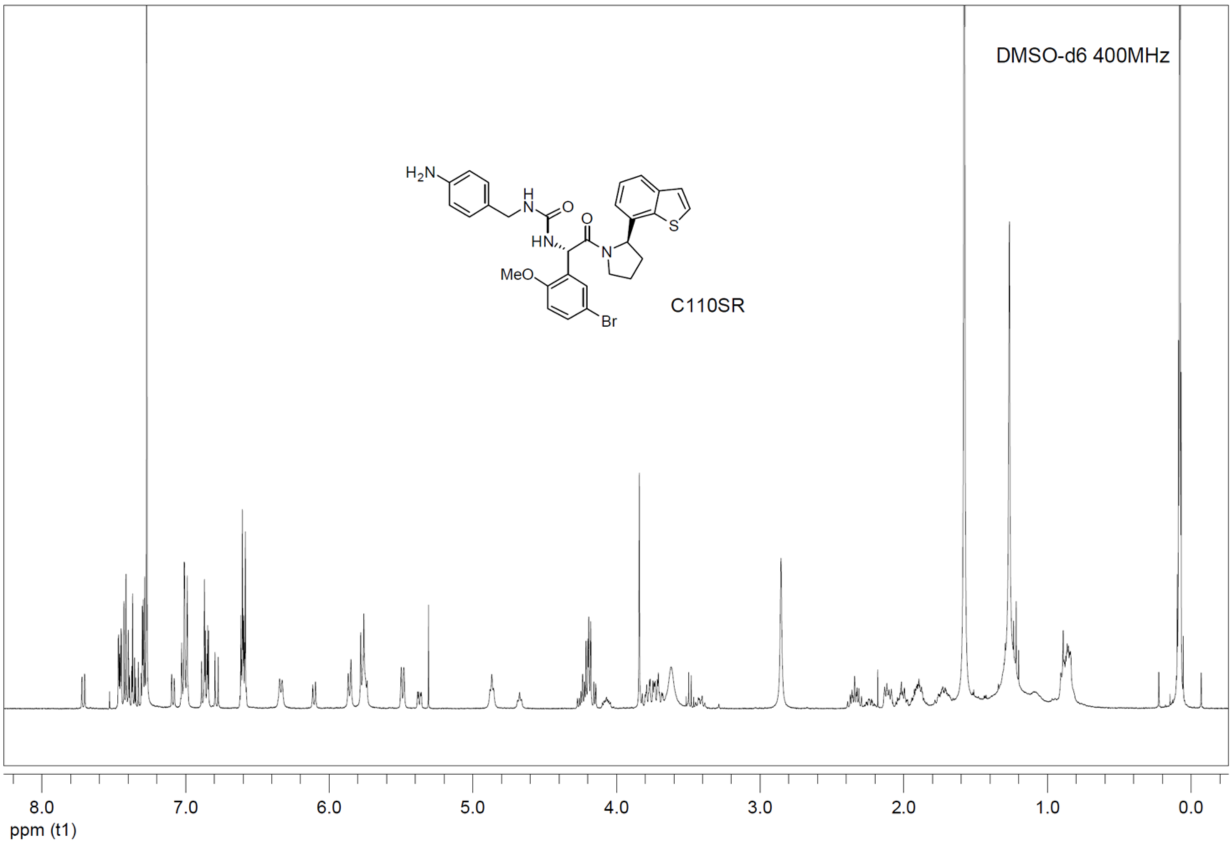

^{1}H NMR (400 MHz, DMSO-d6) spectrum of compound C110SR.

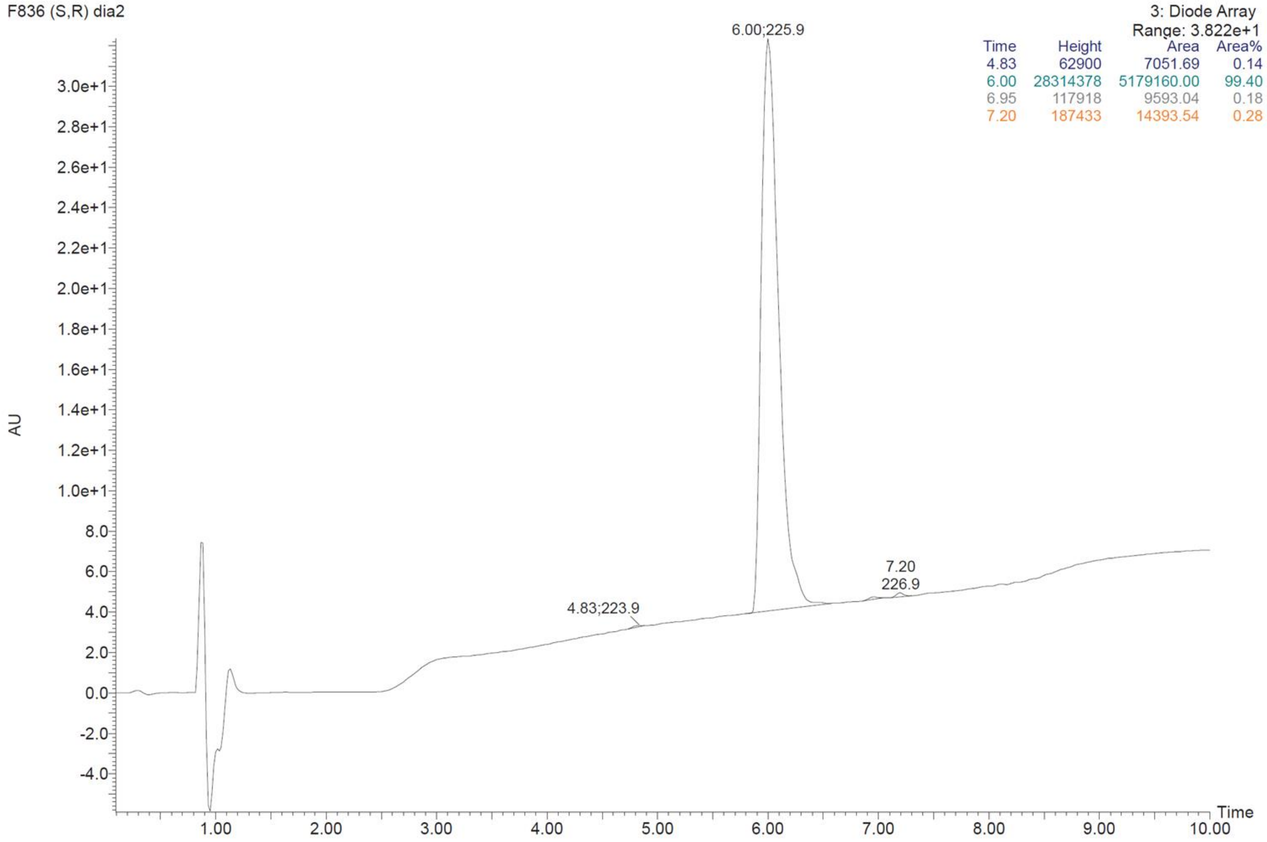

HPLC Chromatogram of compound C110SR.

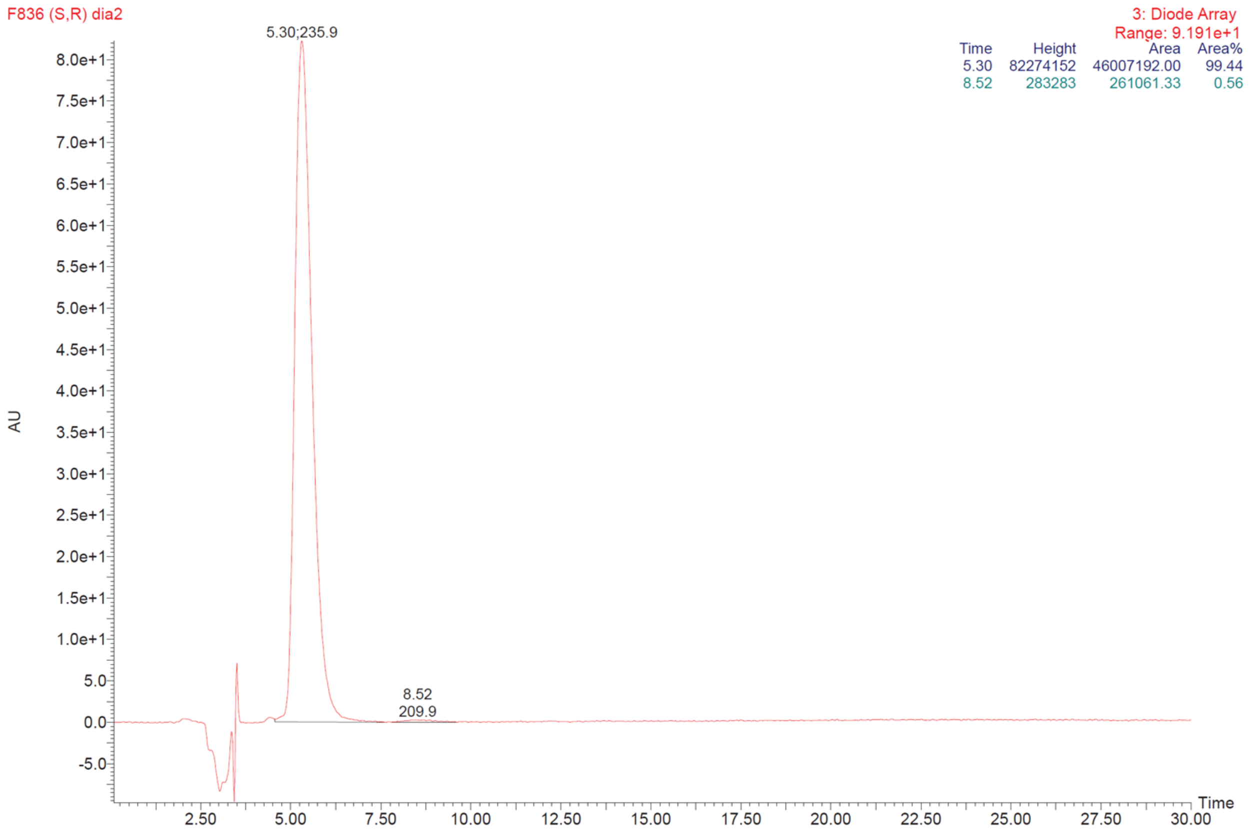

Chiral HPLC Chromatogram of compound C110SR.

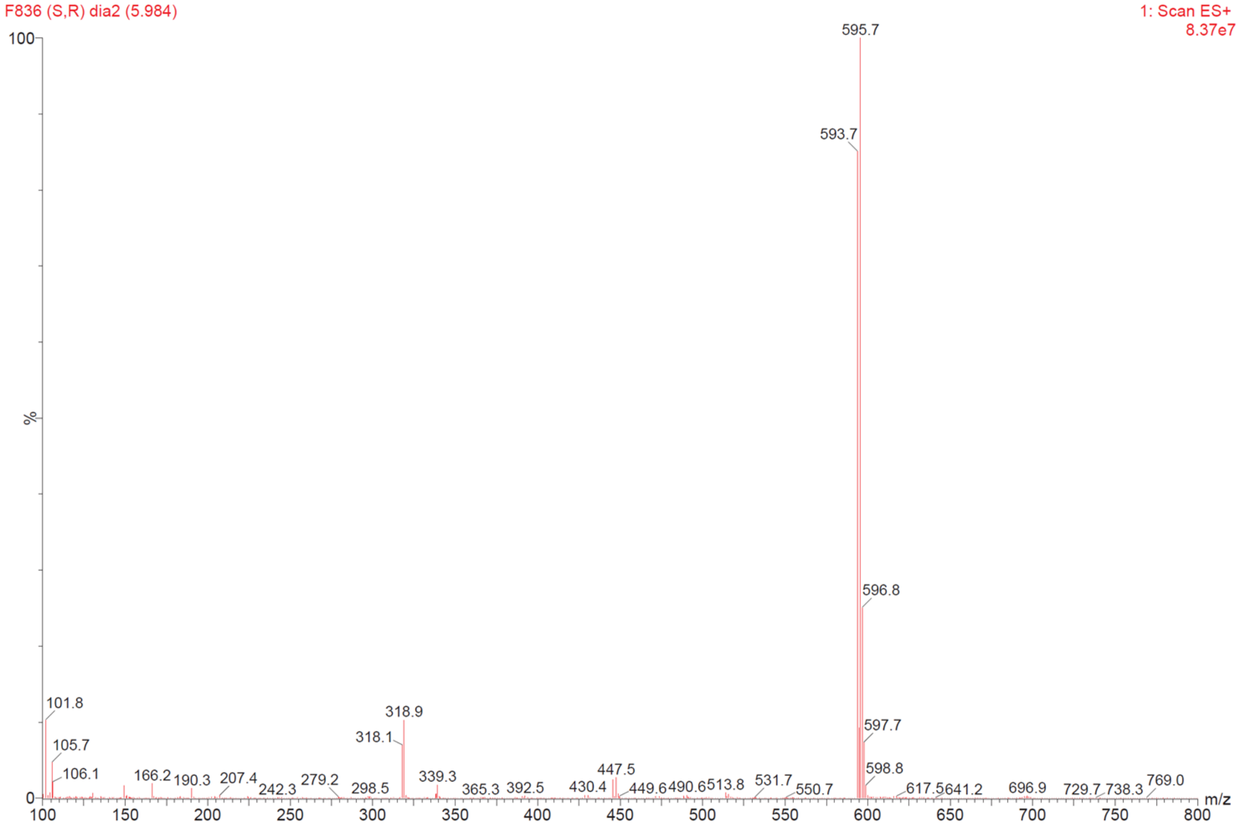

Mass spectrum of compound C110SR.

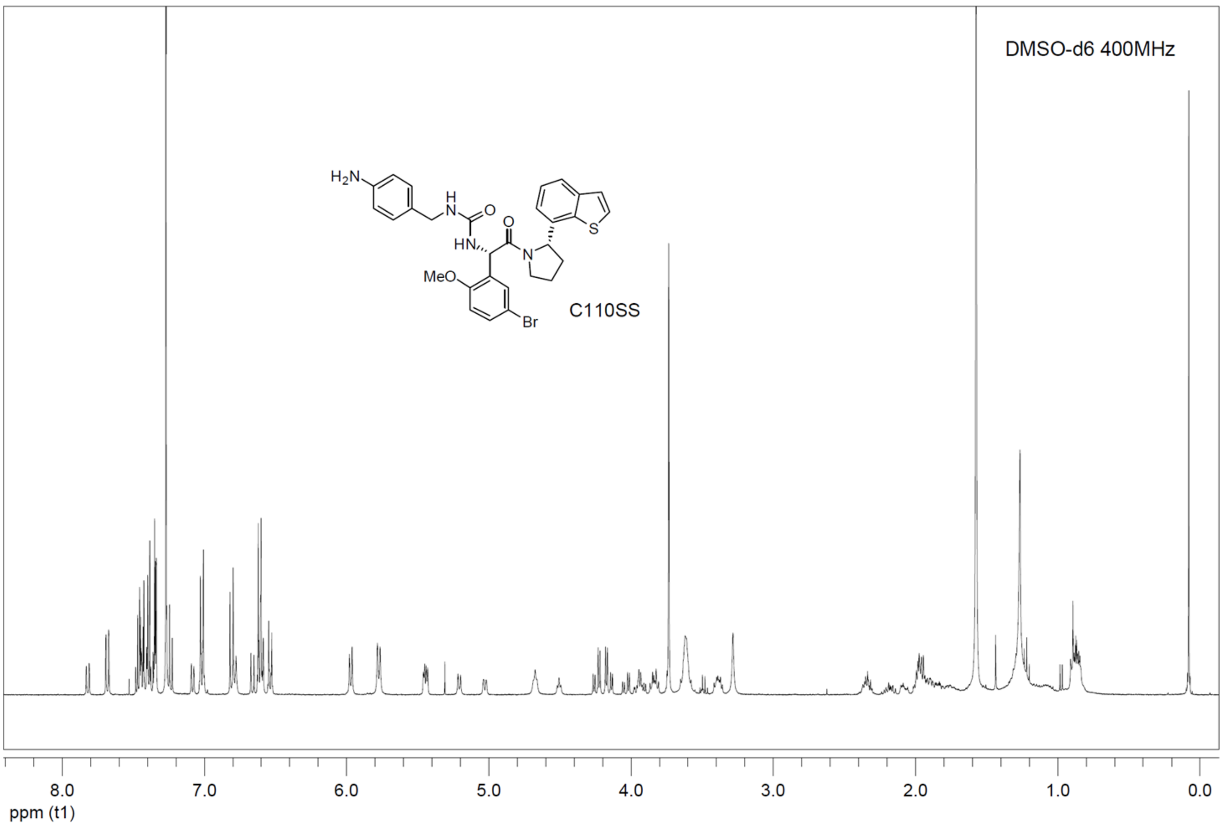

^{1}H NMR (400 MHz, DMSO-d6) spectrum of compound C110SS.

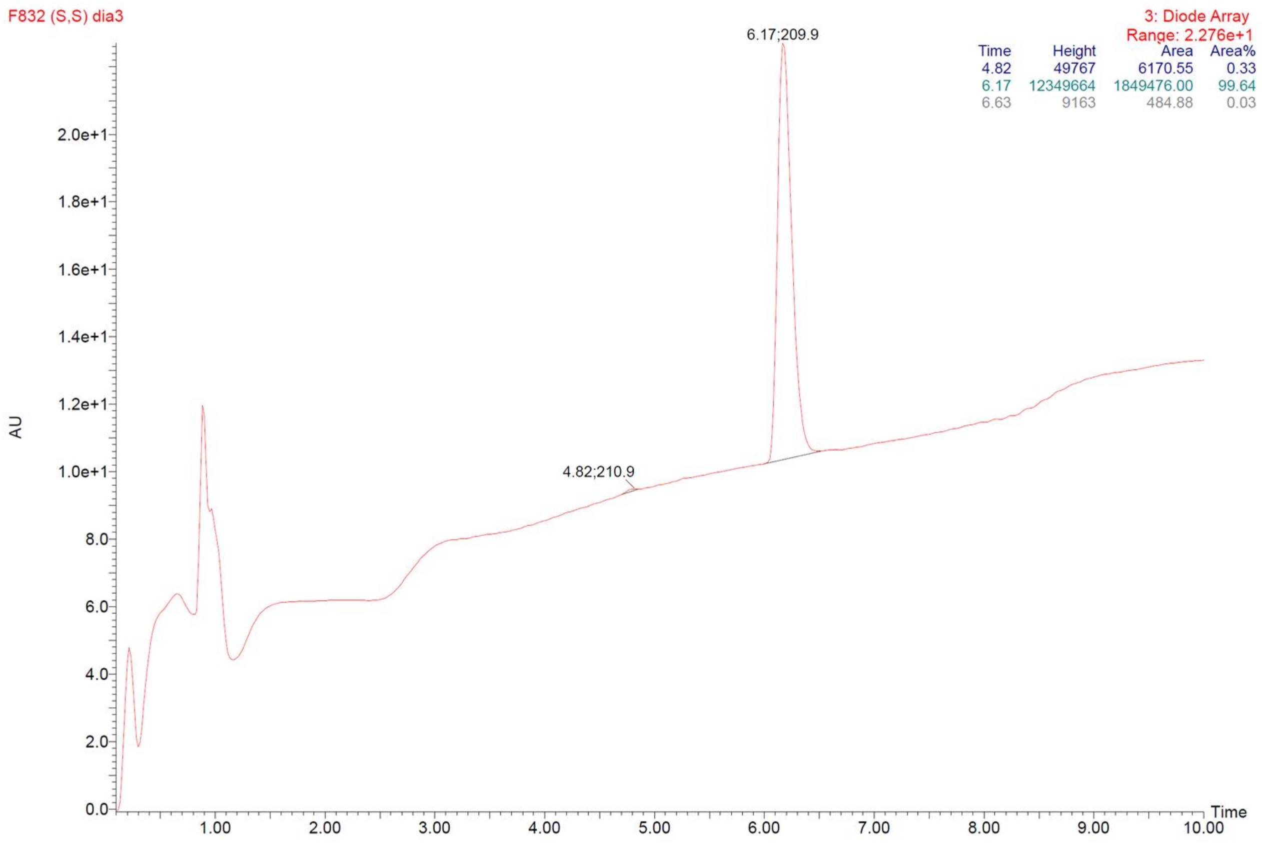

HPLC Chromatogram of compound C110SS.

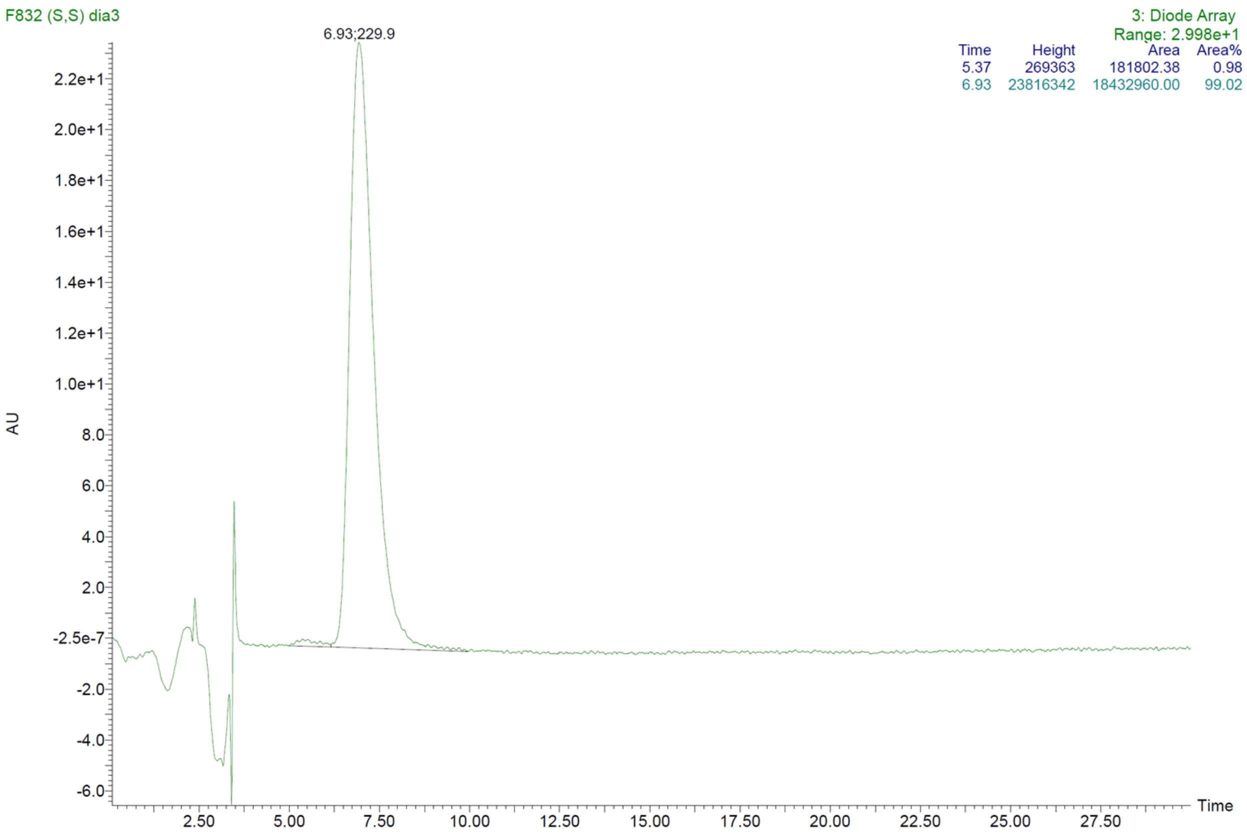

Chiral HPLC Chromatogram of compound C110SS.

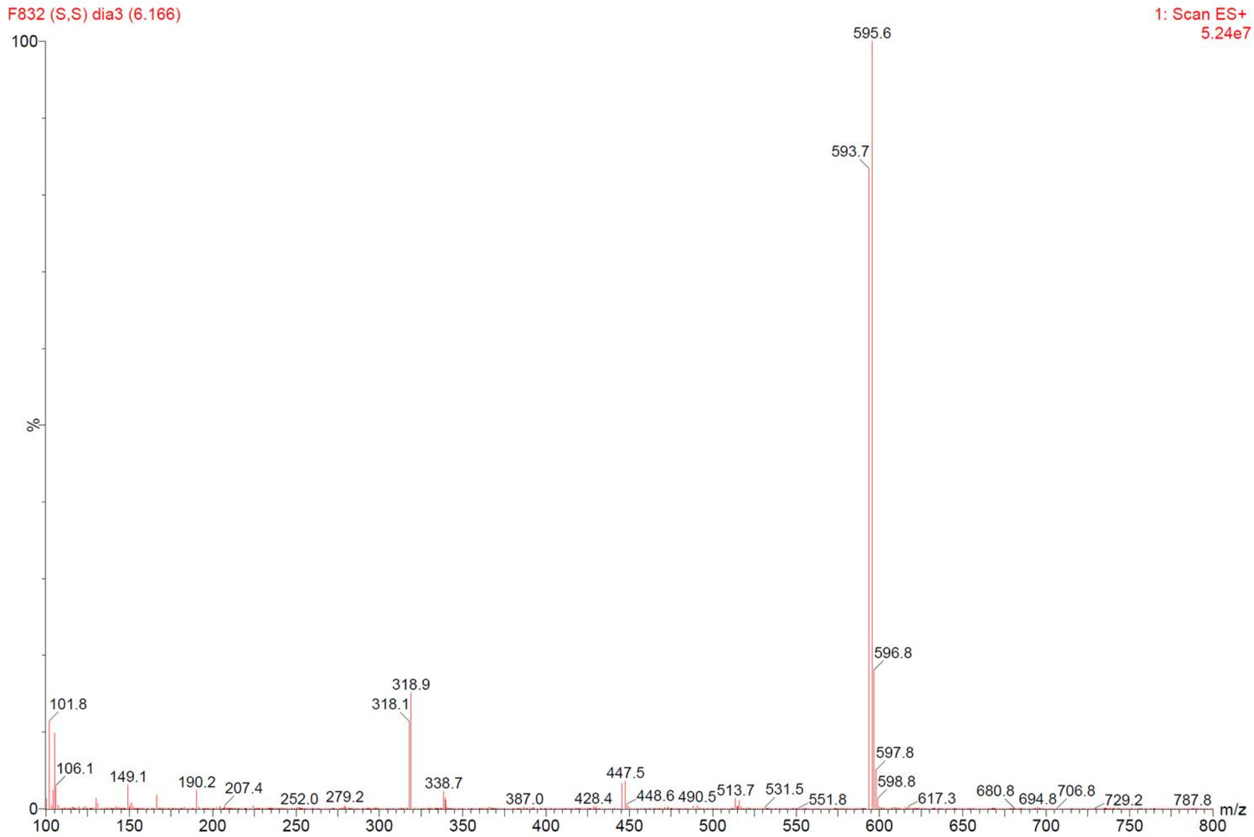

Mass spectrum of compound C110SS.

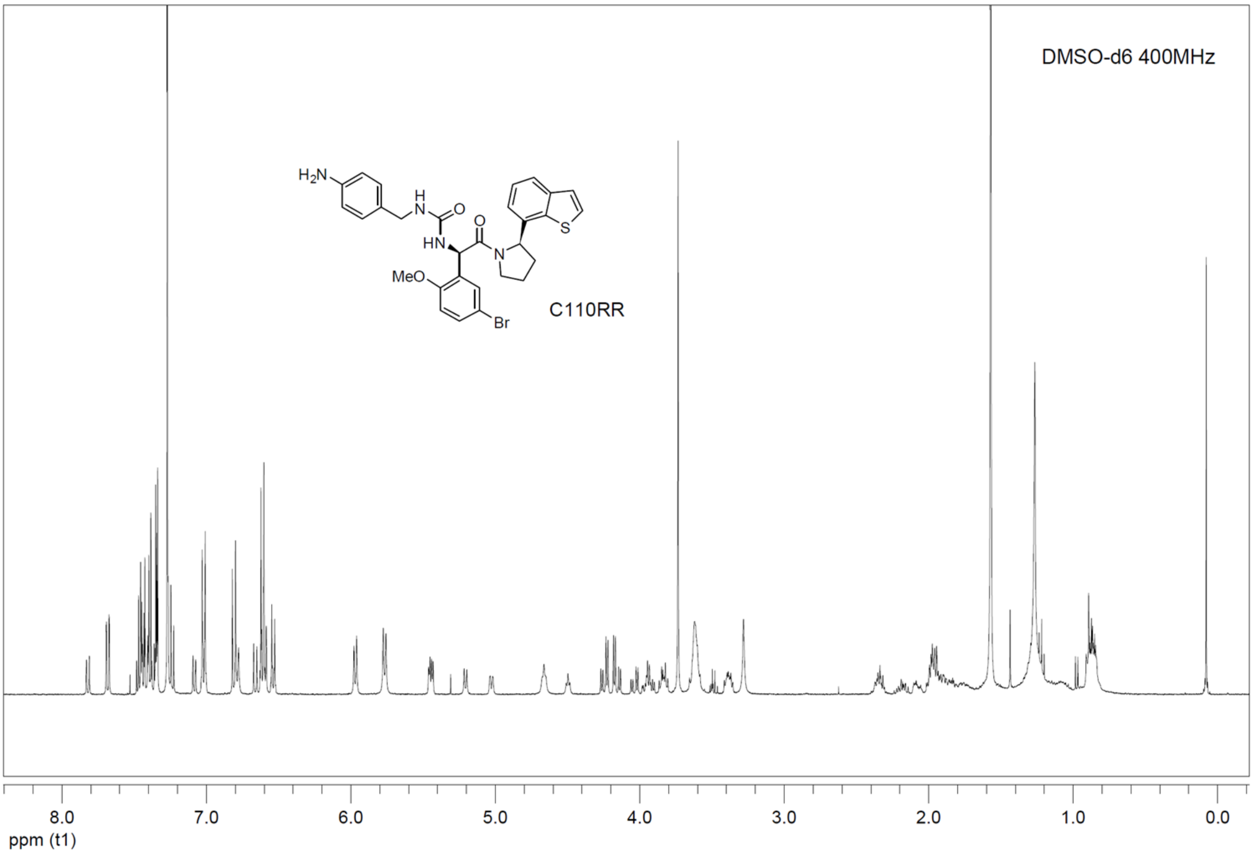

^{1}H NMR (400 MHz, DMSO-d6) spectrum of compound C110RR.

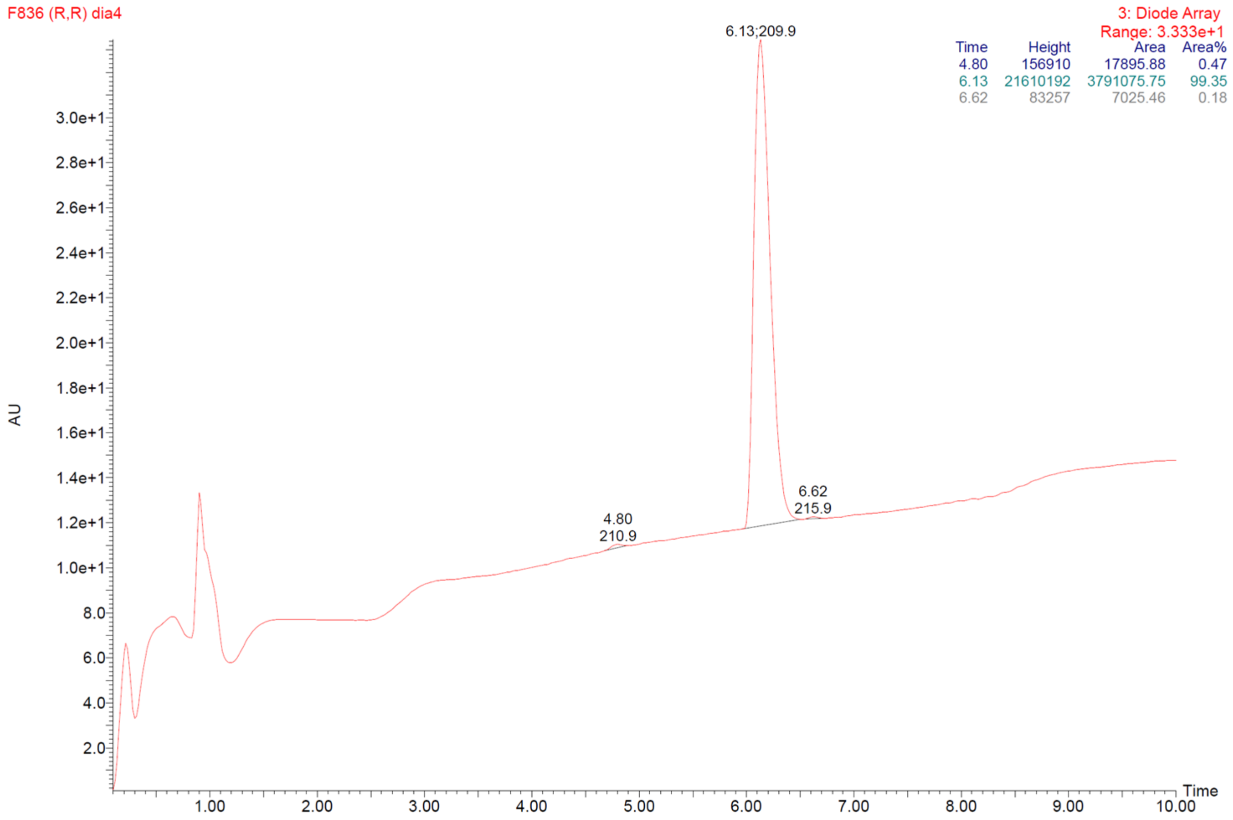

HPLC Chromatogram of compound C110RR.

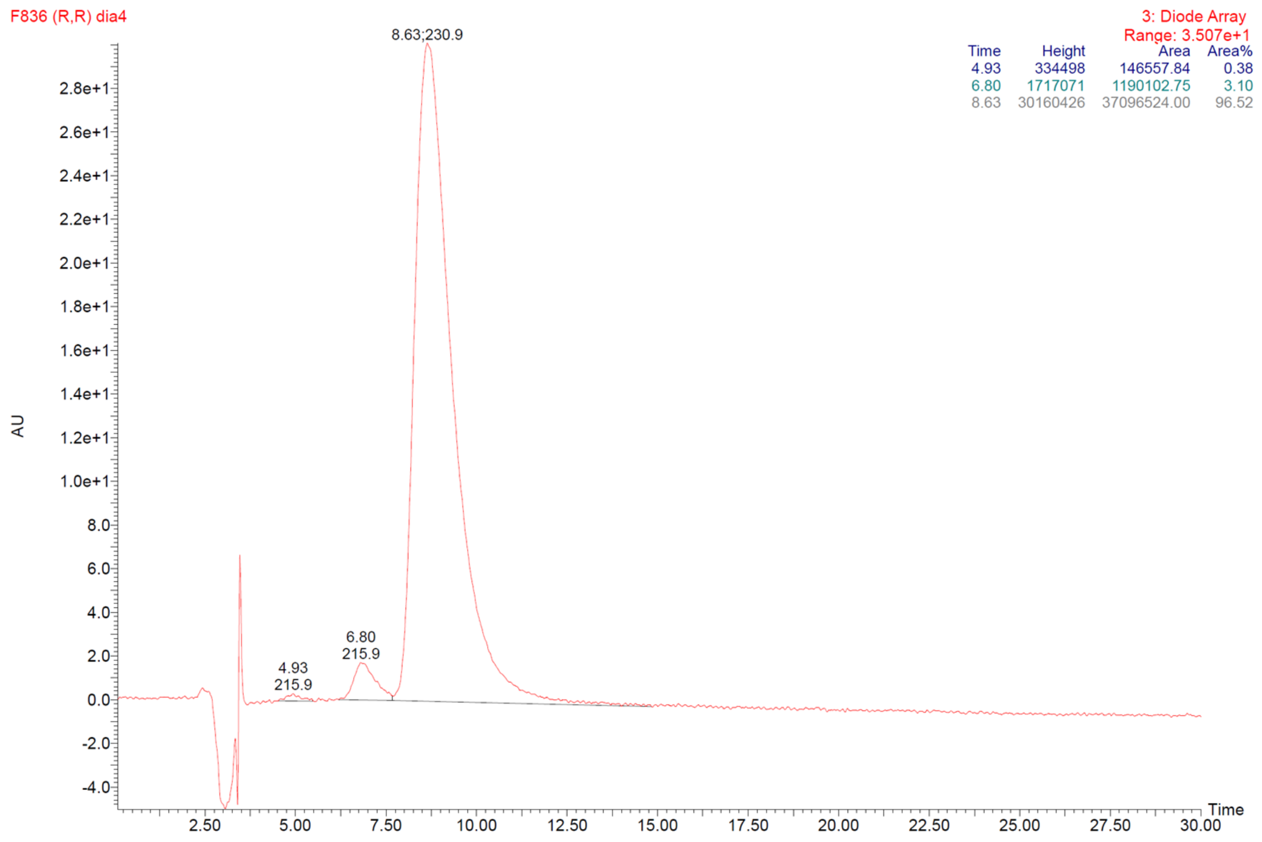

Chiral HPLC Chromatogram of compound C110RR.

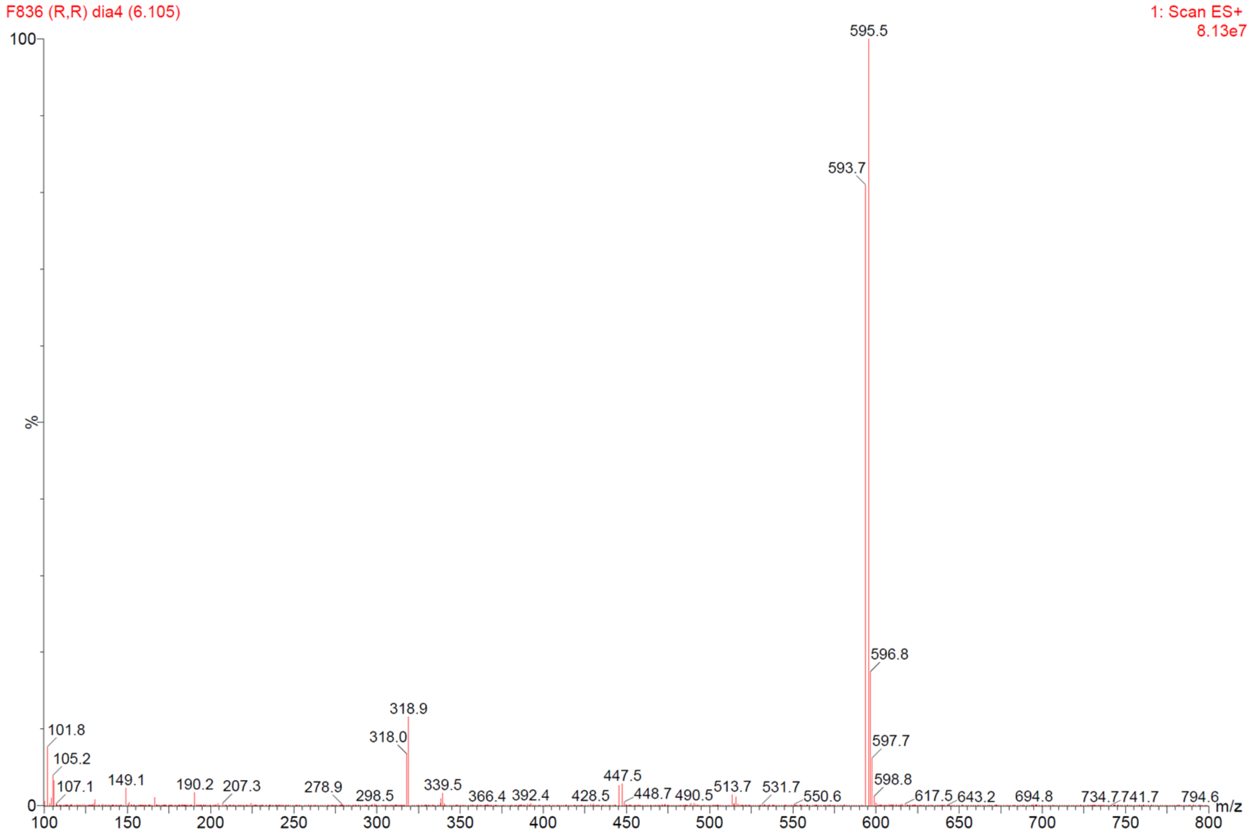

Mass spectrum of compound C110RR.

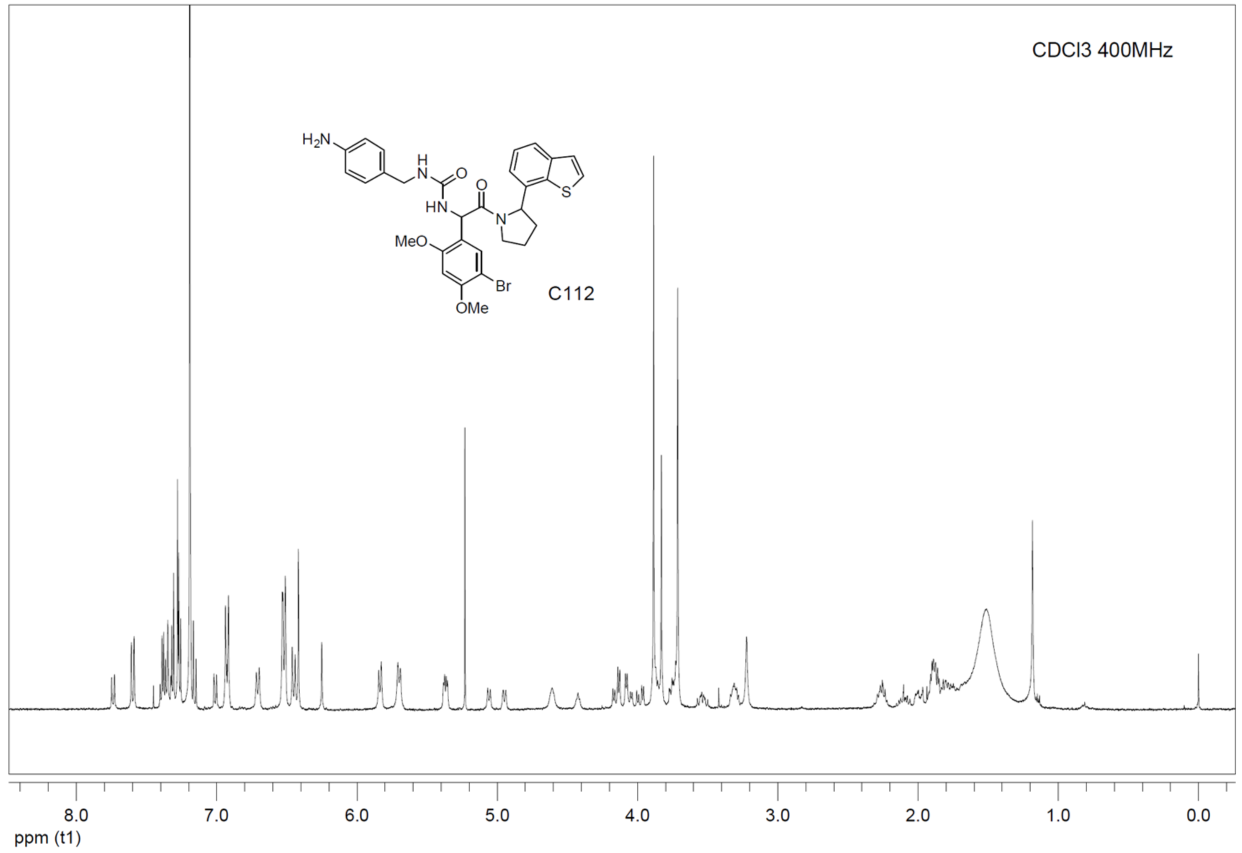

^{1}H NMR (400 MHz, $CDCl_3$) spectrum of compound C112.

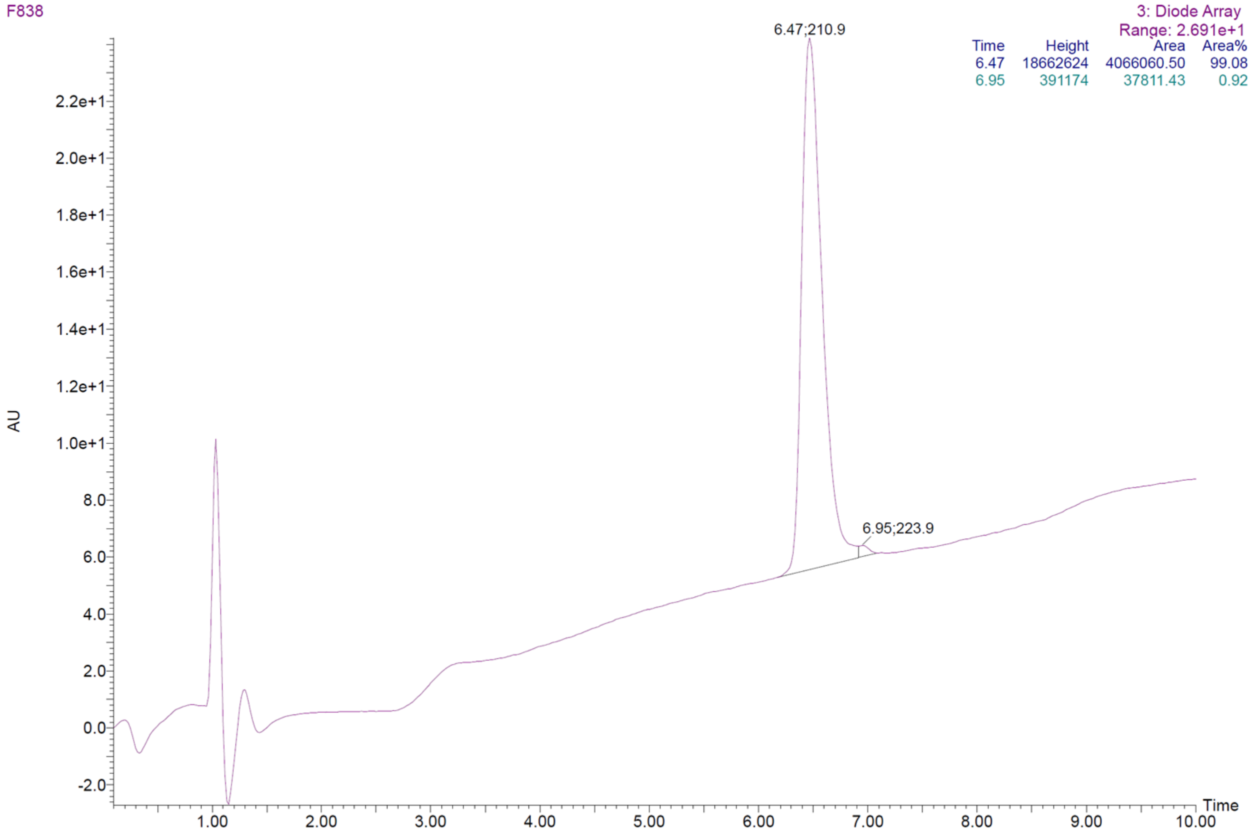

HPLC Chromatogram of compound C112.

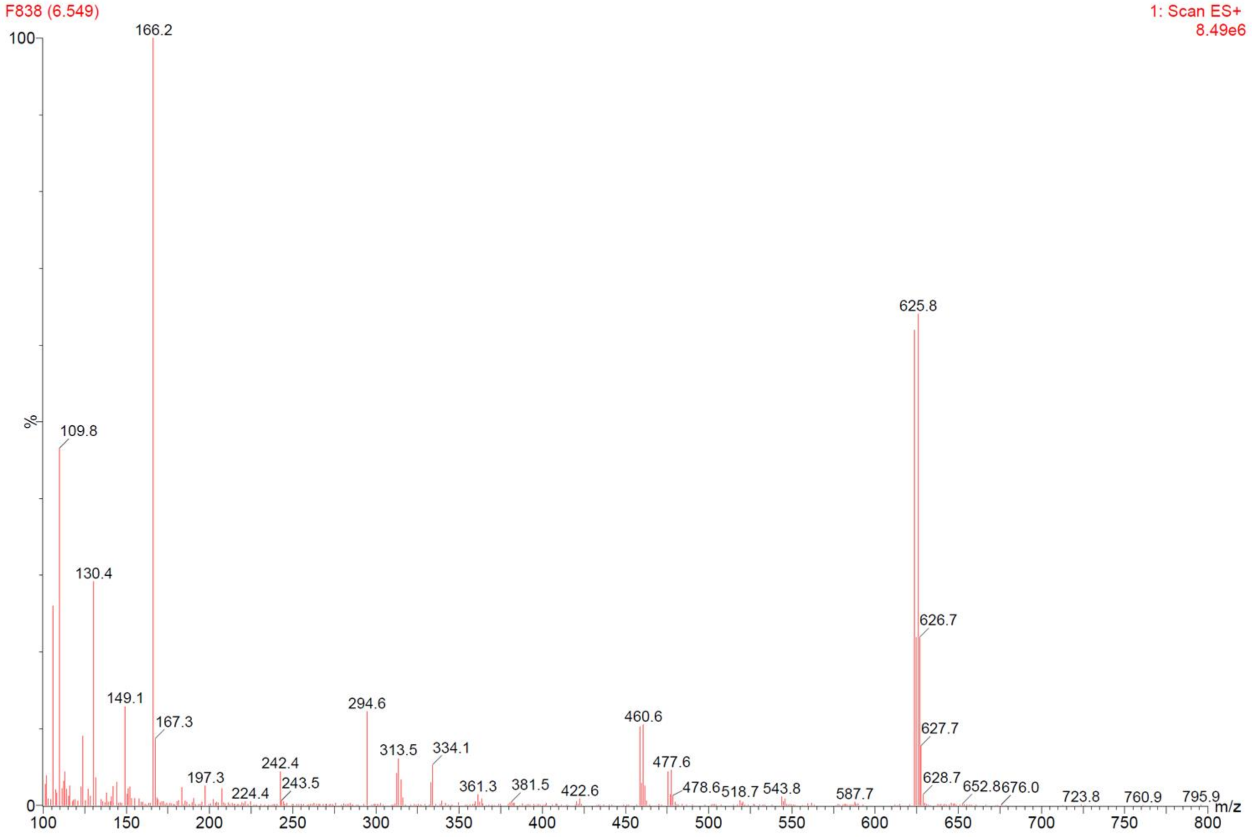

Mass spectrum of compound C112.

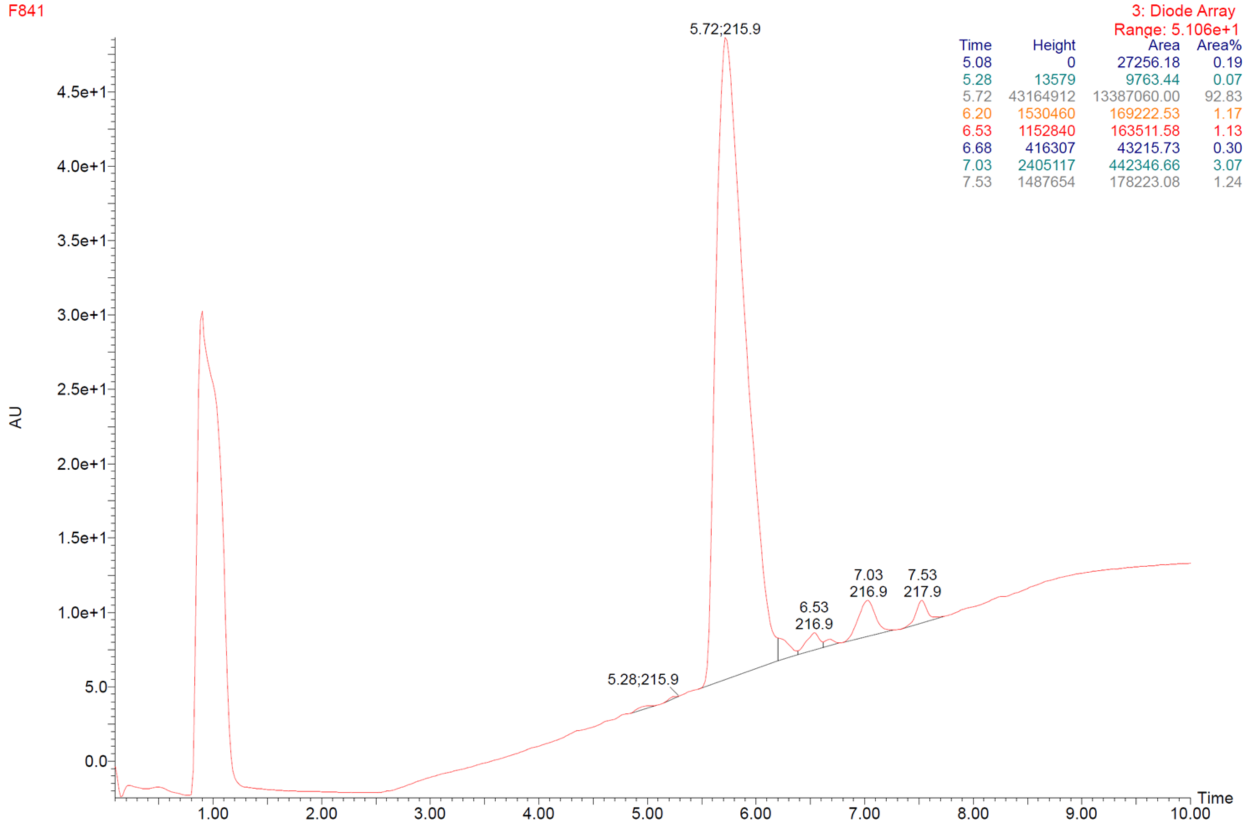

HPLC Chromatogram of compound C115.

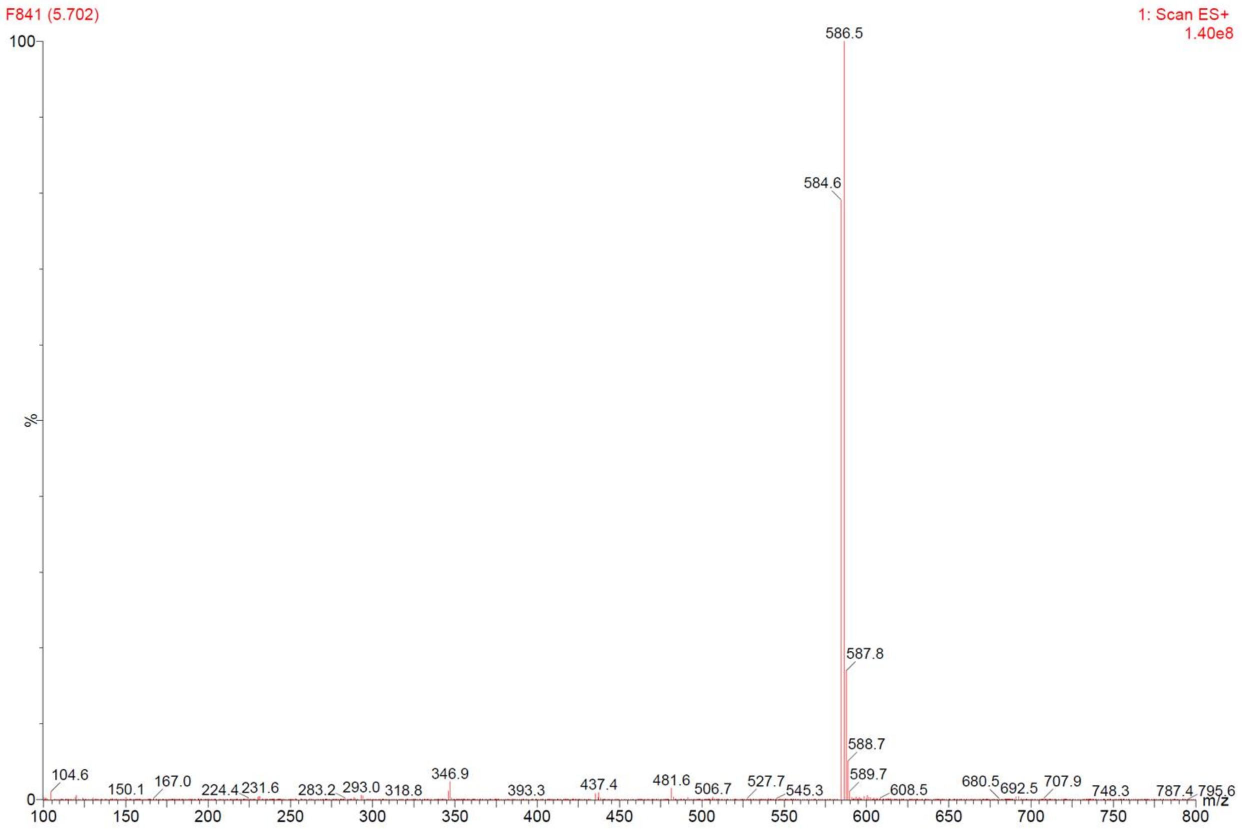

Mass spectrum of compound C115.

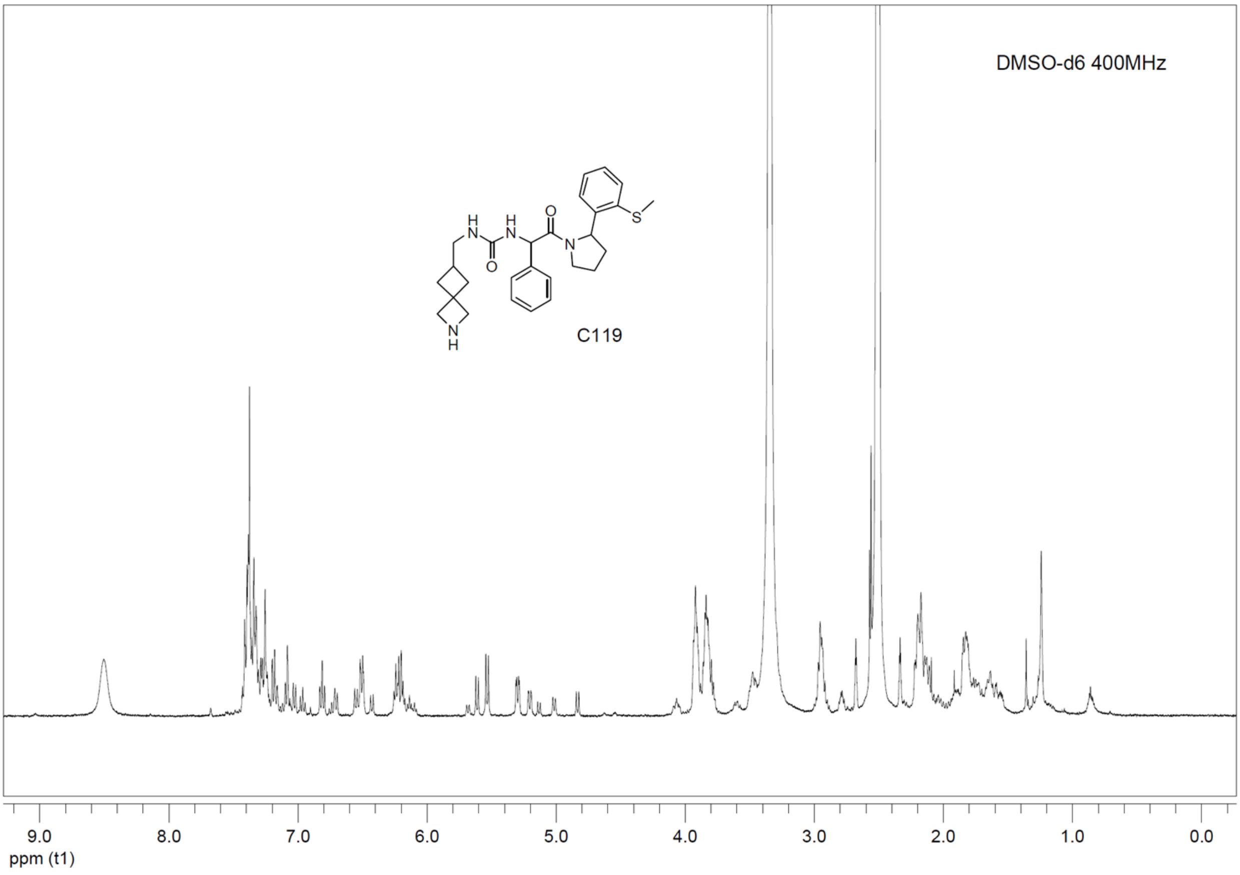

^{1}H NMR (400 MHz, DMSO-d6) spectrum of compound C119.

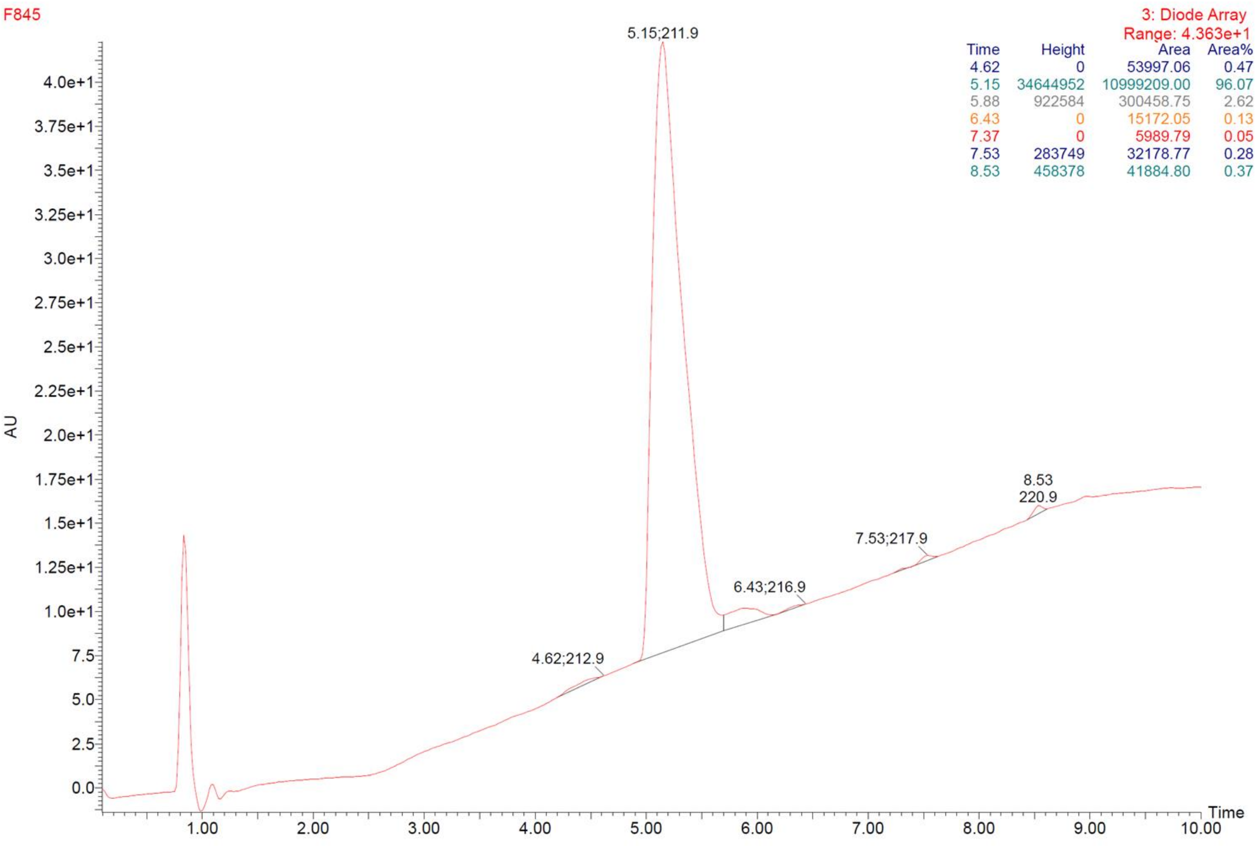

HPLC Chromatogram of compound C119.

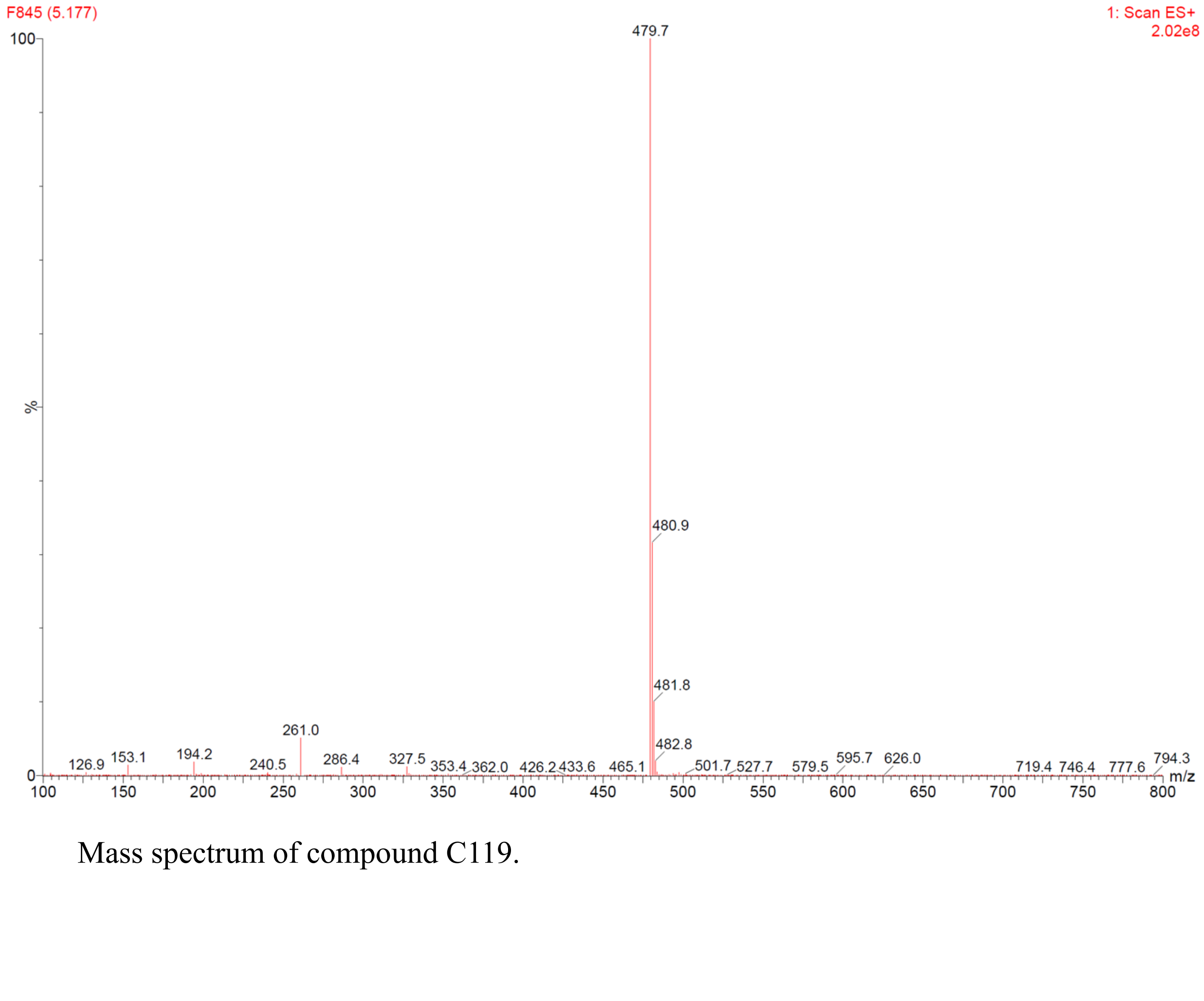

Mass spectrum of compound C119.

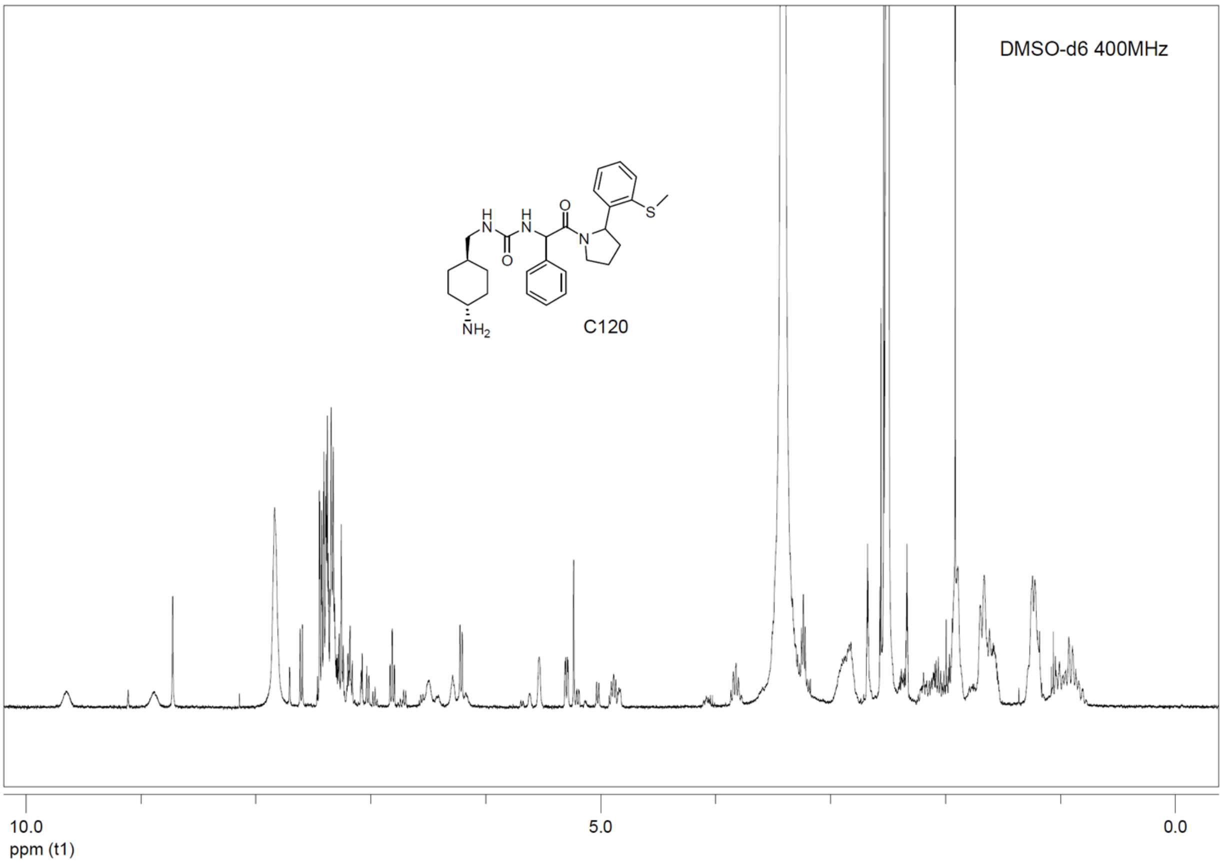

^{1}H NMR (400 MHz, DMSO-d6) spectrum of compound C120.

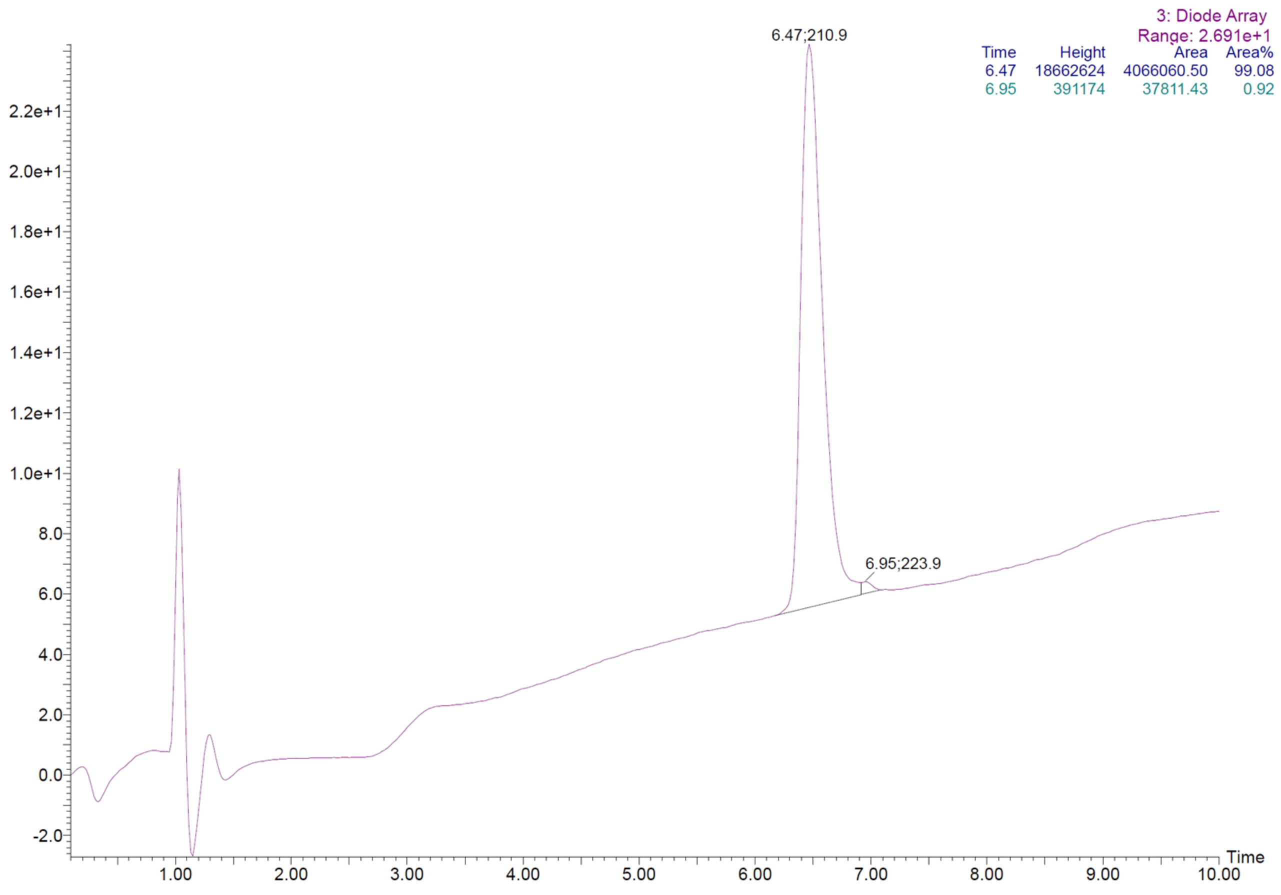

HPLC Chromatogram of compound C120.

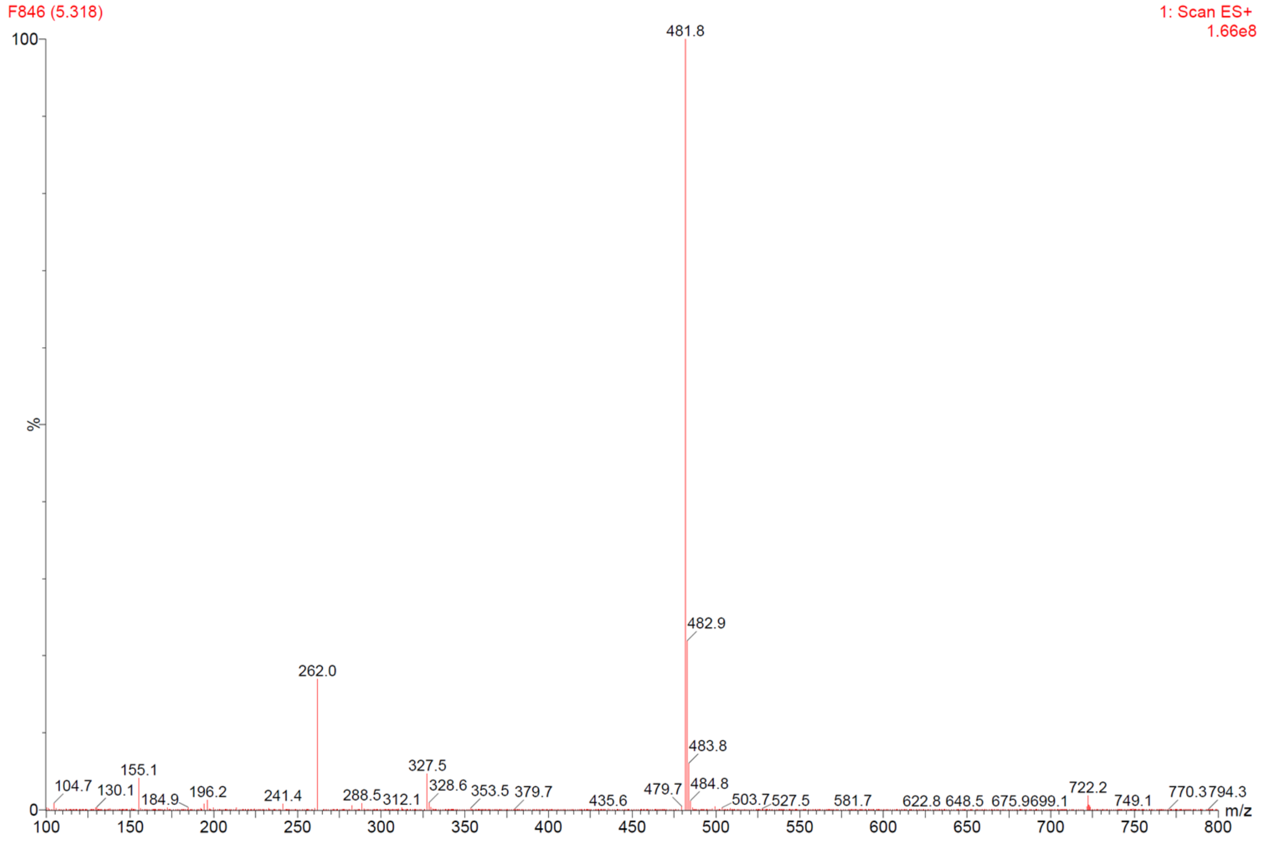

Mass spectrum of compound C120.

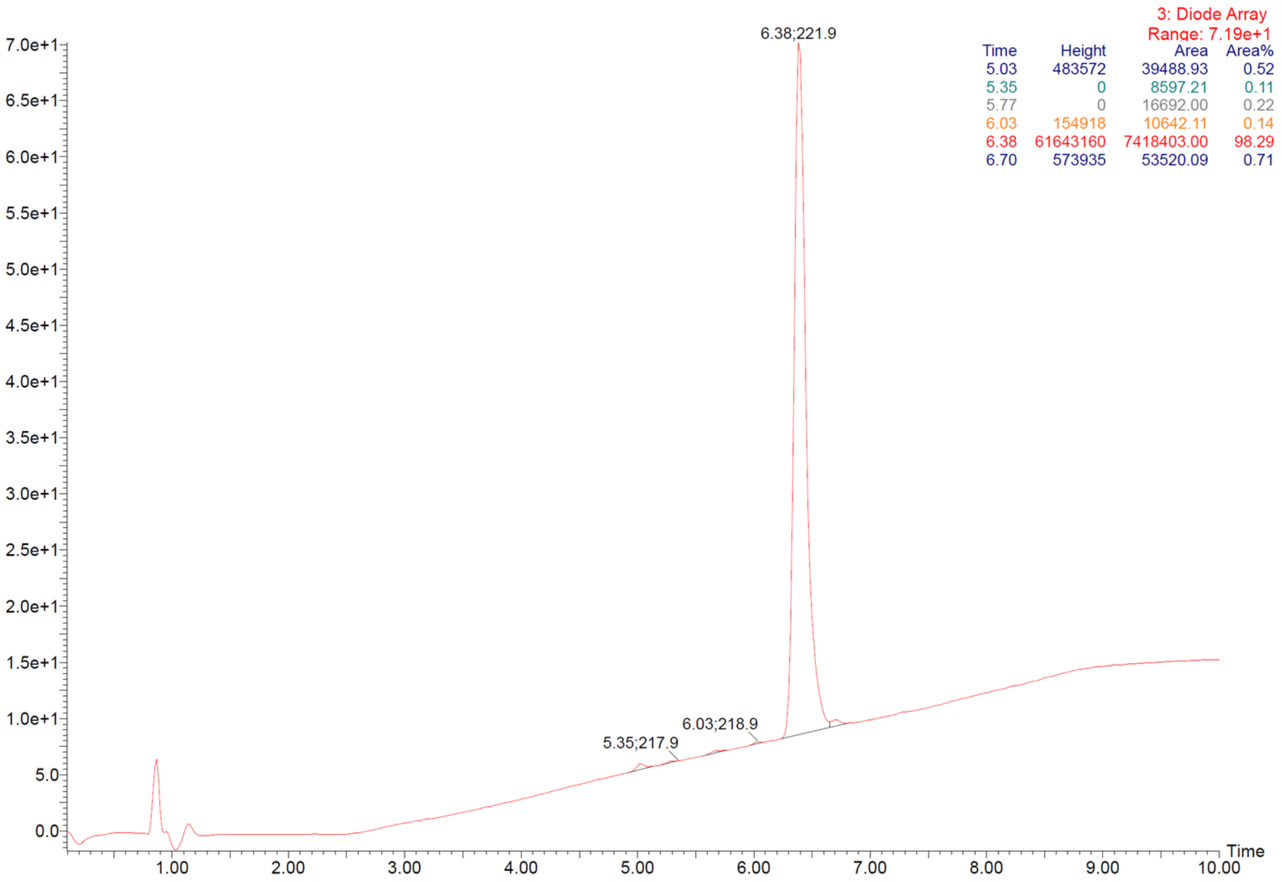

HPLC Chromatogram of compound C123.

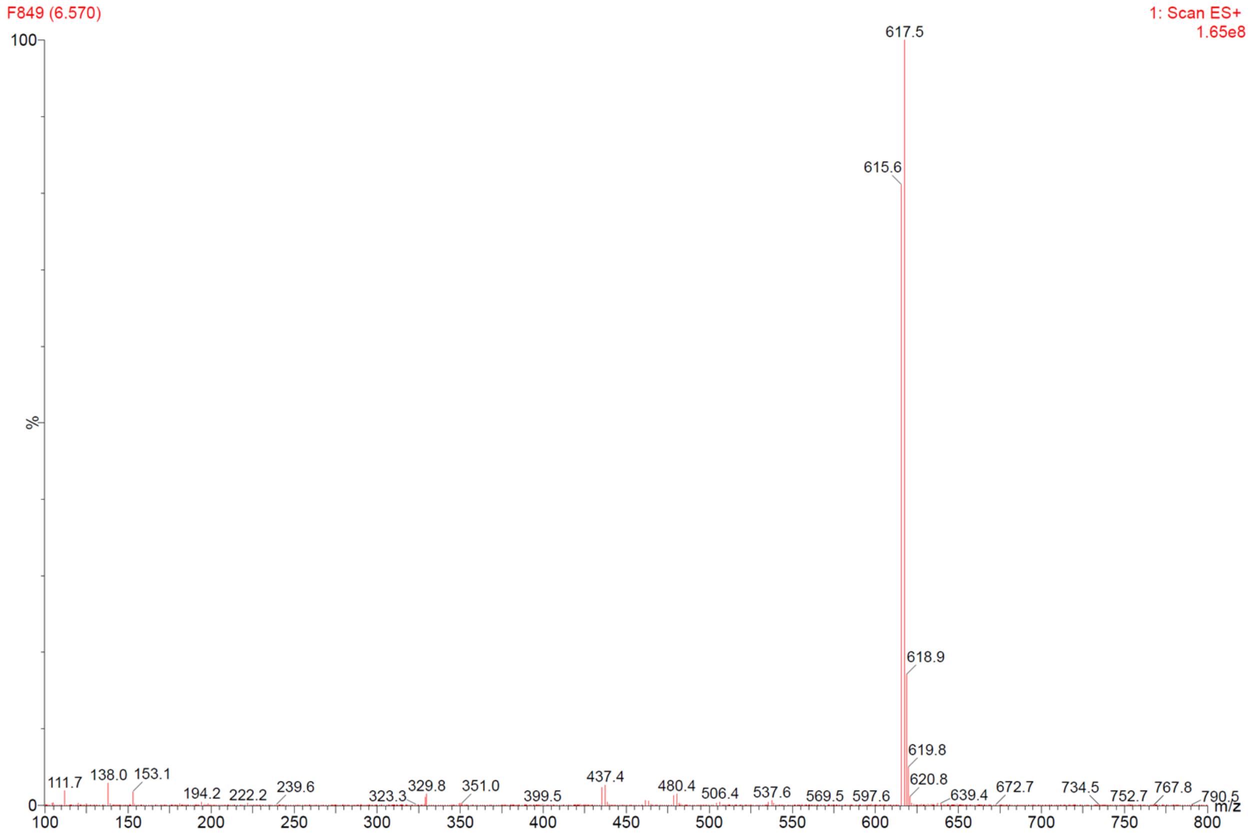

Mass spectrum of compound C123.

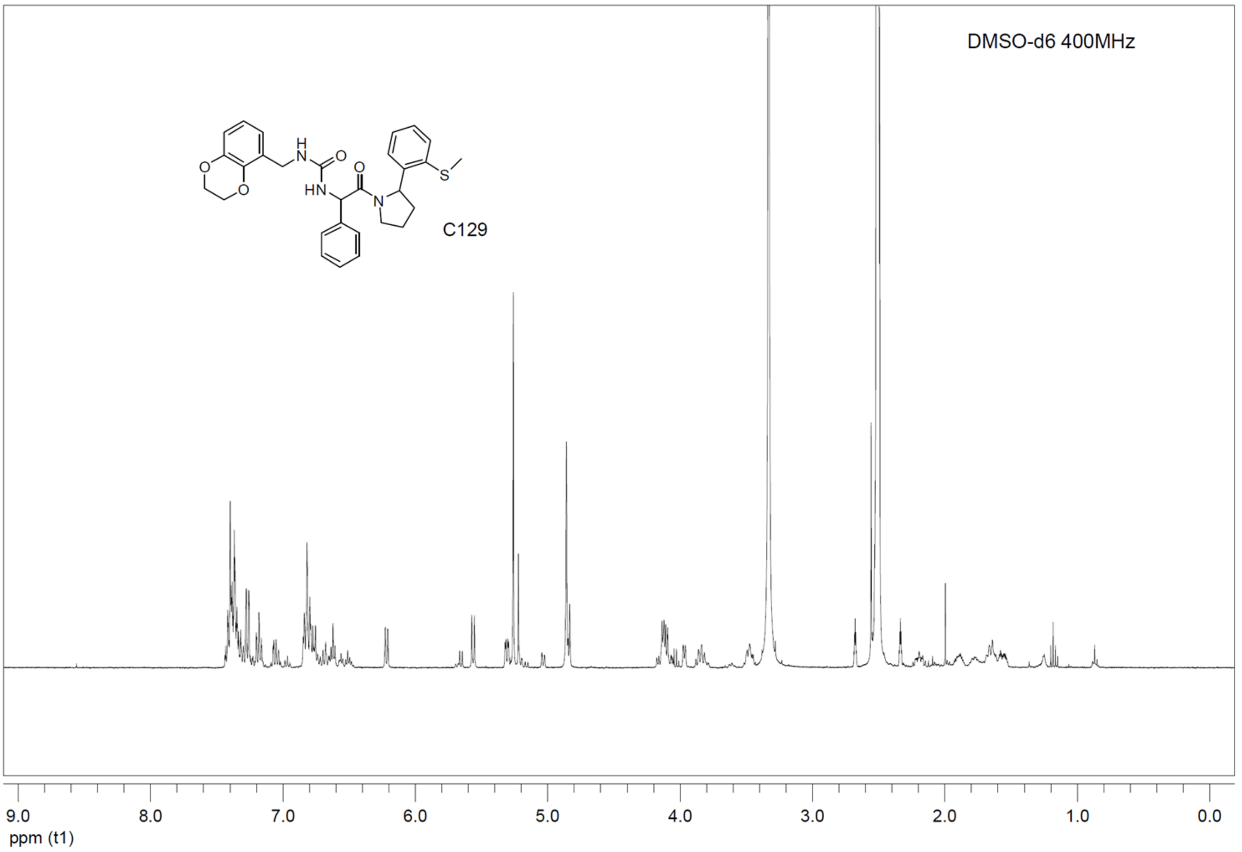

^{1}H NMR (400 MHz, DMSO-d6) spectrum of compound C129.

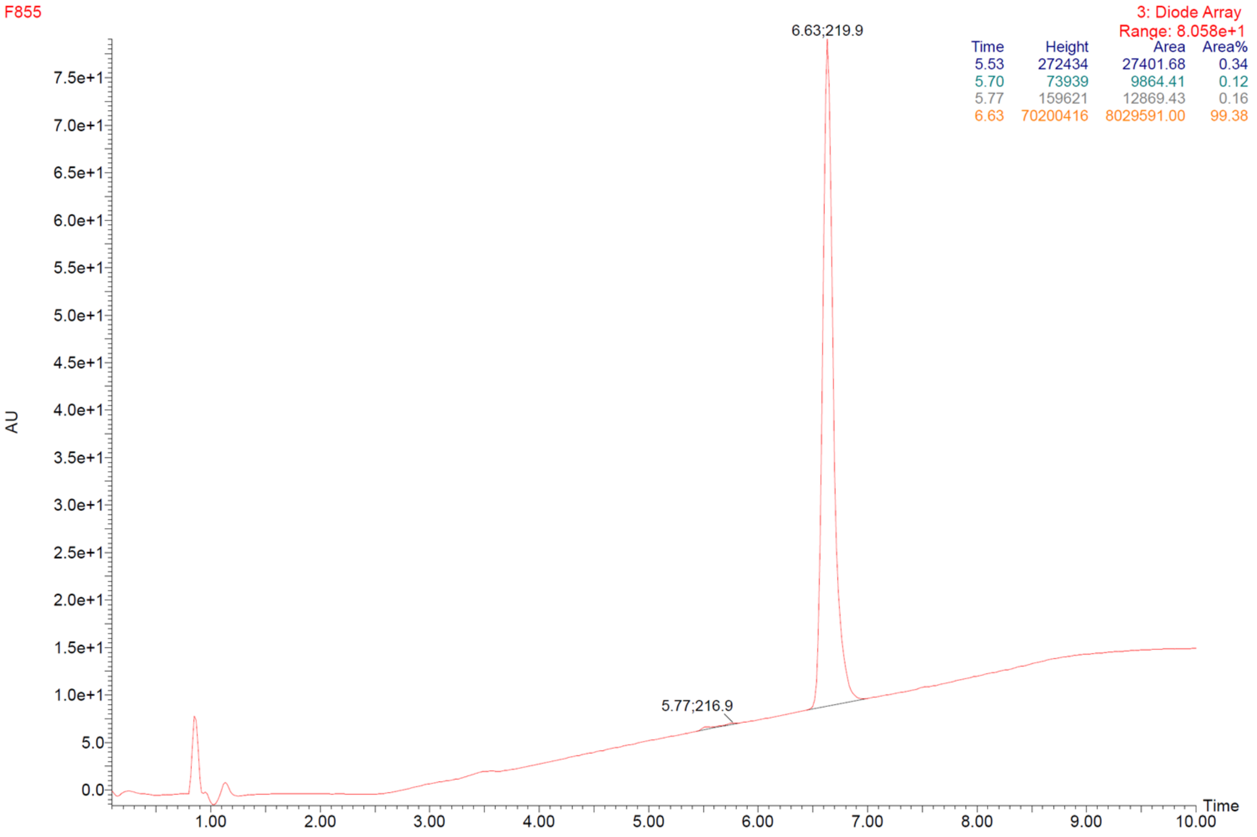

HPLC Chromatogram of compound C129.

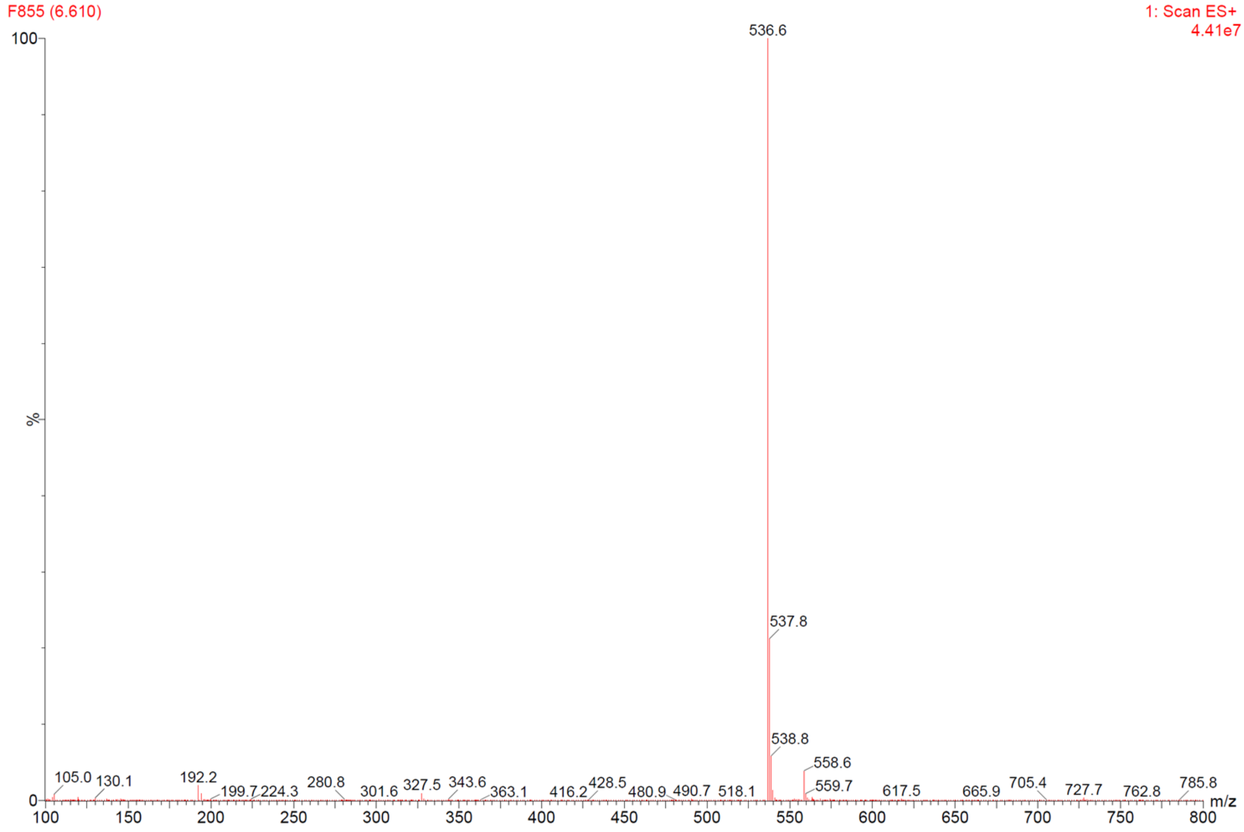

Mass spectrum of compound C129.

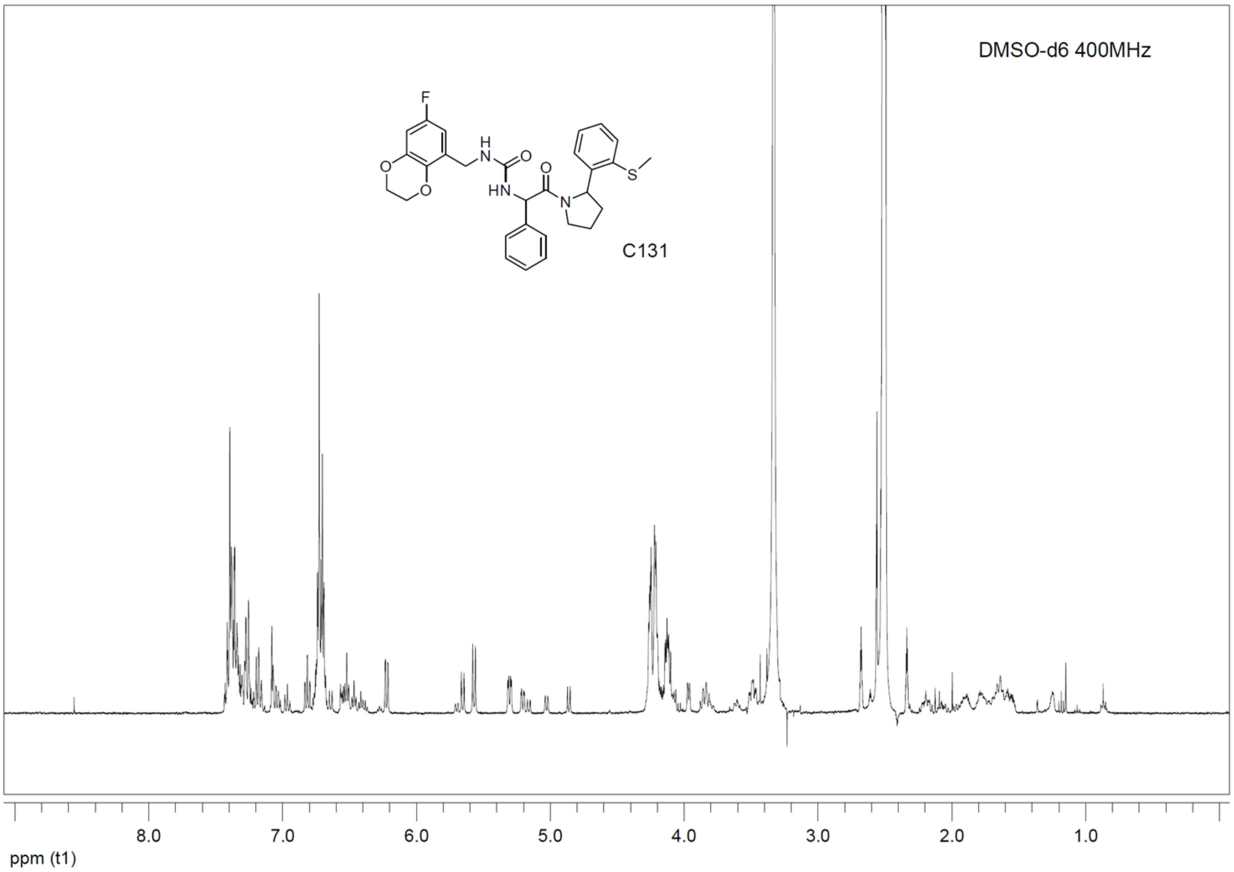

^{1}H NMR (400 MHz, DMSO-d6) spectrum of compound C131.

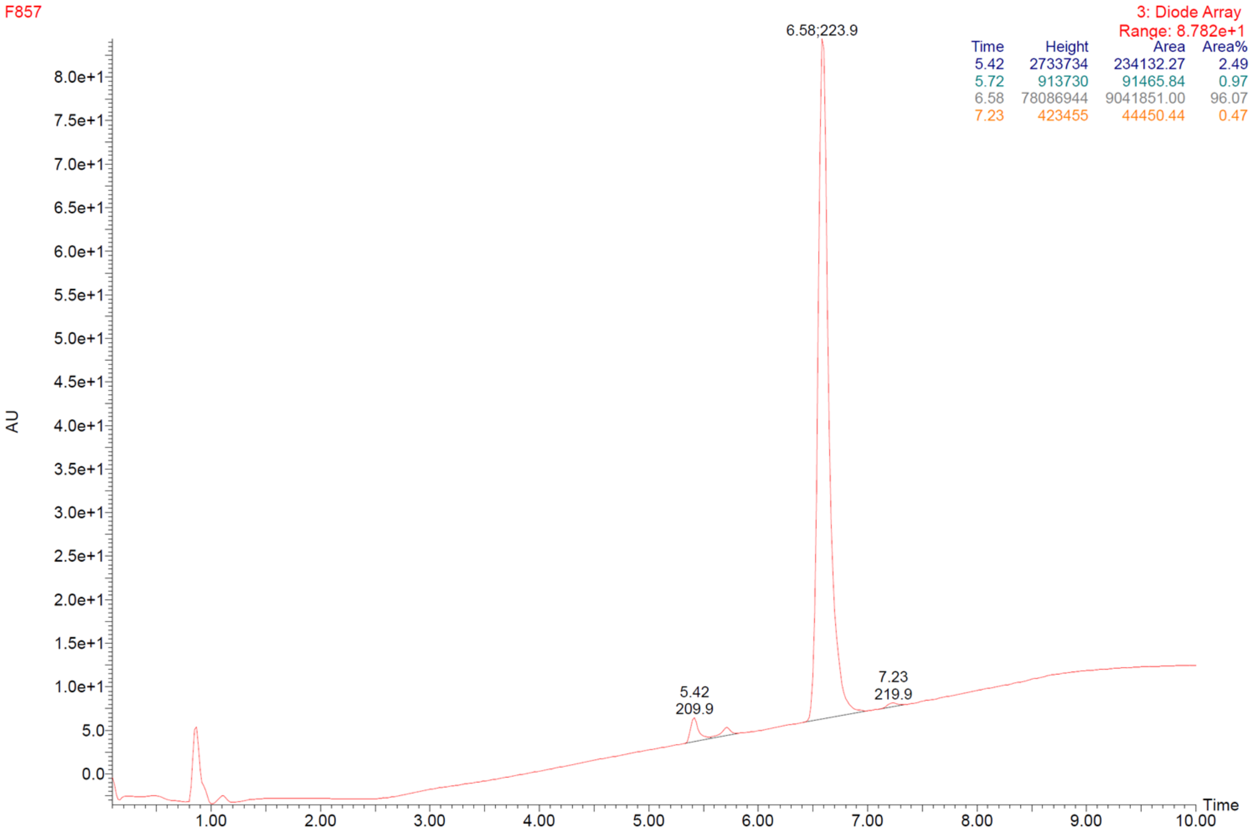

HPLC Chromatogram of compound C131.

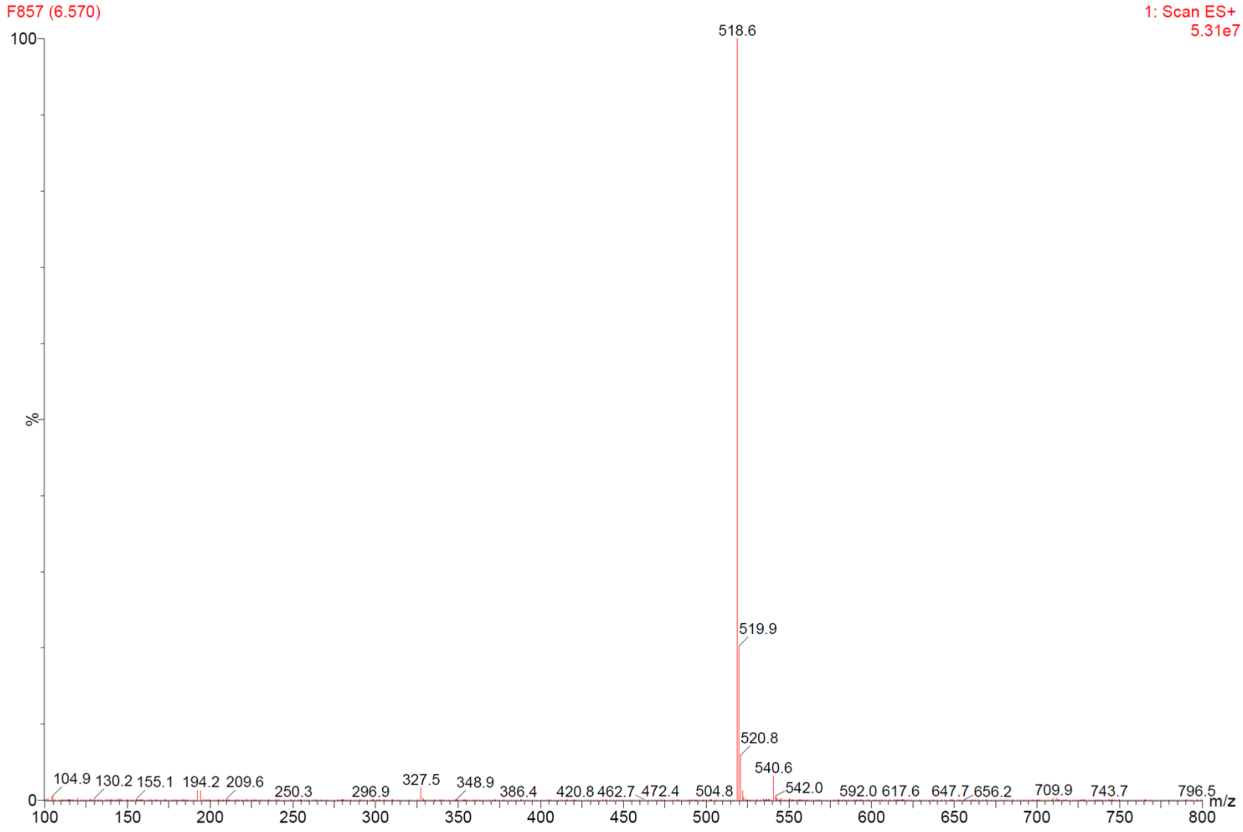

Mass spectrum of compound C131.

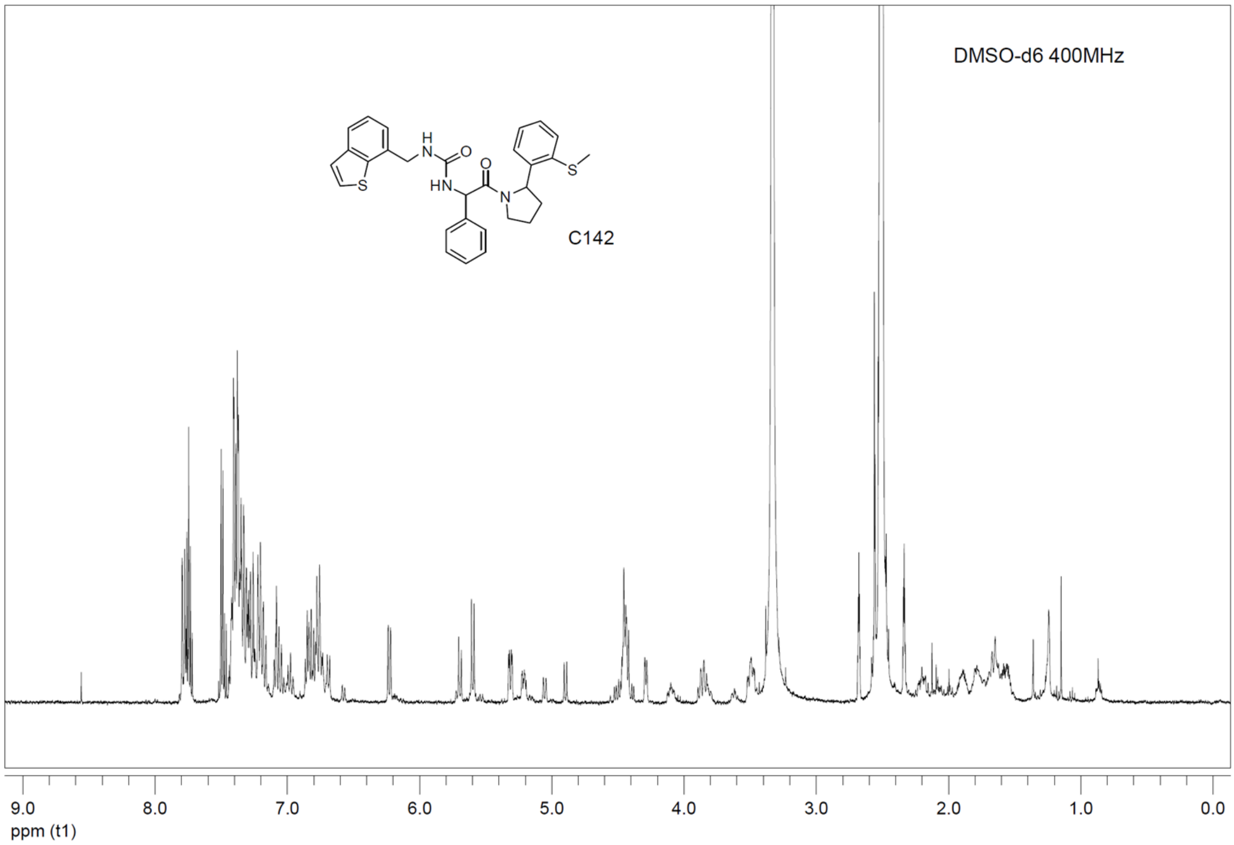

^{1}H NMR (400 MHz, DMSO-d6) spectrum of compound C142.

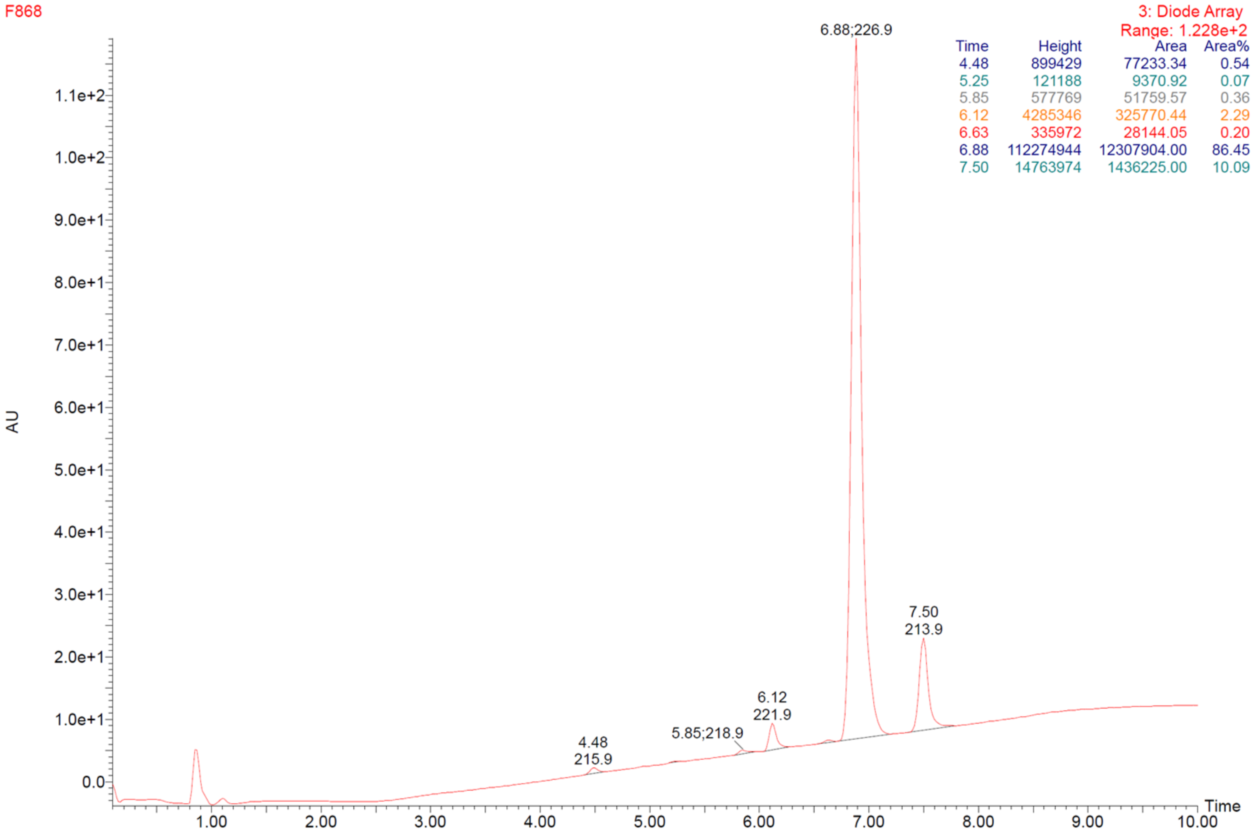

HPLC Chromatogram of compound C142.

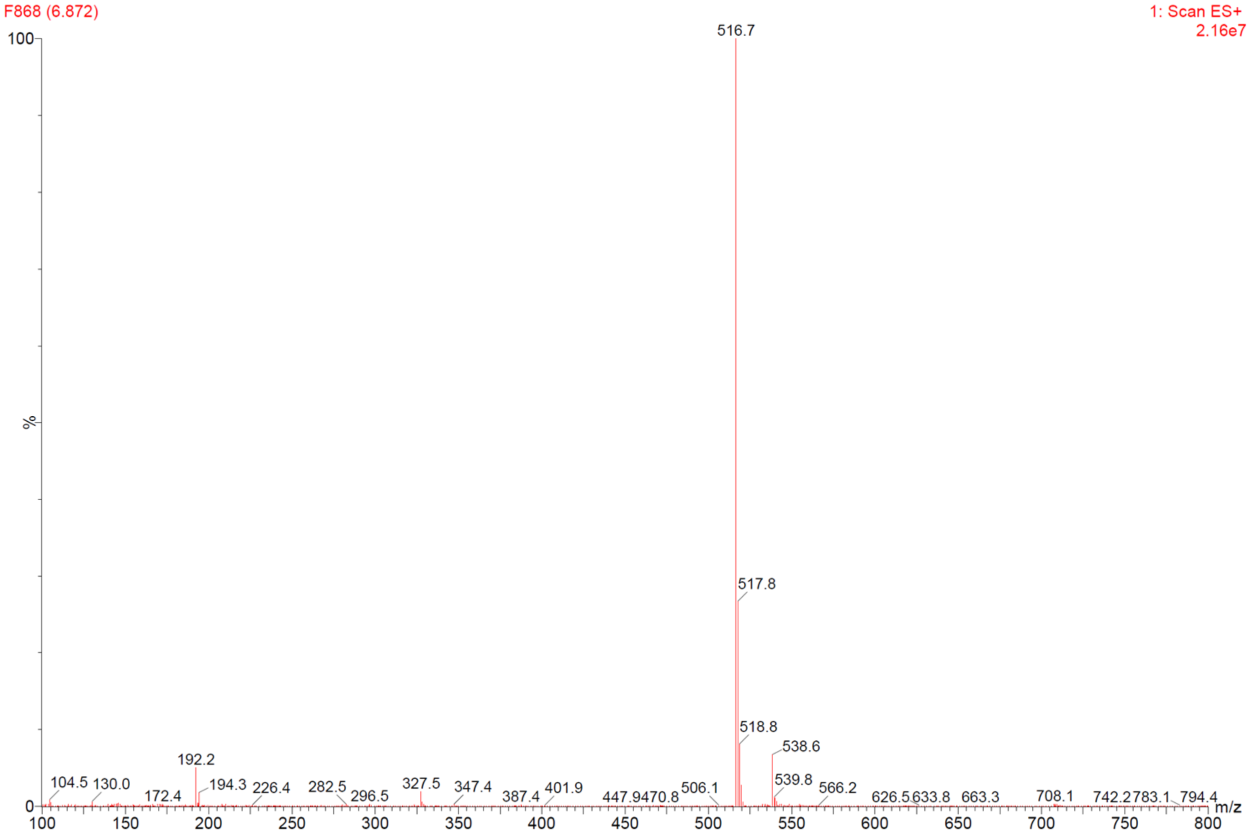

Mass spectrum of compound C142.

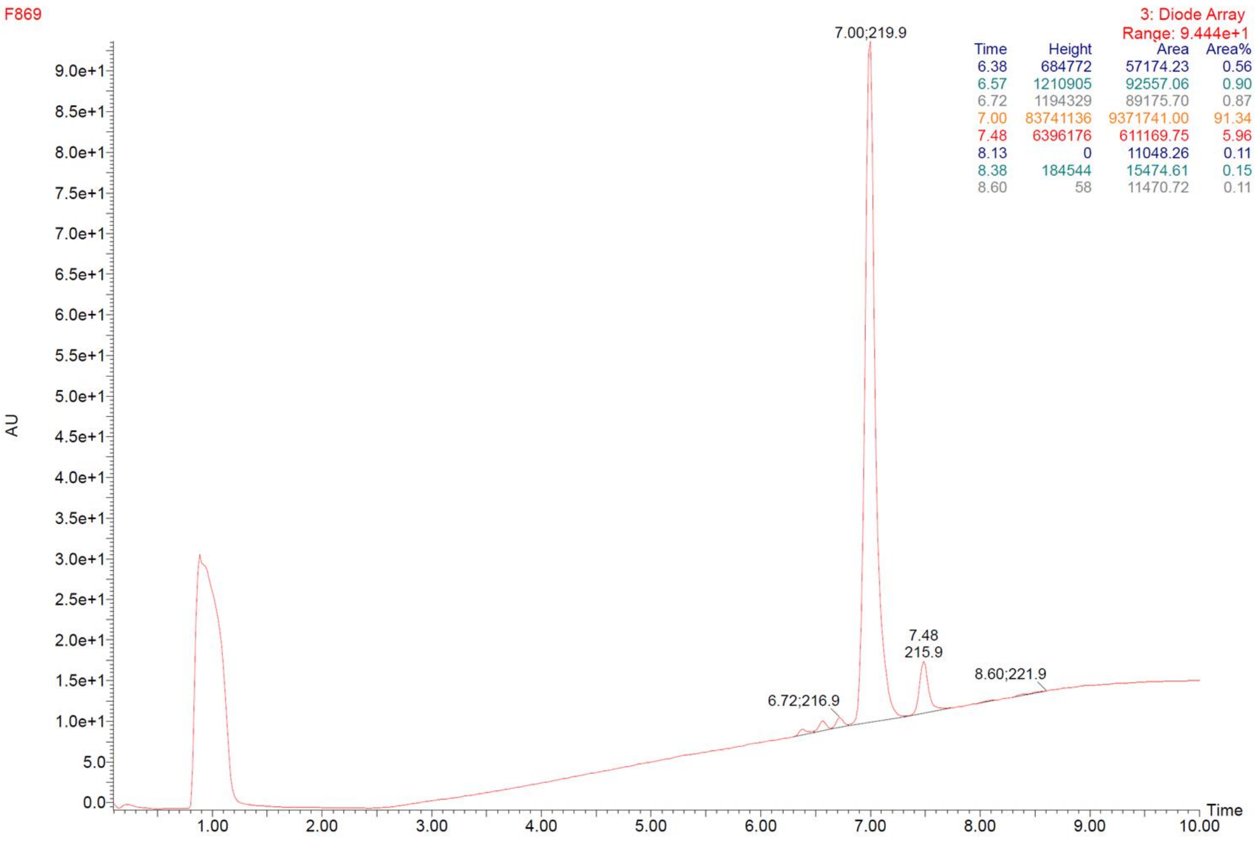

HPLC Chromatogram of compound C143.

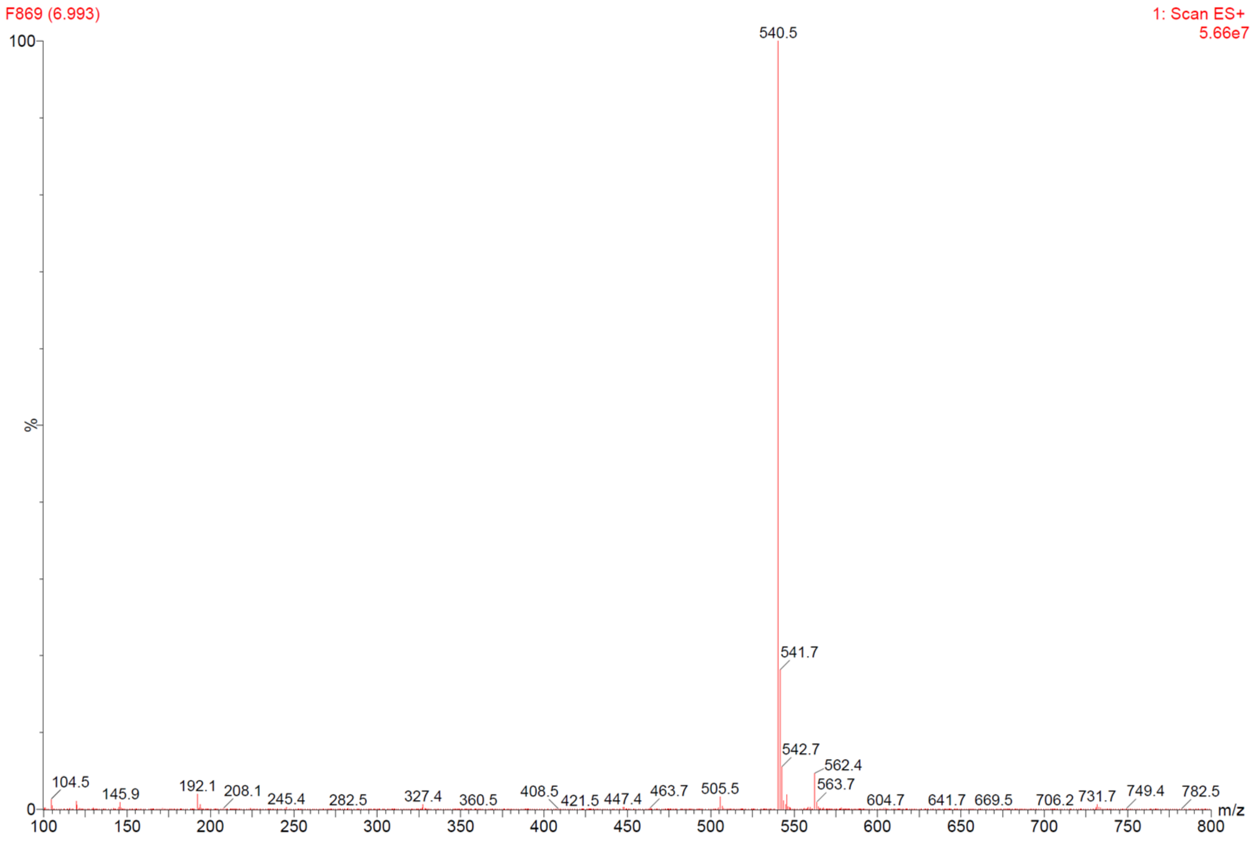

Mass spectrum of compound C143.

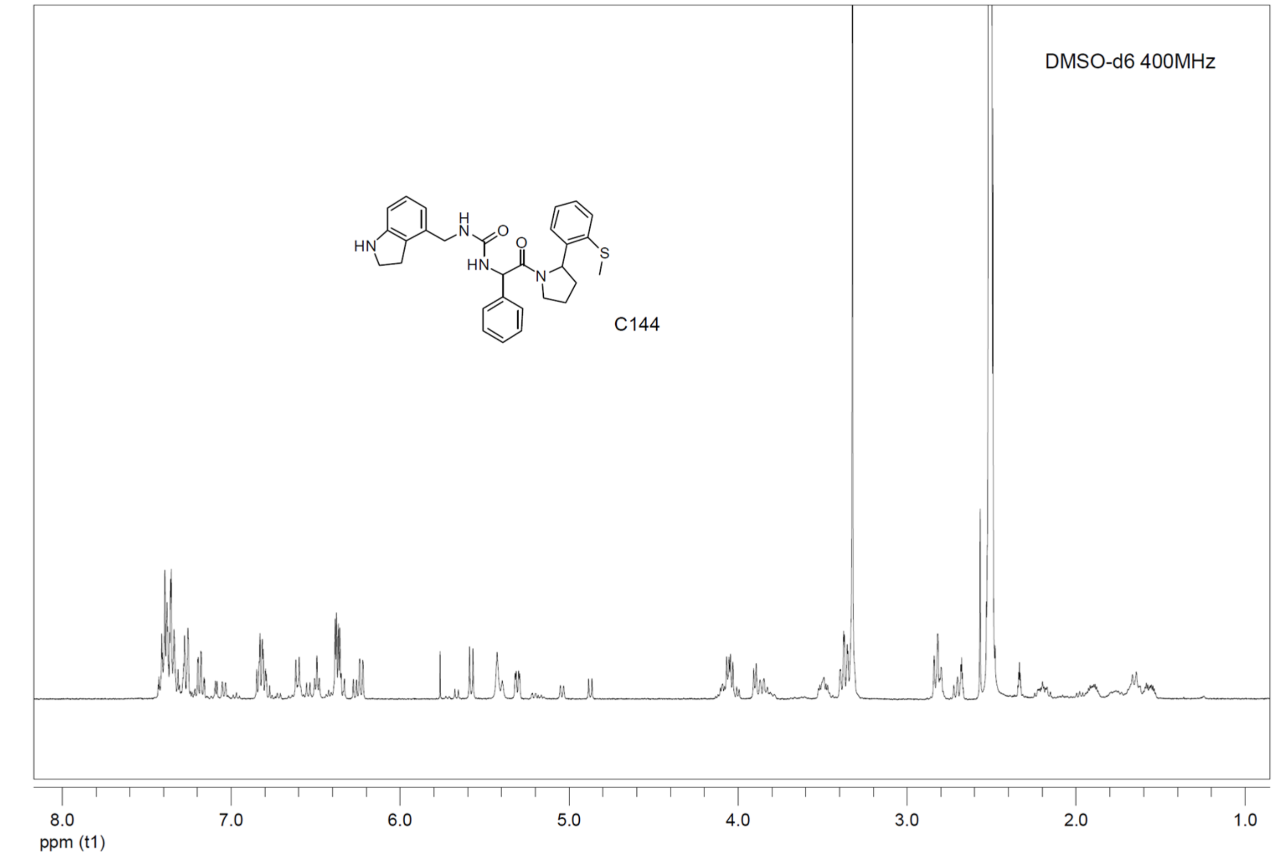

^{1}H NMR (400 MHz, DMSO-d6) spectrum of compound C144.

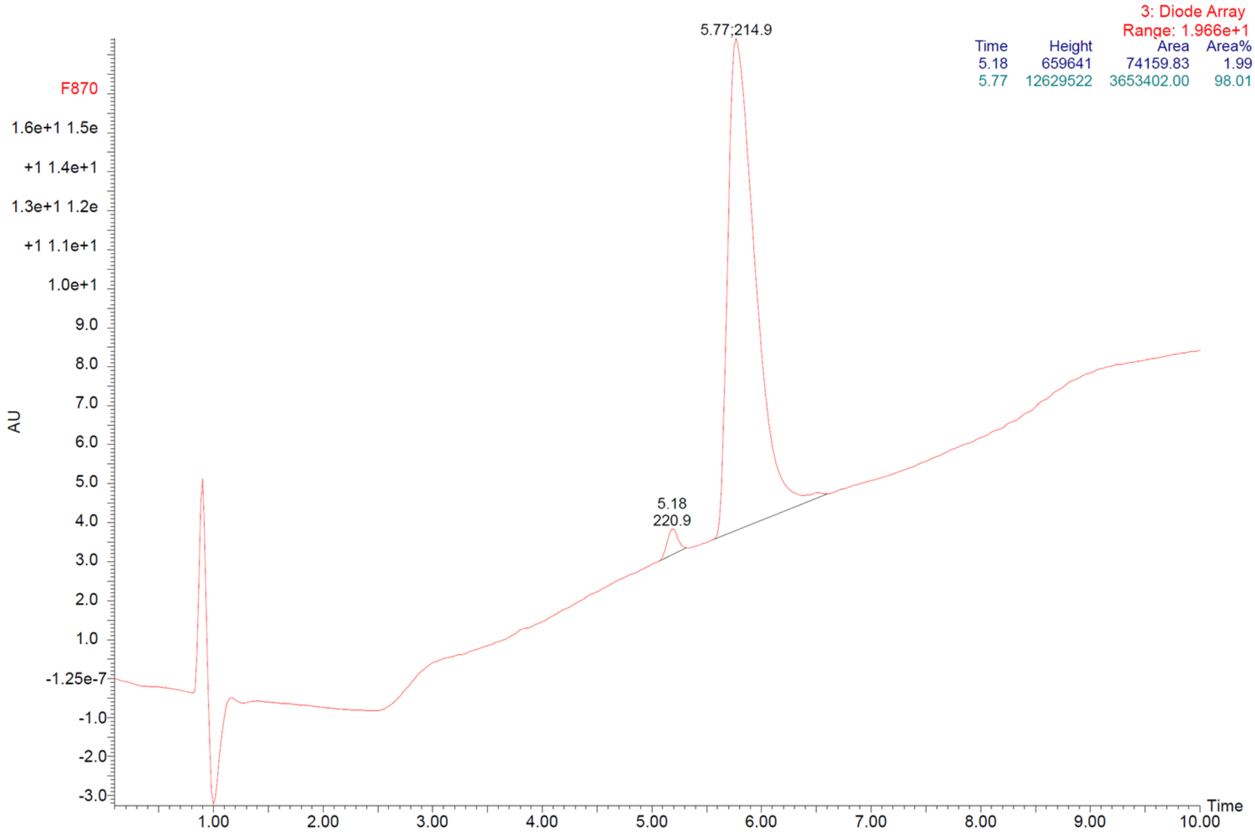

HPLC Chromatogram of compound C144.

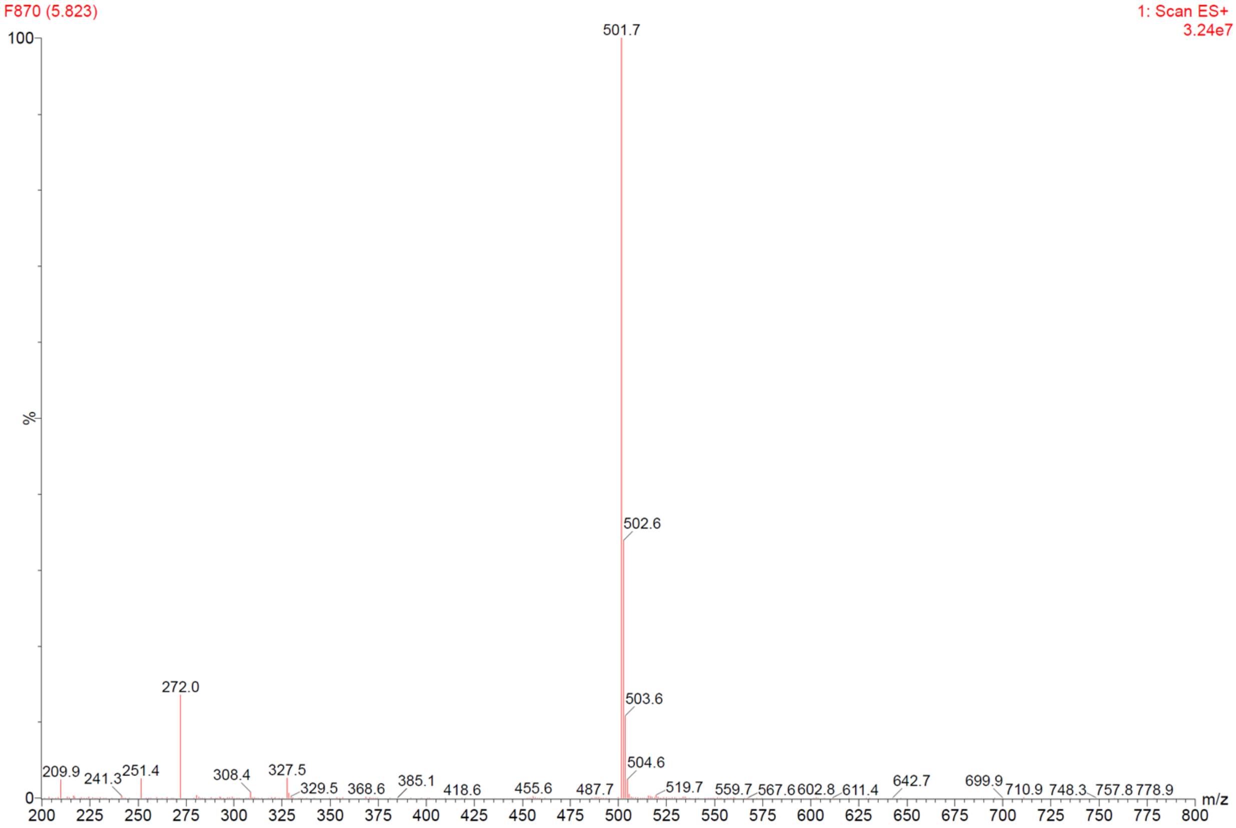

Mass spectrum of compound C144.

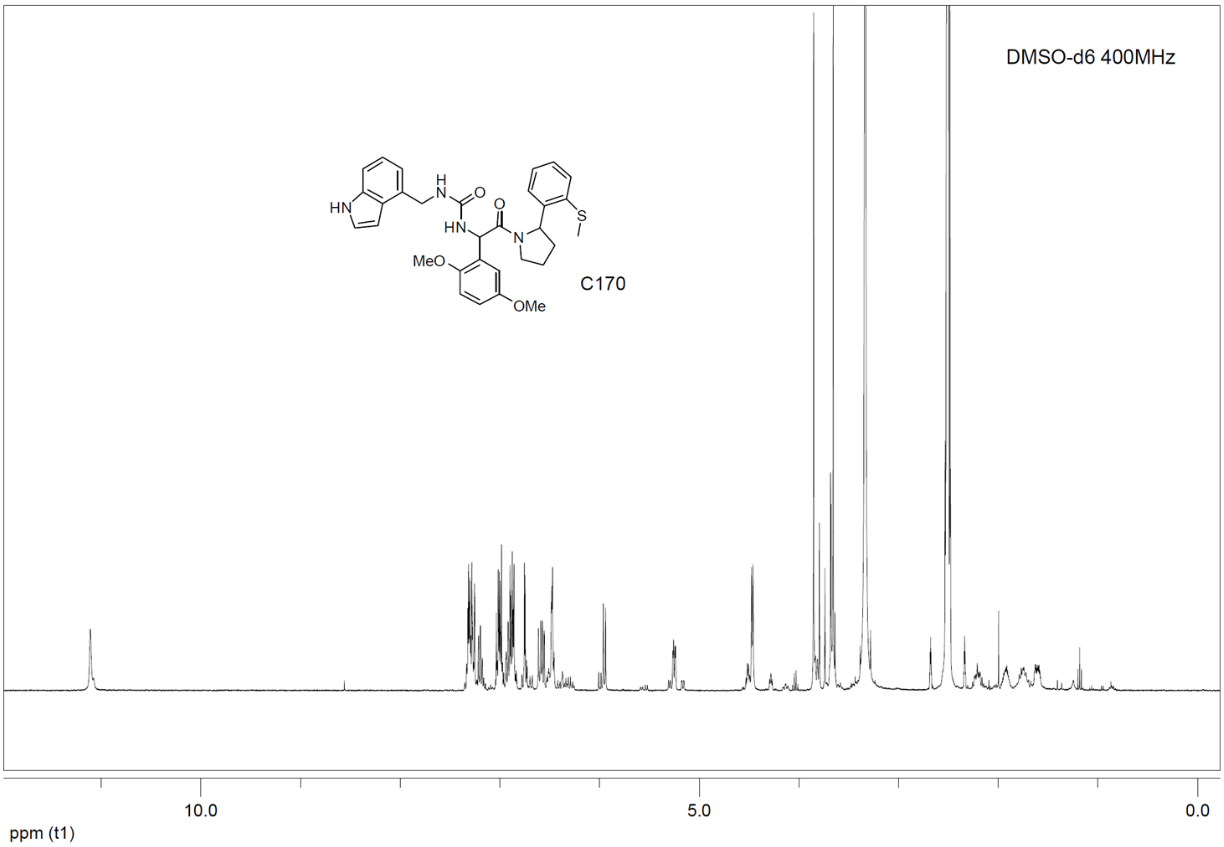

^{1}H NMR (400 MHz, DMSO-d6) spectrum of compound C170.

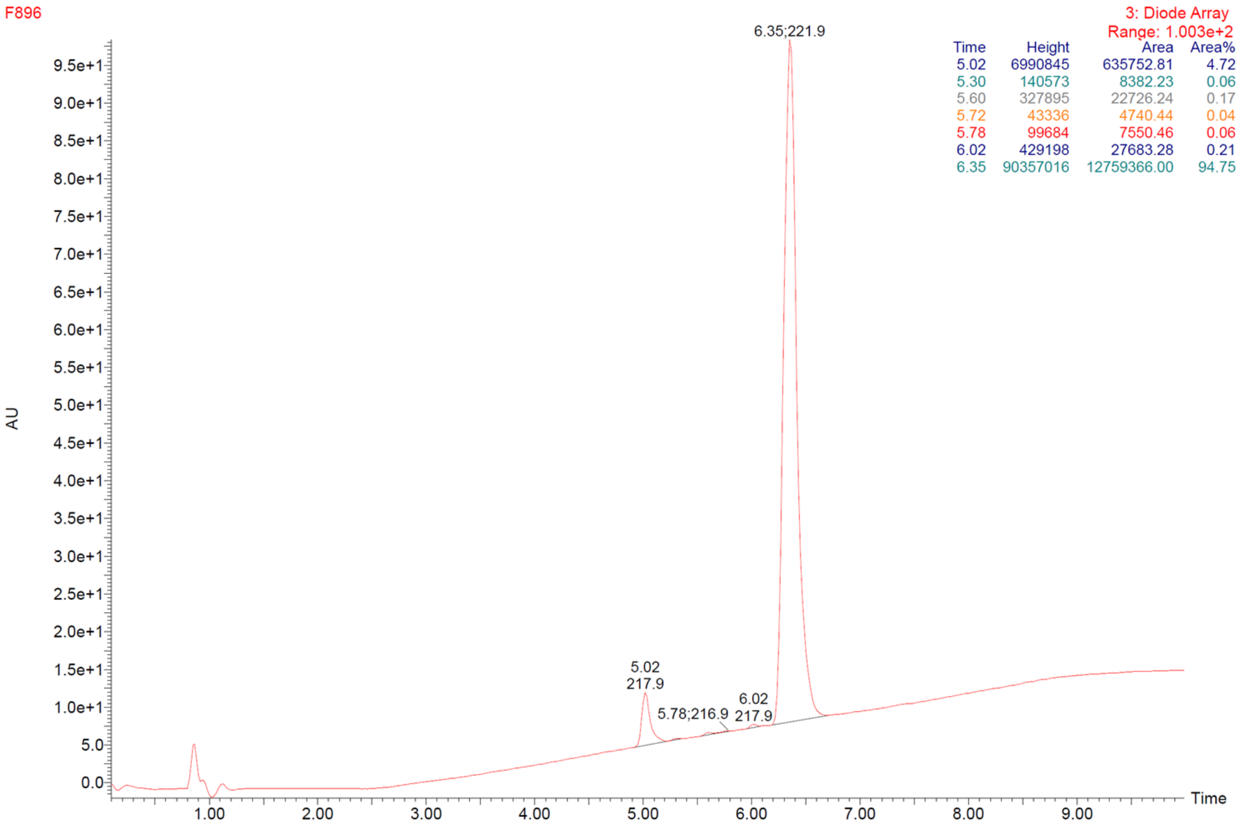

HPLC Chromatogram of compound C170.

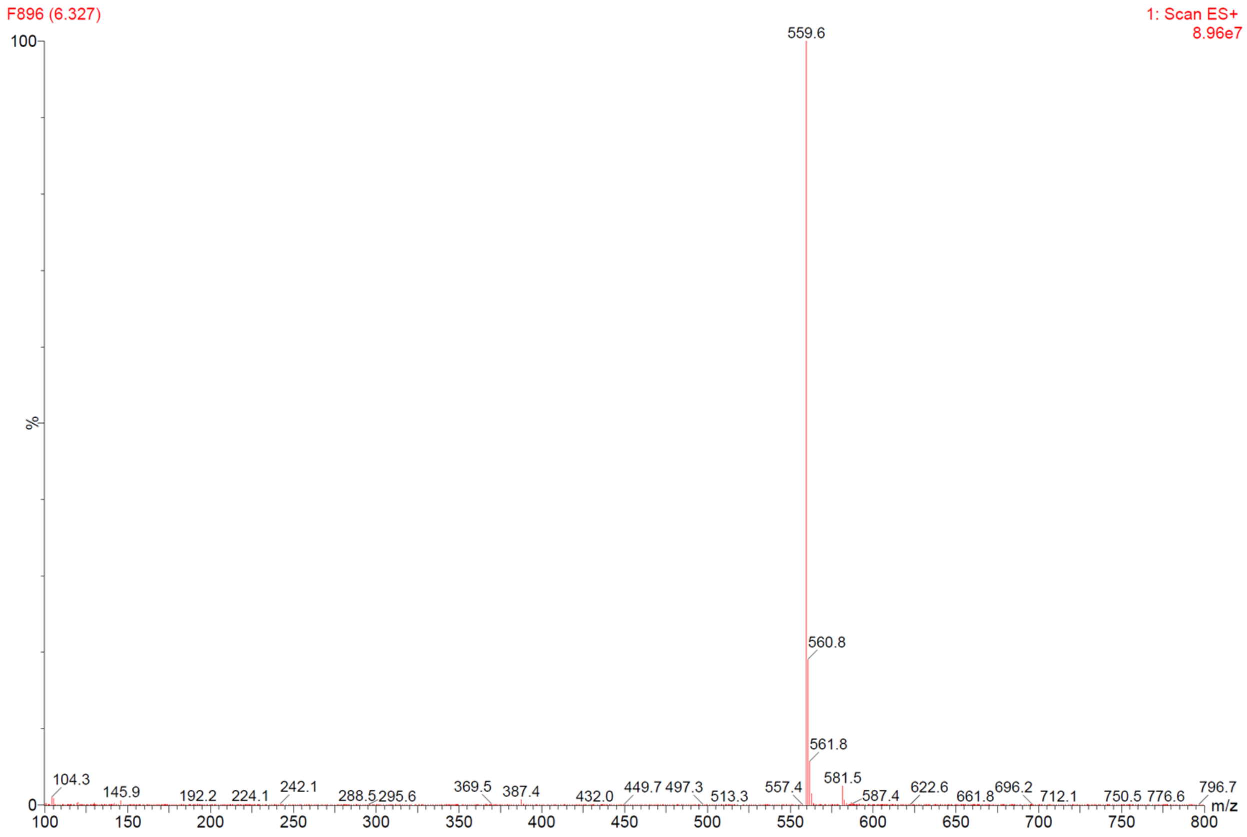

Mass spectrum of compound C170.

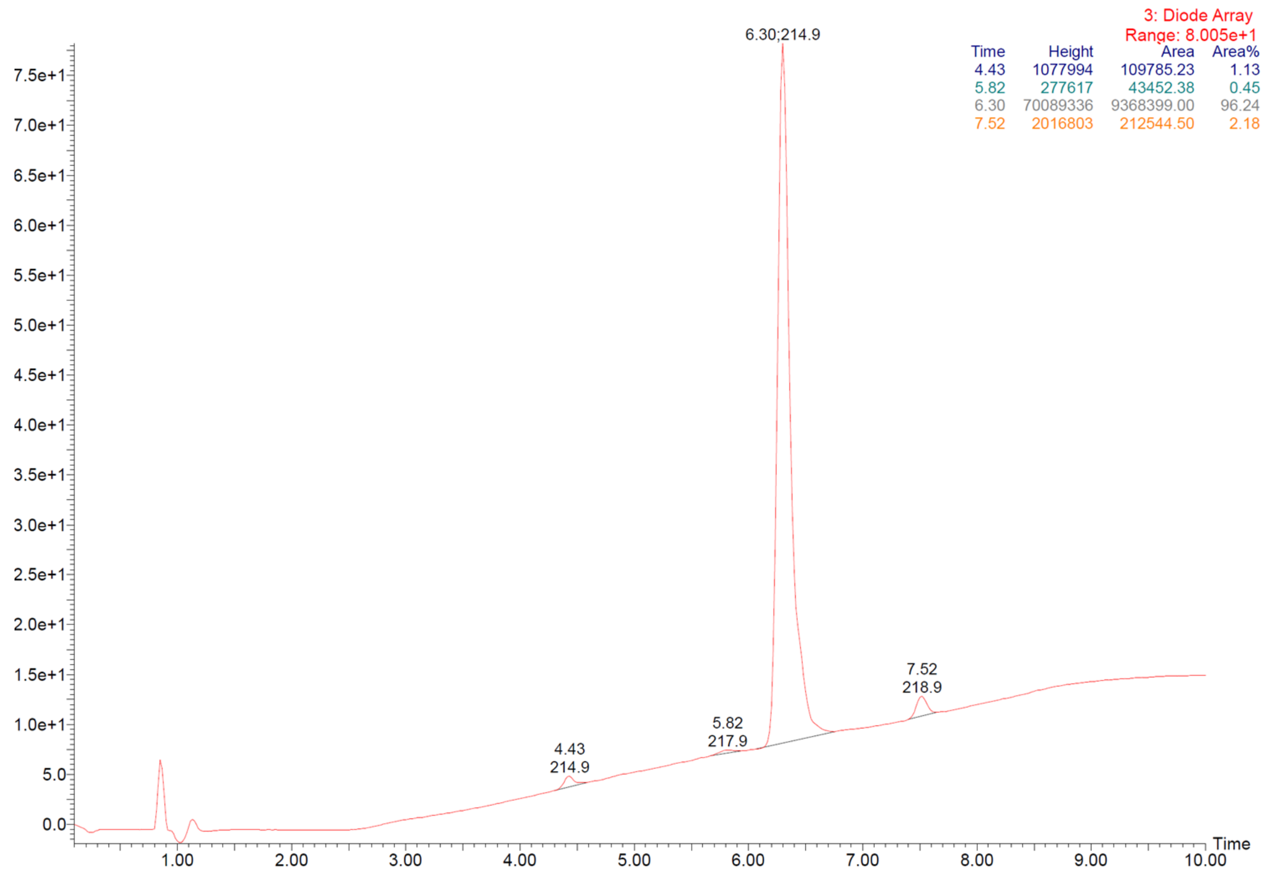

HPLC Chromatogram of compound C171.

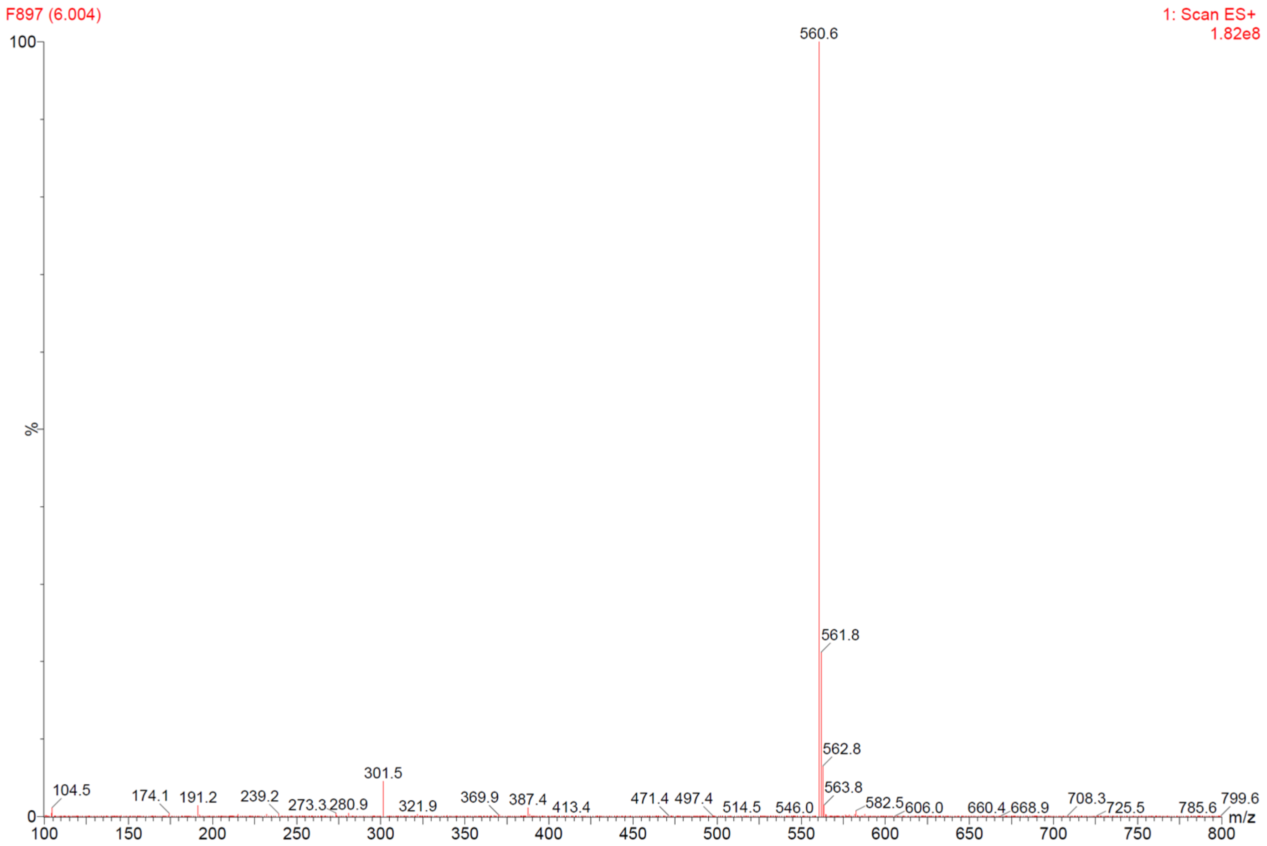

Mass spectrum of compound C171.

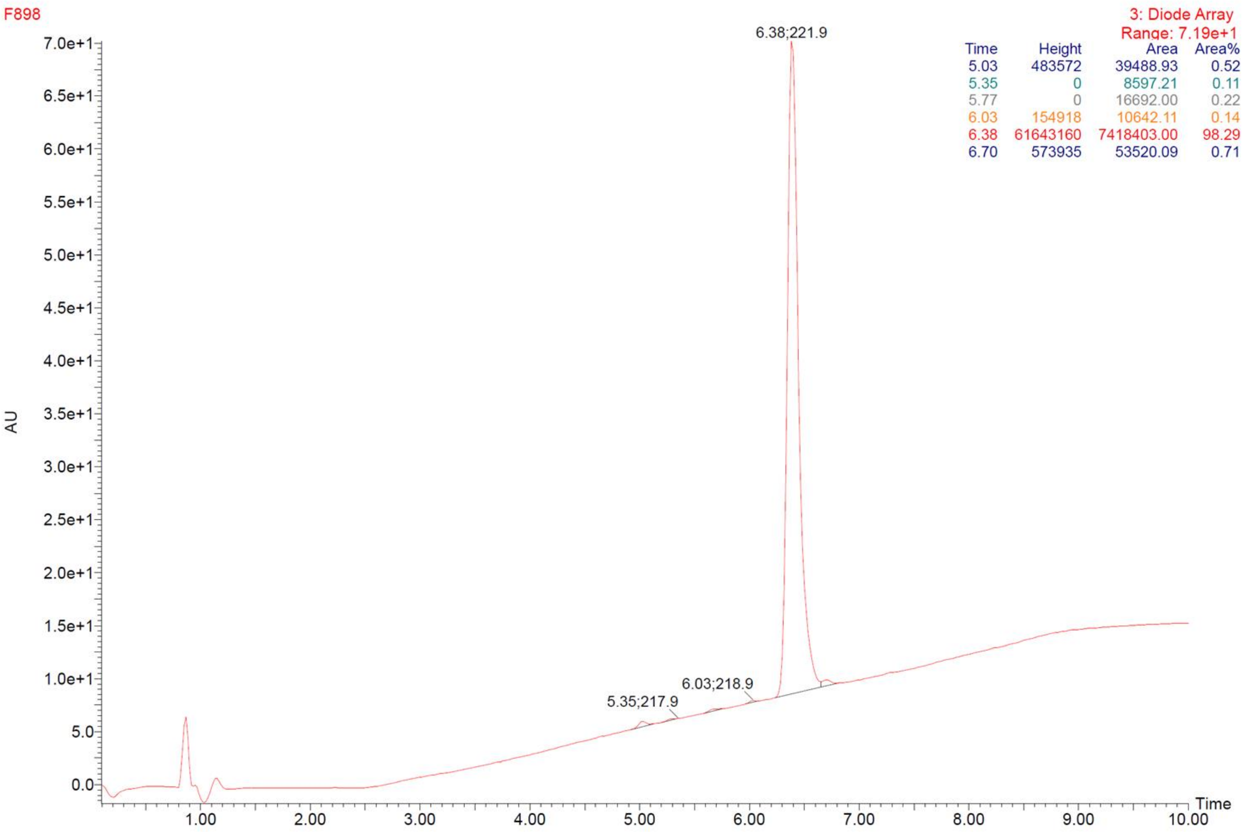

HPLC Chromatogram of compound C172.

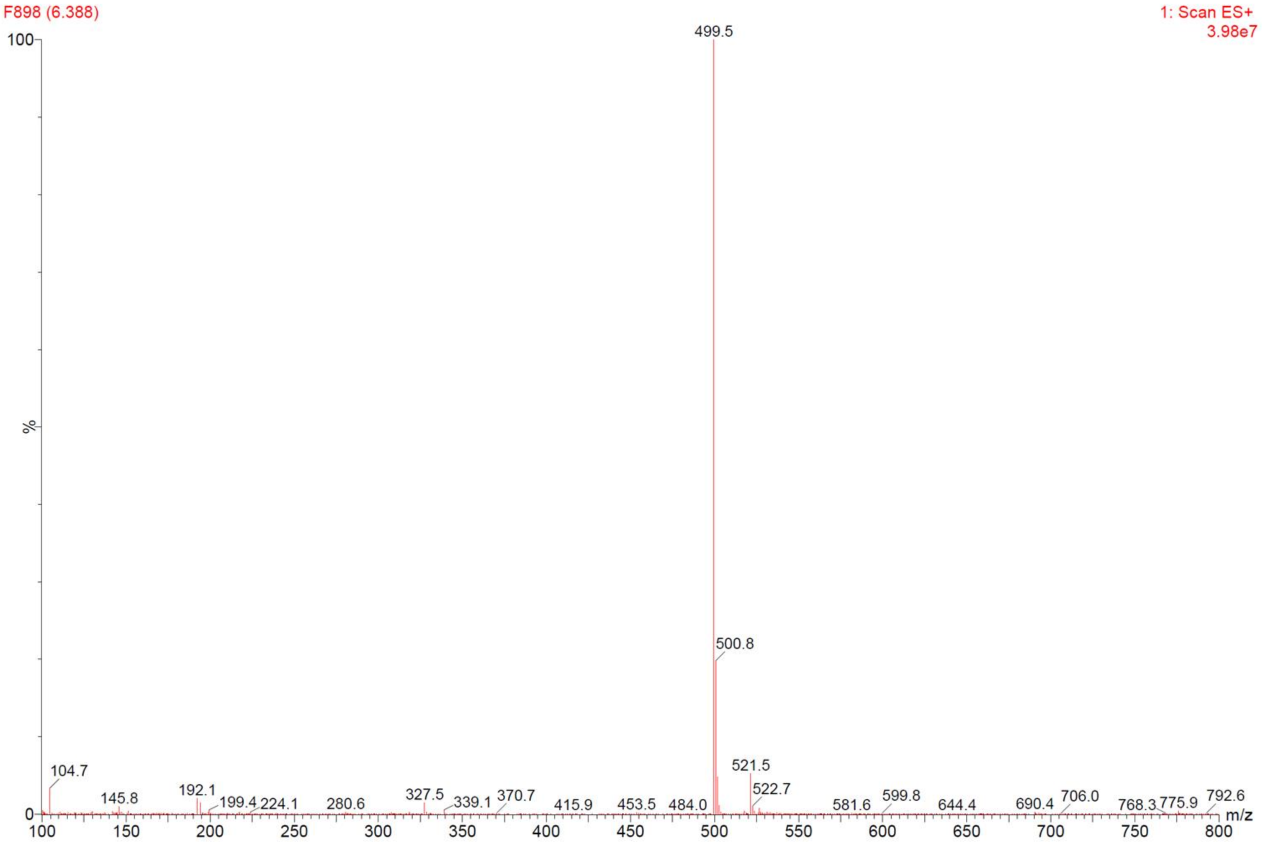

Mass spectrum of compound C172.

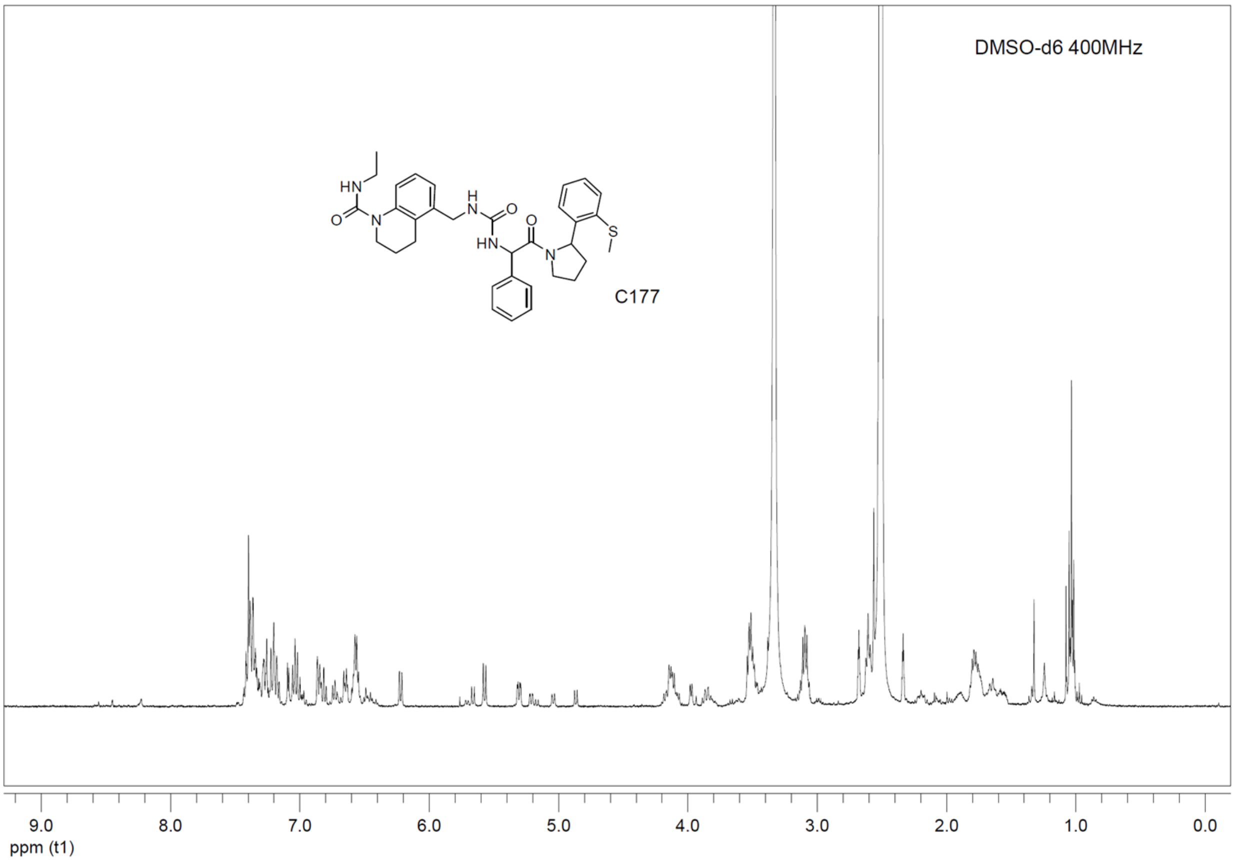

^{1}H NMR (400 MHz, DMSO-d6) spectrum of compound C177.

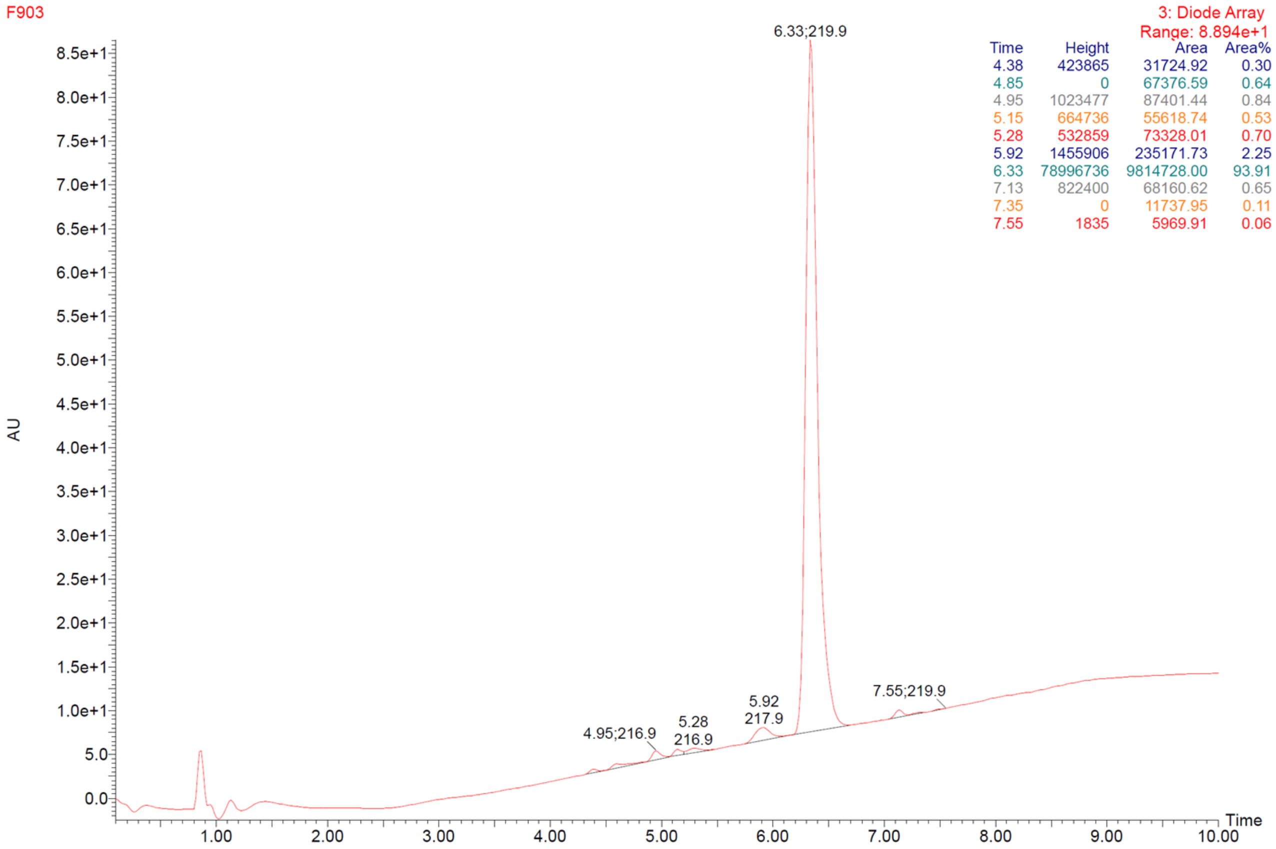

HPLC Chromatogram of compound C177.

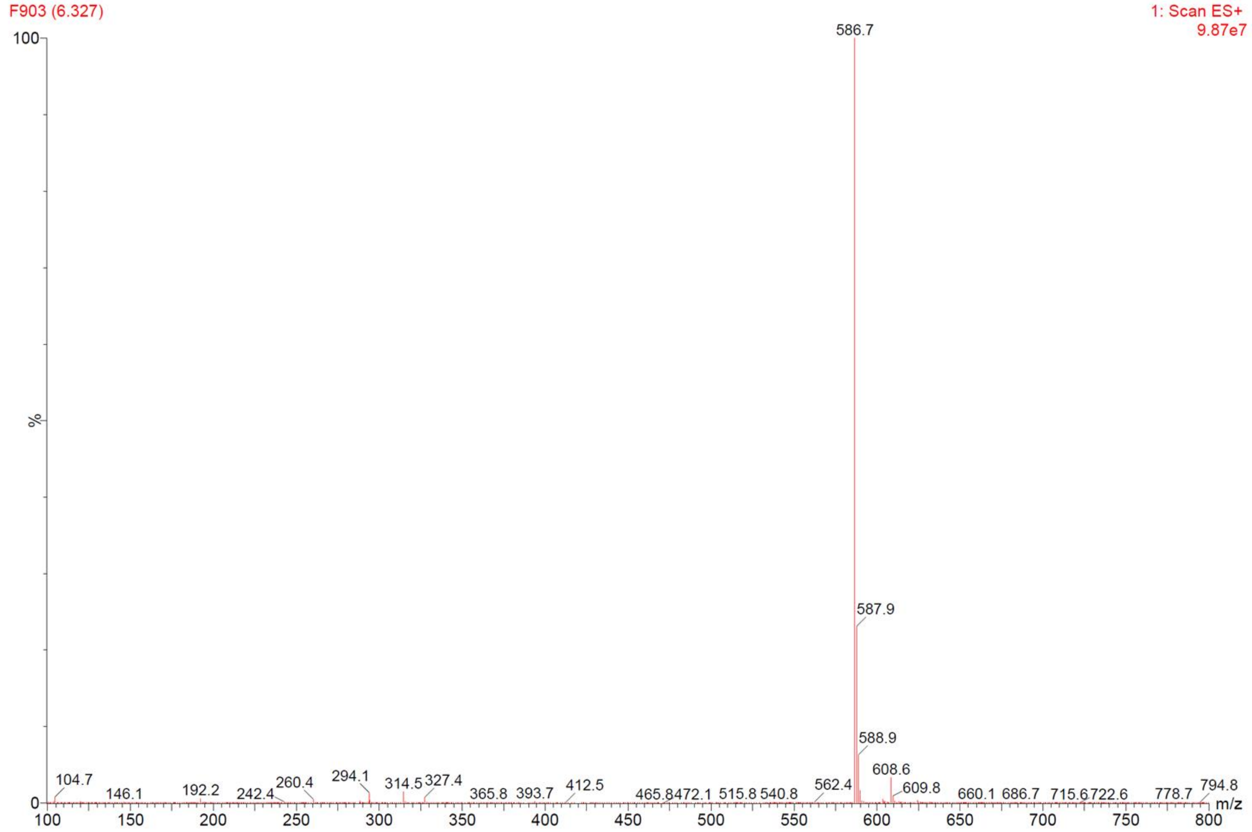

Mass spectrum of compound C177.

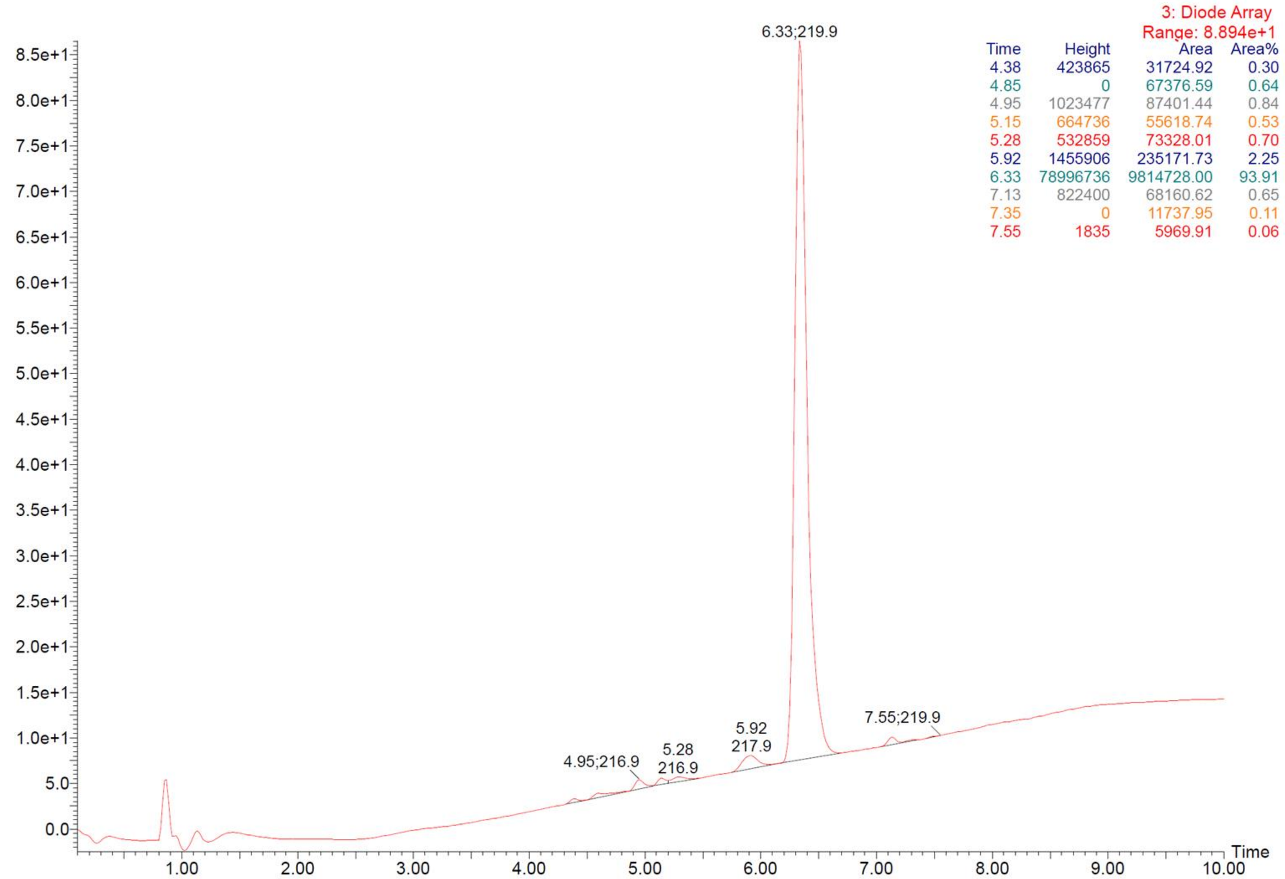

HPLC Chromatogram of compound C178.

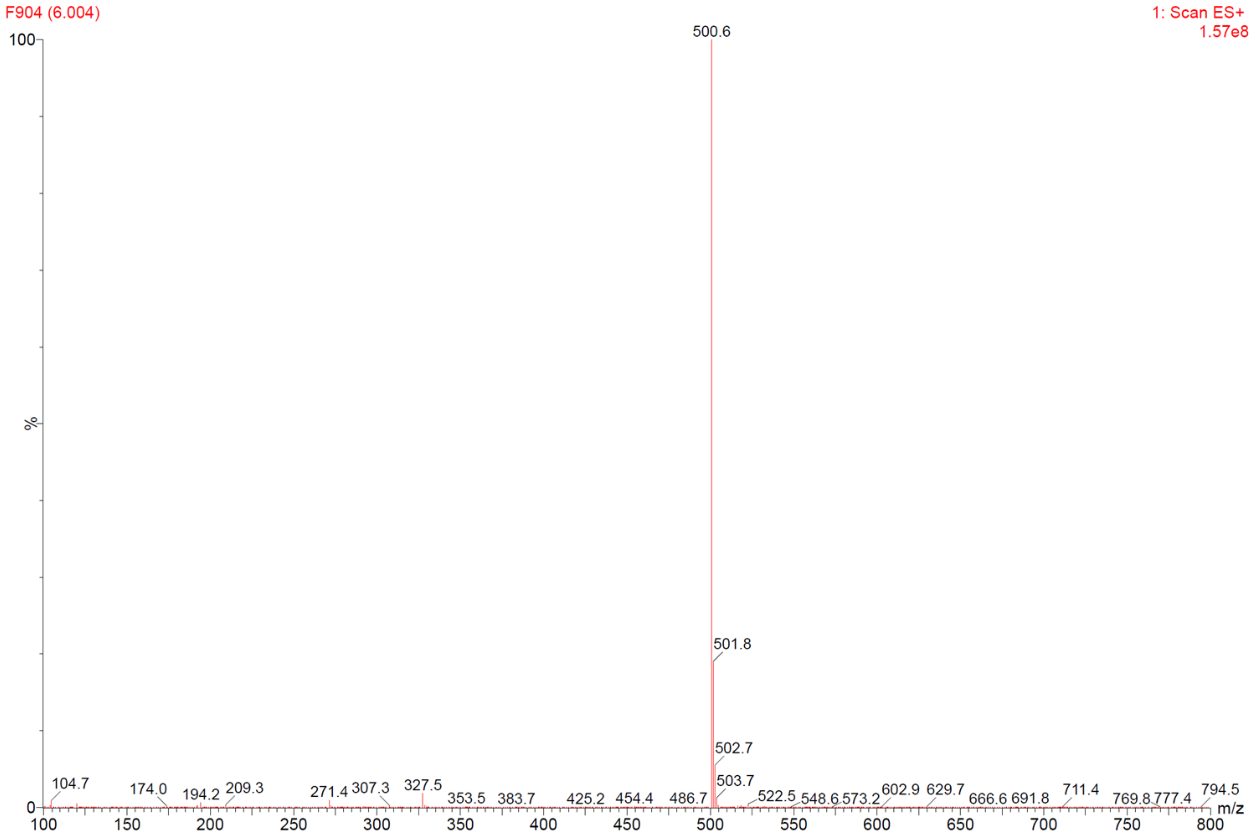

Mass spectrum of compound C178.

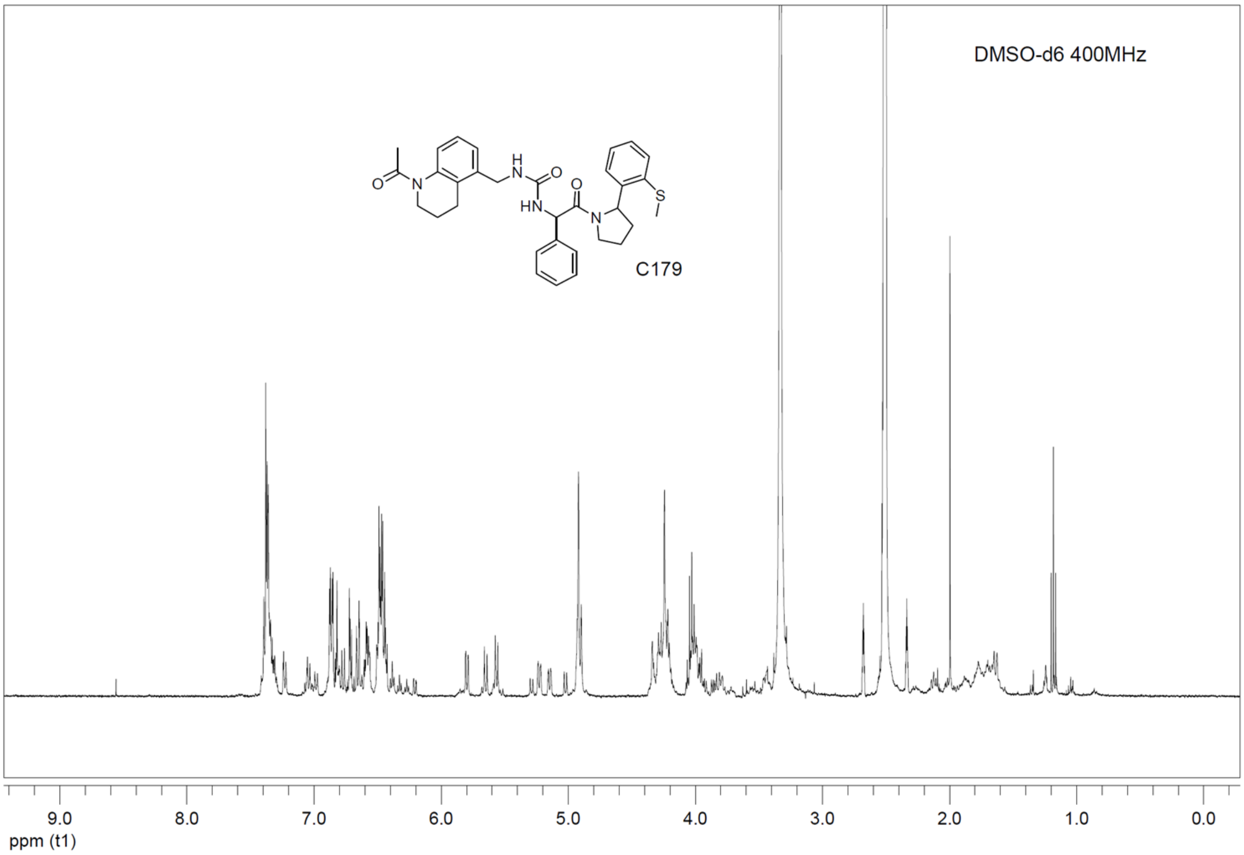

^{1}H NMR (400 MHz, DMSO-d6) spectrum of compound C179.

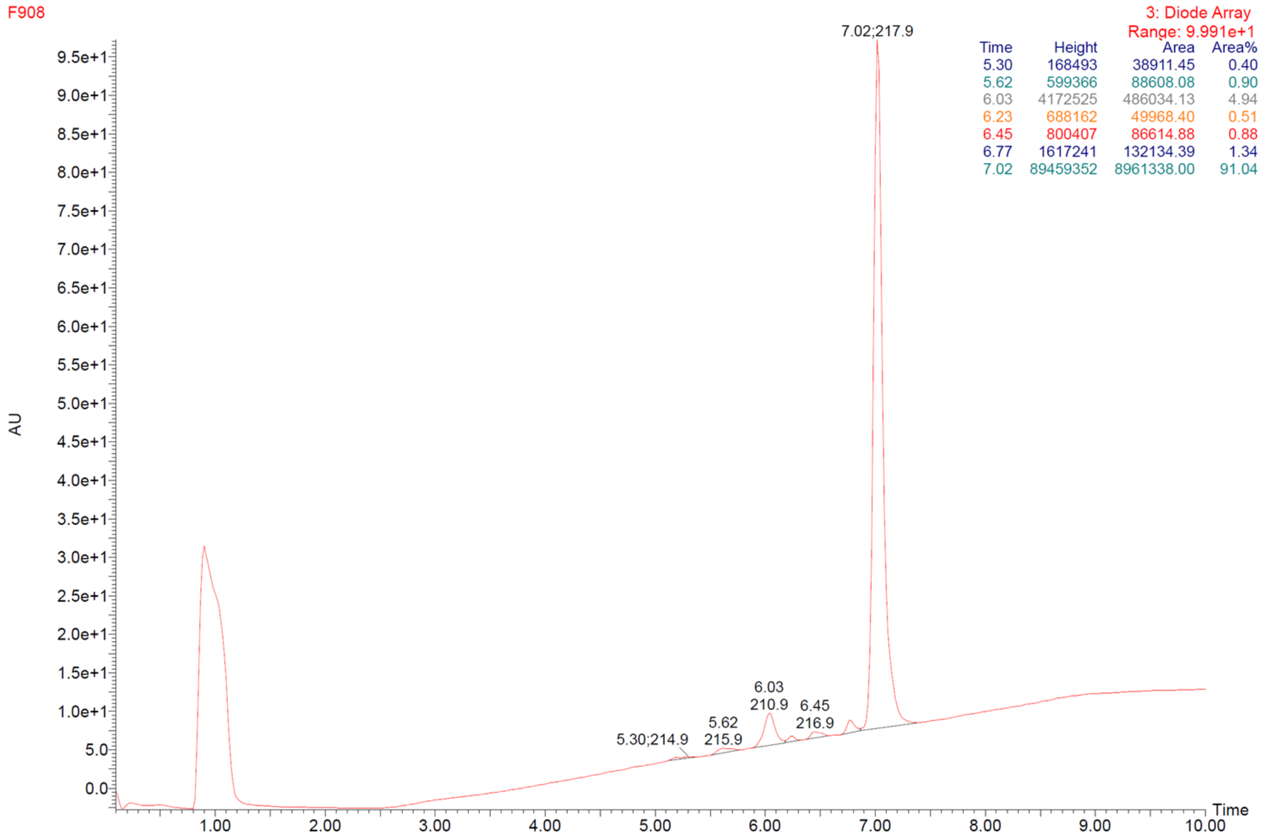

HPLC Chromatogram of compound C182.

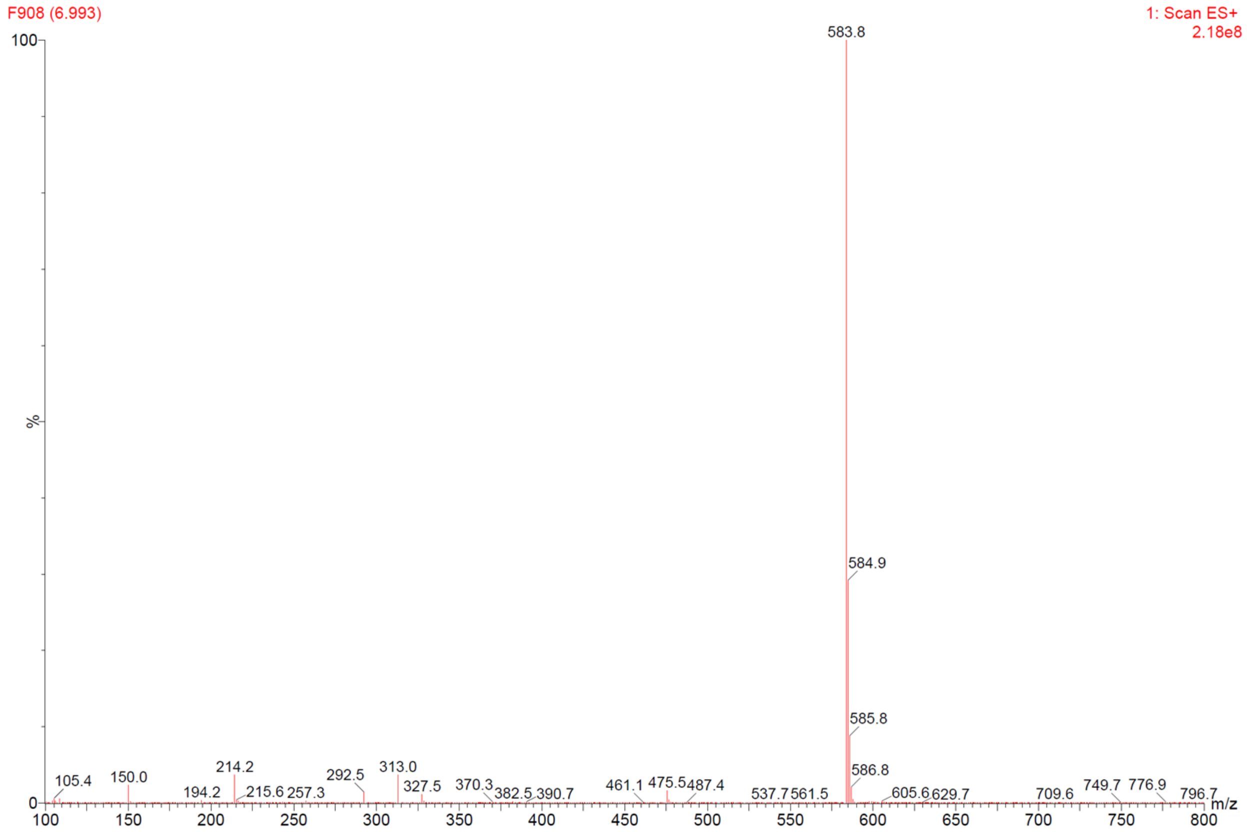

Mass spectrum of compound C182.

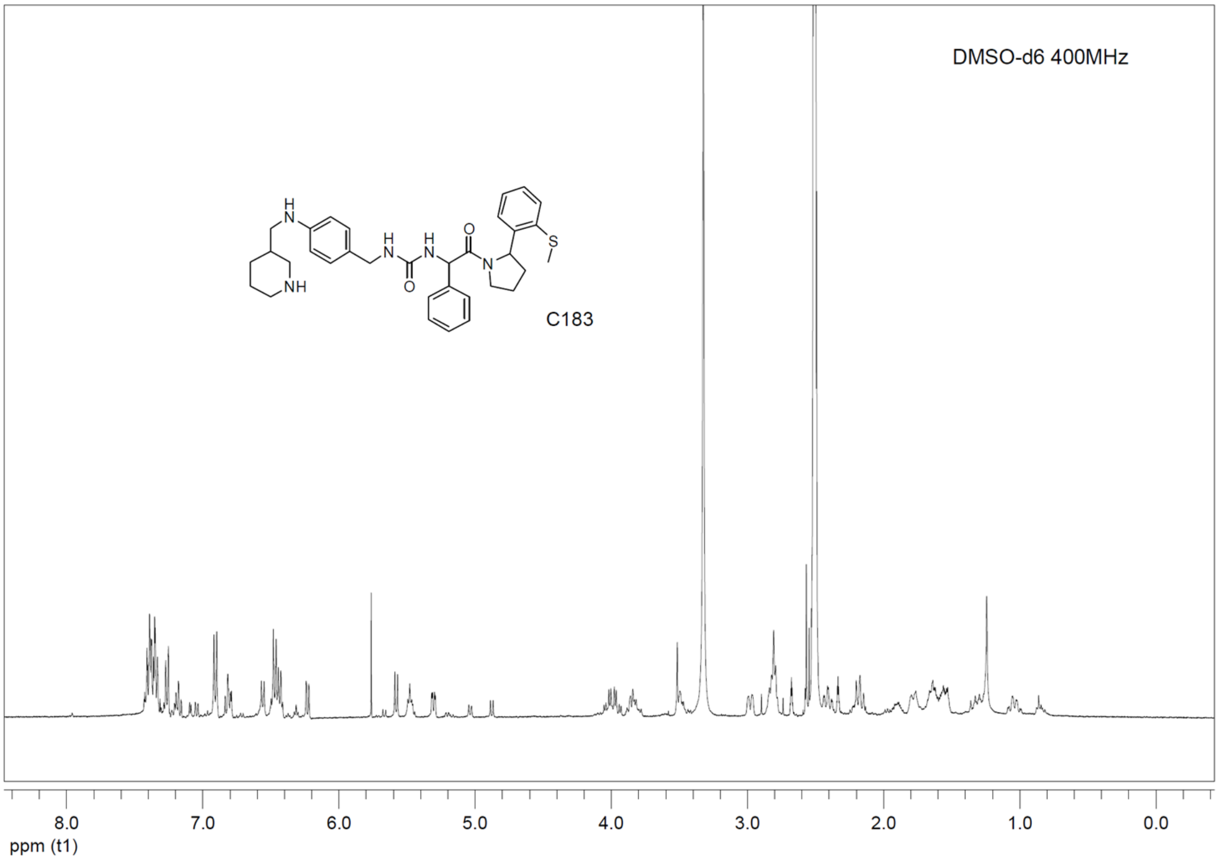

^{1}H NMR (400 MHz, DMSO-d6) spectrum of compound C183.

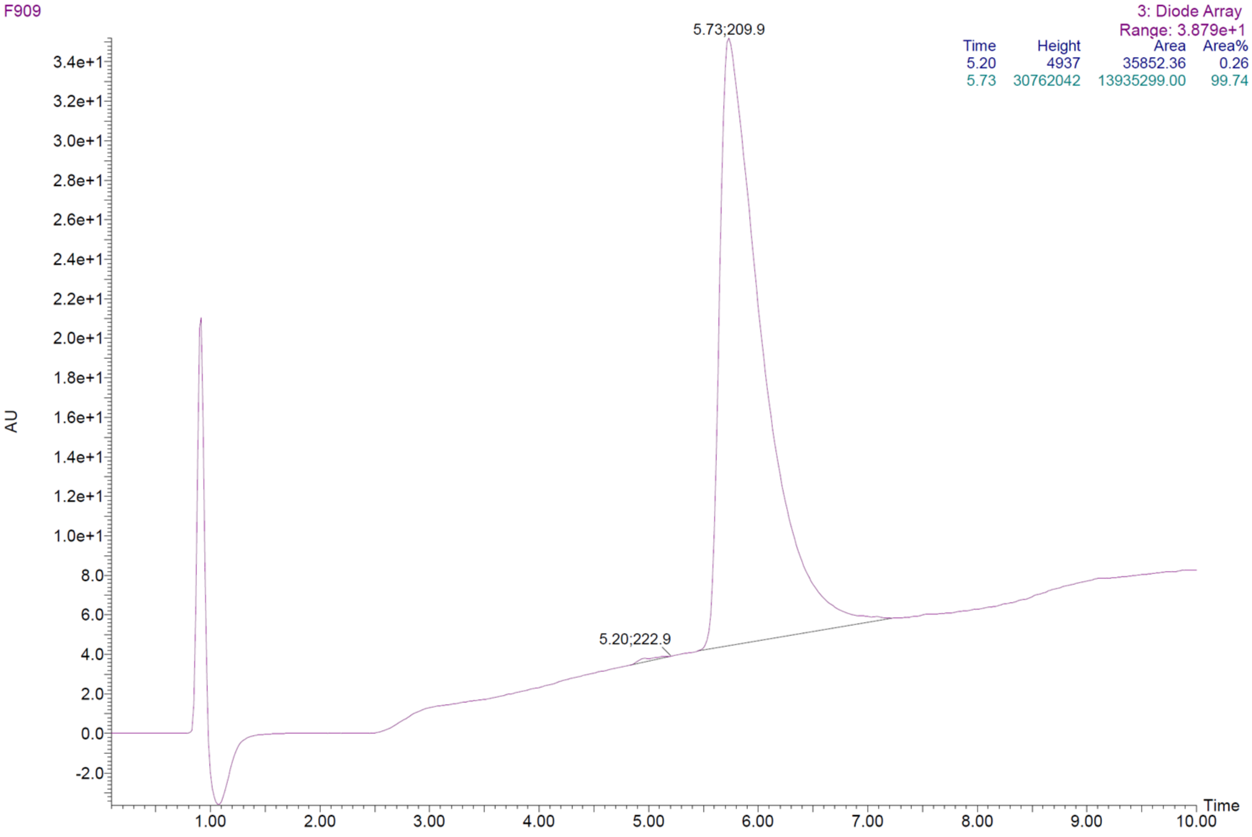

HPLC Chromatogram of compound C183.

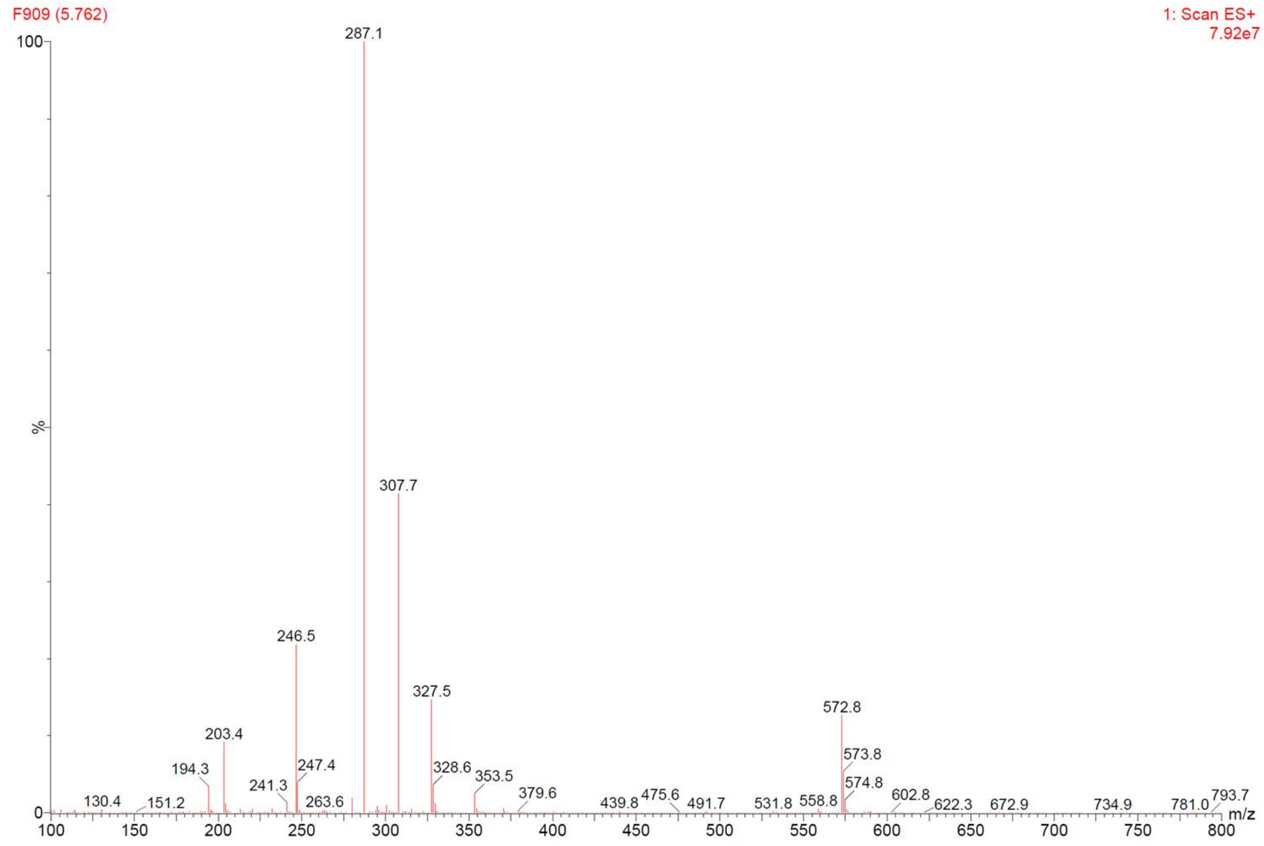

Mass spectrum of compound C183.

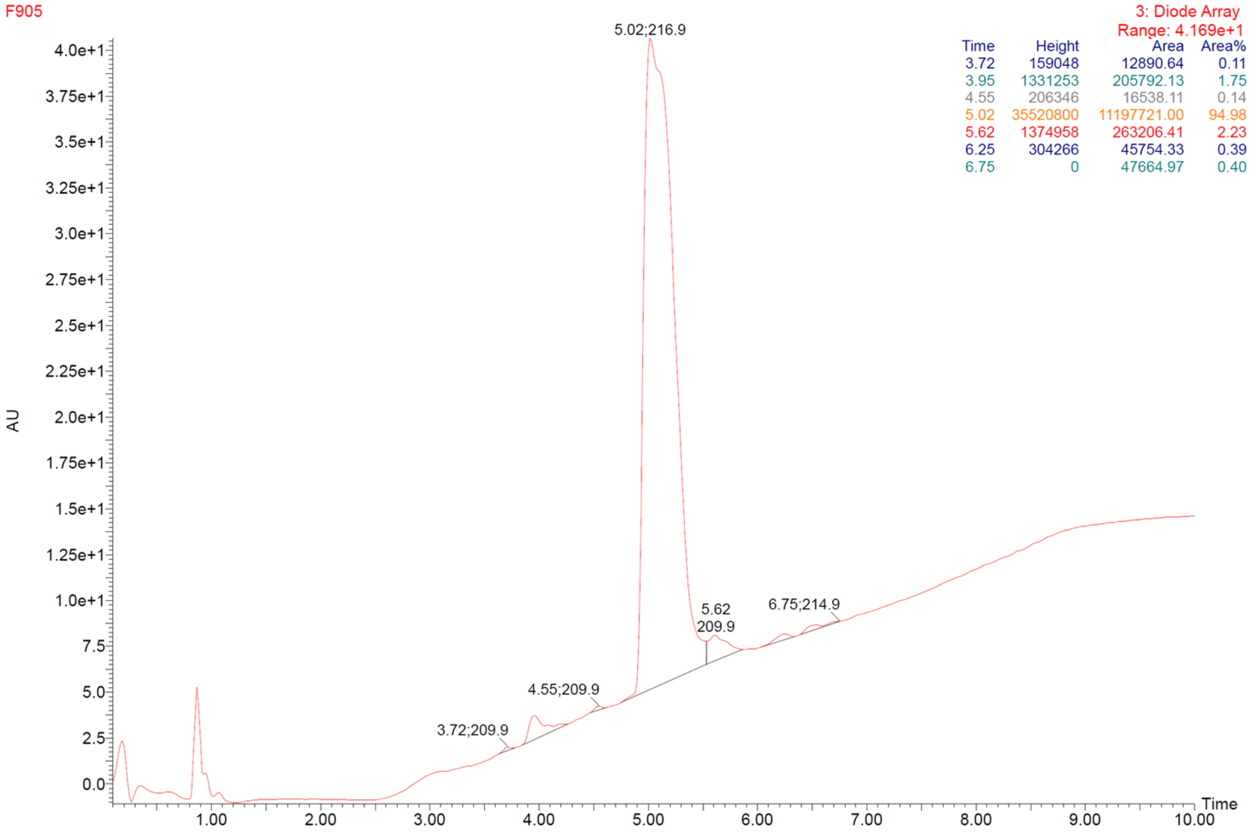

HPLC Chromatogram of compound C185.

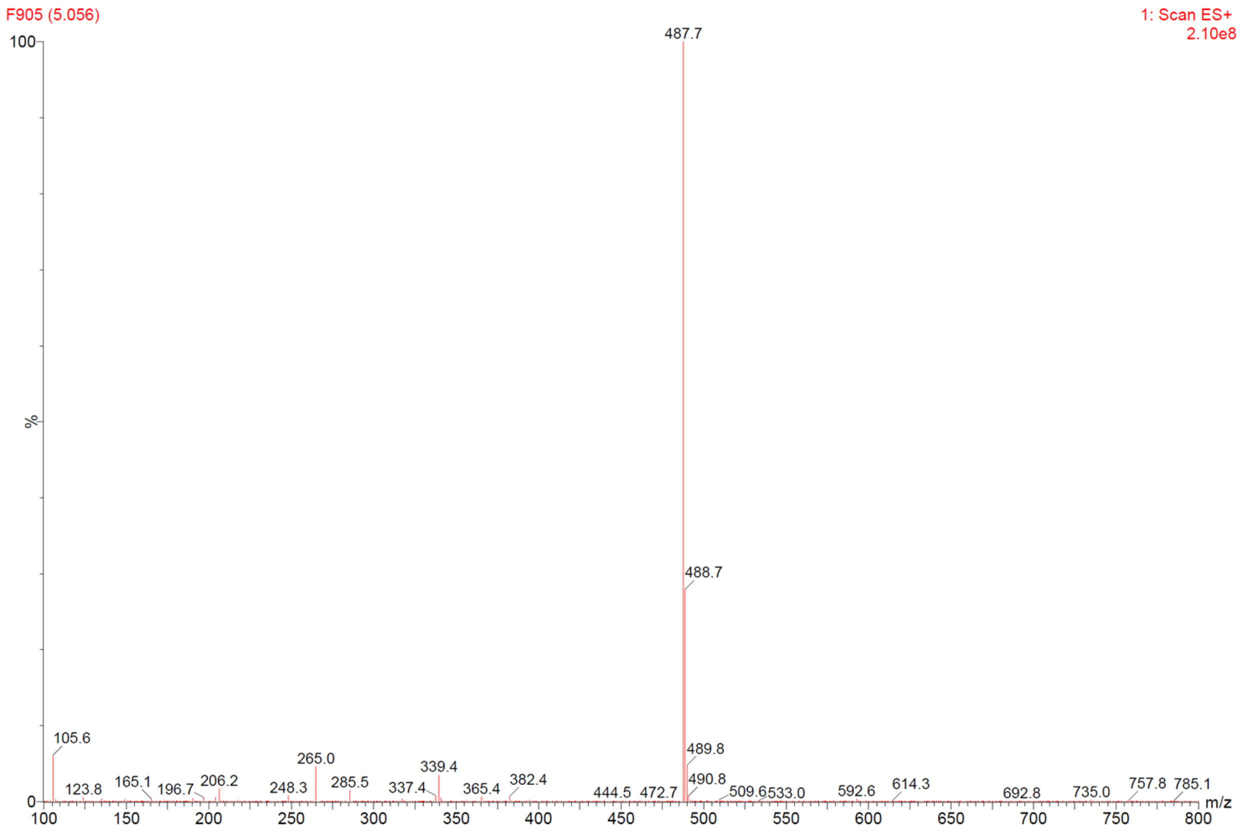

Mass spectrum of compound C185.

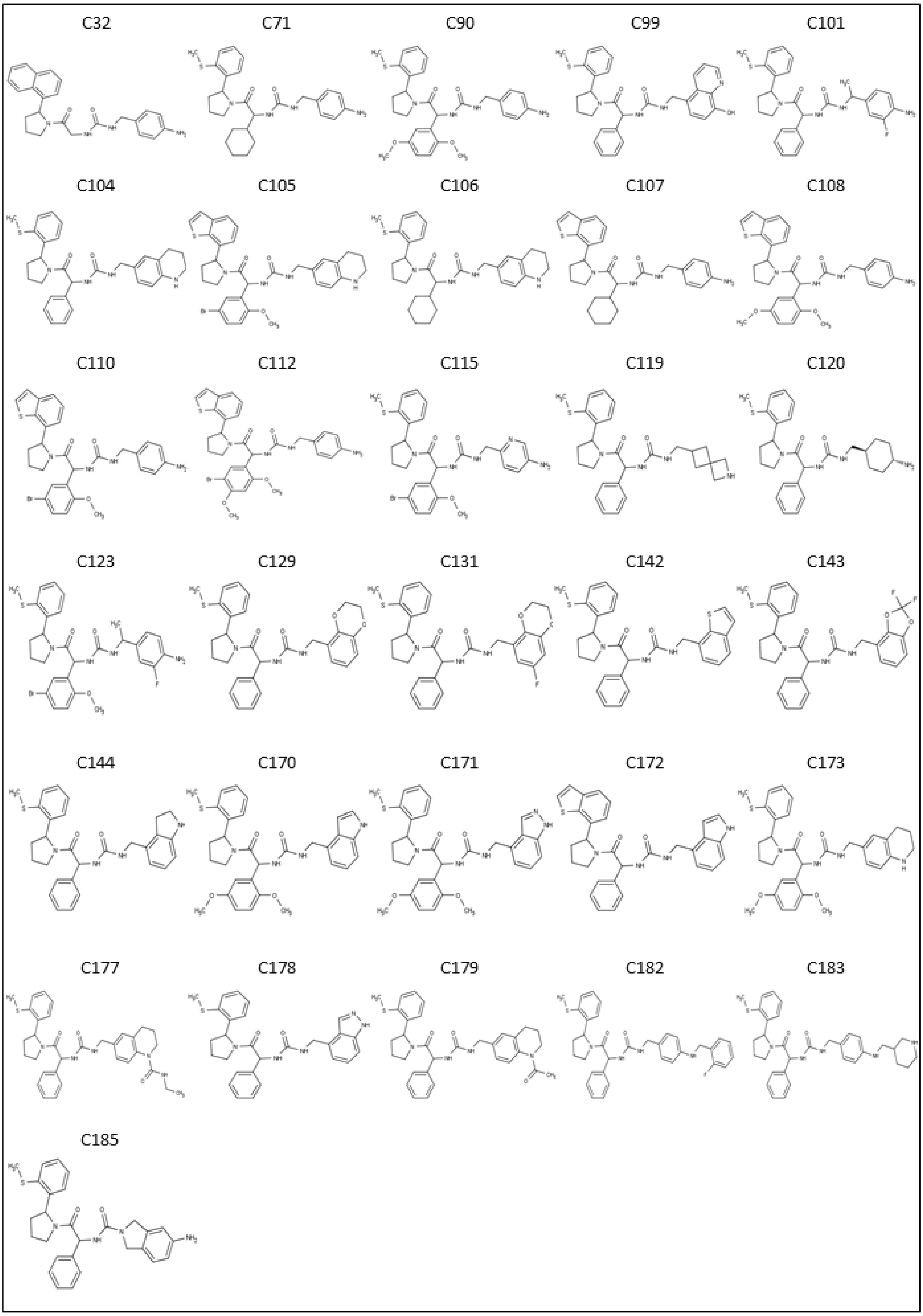

Supplemental Figure 1: Chemical structures of C31 derivatives used in this study.

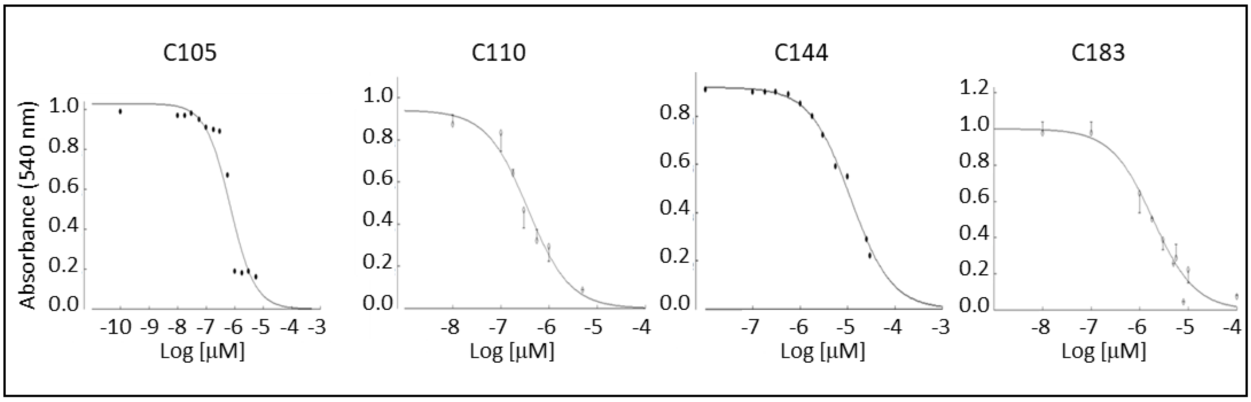

Supplemental Figure 2: Concentration-response curves of mitochondrial swelling inhibition by compounds C105, C110, C144 and C183.

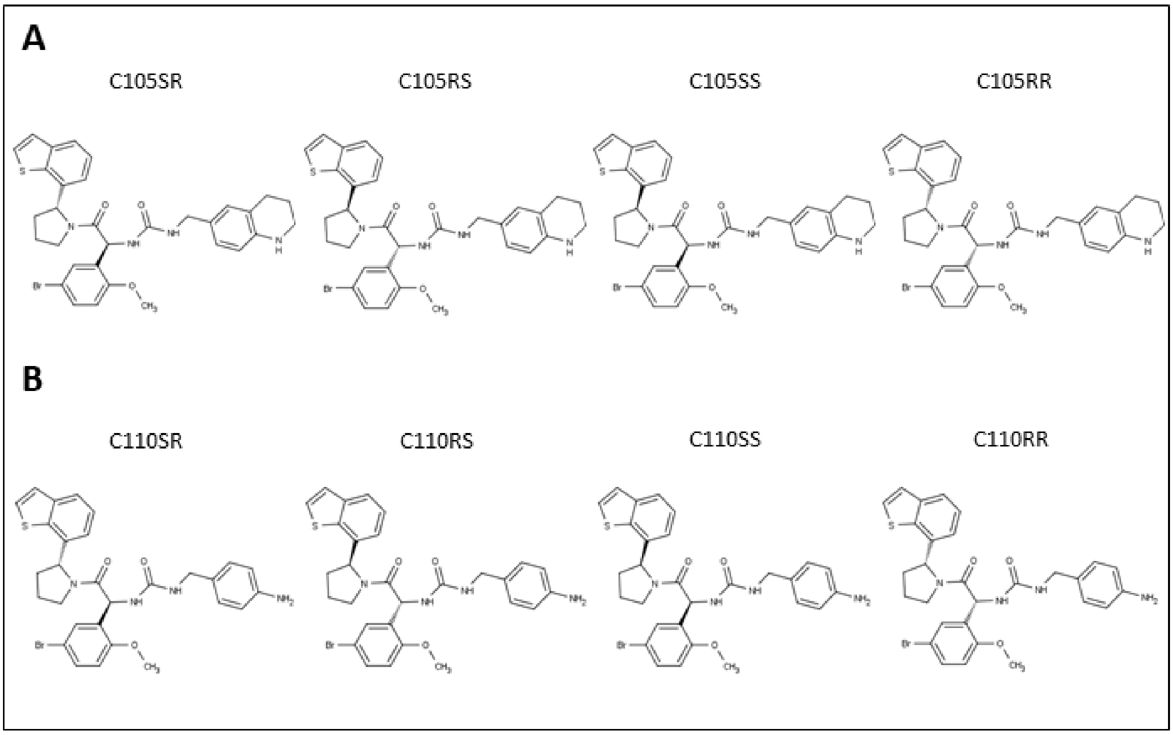

Supplemental Figure 3: Chemical structures of the four diastereoisomers of C105 (A) and C110 (B).

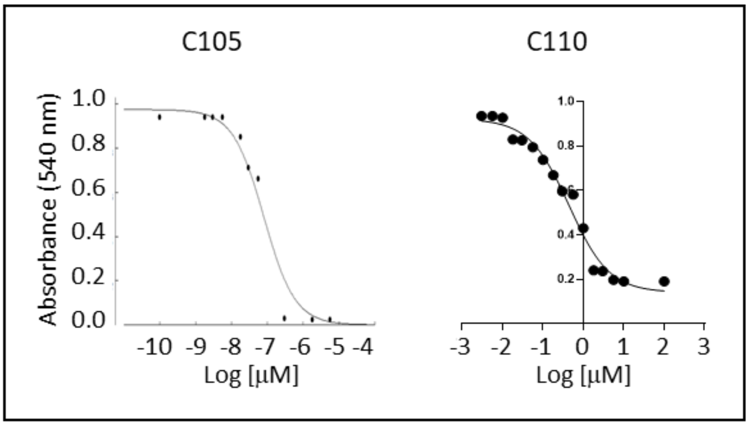

Supplemental Figure 4: Concentration-response curves of mitochondrial swelling inhibition by compounds C105SR and C110SR.

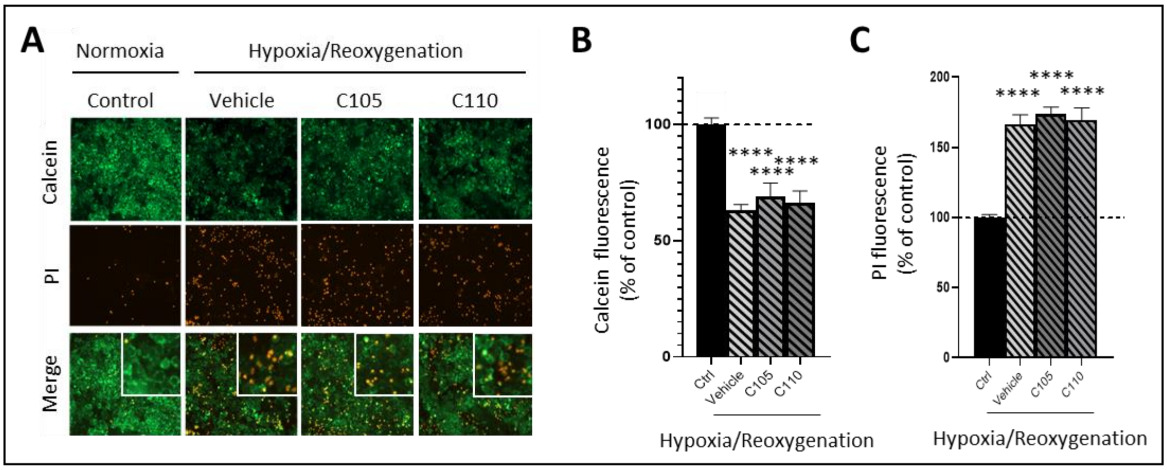

Supplemental Figure 5: Absence of inhibition of mPTP opening and reduction of necrosis by C105 and C110 at 1 µM *in vitro* in a model of hepatic hypoxia/reoxygenation. Cells were pretreated with 1 µM calcein and 1 mM CoCl2 for 30 min and 10 min, respectively, then subjected to 4 h of hypoxia (1% O2) followed by 1 h of reoxygenation (21% O2) in the presence of 3 µM propidium iodide (PI). C105 and C110 were added at 1 µM for the entire duration of hypoxia/reoxygenation. (A) Representative images of calcein (green) and PI (red) labeling in cells exposed to normoxia (control) or hypoxia/reoxygenation in the absence (vehicle) or in the presence of C105 or C110 (original magnification × 400). (B) Calcein fluorescence in cells exposed to normoxia (Ctrl) or hypoxia/reoxygenation in the absence (vehicle) or in the presence of C105 or C110. ****p < 0.0001 vs Ctrl. (C) PI fluorescence in cells exposed to normoxia (Ctrl) or hypoxia/reoxygenation in the absence (vehicle) or in the presence of C105 or C110. ****p < 0.0001 vs Ctrl.

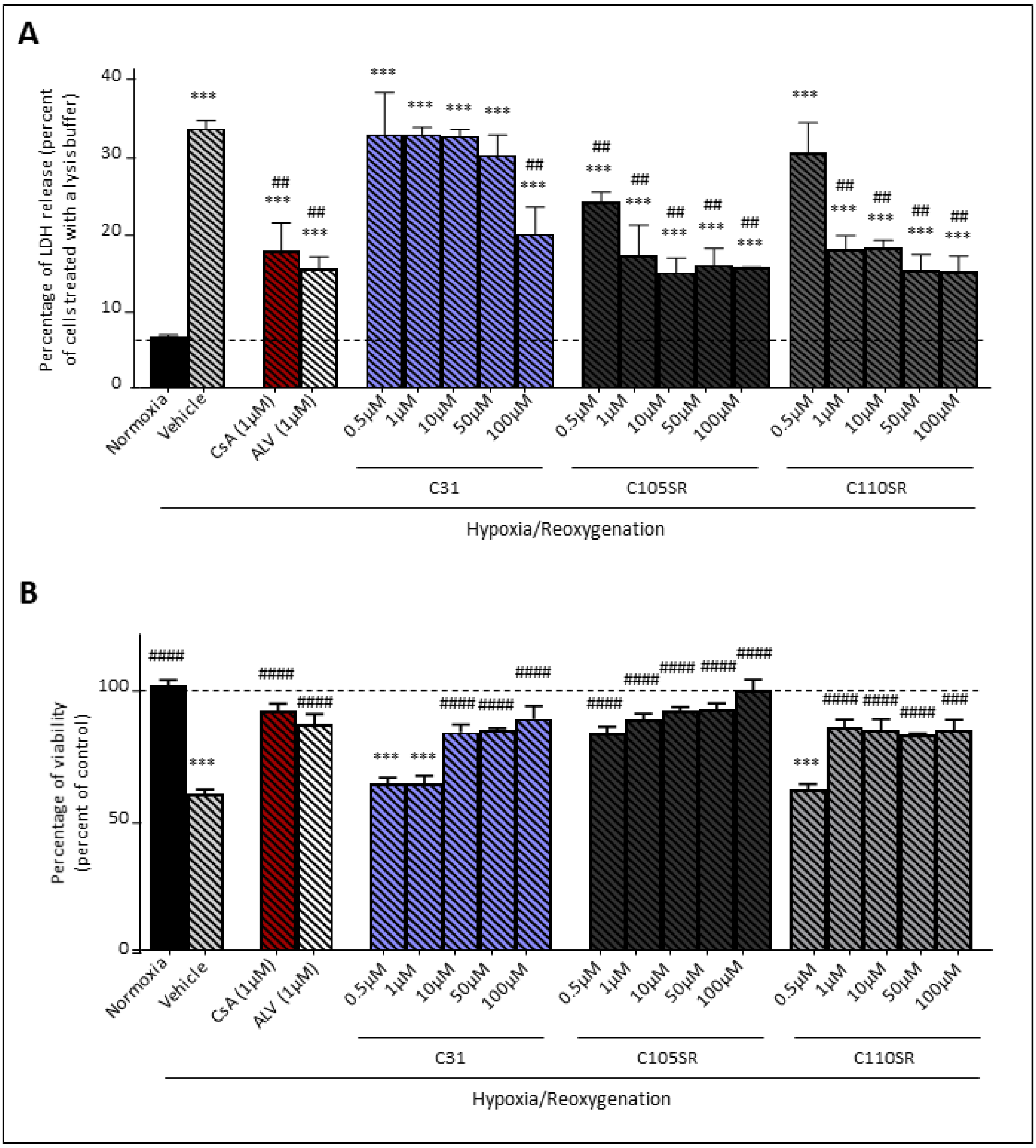

Supplemental Figure 6: Protection against cell death by pharmacological perconditioning with C105SR and C110SR. Cells were subjected to 4 h of hypoxia (1% O_2) followed by 2 h of reoxygenation (21% O_2). CsA and ALV were used as references. CsA and ALV were added at 1 µM while C31, C105SR and C110SR were added at increasing concentrations during the hypoxic phase. (A) LDH release from cells exposed to normoxia (Ctrl) or hypoxia/reoxygenation in the absence (vehicle) or in the presence of CsA, ALV or increasing concentrations of C31, C105SR or C110SR expressed as percentage of LDH release in cells treated with a lysis buffer (Ctrl). $^{***}p < 0.001$ vs Ctrl; $^{\#\#}p < 0.01$ vs hypoxia/reoxygenation vehicle. (B) Cell viability measured by MTT assay in cells exposed to normoxia (Ctrl) or hypoxia/reoxygenation in the absence (vehicle) or in the presence of CsA, ALV or increasing concentrations of C31, C105SR or C110SR expressed as percentage of control (Ctrl). $^{***}p < 0.001$ vs Ctrl ; $^{\#\#}p < 0.05$ vs hypoxia/reoxygenation vehicle.

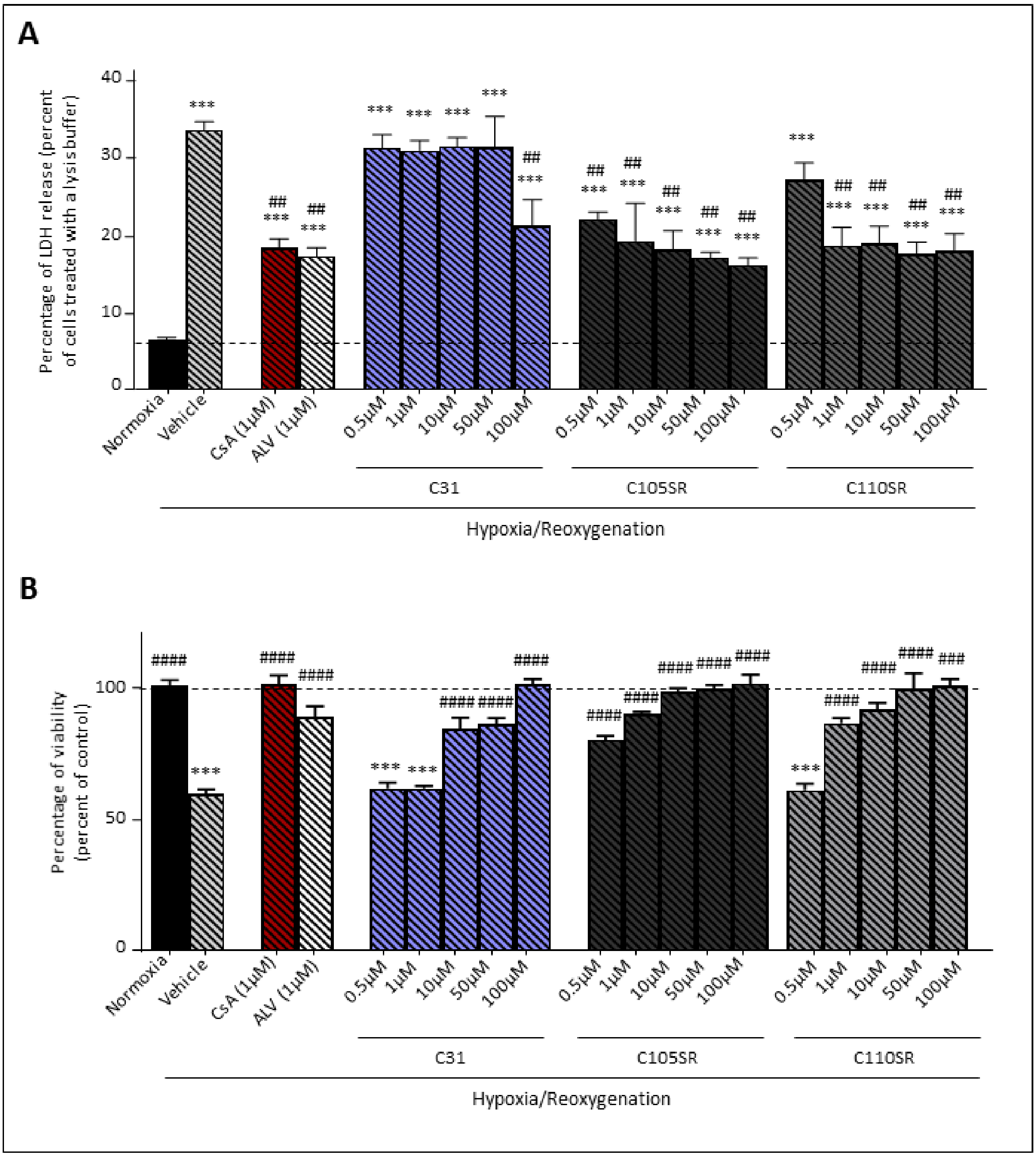

Supplemental Figure 7: Protection against cell death by pharmacological postconditioning with C105SR and C110SR. Cells were subjected to 4 h of hypoxia (1% O_2) followed by 2 h of reoxygenation (21% O_2). CsA and ALV were used as references. CsA and ALV were added at 1 µM while C31, C105SR or C110SR were added at increasing concentrations during the reoxygenation phase. (A) LDH release from cells exposed to normoxia or hypoxia/reoxygenation in the absence (vehicle) or in the presence of CsA, ALV or increasing concentrations of C31, C105SR or C110SR expressed as percentage of LDH release in control cells (Ctrl). ***p < 0.001 vs Ctrl; $^{\#\#}$p < 0.01 vs hypoxia/reoxygenation vehicle. (B) Cell viability measured by MTT assay in cells exposed to normoxia (Ctrl) or hypoxia/reoxygenation in the absence (vehicle) or in the presence of CsA, ALV or increasing concentrations of C31, C105SR or C110SR expressed as percentage of control (normoxia). ***p < 0.001 vs Ctrl ; $^{\#}$p < 0,05 vs hypoxia/reoxygenation vehicle.

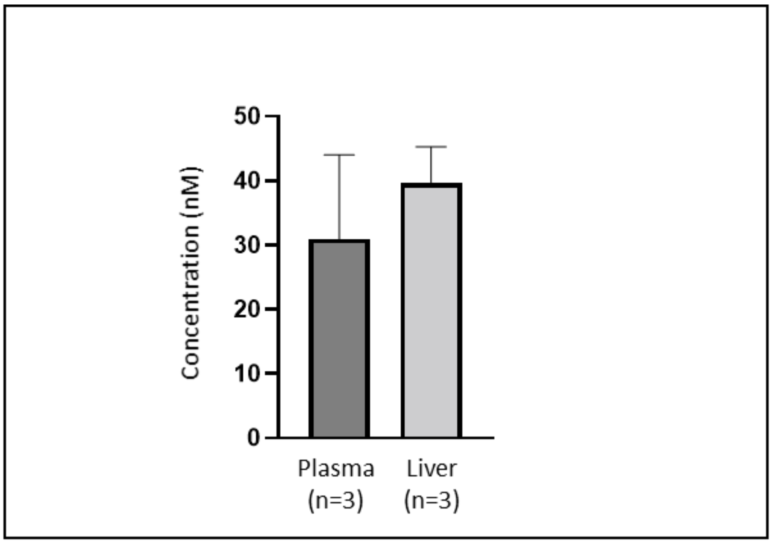

Supplemental Figure 8: Plasmatic and hepatic concentrations of C105SR 24 hours after osmotic pump implantation.
