# Supplementary material for: The novel cyclophilin inhibitor C105SR reduces hepatic ischaemia–reperfusion injury via mitoprotection
Source: JHEP Rep. 2023 Aug 16;5(11):100876. doi: 10.1016/j.jhepr.2023.100876 (PMC10582583; doi:10.1016/j.jhepr.2023.100876)
Supplement: Multimedia component 2 [file mmc2.pdf]

JOURNAL OF HEPATOLOGY

Journal of Hepatology

CTAT methods

Tables for a “Complete, Transparent, Accurate and Timely account” (CTAT) are now mandatory for all revised submissions. The aim is to enhance the reproducibility of methods.

- Only include the parts relevant to your study
- Refer to the CTAT in the main text as ‘Supplementary CTAT Table’
- Do not add subheadings
- Add as many rows as needed to include all information
- Only include one item per row

If the CTAT form is not relevant to your study, please outline the reasons why:

1.1 Antibodies

Name	Citation	Supplier	Cat no.	Clone no.

1.2 Cell lines

Name	Citation	Supplier	Cat no.	Passage no.	Authentication test method
AML-12		ATCC	CRL-2254	< 15	
HepaRG		Gibco	HPRGC10	< 15	

1.3 Organisms

Name	Citation	Supplier	Strain	Sex	Age	Overall n number

1.4 Sequence based reagents

Name	Sequence	Supplier

1.5 Biological samples

Description	Source	Identifier

1.6 Deposited data

Name of repository	Identifier	Link

1.7 Software

Software name	Manufacturer	Version

1.8 Other (e.g. drugs, proteins, vectors etc.)

Calcein-AM	Invitrogen	C3100MP
Calcium Green™-5N	Invitrogen	C3737
Cyclosporin A	Sigma-Aldrich	30024
Alisporivir	Gift from Biopharma	
Propidium iodide	Sigma-Aldrich	P4864
Caspase-Glo® 3/7 assay	Promega	G8091
Alzet® osmotic pump	Charles River	10104845
TUNEL	Abcam	ab206386

1.9 Please provide the details of the corresponding methods author for the manuscript:

TEIXEIRA-CLERC Fatima
INSERM U955 – Equipe Pawlotsky
Hôpital Henri Mondor
1 rue Gustave Eiffel
94010 Créteil
Tel: +33 1 49 81 35 37
Fax: + 33 1 49 81 35 33
Email: fatima.clerc@inserm.fr

2.0 Please confirm for randomised controlled trials all versions of the clinical protocol are included in the submission. These will be published online as supplementary information.
