# Supplementary material for: The novel cyclophilin inhibitor C105SR reduces hepatic ischaemia–reperfusion injury via mitoprotection
Source: JHEP Rep. 2023 Aug 16;5(11):100876. doi: 10.1016/j.jhepr.2023.100876 (PMC10582583; doi:10.1016/j.jhepr.2023.100876)
Supplement: Multimedia component 4 [file mmc4.pdf]

# The novel cyclophilin inhibitor C105SR reduces hepatic ischaemia–reperfusion injury via mitoprotection

Amel Kheyar,[1] Nazim Ahnou,[1] Abdelhakim Ahmed-Belkacem,[1] Anne Hulin,[2,3] Claire Pressiat,[2,3] Bijan Ghaleh,[3] Jean-François Guichou,[4] Didier Morin,[3] Jean-Michel Pawlotsky,[1,5] Fatima Teixeira-Clerc[1,*]

[1]*Équipe "Virus, Hépatologie, Cancer", INSERM U955, IMRB, Université Paris-Est, Créteil, France;* [2]*Laboratoire de Pharmacologie, DMU de Biologie et Pathologie, Hôpitaux Universitaires Henri Mondor, AP-HP, Créteil, France;* [3]*Équipe "Pharmacologie et Technologies pour les Maladies Cardiovasculaires", INSERM U955, IMRB, Université Paris-Est, Créteil, France;* [4]*Centre de Biologie Structurale (CBS), Université de Montpellier, CNRS, INSERM, Montpellier, France;* [5]*Département Prévention, Diagnostic et Traitement des Infections, DMU de Biologie et Pathologie, Hôpitaux Universitaires Henri Mondor, AP-HP, Créteil, France*

JHEP Reports **2023.** https://doi.org/10.1016/j.jhepr.2023.100876

**Background & Aims:** Mitochondrial permeability transition pore (mPTP) opening is critical for mediating cell death during hepatic ischaemia–reperfusion injury (IRI). Blocking mPTP opening by inhibiting cyclophilin D (CypD) is a promising pharmacological approach for the treatment of IRI. Here, we show that diastereoisomers of a new class of small-molecule cyclophilin inhibitors (SMCypIs) have properties that make them attractive candidates for the development of therapeutic agents against liver IRI.
**Methods:** Derivatives of the parent SMCypI were synthesised and evaluated for their ability to inhibit CypD peptidyl-prolyl *cis-trans* isomerase (PPIase) activity and for their mitoprotective properties, evaluated by measuring mitochondrial swelling and calcium retention capacity in liver mitochondria. The ability of the selected compounds to inhibit mPTP opening was evaluated in cells subjected to hypoxia/reoxygenation using a calcein/cobalt assay. Their ability to inhibit cell death was evaluated in cells subjected to hypoxia/reoxygenation by measuring lactate dehydrogenase (LDH) release, propidium iodide staining, and cell viability. The compound performing best *in vitro* was selected for *in vivo* efficacy evaluation in a mouse model of hepatic IRI.
**Results:** The two compounds that showed the strongest inhibition of CypD PPIase activity and mPTP opening, C105 and C110, were selected. Their SR diastereoisomers carried the activity of the racemic mixture and exhibited mitoprotective properties superior to those of the known macrocyclic cyclophilin inhibitors cyclosporin A and alisporivir. C105SR was more potent than C110SR in inhibiting mPTP opening and prevented cell death in a model of hypoxia/reoxygenation. Finally, C105SR substantially protected against hepatic IRI *in vivo* by reducing hepatocyte necrosis and apoptosis.
**Conclusions:** We identified a novel cyclophilin inhibitor with strong mitoprotective properties both *in vitro* and *in vivo* that represents a promising candidate for cellular protection in hepatic IRI.
**Impact and Implications:** Hepatic ischaemia–reperfusion injury (IRI) is one of the main causes of morbidity and mortality during or after liver surgery. However, no effective therapies are available to prevent or treat this devastating syndrome. An attractive strategy to prevent hepatic IRI aims at reducing cell death by targeting mitochondrial permeability transition pore opening, a phenomenon regulated by cyclophilin D. Here, we identified a new small-molecule cyclophilin inhibitor, and demonstrated the enhanced mitoprotective and hepatoprotective properties of one of its diastereoisomers both *in vitro* and *in vivo*, making it an attractive lead compound for subsequent clinical development.
© 2023 The Authors. Published by Elsevier B.V. on behalf of European Association for the Study of the Liver (EASL). This is an open access article under the CC BY license (http://creativecommons.org/licenses/by/4.0/).

## Introduction

Hepatic ischaemia–reperfusion injury (IRI) is a severe complication of various clinical conditions, including haemorrhagic shock, liver resection, and liver transplantation. Hepatic IRI is a leading cause of early allograft dysfunction and a major risk factor for acute and chronic rejection, particularly when liver grafts with expanded criteria are used, including those from marginal, deceased, and non-beating heart donors.[1–3] Hepatic IRI can lead to multi-organ dysfunction or systemic inflammatory response syndrome, both of which carry high risks of mortality. No specific treatment is available to reduce hepatic IRI, and current management is based on supportive care.

IRI occurs as the result of a biphasic phenomenon in which cellular damage caused by hypoxia is paradoxically exacerbated by the restoration of oxygen delivery. Mitochondrial dysfunction plays an important role in the pathogenesis of IRI.[4] In particular,

Keywords: Cellular protection; Peptidyl-prolyl *cis-trans* isomerase activity; Liver necrosis; Mitochondrial calcium retention capacity; Mitochondrial permeability transition pore; Mitochondrial swelling.
*Received 24 February 2023; received in revised form 7 July 2023; accepted 27 July 2023; available online 16 August 2023*
* Corresponding author. Address: INSERM U955, Institut Mondor de Recherche Biomédicale, Hôpital Henri Mondor, 1 rue Gustave Eiffel, 94010 Créteil, France. Tel: +33 1-49-81-35-37; Fax: +33 1-48-98-09-08.
*E-mail address:* fatima.clerc@inserm.fr (F. Teixeira-Clerc).

mitochondrial permeability transition (mPT) is thought to be a critical mediator of the damage that accompanies reperfusion of organs following prolonged ischaemia. mPT is defined as a sudden increase in the permeability of the inner mitochondrial membrane to solutes with a molecular mass less than 1.5 kDa. mPT is mediated by the opening of the mPT pore (mPTP), a high-conductance channel involved in $Ca^{2+}$ homeostasis. Persistent mPTP opening during IRI results in loss of membrane potential, uncoupling of oxidative phosphorylation, ATP depletion, and mitochondrial swelling, ultimately leading to necrotic cell death. In addition, mPTP releases cytochrome C from mitochondria, which in turn triggers apoptosis. Although the molecular composition of the mPTP is still debated, it is recognised that mPTP opening is regulated by cyclophilin D (CypD).[5,6]

CypD is a member of a family of highly homologous peptidyl-prolyl *cis-trans* isomerases (PPIases) that catalyse the interconversion of two energetically preferred conformers (*cis* and *trans*) of the planar peptide bond preceding an internal proline residue. Cyclophilins carry chaperone activity that regulates protein folding. The PPIase domain catalyses cyclophilin isomerase activity. It contains two binding pockets: the catalytic site of PPIase (the S1 pocket) and the so-called "gatekeeper pocket" (the S2 pocket), whose functional role remains unknown.[7] Cyclophilins share a common cyclophilin-like domain (CLD) and a domain of approximately 109 amino acids, flanked by domains specific for each cyclophilin responsible for its subcellular compartmentalisation and functional specialisation. CypD is located in the mitochondrial matrix and acts as a key regulator of mPTP opening.[5,6] Persistent CypD-dependent mPTP opening induces necrotic cell death, which plays a key role in IRI.[8] Conversely, CypD-deficient cells are highly resistant to cell death induced by cytosolic calcium overload and hydrogen peroxide-mediated stress induced by reactive oxygen species.[9] Thus, inhibition of mPTP opening by compounds that target CypD represents an attractive strategy for cellular protection in the context of hepatic IRI.

Cyclosporin A (CsA) and sanglifehrin A (SfA) are two structurally distinct natural Cyp inhibitors that potently inhibit mPTP opening through CypD inhibition, but their immunosuppressive properties limit their therapeutic use as Cyp inhibitors.[10,11] Non-immunosuppressive derivatives of these molecules have been generated by chemical modifications. Among them, NIM811, a CsA analogue, can inhibit mPTP opening and protect against cell death after liver transplantation.[12,13] Alisporivir (Debio-025, ALV), another CsA analogue, was developed for the treatment of hepatitis C virus infection, but its clinical development was halted due to adverse events unrelated to Cyp inhibition.[14] Other CsA or SfA derivatives have been proposed that have potential utility for the treatment of liver diseases.[15,16] However, no compound targeting cyclophilins has been clinically approved to date. Thus, novel potent cyclophilin inhibitors are required to make mPTP opening inhibition credible as a hepatoprotective strategy.

We have recently developed a new family of non-peptidic, small-molecule cyclophilin inhibitors (SMCypIs), which is chemically distinct from all currently known cyclophilin inhibitors.[17] We show that one of the SMCypIs derivatives, compound C31, exerts mitoprotective effects *in vitro* and protects cells in an *in vivo* murine model of liver IRI.[18] Here, we chemically improved C31, identified a new lead SMCypI compound, and demonstrated the enhanced mitoprotective and hepatoprotective properties of one of its diastereoisomers both *in vitro* and *in vivo*, making it an attractive lead compound for subsequent clinical development.

## Materials and methods

### Drugs

Unless otherwise mentioned, all reagents were purchased from Sigma-Aldrich. Calcein-AM and Calcium green 5N were purchased from Invitrogen. DMSO was used as the vehicle control of cyclophilin inhibitors for all *in vitro* and *in vivo* experiments, at the same dilutions as used in making cyclophilin inhibitor working solutions.

### SMCypI synthesis

Chemical reagents were obtained from ThermoFisher, Acros Organics, Spirochem, ACB Blocks, and Enamine, and were used without further purification. Compounds C31 and C32 were synthesised as previously described.[15] The synthesis of the other compounds is detailed in Supporting Materials and Methods.

### Animals

Male C57BL/6J mice (8–12 weeks-old, n = 45) were purchased from Janvier (Le Genest-St-Isle, France). All animals were housed in an air-conditioned room with nycthemeral rhythm (12-h light/dark cycles) and were granted free access to water and standard rodent chow. All animal procedures in this study were conducted in accordance with the directives of the European Parliament (2010/63/EU-848 EEC) and approved by the Animal Ethics Committee ANSES/ENVA/Université Paris-Est-Créteil.

### Hepatic IRI *in vivo* model

Mice (10–12 weeks-old) were anesthetised with isoflurane and subcutaneously implanted with Alzet® osmotic pump containing C105SR (50 mg/kg) or vehicle 24 h before the surgical procedure. The mice were anesthetised with isoflurane and subjected to partial hepatic ischaemia by clamping the hepatic artery and portal vein for 60 min, followed by 6 h of reperfusion induced by removal of the clamp. The sham-operated group underwent laparotomy without vascular occlusion.

### Statistical analysis

All results are expressed as mean ± standard error of the mean (SEM) from at least 3 independent experiments. Statistical analyses were performed with GraphPad Prism v.9.1.2 Software using the Mann-Whitney *U* test or the one-way ANOVA analysis followed by a Tukey's or a Dunnett's post-test if ANOVA produced a significant value of F ($p$ <0.05). Differences were considered significant when $p$ <0.05.

## Results

### Chemical optimisation and validation of novel CypD inhibitors with enhanced mitoprotective properties

To improve the potency of our previously reported SMCypI C31, chemical modifications of its three functional regions, including R1 that binds to the S1 pocket, R2 that binds to the S2 pocket, and R3 that interacts with residues between the two pockets (Fig. 1A and B), were performed. A library of compounds was generated in which: (i) the phenyl-pyrolidine group (R1 position) was modified to remove the thio-methyl group using benzothiophene ring; (ii) the aniline group (R2 position) was modified to remove the aniline motif using, for example, different

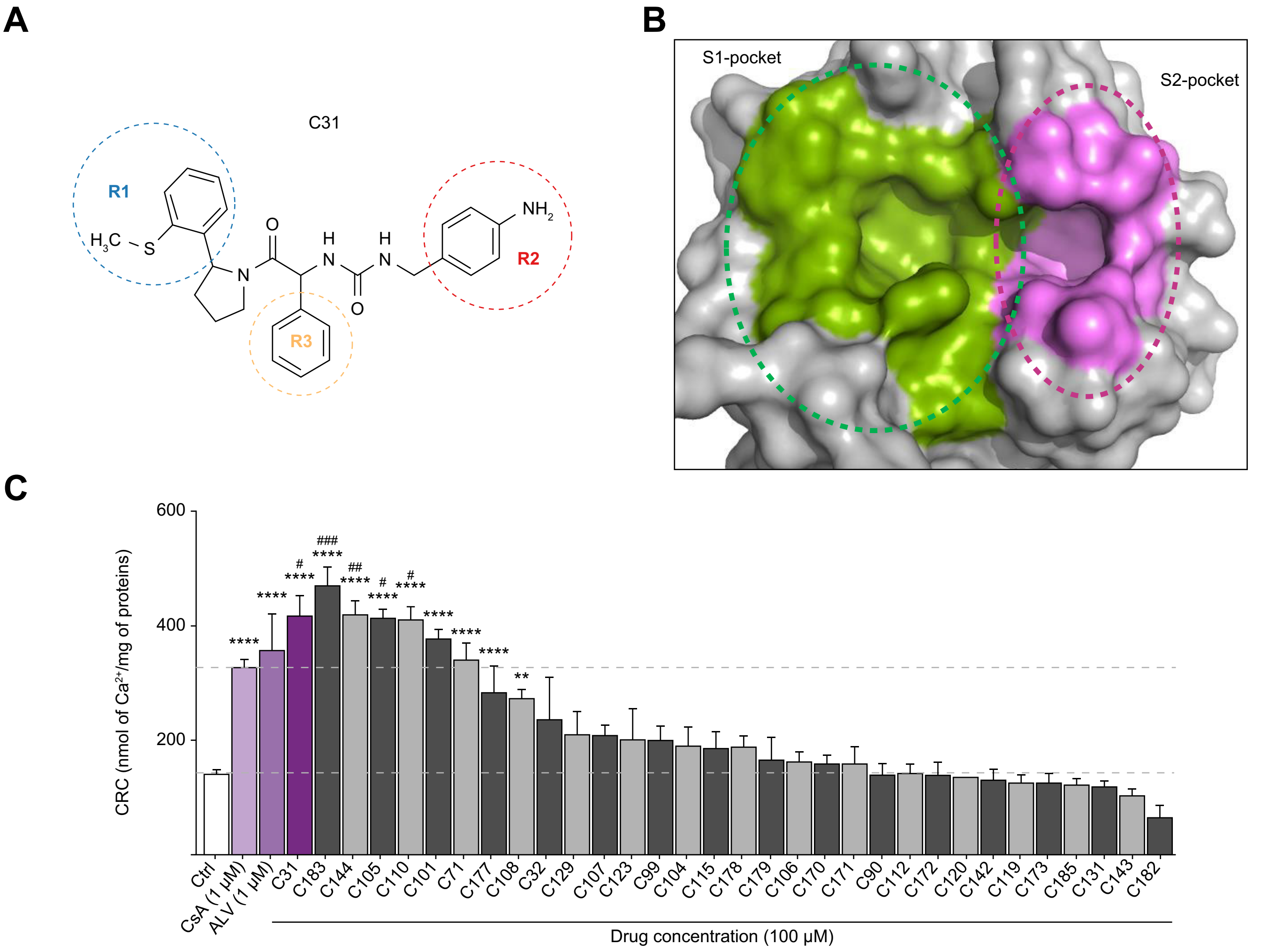


**Fig. 1. Concentration-dependent enhancement of calcium retention capacity of isolated mouse liver mitochondria by SMCypI C31 derivatives.** (A) Chemical structure of compound C31. (B) Surface representation of CypD showing the PPIase catalytic site (S1 pocket) and the gatekeeper pocket (S2 pocket). (C) CRC of mouse liver mitochondria in the absence (Ctrl) or in the presence of CsA (1 µM), ALV (1 µM), C31 (100 µM), or C31 derivatives (100 µM), ranked by decreasing level of CRC, expressed as nmol of calcium per mg of mitochondrial proteins. Data are shown as mean ± SEM. One way ANOVA analysis followed by Tukey's or Dunnett's post-test if ANOVA produced a significant value of F. $^{**}p$ <0.001 *vs.* Ctrl; $^{****}p$ <0.0001 *vs.* Ctrl; $^{\#}p$ <0.05 *vs.* CsA; $^{\#\#}p$ <0.01 *vs.* CsA; $^{\#\#\#}p$ <0.0001 *vs.* CsA. ALV, alisporivir; CsA, cyclosporin A; CypD, cyclophilin D; CRC, calcium retention capacity; PPIase, peptidyl-prolyl *cis-trans* isomerase; SMCypIs, small-molecule cyclophilin inhibitors.

heterocycles containing an amine moiety; (iii) the phenyl group (R3 position) was substituted to allow interaction with CypD residues R97 and H168. A total of 31 derivatives with single, double, or triple chemical modifications of the R1, R2, and/or R3 groups were selected to reflect these changes (Fig. S1).

We first assessed the capacity of the newly generated compounds to inhibit CypD PPIase activity using a standard chymotrypsin-coupled assay. The assay relies on the ability of α-chymotrypsin to release *p*-nitroanilide from peptides such as N-succinyl-Ala-Ala-Pro-Phe *p*-nitroanilide only if the prolyl amide bond is in the *trans* conformation. The release of *p*-nitroanilide is enhanced by the PPIase activity of CypD after isomerisation of the *cis* conformer into *trans*. The $IC_{50}$ values in the PPIase assay ranged from 0.10 ± 0.02 to 1.26 ± 0.37 µM (Table 1), *vs*. 0.02 ± 0.003 µM and 0.03 ± 0.005 µM, for CsA and ALV, two potent macrocyclic cyclophilin inhibitors, respectively (Table 1).

We next evaluated the ability of the newly generated SMCypIs to increase mitochondrial calcium retention capacity (CRC) in isolated mouse liver mitochondria. In basal conditions, 160 ± 8 nmol $Ca^{2+}$/mg of mitochondrial proteins were required to induce mPTP opening. In the presence of CsA or ALV at 1 µM, the amount of $Ca^{2+}$ required to open mPTP was raised to 328 ± 10 and 373 ± 59 nmol $Ca^{2+}$/mg of mitochondrial proteins, respectively (Fig. 1C). Because we previously observed that the maximal effect of C31 was achieved at a concentration of 100 µM,[16] we first evaluated the ability of the new derivatives to increase the mitochondrial CRC at this concentration. As shown in Fig. 1C, eight compounds increased the mitochondrial CRC. Among them, four compounds displayed a greater maximal mitochondrial CRC than CsA, including C183 (473 ± 38 nmol $Ca^{2+}$/mg of mitochondrial proteins), C144 (422 ± 44 nmol $Ca^{2+}$/mg of mitochondrial proteins), C105 (407 ± 13 nmol $Ca^{2+}$/mg of mitochondrial proteins), and C110 (400 ± 27 nmol $Ca^{2+}$/mg of mitochondrial proteins) (Fig. 1C). These four compounds induced a concentration-dependent increase in the CRC with $EC_{50}$ values of 10.21 ± 0.06 µM for C183, 11.76 ± 0.01 µM for C144, 2.37 ±

**Table 1. $IC_{50}$ values for inhibition of CypD PPIase activity by C31 derivatives**

| Compound | CypD PPIase activity $IC_{50}$ (μM) |
|---|---|
| CsA | 0.02 ± 0003 |
| ALV | 0.03 ± 0.005 |
| C31 | 0.20 ± 0.10 |
| C32 | 1.18 ± 0.43 |
| C71 | 0.79 ± 0.21 |
| C90 | 0.32 ± 0.16 |
| C99 | 0.63 ± 0.49 |
| C101 | 0.40 ± 0.19 |
| C104 | 0.22 ± 0.06 |
| C105 | 0.35 ± 0.16 |
| C106 | 1.26 ± 0.11 |
| C107 | 0.17 ± 0.05 |
| C108 | 0.48 ± 0.23 |
| C110 | 0.10 ± 0.02 |
| C112 | 0.48 ± 0.04 |
| C115 | 0.50 ± 0.32 |
| C119 | 0.15 ± 0.03 |
| C120 | 0.15 ± 0.05 |
| C123 | 1.26 ± 0.37 |
| C129 | 0.26 ± 0.07 |
| C131 | 0.48 ± 0.12 |
| C142 | 0.42 ± 0.10 |
| C143 | 0.72 ± 0.21 |
| C144 | 0.31 ± 0.16 |
| C170 | 1.05 ± 0.72 |
| C171 | 0.34 ± 0.13 |
| C172 | 0.21 ± 0.07 |
| C173 | 0.65 ± 0.14 |
| C177 | 0.49 ± 0.32 |
| C178 | 0.40 ± 0.19 |
| C179 | 0.28 ± 0.06 |
| C182 | 0.16 ± 0.03 |
| C183 | 0.26 ± 0.13 |
| C185 | 0.59 ± 0.19 |

ALV, alisporivir; CsA, cyclosporin A; CypD, cyclophilin D; PPIase, peptidyl-prolyl *cis-trans* isomerase.

0.11 μM for C105, and 2.83 ± 0.09 μM for C110, *vs.* 0.23 ± 0.02 μM and 0.08 ± 0.02 μM for CsA and ALV, respectively (Table 2).

The four compounds also inhibited $Ca^{2+}$-induced swelling of energised isolated mouse liver mitochondria in a concentration-dependent manner (Fig. S2) with $IC_{50}$ values of 2.56 ± 0.02 μM for C183, 11.57 ± 0.01 μM for C144, 0.69 ± 0.05 μM for C105, and 0.37 ± 0.006 μM for C110, *vs.* 0.05 ± 0.01 μM and 0.05 ± 0.02 μM for CsA and ALV, respectively (Table 2). Among the tested compounds, C105 and C110 were found to be the most potent cyclophilin inhibitors with the lowest $IC_{50}$ values. C105 and C110 shared similar substitutions of the R1 (benzothiophene) and R3 (3-bromo-6-methoxy-phenyl) moieties, while C105 had an additional modification of the R2 group (tetrahydroquinoline), as compared with the parent SMCypI compound C31 (Fig. S1 and Fig. 1A).

### Improvement of the mitoprotective properties of the SMCypIs by stereoselectivity

Both C105 and C110 display two asymmetric carbons. We synthesised their four diastereoisomers (SS, SR, RS, RR) (Fig. S3) and evaluated their mitoprotective properties by assessing their ability to inhibit CypD PPIase activity and mPTP opening. Only C105SR and C110SR inhibited CypD PPIase activity (Fig. 2A) and this effect was concentration-dependent ($IC_{50}$ values: 0.005 ± 0.001 μM and 0.008 ± 0.002 μM, respectively) (Table 2 and Fig. 2B). Apart from compound C105SS, which exerted a weak effect, C105SR and C110SR were the only diastereoisomers capable to induce greater than control mitochondrial CRC at 100 μM (Fig. 2C), an effect that was concentration-dependent ($EC_{50}$ values: 0.05 ± 0.02 μM and 0.14 ± 0.02 μM, respectively) (Table 2 and Fig. 2D). Interestingly, C105SR and C110SR showed greater maximal mitochondrial CRC than their respective racemic mixtures (595 ± 38 *vs.* 407 ± 13 nmol $Ca^{2+}$/mg of mitochondrial proteins for C105SR and C105, respectively and 670 ± 52 *vs.* 400 ± 27 nmol $Ca^{2+}$/mg of mitochondrial proteins for C110SR and C110, respectively) (Fig. 2E). At the 5 μM concentration and above, both C105SR and C110SR exerted a stronger effect than the maximum effect achieved by CsA (Fig. 2E). At lower concentrations (0.5 and 1 μM), their protective effects were comparable to those of CsA and ALV (Fig. 2E).

Similarly, only compounds C105SR and C110SR were capable to inhibit $Ca^{2+}$-induced mitochondrial swelling ($IC_{50}$ values: 0.009 ± 0.001 μM and 0.04 ± 0.005 μM, respectively, Fig. S4). C105SR was 10-times more potent than C110SR, suggesting the importance of the substitution of the R2 group that differentiates these two compounds (Table 2). The $EC_{50}$ values of C105SR were better than those of CsA and ALV (Table 2). Together, these results demonstrate that only the SR diastereoisomers of compounds C105 and C110 carry their biological activity, with greater mitoprotective properties than the parent compound C31, their racemic mixtures, and the macrocyclic Cyp inhibitors, CsA and ALV.

### Effects of the new SMCypIs on mPTP opening *in vitro*

We next evaluated the ability of compounds C105SR and C110SR to inhibit mPTP opening in cells. The hepatocyte cell line AML-12 was subjected to 4 h of hypoxia followed by 1 h of reoxygenation and mPTP opening was monitored using the $CoCl_2$-calcein-acetoxymethyl ester (AM) fluorescence-quenching method.[19]

**Table 2. $IC_{50}$ or $EC_{50}$ values of CsA, ALV, and different SMCypIs derivatives from C31 for inhibition of CypD PPIase activity in an enzyme assay, and for mitochondrial swelling and calcium retention capacity in isolated mouse liver mitochondria**

| Compound | CypD PPIase activity $IC_{50}$ (μM) | Mitochondrial swelling $IC_{50}$ (μM) | Calcium retention capacity $EC_{50}$ (μM) |
|---|---|---|---|
| CsA | 0.02 ± 0.003 | 0.05 ± 0.01 | 0.23 ± 0.02 |
| ALV | 0.03 ± 0.005 | 0.05 ± 0.02 | 0.08 ± 0.02 |
| C31 | 0.20 ± 0.10 | 1.30 ± 0.05 | 9.09 ± 0.21 |
| C183 | 1.03 ± 0.13 | 2.56 ± 0.02 | 10.21 ± 0.06 |
| C144 | 0.64 ± 0.15 | 11.57 ± 0.01 | 11.76 ± 0.01 |
| C105 | 0.57 ± 0.12 | 0.69 ± 0.05 | 2.37 ± 0.11 |
| C110 | 0.11 ± 0.01 | 0.37 ± 0.006 | 2.83 ± 0.09 |
| C105SR | 0.005 ± 0.001 | 0.009 ± 0.001 | 0.05 ± 0.02 |
| C110SR | 0.008 ± 0.002 | 0.04 ± 0.005 | 0.14 ± 0.02 |

ALV, alisporivir; CsA, cyclosporin A; CypD, cyclophilin D; PPIase, peptidyl-prolyl *cis-trans* isomerase.

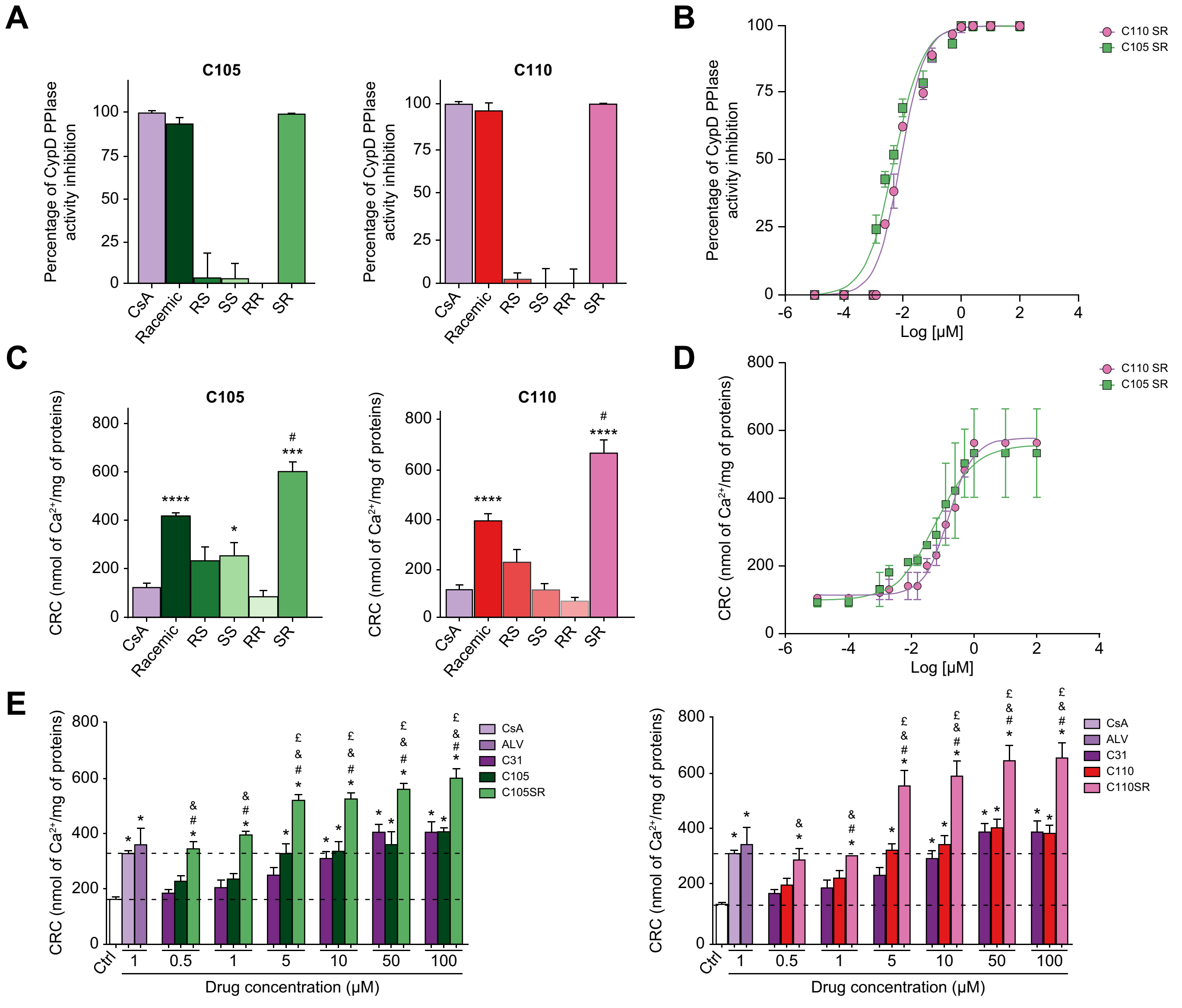


**Fig. 2. Biological activity of the racemic mixtures and diastereoisomers of compounds C105 and C110.** (A) Inhibition of CypD PPIase activity by C105 (left) and C110 (right) racemic mixtures and their diastereoisomers at 10 µM expressed as percent of the complete inhibition of CypD PPIase activity induced by CsA. (B) Concentration-response curves of CypD PPIase activity inhibition by diastereoisomers C105SR and C110SR. (C) Mitochondrial CRC of mouse liver mitochondria in the absence (Ctrl) or in the presence of C105 (left) and C110 (right) racemic mixture and their diastereoisomers at 100 µM. Data are shown as mean ± SEM. One way ANOVA analysis followed by Tukey's or Dunnett's post-test if ANOVA produced a significant value of F. $^{*}p$ <0.05 *vs.* Ctrl;; $^{***}p$ <0.001 *vs.* Ctrl; $^{****}p$ <0.0001 *vs.* Ctrl; $^{\#}p$ <0.05 *vs.* racemic mixture. (D) Concentration-response curves of mitochondrial CRC of compounds C105SR and C110SR. (E) Mitochondrial CRC in the absence or in the presence of 1 µM CsA, or 1 µM ALV or increasing concentrations of C31, C105, and C105SR (left) or C31, C110, and C110SR (right). Data are shown as mean ± SEM. One way ANOVA analysis followed by Tukey's or Dunnett's post-test if ANOVA produced a significant value of F. $^{*}p$ <0.05 *vs.* Ctrl; $^{\#}p$ <0.05 *vs.* racemic mixture; $^{\&}p$ <0.05 *vs.* C31; $^{£}p$ <0.05 *vs.* CsA. CRC, calcium retention capacity; CsA, cyclosporin A; CypD, cyclophilin D; PPIase, peptidyl-prolyl *cis-trans* isomerase.

Calcein-AM is a membrane permeable fluorophore which passively diffuses in all subcellular compartments including mitochondria. In the cell, the AM group of the fluorophore is cleaved by ubiquitous intracellular esterases, generating a hydrophilic product that is retained within the cell. The cells are then loaded with the divalent cobalt cation ($Co^{2+}$), which quenches calcein fluorescence in all subcellular compartments except the mitochondrial matrix, because the inner mitochondrial membrane is impermeable to cobalt. However, when mPTP is open, cobalt enters mitochondria and quenches mitochondrial calcein fluorescence.

Cells subjected to hypoxia/reoxygenation exhibited lower calcein fluorescence than normoxic cells, indicating that mPTP opening occurred (Fig. 3A and 3B). This effect was substantially prevented by CsA, ALV, C105SR, and C110SR, but not by C105 and C110 racemic mixtures (Fig. S5), nor by C31 at 1 µM (Fig. 3A and

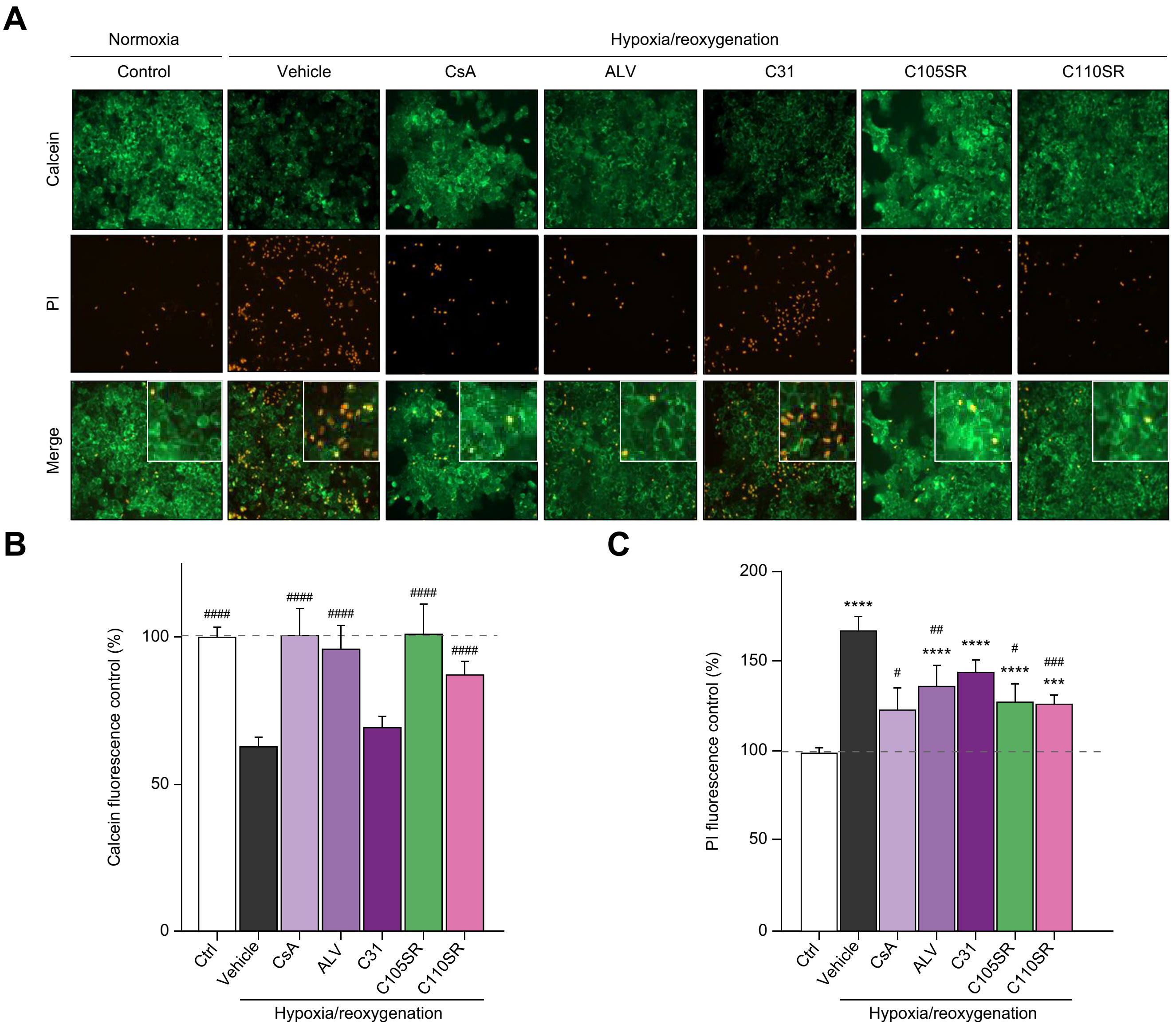


**Fig. 3. *In vitro* inhibition of mPTP opening and reduction of necrosis by C105SR and C110SR in a model of hepatic hypoxia/reoxygenation.** Cells were pretreated with 1 μM calcein and 1 mM $CoCl_2$ for 30 min and 10 min, respectively, then subjected to 4 h of hypoxia (1% $O_2$) followed by 1 h of reoxygenation (21% $O_2$) in the presence of 3 μM propidium iodide (PI). CsA and ALV were used as references. CsA, ALV, C31, C105SR, and C110SR were added at 1 μM for the entire duration of hypoxia/reoxygenation. (A) Representative images of calcein (green) and PI (red) labelling in cells exposed to normoxia (control) or hypoxia/reoxygenation in the absence (vehicle) or in the presence of CsA, ALV, C31, C105SR, or C110SR (original magnification, 400 × ). (B) Calcein fluorescence in cells exposed to normoxia (Ctrl) or hypoxia/reoxygenation in the absence (vehicle) or in the presence of CsA, ALV, C31, C105SR, or C110SR. Data are shown as mean ± SEM. One way ANOVA analysis followed by Tukey's or Dunnett's post-test if ANOVA produced a significant value of F. $^{####}p$ <0.001 *vs.* vehicle. (C) PI fluorescence in cells exposed to normoxia (Ctrl) or hypoxia/reoxygenation in the absence (vehicle) or in the presence of CsA, ALV, C31, C105SR or C110SR. Data are shown as mean ± SEM. One way ANOVA analysis followed by a Tukey's or a Dunnett's post-test if ANOVA produced a significant value of F. $^{***}p$ <0.001 *vs.* Ctrl; $^{****}p$ <0.0001 *vs.* Ctrl; $^{#}p$ <0.05 *vs.* vehicle; $^{##}p$ <0.01 *vs.* vehicle; $^{###}p$ <0.001 *vs.* vehicle. ALV, alisporivir; CsA, cyclosporin A; mPTP, mitochondrial permeability transition pore.

3B). These results indicate that compounds C105SR and C110SR inhibit mPTP opening at low concentrations in an *in vitro* hypoxia/reoxygenation model.

### Hepatoprotective properties of the new SMCypIs *in vitro*

To assess whether the new SMCypIs reduced mPTP opening-induced cell death, propidium iodide fluorescence was measured in cells that had been co-loaded with calcein-AM. As expected, the loss of calcein fluorescence in cells subjected to hypoxia/reoxygenation was associated with enhanced propidium iodide fluorescence (Fig. 3A and 3C). In contrast, high calcein fluorescence in cells treated with 1 μM CsA, ALV, C105SR or C110SR was associated with low propidium iodide fluorescence (Fig. 3A and 3C). These results suggest that C105SR and C110SR reduce mPTP opening-induced cell death.

To confirm these results, LDH release was measured in culture media, while cell viability was assessed by means of an MTT assay after 4 h of hypoxia and 2 h of reoxygenation. As expected, cells subjected to hypoxia/reoxygenation were characterised by greater LDH release and reduced cell viability as compared to

untreated controls (Fig. 4A and 4B). C105SR and C110SR, applied during hypoxia and reoxygenation, protected against cell death, as shown by the reduced LDH release and the increased cell viability (Fig. 4A and 4B). Both compounds were more efficient than C31, with protective effects comparable to those of CsA and ALV (Fig. 4A and 4B). C105SR showed potent activity inhibiting LDH release and increased cell viability by approximatively 75% at 0.5 μM, whereas C110SR was not effective at this concentration (Fig. 4A and 4B). Similar results were obtained when C105SR and C110SR were applied only during hypoxia (pharmacological preconditioning, Fig. S6) or only during reoxygenation (pharmacological postconditioning, Fig. S7). Together, these results demonstrate that C105SR is the most effective compound in reducing hypoxia/reoxygenation-induced cell death.

Necrosis is the major cause of cell death during hepatic IRI. However, mPTP opening also leads to apoptosis resulting from mitochondrial swelling, rupture of the outer mitochondrial membrane and subsequent release of cytochrome C into the cytosol.[20] We investigated whether C105SR and C110SR protected against mPTP opening-induced apoptotic cell death. Cells subjected to hypoxia/reoxygenation showed enhanced apoptosis characterised by enhanced caspase 3/7 activity (Fig. 4C). C105SR and C110SR, applied during hypoxia and reoxygenation, protected against hypoxia/reoxygenation-induced cell apoptosis (Fig. 4C).

### Molecular modelling of the interaction of C105SR with CypD

The crystal structures of CypD in complex with the ligands were used to dock C31 and the different C105 diastereoisomers using the @TOME-2 server. Comparative docking of ligands cocrystallised with the structure templates confirmed proper modelling of the interactions predicted in the binding sites. The resulting complexes predicted the interaction of C105SR with CypD (Fig. 5A and 5B), whereas the other C105 diastereoisomers were not predicted to properly interact with CypD in keeping with our results showing activity only for diastereoisomer C105SR. The predicted binding mode of C105SR (Fig. 5A and 5B) showed binding to both the PPIase catalytic site and the gatekeeper pocket, with key interactions maintained between CypD and C105SR, including hydrogen bonds with R97, Q105, N146, and T149 and van der Waals interactions with I99, F102, M103, A145, Q153, W163, and L164. C105SR displayed another hydrogen bond between R97 and the methoxy-phenyl moiety (R3), an increase of van der Waals interaction between I99, F102, W163, L164, H168 and the benzothiophene (R1) and the bromophenyl (R3) moieties. The binding mode appeared to be similar for C31 and C105SR (Fig. 5C and 5D). Together, these docking experiments validate C105SR as the most effective CypD inhibitor in this study.

### *In vivo* hepatoprotective properties of the new SMCypIs

The protective effects of the most potent SMCypI derivative, C105SR, were measured in a mouse model of liver IRI. Briefly, 70% of the liver was subjected to 1 h of ischaemia followed by 6 h of reperfusion, as recently described.[16] C105SR was administered using osmotic pumps at a dose of 50 mg/kg. A tissue distribution study showed that plasmatic and hepatic concentrations of C105SR were around 30 nM (Fig. S8). IRI resulted in a significant increase in hepatocyte necrosis and serum alanine aminotransferase (ALT) and aspartate aminotransferase (AST) levels, as compared with sham-operated mice. As shown in Fig. 6A (histology) and 6B (ALT and AST levels), C105SR significantly protected mouse livers against the effects of ischaemia and reperfusion. Moreover, as shown in Fig. 6C, the number of TUNEL-positive apoptotic cells was significantly reduced in C105SR-treated mice with IRI, as compared with mice treated with the vehicle. Together, these results demonstrate that C105SR bears protective properties against hepatic IRI.

## Discussion

Hepatic IRI induces compromised liver function and leads to frequent graft rejection and represents one of the most challenging liver disorders faced by hepatologists and liver surgeons. Therefore, there is an urgent need to develop effective treatments against hepatic IRI. In this respect, opening of the mPTP has emerged as a critical pathophysiological event mediating cell death during IRI, making it a promising therapeutic target for hepatoprotection. The lack of a clear model of the molecular structure of the mPTP has focused attention on the development of inhibitors of the best characterised mPTP modulator, CypD. However, current Cyp inhibitors are limited by their immunosuppressive properties or off-target effects. Therefore, novel mPTP inhibitors are required to translate this promising therapeutic strategy into clinical practice.

We previously developed a new family of SMCypIs, which exerted mitoprotective and hepatoprotective effects both *in vitro* and *in vivo* in an experimental mouse model of hepatic IRI.[16] Here, we used structure-guided optimisation to improve the mitoprotective and hepatoprotective properties of our initial best compound C31. We identified a new SMCypI diastereoisomer, C105SR, that was 144- and 182-fold more potent than C31 in inhibiting mitochondrial swelling and increasing mitochondrial CRC, respectively. In addition, C105SR inhibited mPTP opening and cell death at a very low concentration (0.5 μM), whereas C31 was not effective at this concentration.

Molecular modelling of the interaction of C105SR with CypD showed the capability of the compound to interact with the two CypD pockets. The three-dimensional structure predictions identified seven hydrogen bonds and a large number of van der Waals contacts, which could explain the high potency of C105SR against CypD PPIase activity. Docking studies were in keeping with the observation that C105SR was the active diastereoisomer. The use of a single enantiomer of a chiral drug has potential clinical advantages, such as an improved therapeutic index and pharmacological profile, simplified pharmacokinetics, and reduced drug interactions.

Our study showed that C105SR protects against hepatic IRI *in vivo* at a dose of 50 mg/kg, i.e. a lower dose than that used in our previous study (150 mg/kg).[16] The better efficacy of C105SR at a lower dose could be at least partly explained by the mode of administration. Indeed, in our initial study, C31 was administered by infusion 1 min before and during the first 8 min of reperfusion, whereas in the present study, C105SR was administered using ALZET osmotic pumps implanted 24 h before surgery to achieve a continuous delivery of the compound. C105SR had the capacity to protect cells exposed to hypoxia/reoxygenation injury against cell death when applied during the ischemic and the reoxygenation period. Such pharmacologic preconditioning is transposable to clinical situations in which IRI can be anticipated, such as surgical resection and transplantation. However, it has been well established that mPTP opening occurs mainly at the onset of reperfusion.[21] We therefore investigated whether C105SR could also protect against

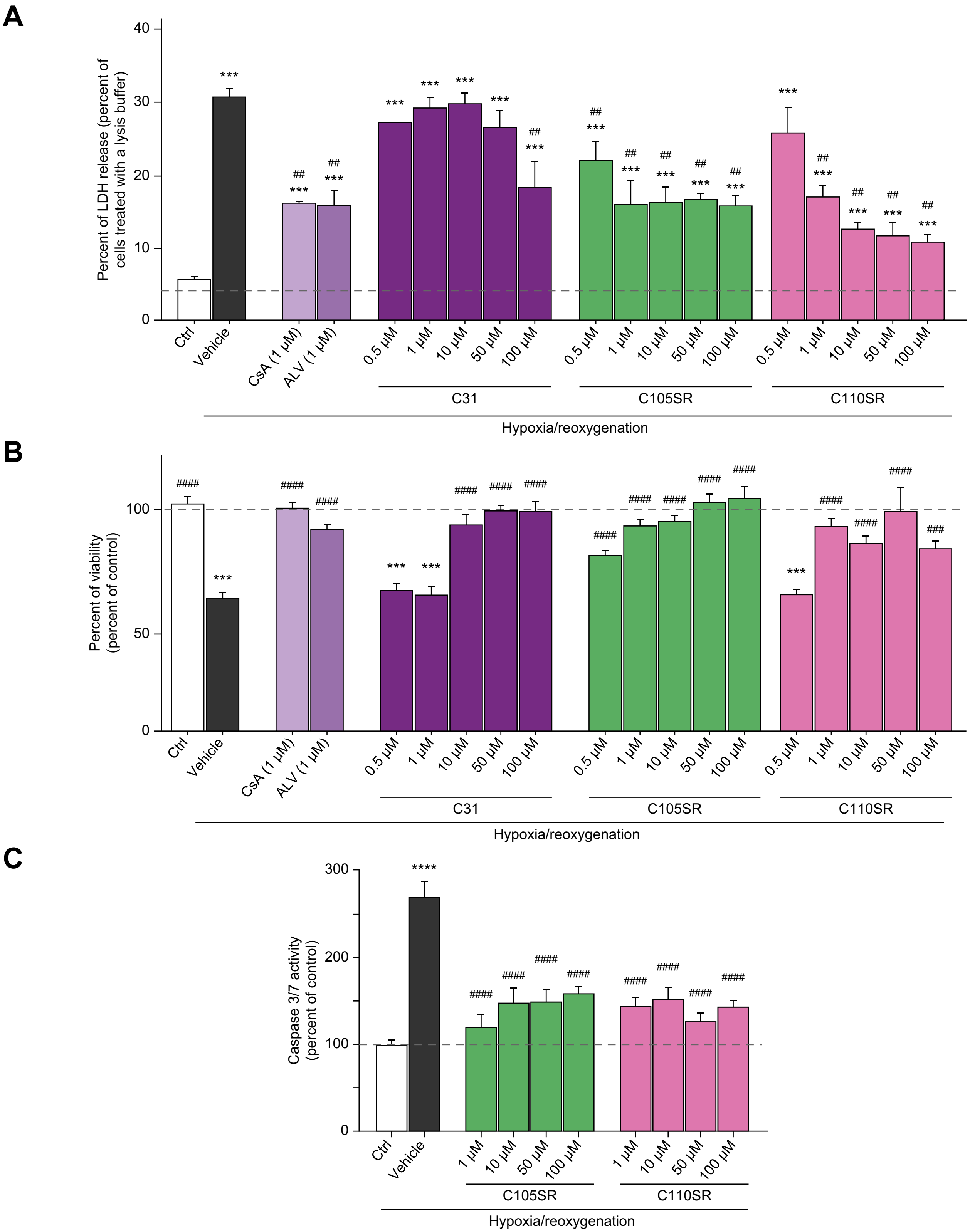


**Fig. 4. *In vitro* reduction of LDH release and increase in cell viability induced by C105SR and C110SR in a model of hepatic hypoxia/reoxygenation.** Cells were subjected to 4 h of hypoxia (1% $O_2$) followed by 2 h of reoxygenation (21% $O_2$). CsA and ALV were used as references. CsA and ALV were added at 1 μM while C31, C105SR, and C110SR were added at increasing concentrations during the hypoxic and reoxygenation phases. (A) LDH release from cells exposed to normoxia (Ctrl) or hypoxia/reoxygenation in the absence (vehicle) or in the presence of CsA, ALV, or increasing concentrations of C31, C105SR, or C110SR expressed as percentage of LDH release in cells treated with a lysis buffer. Data are shown as mean ± SEM. One way ANOVA analysis followed by Tukey's or Dunnett's post-test if ANOVA produced a significant value of F. $^{***}p$ <0.001 *vs.* Ctrl; $^{\#\#}p$ <0.01 *vs.* Vehicle. (B) Cell viability measured by MTT assay in cells exposed to normoxia (Ctrl) or

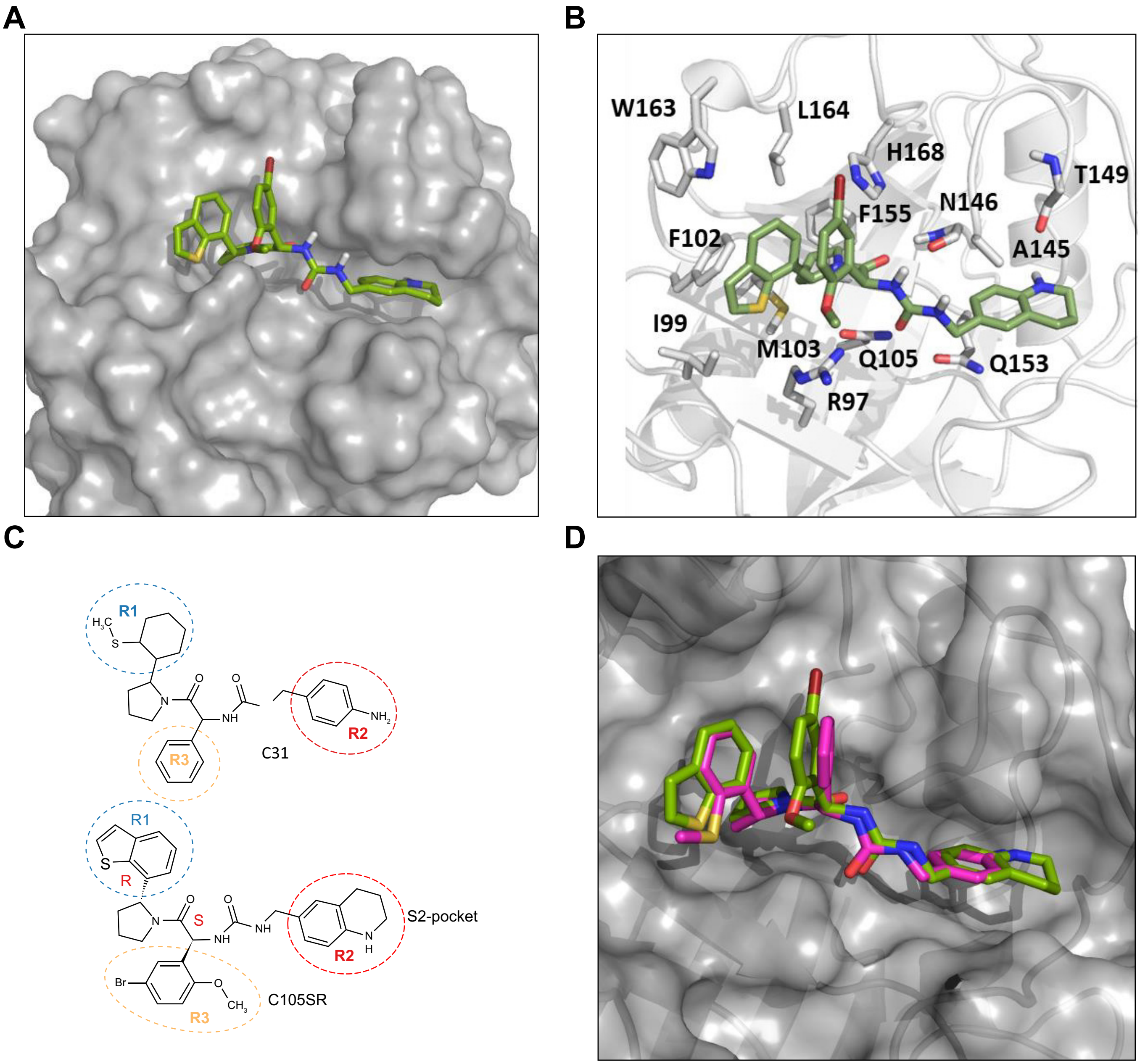


**Fig. 5. Docking of C31 and C105SR on CypD. CypD, C31, and C105SR are shown in grey, pink, and green, respectively.** (A) Large view of C105SR docked on CypD. (B) Zoom view of C105SR docked on CypD showing amino acid interactions. (C) Chemical structures of C31 and C105SR, showing their similar backbones and the differences in R1, R2, and R3 functional regions. (D) Superposition of docking poses of C31 and C105SR on CypD. CypD, cyclophilin D.

death in cells exposed to hypoxia/reoxygenation when applied only during the reoxygenation period. Our data showed similar hepatoprotective effects of C105SR when the compound was applied during the hypoxia period, during the reoxygenation period, or during both (Fig. S3 and S4 and Fig. 4).

An important finding of the present study was that C105SR was more potent than CsA and ALV, two prototypical cyclophilin inhibitors. Indeed, C105SR inhibited CypD PPIase activity and mitochondrial swelling and increased mitochondrial CRC, with $EC_{50}$ values in the nanomolar range. CsA was identified several

hypoxia/reoxygenation in the absence (vehicle) or in the presence of CsA, ALV, or increasing concentrations of C31, C105SR, or C110SR expressed as percentage of control. Data are shown as mean ± SEM. One-way ANOVA analysis followed by Tukey's or Dunnett's post-test if ANOVA produced a significant value of F.****p* <0.001 *vs.* Ctrl; ###*p* <0.001 *vs.* Vehicle; ####*p* <0.0001 *vs.* Vehicle. (C) Caspase 3/7 activity expressed as percentage of control (Ctrl) in cells subjected to 4 h of hypoxia and 2 h of reoxygenation treated with vehicle or increasing concentrations of C105SR or C110SR. Data are shown as mean ± SEM. One way ANOVA analysis followed by Tukey's or Dunnett's post-test if ANOVA produced a significant value of F *****p* <0.0001 *vs.* Ctrl; ####*p* <0.0001 *vs.* Vehicle. ALV, alisporivir; CsA, cyclosporin A; LDH, lactate dehydrogenase.

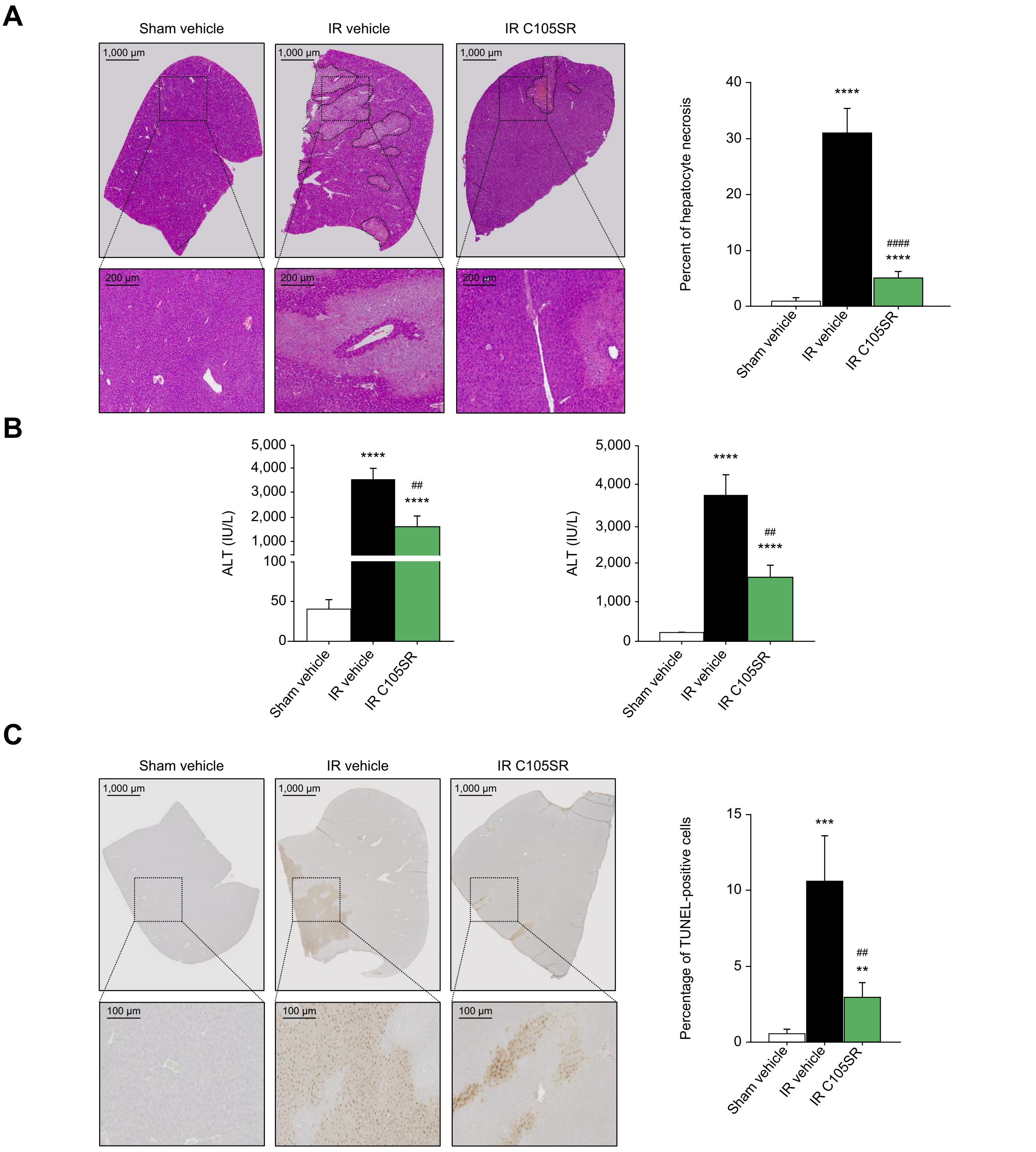


**Fig. 6. *In vivo* protective effects of C105SR (50 mg/kg) against hepatic ischaemia–reperfusion injury.** (A) Representative haematoxylin and eosin staining images (magnification, 200 × ) of liver lobes (left) and percent of hepatocyte necrosis (right) in mice subjected to laparotomy without (sham vehicle) or with ischaemia–reperfusion (IR) in the absence (vehicle) or in the presence of C105SR. n = 8 for sham vehicle; n = 24 for IR vehicle; n = 13 for IR C105SR. Data are shown as mean ± SEM. Mann–Whitney *U* test ****$p$ <0.0001 *vs.* sham vehicle; ####$p$ <0.0001 *vs.* IR vehicle. (B) Serum ALT and AST levels in mice subjected to laparotomy without (sham vehicle) or with ischaemia–reperfusion (IR) in the absence (vehicle) or in the presence of C105SR. n = 8 for sham vehicle; n = 24 for IR vehicle; n = 13 for IR C105SR. Data are shown as mean ± SEM. Mann–Whitney *U* test. ****$p$ <0.0001 *vs.* sham vehicle; ##$p$ <0.01 *vs.* IR vehicle (C) Representative TUNEL staining (left) and number of TUNEL-positive cells (right) in livers of mice subjected to laparotomy without (sham vehicle) or with ischaemia–reperfusion (IR) in the absence (vehicle) or in the presence of C105SR. n = 4 for sham vehicle; n = 8 for IR vehicle; n = 8 for IR C105SR. Data are shown as mean ± SEM. Mann–Whitney *U* test **$p$ <0.01 *vs.* sham vehicle; ***$p$ <0.001 *vs.* sham vehicle; ##$p$ <0.01 *vs.* IR vehicle. ALT, alanine aminotransferase; AST, aspartate aminotransferase; IR, ischaemia–reperfusion.

years ago as a potent inhibitor of Cyp PPIase activity and of mPTP opening.[22] However, its strong immunosuppressive properties have been a major obstacle to its clinical use in the context of IRI. ALV is a non-immunosuppressive analogue of CsA, developed for the treatment of hepatitis C virus infection, that also protects against IRI.[23] However, its development was halted in a Phase III clinical trial due to severe side effects, unrelated to its anti-Cyp activity.[24] CsA derivatives devoid of immunosuppressive properties are macromolecules resulting in poor cell permeability and complex multistep synthesis. These molecules do not bind to the "gatekeeper" pocket of the active site of Cyps. In contrast, C105SR belongs to a new family of CsA-unrelated, non-peptidic, small-molecule Cyp inhibitors, with a molar mass below 700 kDa and easy synthesis. At the structural level, using *in silico* docking, we showed in this study that C105SR binds to the two pockets of CypD, including the PPIase catalytic site and the 'gatekeeper' pocket. The 'gatekeeper' pocket has been associated with substrate specificity,[25] suggesting that the mode of binding of our compounds could lead to the synthesis of CypD-specific inhibitors in the future.

In conclusion, we identified a novel SMCypI diastereoisomer, C105SR, with the capacity to potently inhibit mPTP opening and prevent cell death *in vitro* in a model of hypoxia/reoxygenation. This compound also exhibited remarkable activity against hepatic IRI in an *in vivo* murine model. The results of this study provide a solid basis for considering C105SR as a promising candidate drug for hepatocellular protection during IRI. This compound also represents a promising candidate drug for the treatment of other liver and non-hepatic diseases that involve mitochondrial CypD-related mechanisms of cell death.

## Abbreviations

ALT, alanine aminotransferase; ALV, alisporivir; AST, aspartate aminotransferase; CRC, calcium retention capacity; CsA, cyclosporin A; CypD, cyclophilin D; H/R, hypoxia/reoxygenation; IR, ischaemia–reperfusion; IRI, ischaemia–reperfusion injury; LDH, lactate dehydrogenase; mPT, mitochondrial permeability transition; mPTP, mitochondrial permeability transition pore; MTT, 3-(4,5-dimethylthiazol-2-yl)-2,5-diphenyltetrazolium bromide; PI, propidium iodide; PPIase, peptidyl-prolyl *cis-trans* isomerase; SMCypIs, small-molecule cyclophilin inhibitors; TUNEL, terminal deoxynucleotidyl transferase (TdT) dUTP nick-end labelling; WT, wild-type.

## Financial support

This work was supported by the Inserm, the Université Paris-Est Créteil, a grant of the Agence de la Biomédecine (21GREFFE014) and a FEDER grant (48748).

## Conflicts of interest

The authors have no conflict of interest to disclose.

Please refer to the accompanying ICMJE disclosure forms for further details.

## Authors' contributions

Performed experiments and procedures: AK, NA, AH. Analysis and interpretation of the data: All authors. Concept and design of the study, funding acquisition, study supervision, preparation of original draft: FTC. Review and approval of the final manuscript: All authors.

## Data availability statement

The data that support the findings of this study are available from the corresponding authors, upon reasonable request.

## Acknowledgements

We thank Alice Da Silva, Damien Fois, Florine Weber, and Diana Gelperowic for their help with the *in vivo* experiments, and Nadir Mouri for determination of transaminase levels.

## Supplementary data

Supplementary data to this article can be found online at https://doi.org/10.1016/j.jhepr.2023.100876.

## References

*Author names in bold designate shared co-first authorship*

[1] Howard TK, Klintmalm GB, Cofer JB, Husberg BS, Goldstein RM, Gonwa TA. The influence of preservation injury on rejection in the hepatic transplant recipient. Transplantation 1990;49:103–107.

[2] Fellström B, Aküyrek LM, Backman U, Larsson E, Melin J, Zezina L. Postischemic reperfusion injury and allograft arteriosclerosis. Transpl Proc 1998;30:4278–4280.

[3] **Zhou J**, **Chen J**, Wei Q, Saeb-Parsy K, Xu X. The role of ischaemia/reperfusion injury in early hepatic allograft dysfunction. Liver Transpl 2020;26:1034–1048.

[4] Go KL, Lee S, Zendejas I, Behrns KE, Kim JS. Mitochondrial dysfunction and autophagy in hepatic ischaemia/reperfusion injury. Biomed Res Int 2015;2015:183469.

[5] Halestrap AP. What is the mitochondrial permeability transition pore? J Mol Cell Cardiol 2009;46:821–831.

[6] Briston T, Selwood DL, Szabadkai G, Duchen MR. Mitochondrial permeability transition: a molecular lesion with multiple drug targets. Trends Pharmacol Sci 2019;40:50–70.

[7] Davis TL, Walker JR, Campagna-Slater V, Finerty PJ, Paramanathan R, Bernstein G, et al. Structural and biochemical characterization of the human cyclophilin family of peptidyl-prolyl isomerases. PLoS Biol 2010;8:e1000439.

[8] Karch J, Molkentin JD. Regulated necrotic cell death: the passive aggressive side of Bax and Bak. Circ Res 2015;116:1800–1809.

[9] Nguyen TT, Stevens MV, Kohr M, Steenbergen C, Sack MN, Murphy E. Cysteine 203 of cyclophilin D is critical for cyclophilin D activation of the mitochondrial permeability transition pore. J Biol Chem 2011;286:40184–40192.

[10] Halestrap AP, Davidson AM. Inhibition of Ca2(+)-induced large-amplitude swelling of liver and heart mitochondria by cyclosporin is probably caused by the inhibitor binding to mitochondrial-matrix peptidyl-prolyl cis-trans isomerase and preventing it interacting with the adenine nucleotide translocase. Biochem J 1990;268:153–160.

[11] Clarke SJ, McStay GP, Halestrap AP. Sanglifehrin A acts as a potent inhibitor of the mitochondrial permeability transition and reperfusion injury of the heart by binding to cyclophilin-D at a different site from cyclosporin A. J Biol Chem 2002:27734793–27734799.

[12] Waldmeier PC, Feldtrauer JJ, Qian T, Lemasters JJ. Inhibition of the mitochondrial permeability transition by the nonimmunosuppressive cyclosporin derivative NIM811. Mol Pharmacol 2002;62:22–29.

[13] Theruvath TP, Zhong Z, Pediaditakis P, Ramshesh VK, Currin RT, Tikunov A, et al. Minocycline and N-methyl-4-isoleucine cyclosporin (NIM811) mitigate storage/reperfusion injury after rat liver transplantation through suppression of the mitochondrial permeability transition. Hepatology 2008;47:236–246.

[14] Pawlotsky JM, Flisiak R, Sarin SK, Rasenack J, Piratvisuth T, Chuang WL, et al. Alisporivir plus ribavirin, interferon free or in combination with pegylated interferon, for hepatitis C virus genotype 2 or 3 infection. Hepatology 2015;62:1013–1023.

[15] Kuo J, Bobardt M, Chatterji U, Mayo PR, Trepanier DJ, Foster RT, et al. A pan-cyclophilin inhibitor, CRV431, decreases fibrosis and tumor development in chronic liver disease models. J Pharmacol Exp Ther 2019;371:231–241.

[16] Simón Serrano S, Grönberg A, Longato L, Rombouts K, Kuo J, Gregory M, et al. Evaluation of NV556, a novel cyclophilin inhibitor, as a potential antifibrotic compound for liver fibrosis. Cells 2019;8:1409.

[17] Ahmed-Belkacem A, Colliandre L, Ahnou N, Nevers Q, Gelin M, Bessin Y, et al. Fragment-based discovery of a new family of non-peptidic small-

molecule cyclophilin inhibitors with potent antiviral activities. Nat Commun 2016;7:12777.
[18] Panel M, Ruiz I, Brillet R, Lafdil F, Teixeira-Clerc F, Nguyen CT, et al. Small-molecule inhibitors of cyclophilins block opening of the mitochondrial permeability transition pore and protect mice from hepatic ischaemia/reperfusion injury. Gastroenterology 2019;157:1368–1382.
[19] Petronilli V, Miotto G, Canton M, Colonna R, Bernardi P, Di Lisa F. Imaging the mitochondrial permeability transition pore in intact cells. Biofactors 1998;8:263–272.
[20] Lemasters JJ. Dying a thousand deaths: redundant pathways from different organelles to apoptosis and necrosis. Gastroenterology 2005;129:351–360.
[21] Halestrap AP, Clarke SJ, Javadov SA. Mitochondrial permeability transition pore opening during myocardial reperfusion–a target for cardioprotection. Cardiovasc Res 2004;61:372–385.
[22] Broekemeier KM, Dempsey ME, Pfeiffer DR. Cyclosporin A is a potent inhibitor of the inner membrane permeability transition in liver mitochondria. J Biol Chem 1989 May 15;264(14):7826–7830.
[23] Gomez L, Thibault H, Gharib A, Dumont JM, Vuagniaux G, Scalfaro P, et al. Inhibition of mitochondrial permeability transition improves functional recovery and reduces mortality following acute myocardial infarction in mice. Am J Physiol Heart Circ Physiol 2007;293:H1654–H1661.
[24] Zeuzem S, Flisiak R, Vierling JM, Mazur W, Mazzella G, Thongsawat S, et al. Randomised clinical trial: alisporivir combined with peginterferon and ribavirin in treatment-naïve patients with chronic HCV genotype 1 infection (ESSENTIAL II). Aliment Pharmacol Ther 2015;42:829–844.
[25] Davis TL, Walker JR, Campagna-Slater V, Finerty PJ, Paramanathan R, Bernstein G, et al. Structural and biochemical characterization of the human cyclophilin family of peptidyl-prolyl isomerases. PLoS Biol 2010;8(7):e1000439.

Journal of Hepatology, Volume *5*

## Supplemental information

# The novel cyclophilin inhibitor C105SR reduces hepatic ischaemia–reperfusion injury via mitoprotection

**Amel Kheyar, Nazim Ahnou, Abdelhakim Ahmed-Belkacem, Anne Hulin, Claire Pressiat, Bijan Ghaleh, Jean-François Guichou, Didier Morin, Jean-Michel Pawlotsky, and Fatima Teixeira-Clerc**

**The novel cyclophilin inhibitor C105SR reduces hepatic ischaemia-**

**reperfusion injury via mitoprotection**

Amel Kheyar, Nazim Ahnou, Abdelhakim Ahmed-Belkacem, Anne Hulin, Claire Pressiat,

Bijan Ghaleh, Jean-François Guichou, Didier Morin, Jean-Michel Pawlotsky, Fatima

Teixeira-Clerc

Table of contents

Supplementary materials and methods……………………………………………………………………………………………………2

Supplementary figures…………………………………………………………………………………………………………………113

**Supplementary materials and methods**

***Peptidyl-prolyl cis-trans isomerase (PPIase) activity assay.*** CypD PPIase activity and its inhibition by SMCypIs were measured at 20°C using standard chymotrypsin-coupled assay. The assay buffer (25 mM Hepes, 100 mM NaCl, pH 7.8) and CypD (1.9 µM stock solution) were pre-cooled to 4°C in the presence of SMCypIs, CsA or ALV. Then, 5 µL of 50 mg/mL chymotrypsin in 1 mM HCl was added. The reaction was started by adding 20 µL of 3.2 mM peptide substrate (N-Succinyl-Ala-Ala-Cis-Pro-Phe-p-nitroanilide, AAPF) in lithium chloride/trifluoroethanol (LiCl/TFE) solution with rapid inversion. P-nitroanilide absorbance was measured at 390 nm for 60 sec and absorbance was measured at 1-sec intervals by a spectrophotometer. CsA and ALV were used as positive controls of CypD PPIase activity inhibition in all measurements. For SMCypI PPIase inhibitory activity assessments, 5 µL of different concentrations in DMSO of the tested compound were added to the CypD solution in the assay buffer. The percent inhibition of CypD PPIase activity was calculated from the slopes and the half-maximal inhibitory concentration ($IC_{50}$) values were obtained from percent inhibition curves using Sigmaplot software (Systat, San Jose, California).

***Isolation of mouse liver mitochondria.*** Mouse livers were cut and homogenized in ice-cold homogenization buffer (220 nM mannitol, 70 mM sucrose, 10 mM HEPES, 4 mM ethylene glycol-bis (β-aminoethyl-ether)-N,N,N',N'-tetraacetic acid (EGTA), pH 7.4 at 4°C) using a Potter-Elvehjem glass homogenizer in a final volume of 10 mL of buffer per g of tissue. The homogenate was centrifuged at 1,000 g for 5 min at 4°C. The supernatant was then transferred to a clean tube and centrifuged at 9,000 g for 10 min at 4°C. The mitochondrial pellet was resuspended in a final volume of 600 µL of homogenization buffer without EGTA. Protein concentration was determined using the advanced protein assay reagent (Sigma).

***Calcium retention capacity (CRC) assay.*** Isolated liver mitochondria (1 mg/mL) energized with 5 mM glutamate/malate were incubated in a buffer allowing respiration of mitochondria (100 mM KCl, 50 mM sucrose, 10 mM HEPES, 5 mM $KH_2PO_4$, pH 7.4 at 30°C) supplemented with 1 µM calcium green 5N fluorescent probe. Mitochondria were pulsed with sequential additions of $CaCl_2$ (20 µM) until mPTP opening along with rapid calcium release happened. The calcium concentration in the extra-mitochondrial medium was monitored with a Jasco FP-6300 spectrofluorimeter (Jasco, Bouguenais, France) at 506 nm excitation and 532 nm emission wavelengths.

***Mitochondrial swelling assay.*** Mitochondrial swelling was assessed by measuring changes in absorbance at 540 nm using a Jasco V-530 spectrophotometer (Bouguenais, France) equipped with magnetic stirring and thermostatic control (30°C). Isolated liver mitochondria (0.5 mg/mL) were energized with 5 mM of pyruvate/malate. Mitochondria were incubated for 30 sec in the same buffer as CRC assay before the induction of swelling with 100 µM $CaCl_2$. SMCypIs at various concentrations were added before the induction of mitochondrial swelling. CsA and ALV (1 µM) were used as positive controls of mitochondrial swelling inhibition.

***Hypoxia/reoxygenation (H/R) in vitro model.*** AML-12 mouse hepatocyte line was obtained from American Type Culture Collection (ATCC) and maintained in DMEM/F12 culture medium at 37°C with 5% $CO_2$. HepaRG human hepatocyte line was obtained from American Type Culture Collection (ATCC) and maintained in William'S E culture medium at 37°C with 5% $CO_2$.When indicated, cells were incubated in phosphate buffered saline at 37°C in a hypoxic chamber filled with 94% $N_2$, 1% $O_2$, 5% $CO_2$ for 4 h to mimic ischemic conditions. Then, cells were transferred to a $CO_2$ incubator at 37°C in regular DMEM/F12 culture medium for 1 or 2 h under normoxic conditions (74% $N_2$, 21% $O_2$, 5% $CO_2$). SMCypIs or vehicle (DMSO) were added during the hypoxia period, during the reoxygenation period, or during both. CsA and ALV were used as positive controls for hepatic protection against H/R injury.

***Monitoring of mPTP opening.*** mPTP opening was monitored in AML-12 cells using the $CoCl_2$-calcein AM fluorescence-quenching assay. Cells were loaded with 1 µM calcein-AM (green) for 20 min. Then, 1 mM $CoCl_2$ was added and cells were incubated for another 10 min. Cells were washed and subjected to 4 h of hypoxia followed by 1 h of reoxygenation. Calcein fluorescence was measured using a spectrofluorimeter at excitation and emission wavelengths of 485 nm and 535 nm, respectively. Images were acquired using a standard inverted fluorescence microscope (Zeiss Axio, Oberkochen, Germany). SMCypIs, CsA or ALV were added at the concentration of 1 µM in culture media during the entire H/R period. Images shown are representative images of 12-18 images.

***Propidium iodide staining.*** Propidium iodide (PI) was used to identify non-viable cells as it is only permeant to cells with compromised plasma membrane integrity.[1] PI (3 µM) was added to culture media upon the reoxygenation period. Fluorescence was measured using an Infinite 200 Pro fluorescent plate reader (Tecan, Mannedorf, Switzerland) at excitation and emission wavelengths of 533 nm and 617 nm, respectively. Images were acquired using a standard inverted fluorescence microscope (Zeiss Axio, Oberkochen, Germany). Images shown are representative images of 12-18 images.

***Cell viability assays.*** Lactate dehydrogenase (LDH) release in culture medium was assessed using the commercially non-radioactive CytoTox-96 kit (Promega, Madison, Wisconsin) following the manufacturer’s instructions. Briefly, cell culture media were collected and the LDH activity was determined by measuring absorbance at 491 nm with a spectrometer (Multiskan skyHigh). For 3-(4,5-dimethylthiazol-2-yl)-2,5-diphenyltetrazolium bromide (MTT) assay, cells were incubated with a solution of MTT (0.5 mg/mL) for 2 h at 37°C. The medium was then removed and 50 µL DMSO was added to each well. Optical density of purple formazan product was measured at 550 nm. Cell viability was expressed as a percentage of the untreated control.

***Mouse serum analysis.*** Blood was collected at 6 h of reperfusion. Alanine aminotransferase (ALT) and aspartate aminotransferase (AST) activities were measured on an automated analyzer in the Biochemistry Department of Henri Mondor Hospital.

***Histological Analysis.*** Hematoxylin and eosin staining was performed on 4-µm thick formalin-fixed paraffin-embedded tissue sections. Slides were scanned at x20 magnification using a virtual slide scanning system (Axio scan7/ZEN slidescan). Necrosis areas were quantified with QuPath.

***TUNEL Assay.*** Terminal deoxynucleotidyl-transferase (TdT) dUTP Nick-End Labeling (TUNEL) was performed on formalin-fixed paraffin-embedded tissue sections using a commercial kit (Abcam, Cambridge, United Kingdom) following the manufacturer's instructions. The number of TUNEL-positive cells were quantified using QuPath on slides scanned at x20 magnification using a virtual slide scanning system (Axio scan7/ZEN slidescan).

***Caspase 3/7 activity assay.*** Caspase 3/7 activity was assessed using the commercially Caspase-Glo® 3/7 assay (Promega, Madison, Wisconsin) following the manufacturer's instructions. Briefly, Caspase-Glo® 3/7 Reagent was incubated with the cells and the caspase3/7 activity was determined by measuring luminescence with a luminometer (Berthold). Caspase3/7 activity was expressed as a percentage of the untreated control.

***In silico modeling and docking.*** The CypD sequence (P30405) was recovered from UniProt (UniProt, http://www.uniprot.org/uniprot/). The search for ligand-CypD 3D crystal complexes was performed using the @TOME-2 server (https://atome.cbs.cnrs.fr/ATOME_V3/index.html).[2] Ligand files were generated with MarvinSketch 6.2.2 for SMILES and Frog2 server for mol2. Docking

simulation was performed using @TOME-2 server with no constraint. The images were generated using PyMOL and MarvinSketch.

***Determination of plasmatic and hepatic concentration of C105SR.*** Mice (10-12 weeks-old, n=6) were anesthetized with isoflurane and subcutaneously implanted with Alzet® osmotic pump containing C105SR (50 mg/kg) or vehicle. 24h after, the blood and the liver were collected. C105SR in plasma and liver homogenates was extracted with cold acetonitrile containing 0.1% of acetic acid and centrifugation at 13,000 x g for 10 min at 4 °C. C105SR dosage was carried out by tandem mass spectrometric detection with a TSQ QUANTIS® mass spectrometer (ThermoFisher Scientific, Villebon-sur-Yvette, France), with an electrospray ionization source (ESI) which was set in positive electro-spray ionization (ESI+). The acquired data were processed using Trace Finder® software version 4.1 (Thermo Fisher Scientific, Villebon-sur-Yvette, France). Optimization of the MS conditions has been performed by single direct infusion of reference standards of the analyte. Two ion transitions were used per analyte (633.17/146.14 and 444.85).

**References**

1. Brana C, Benham C, Sundstrom L. A method for characterising cell death *in vitro* by combining propidium iodide staining with immunohistochemistry. Brain Res Brain Res Protoc. 2002 Oct;10(2):109-14.

2. Pons JL, Labesse G. @TOME-2: a new pipeline for comparative modeling of protein-ligand complexes. Nucleic Acids Res. 2009;37(Web Server issue):W485-91.

*Synthesis and the characterization of the compounds*

**Equipment and analytical methods used for the syntheses of examples:**

**Microwaves irradiation**: Apparatus: CEM Discover with Synergy Software.
Method: 10 mL or 30 mL sealed tube, power up to 50W, high stirring.

**Flash chromatography**:
Apparatus: Biotage SP with auto-collector and UV detection (2 wavelengths).
Normal phase columns: 10, 25 or 120 g Biotage external dry load cartridge kit, packed with Sigma-Aldrich 40-63 µm silica gel.
Reverse phase column: 30 g Biotage SNAP Cartridges, KP-C18-HS.
Chiral column: Daicel ChiralFlash IG 100 x 30 mm 20 µM.

**Liquid Chromatography:HPLC**
Apparatus: Waters alliance 2695 HPLC system with autosampler and Waters 2996 diode array detector.

**Reverse phase conditions:**

Column: Macherey-Nagel Nucleoshell RP18 plus (5 µm, 4 mm x 100 mm).
Column temperature: 40°C.
Solvents: A ($H_2O$ 99.9%, $H_2CO_2$ 0.1%); B (MeCN 99.9%, $H_2CO_2$ 0.1%).
Flow rate: 1mL/min.

Gradient (A/B v/v): 95/5 (t=0min), 95/5 (t=1min), 0/100 (t=7min), 0/100 (t=10min).

**Chiral phase conditions:**

Column: Daicel ChiralPak IG (Amylose-based) 20 µm, 4.6 mm x 250 mm.
Column temperature: 25°C.
Solvents: EtOH containing 0.1% TEA 90%/DCM 10%.
Flow rate: 1mL/min.
Run time: 30min

**Mass Spectrometer:**

Apparatus: Waters Micromass ZQ (simple quad).
Mass detection method: Electrospray positive mode (ESI+), mass range: 50-800 uma.
Detection: 210-400nm range.

**NMR Spectrometer:**

Apparatus: Bruker 400 MHz.

**Example 1: Synthesis of 1-(4-aminobenzyl)-3-(1-(2,5-dimethoxyphenyl)-2-(2-(2-(methylthio)phenyl)pyrrolidin-1-yl)-2-oxoethyl)urea: C90**

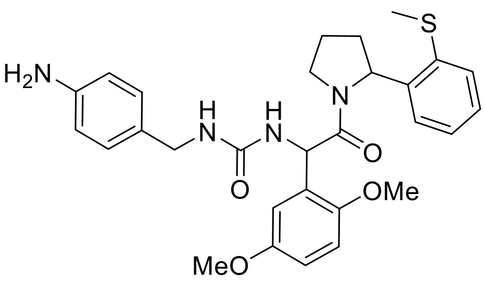

**Step 1: methyl 2-amino-2-(2,5-dimethoxyphenyl)acetate**

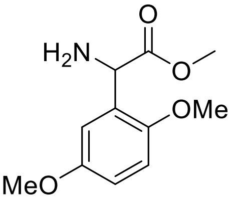

250 mg (1.18 mmol, 1 eq) of 2-amino-2-(2,5-dimethoxyphenyl)acetic acid are dissolved in 3 mL of dry MeOH under Argon. The solution is cooled to 0°C and 90 µL (1.42 mmol, 1.2 eq) of $SOCl_2$ are added. The mixture is stirred 5 min at this temperature and then 2h at reflux. Reaction is diluted with EtOAc and washed 3 times with a saturated $NaHCO_3$ solution. Aqueous layer is extracted 3 times with EtOAc. Combined organic layers are dried over $Na_2SO_4$, filtered and evaporated unde reduced pressure to give 162 mg of a yellow oil.

Yield : 61%

MH+ : 226.3 (M+1)

**Step 2: methyl 2-((tert-butoxycarbonyl)amino)-2-(2,5-dimethoxyphenyl)acetate**

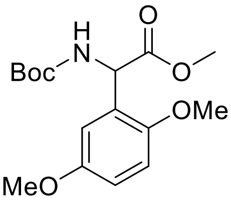

162 mg (0.72 mmol, 1 eq) of methyl 2-amino-2-(2,5-dimethoxyphenyl)acetate (described in previous step) are dissolved in 3 mL of dry THF under Argon. 164 mg (0.76 mmol, 1.05 eq) of $Boc_2O$ are added and the solution is stirred 1h at room temperature. Reaction is diluted with EtOAc and washed 2 times with a saturated $NaHCO_3$ solution. Organic layer is dried over $Na_2SO_4$, filtered and evaporated unde reduced pressure. Crude product is purified by flash chromatography using a silica gel column and an Hexane/EtOAc mixture as eluent. 215 mg of the title compound are obtained as a pale yellow oil.

Yield : 92%

MH+ : 326.5 (M+1)

**Step 3: 2-((tert-butoxycarbonyl)amino)-2-(2,5-dimethoxyphenyl)acetic acid**

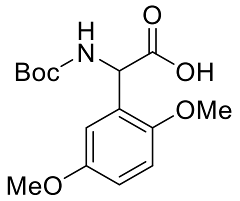


215 mg (0.66 mmol, 1 eq) of methyl 2-((tert-butoxycarbonyl)amino)-2-(2,5-dimethoxyphenyl)acetate (described in previous step) are dissolved in 6 mL of $H_2O$/MeOH solution (1/1, v/v). 58 mg (1.45 mmol, 2.2 eq) of NaOH are added and the solution is heated 1h at 70°C. Reaction is diluted with water and pH is adjusted to 9 with NaOH 2N. Aqueous layer is extracted 3 times with EtOAc, then aqueous layer pH is acidified with HCl 2N to reached a value of 2. Acidic aqueous layer is extracted 3 times with EtOAc and this organic layer is dried over $Na_2SO_4$, filtered and evaporated unde reduced pressure to give 168 mg of a yellow oil.

Yield : 82%

MH+ : 312.5 (M+1)

**Step 4: tert-butyl (1-(2,5-dimethoxyphenyl)-2-(2-(2-(methylthio)phenyl)pyrrolidin-1-yl)-2-oxoethyl)carbamate**

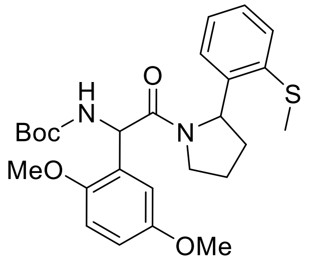


84 mg (0.27 mmol, 1 eq) of 2-((tert-butoxycarbonyl)amino)-2-(2,5-dimethoxyphenyl)acetic acid (described in previous step), 52 mg (0.27 mmol, 1 eq) of 2-(2-(methylthio)phenyl)pyrrolidine and 280 µL (1.62 mmol, 6 eq) DIEA are dissolved in 2 mL of dry DCM under Argon. 62 mg (0.32 mmol, 1.2 eq) of EDC and 44 mg (0.32 mmol, 1.2 eq) of HOBt are added and the solution is stirred overnight at room temperature. Reaction is diluted with EtOAc and washed one time with water, 2 times with a saturated $NaHCO_3$ solution and one time with brine. Organic layer is dried over $Na_2SO_4$, filtered and evaporated unde reduced pressure. Crude product is purified by flash chromatography using a silica gel column and an DCM/MeOH mixture as eluent. 140 mg of the title compound is obtained as a pale yellow oil.

Yield : quant.

MH+ : 487.7 (M+1)

**Step 5: 2-amino-2-(2,5-dimethoxyphenyl)-1-(2-(2-(methylthio)phenyl)pyrrolidin-1-yl)ethan-1-one**

131 mg (0.27 mmol, 1 eq) of tert-butyl (1-(2,5-dimethoxyphenyl)-2-(2-(2-(methylthio)phenyl)pyrrolidin-1-yl)-2-oxoethyl)carbamate (described in previous step) is dissolved in 1.5 mL of dry DCM under Argon. 1.5 mL of TFA is added and the solution is stirred 1h at room temperature. Reaction is evaporated under reduced pressure, diluted with a saturated $NaHCO_3$ solution and extracted 3 times with DCM. Combined organic layers are dried over $Na_2SO_4$, filtered and evaporated unde reduced pressure. Crude product is purified by flash chromatography using a silica gel column and an DCM/MeOH mixture as eluent. 63 mg of the title compound is obtained as a pale yellow oil.

Yield : 60%

MH+ : 387.5 (M+1)

**Step 6: tert-butyl (4-((3-(1-(2,5-dimethoxyphenyl)-2-(2-(2-(methylthio)phenyl)pyrrolidin-1-yl)-2-oxoethyl)ureido)methyl)phenyl)carbamate**

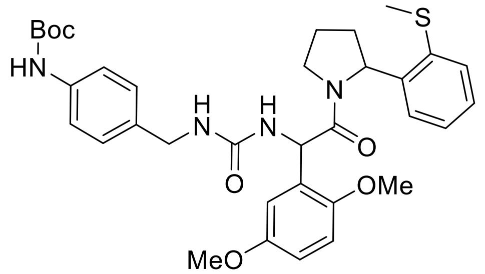


71 mg (0.32 mmol, 1 eq) of tert-butyl (4-(aminomethyl)phenyl)carbamate is dissolved in 2 mL of dry DCM under Argon. 55 µL (0.32 mmol, 1 eq) of DIEA and 52 mg (0.32 mmol, 1 eq) of CDI are added and the solution is stirred 15min at room temperature. 124 mg (0.32 mmol, 1 eq) of 2-amino-2-(2,5-dimethoxyphenyl)-1-(2-(2-(methylthio)phenyl)pyrrolidin-1-yl)ethan-1-one (described in previous step) dissolved in 2 mL of dry DCM is added. The solution is heated at 40°C overnight. Reaction is diluted with EtOAc and washed 3 times with water. Organic layer is dried over $Na_2SO_4$, filtered and evaporated unde reduced pressure. Crude product is purified by flash chromatography using a silica gel column and an DCM/MeOH mixture as eluent. 150 mg of the title compound is obtained as a pale yellow oil.

Yield : 74%

MH+ : 635.6 (M+1)

**Step 7: 1-(4-aminobenzyl)-3-(1-(2,5-dimethoxyphenyl)-2-(2-(2-(methylthio)phenyl)pyrrolidin-1-yl)-2-oxoethyl)urea C90**

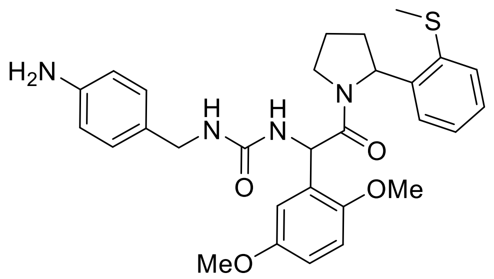


150 mg (0.23 mmol, 1 eq) of tert-butyl (4-((3-(1-(2,5-dimethoxyphenyl)-2-(2-(2-(methylthio)phenyl)pyrrolidin-1-yl)-2-oxoethyl)ureido)methyl)phenyl)carbamate (described in the previous step) is dissolved in 2 mL of dry DCM under Argon. Then 1 mL of TFA is added and the solution is stirred 1h at room temperature. Reaction is evaporated under reduced pressure, diluted with a saturated $NaHCO_3$ solution and extracted 3 times with DCM. Combined organic layers are dried over $Na_2SO_4$, filtered and evaporated unde reduced pressure. Crude product is purified by flash chromatography using a C18 column and an $H_2O$/MeOH mixture as eluent. 70 mg of the title compound is obtained as a pale yellow powder.

Yield : 55%

MH+ : 535.6 (M+1)

**Example 2: Synthesis of 1-(1-(2,5-dimethoxyphenyl)-2-(2-(2-(methylthio)phenyl)pyrrolidin-1-yl)-2-oxoethyl)-3-((1,2,3,4-tetrahydroquinolin-6-yl)methyl)urea: C173**

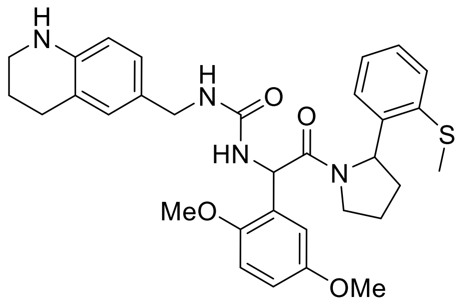


**Step 1: tert-butyl 6-((3-(1-(2,5-dimethoxyphenyl)-2-(2-(2-(methylthio)phenyl)pyrrolidin-1-yl)-2-oxoethyl)ureido)methyl)-3,4-dihydroquinoline-1(2H)-carboxylate**

The compound is obtained by the procedure described in Example 1, Step 6, starting from 74 mg (0.19 mmol) of 2-amino-2-(2,5-dimethoxyphenyl)-1-(2-(2-(methylthio)phenyl)pyrrolidin-1-yl)ethan-1-one (described in Example 1, Step 5) and tert-butyl 6-(aminomethyl)-3,4-dihydroquinoline-1(2H)-carboxylate instead of tert-butyl (4-(aminomethyl)phenyl)carbamate. The title compound was directly engaged in next step.

Yield: 52%

MH+ : 675.6 (M+1)

**Step 2: 1-(1-(2,5-dimethoxyphenyl)-2-(2-(2-(methylthio)phenyl)pyrrolidin-1-yl)-2-oxoethyl)-3-((1,2,3,4-tetrahydroquinolin-6-yl)methyl)urea**

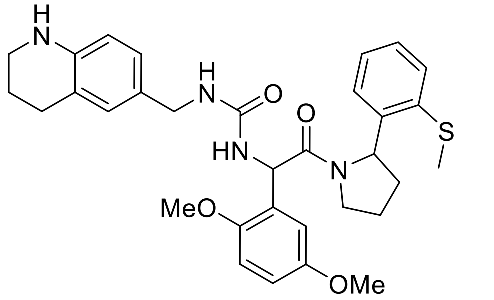


The compound is obtained by the procedure described in Example 1, Step 7, starting from crude of tert-butyl 6-((3-(1-(2,5-dimethoxyphenyl)-2-(2-(2-(methylthio)phenyl)pyrrolidin-1-yl)-2-oxoethyl)ureido)methyl)-3,4-dihydroquinoline-1(2H)-carboxylate (described in the previous step). 6 mg of the title compound are obtained as a white powder.

Yield: 10%

MH+ : 575.5 (M+1)

**Example 3: Synthesis of 1-((1H-indol-4-yl)methyl)-3-(1-(2,5-dimethoxyphenyl)-2-(2-(2-(methylthio)phenyl)pyrrolidin-1-yl)-2-oxoethyl)urea: C170**

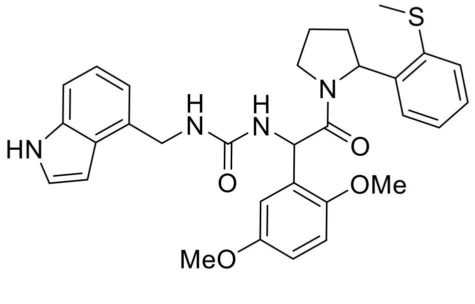

**Step 1: 1-((1H-indol-4-yl)methyl)-3-(1-(2,5-dimethoxyphenyl)-2-(2-(2-(methylthio)phenyl)pyrrolidin-1-yl)-2-oxoethyl)urea**

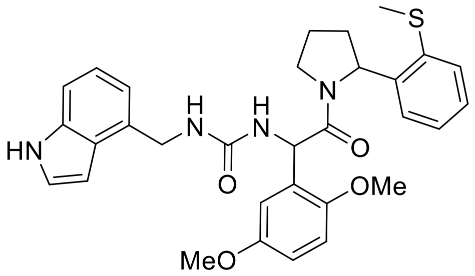

The compound is obtained by the procedure described in Example 1, Step 6, starting from 132 mg (0.34 mmol) of 2-amino-2-(2,5-dimethoxyphenyl)-1-(2-(2-(methylthio)phenyl)pyrrolidin-1-yl)ethan-1-one (described in Example 1, Step 5) and (1H-indol-4-yl)methanamine instead of tert-butyl (4-(aminomethyl)phenyl)carbamate. 32 mg of the title compound are obtained as a white powder.

Yield: 17%

MH+ : 559.3 (M+1)

**<u>Example 4: Synthesis of 1-((1H-indazol-4-yl)methyl)-3-(1-(2,5-dimethoxyphenyl)-2-(2-(2-(methylthio)phenyl)pyrrolidin-1-yl)-2-oxoethyl)urea: C171</u>**

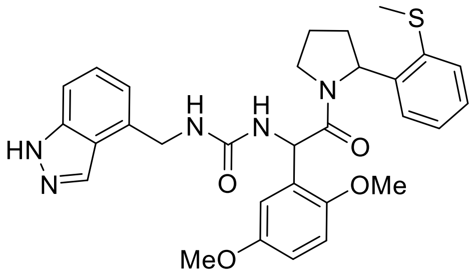

**Step 1: 1-((1H-indazol-4-yl)methyl)-3-(1-(2,5-dimethoxyphenyl)-2-(2-(2-(methylthio)phenyl)pyrrolidin-1-yl)-2-oxoethyl)urea**

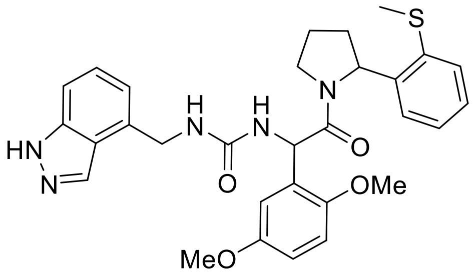

The compound is obtained by the procedure described in Example 1, Step 6, starting from 130 mg (0.34 mmol) of 2-amino-2-(2,5-dimethoxyphenyl)-1-(2-(2-(methylthio)phenyl)pyrrolidin-1-yl)ethan-1-one (described in Example 1, Step 5) and (1H-indazol-4-yl)methanamine instead of tert-butyl (4-(aminomethyl)phenyl)carbamate. 5 mg of the title compound are obtained as a white powder.

Yield: 3%

MH+ : 560.3 (M+1)

**Example 5: Synthesis of 1-(4-aminobenzyl)-3-(2-(2-(benzo[b]thiophen-7-yl)pyrrolidin-1-yl)-1-(2,5-dimethoxyphenyl)-2-oxoethyl)urea:F834 C108**

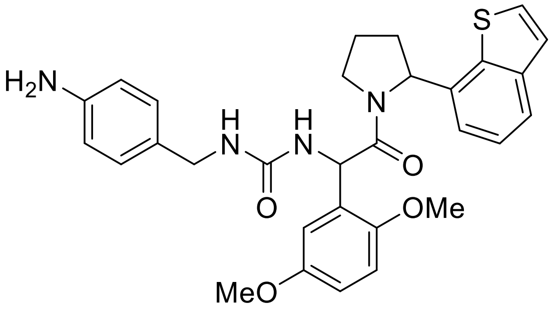

**Step 1: tert-butyl (2-(2-(benzo[b]thiophen-7-yl)pyrrolidin-1-yl)-1-(2,5-dimethoxyphenyl)-2-oxoethyl)carbamate**

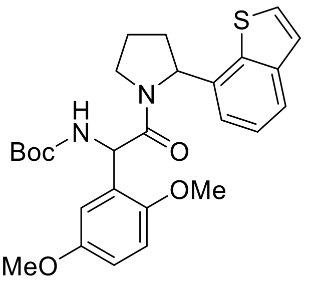

The compound is obtained by the procedure described in Example 1, Step 4, starting from 540 mg (1.73 mmol) of 2-((tert-butoxycarbonyl)amino)-2-(2,5-dimethoxyphenyl)acetic acid (described in Example 1, Step 3) and 2-(benzo[b]thiophen-7-yl)pyrrolidine instead of 2-(2-(methylthio)phenyl)pyrrolidine. 737 mg of the title compound are obtained as a white oil.

Yield: 86%

MH+ : 497.6 (M+1)

**Step 2: 2-amino-1-(2-(benzo[b]thiophen-7-yl)pyrrolidin-1-yl)-2-(2,5-dimethoxyphenyl)ethan-1-one**

The compound is obtained by the procedure described in Example 1, Step 5, starting from 700 mg (1.41 mmol) of tert-butyl (2-(2-(benzo[b]thiophen-7-yl)pyrrolidin-1-yl)-1-(2,5-

dimethoxyphenyl)-2-oxoethyl)carbamate (described in previous step). 348 mg of the title compound are obtained as a white powder.

Yield: 62%

MH+ : 397.5 (M+1)

**Step 3: tert-butyl (4-((3-(2-(2-(benzo[b]thiophen-7-yl)pyrrolidin-1-yl)-1-(2,5-dimethoxyphenyl)-2-oxoethyl)ureido)methyl)phenyl)carbamate**

The compound is obtained by the procedure described in Example 1, Step 6, starting from 170 mg (0.428 mmol) of 2-amino-1-(2-(benzo[b]thiophen-7-yl)pyrrolidin-1-yl)-2-(2,5-dimethoxyphenyl)ethan-1-one (described in previous step). 52 mg of the title compound are obtained as a white powder.

Yield: 19%

MH+ : 645.6 (M+1)

**Step 4: 1-(4-aminobenzyl)-3-(2-(2-(benzo[b]thiophen-7-yl)pyrrolidin-1-yl)-1-(2,5-dimethoxyphenyl)-2-oxoethyl)urea**

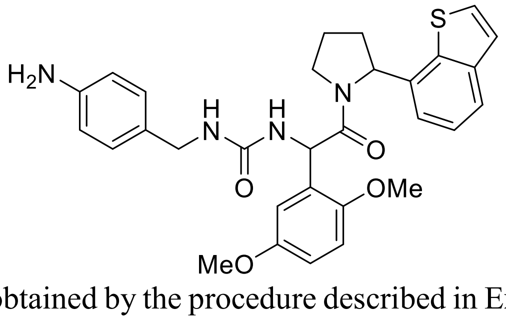


The compound is obtained by the procedure described in Example 1, Step 7, starting from 52 mg (0.08 mmol) of tert-butyl (4-((3-(2-(2-(benzo[b]thiophen-7-yl)pyrrolidin-1-yl)-1-(2,5-dimethoxyphenyl)-2-oxoethyl)ureido)methyl)phenyl)carbamate (described in previous step). 32 mg of the title compound are obtained as a white powder.

Yield: 73%

MH+ : 545.6 (M+1)

**Example 6: Synthesis of 1-(4-aminobenzyl)-3-(2-(2-(benzo[b]thiophen-7-yl)pyrrolidin-1-yl)-1-(5-bromo-2,4-dimethoxyphenyl)-2-oxoethyl)urea: C112**

**Step 1: methyl 2-amino-2-(5-bromo-2,4-dimethoxyphenyl)acetate**

The compound is obtained by the procedure described in Example 1, Step 1, starting from 500 mg (1.7 mmol) of 2-amino-2-(5-bromo-2,4-dimethoxyphenyl)acetic acid instead of 2-amino-2-(2,5-dimethoxyphenyl)acetic acid. 335 mg of the title compound are obtained as a pale yellow oil.

Yield: 64%.

MH+ : 304.3-306.3 (M+1)

**Step 2: methyl 2-(5-bromo-2,4-dimethoxyphenyl)-2-((tert-butoxycarbonyl)amino)acetate**

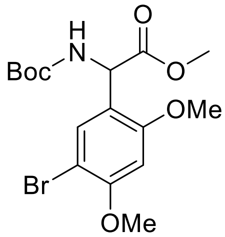


The compound is obtained by the procedure described in Example 1, Step 2, starting from 335 mg (1.1 mmol) of methyl 2-amino-2-(5-bromo-2,4-dimethoxyphenyl)acetate (described in the previous step). 432 mg of the title compound are obtained as a yellow solid foam.

Yield: 97%.

MH+ : 404.4-406.5 (M+1)

**Step 3: 2-(5-bromo-2,4-dimethoxyphenyl)-2-((tert-butoxycarbonyl)amino)acetic acid**

The compound is obtained by the procedure described in Example 1, Step 3, starting from 400 mg (0.99 mmol) of methyl 2-(5-bromo-2,4-dimethoxyphenyl)-2-((tert-butoxycarbonyl)amino)acetate (described in the previous step). 437 mg of the title compound are obtained as a white solid foam.

Yield: quant.

MH+ : 390.4-392.4 (M+1)

**Step 4: tert-butyl (2-(2-(benzo[b]thiophen-7-yl)pyrrolidin-1-yl)-1-(5-bromo-2,4-dimethoxyphenyl)-2-oxoethyl)carbamate**

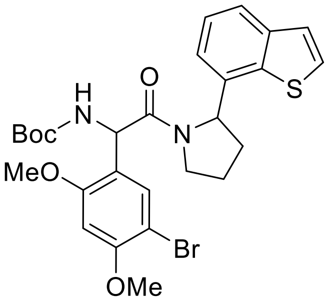


The compound is obtained by the procedure described in Example 1, Step 4, starting from 208 mg (0.53 mmol) of 2-(5-bromo-2,4-dimethoxyphenyl)-2-((tert-butoxycarbonyl)amino)acetic acid (described in the previous step) and 2-(benzo[b]thiophen-7-yl)pyrrolidine instead of 2-(2-(methylthio)phenyl)pyrrolidine. 226 mg of the title compound are obtained as a white solid foam.

Yield: 74%

MH+ : 575.5-577.5 (M+1)

**Step 5: 2-amino-1-(2-(benzo[b]thiophen-7-yl)pyrrolidin-1-yl)-2-(5-bromo-2,4-dimethoxyphenyl)ethan-1-one**

The compound is obtained by the procedure described in Example 1, Step 5, starting from 226 mg (0.39 mmol) of tert-butyl (2-(2-(benzo[b]thiophen-7-yl)pyrrolidin-1-yl)-1-(5-bromo-2,4-dimethoxyphenyl)-2-oxoethyl)carbamate (described in the previous step). 129 mg of the title compound are obtained as a white solid foam.

Yield: quant.

MH+ : 475.4-477.4 (M+1)

**Step 6: tert-butyl (4-((3-(2-(2-(benzo[b]thiophen-7-yl)pyrrolidin-1-yl)-1-(5-bromo-2,4-dimethoxyphenyl)-2-oxoethyl)ureido)methyl)phenyl)carbamate**

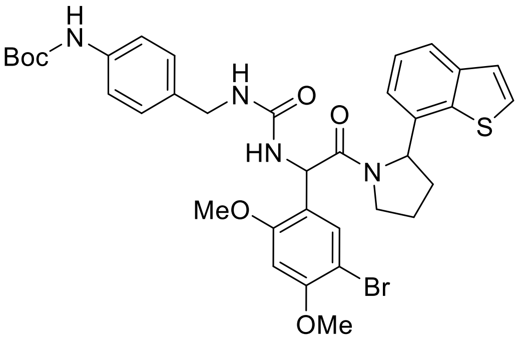


The compound is obtained by the procedure described in Example 1, Step 6, starting from 129 mg (0.27 mmol) of 2-amino-1-(2-(benzo[b]thiophen-7-yl)pyrrolidin-1-yl)-2-(5-bromo-2,4-dimethoxyphenyl)ethan-1-one (described in the previous step). 164 mg of the title compound are obtained as a white solid.

Yield: 84%

MH+ : 723.5-725.5 (M+1)

**Step 7: 1-(4-aminobenzyl)-3-(2-(2-(benzo[b]thiophen-7-yl)pyrrolidin-1-yl)-1-(5-bromo-2,4-dimethoxyphenyl)-2-oxoethyl)urea**

The compound is obtained by the procedure described in Example 1, Step 7, starting from 164 mg (0.23 mmol) of tert-butyl (4-((3-(2-(2-(benzo[b]thiophen-7-yl)pyrrolidin-1-yl)-1-(5-bromo-2,4-dimethoxyphenyl)-2-oxoethyl)ureido)methyl)phenyl)carbamate (described in the previous step). 47 mg of the title compound are obtained as an off white solid.

Yield: 33%

MH+ : 623.6-625.5 (M+1)

**<u>Example 7: Synthesis of 1-(4-aminobenzyl)-3-(2-(2-(benzo[b]thiophen-7-yl)pyrrolidin-1-yl)-1-cyclohexyl-2-oxoethyl)urea: C107</u>**

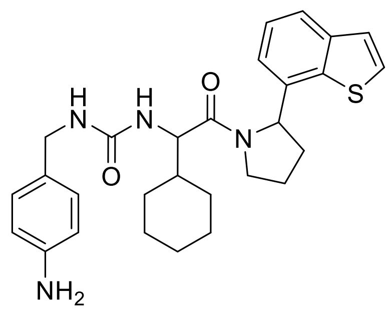

**Step 1: tert-butyl (2-(2-(benzo[b]thiophen-7-yl)pyrrolidin-1-yl)-1-cyclohexyl-2-oxoethyl)carbamate**

The compound is obtained by the procedure described in Example 1, Step 4, starting from 100 mg (0.39 mmol) of 2-((tert-butoxycarbonyl)amino)-2-cyclohexylacetic acid instead of 2-((tert-butoxycarbonyl)amino)-2-(2,5-dimethoxyphenyl)acetic acid and 2-(benzo[b]thiophen-7-yl)pyrrolidine instead of 2-(2-(methylthio)phenyl)pyrrolidine. 177 mg of the title compound are obtained as a colorless oil.

Yield: quant.

MH+ : 443.7 (M+1)

**Step 2: 2-amino-1-(2-(benzo[b]thiophen-7-yl)pyrrolidin-1-yl)-2-cyclohexylethan-1-one**

The compound is obtained by the procedure described in Example 1, Step 5, starting from 172 mg (0.39 mmol) of tert-butyl (2-(2-(benzo[b]thiophen-7-yl)pyrrolidin-1-yl)-1-cyclohexyl-2-oxoethyl)carbamate (described in the previous step). 89 mg of the title compound are obtained as a yellow oil.

Yield: 67%

MH+ : 343.8 (M+1)

**Step 3: tert-butyl (4-((3-(2-(2-(benzo[b]thiophen-7-yl)pyrrolidin-1-yl)-1-cyclohexyl-2-oxoethyl)ureido)methyl)phenyl)carbamate**

The compound is obtained by the procedure described in Example 1, Step 6, starting from 89 mg (0.26 mmol) of 2-amino-1-(2-(benzo[b]thiophen-7-yl)pyrrolidin-1-yl)-2-cyclohexylethan-1-one (described in the previous step). 157 mg of the title compound are obtained as a white solid.

Yield: quant.

MH+ : 591.7 (M+1)

**Step 4: 1-(4-aminobenzyl)-3-(2-(2-(benzo[b]thiophen-7-yl)pyrrolidin-1-yl)-1-cyclohexyl-2-oxoethyl)urea**

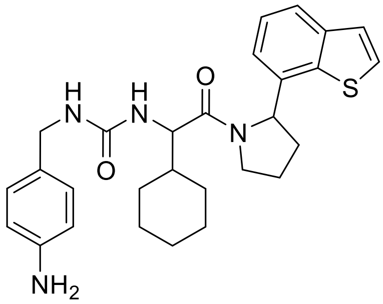

The compound is obtained by the procedure described in Example 1, Step 7, starting from 157 mg (0.26 mmol) of tert-butyl (4-((3-(2-(2-(benzo[b]thiophen-7-yl)pyrrolidin-1-yl)-1-cyclohexyl-2-oxoethyl)ureido)methyl)phenyl)carbamate (described in the previous step). 88 mg of the title compound are obtained as a slightly yellow solid.

Yield: 69%

MH+ : 491.6 (M+1)

<u>**Example 8: Synthesis of 1-(2-(2-(benzo[b]thiophen-7-yl)pyrrolidin-1-yl)-1-cyclohexyl-2-oxoethyl)-3-((1,2,3,4-tetrahydroquinolin-6-yl)methyl)urea: C106**</u>

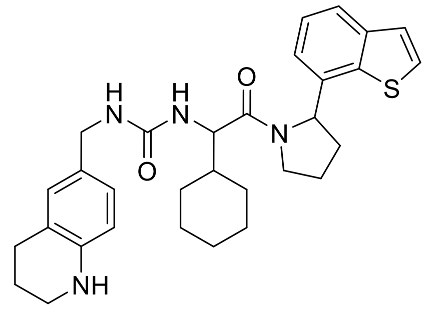

**Step 1: tert-butyl 6-((3-(2-(2-(benzo[b]thiophen-7-yl)pyrrolidin-1-yl)-1-cyclohexyl-2-oxoethyl)ureido)methyl)-3,4-dihydroquinoline-1(2H)-carboxylate**

The compound is obtained by the procedure described in Example 1, Step 6, starting from 459 mg (1.75 mmol) of 2-amino-1-(2-(benzo[b]thiophen-7-yl)pyrrolidin-1-yl)-2-cyclohexylethan-1-one (described in Example 8, Step 2). 1.05 g of the title compound are obtained as a colorless gel.

Yield: 95%

MH+ : 631.8 (M+1)

**Step 2: 1-(2-(2-(benzo[b]thiophen-7-yl)pyrrolidin-1-yl)-1-cyclohexyl-2-oxoethyl)-3-((1,2,3,4-tetrahydroquinolin-6-yl)methyl)urea**

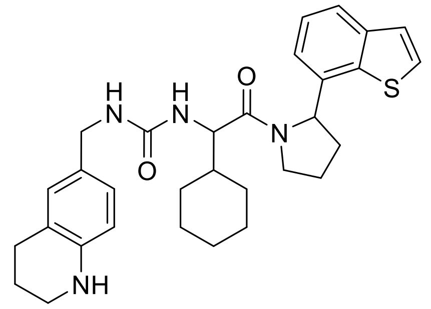

The compound is obtained by the procedure described in Example 1, Step 7, starting from 1.05 g (1.66 mmol) of tert-butyl 6-((3-(2-(2-(benzo[b]thiophen-7-yl)pyrrolidin-1-yl)-1-cyclohexyl-2-oxoethyl)ureido)methyl)-3,4-dihydroquinoline-1(2H)-carboxylate (described in the previous step). 515 mg of the title compound are obtained as a white powder.

Yield: 58%

MH+ : 531.8 (M+1)

**Example 9: Synthesis of 1-(4-aminobenzyl)-3-(1-cyclohexyl-2-(2-(2-(methylthio)phenyl)pyrrolidin-1-yl)-2-oxoethyl)urea: C71**

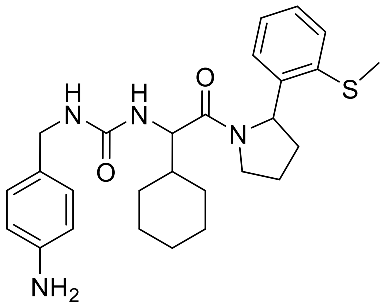


**Step 1: tert-butyl (1-cyclohexyl-2-(2-(2-(methylthio)phenyl)pyrrolidin-1-yl)-2-oxoethyl)carbamate**

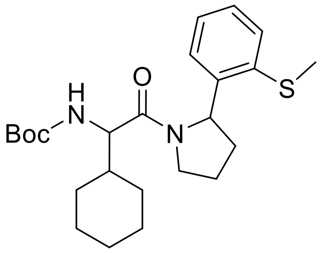


The compound is obtained by the procedure described in Example 1, Step 4, starting from 148 mg (0.58 mmol) of 2-((tert-butoxycarbonyl)amino)-2-cyclohexylacetic acid instead of 2-((tert-butoxycarbonyl)amino)-2-(2,5-dimethoxyphenyl)acetic acid. 125 mg of the title compound are obtained as a white powder.

Yield: 51%

MH+ : 432.5 (M+1)

**Step 2: 2-amino-2-cyclohexyl-1-(2-(2-(methylthio)phenyl)pyrrolidin-1-yl)ethan-1-one**

The compound is obtained by the procedure described in Example 1, Step 5, starting from 121 mg (0.28 mmol) of tert-butyl (1-cyclohexyl-2-(2-(2-(methylthio)phenyl)pyrrolidin-1-yl)-2-oxoethyl)carbamate (described in the previous step). 86 mg of the title compound are obtained as a white powder.

Yield: 92%

MH+ : 332.5 (M+1)

**Step 3: tert-butyl (4-((3-(1-cyclohexyl-2-(2-(2-(methylthio)phenyl)pyrrolidin-1-yl)-2-oxoethyl)ureido)methyl)phenyl)carbamate**

The compound is obtained by the procedure described in Example 1, Step 6, starting from 79 mg (0.195 mmol) of 2-amino-2-cyclohexyl-1-(2-(2-(methylthio)phenyl)pyrrolidin-1-yl)ethan-1-one (described in the previous step). 91 mg of the title compound are obtained as a white powder.

Yield: 80%

MH+ : 581.8 (M+1)

**Step 4: 1-(4-aminobenzyl)-3-(1-cyclohexyl-2-(2-(2-(methylthio)phenyl)pyrrolidin-1-yl)-2-oxoethyl)urea**

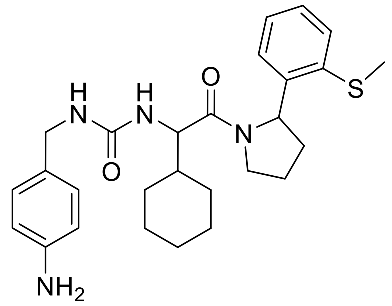


The compound is obtained by the procedure described in Example 1, Step 7, starting from 90 mg (0.155 mmol) of tert-butyl (4-((3-(1-cyclohexyl-2-(2-(2-(methylthio)phenyl)pyrrolidin-1-yl)-2-oxoethyl)ureido)methyl)phenyl)carbamate (described in the previous step). 38 mg of the title compound are obtained as a white powder.

Yield: 51%

MH+ : 481.6 (M+1)

**<u>Example 10: Synthesis of 1-(indolin-4-ylmethyl)-3-(2-(2-(2-(methylthio)phenyl)pyrrolidin-1-yl)-2-oxo-1-phenylethyl)urea: C144</u>**

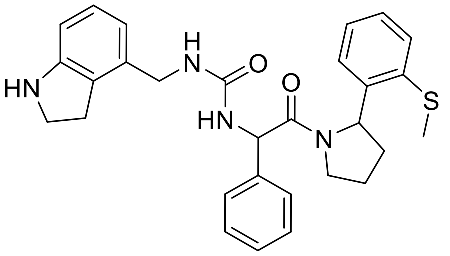


**Step 1: tert-butyl (2-(2-(2-(methylthio)phenyl)pyrrolidin-1-yl)-2-oxo-1-phenylethyl)carbamate**

The compound is obtained by the procedure described in Example 1, Step 4, starting from 200 mg (0.79 mmol) of 2-((tert-butoxycarbonyl)amino)-2-phenylacetic acid instead of 2-((tert-butoxycarbonyl)amino)-2-(2,5-dimethoxyphenyl)acetic acid. 264 mg of the title compound are obtained as a yellow gel.

Yield: 78%

MH+ : 427.5 (M+1)

**Step 2: 2-amino-1-(2-(2-(methylthio)phenyl)pyrrolidin-1-yl)-2-phenylethan-1-one**

The compound is obtained by the procedure described in Example 1, Step 5, starting from 264 mg (0.62 mmol) of tert-butyl (2-(2-(2-(methylthio)phenyl)pyrrolidin-1-yl)-2-oxo-1-phenylethyl)carbamate (described in the previous step). 250 mg of the title compound are obtained as a pale yellow gel.

Yield: quant.

MH+ : 327.6 (M+1)

**Step 3: tert-butyl 4-((3-(2-(2-(2-(methylthio)phenyl)pyrrolidin-1-yl)-2-oxo-1-phenylethyl)ureido)methyl)indoline-1-carboxylate**

The compound is obtained by the procedure described in Example 1, Step 6, starting from 51 mg (0.12 mmol) of 2-amino-1-(2-(2-(methylthio)phenyl)pyrrolidin-1-yl)-2-phenylethan-1-one (described in the previous step) and tert-butyl 4-(aminomethyl)indoline-1-carboxylate instead of tert-butyl (4-(aminomethyl)phenyl)carbamate. 67 mg of the title compound are obtained as a yellow solid.

Yield: 96%

MH+ : 601.8 (M+1)

**Step 4: 1-(indolin-4-ylmethyl)-3-(2-(2-(2-(methylthio)phenyl)pyrrolidin-1-yl)-2-oxo-1-phenylethyl)urea**

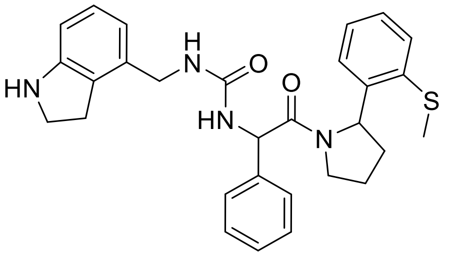


The compound is obtained by the procedure described in Example 1, Step 7, starting from 67 mg (0.11 mmol) of tert-butyl 4-((3-(2-(2-(2-(methylthio)phenyl)pyrrolidin-1-yl)-2-oxo-1-phenylethyl)ureido)methyl)indoline-1-carboxylate (described in the previous step). 40 mg of the title compound are obtained as a slightly yellow solid.

Yield: 71%

MH+ : 501.7 (M+1)

**<u>Example 11: Synthesis of 1-((2,3-dihydrobenzo[b][1,4]dioxin-5-yl)methyl)-3-(2-(2-(2-(methylthio)phenyl)pyrrolidin-1-yl)-2-oxo-1-phenylethyl)urea: C129</u>**

**Step 1: 1-((2,3-dihydrobenzo[b][1,4]dioxin-5-yl)methyl)-3-(2-(2-(2-(methylthio)phenyl)pyrrolidin-1-yl)-2-oxo-1-phenylethyl)urea**

The compound is obtained by the procedure described in Example 1, Step 6, starting from 200 mg (0.61 mmol) of 2-amino-1-(2-(2-(methylthio)phenyl)pyrrolidin-1-yl)-2-phenylethan-1-one (described in Example 11, Step 2) and (2,3-dihydrobenzo[b][1,4]dioxin-5-yl)methanamine instead of tert-butyl (4-(aminomethyl)phenyl)carbamate. 41 mg of the title compound are obtained as a white powder.

Yield: 13%

MH+ : 518.2 (M+1)

**Example 12: Synthesis of 1-((7-fluoro-2,3-dihydrobenzo[b][1,4]dioxin-5-yl)methyl)-3-(2-(2-(2-(methylthio)phenyl)pyrrolidin-1-yl)-2-oxo-1-phenylethyl)urea: C131**

**Step 1: 1-((7-fluoro-2,3-dihydrobenzo[b][1,4]dioxin-5-yl)methyl)-3-(2-(2-(2-(methylthio)phenyl)pyrrolidin-1-yl)-2-oxo-1-phenylethyl)urea**

The compound is obtained by the procedure described in Example 1, Step 6, starting from 200 mg (0.61 mmol) of 2-amino-1-(2-(2-(methylthio)phenyl)pyrrolidin-1-yl)-2-phenylethan-1-one (described in Example 11, Step 2) and (7-fluoro-2,3-dihydrobenzo[b][1,4]dioxin-5-yl)methanamine instead of tert-butyl (4-(aminomethyl)phenyl)carbamate. 32 mg of the title compound are obtained as a white powder.

Yield: 10%

MH+ : 536.2 (M+1)

**Example 13: Synthesis of 1-(benzo[b]thiophen-7-ylmethyl)-3-(2-(2-(2-(methylthio)phenyl)pyrrolidin-1-yl)-2-oxo-1-phenylethyl)urea: C142**

**Step 1: 1-(benzo[b]thiophen-7-ylmethyl)-3-(2-(2-(2-(methylthio)phenyl)pyrrolidin-1-yl)-2-oxo-1-phenylethyl)urea**

The compound is obtained by the procedure described in Example 1, Step 6, starting from 200 mg (0.61 mmol) of 2-amino-1-(2-(2-(methylthio)phenyl)pyrrolidin-1-yl)-2-phenylethan-1-one (described in Example 11, Step 2) and benzo[b]thiophen-7-ylmethanamine instead of tert-butyl (4-(aminomethyl)phenyl)carbamate. 127 mg of the title compound are obtained as a white powder.

Yield: 40%

MH+ : 516.7 (M+1)

**Example 14: Synthesis of 1-((2,2-difluorobenzo[d][1,3]dioxol-4-yl)methyl)-3-(2-(2-(2-(methylthio)phenyl)pyrrolidin-1-yl)-2-oxo-1-phenylethyl)urea: C143**

**Step 1: 1-((2,2-difluorobenzo[d][1,3]dioxol-4-yl)methyl)-3-(2-(2-(2-(methylthio)phenyl)pyrrolidin-1-yl)-2-oxo-1-phenylethyl)urea**

The compound is obtained by the procedure described in Example 1, Step 6, starting from 200 mg (0.61 mmol) of 2-amino-1-(2-(2-(methylthio)phenyl)pyrrolidin-1-yl)-2-phenylethan-1-one (described in Example 11, Step 2) and (2,2-difluorobenzo[d][1,3]dioxol-4-yl)methanamine instead of tert-butyl (4-(aminomethyl)phenyl)carbamate. 36 mg of the title compound are obtained as a white powder.

Yield: 11%

MH+ : 540.5 (M+1)

**Example 15: Synthesis of 1-(2-(2-(2-(methylthio)phenyl)pyrrolidin-1-yl)-2-oxo-1-phenylethyl)-3-((1,2,3,4-tetrahydroquinolin-6-yl)methyl)urea: C104**

**Step 1: tert-butyl 6-((3-(2-(2-(2-(methylthio)phenyl)pyrrolidin-1-yl)-2-oxo-1-phenylethyl)ureido)methyl)-3,4-dihydroquinoline-1(2H)-carboxylate**

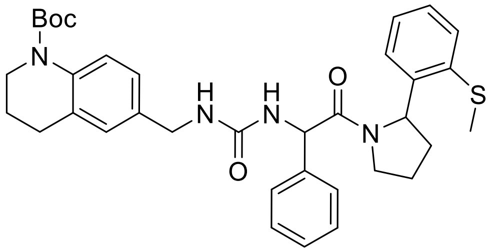


The compound is obtained by the procedure described in Example 1, Step 6, starting from 112 mg (0.34 mmol) of 2-amino-1-(2-(2-(methylthio)phenyl)pyrrolidin-1-yl)-2-phenylethan-1-one (described in Example 11, Step 2) and tert-butyl 6-(aminomethyl)-3,4-dihydroquinoline-1(2H)-carboxylate instead of tert-butyl (4-(aminomethyl)phenyl)carbamate. 165 mg of the title compound are obtained as a yellow oil.

Yield: 78%

MH+ : 615.5 (M+1)

**Step 2: 1-(2-(2-(2-(methylthio)phenyl)pyrrolidin-1-yl)-2-oxo-1-phenylethyl)-3-((1,2,3,4-tetrahydroquinolin-6-yl)methyl)urea**

The compound is obtained by the procedure described in Example 1, Step 7, starting from 165 mg (0.27 mmol) of tert-butyl 6-((3-(2-(2-(2-(methylthio)phenyl)pyrrolidin-1-yl)-2-oxo-1-phenylethyl)ureido)methyl)-3,4-dihydroquinoline-1(2H)-carboxylate (described in the previous step). 107 mg of the title compound are obtained as a slightly white powder.

Yield: 78%

MH+ : 515.6 (M+1)

**Example 16: Synthesis of 1-((1H-indol-4-yl)methyl)-3-(2-(2-(2-(methylthio)phenyl)pyrrolidin-1-yl)-2-oxo-1-phenylethyl)urea: C172**

**Step 1: 1-((1H-indol-4-yl)methyl)-3-(2-(2-(2-(methylthio)phenyl)pyrrolidin-1-yl)-2-oxo-1-phenylethyl)urea**

The compound is obtained by the procedure described in Example 1, Step 6, starting from 111 mg (0.34 mmol) of 2-amino-1-(2-(2-(methylthio)phenyl)pyrrolidin-1-yl)-2-phenylethan-1-one (described in Example 11, Step 2) and (1H-indol-4-yl)methanamine instead of tert-butyl (4-(aminomethyl)phenyl)carbamate. 11 mg of the title compound are obtained as a white powder.

Yield: 7%

MH+ : 499.5 (M+1)

**Example 17: Synthesis of 1-(5-aminoisoindolin-2-yl)-3-(2-(2-(2-(methylthio)phenyl)pyrrolidin-1-yl)-2-oxo-1-phenylethyl)urea: C185**

**Step 1: tert-butyl (2-(3-(2-(2-(2-(methylthio)phenyl)pyrrolidin-1-yl)-2-oxo-1-phenylethyl)ureido)isoindolin-5-yl)carbamate**

The compound is obtained by the procedure described in Example 1, Step 6, starting from 70 mg (0.21 mmol) of 2-amino-1-(2-(2-(methylthio)phenyl)pyrrolidin-1-yl)-2-phenylethan-1-one (described in Example 11, Step 2) and tert-butyl isoindolin-5-ylcarbamate instead of tert-butyl (4-(aminomethyl)phenyl)carbamate. 71 mg of the title compound are obtained as a slightly pink solid.

Yield: 57%

MH+ : 587.5 (M+1)

**Step 2: 1-(5-aminoisoindolin-2-yl)-3-(2-(2-(2-(methylthio)phenyl)pyrrolidin-1-yl)-2-oxo-1-phenylethyl)urea**

The compound is obtained by the procedure described in Example 1, Step 7, starting from 71 mg (0.12 mmol) of tert-butyl (2-(3-(2-(2-(2-(methylthio)phenyl)pyrrolidin-1-yl)-2-oxo-1-phenylethyl)ureido)isoindolin-5-yl)carbamate (described in the previous step). 2 mg of the title compound are obtained as a white solid.

Yield: 3%

MH+ : 487.5 (M+1)

**Example 18: Synthesis of 1-((1H-indazol-4-yl)methyl)-3-(2-(2-(2-(methylthio)phenyl)pyrrolidin-1-yl)-2-oxo-1-phenylethyl)urea: C178**

**Step 1: 1-((1H-indazol-4-yl)methyl)-3-(2-(2-(2-(methylthio)phenyl)pyrrolidin-1-yl)-2-oxo-1-phenylethyl)urea**

The compound is obtained by the procedure described in Example 1, Step 6, starting from 110 mg (0.34 mmol) of 2-amino-1-(2-(2-(methylthio)phenyl)pyrrolidin-1-yl)-2-phenylethan-1-one (described in Example 11, Step 2) and (1H-indazol-4-yl)methanamine instead of tert-butyl (4-(aminomethyl)phenyl)carbamate. 6 mg of the title compound are obtained as a white powder.

Yield: 4%

MH+ : 500.6 (M+1)

**<u>Example 19: Synthesis of 1-((8-hydroxyquinolin-5-yl)methyl)-3-(2-(2-(2-(methylthio)phenyl)pyrrolidin-1-yl)-2-oxo-1-phenylethyl)urea: C99</u>**

**Step 1: 1-((8-hydroxyquinolin-5-yl)methyl)-3-(2-(2-(2-(methylthio)phenyl)pyrrolidin-1-yl)-2-oxo-1-phenylethyl)urea**

The compound is obtained by the procedure described in Example 1, Step 6, starting from 265 mg (0.81 mmol) of 2-amino-1-(2-(2-(methylthio)phenyl)pyrrolidin-1-yl)-2-phenylethan-1-one (described in Example 11, Step 2) and 5-(aminomethyl)quinolin-8-ol instead of tert-butyl (4-(aminomethyl)phenyl)carbamate. 14 mg of the title compound are obtained as a white powder.

Yield: 3%

MH+ : 527.6 (M+1)

**Example 20: Synthesis of 1-(1-(4-amino-3-fluorophenyl)ethyl)-3-(2-(2-(2-(methylthio)phenyl)pyrrolidin-1-yl)-2-oxo-1-phenylethyl)urea: C101**

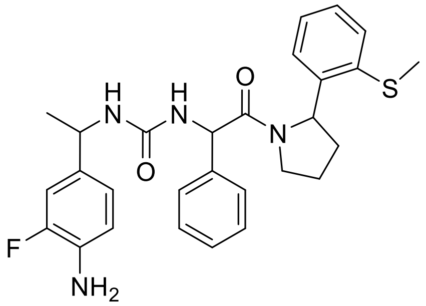


**Step 1: 1-(1-(4-amino-3-fluorophenyl)ethyl)-3-(2-(2-(2-(methylthio)phenyl)pyrrolidin-1-yl)-2-oxo-1-phenylethyl)urea**

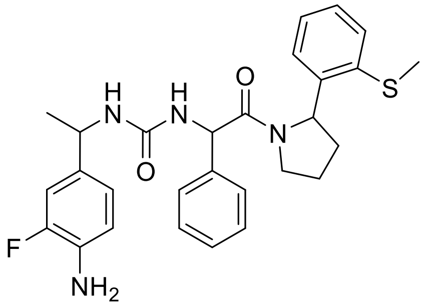


The compound is obtained by the procedure described in Example 1, Step 6, starting from 200 mg (0.61 mmol) of 2-amino-1-(2-(2-(methylthio)phenyl)pyrrolidin-1-yl)-2-phenylethan-1-one (described in Example 11, Step 2) and 4-(1-aminoethyl)-2-fluoroaniline instead of tert-butyl (4-(aminomethyl)phenyl)carbamate. 28 mg of the title compound are obtained as a white powder.

Yield: 9%

MH+ : 507.7 (M+1)

**Example 21: Synthesis of 1-((2-azaspiro[3.3]heptan-6-yl)methyl)-3-(2-(2-(2-(methylthio)phenyl)pyrrolidin-1-yl)-2-oxo-1-phenylethyl)urea: C119**

**Step 1: tert-butyl (4-((3-(2-(2-(2-(methylthio)phenyl)pyrrolidin-1-yl)-2-oxo-1-phenylethyl)ureido)methyl)2-azaspiro[3.3]heptan-6yl)carbamate**

The compound is obtained by the procedure described in Example 1, Step 6, starting from 200 mg (0.61 mmol) of 2-amino-1-(2-(2-(methylthio)phenyl)pyrrolidin-1-yl)-2-phenylethan-1-one (described in Example 11, Step 2). 119 mg of the title compound are obtained as a colorless gel.

Yield: 34%

MH+ : 579.8 (M+1)

**Step 2: 1-((2-azaspiro[3.3]heptan-6-yl)methyl)-3-(2-(2-(2-(methylthio)phenyl)pyrrolidin-1-yl)-2-oxo-1-phenylethyl)urea**

The compound is obtained by the procedure described in Example 1, Step 6, starting from 119 mg (0.206 mmol) of 2-amino-1-(2-(2-(methylthio)phenyl)pyrrolidin-1-yl)-2-phenylethan-1-one (described in Example 11, Step 2) and (2-azaspiro[3.3]heptan-6-yl)methanamine instead of tert-butyl (4-(aminomethyl)phenyl)carbamate. 76 mg of the title compound are obtained as a white powder.

Yield: 78%

MH+ : 479.7 (M+1)

**Example 22: Synthesis of 1-(((1r,4r)-4-aminocyclohexyl)methyl)-3-(2-(2-(2-(methylthio)phenyl)pyrrolidin-1-yl)-2-oxo-1-phenylethyl)urea: C120**

**Step 1: tert-butyl (4-((3-(2-(2-(2-(methylthio)phenyl)pyrrolidin-1-yl)-2-oxo-1-phenylethyl)ureido)methyl)-trans-cyclohexyl)carbamate**

The compound is obtained by the procedure described in Example 1, Step 6, starting from 200 mg (0.61 mmol) of 2-amino-1-(2-(2-(methylthio)phenyl)pyrrolidin-1-yl)-2-phenylethan-1-one (described in Example 11, Step 2). 320 mg of the title compound are obtained as a colorless gel.

Yield: 82%

MH+ : 597.8 (M+1)

**Step 2: 1-(((1r,4r)-4-aminocyclohexyl)methyl)-3-(2-(2-(2-(methylthio)phenyl)pyrrolidin-1-yl)-2-oxo-1-phenylethyl)urea**

The compound is obtained by the procedure described in Example 1, Step 6, starting from 300 mg (0.50 mmol) of 2-amino-1-(2-(2-(methylthio)phenyl)pyrrolidin-1-yl)-2-phenylethan-1-one (described in Example 11, Step 2) and (1r,4r)-4-(aminomethyl)cyclohexan-1-amine instead of tert-butyl (4-(aminomethyl)phenyl)carbamate. 86 mg of the title compound are obtained as a white powder.

Yield: 35%

MH+ : 497.7 (M+1)

**Example 23: Synthesis of N-ethyl-5-((3-(2-(2-(2-(methylthio)phenyl)pyrrolidin-1-yl)-2-oxo-1-phenylethyl)ureido)methyl)-3,4-dihydroquinoline-1(2H)-carboxamide:F903 C177**

**Step 1: tert-butyl 5-((3-(2-(2-(2-(methylthio)phenyl)pyrrolidin-1-yl)-2-oxo-1-phenylethyl)ureido)methyl)-3,4-dihydroquinoline-1(2H)-carboxylate**

The compound is obtained by the procedure described in Example 1, Step 6, starting from 62 mg (0.19 mmol) of 2-amino-1-(2-(2-(methylthio)phenyl)pyrrolidin-1-yl)-2-phenylethan-1-one (described in Example 11, Step 2) and tert-butyl 5-(aminomethyl)-3,4-dihydroquinoline-1(2H)-carboxylate instead of tert-butyl (4-(aminomethyl)phenyl)carbamate. 66 mg of the title compound are obtained as a white solid.

Yield: 56%

MH+ : 615.0 (M+1)

**Step 2: 1-(2-(2-(2-(methylthio)phenyl)pyrrolidin-1-yl)-2-oxo-1-phenylethyl)-3-((1,2,3,4-tetrahydroquinolin-5-yl)methyl)urea**

The compound is obtained by the procedure described in Example 1, Step 7, starting from 66 mg (0.11 mmol) of tert-butyl 5-((3-(2-(2-(2-(methylthio)phenyl)pyrrolidin-1-yl)-2-oxo-1-phenylethyl)ureido)methyl)-3,4-dihydroquinoline-1(2H)-carboxylate (described in the previous step). 18 mg of the title compound are obtained as a white solid.

Yield: 33%

MH+ : 515.4 (M+1)

**Step 3: N-ethyl-5-((3-(2-(2-(2-(methylthio)phenyl)pyrrolidin-1-yl)-2-oxo-1-phenylethyl)ureido)methyl)-3,4-dihydroquinoline-1(2H)-carboxamide**

15 mg (0.03 mmol, 1 eq) of 1-(2-(2-(2-(methylthio)phenyl)pyrrolidin-1-yl)-2-oxo-1-phenylethyl)-3-((1,2,3,4-tetrahydroquinolin-5-yl)methyl)urea (described in previous step), is dissolved in 500 µL of dry DCM under Argon. 3 µL (0.03 mmol, 1 eq) of ethyl isocyanate is added and the solution is stirred overnight at room temperature. Reaction is dried under reduced pressure. 17 mg of the title compound is obtained as an off white powder.

Yield : quant.

MH+ : 586.5 (M+1)

**<u>Example 24: Synthesis of 1-((1-acetyl-1,2,3,4-tetrahydroquinolin-5-yl)methyl)-3-(2-(2-(2-(methylthio)phenyl)pyrrolidin-1-yl)-2-oxo-1-phenylethyl)urea: C179</u>**

**Step 1: 1-((1-acetyl-1,2,3,4-tetrahydroquinolin-5-yl)methyl)-3-(2-(2-(2-(methylthio)phenyl)pyrrolidin-1-yl)-2-oxo-1-phenylethyl)urea**

15 mg (0.03 mmol, 1 eq) of 1-(2-(2-(2-(methylthio)phenyl)pyrrolidin-1-yl)-2-oxo-1-phenylethyl)-3-((1,2,3,4-tetrahydroquinolin-5-yl)methyl)urea (described in Example 25, Step 2), is dissolved in 500 µL of dry DCM under Argon. The solution is cooled to 0°C, then 4.7 µL (0.06 mmol, 2 eq) of pyridine and 2.5 µL (0.04 mmol, 1.2 eq) are added. The mixture is stirred 24h at room temperature. Reaction is diluted with a saturated NH4Cl solution and extracted 2 times with EtOAc. Combined organic layers are dried over $Na_2SO_4$, filtered and evaporated unde reduced pressure. Crude compound is triturated in Hexane, the solid is filtered and dried under vacuum to give 1.2 mg of a white powder.

Yield : 8%

MH+ : 557.5 (M+1)

**Example 25: Synthesis of 1-(4-((2-fluorobenzyl)amino)benzyl)-3-(2-(2-(2-(methylthio)phenyl)pyrrolidin-1-yl)-2-oxo-1-phenylethyl)urea: C182**

**Step 1: 1-(4-((2-fluorobenzyl)amino)benzyl)-3-(2-(2-(2-(methylthio)phenyl)pyrrolidin-1-yl)-2-oxo-1-phenylethyl)urea**

100 mg (0.21 mmol, 1 eq) of 1-(4-aminobenzyl)-3-(2-(2-(2-(methylthio)phenyl)pyrrolidin-1-yl)-2-oxo-1-phenylethyl)urea (described in Example 20, Step 2) and 22µL (0.21 mmol, 1 eq) of 2-fluorobenzaldehyde are dissolved in 4 mL of dry MeOH under Argon. 12µL (0.21 mmol, 1 eq) of AcOH are added and the solution is stirred 10min at room temperature. 8 mg (0.21 mmol, 1 eq) of $NaBH_4$ are added and reaction is stirred overnight at room temperature. Reaction is diluted with water and extracted 2 times with DCM. Combined organic layers are washed with brine, dried over $Na_2SO_4$, filtered and evaporated unde reduced pressure. Crude product is purified by flash chromatography using a silica gel column and an Hexane/EtOAc mixture as eluent. 14 mg of the title compound is obtained as a pale colorless oil.

Yield : 11%

MH+ : 583.5 (M+1)

**Example 26: Synthesis of 1-(2-(2-(2-(methylthio)phenyl)pyrrolidin-1-yl)-2-oxo-1-phenylethyl)-3-(4-((piperidin-3-ylmethyl)amino)benzyl)urea: C183**

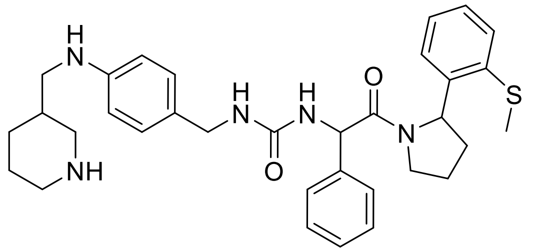

**Step 1: tert-butyl 3-(((4-((3-(2-(2-(2-(methylthio)phenyl)pyrrolidin-1-yl)-2-oxo-1-phenylethyl)ureido)methyl)phenyl)amino)methyl)piperidine-1-carboxylate**

The compound is obtained by the procedure described in Example 27, Step 1, starting from 35 mg (0.07 mmol) of 1-(4-aminobenzyl)-3-(2-(2-(2-(methylthio)phenyl)pyrrolidin-1-yl)-2-oxo-1-phenylethyl)urea (described in Example 20, Step 2) and tert-butyl 3-formylpiperidine-1-carboxylate instead of 2-fluorobenzaldehyde. 30 mg of the title compound are obtained as a white powder.

Yield: 60%

MH+ : 672.7 (M+1)

**Step 2: 1-(2-(2-(2-(methylthio)phenyl)pyrrolidin-1-yl)-2-oxo-1-phenylethyl)-3-(4-((piperidin-3-ylmethyl)amino)benzyl)urea**

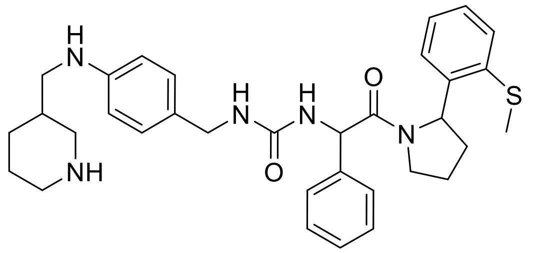

The compound is obtained by the procedure described in Example 1, Step 7, starting from 30 mg (0.04 mmol) of tert-butyl 3-(((4-((3-(2-(2-(2-(methylthio)phenyl)pyrrolidin-1-yl)-2-oxo-1-phenylethyl)ureido)methyl)phenyl)amino)methyl)piperidine-1-carboxylate (described in the previous step). 8 mg of the title compound are obtained as a pale yellow powder.

Yield: 31%

MH+ : 572.7 (M+1)

**Example 27: Synthesis of 1-((5-aminopyridin-2-yl)methyl)-3-(1-(5-bromo-2-methoxyphenyl)-2-(2-(2-(methylthio)phenyl)pyrrolidin-1-yl)-2-oxoethyl)urea: C115**

**Step 1: tert-butyl (1-(5-bromo-2-methoxyphenyl)-2-(2-(2-(methylthio)phenyl)pyrrolidin-1-yl)-2-oxoethyl)carbamate**

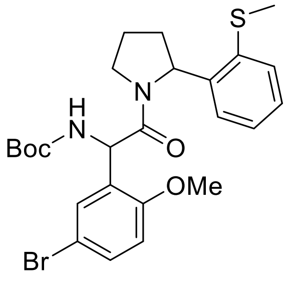

The compound is obtained by the procedure described in Example 1, Step 4, starting from 400 mg (1.12 mmol) of 2-(5-bromo-2-methoxyphenyl)-2-((tert-butoxycarbonyl)amino)acetic acid instead of 2-((tert-butoxycarbonyl)amino)-2-(2,5-dimethoxyphenyl)acetic acid. 478 mg of the title compound are obtained as a colorless oil.

Yield: 80%

MH+ : 534.6-536.6 (M+1)

**Step 2: 2-amino-2-(5-bromo-2-methoxyphenyl)-1-(2-(2-(methylthio)phenyl)pyrrolidin-1-yl)ethan-1-one**

The compound is obtained by the procedure described in Example 1, Step 5, starting from 200 mg (0.37 mmol) of tert-butyl (1-(5-bromo-2-methoxyphenyl)-2-(2-(2-(methylthio)phenyl)pyrrolidin-1-yl)-2-oxoethyl)carbamate (described in the previous step). 117 mg of the title compound are obtained as a slightly green oil.

Yield: 72%

MH+ : 434.5-436.5 (M+1)

**Step 3: tert-butyl (6-((3-(1-(5-bromo-2-methoxyphenyl)-2-(2-(2-(methylthio)phenyl)pyrrolidin-1-yl)-2-oxoethyl)ureido)methyl)pyridin-3-yl)carbamate**

The compound is obtained by the procedure described in Example 1, Step 6, starting from 81 mg (0.19 mmol) of 2-amino-2-(5-bromo-2-methoxyphenyl)-1-(2-(2-(methylthio)phenyl)pyrrolidin-1-yl)ethan-1-one (described in the previous step) and tert-butyl (6-(aminomethyl)pyridin-3-yl)carbamate instead of tert-butyl (4-(aminomethyl)phenyl)carbamate. 64 mg of the title compound are obtained as a colorless oil.

Yield: 56%

MH+ : 684.6-686.5 (M+1)

**Step 4: 1-((5-aminopyridin-2-yl)methyl)-3-(1-(5-bromo-2-methoxyphenyl)-2-(2-(2-(methylthio)phenyl)pyrrolidin-1-yl)-2-oxoethyl)urea**

The compound is obtained by the procedure described in Example 1, Step 7, starting from 64 mg (0.093 mmol) of tert-butyl (6-((3-(1-(5-bromo-2-methoxyphenyl)-2-(2-(2-(methylthio)phenyl)pyrrolidin-1-yl)-2-oxoethyl)ureido)methyl)pyridin-3-yl)carbamate (described in the previous step).157 mg of the title compound are obtained as a white solid.

Yield: 27%

MH+ : 584.6-586.5 (M+1)

**Example 28: Synthesis of 1-(1-(4-amino-3-fluorophenyl)ethyl)-3-(1-(5-bromo-2-methoxyphenyl)-2-(2-(2-(methylthio)phenyl)pyrrolidin-1-yl)-2-oxoethyl)urea: C123**

**Step 1: 1-(1-(4-amino-3-fluorophenyl)ethyl)-3-(1-(5-bromo-2-methoxyphenyl)-2-(2-(2-(methylthio)phenyl)pyrrolidin-1-yl)-2-oxoethyl)urea**

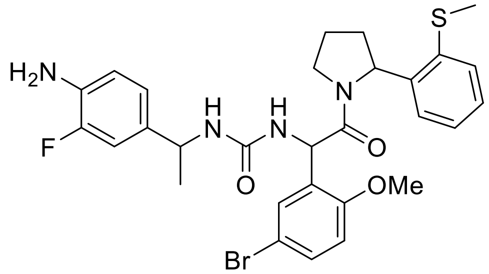


The compound is obtained by the procedure described in Example 1, Step 6, starting from 166 mg (0.38 mmol) of 2-amino-2-(5-bromo-2-methoxyphenyl)-1-(2-(2-(methylthio)phenyl)pyrrolidin-1-yl)ethan-1-one (described in Example 29, Step 2) and 4-(1-aminoethyl)-2-fluoroaniline instead of tert-butyl (4-(aminomethyl)phenyl)carbamate. 50 mg of the title compound are obtained as a white powder.

Yield: 22%

MH+ : 615.6-617.5 (M+1)

**Example 29: Synthesis of 1-(4-aminobenzyl)-3-(2-(2-(benzo[b]thiophen-7-yl)pyrrolidin-1-yl)-1-(5-bromo-2-methoxyphenyl)-2-oxoethyl)urea: C110**

**Step 1: tert-butyl (2-(2-(benzo[b]thiophen-7-yl)pyrrolidin-1-yl)-1-(5-bromo-2-methoxyphenyl)-2-oxoethyl)carbamate**

The compound is obtained by the procedure described in Example 1, Step 4, starting from 200 mg (0.56 mmol) of 2-(5-bromo-2-methoxyphenyl)-2-((tert-butoxycarbonyl)amino)acetic acid instead of 2-((tert-butoxycarbonyl)amino)-2-(2,5-dimethoxyphenyl)acetic acid and 2-(benzo[b]thiophen-7-yl)pyrrolidine instead of 2-(2-(methylthio)phenyl)pyrrolidine. 301 mg of the title compound are obtained as a colorless oil.

Yield: 99%

MH+ : 545.5-547.5 (M+1)

**Step 2: 2-amino-1-(2-(benzo[b]thiophen-7-yl)pyrrolidin-1-yl)-2-(5-bromo-2-methoxyphenyl)ethan-1-one**

The compound is obtained by the procedure described in Example 1, Step 5, starting from 301 mg (0.55 mmol) of tert-butyl (2-(2-(benzo[b]thiophen-7-yl)pyrrolidin-1-yl)-1-(5-bromo-2-methoxyphenyl)-2-oxoethyl)carbamate (described in the previous step). 176 mg of the title compound are obtained as a slightly green oil.

Yield: 72%

MH+ : 445.5-447.4 (M+1)

**Step 3: tert-butyl (4-((3-(2-(2-(benzo[b]thiophen-7-yl)pyrrolidin-1-yl)-1-(5-bromo-2-methoxyphenyl)-2-oxoethyl)ureido)methyl)phenyl)carbamate**

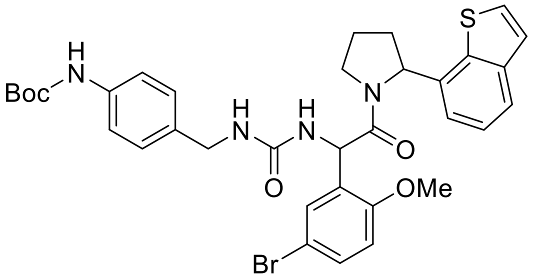


The compound is obtained by the procedure described in Example 1, Step 6, starting from 87 mg (0.19 mmol) of 2-amino-1-(2-(benzo[b]thiophen-7-yl)pyrrolidin-1-yl)-2-(5-bromo-2-methoxyphenyl)ethan-1-one (described in the previous step). 100 mg of the title compound are obtained as a colorless oil.

Yield: 74%

MH+ : 693.5-695.5 (M+1)

**Step 4: 1-(4-aminobenzyl)-3-(2-(2-(benzo[b]thiophen-7-yl)pyrrolidin-1-yl)-1-(5-bromo-2-methoxyphenyl)-2-oxoethyl)urea**

The compound is obtained by the procedure described in Example 1, Step 7, starting from 100 mg (0.14 mmol) of tert-butyl (4-((3-(2-(2-(benzo[b]thiophen-7-yl)pyrrolidin-1-yl)-1-(5-bromo-2-methoxyphenyl)-2-oxoethyl)ureido)methyl)phenyl)carbamate (described in the previous step). 57 mg of the title compound are obtained as a white solid.

Yield: 67%

MH+ : 593.5-595.5 (M+1)

**<u>Example 30: Synthesis of 1-(4-aminobenzyl)-3-((R)-2-((S)-2-(benzo[b]thiophen-7-yl)pyrrolidin-1-yl)-1-(5-bromo-2-methoxyphenyl)-2-oxoethyl)urea: C110RS</u>**

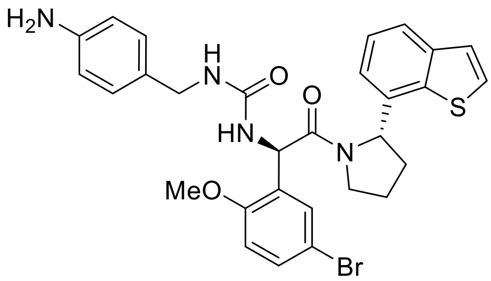

**Step 1: (R)-2-(5-bromo-2-methoxyphenyl)-2-((tert-butoxycarbonyl)amino)acetic acid**

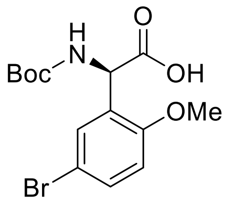


290 mg (0.98 mmol, 1 eq) of (R)-2-amino-2-(5-bromo-2-methoxyphenyl)acetic acid HCl are dissolved in 10 mL of a THF/$H_2O$ mixture (1/1, v/v). The solution is cooled to 0°C and 125 mg (3.13 mmol, 3.2 eq) of NaOH are added. The mixture is stirred 10 min at this temperature, then 213 mg (0.98 mmol, 1 eq) of $Boc_2O$ are added and reaction is stirred 40min at 0°C. Reaction is diluted with $H_2O$ and extracted 3 times with DCM. Aqueous layer is cooled to 0°C and acidified with HCl 37% until pH reached a value of 2. Aqueous layer is extracted 3 times with DCM and this organic layer is dried over $Na_2SO_4$, filtered and evaporated unde reduced pressure to give 411 mg of a colorless gel.

Yield : quant.

MH+ : 360.4-362.4 (M+1)

**Step 2: tert-butyl (R)-(2-(2-(benzo[b]thiophen-7-yl)pyrrolidin-1-yl)-1-(5-bromo-2-methoxyphenyl)-2-oxoethyl)carbamate**

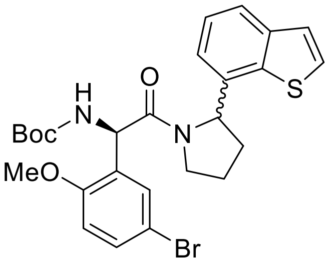


The compound is obtained by the procedure described in Example 1, Step 4, starting from 352 mg (0.98 mmol) of (R)-2-(5-bromo-2-methoxyphenyl)-2-((tert-butoxycarbonyl)amino)acetic acid (described in the previous step) and 2-(benzo[b]thiophen-7-yl)pyrrolidine instead of 2-(2-(methylthio)phenyl)pyrrolidine. 507 mg of the title compound are obtained as a yellow gel.

Yield: 95%

MH+ : 545.5-547.5 (M+1)

**Step 3: (R)-2-amino-1-(2-(benzo[b]thiophen-7-yl)pyrrolidin-1-yl)-2-(5-bromo-2-methoxyphenyl)ethan-1-one**

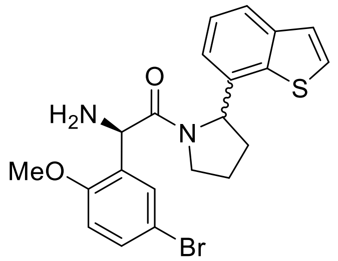


The compound is obtained by the procedure described in Example 1, Step 5, starting from 507 mg (0.93 mmol) of tert-butyl tert-butyl (R)-(2-(2-(benzo[b]thiophen-7-yl)pyrrolidin-1-yl)-1-(5-bromo-2-methoxyphenyl)-2-oxoethyl)carbamate (described in the previous step). 280 mg of the title compound are obtained as a yellow gel.

Yield: 68%

MH+ : 445.5-447.4 (M+1)

**Step 4: tert-butyl (R)-(4-((3-(2-(2-(benzo[b]thiophen-7-yl)pyrrolidin-1-yl)-1-(5-bromo-2-methoxyphenyl)-2-oxoethyl)ureido)methyl)phenyl)carbamate**

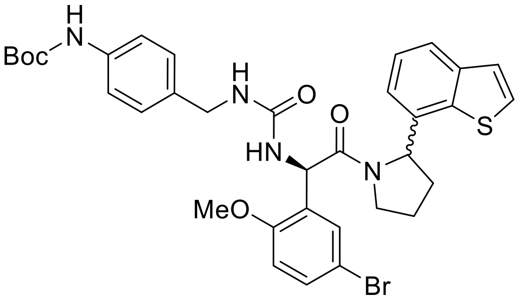


The compound is obtained by the procedure described in Example 1, Step 6, starting from 150 mg (0.34 mmol) of (R)-2-amino-1-(2-(benzo[b]thiophen-7-yl)pyrrolidin-1-yl)-2-(5-bromo-2-methoxyphenyl)ethan-1-one (described in the previous step). 201 mg of the title compound are obtained as a white solid.

Yield: 86%

MH+ : 693.5-695.5 (M+1)

**Step 5: (R)-1-(4-aminobenzyl)-3-(2-(2-(benzo[b]thiophen-7-yl)pyrrolidin-1-yl)-1-(5-bromo-2-methoxyphenyl)-2-oxoethyl)urea**

The compound is obtained by the procedure described in Example 1, Step 7, starting from 201 mg (0.29 mmol) of tert-butyl (R)-(4-((3-(2-(2-(benzo[b]thiophen-7-yl)pyrrolidin-1-yl)-1-(5-bromo-2-methoxyphenyl)-2-oxoethyl)ureido)methyl)phenyl)carbamate (described in the previous step). 125 mg of the title compound are obtained as a white solid.

Yield: 73%

MH+ : 593.5-595.5 (M+1)

**Step 6: 1-(4-aminobenzyl)-3-((R)-2-((S)-2-(benzo[b]thiophen-7-yl)pyrrolidin-1-yl)-1-(5-bromo-2-methoxyphenyl)-2-oxoethyl)urea**

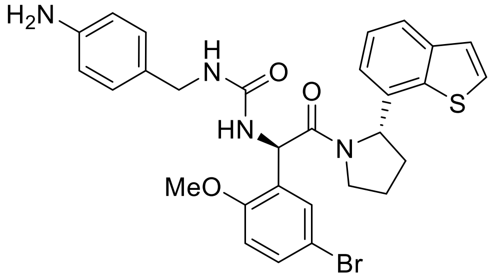


(R)-1-(4-aminobenzyl)-3-(2-(2-(benzo[b]thiophen-7-yl)pyrrolidin-1-yl)-1-(5-bromo-2-methoxyphenyl)-2-oxoethyl)urea (described in previous step) is separated by flash chromatography using a Chiralflash IG column and an EtOH/DCM mixture as the mobile phase. First fraction to be eluted is 1-(4-aminobenzyl)-3-((R)-2-((S)-2-(benzo[b]thiophen-7-yl)pyrrolidin-1-yl)-1-(5-bromo-2-methoxyphenyl)-2-oxoethyl)urea with ee > 98%. Product is purified by flash chromatography using a C18 column and an $H_2O$/MeOH mixture as eluent. 33 mg of the title compound is obtained as a white solid.

MH+ : 593.5-595.5 (M+1)

**<u>Example 31: Synthesis of 1-(4-aminobenzyl)-3-((R)-2-((R)-2-(benzo[b]thiophen-7-yl)pyrrolidin-1-yl)-1-(5-bromo-2-methoxyphenyl)-2-oxoethyl)urea: C110RR</u>**

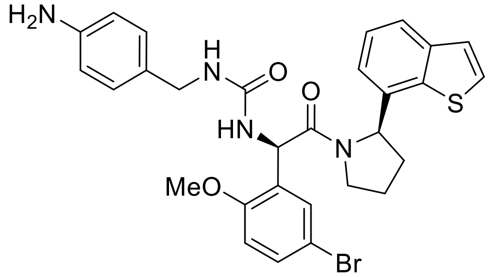

**Step 1: 1-(4-aminobenzyl)-3-((R)-2-((R)-2-(benzo[b]thiophen-7-yl)pyrrolidin-1-yl)-1-(5-bromo-2-methoxyphenyl)-2-oxoethyl)urea**

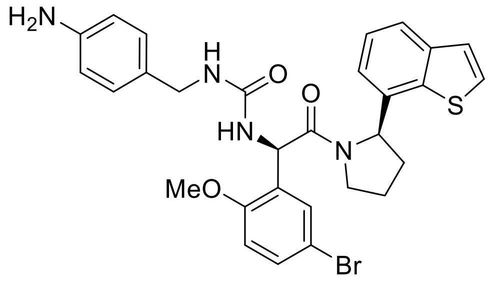


(R)-1-(4-aminobenzyl)-3-(2-(2-(benzo[b]thiophen-7-yl)pyrrolidin-1-yl)-1-(5-bromo-2-methoxyphenyl)-2-oxoethyl)urea (described in Example 32, Step 5) is separated by flash chromatography using a Chiralflash IG column and an EtOH/DCM mixture as the mobile phase. Second fraction to be eluted is 1-(4-aminobenzyl)-3-((R)-2-((R)-2-(benzo[b]thiophen-7-yl)pyrrolidin-1-yl)-1-(5-bromo-2-methoxyphenyl)-2-oxoethyl)urea with ee > 96%. Product is purified by flash chromatography using a C18 column and an $H_2O$/MeOH mixture as eluent. 48 mg of the title compound is obtained as a white solid.

MH+ : 593.5-595.5 (M+1)

**Example 32: Synthesis of 1-(4-aminobenzyl)-3-((S)-2-((R)-2-(benzo[b]thiophen-7-yl)pyrrolidin-1-yl)-1-(5-bromo-2-methoxyphenyl)-2-oxoethyl)urea: C110SR**

**Step 1: (S)-2-(5-bromo-2-methoxyphenyl)-2-((tert-butoxycarbonyl)amino)acetic acid**

The compound is obtained by the procedure described in Example 32, Step 1, starting from 265 mg (0.89 mmol) of (S)-2-amino-2-(5-bromo-2-methoxyphenyl)acetic acid HCl instead of (R)-2-amino-2-(5-bromo-2-methoxyphenyl)acetic acid HCl. 363 mg of the title compound are obtained as a colorless gel.

Yield: quant.

MH+ : 360.4-362.4 (M+1)

**Step 2: tert-butyl (S)-(2-(2-(benzo[b]thiophen-7-yl)pyrrolidin-1-yl)-1-(5-bromo-2-methoxyphenyl)-2-oxoethyl)carbamate**

The compound is obtained by the procedure described in Example 1, Step 4, starting from 322 mg (0.89 mmol) of (S)-2-(5-bromo-2-methoxyphenyl)-2-((tert-butoxycarbonyl)amino)acetic acid (described in the previous step) and 2-(benzo[b]thiophen-7-yl)pyrrolidine instead of 2-(2-(methylthio)phenyl)pyrrolidine. 501 mg of the title compound are obtained as a yellow gel.

Yield: quant.

MH+ : 545.5-547.5 (M+1)

**Step 3: (S)-2-amino-1-(2-(benzo[b]thiophen-7-yl)pyrrolidin-1-yl)-2-(5-bromo-2-methoxyphenyl)ethan-1-one**

The compound is obtained by the procedure described in Example 1, Step 5, starting from 501 mg (0.92 mmol) of tert-butyl (S)-(2-(2-(benzo[b]thiophen-7-yl)pyrrolidin-1-yl)-1-(5-bromo-2-methoxyphenyl)-2-oxoethyl)carbamate (described in the previous step). 270 mg of the title compound are obtained as a yellow gel.

Yield: 66%

MH+ : 445.5-447.4 (M+1)

**Step 4: tert-butyl (S)-(4-((3-(2-(2-(benzo[b]thiophen-7-yl)pyrrolidin-1-yl)-1-(5-bromo-2-methoxyphenyl)-2-oxoethyl)ureido)methyl)phenyl)carbamate**

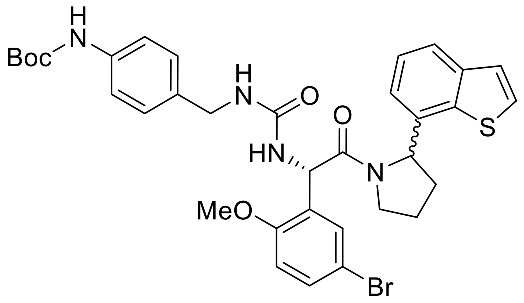


The compound is obtained by the procedure described in Example 1, Step 6, starting from 140 mg (0.31 mmol) of (S)-2-amino-1-(2-(benzo[b]thiophen-7-yl)pyrrolidin-1-yl)-2-(5-bromo-2-methoxyphenyl)ethan-1-one (described in the previous step). 186 mg of the title compound are obtained as a white solid.

Yield: 85%

MH+ : 693.5-695.5 (M+1)

**Step 5: (S)-1-(4-aminobenzyl)-3-(2-(2-(benzo[b]thiophen-7-yl)pyrrolidin-1-yl)-1-(5-bromo-2-methoxyphenyl)-2-oxoethyl)urea**

The compound is obtained by the procedure described in Example 1, Step 7, starting from 186 mg (0.29 mmol) of tert-butyl (S)-(4-((3-(2-(2-(benzo[b]thiophen-7-yl)pyrrolidin-1-yl)-1-(5-bromo-2-methoxyphenyl)-2-oxoethyl)ureido)methyl)phenyl)carbamate (described in the previous step). 109 mg of the title compound are obtained as a white solid.

Yield: 69%

MH+ : 593.5-595.5 (M+1)

**Step 6: 1-(4-aminobenzyl)-3-((S)-2-((R)-2-(benzo[b]thiophen-7-yl)pyrrolidin-1-yl)-1-(5-bromo-2-methoxyphenyl)-2-oxoethyl)urea**

(S)-1-(4-aminobenzyl)-3-(2-(2-(benzo[b]thiophen-7-yl)pyrrolidin-1-yl)-1-(5-bromo-2-methoxyphenyl)-2-oxoethyl)urea (described in previous step) is separated by flash chromatography using a Chiralflash IG column and an EtOH/DCM mixture as the mobile phase. First fraction to be eluted is 1-(4-aminobenzyl)-3-((S)-2-((R)-2-(benzo[b]thiophen-7-yl)pyrrolidin-1-yl)-1-(5-bromo-2-methoxyphenyl)-2-oxoethyl)urea with ee > 99%. Product is purified by flash chromatography using a C18 column and an $H_2O$/MeOH mixture as eluent. 20 mg of the title compound is obtained as a white solid.

MH+ : 593.5-595.5 (M+1)

**Example 33: Synthesis of 1-(4-aminobenzyl)-3-((S)-2-((S)-2-(benzo[b]thiophen-7-yl)pyrrolidin-1-yl)-1-(5-bromo-2-methoxyphenyl)-2-oxoethyl)urea: C110SS**

**Step 1: 1-(4-aminobenzyl)-3-((S)-2-((S)-2-(benzo[b]thiophen-7-yl)pyrrolidin-1-yl)-1-(5-bromo-2-methoxyphenyl)-2-oxoethyl)urea**

(S)-1-(4-aminobenzyl)-3-(2-(2-(benzo[b]thiophen-7-yl)pyrrolidin-1-yl)-1-(5-bromo-2-methoxyphenyl)-2-oxoethyl)urea (described in Example 34, Step 5) is separated by flash chromatography using a Chiralflash IG column and an EtOH/DCM mixture as the mobile phase. Second fraction to be eluted is 1-(4-aminobenzyl)-3-((S)-2-((S)-2-(benzo[b]thiophen-7-yl)pyrrolidin-1-yl)-1-(5-bromo-2-methoxyphenyl)-2-oxoethyl)urea with ee > 99%. Product is purified by flash chromatography using a C18 column and an $H_2O$/MeOH mixture as eluent. 32 mg of the title compound is obtained as a white solid.

MH+ : 593.5-595.5 (M+1)

**Example 34: Synthesis of 1-(2-(2-(benzo[b]thiophen-7-yl)pyrrolidin-1-yl)-1-(5-bromo-2-methoxyphenyl)-2-oxoethyl)-3-((1,2,3,4-tetrahydroquinolin-6-yl)methyl)urea: C105**

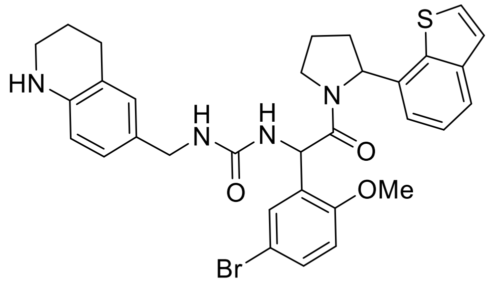


**Step 1: tert-butyl 6-((3-(2-(2-(benzo[b]thiophen-7-yl)pyrrolidin-1-yl)-1-(5-bromo-2-methoxyphenyl)-2-oxoethyl)ureido)methyl)-3,4-dihydroquinoline-1(2H)-carboxylate**

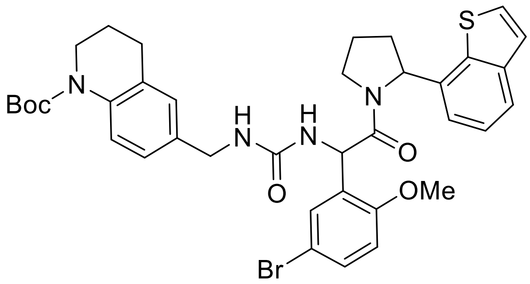


The compound is obtained by the procedure described in Example 1, Step 6, starting from 77 mg (0.17 mmol) of 2-amino-1-(2-(benzo[b]thiophen-7-yl)pyrrolidin-1-yl)-2-(5-bromo-2-methoxyphenyl)ethan-1-one (described in Example 31, Step 2) and tert-butyl 6-(aminomethyl)-3,4-dihydroquinoline-1(2H)-carboxylate instead of tert-butyl (4-(aminomethyl)phenyl)carbamate. 89 mg of the title compound are obtained as a colorless oil.

Yield: 70%

MH+ : 733.5-735.6 (M+1)

**Step 2: 1-(2-(2-(benzo[b]thiophen-7-yl)pyrrolidin-1-yl)-1-(5-bromo-2-methoxyphenyl)-2-oxoethyl)-3-((1,2,3,4-tetrahydroquinolin-6-yl)methyl)urea**

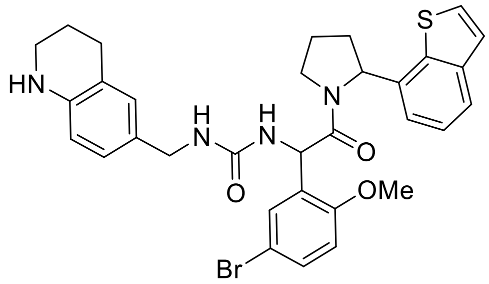


The compound is obtained by the procedure described in Example 1, Step 7, starting from 89 mg (0.12 mmol) of tert-butyl 6-((3-(2-(2-(benzo[b]thiophen-7-yl)pyrrolidin-1-yl)-1-(5-bromo-2-methoxyphenyl)-2-oxoethyl)ureido)methyl)-3,4-dihydroquinoline-1(2H)-carboxylate (described in the previous step). 59 mg of the title compound are obtained as a slightly yellow solid.

Yield: 77%

MH+ : 633.5-635.6 (M+1)

**Example 35: Synthesis of 1-((R)-2-((S)-2-(benzo[b]thiophen-7-yl)pyrrolidin-1-yl)-1-(5-bromo-2-methoxyphenyl)-2-oxoethyl)-3-((1,2,3,4-tetrahydroquinolin-6-yl)methyl)urea: C105RS**

**Step 1: tert-butyl (R)-6-((3-(2-(2-(benzo[b]thiophen-7-yl)pyrrolidin-1-yl)-1-(5-bromo-2-methoxyphenyl)-2-oxoethyl)ureido)methyl)-3,4-dihydroquinoline-1(2H)-carboxylate**

The compound is obtained by the procedure described in Example 1, Step 6, starting from 130 mg (0.29 mmol) of (R)-2-amino-1-(2-(benzo[b]thiophen-7-yl)pyrrolidin-1-yl)-2-(5-bromo-2-methoxyphenyl)ethan-1-one (described in Example 32, Step 3). 151 mg of the title compound are obtained as a white solid.

Yield: 71%

MH+ : 733.5-735.6 (M+1)

**Step 2: (R)-1-(2-(2-(benzo[b]thiophen-7-yl)pyrrolidin-1-yl)-1-(5-bromo-2-methoxyphenyl)-2-oxoethyl)-3-((1,2,3,4-tetrahydroquinolin-6-yl)methyl)urea**

The compound is obtained by the procedure described in Example 1, Step 6, starting from 151 mg (0.21 mmol) of tert-butyl (R)-6-((3-(2-(2-(benzo[b]thiophen-7-yl)pyrrolidin-1-yl)-1-(5-bromo-2-methoxyphenyl)-2-oxoethyl)ureido)methyl)-3,4-dihydroquinoline-1(2H)-carboxylate (described in the previous step). 107 mg of the title compound are obtained as a white solid.

Yield: 82%

MH+ : 633.5-635.6 (M+1)

**Step 3: 1-((R)-2-((S)-2-(benzo[b]thiophen-7-yl)pyrrolidin-1-yl)-1-(5-bromo-2-methoxyphenyl)-2-oxoethyl)-3-((1,2,3,4-tetrahydroquinolin-6-yl)methyl)urea**

(R)-1-(2-(2-(benzo[b]thiophen-7-yl)pyrrolidin-1-yl)-1-(5-bromo-2-methoxyphenyl)-2-oxoethyl)-3-((1,2,3,4-tetrahydroquinolin-6-yl)methyl)urea (described in previous step) is separated by flash chromatography using a Chiralflash IG column and an EtOH/DCM mixture as the mobile phase. First fraction to be eluted is 1-((R)-2-((S)-2-(benzo[b]thiophen-7-yl)pyrrolidin-1-yl)-1-(5-bromo-2-methoxyphenyl)-2-oxoethyl)-3-((1,2,3,4-tetrahydroquinolin-6-yl)methyl)urea with ee > 99%. Product is purified by flash chromatography using a C18 column and an $H_2O$/MeOH mixture as eluent. 25 mg of the title compound is obtained as a white solid.

MH+ : 633.5-635.6 (M+1)

**<u>Example 36: Synthesis of 1-((R)-2-((R)-2-(benzo[b]thiophen-7-yl)pyrrolidin-1-yl)-1-(5-bromo-2-methoxyphenyl)-2-oxoethyl)-3-((1,2,3,4-tetrahydroquinolin-6-yl)methyl)urea: C105RR</u>**

**Step 1: 1-((R)-2-((R)-2-(benzo[b]thiophen-7-yl)pyrrolidin-1-yl)-1-(5-bromo-2-methoxyphenyl)-2-oxoethyl)-3-((1,2,3,4-tetrahydroquinolin-6-yl)methyl)urea**

(R)-1-(2-(2-(benzo[b]thiophen-7-yl)pyrrolidin-1-yl)-1-(5-bromo-2-methoxyphenyl)-2-oxoethyl)-3-((1,2,3,4-tetrahydroquinolin-6-yl)methyl)urea (described in Example 37, Step 2) is separated by flash chromatography using a Chiralflash IG column and an EtOH/DCM mixture as the mobile phase. Second fraction to be eluted is 1-((R)-2-((R)-2-(benzo[b]thiophen-7-yl)pyrrolidin-1-yl)-1-(5-bromo-2-methoxyphenyl)-2-oxoethyl)-3-((1,2,3,4-tetrahydroquinolin-6-yl)methyl)urea with ee > 96%. Product is purified by flash chromatography using a C18 column and an $H_2O$/MeOH mixture as eluent. 33 mg of the title compound is obtained as a white solid.

MH+ : 633.5-635.6 (M+1)

**<u>Example 37: Synthesis of 1-((S)-2-((R)-2-(benzo[b]thiophen-7-yl)pyrrolidin-1-yl)-1-(5-bromo-2-methoxyphenyl)-2-oxoethyl)-3-((1,2,3,4-tetrahydroquinolin-6-yl)methyl)urea: C105SR</u>**

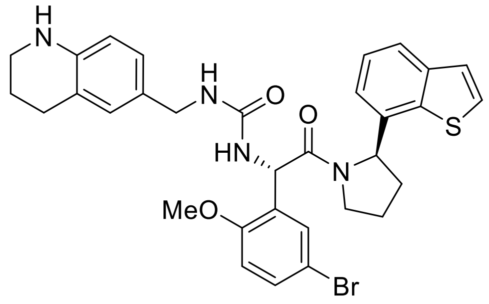


**Step 1: tert-butyl (S)-6-((3-(2-(2-(benzo[b]thiophen-7-yl)pyrrolidin-1-yl)-1-(5-bromo-2-methoxyphenyl)-2-oxoethyl)ureido)methyl)-3,4-dihydroquinoline-1(2H)-carboxylate**

The compound is obtained by the procedure described in Example 1, Step 6, starting from 124 mg (0.28 mmol) of (S)-2-amino-1-(2-(benzo[b]thiophen-7-yl)pyrrolidin-1-yl)-2-(5-bromo-2-methoxyphenyl)ethan-1-one (described in Example 34, Step 3). 162 mg of the title compound are obtained as a colorless gel.

Yield: 79%

MH+ : 733.5-735.6 (M+1)

**Step 2: (S)-1-(2-(2-(benzo[b]thiophen-7-yl)pyrrolidin-1-yl)-1-(5-bromo-2-methoxyphenyl)-2-oxoethyl)-3-((1,2,3,4-tetrahydroquinolin-6-yl)methyl)urea**

The compound is obtained by the procedure described in Example 1, Step 6, starting from 162 mg (0.22 mmol) of tert-butyl (S)-6-((3-(2-(2-(benzo[b]thiophen-7-yl)pyrrolidin-1-yl)-1-(5-bromo-2-methoxyphenyl)-2-oxoethyl)ureido)methyl)-3,4-dihydroquinoline-1(2H)-carboxylate (described in the previous step). 106 mg of the title compound are obtained as a white solid.

Yield: 76%

MH+ : 633.5-635.6 (M+1)

**Step 3: 1-((S)-2-((R)-2-(benzo[b]thiophen-7-yl)pyrrolidin-1-yl)-1-(5-bromo-2-methoxyphenyl)-2-oxoethyl)-3-((1,2,3,4-tetrahydroquinolin-6-yl)methyl)urea**

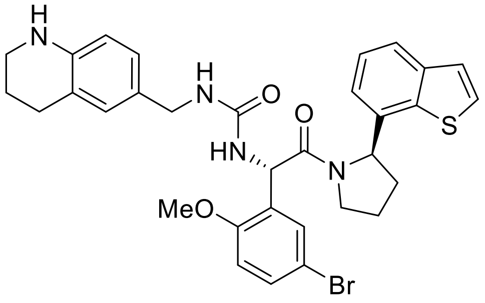


(S)-1-(2-(2-(benzo[b]thiophen-7-yl)pyrrolidin-1-yl)-1-(5-bromo-2-methoxyphenyl)-2-oxoethyl)-3-((1,2,3,4-tetrahydroquinolin-6-yl)methyl)urea (described in previous step) is separated

by flash chromatography using a Chiralflash IG column and an EtOH/DCM mixture as the mobile phase. First fraction to be eluted is 1-((S)-2-((R)-2-(benzo[b]thiophen-7-yl)pyrrolidin-1-yl)-1-(5-bromo-2-methoxyphenyl)-2-oxoethyl)-3-((1,2,3,4-tetrahydroquinolin-6-yl)methyl)urea with ee > 98%. Product is purified by flash chromatography using a C18 column and an $H_2O$/MeOH mixture as eluent. 15 mg of the title compound is obtained as a pale yellow solid.

MH+ : 633.5-635.6 (M+1)

**Example 38: Synthesis of 1-((S)-2-((S)-2-(benzo[b]thiophen-7-yl)pyrrolidin-1-yl)-1-(5-bromo-2-methoxyphenyl)-2-oxoethyl)-3-((1,2,3,4-tetrahydroquinolin-6-yl)methyl)urea: C105SS**

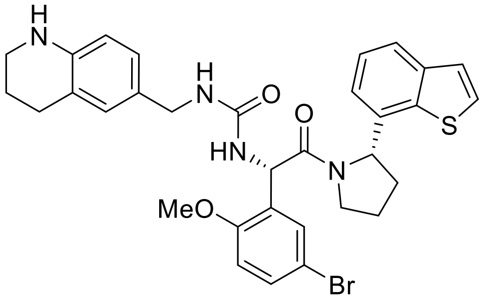


**Step 1: 1-((S)-2-((S)-2-(benzo[b]thiophen-7-yl)pyrrolidin-1-yl)-1-(5-bromo-2-methoxyphenyl)-2-oxoethyl)-3-((1,2,3,4-tetrahydroquinolin-6-yl)methyl)urea**

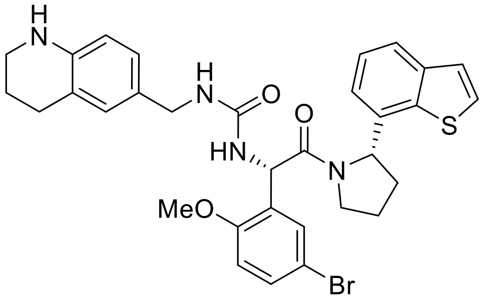


(S)-1-(2-(2-(benzo[b]thiophen-7-yl)pyrrolidin-1-yl)-1-(5-bromo-2-methoxyphenyl)-2-oxoethyl)-3-((1,2,3,4-tetrahydroquinolin-6-yl)methyl)urea (described in Example 39, Step 2) is separated by flash chromatography using a Chiralflash IG column and an EtOH/DCM mixture as the mobile phase. Second fraction to be eluted is 1-((S)-2-((S)-2-(benzo[b]thiophen-7-yl)pyrrolidin-1-yl)-1-(5-bromo-2-methoxyphenyl)-2-oxoethyl)-3-((1,2,3,4-tetrahydroquinolin-6-yl)methyl)urea with ee > 98%. Product is purified by flash chromatography using a C18 column and an $H_2O$/MeOH mixture as eluent. 29 mg of the title compound is obtained as a white solid.

MH+ : 633.5-635.6 (M+1)

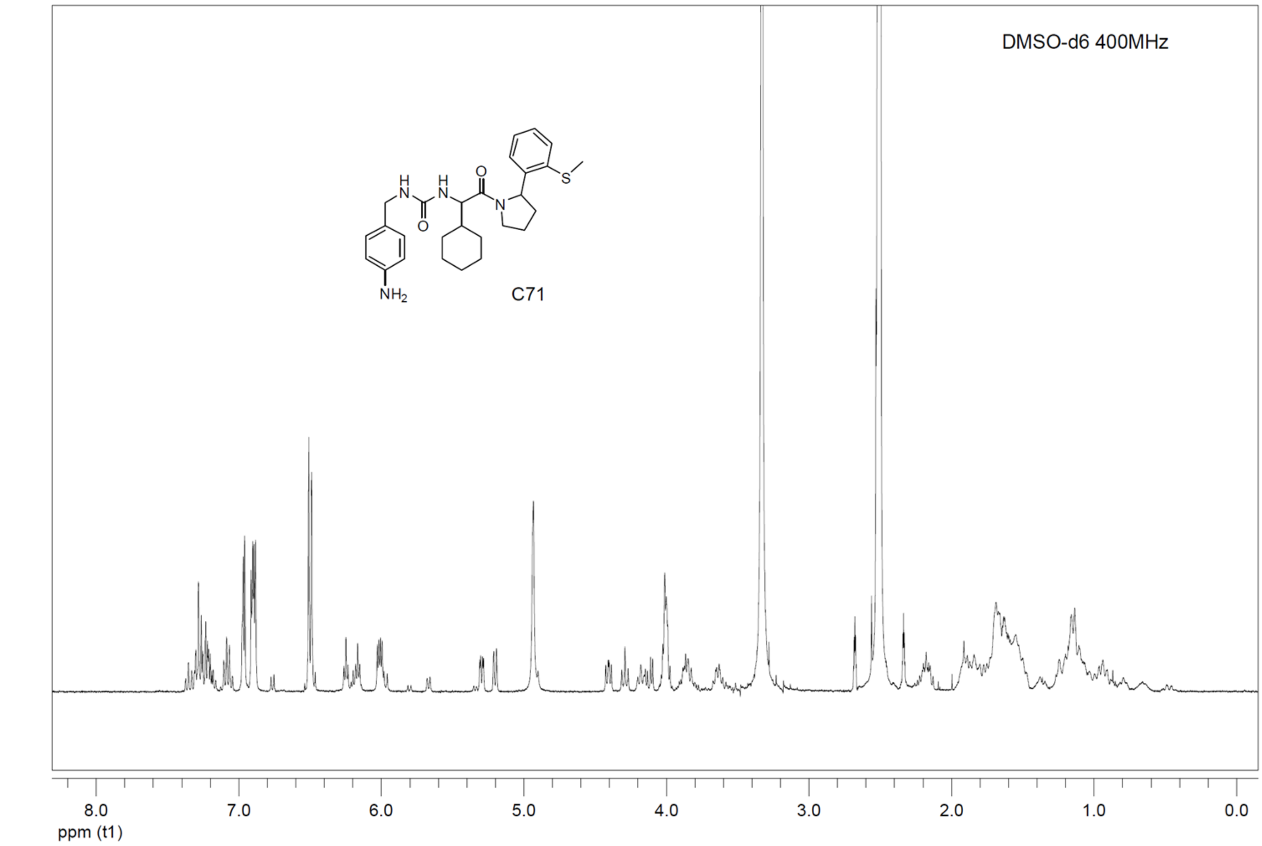


$^{1}$H NMR (400 MHz, DMSO-d6) spectrum of compound C71.

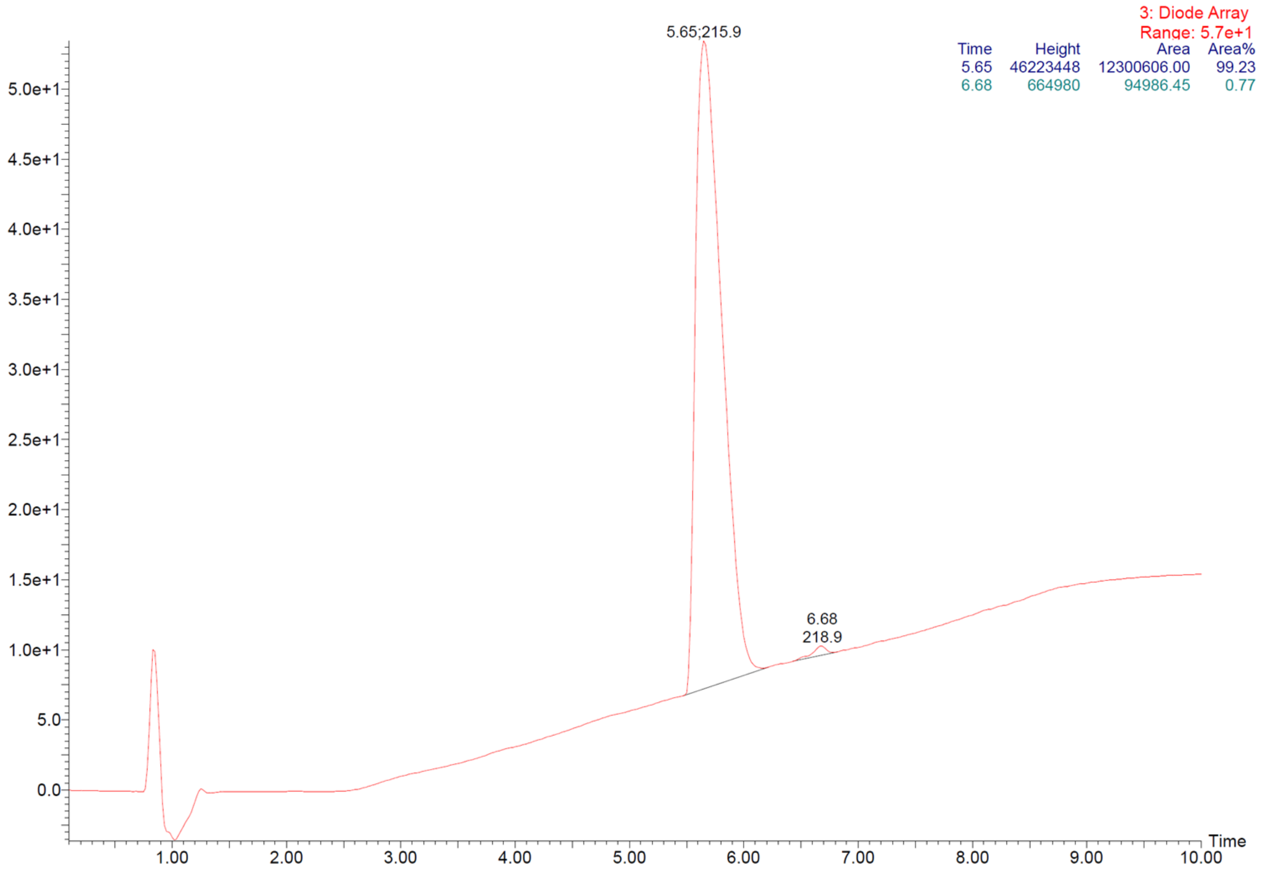


HPLC Chromatogram of compound C71.

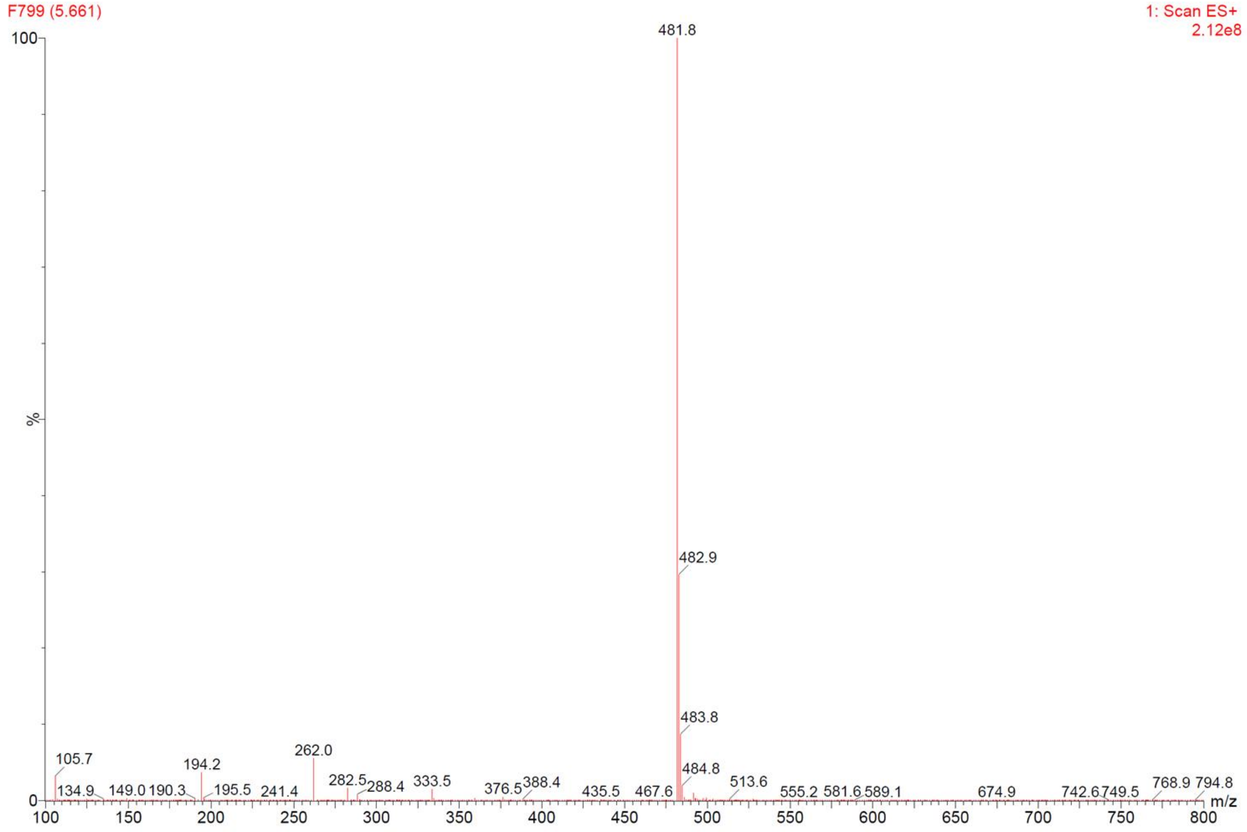


Mass spectrum of compound C71

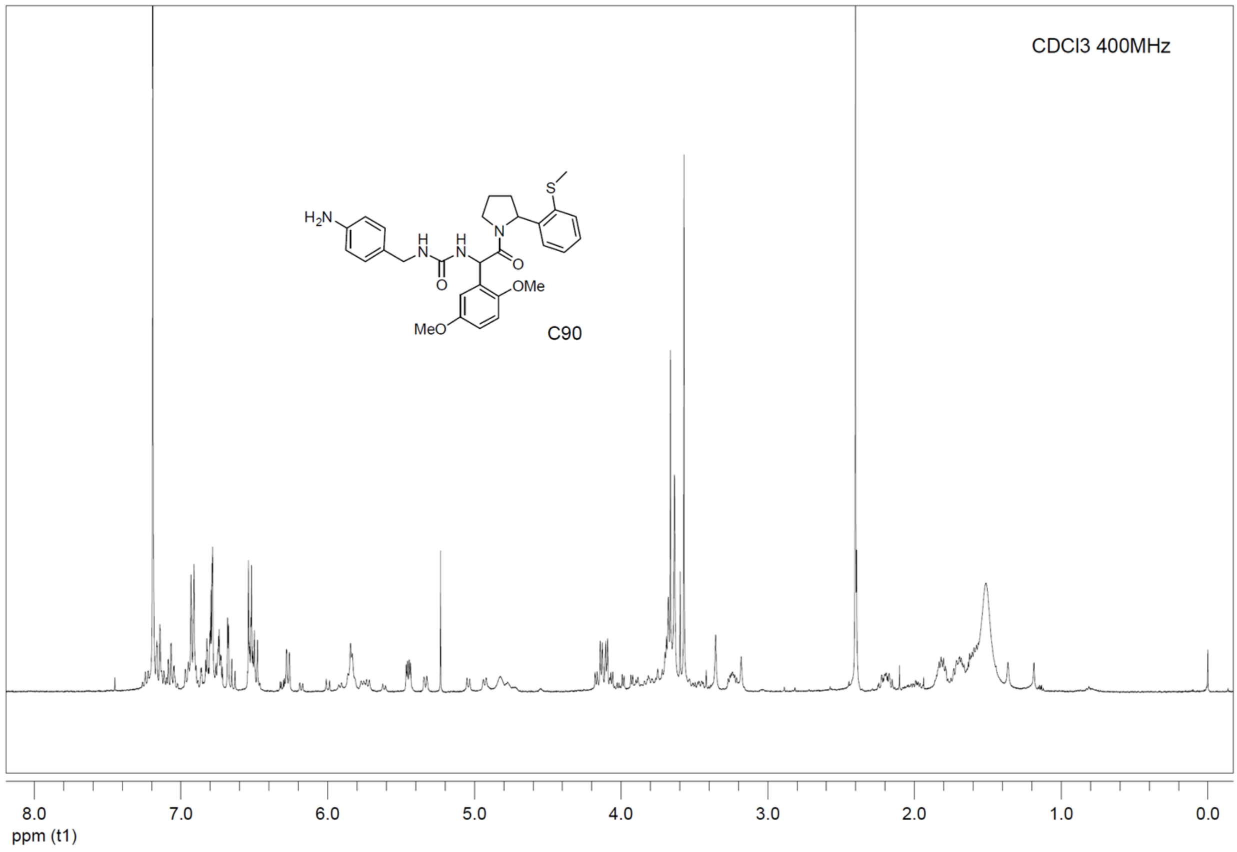


$^1H$ NMR (400 MHz, $CDCl_3$) spectrum of compound C90.

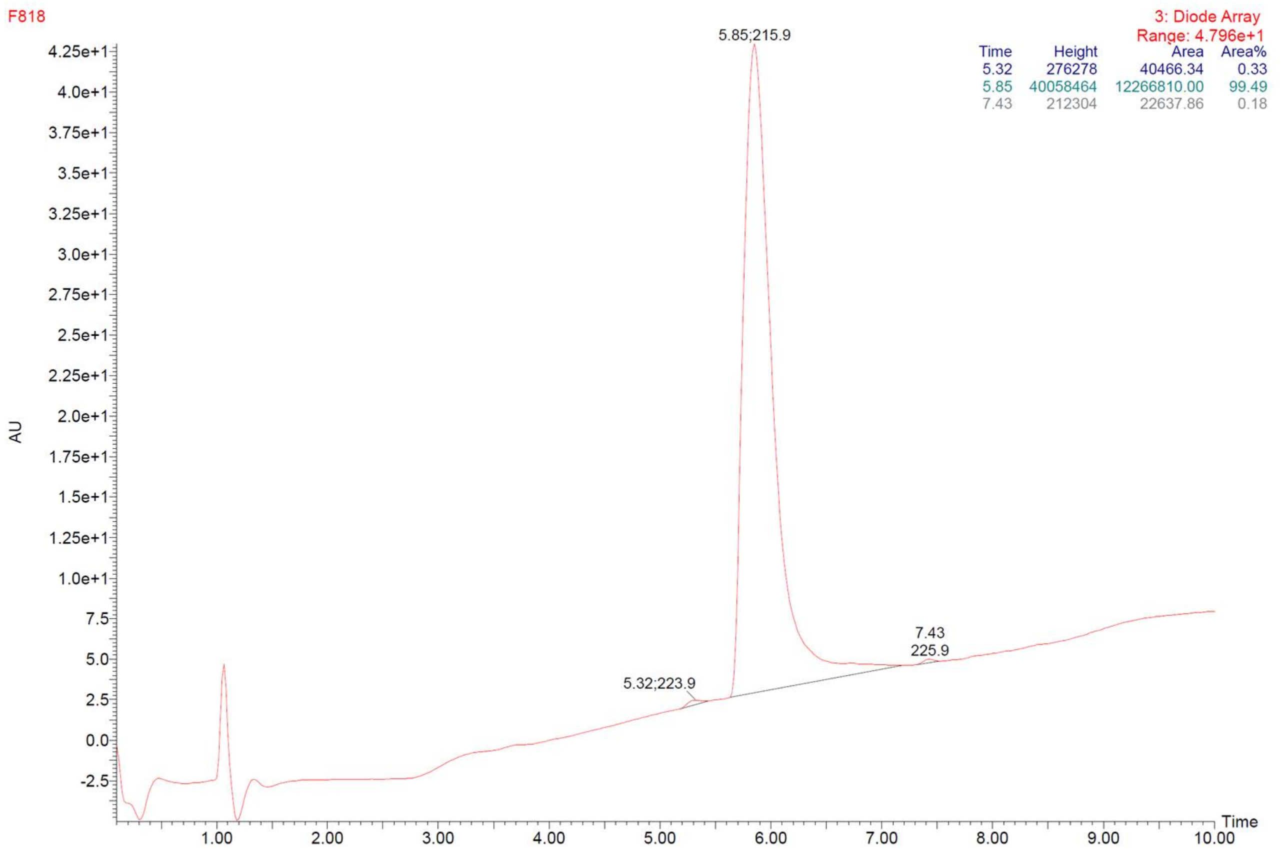


HPLC Chromatogram of compound C90.

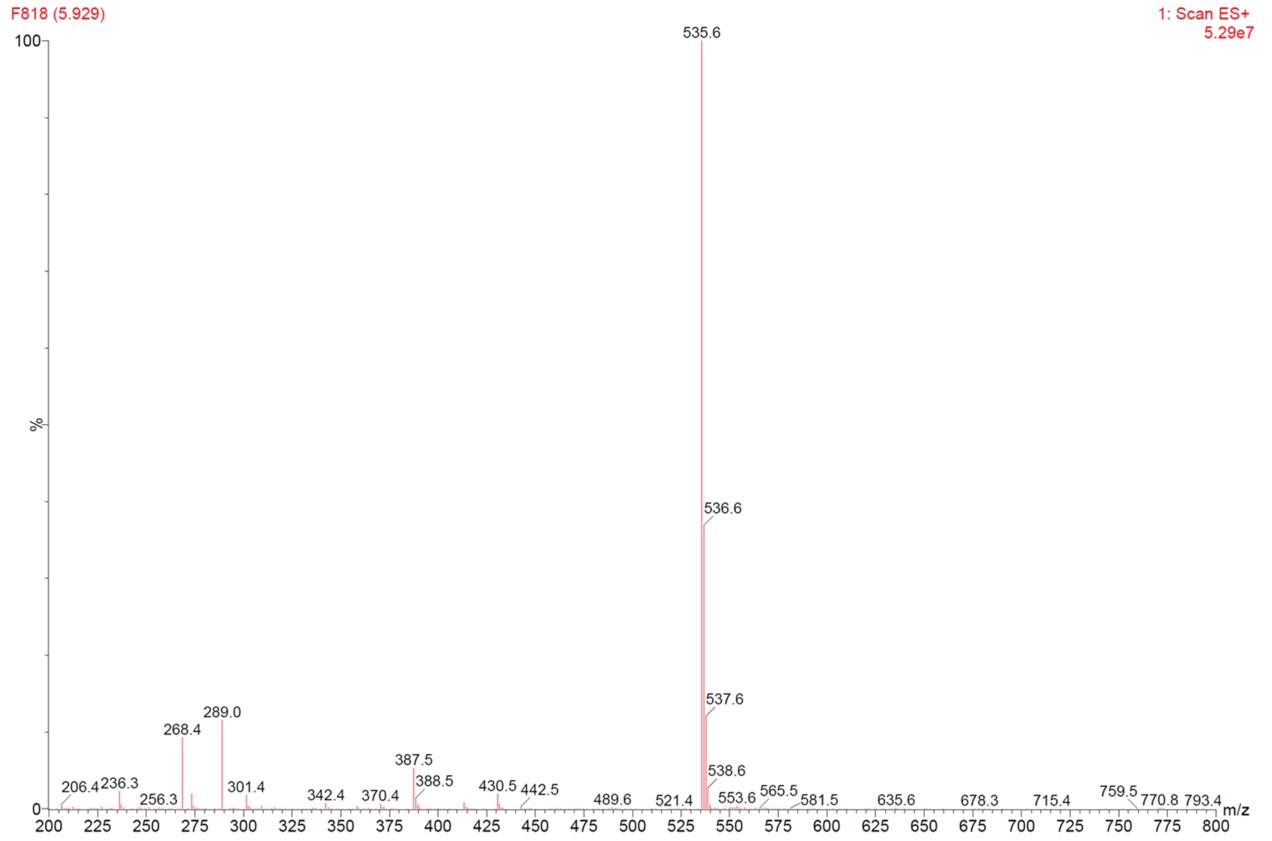


Mass spectrum of compound C90.

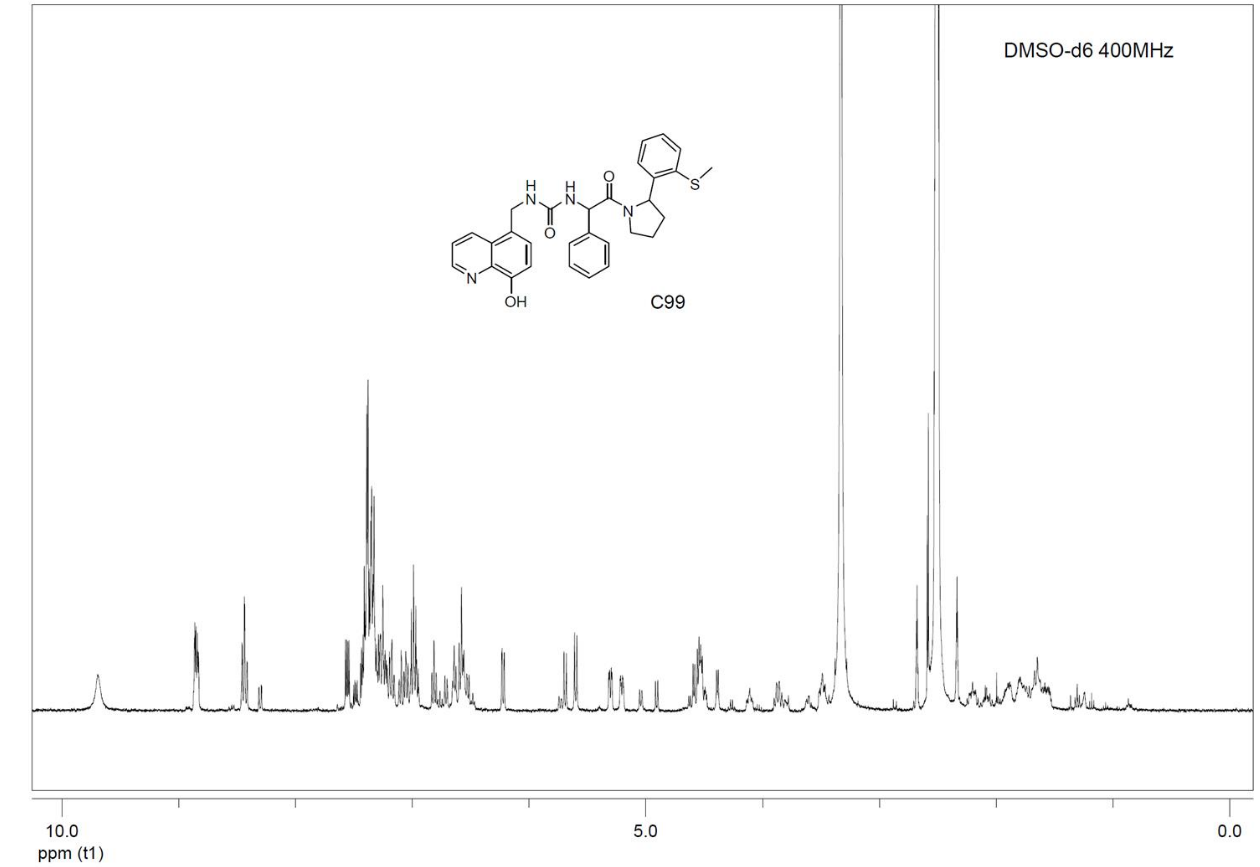


$^{1}$H NMR (400 MHz, DMSO-d6) spectrum of compound C99.

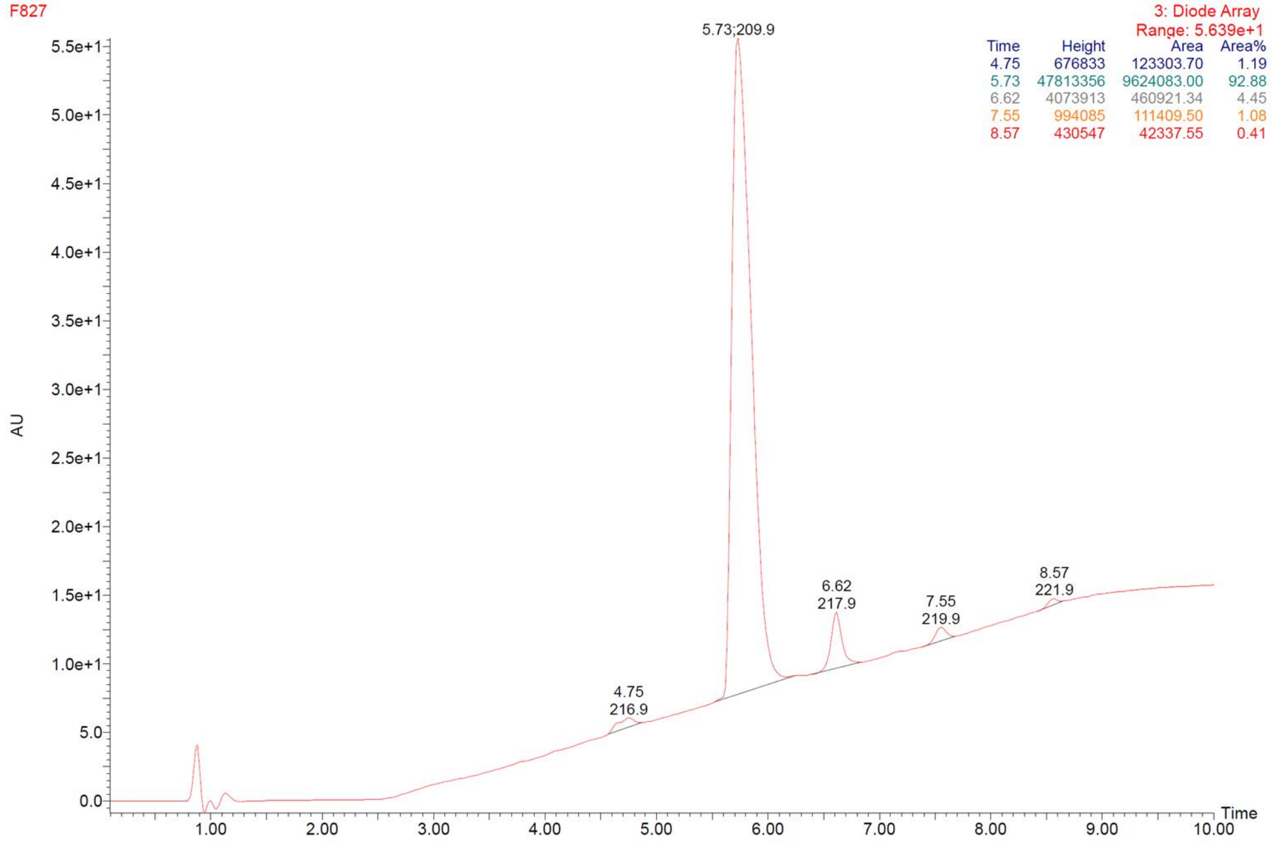


HPLC Chromatogram of compound C99.

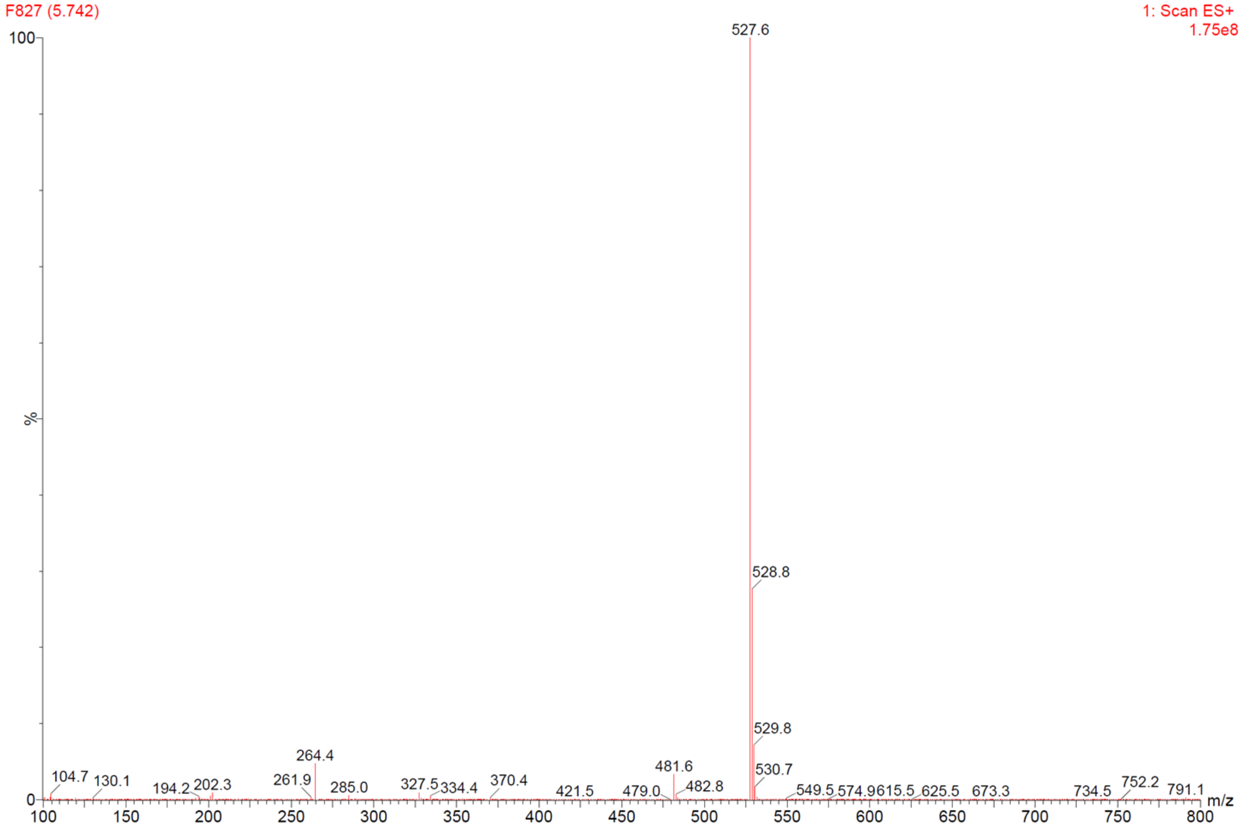


Mass spectrum of compound C99.

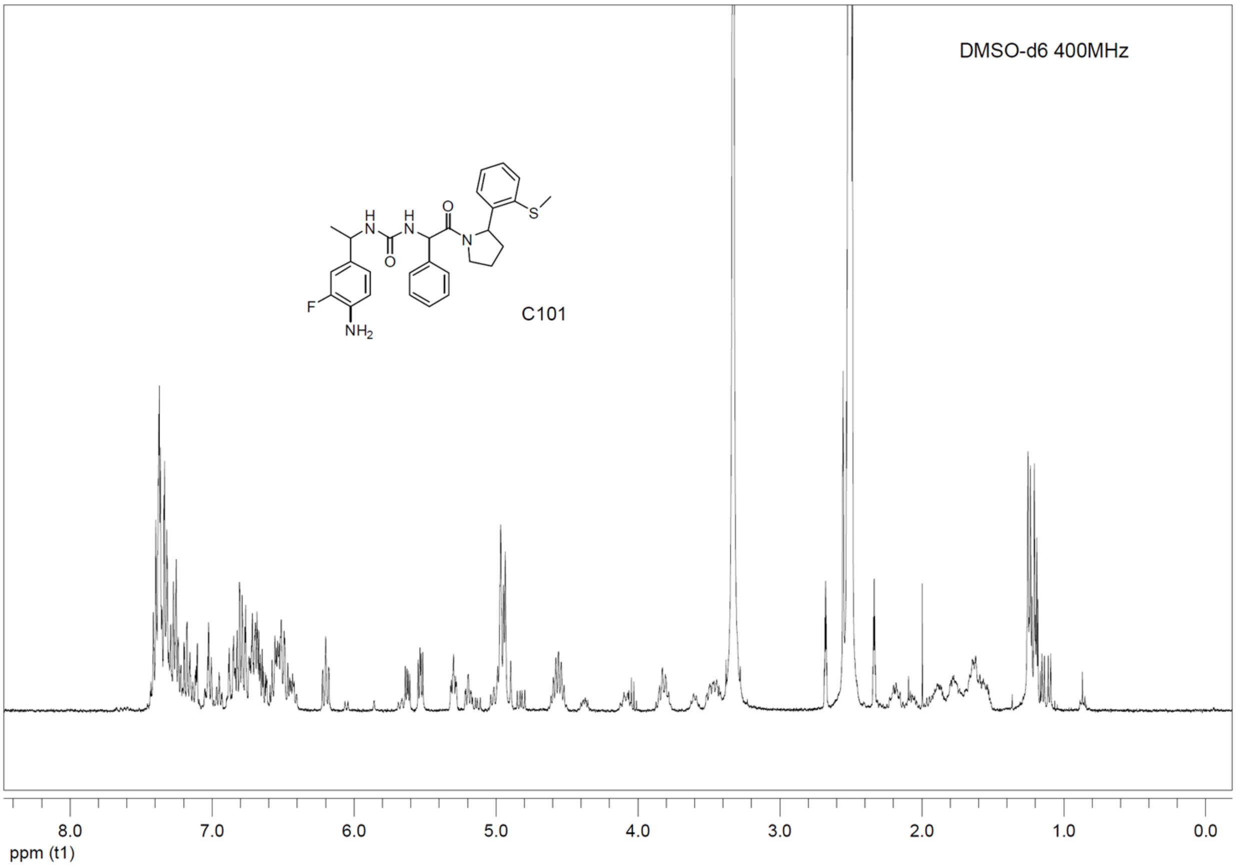


$^{1}$H NMR (400 MHz, DMSO-d6) spectrum of compound C101.

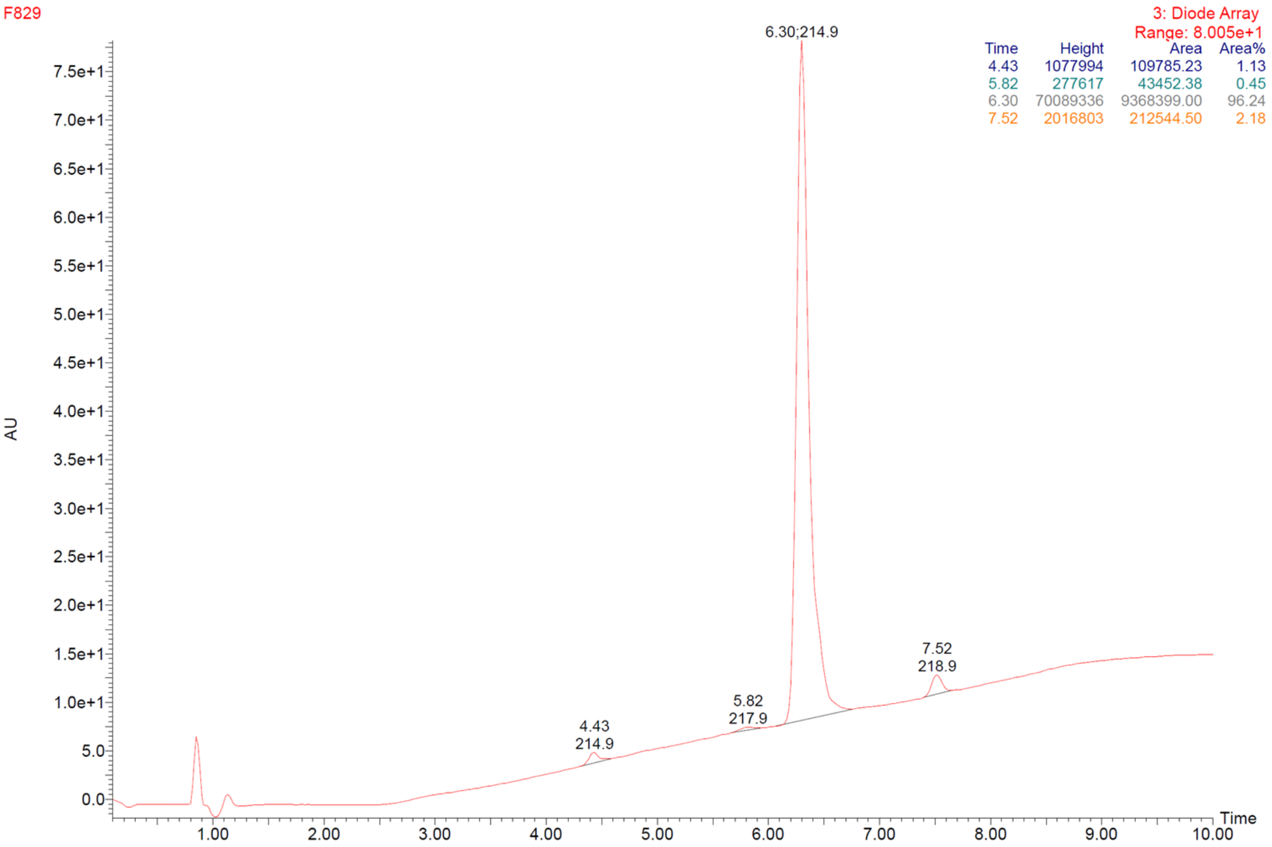


HPLC Chromatogram of compound C101.

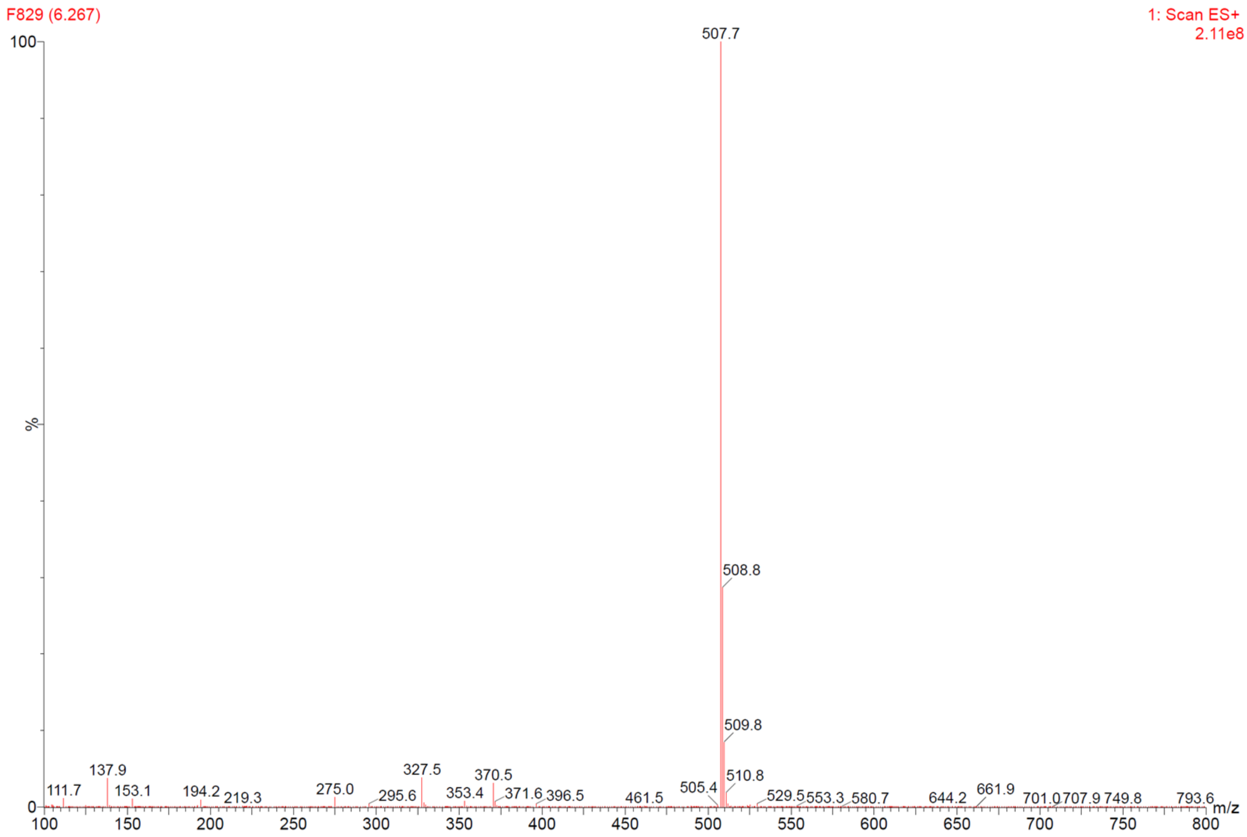


Mass spectrum of compound C101.

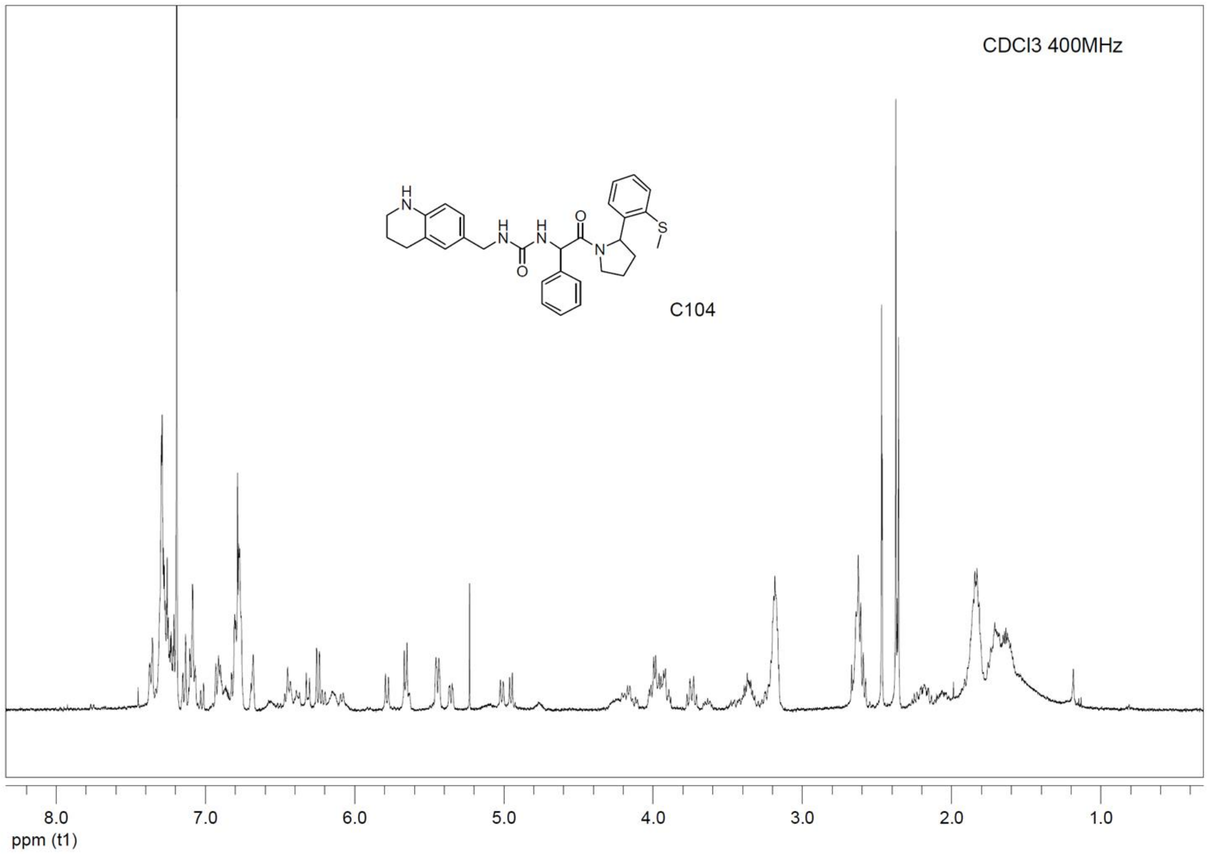


$^{1}$H NMR (400 MHz, $CDCl_3$) spectrum of compound C104.

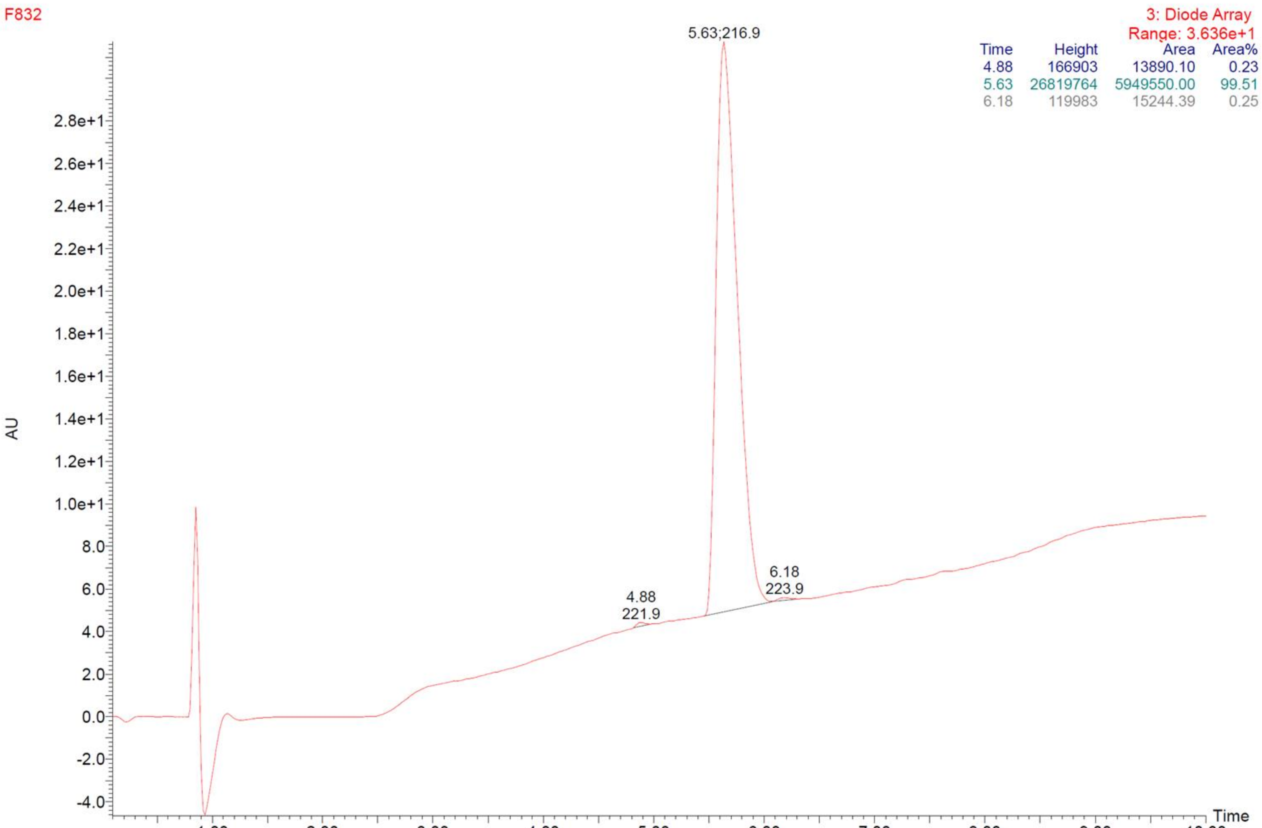


HPLC Chromatogram of compound C104.

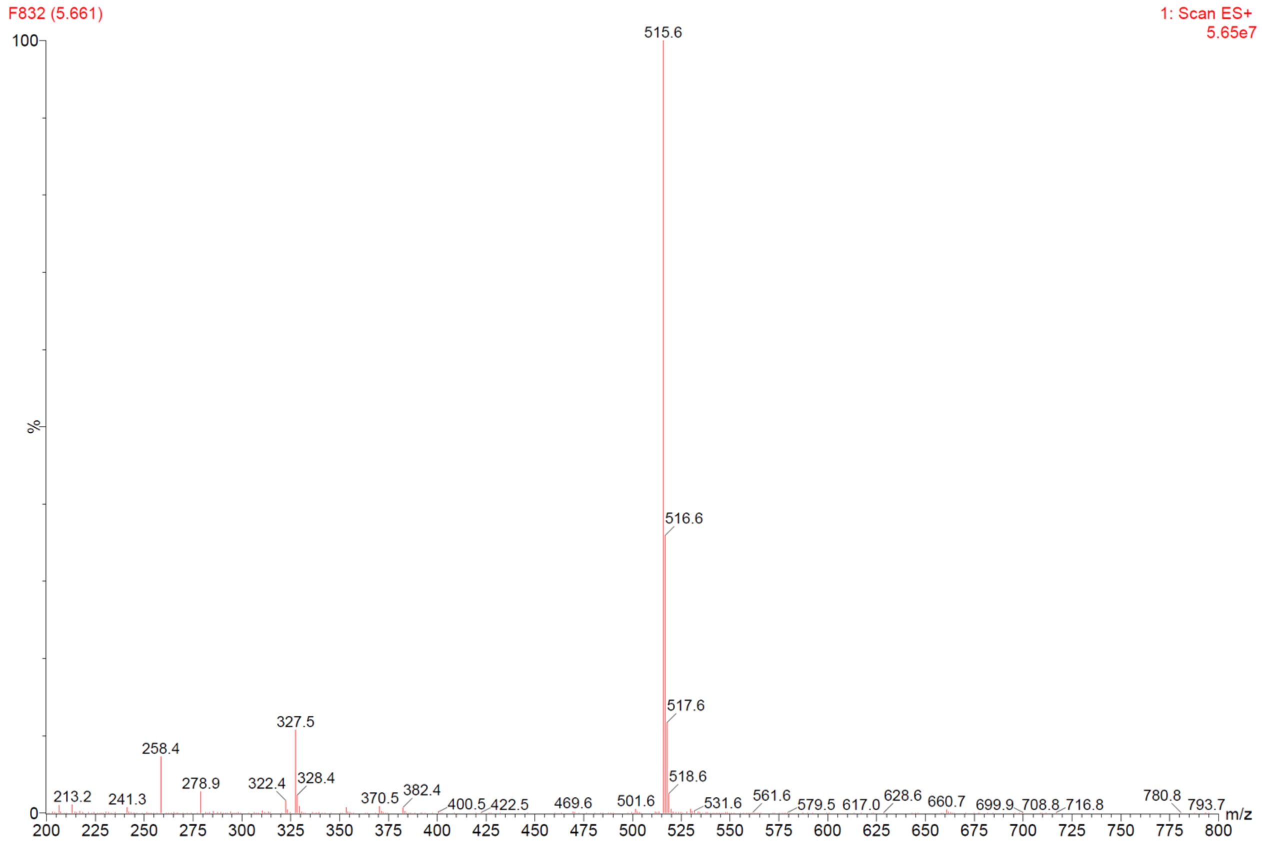


Mass spectrum of compound C104.

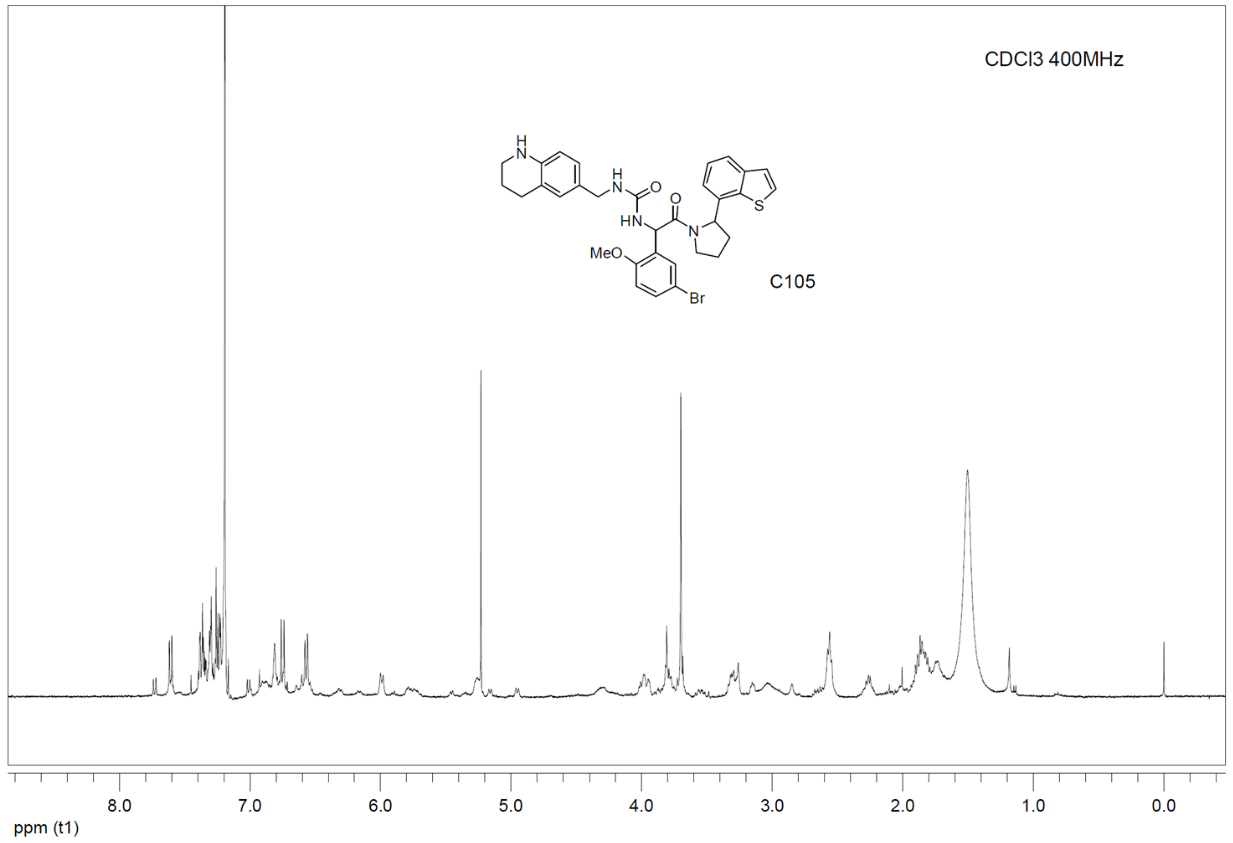


$^{1}H$ NMR (400 MHz, $CDCl_3$) spectrum of compound C105.

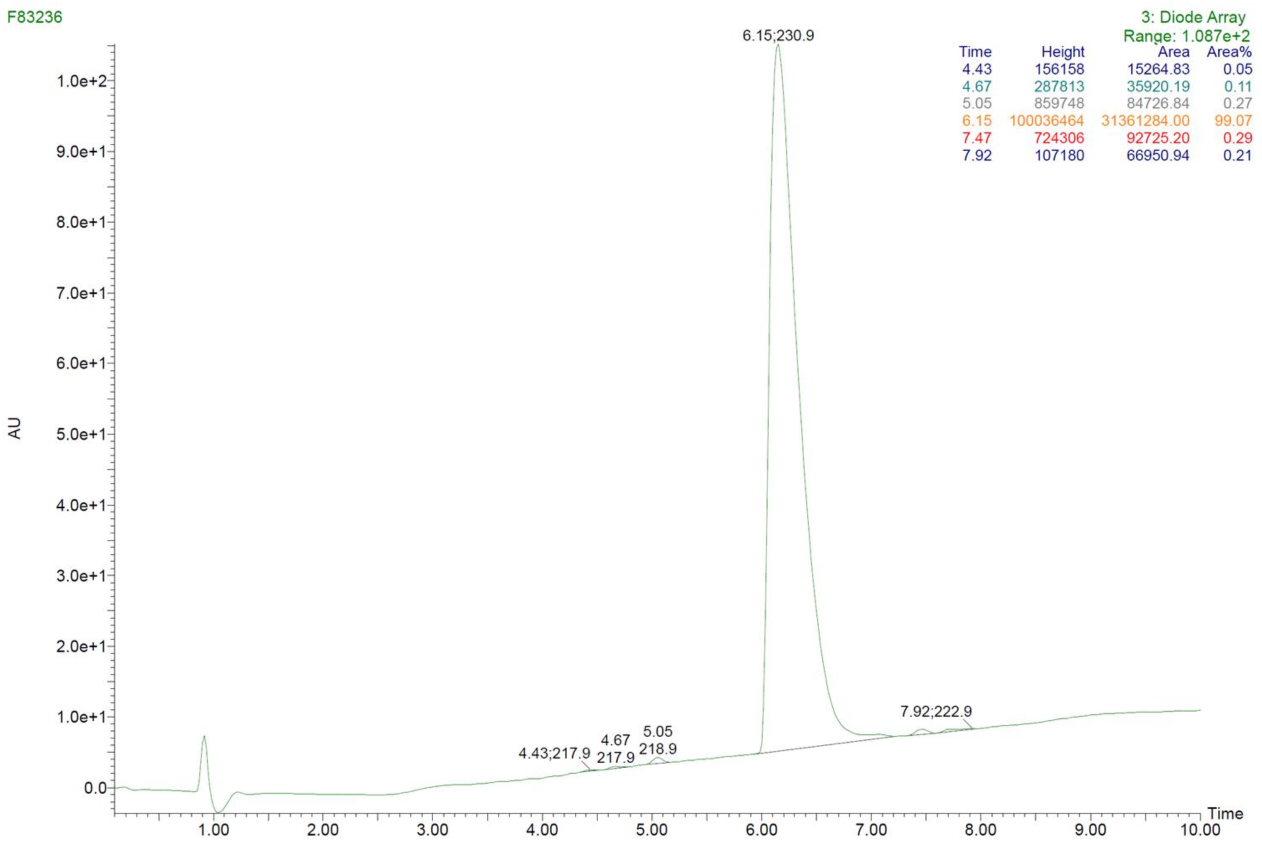


HPLC Chromatogram of compound C105.

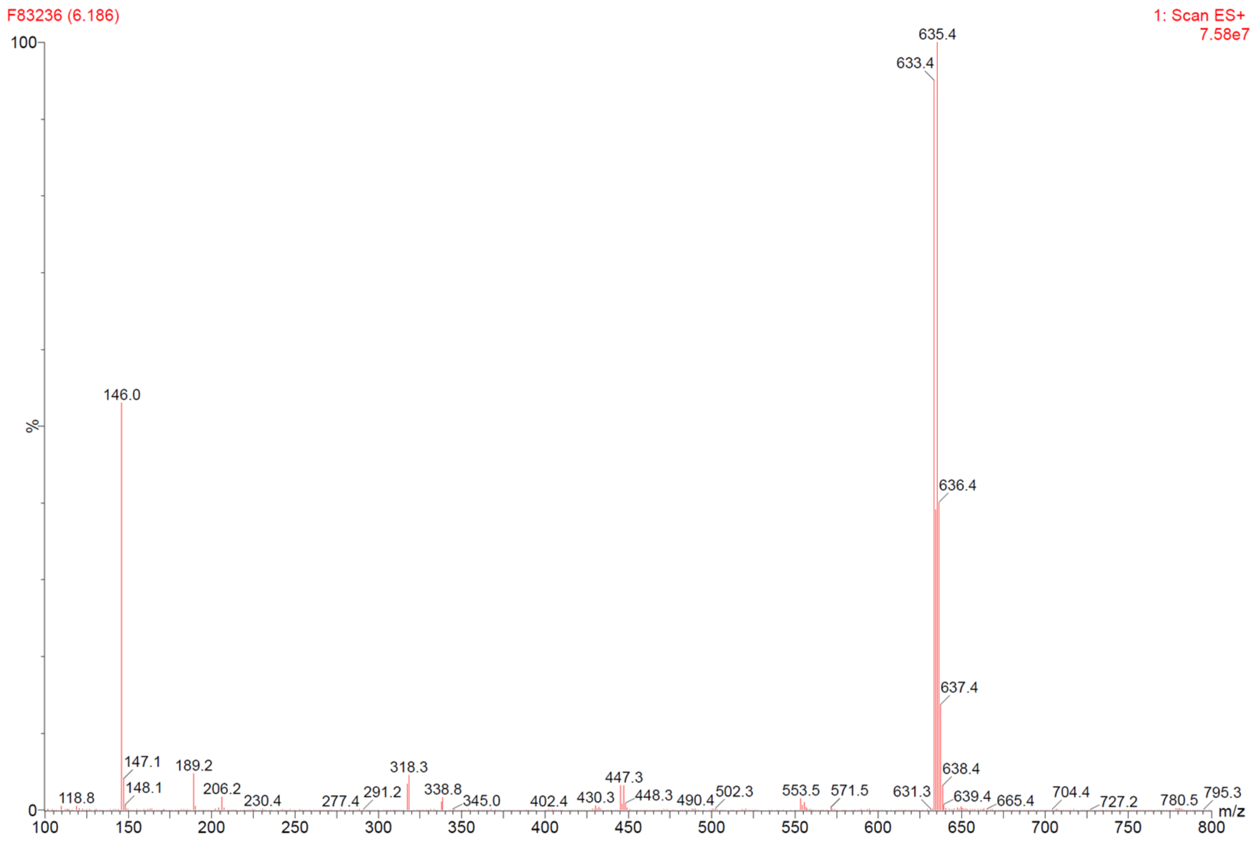


Mass spectrum of compound C105.

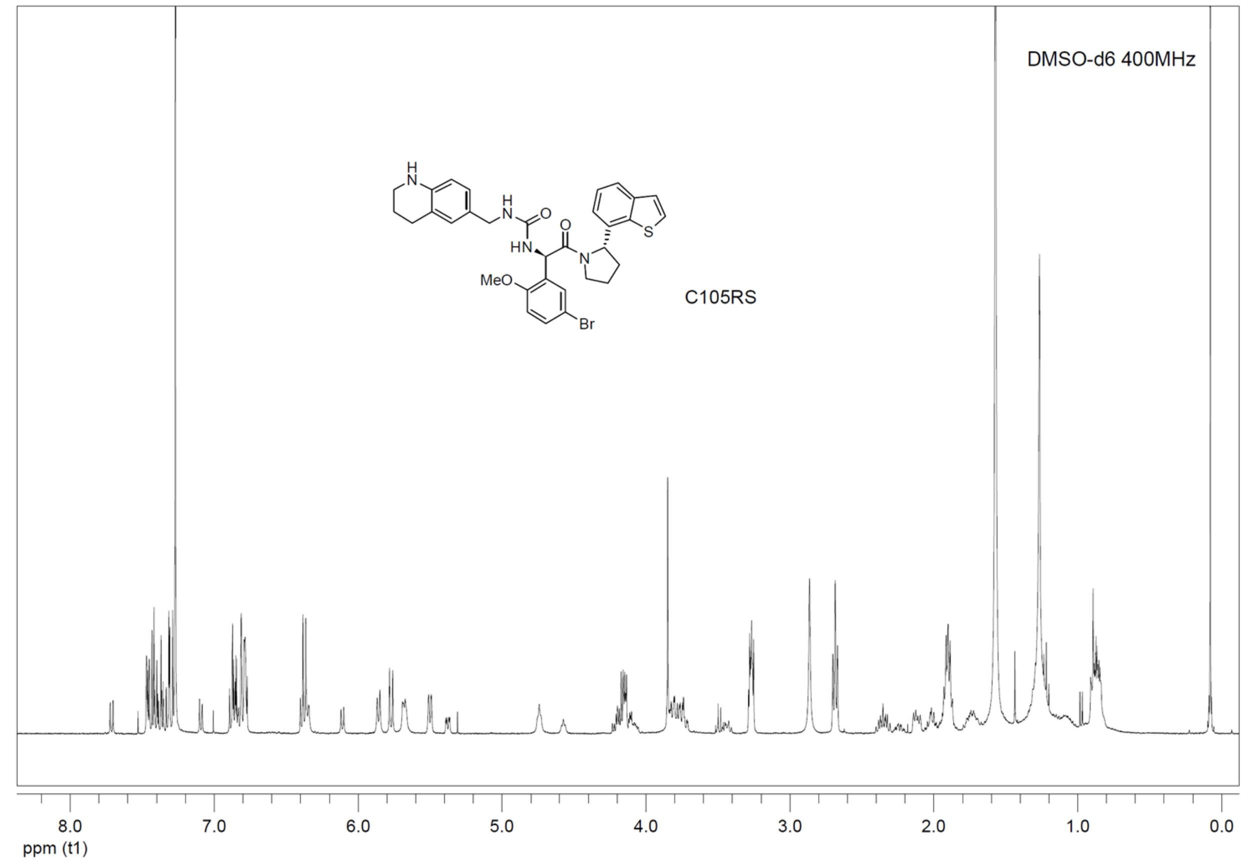


$^1$H NMR (400 MHz, DMSO-d6) spectrum of compound C105RS.

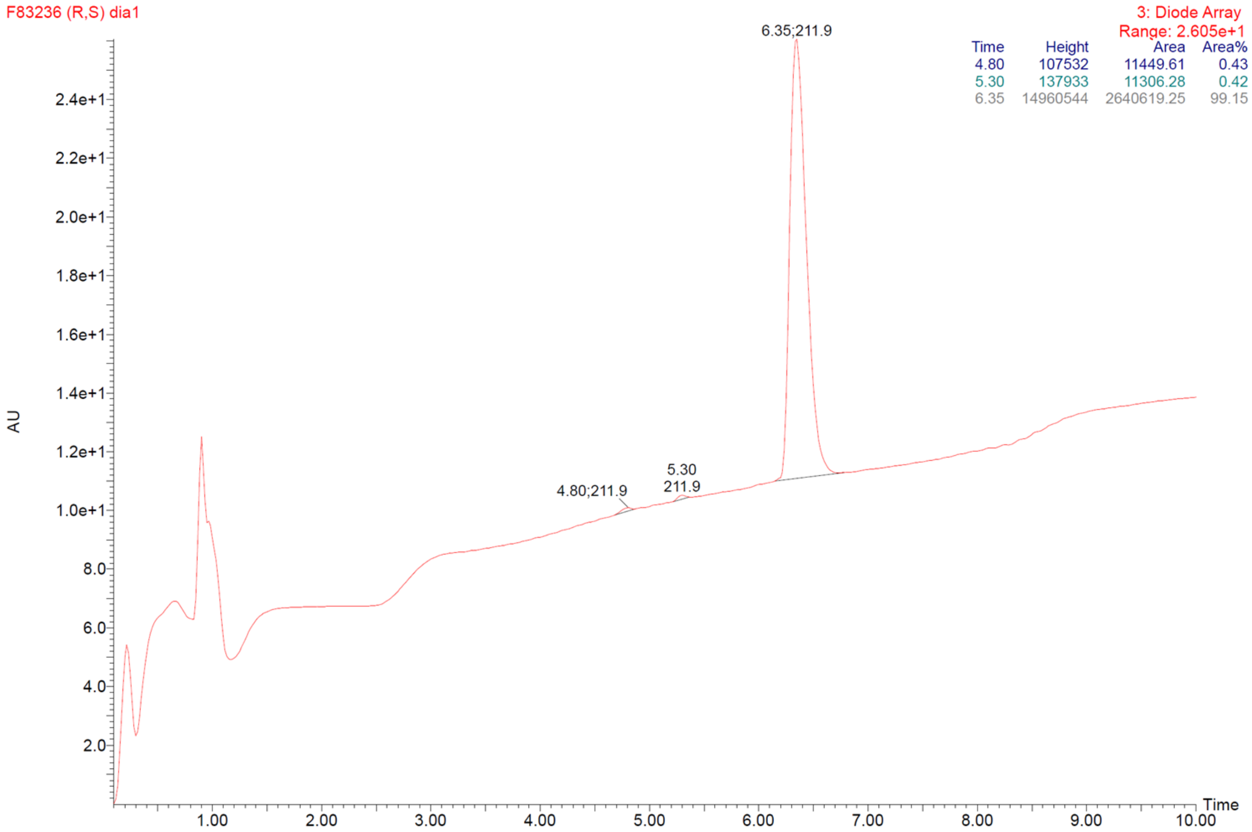


HPLC Chromatogram of compound C105RS.

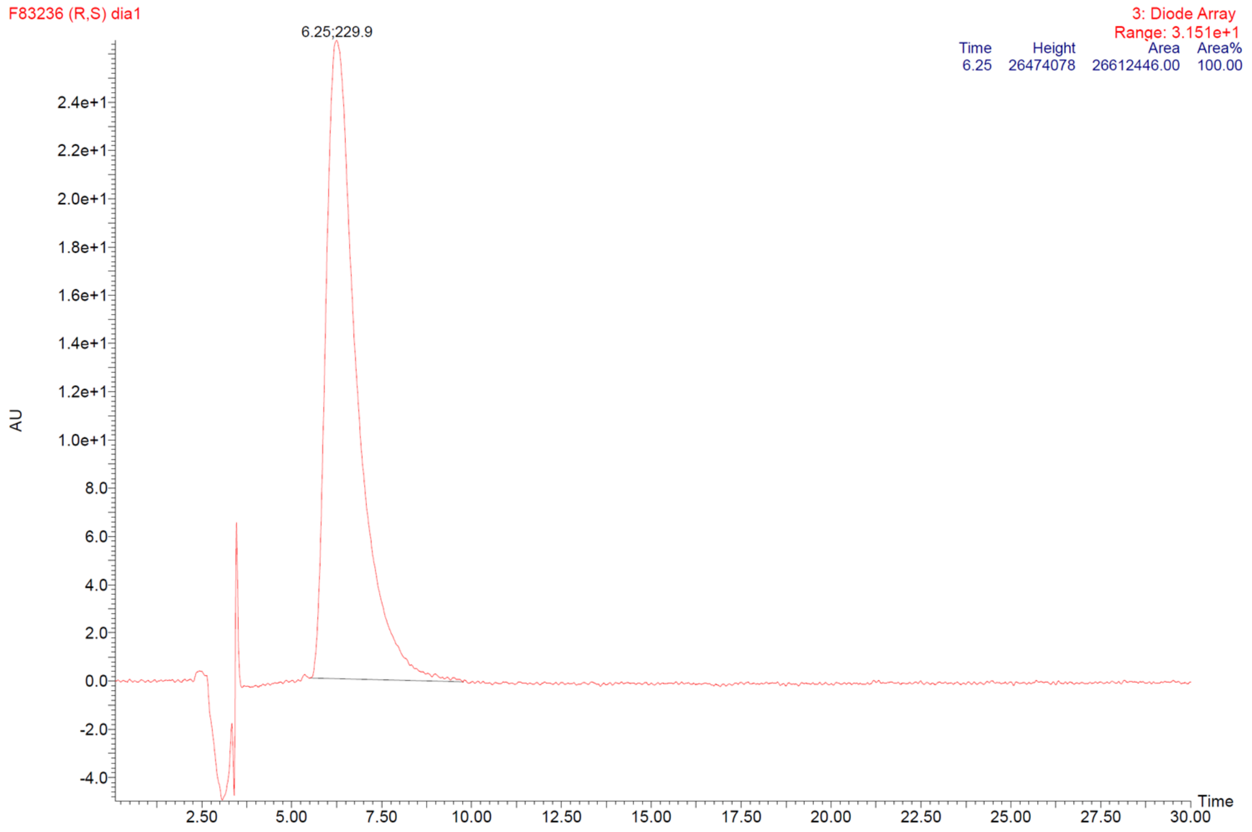


Chiral HPLC Chromatogram of compound C105RS.

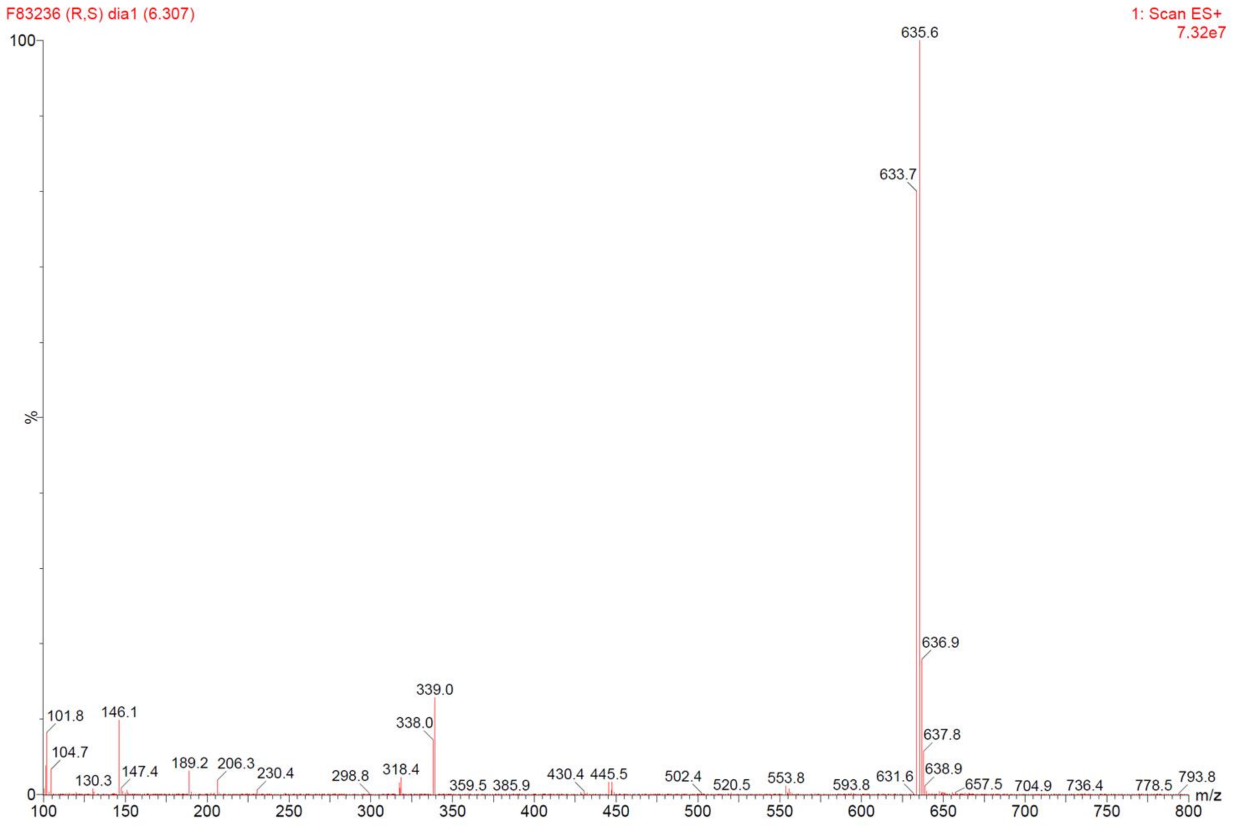


Mass spectrum of compound C105RS.

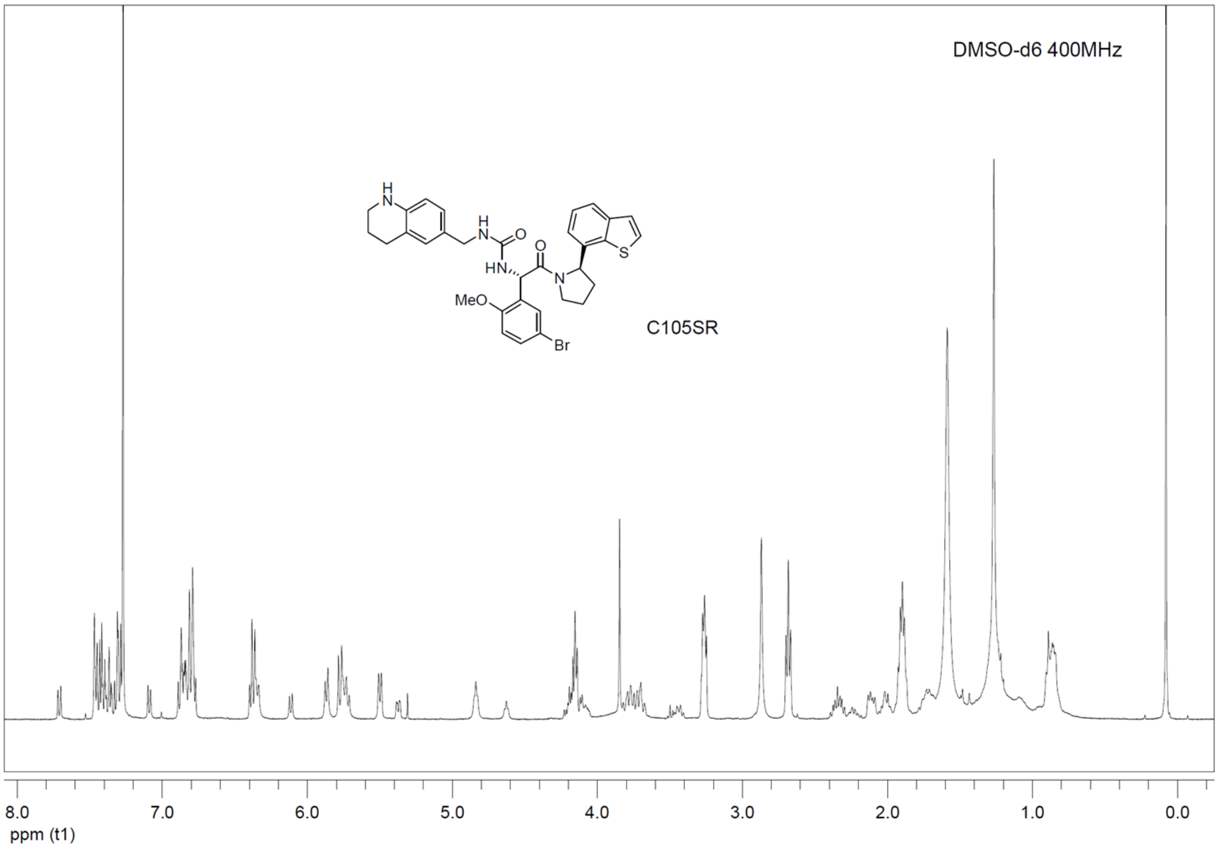

$^{1}$H NMR (400 MHz, DMSO-d6) spectrum of compound C105SR.

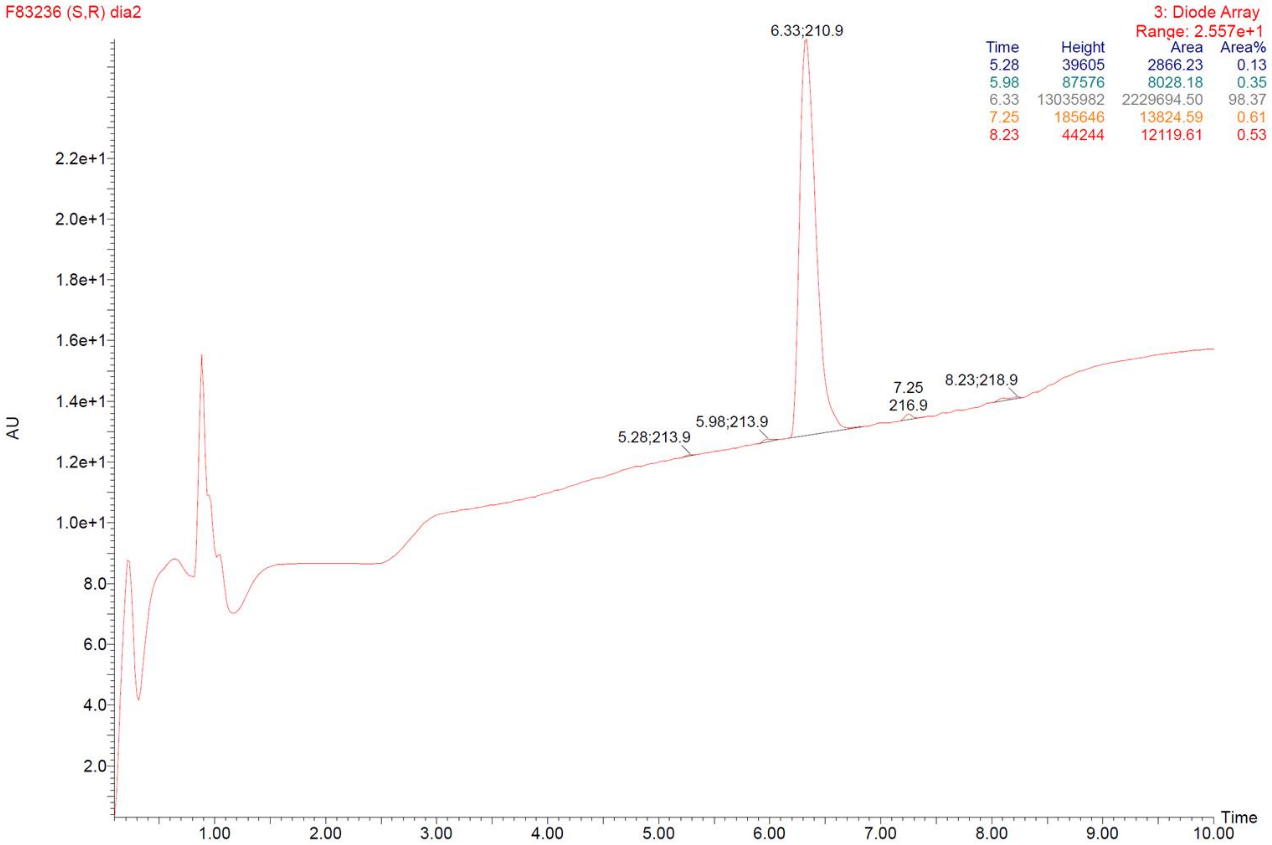

HPLC Chromatogram of compound C105SR.

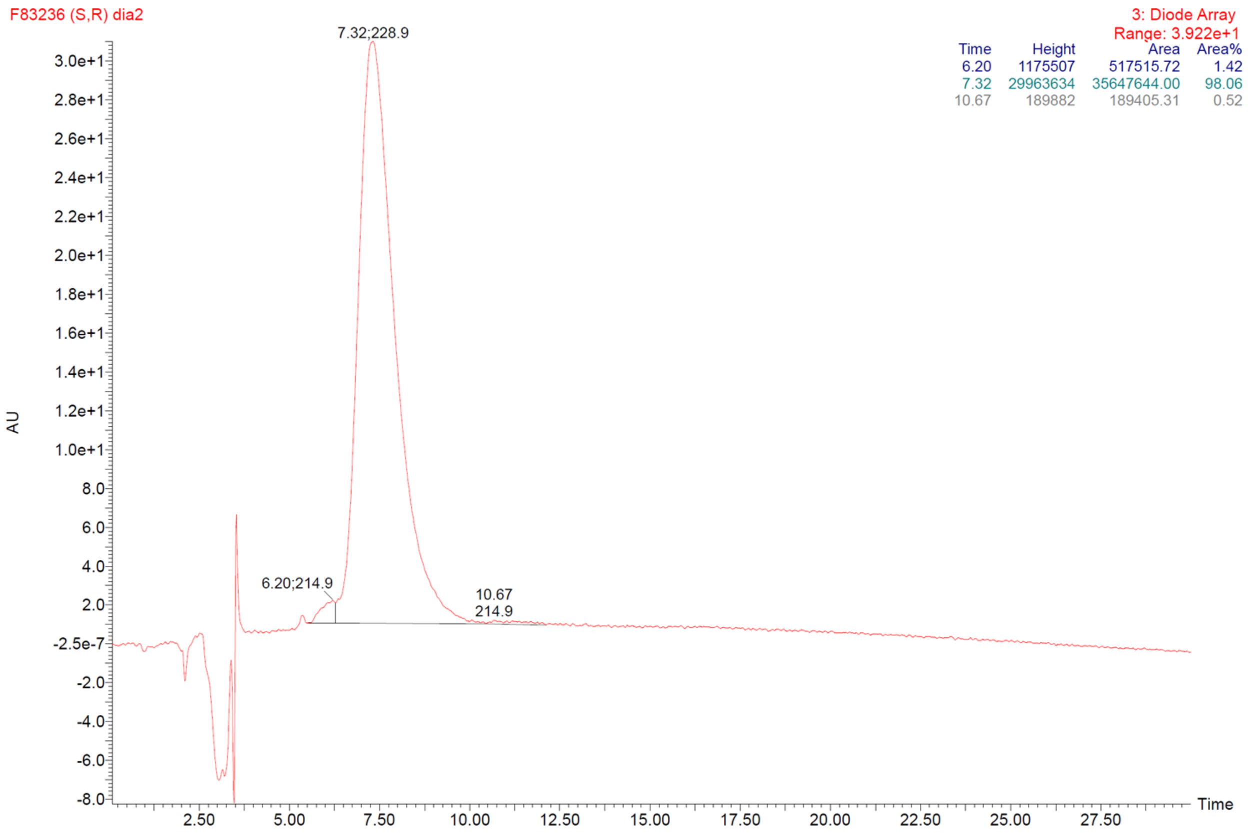


Chiral HPLC Chromatogram of compound C105SR.

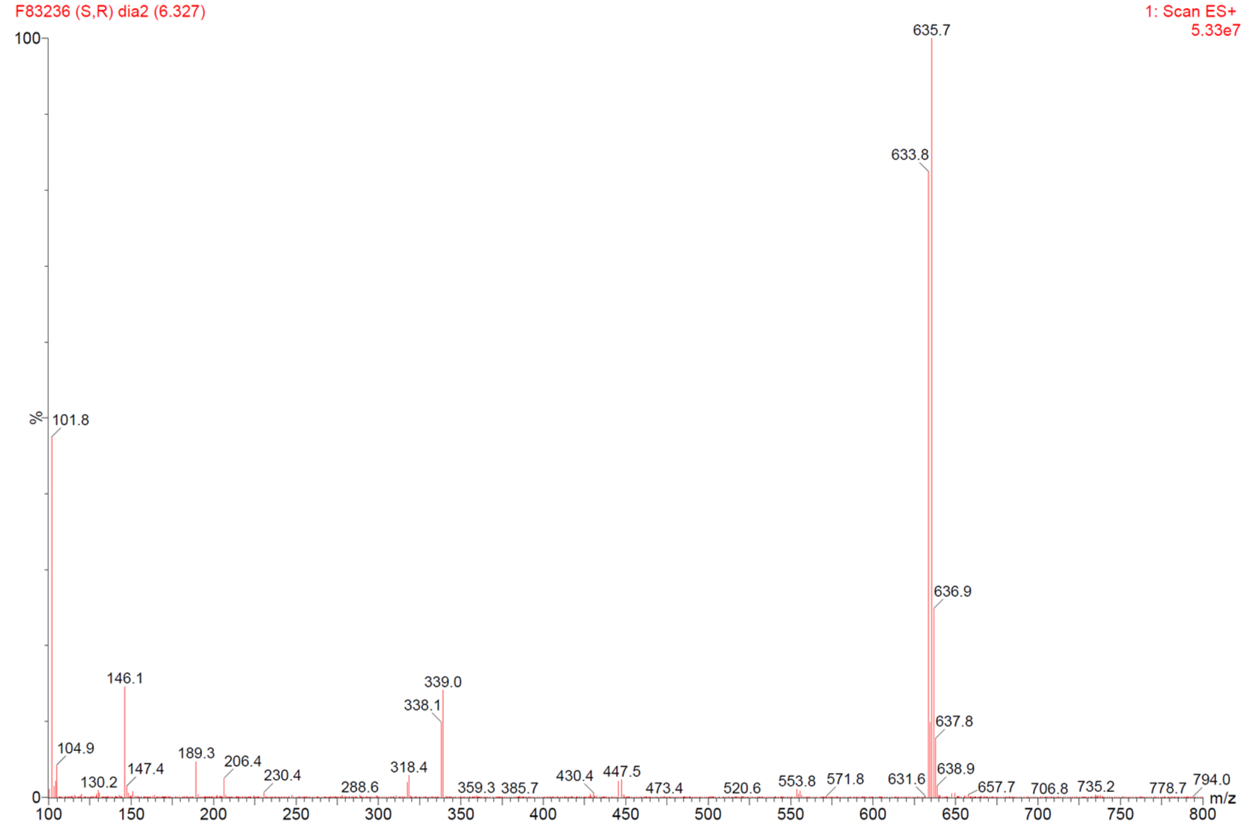


Mass spectrum of compound C105SR.

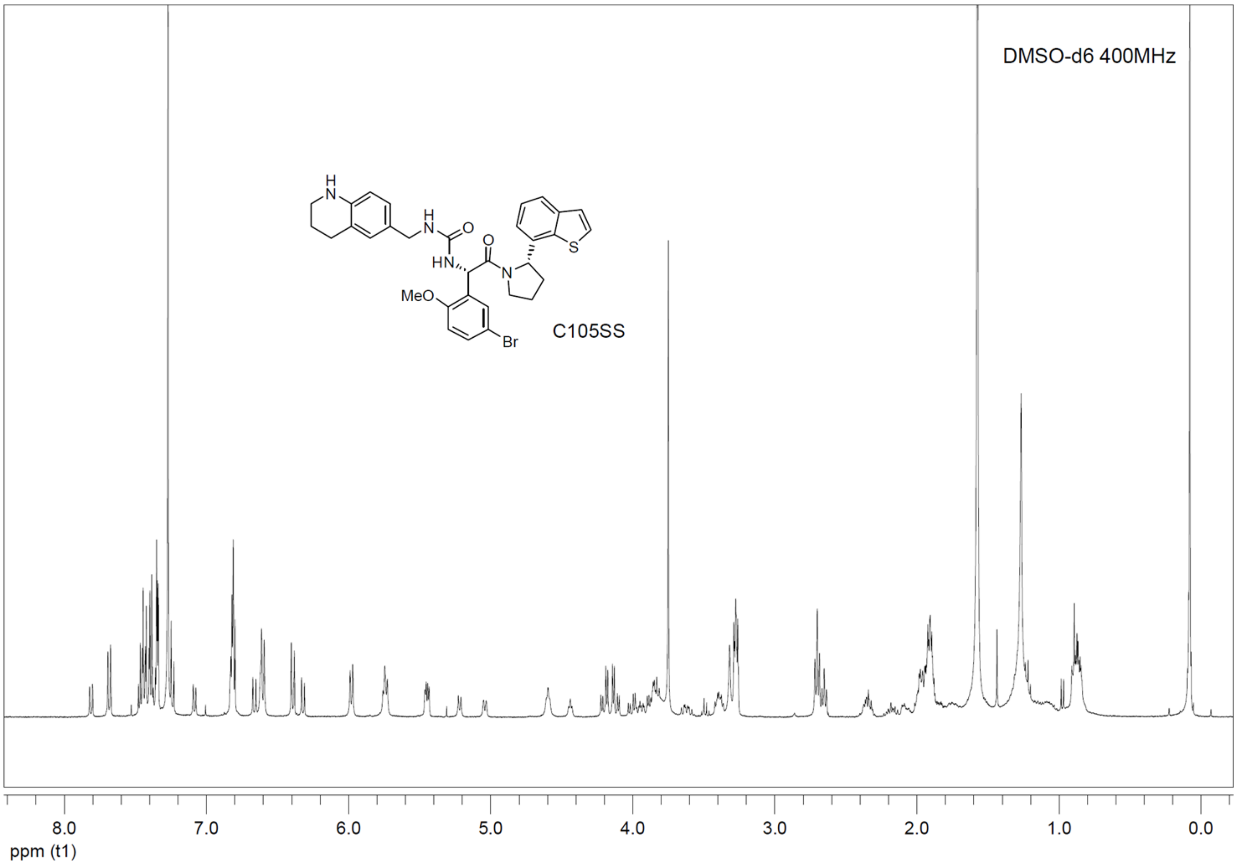


$^{1}$H NMR (400 MHz, DMSO-d6) spectrum of compound C105SS.

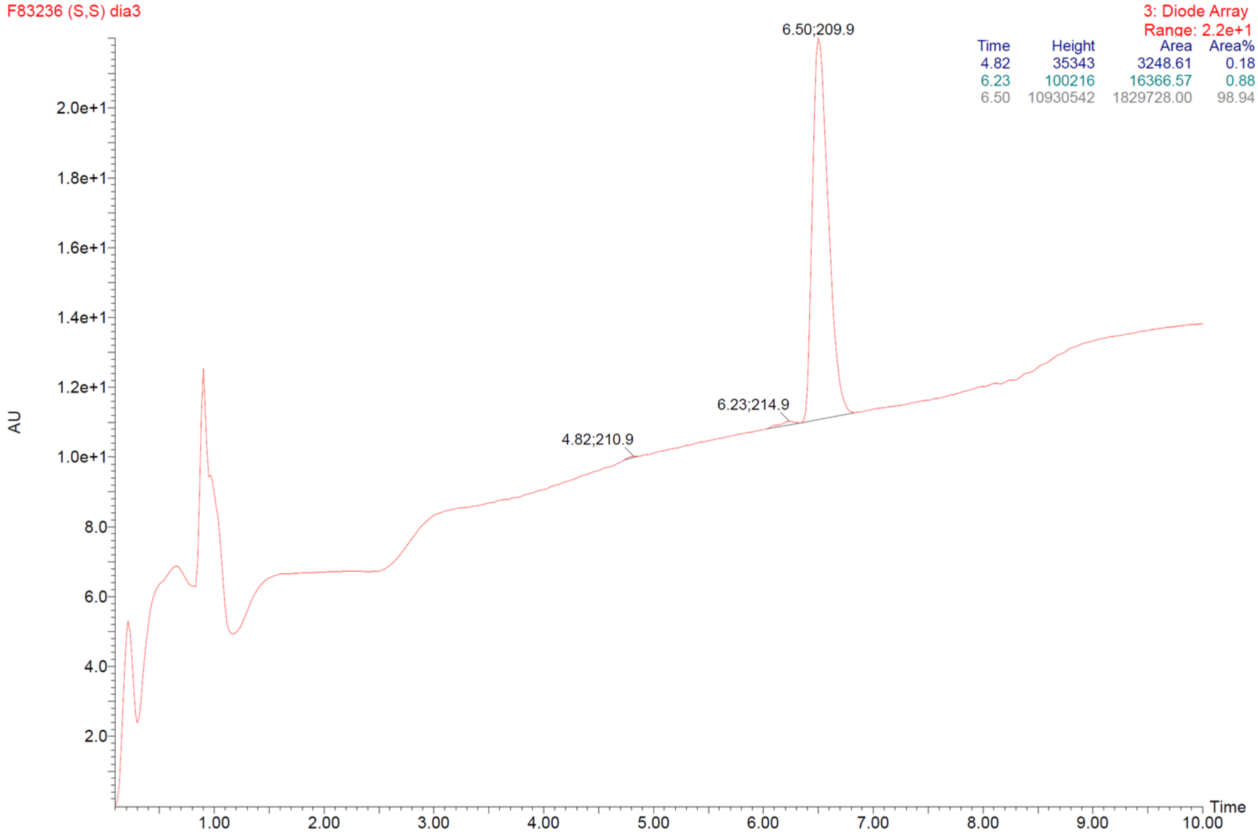


HPLC Chromatogram of compound C105SS.

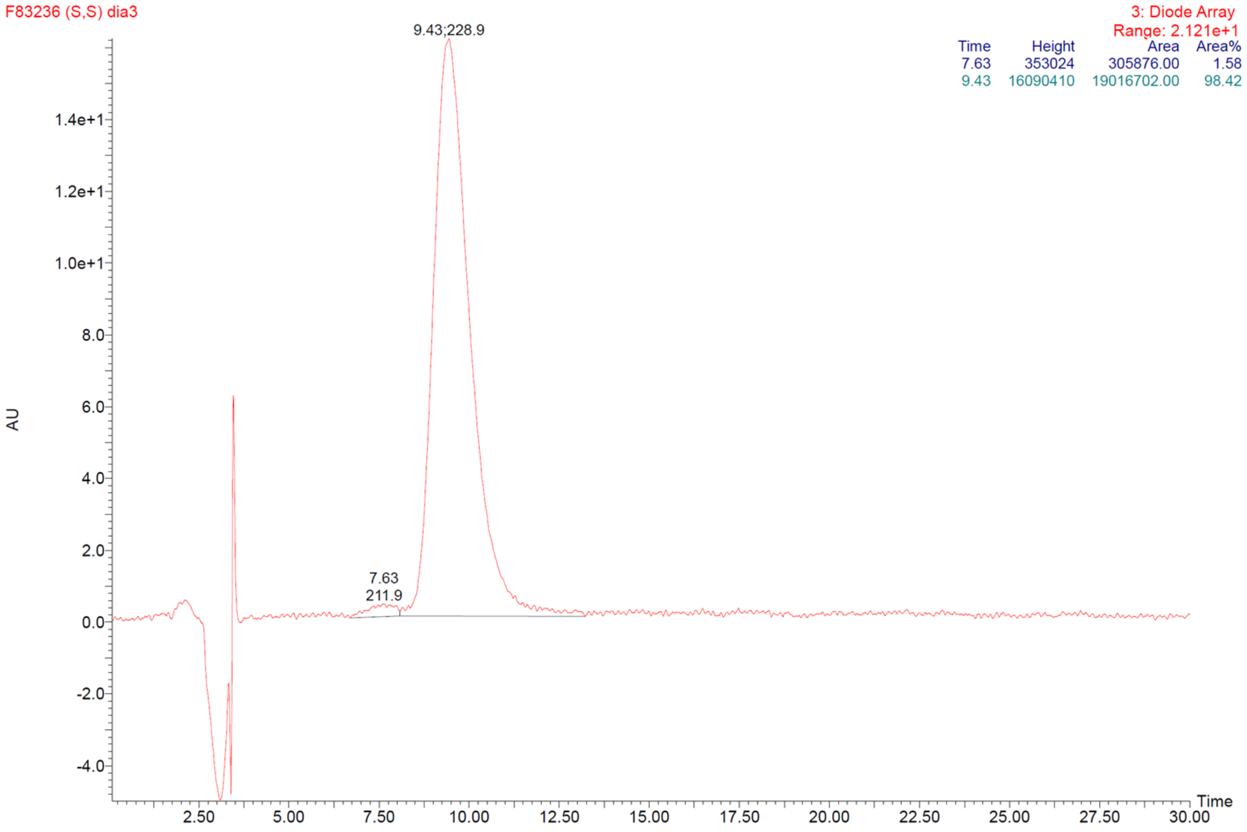


Chiral HPLC Chromatogram of compound C105SS.

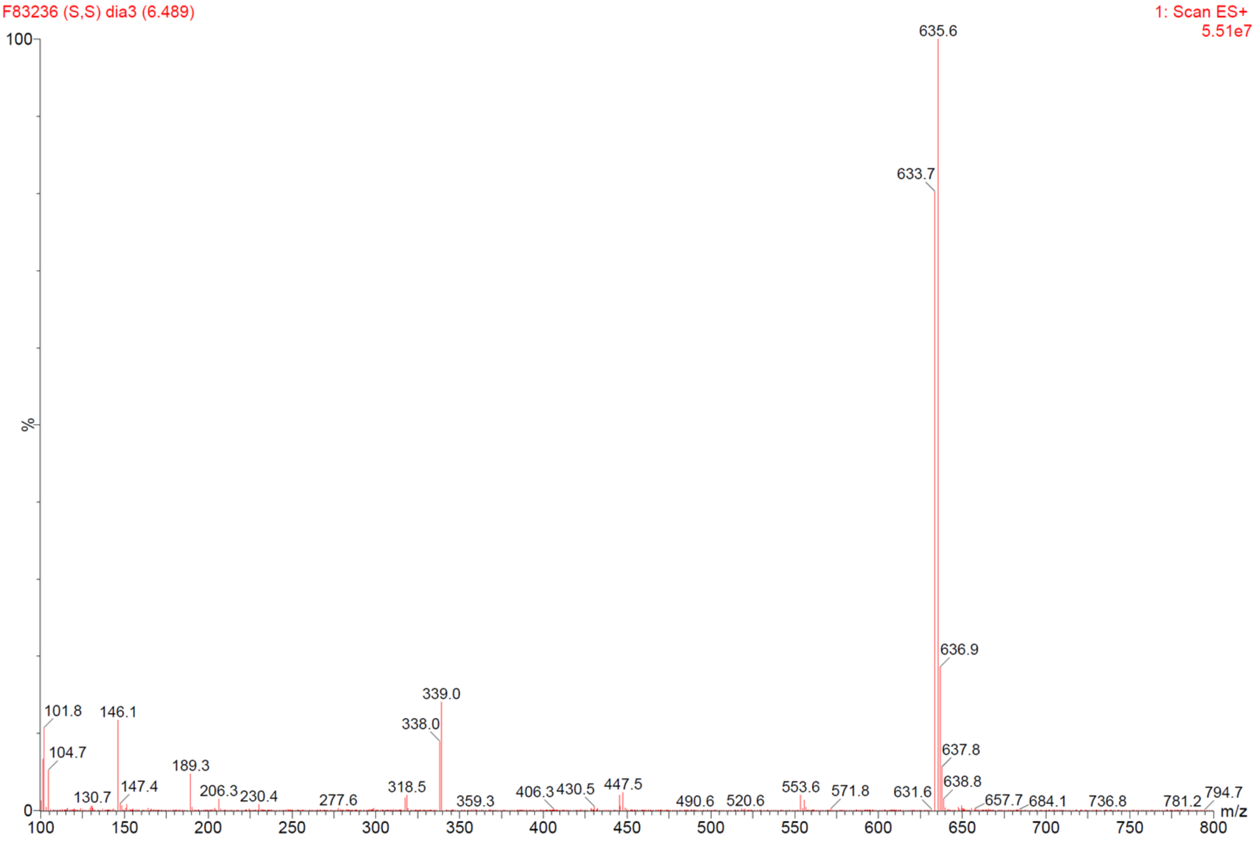


Mass spectrum of compound C105SS.

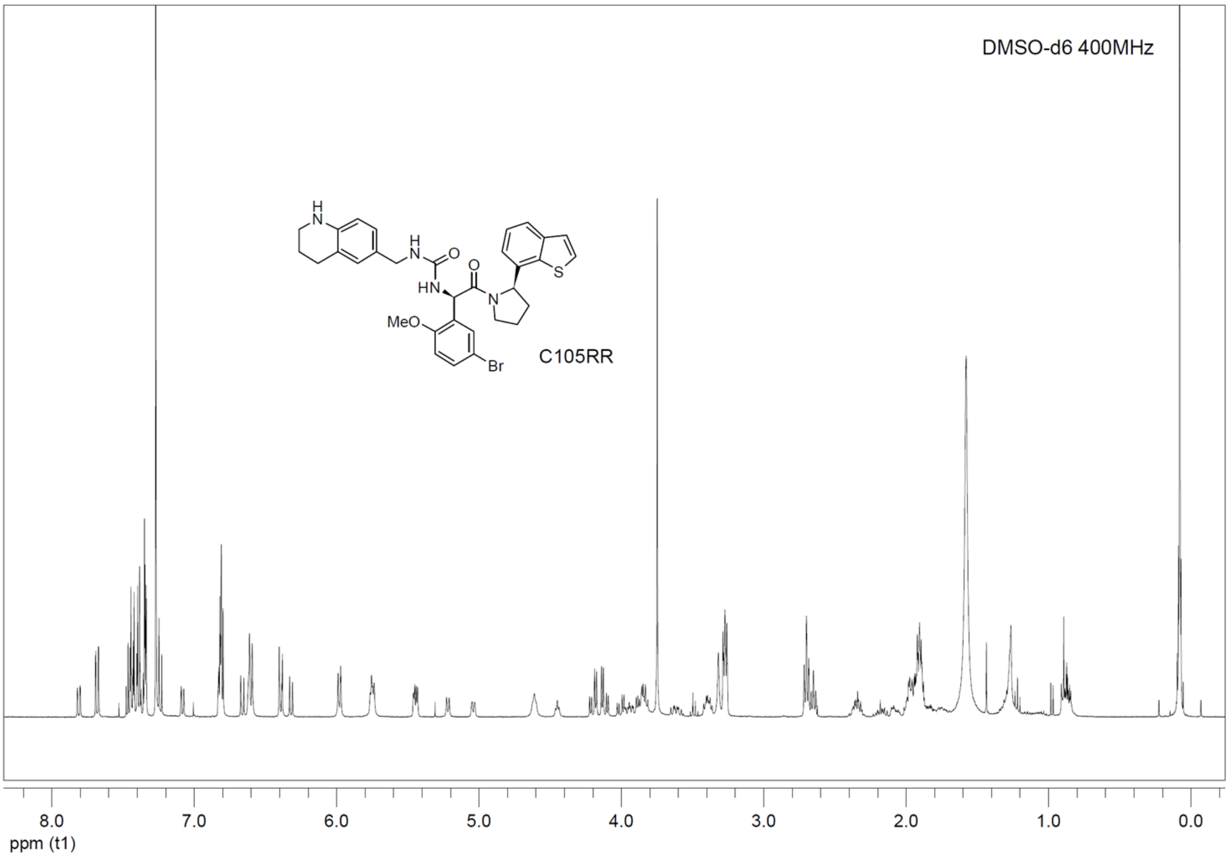


$^{1}$H NMR (400 MHz, DMSO-d6) spectrum of compound C105RR.

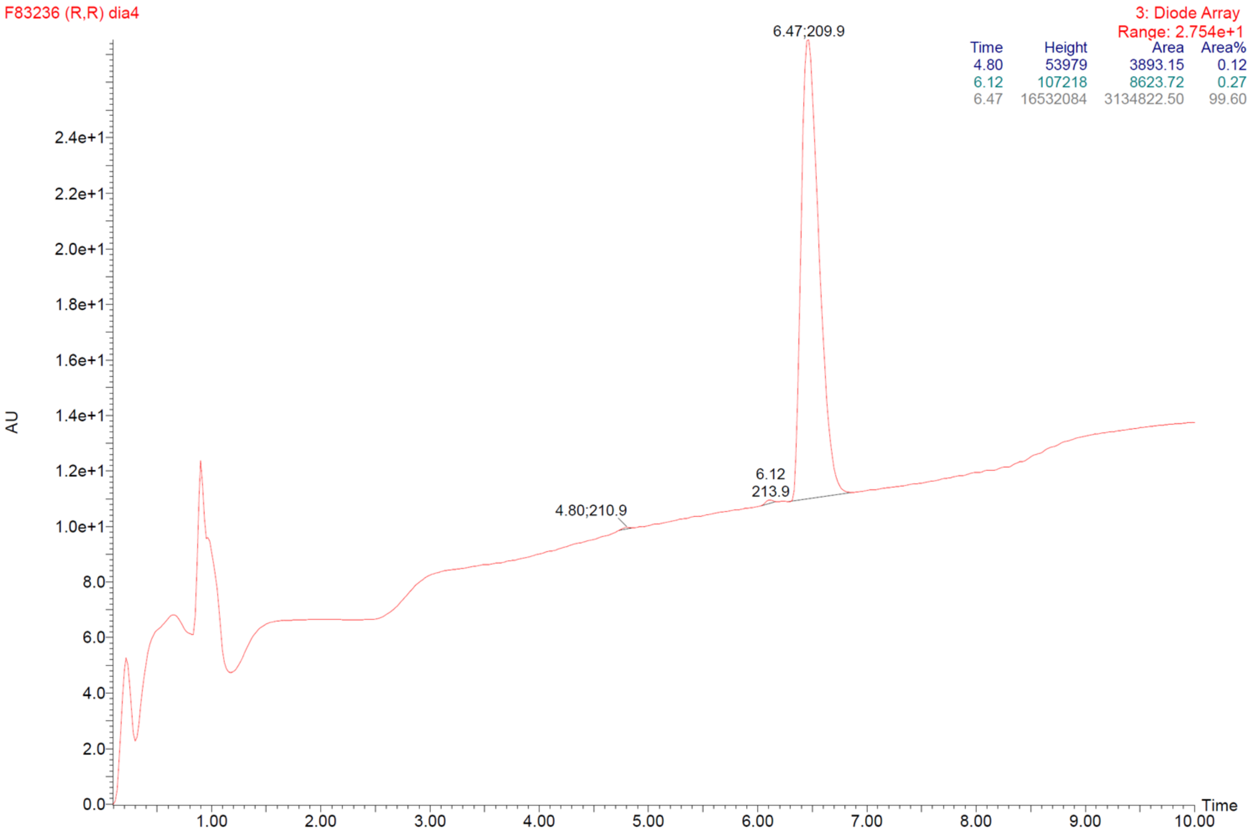


HPLC Chromatogram of compound C105RR.

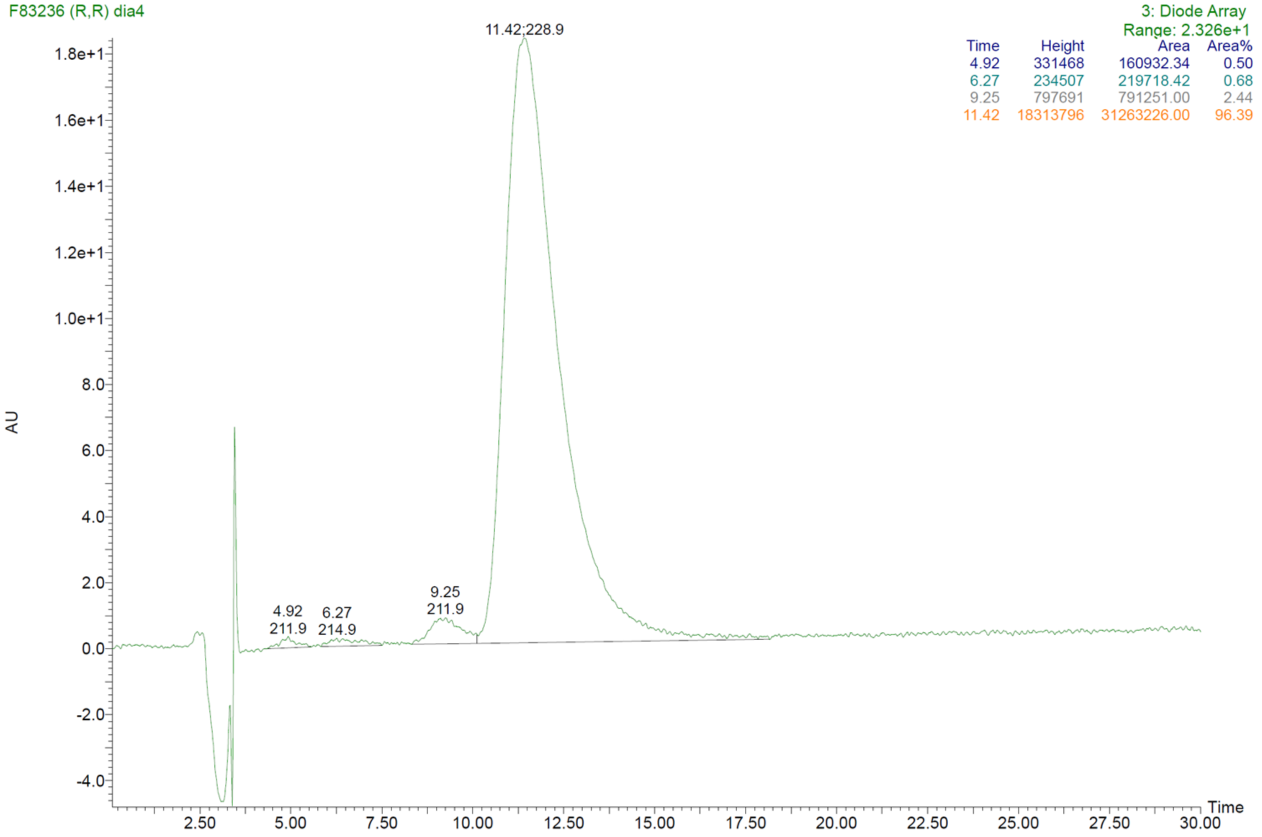


Chiral HPLC Chromatogram of compound C105RR.

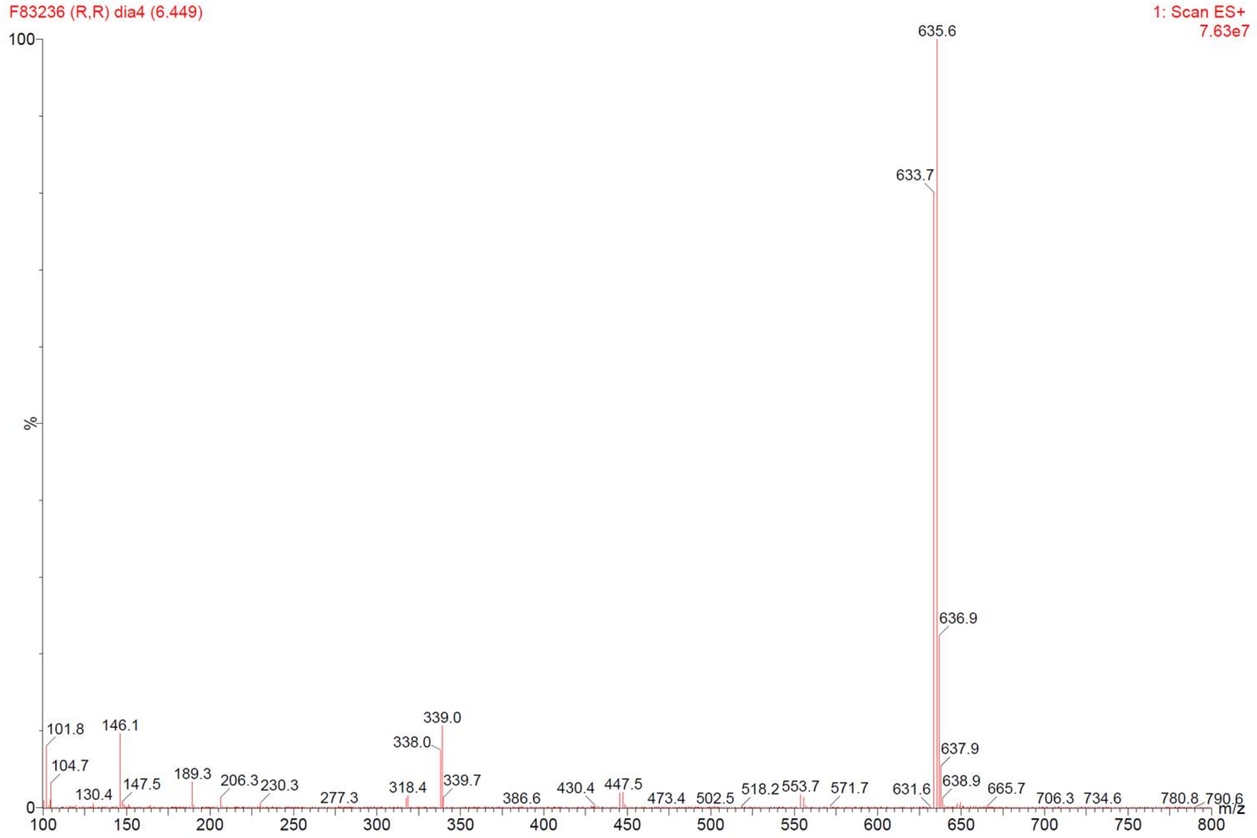


Mass spectrum of compound C105RR.

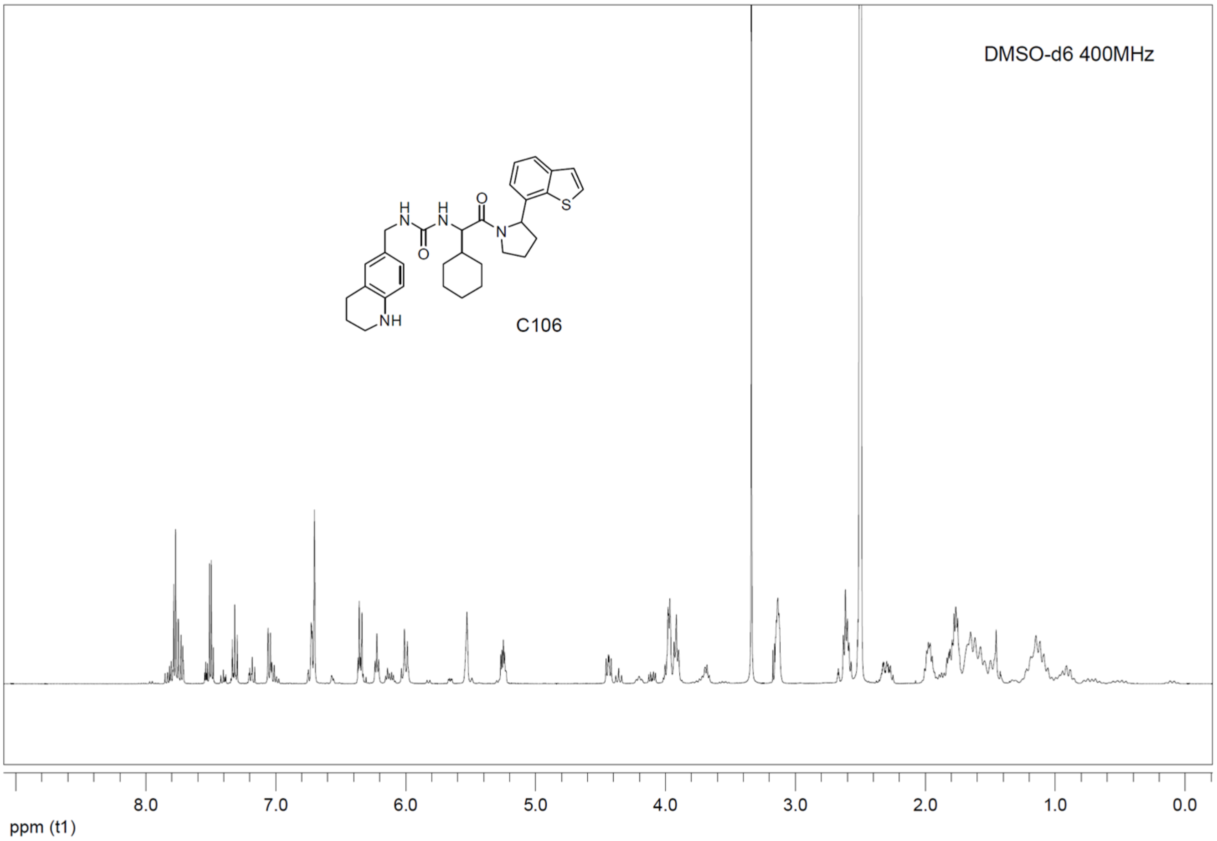


$^{1}$H NMR (400 MHz, DMSO-d6) spectrum of compound C106.

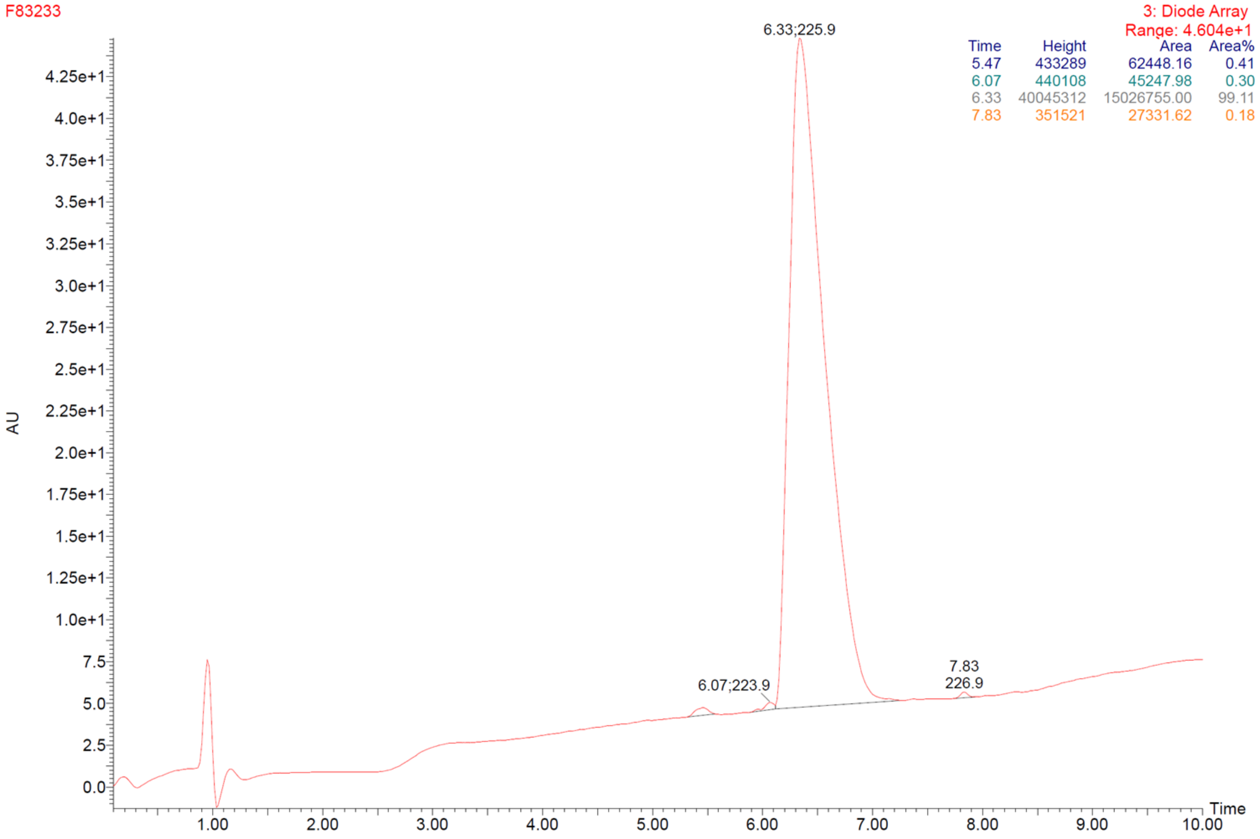


HPLC Chromatogram of compound C106.

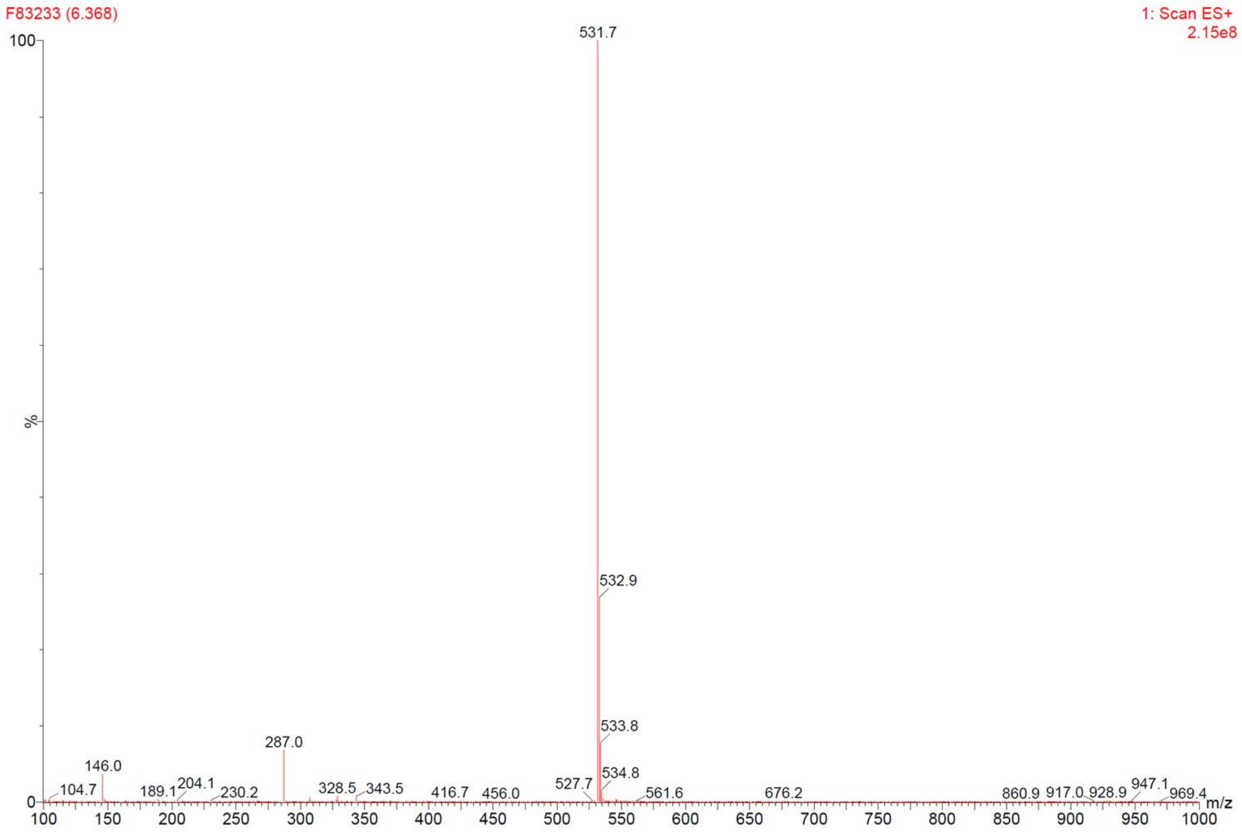


Mass spectrum of compound C106.

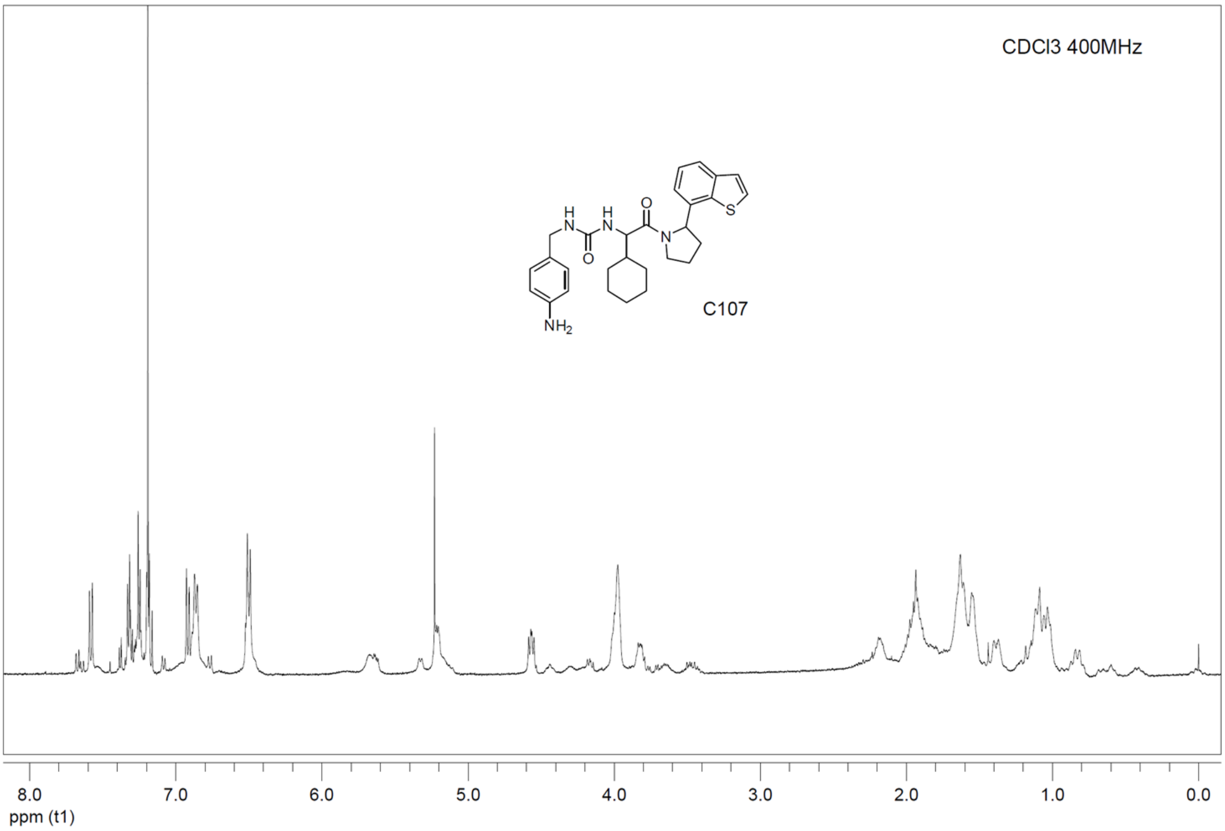


$^1H$ NMR (400 MHz, $CDCl_3$) spectrum of compound C107.

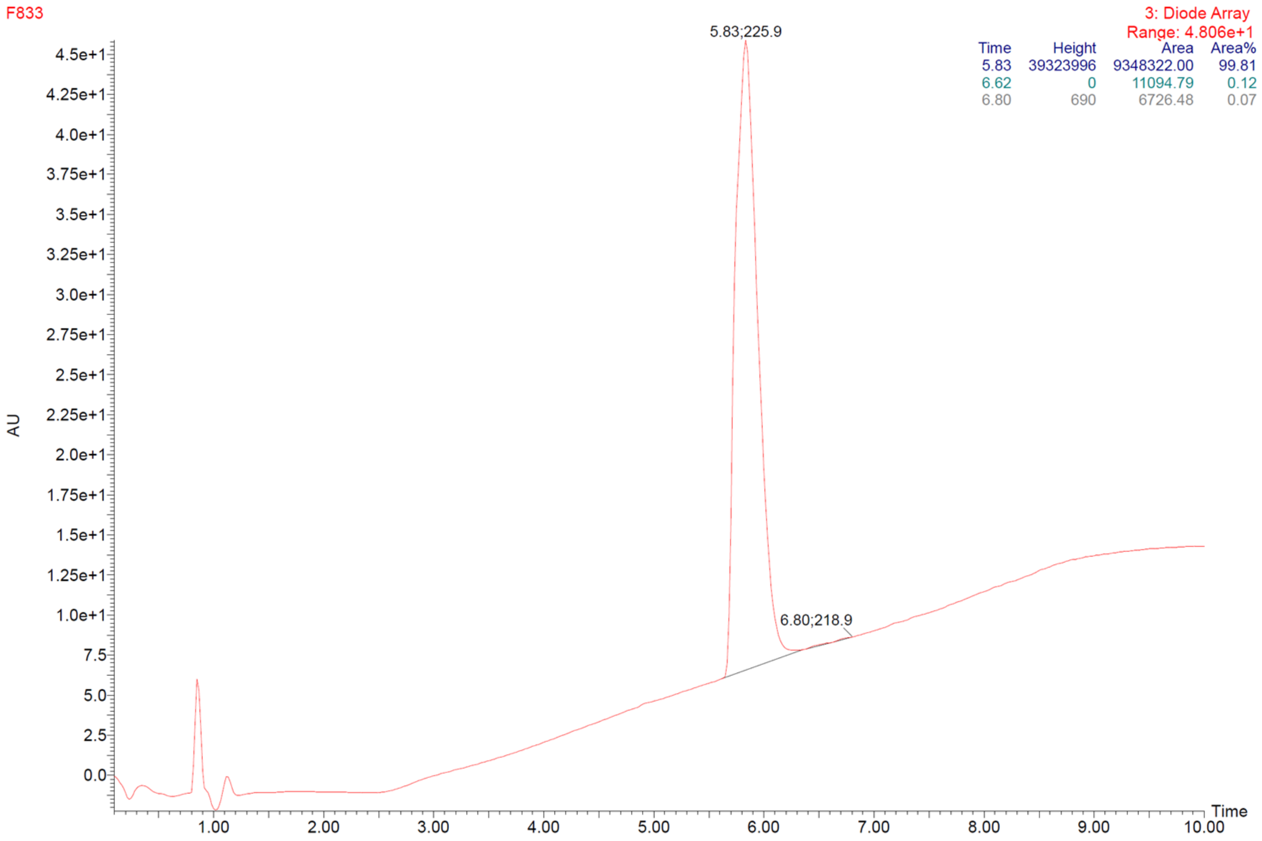


HPLC Chromatogram of compound C107.

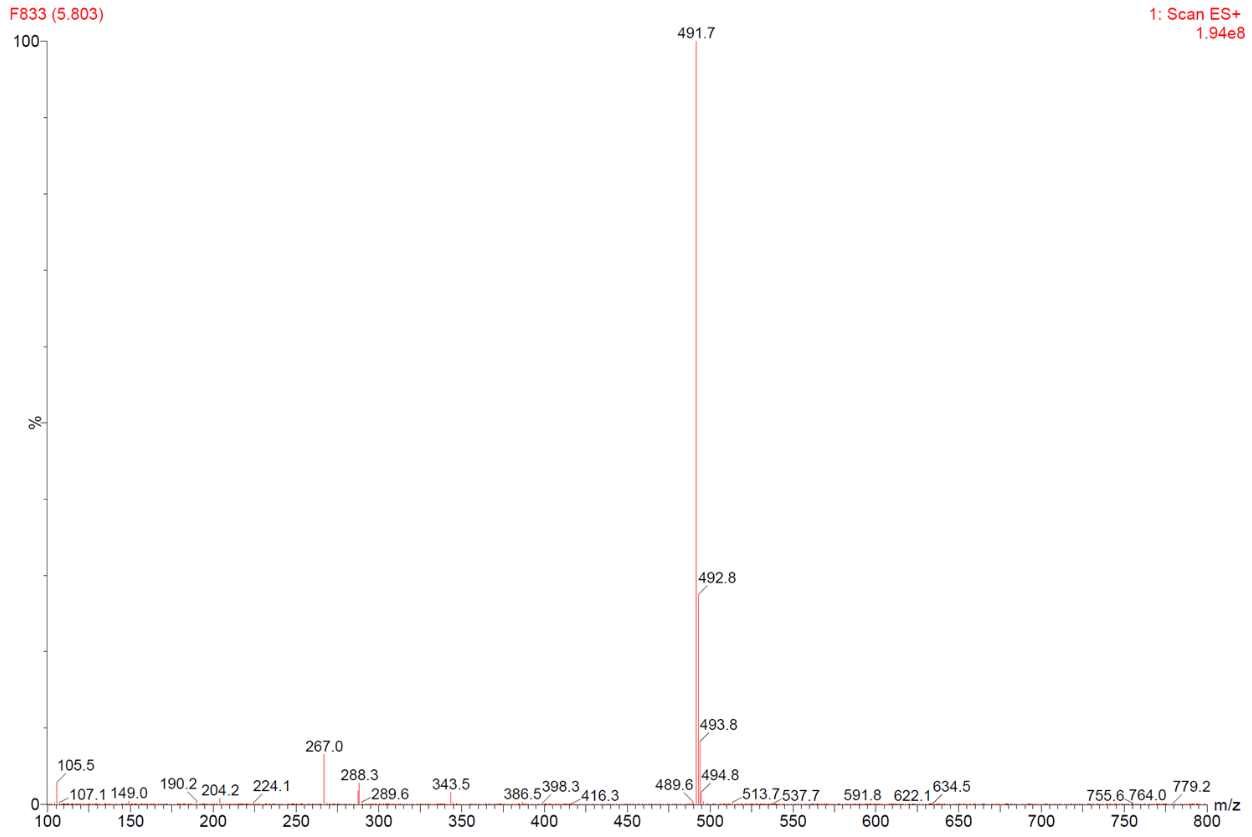


Mass spectrum of compound C107.

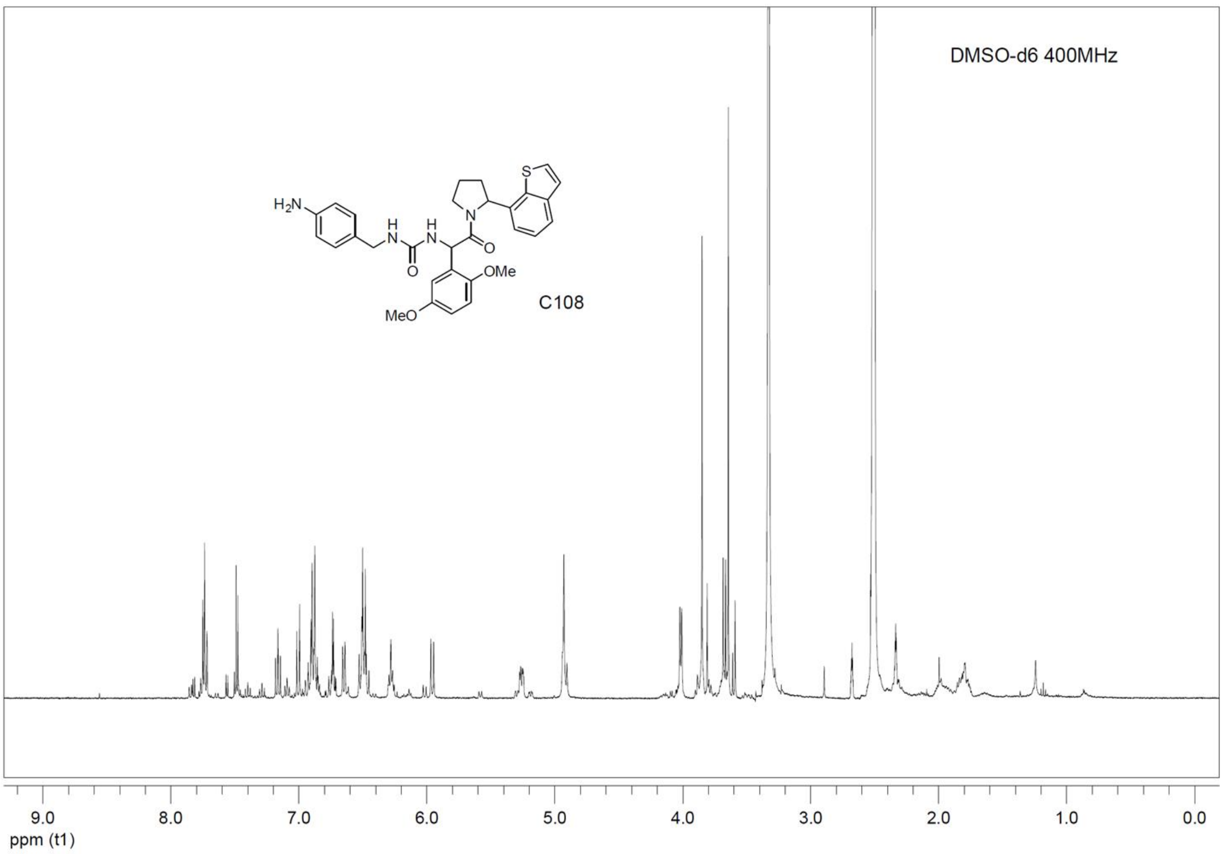


$^1$H NMR (400 MHz, DMSO-d6) spectrum of compound C108.

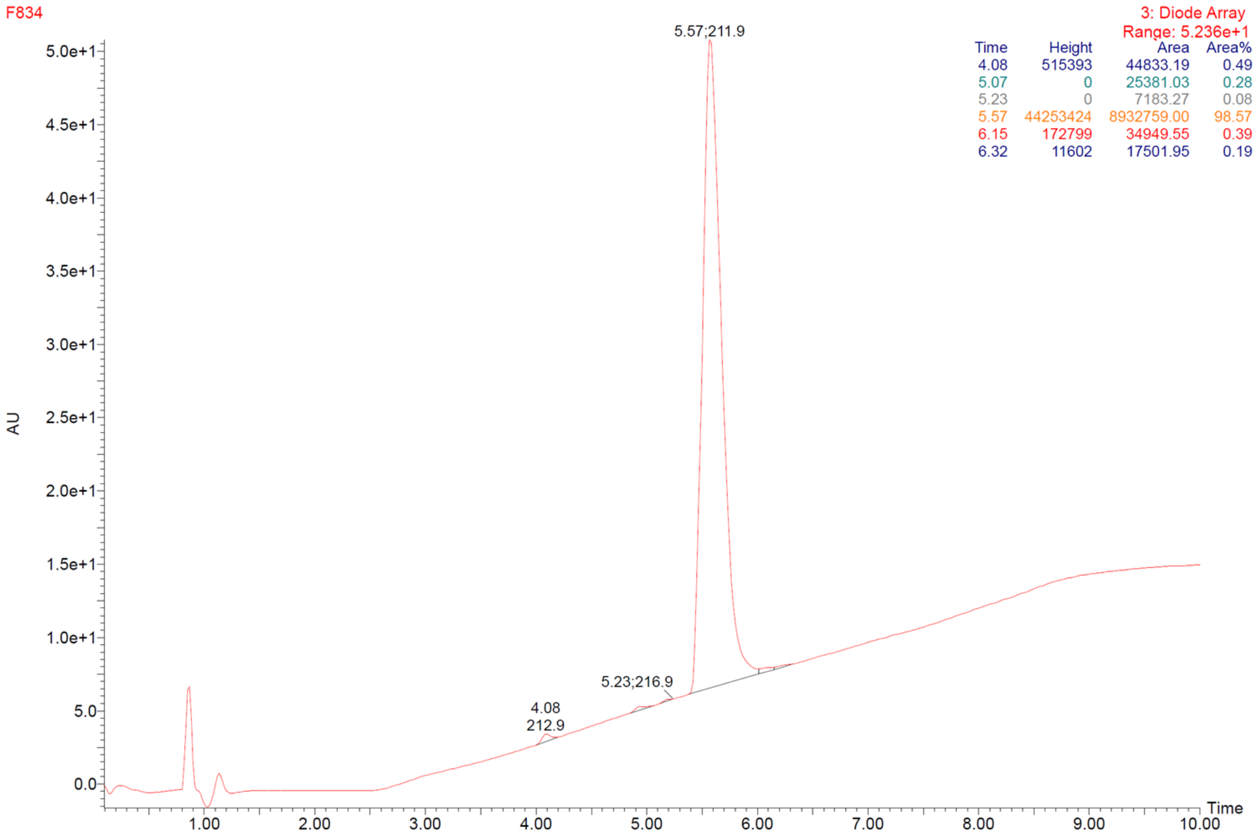


HPLC Chromatogram of compound C108.

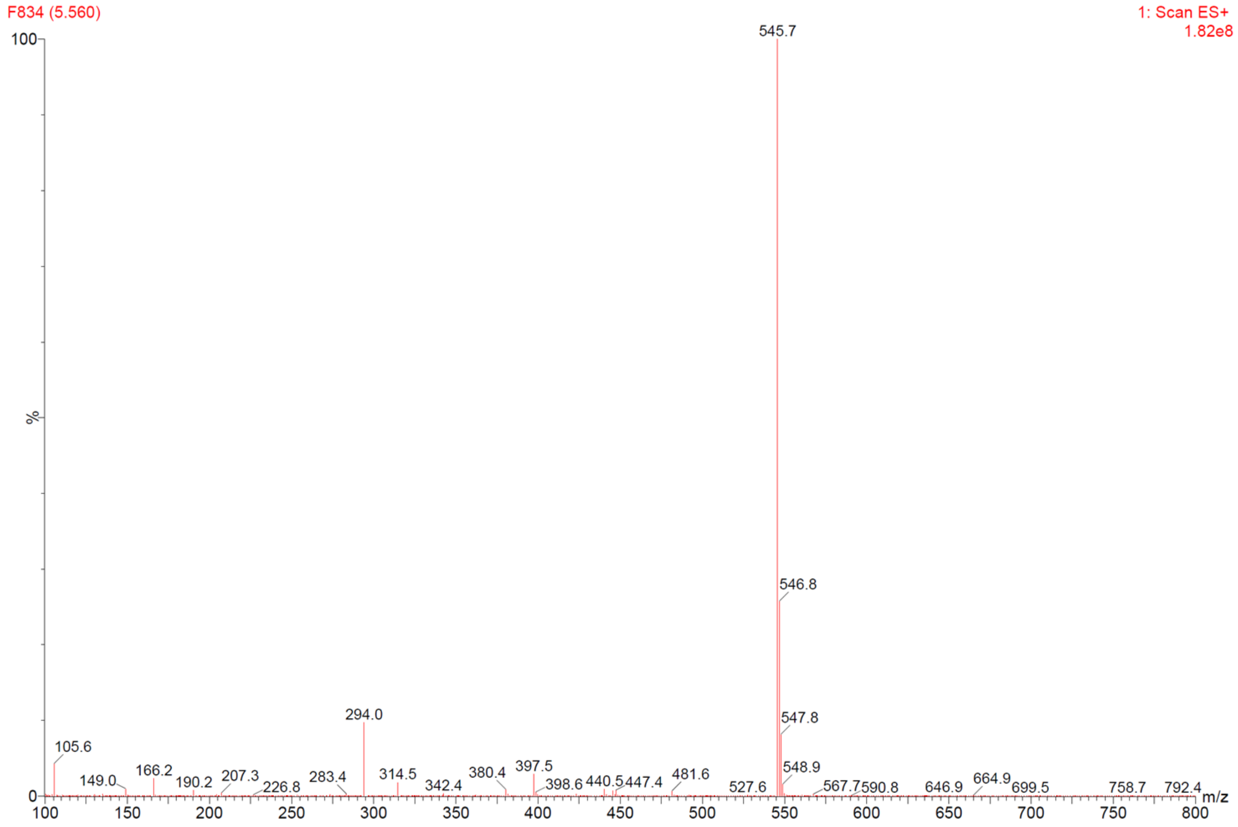


Mass spectrum of compound C108.

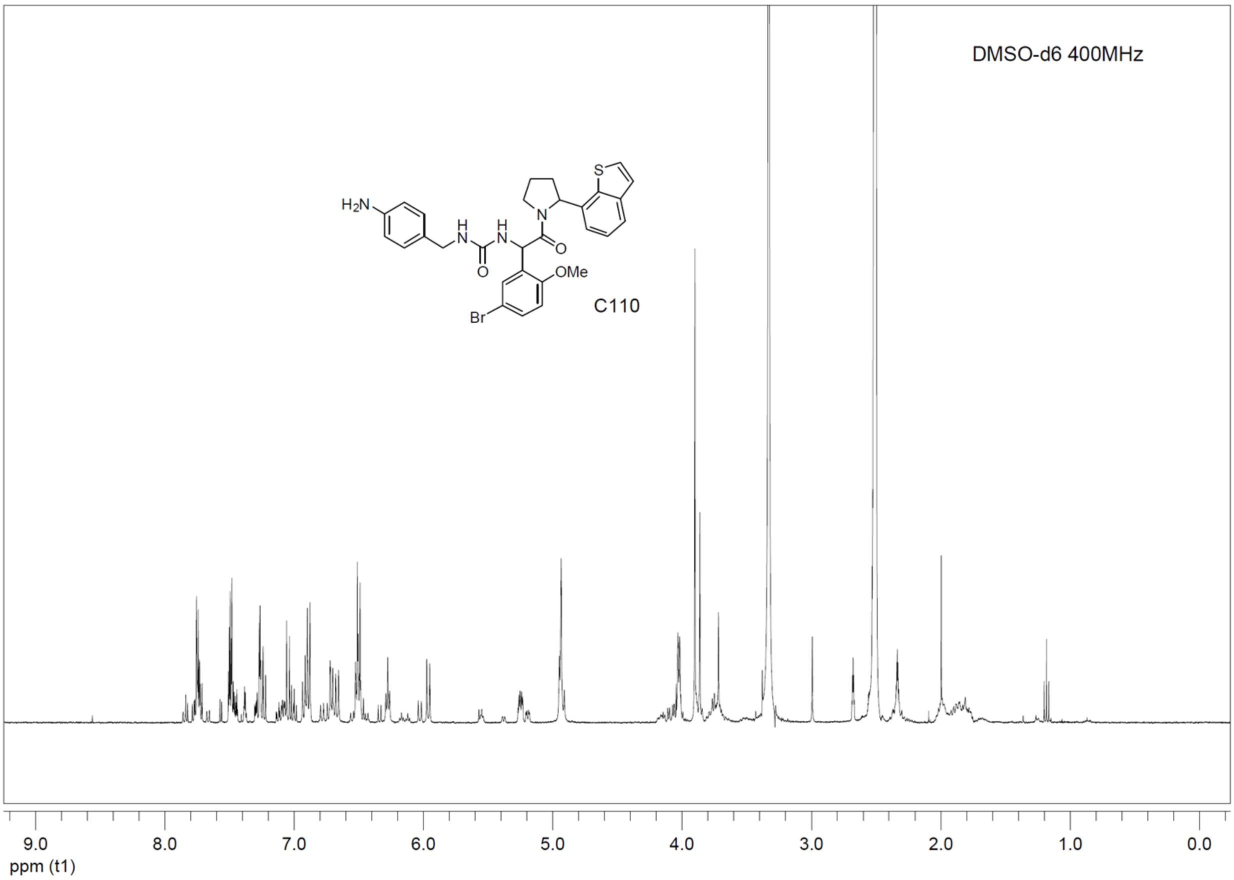


$^{1}$H NMR (400 MHz, DMSO-d6) spectrum of compound C110.

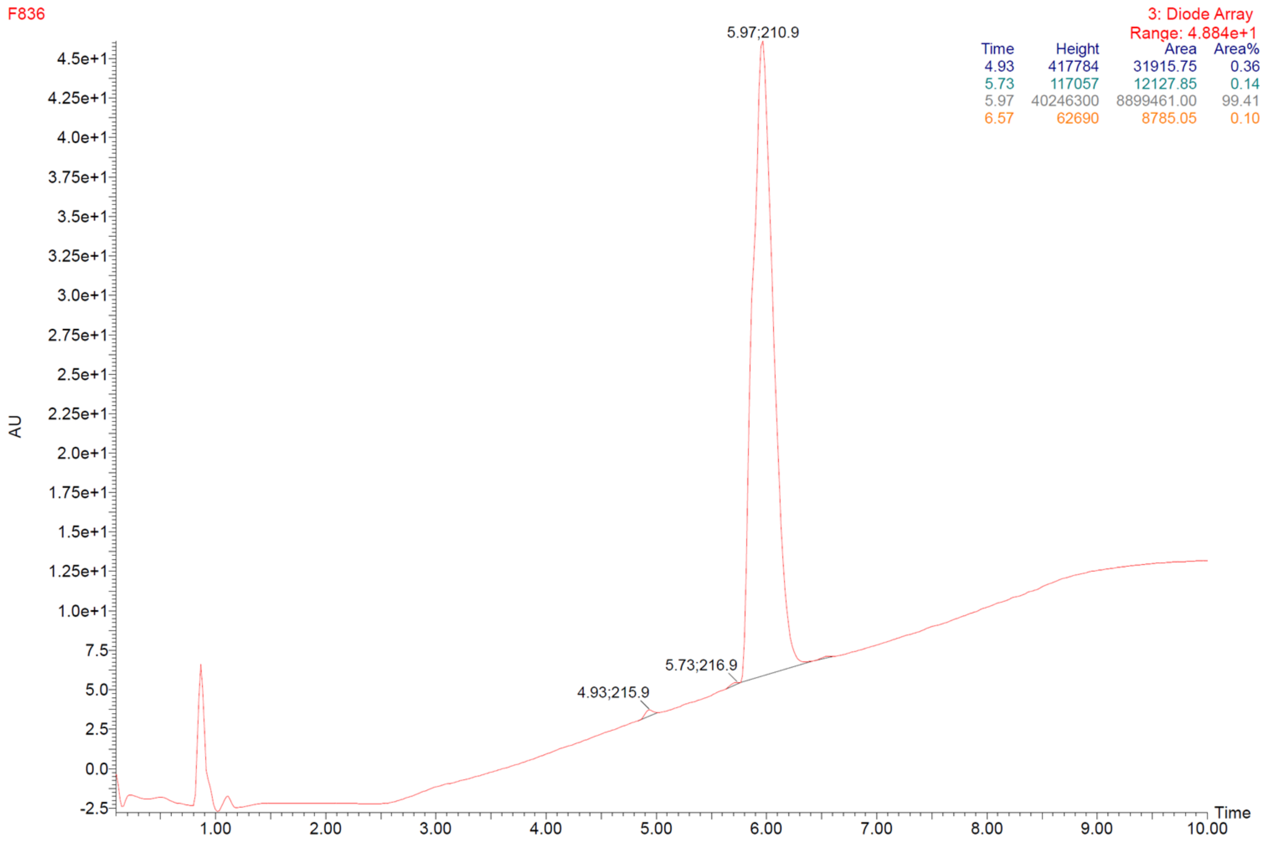


HPLC Chromatogram of compound C110.

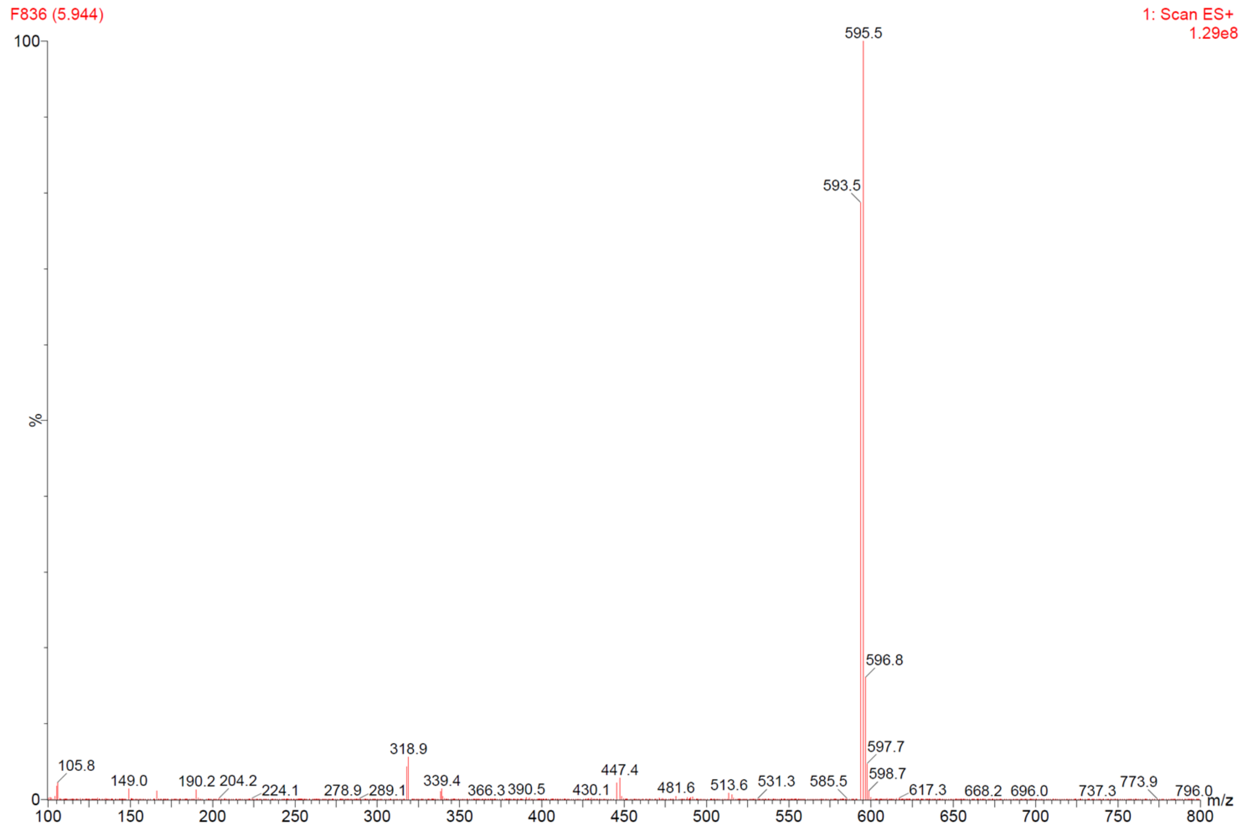


Mass spectrum of compound C110.

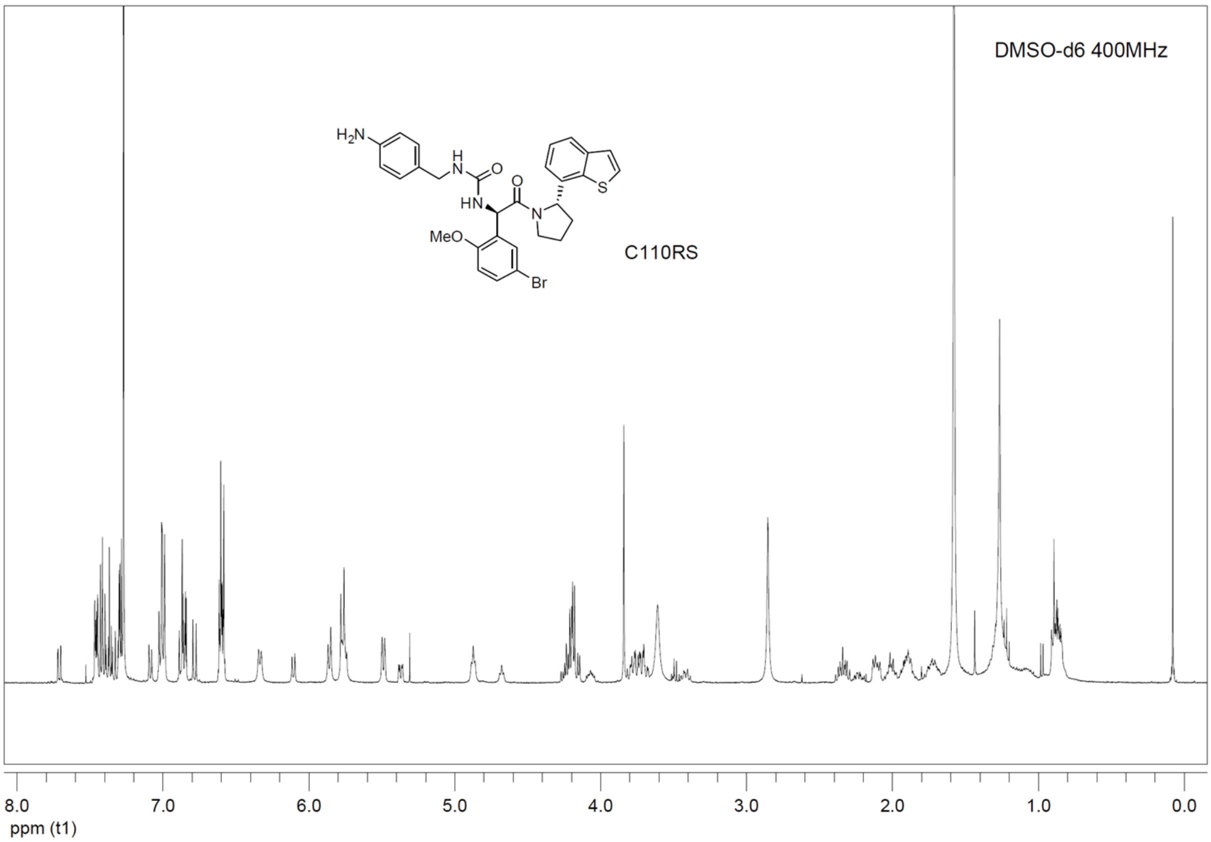


$^{1}$H NMR (400 MHz, DMSO-d6) spectrum of compound C110RS

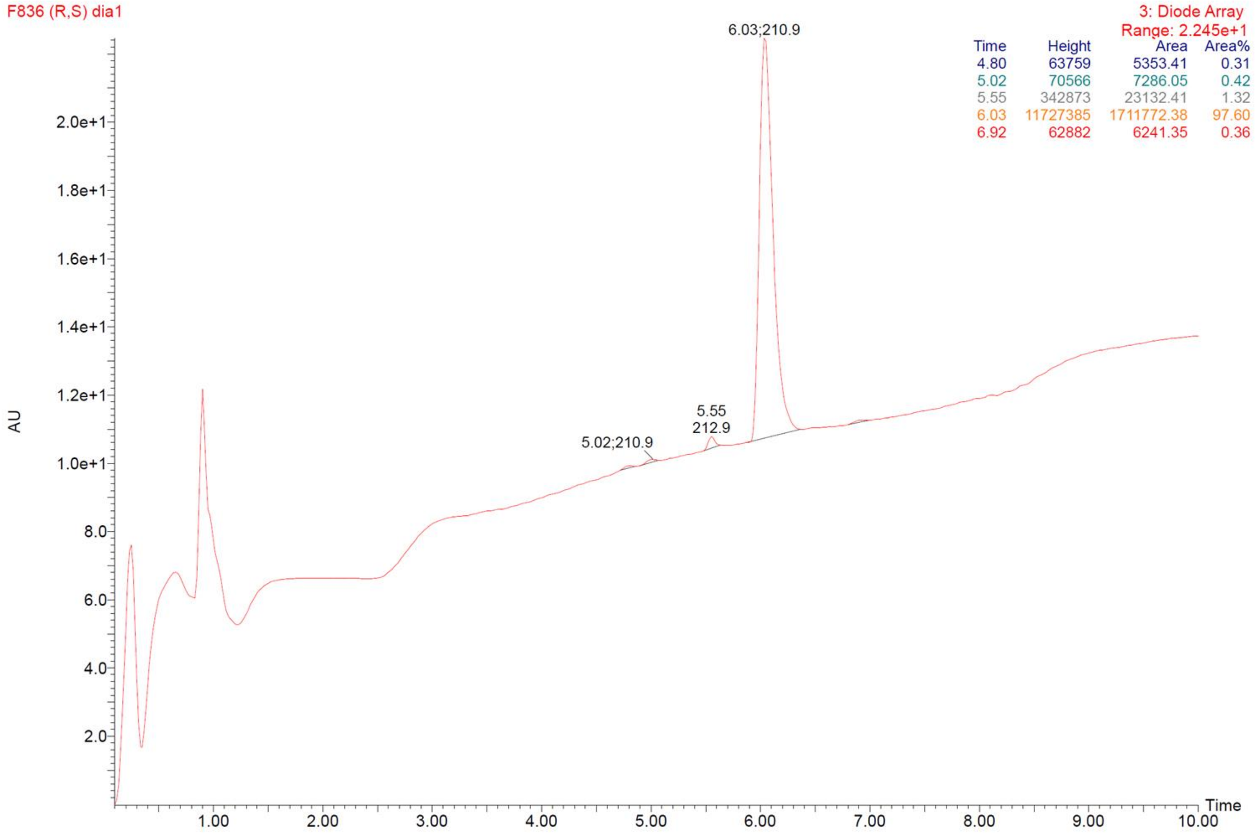


HPLC Chromatogram of compound C110RS.

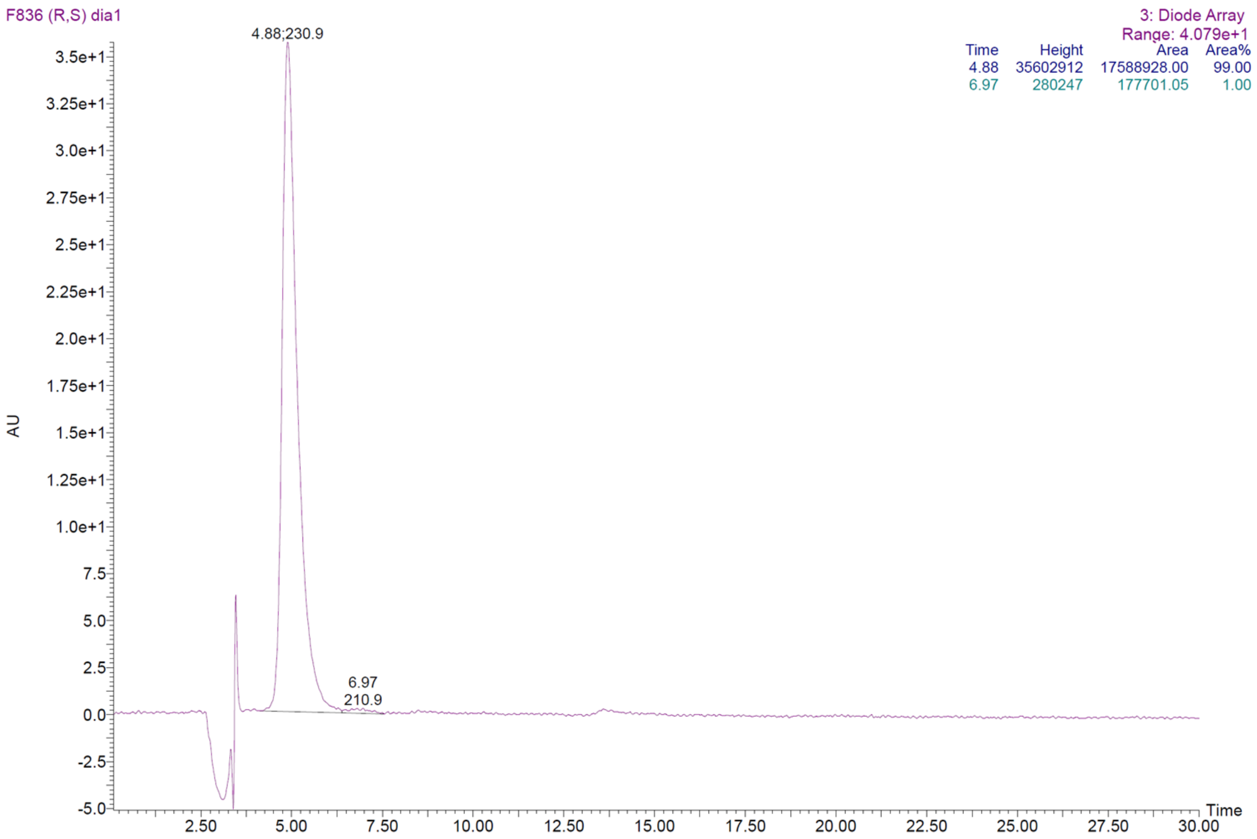


Chiral HPLC Chromatogram of compound C110RS.

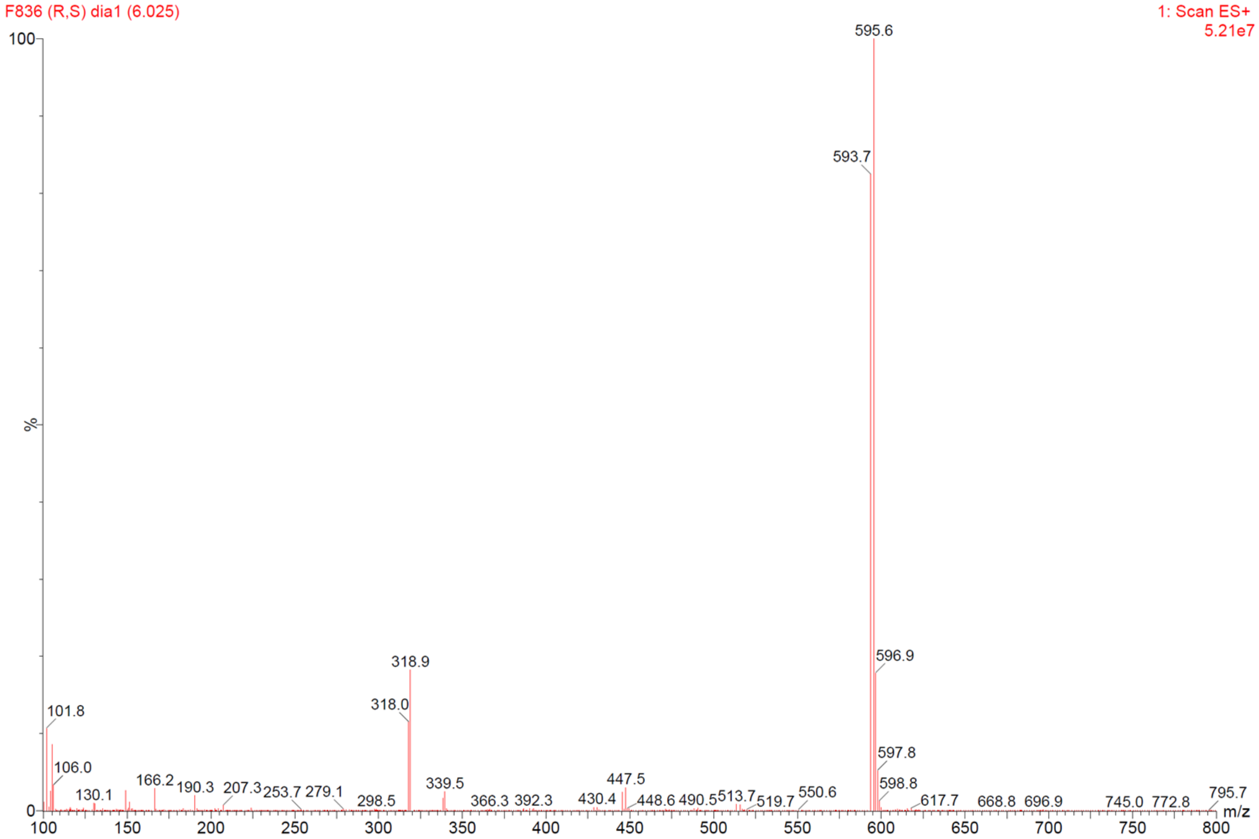


Mass spectrum of compound C110RS.

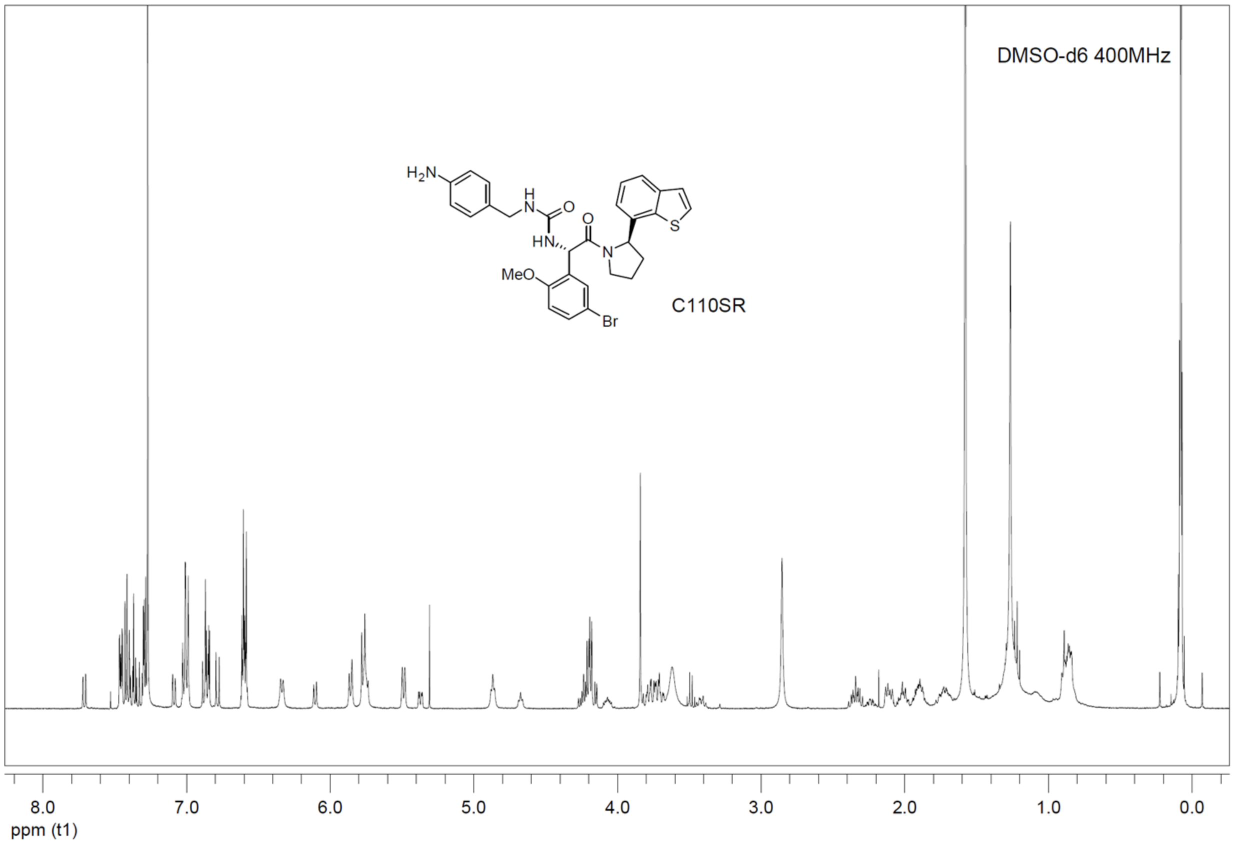


$^{1}$H NMR (400 MHz, DMSO-d6) spectrum of compound C110SR.

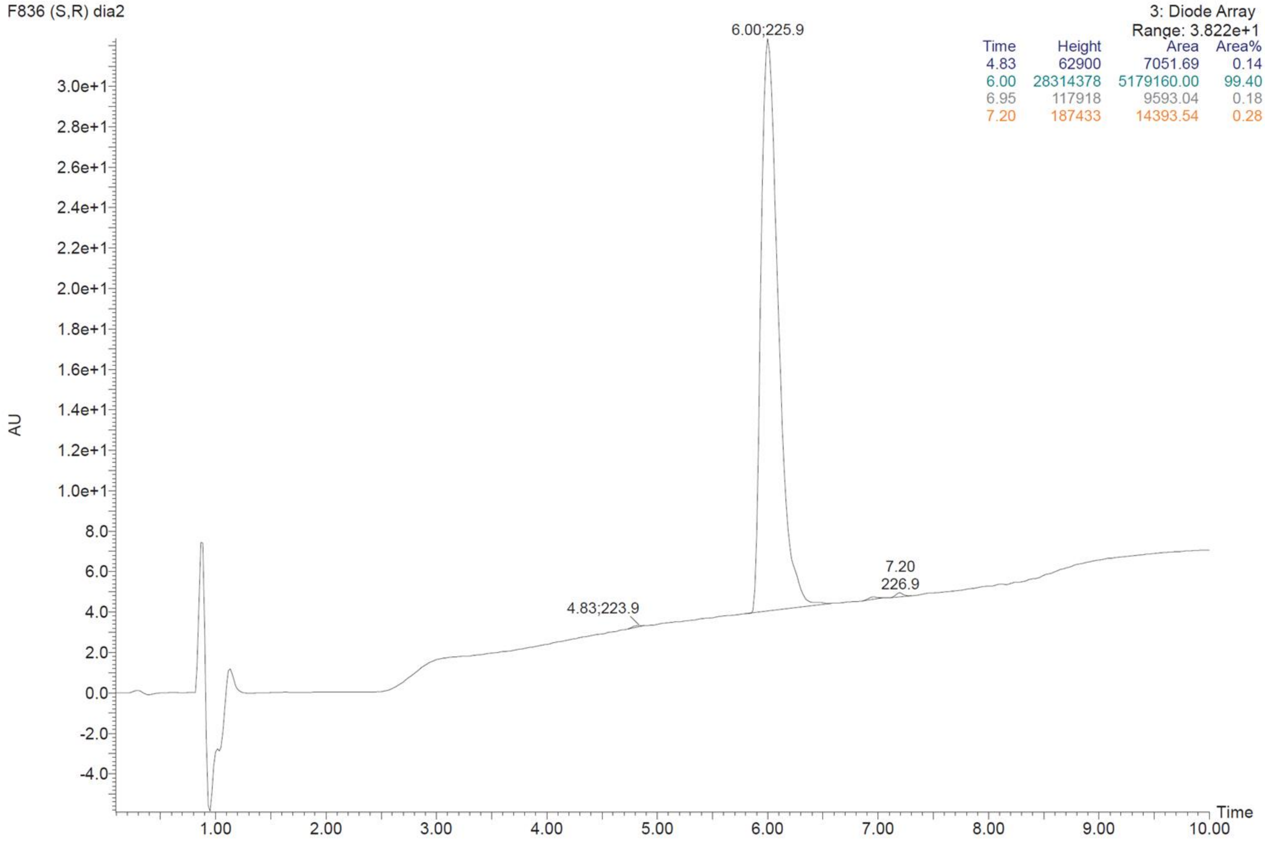


HPLC Chromatogram of compound C110SR.

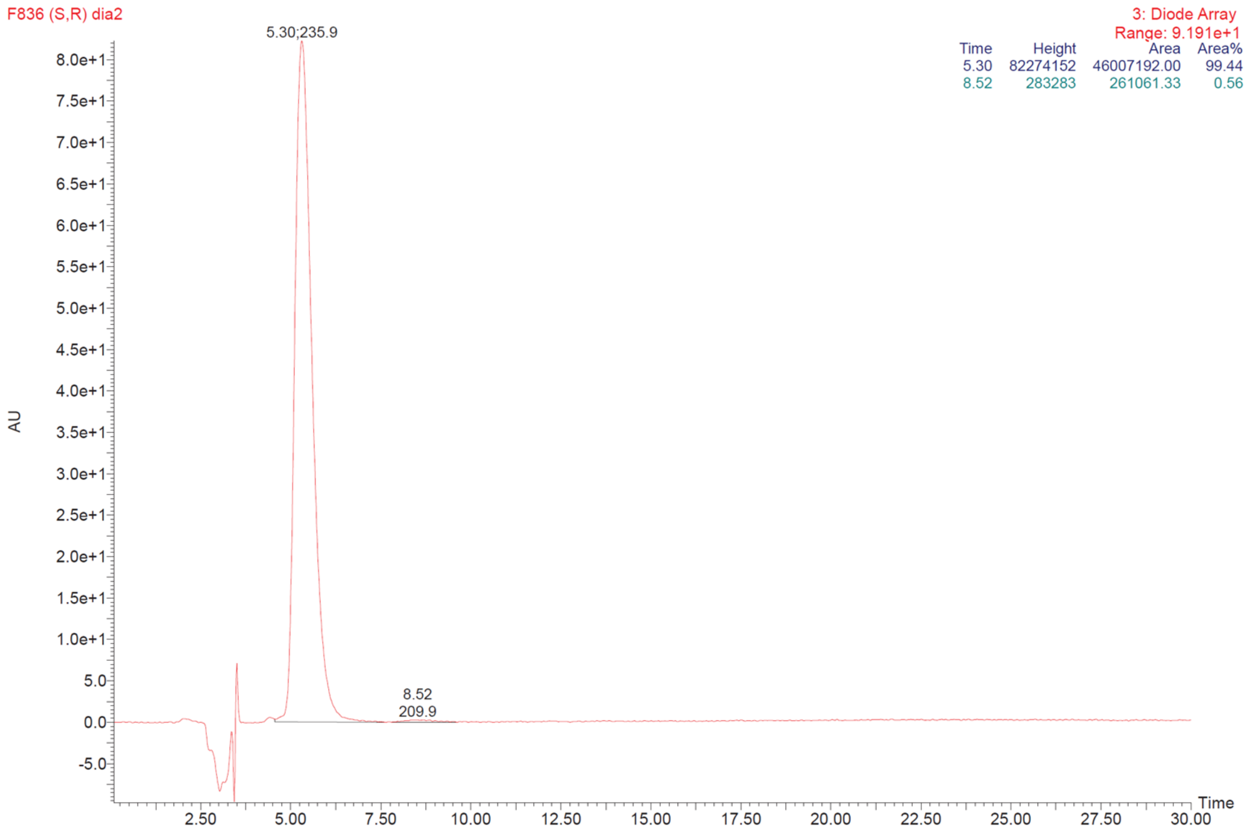


| Time | Height | Area | Area% |
|---|---|---|---|
| 5.30 | 82274152 | 46007192.00 | 99.44 |
| 8.52 | 283283 | 261061.33 | 0.56 |

Chiral HPLC Chromatogram of compound C110SR.

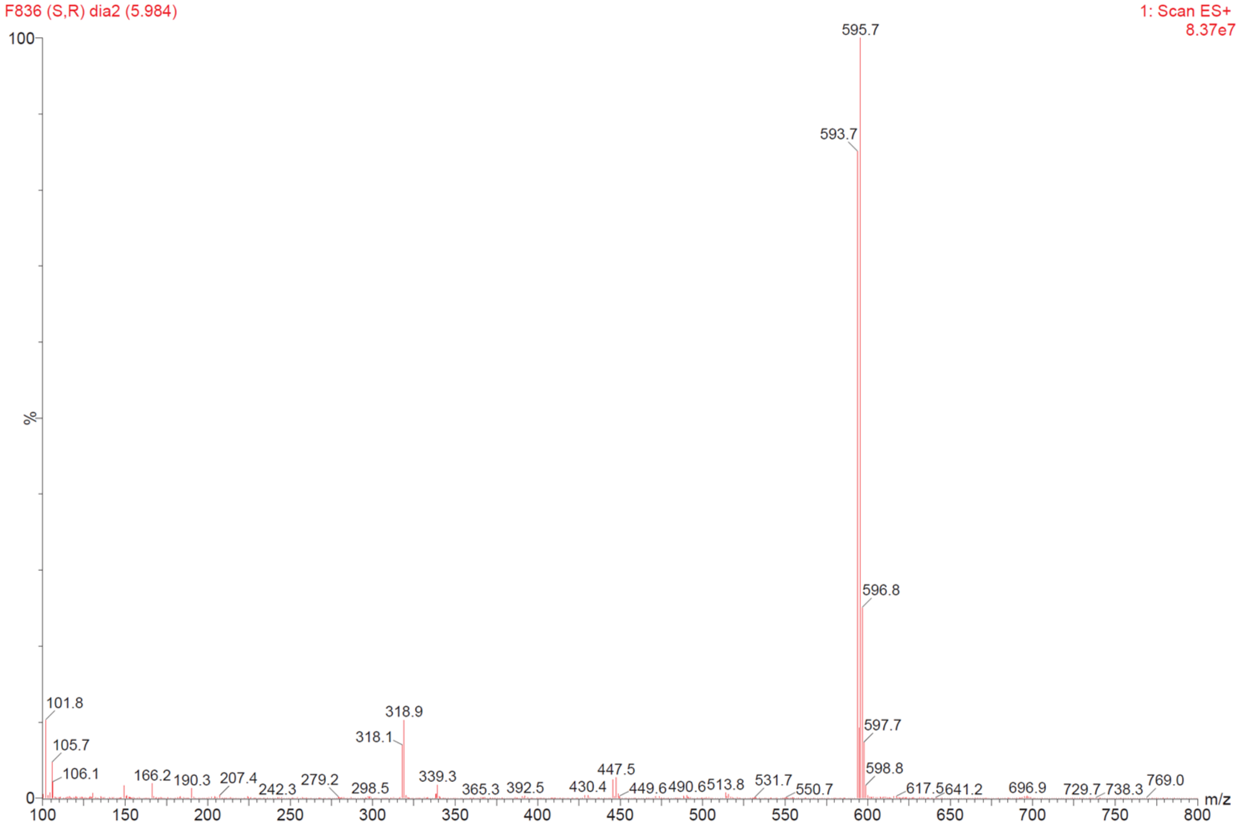


Mass spectrum of compound C110SR.

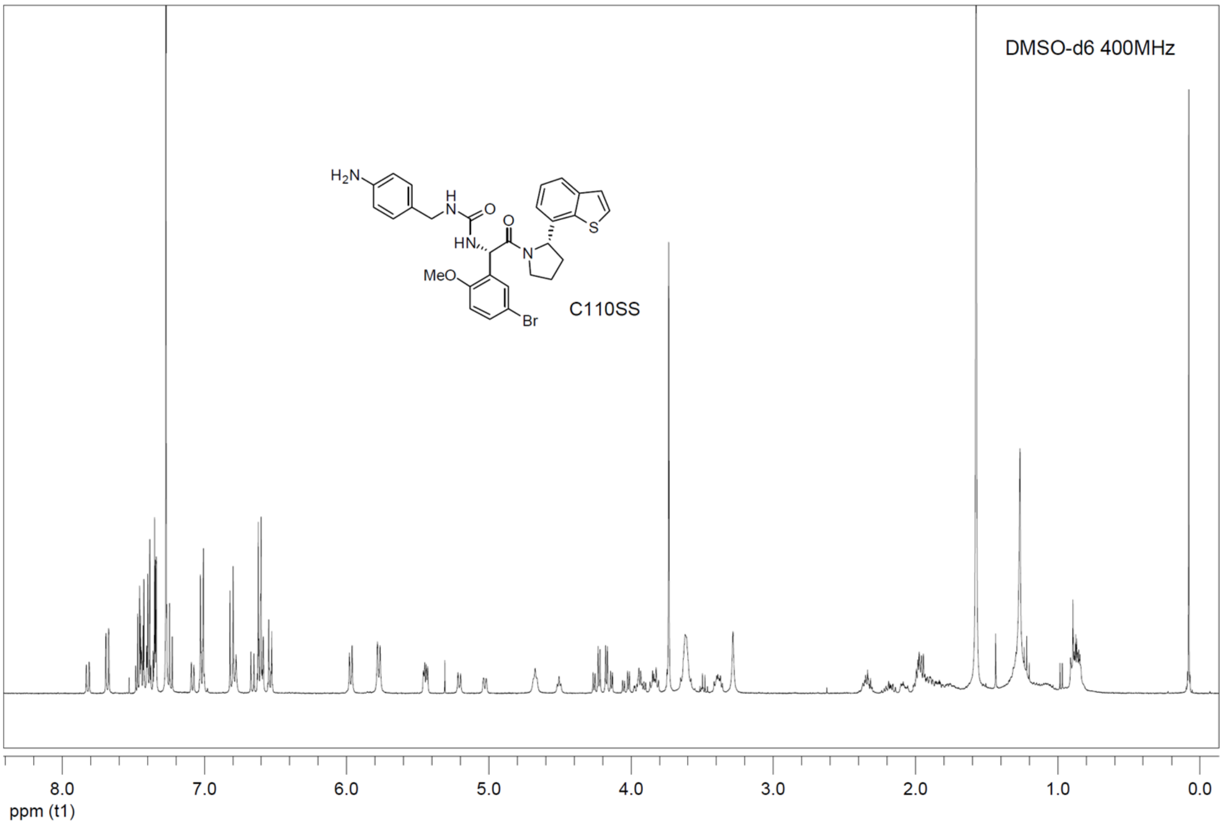


$^{1}$H NMR (400 MHz, DMSO-d6) spectrum of compound C110SS.

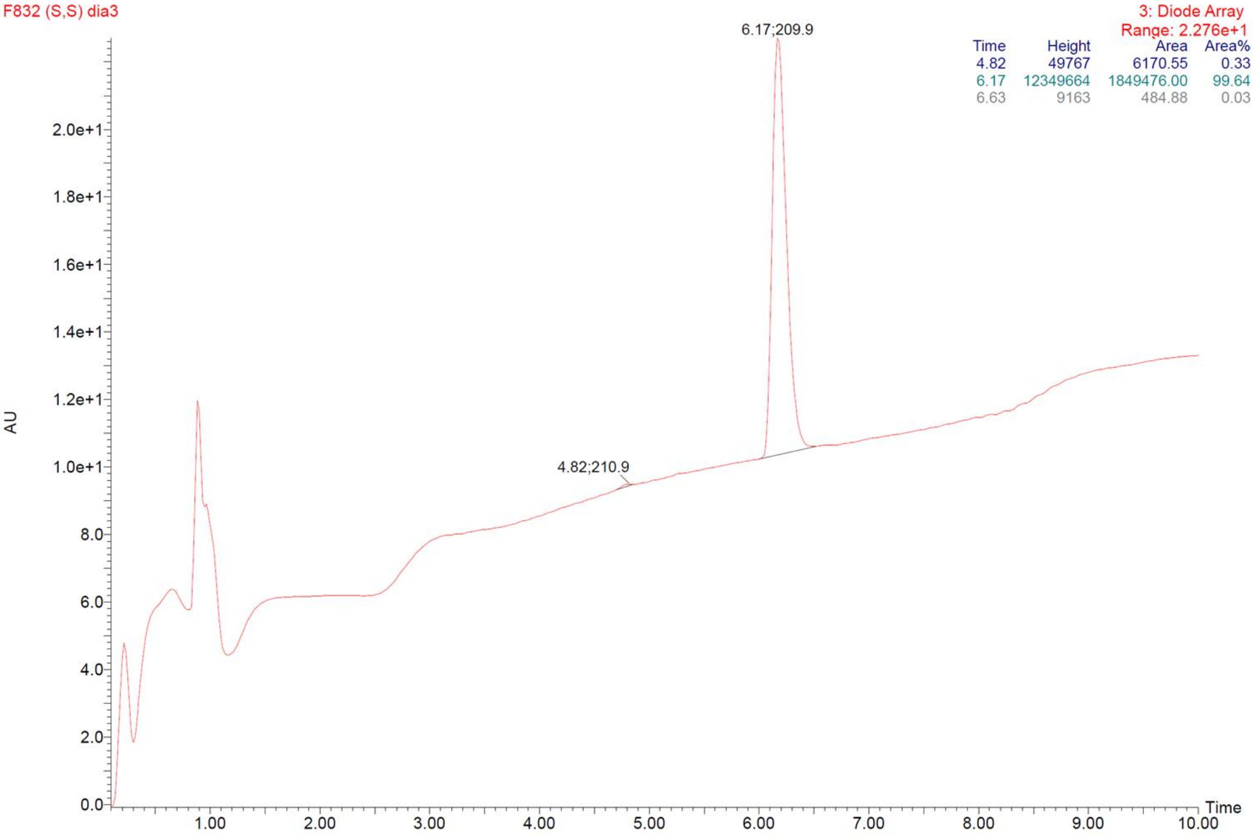


HPLC Chromatogram of compound C110SS.

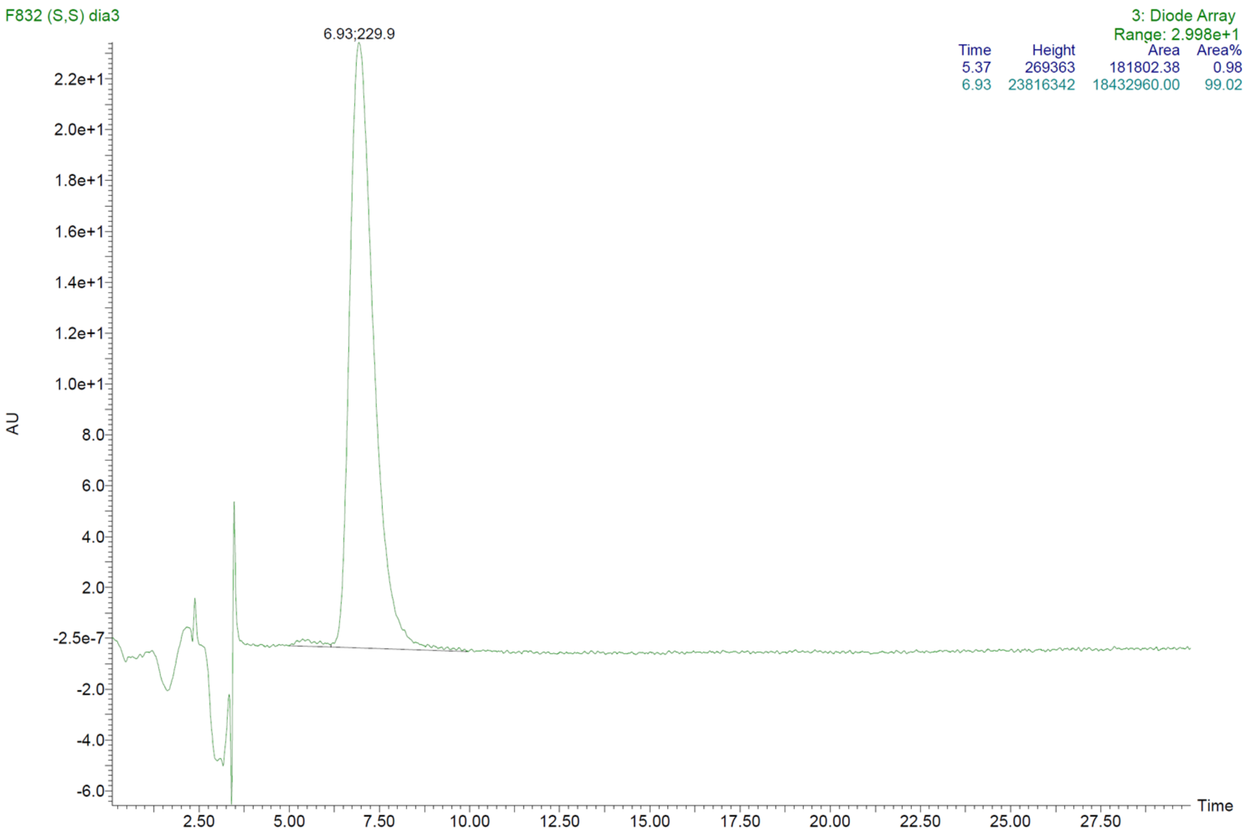


Chiral HPLC Chromatogram of compound C110SS.

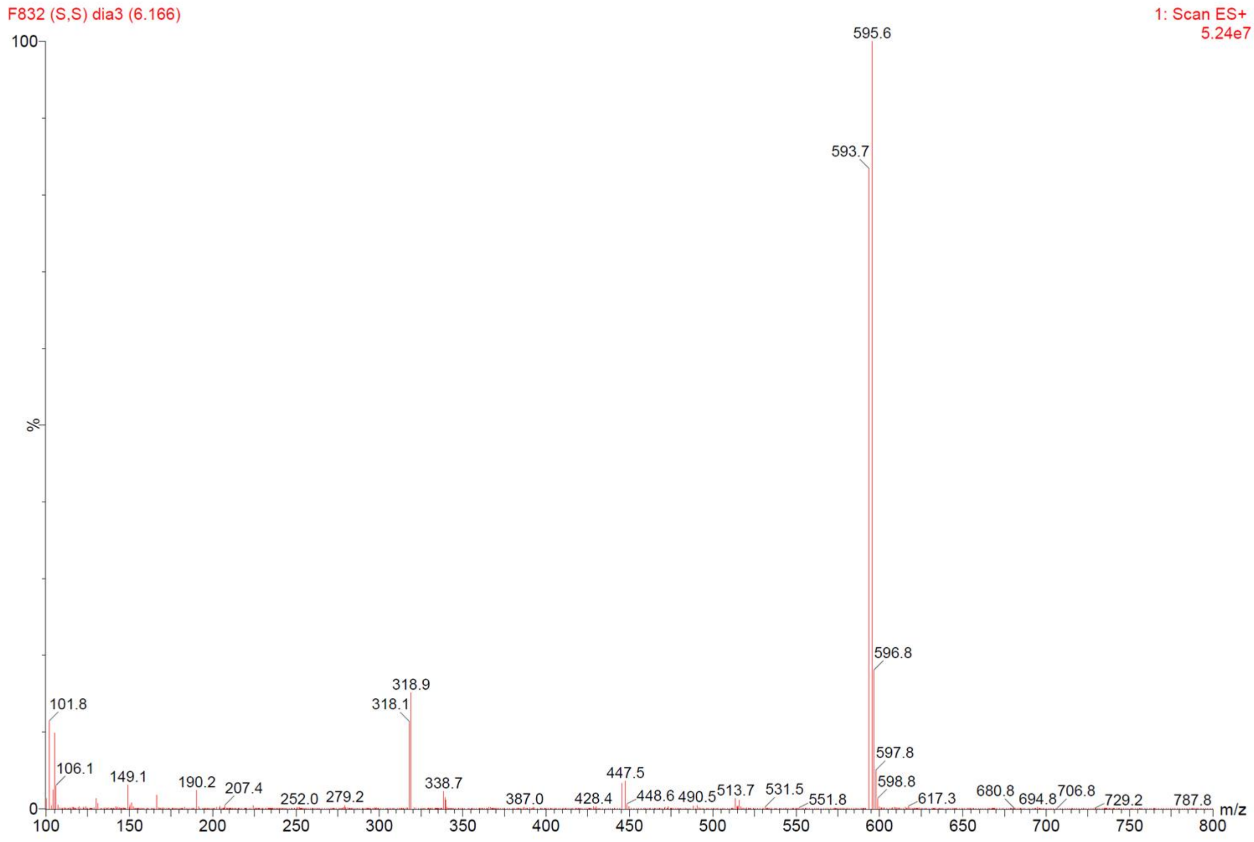


Mass spectrum of compound C110SS.

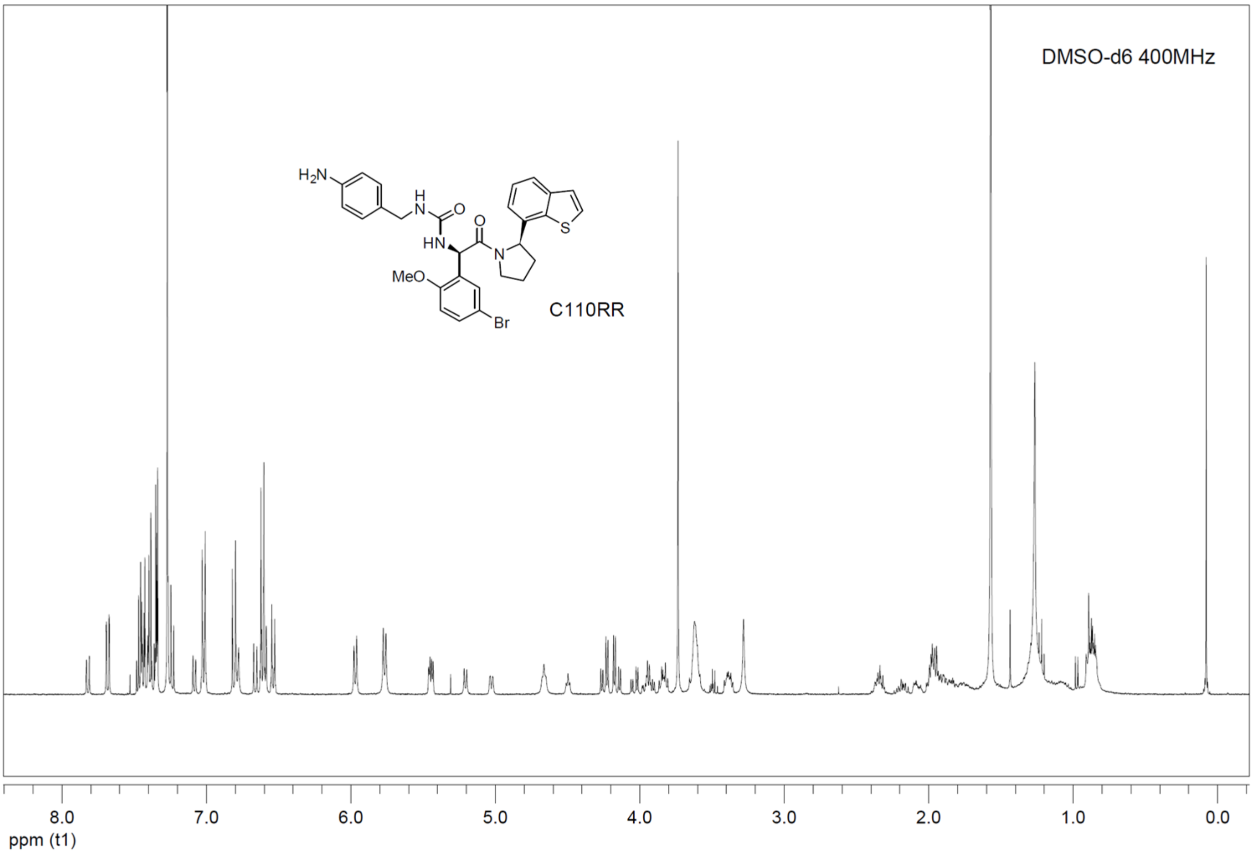


$^{1}H$ NMR (400 MHz, DMSO-d6) spectrum of compound C110RR.

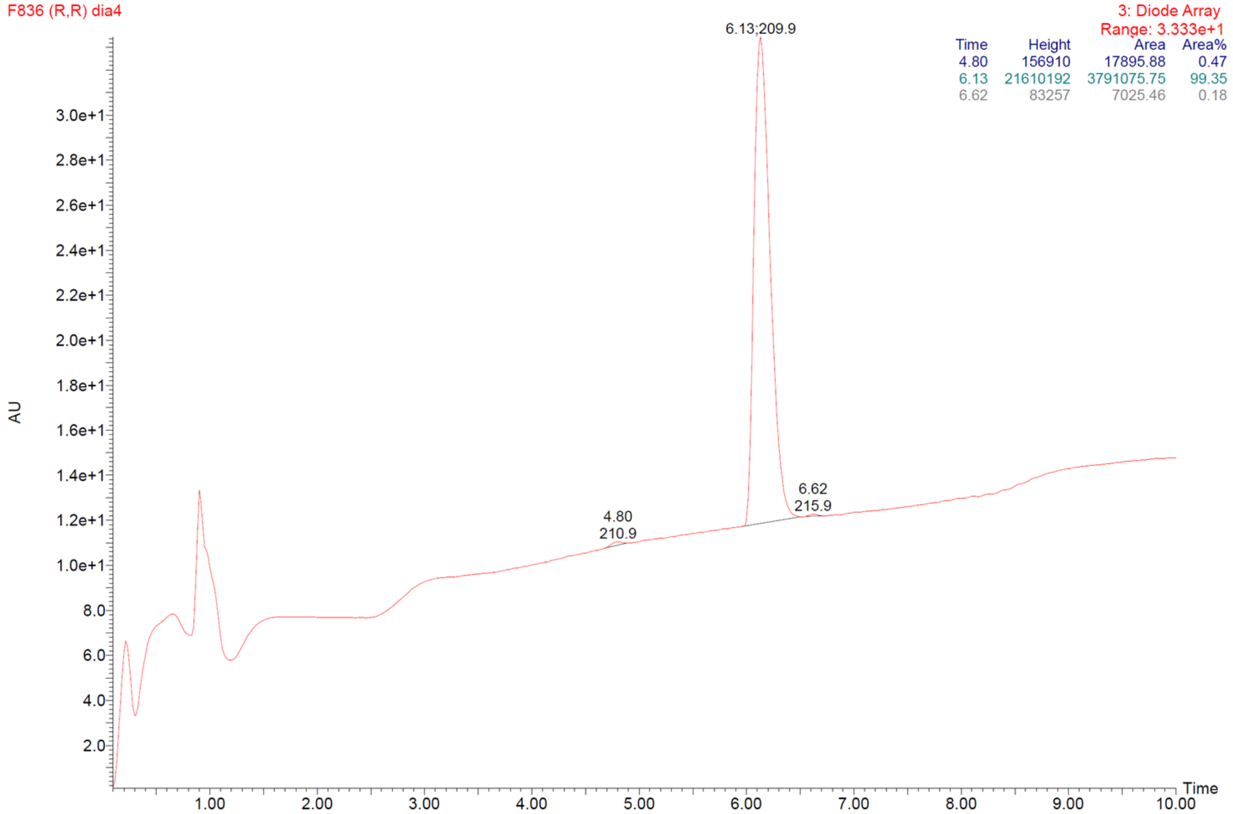


HPLC Chromatogram of compound C110RR.

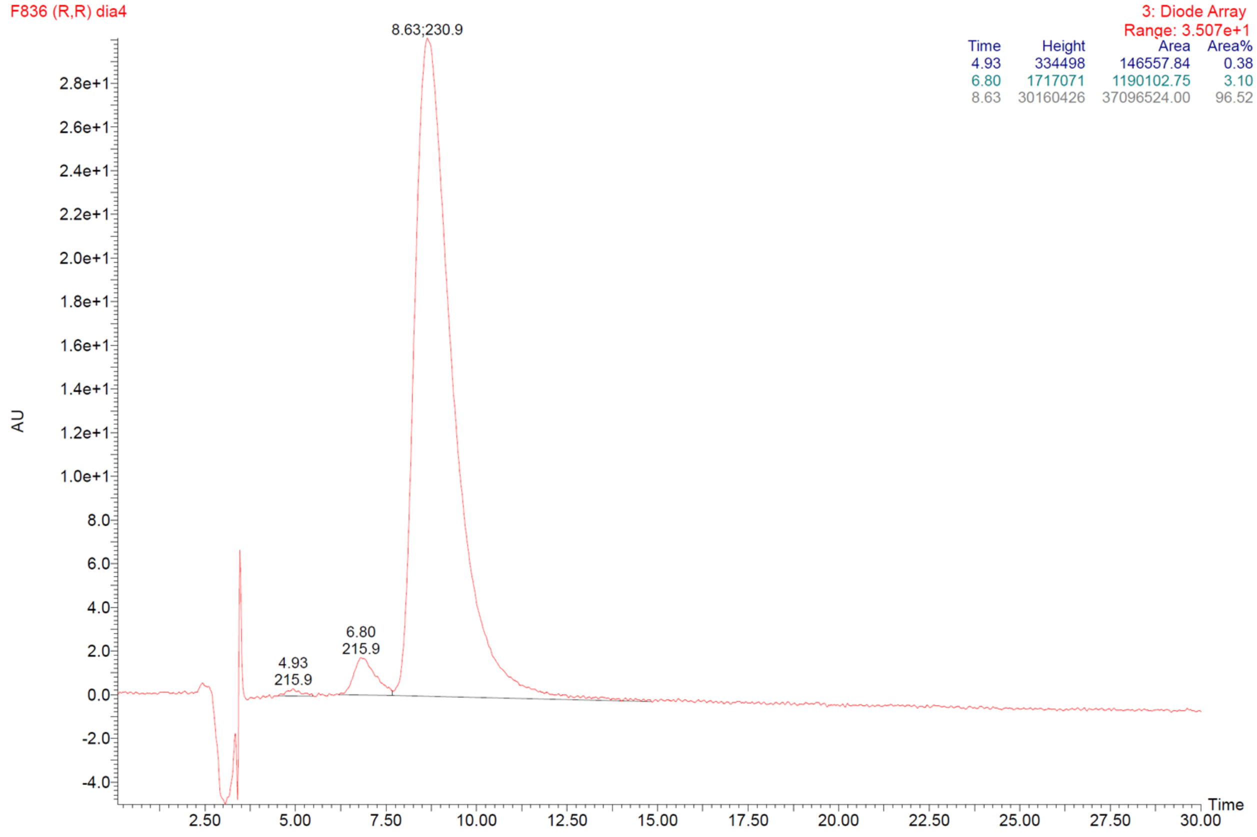


Chiral HPLC Chromatogram of compound C110RR.

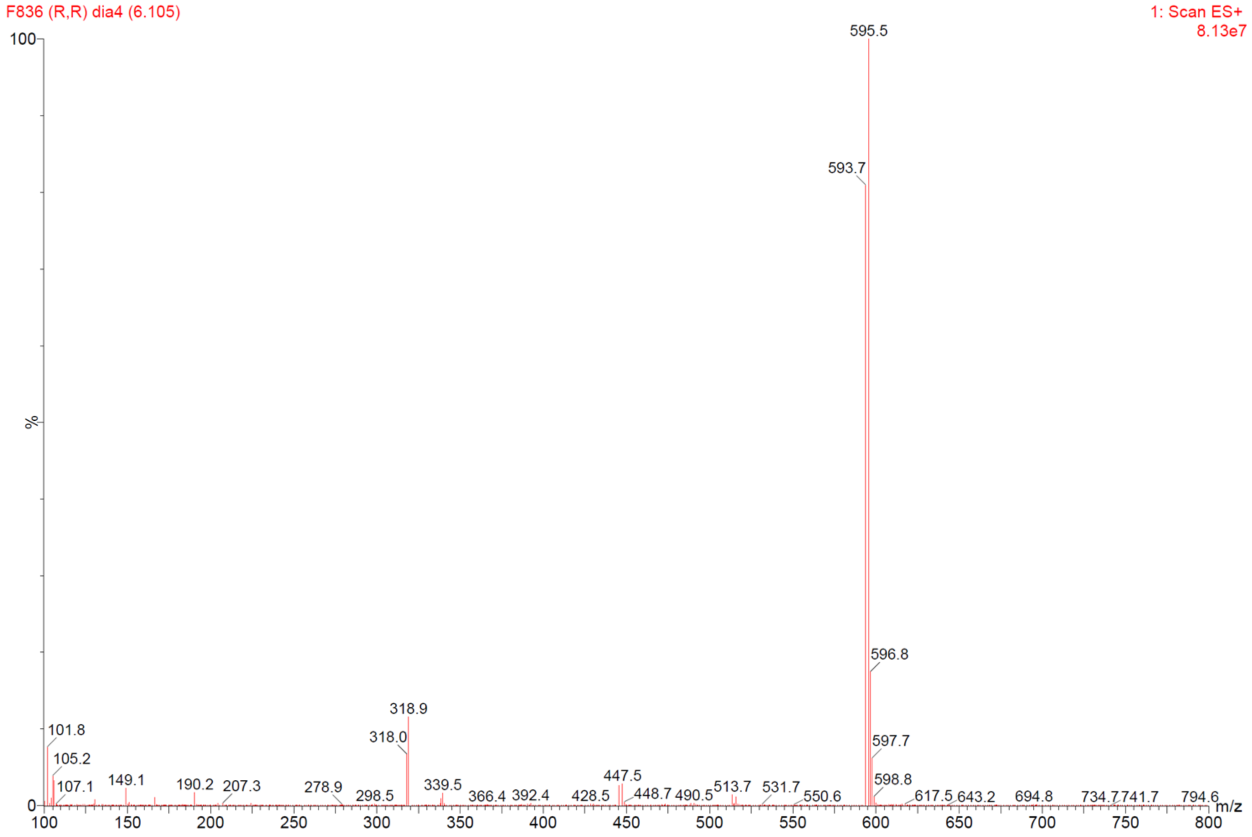


Mass spectrum of compound C110RR.

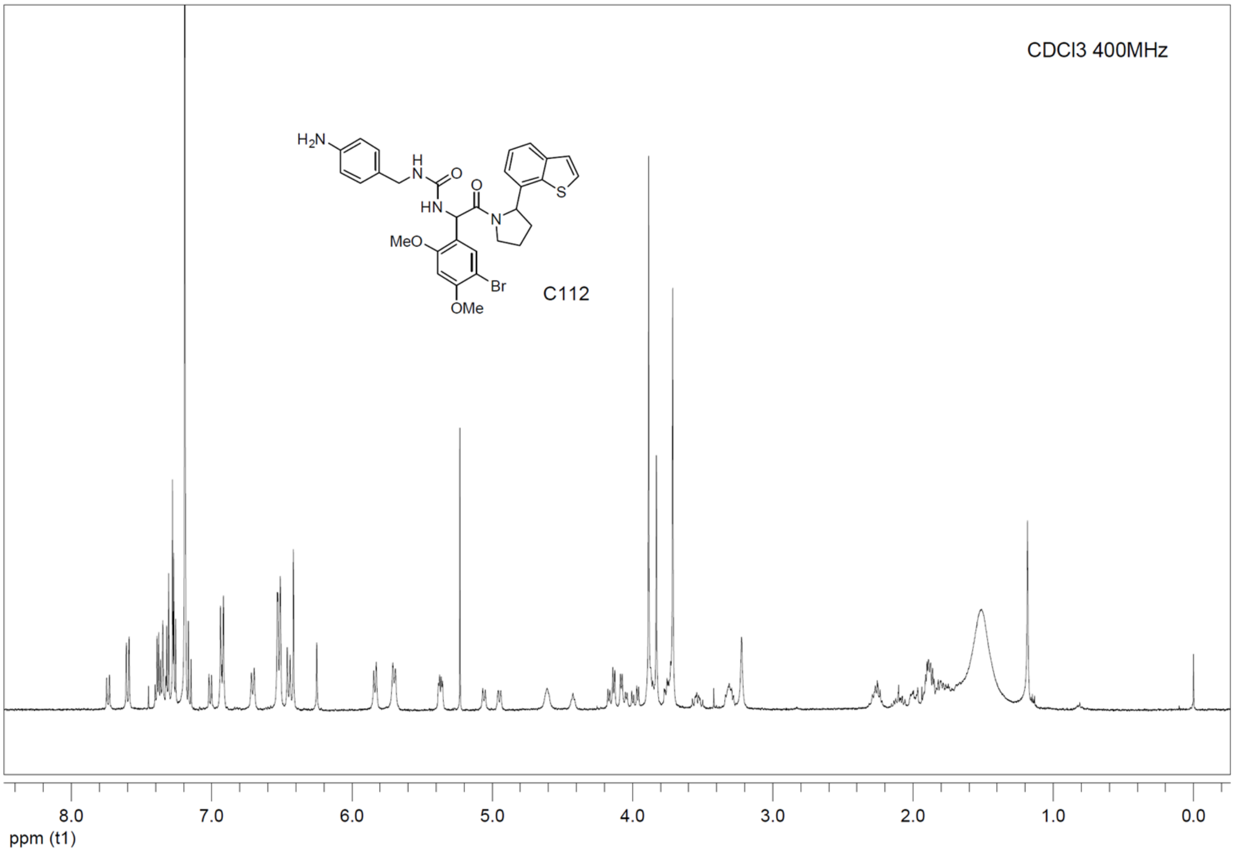


$^1$H NMR (400 MHz, $CDCl_3$) spectrum of compound C112.

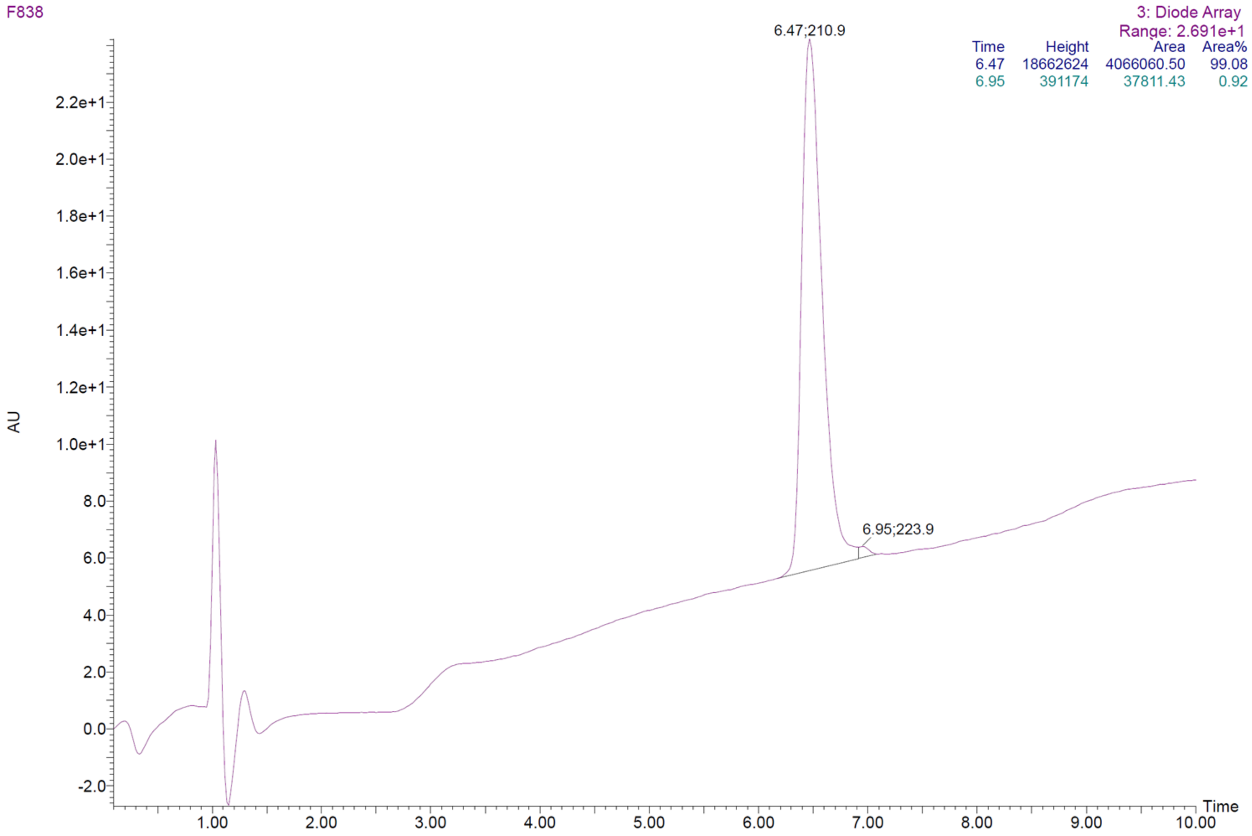


HPLC Chromatogram of compound C112.

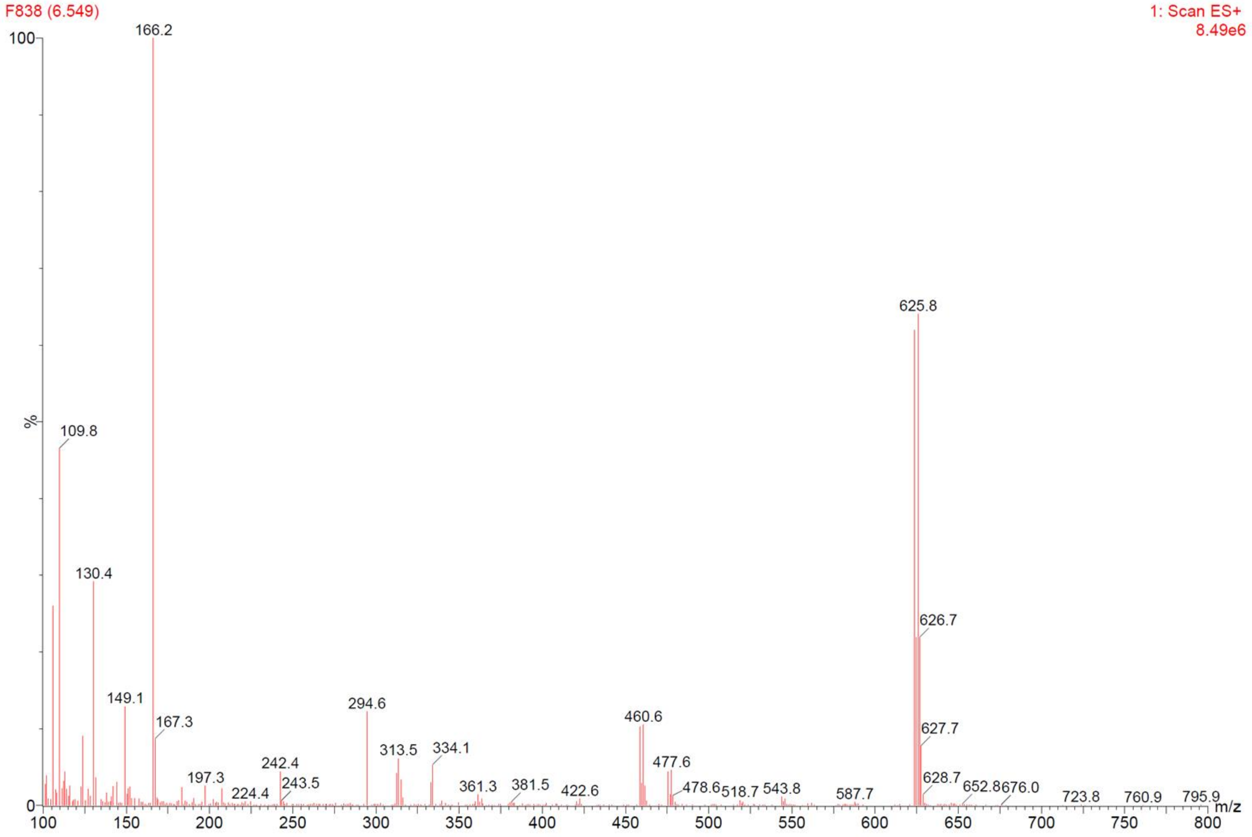


Mass spectrum of compound C112.

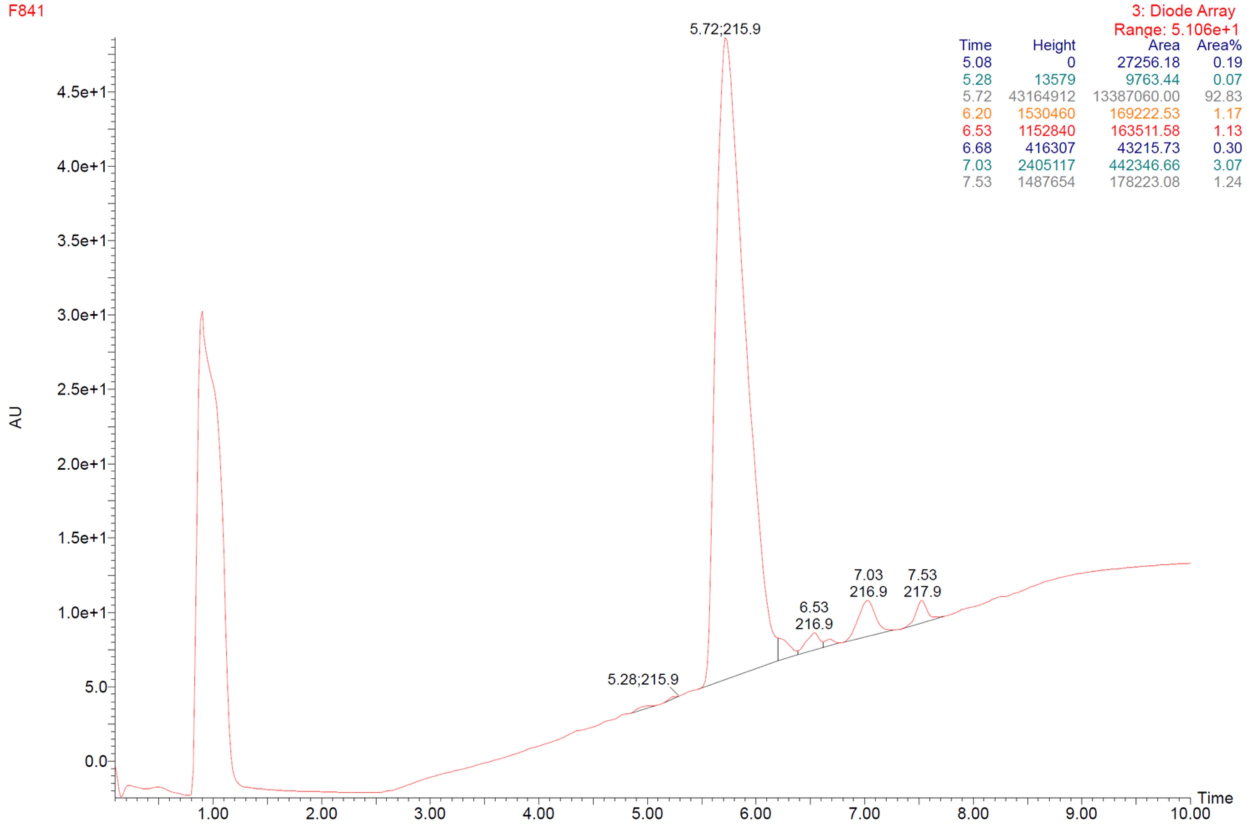


HPLC Chromatogram of compound C115.

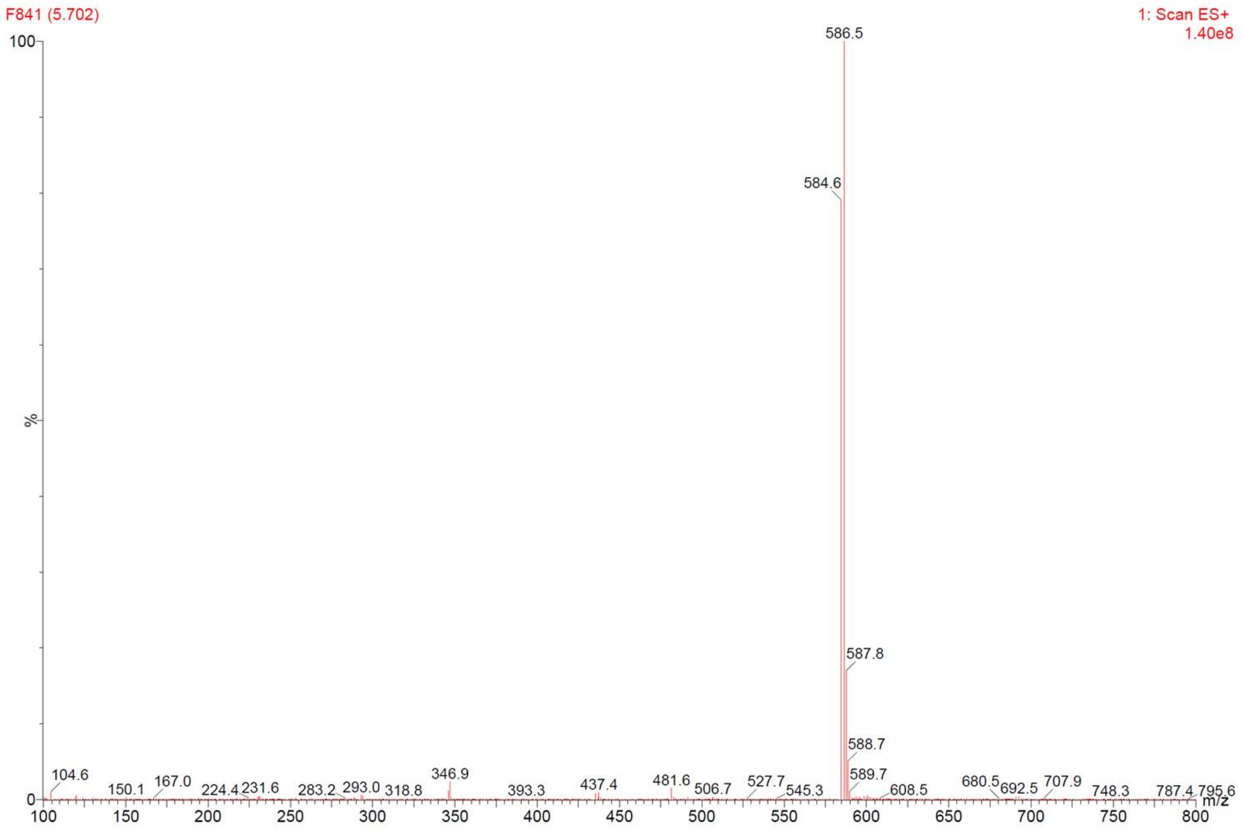


Mass spectrum of compound C115.

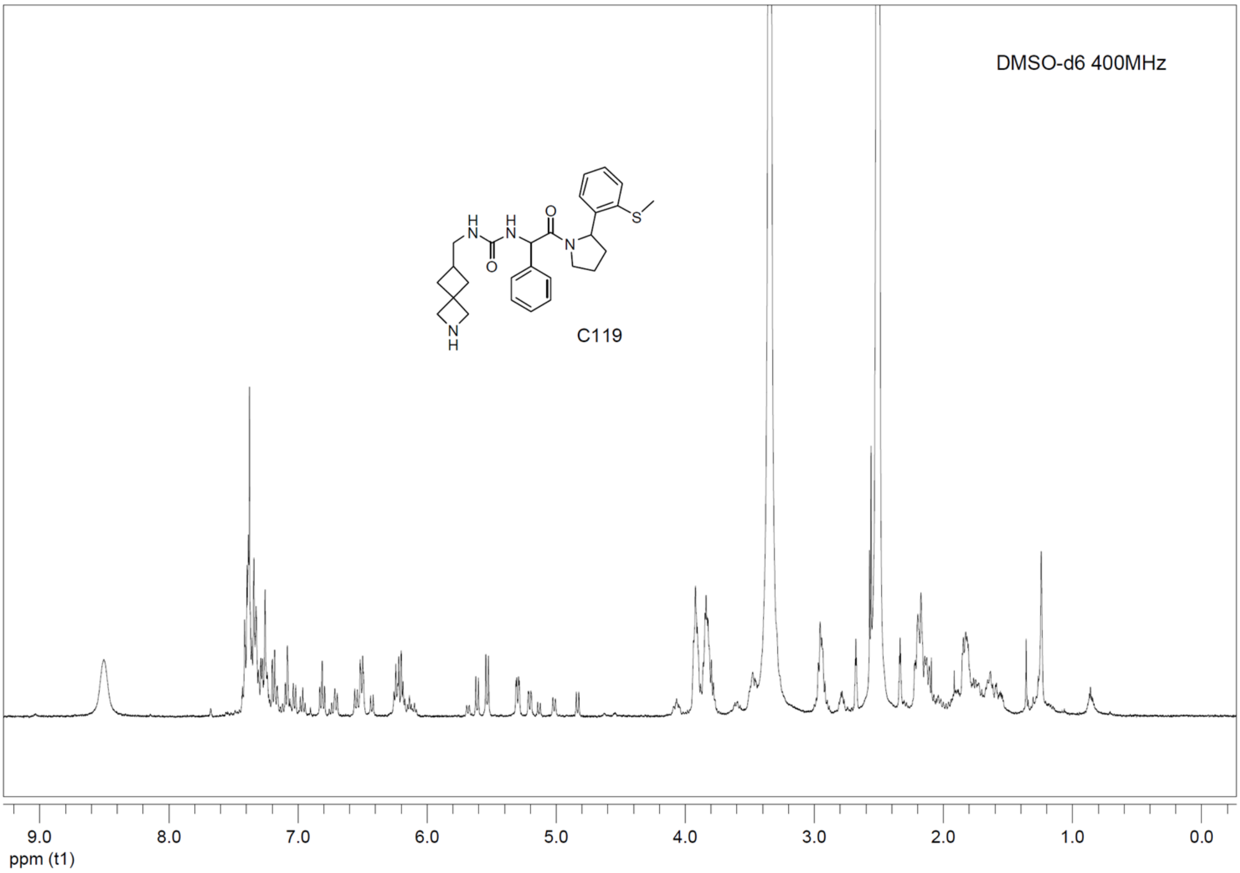


$^{1}$H NMR (400 MHz, DMSO-d6) spectrum of compound C119.

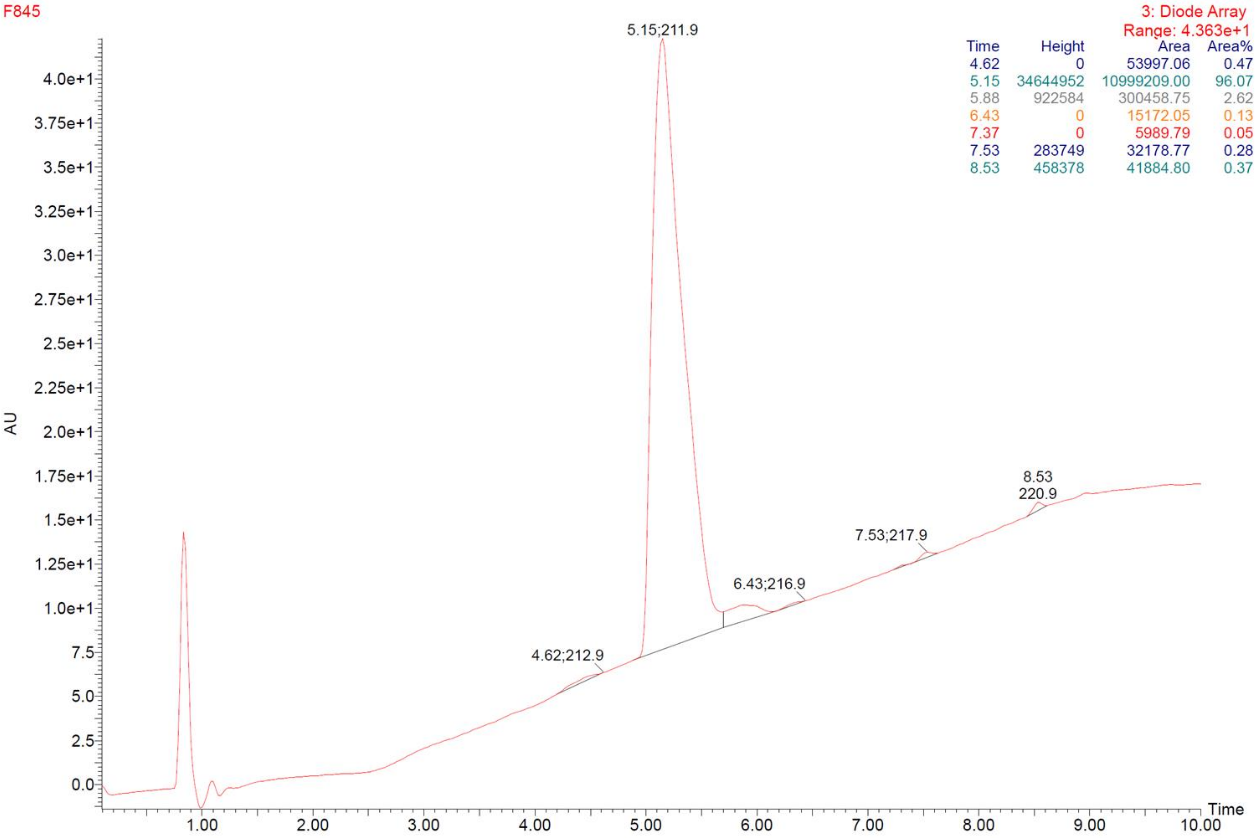


HPLC Chromatogram of compound C119.

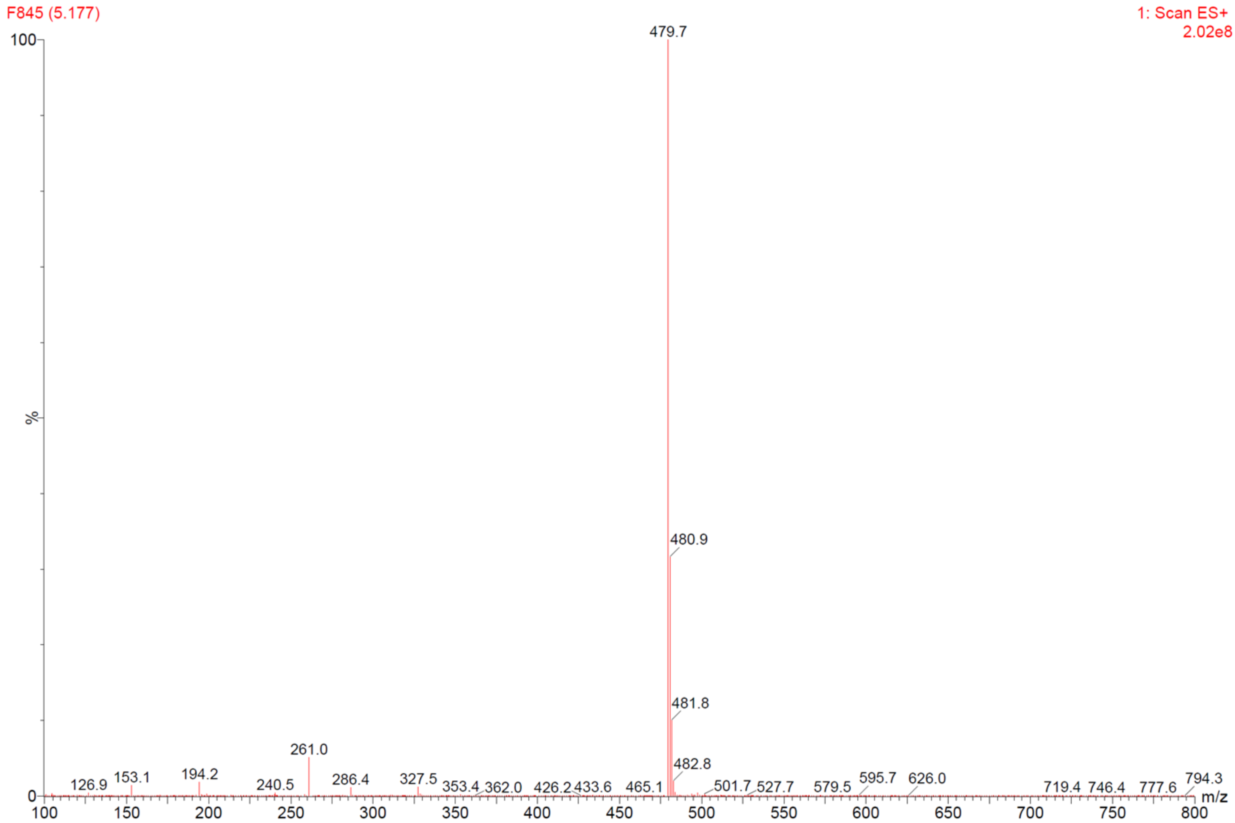


Mass spectrum of compound C119.

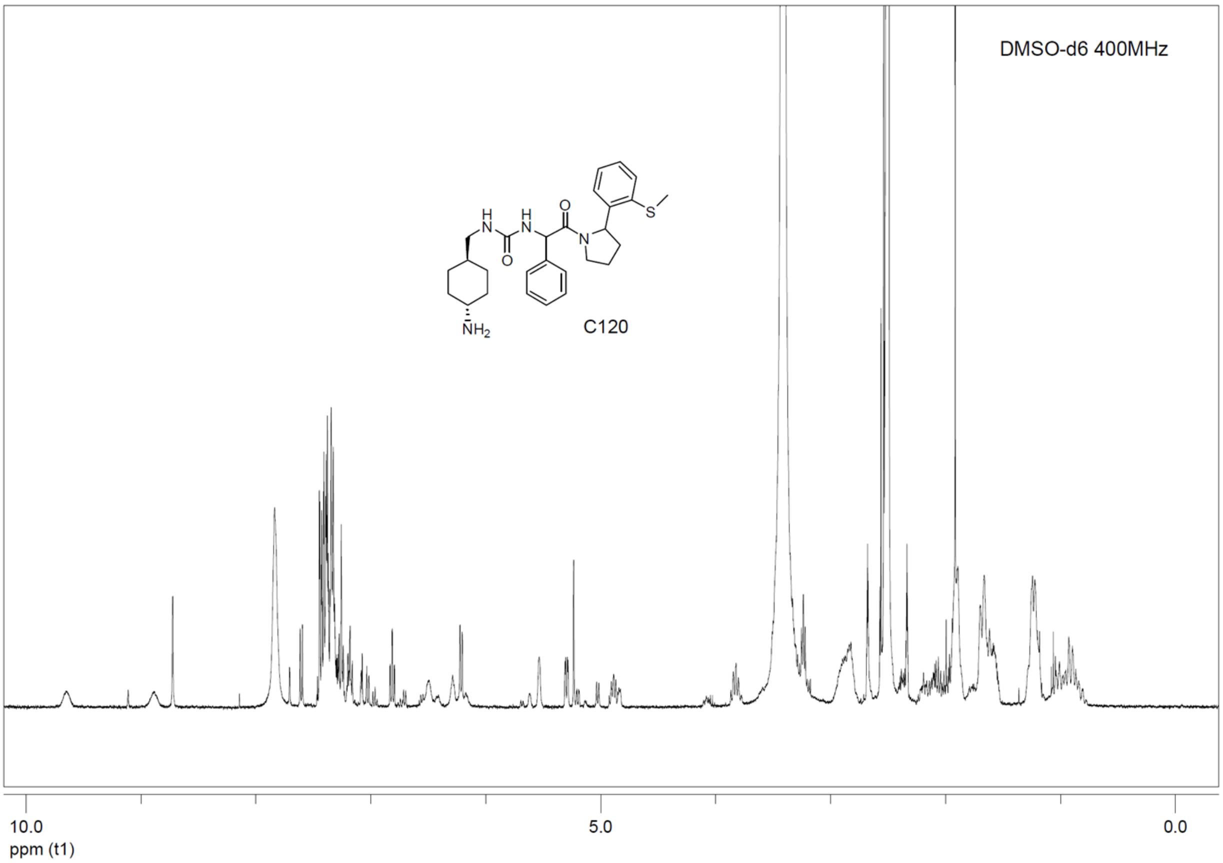


$^{1}$H NMR (400 MHz, DMSO-d6) spectrum of compound C120.

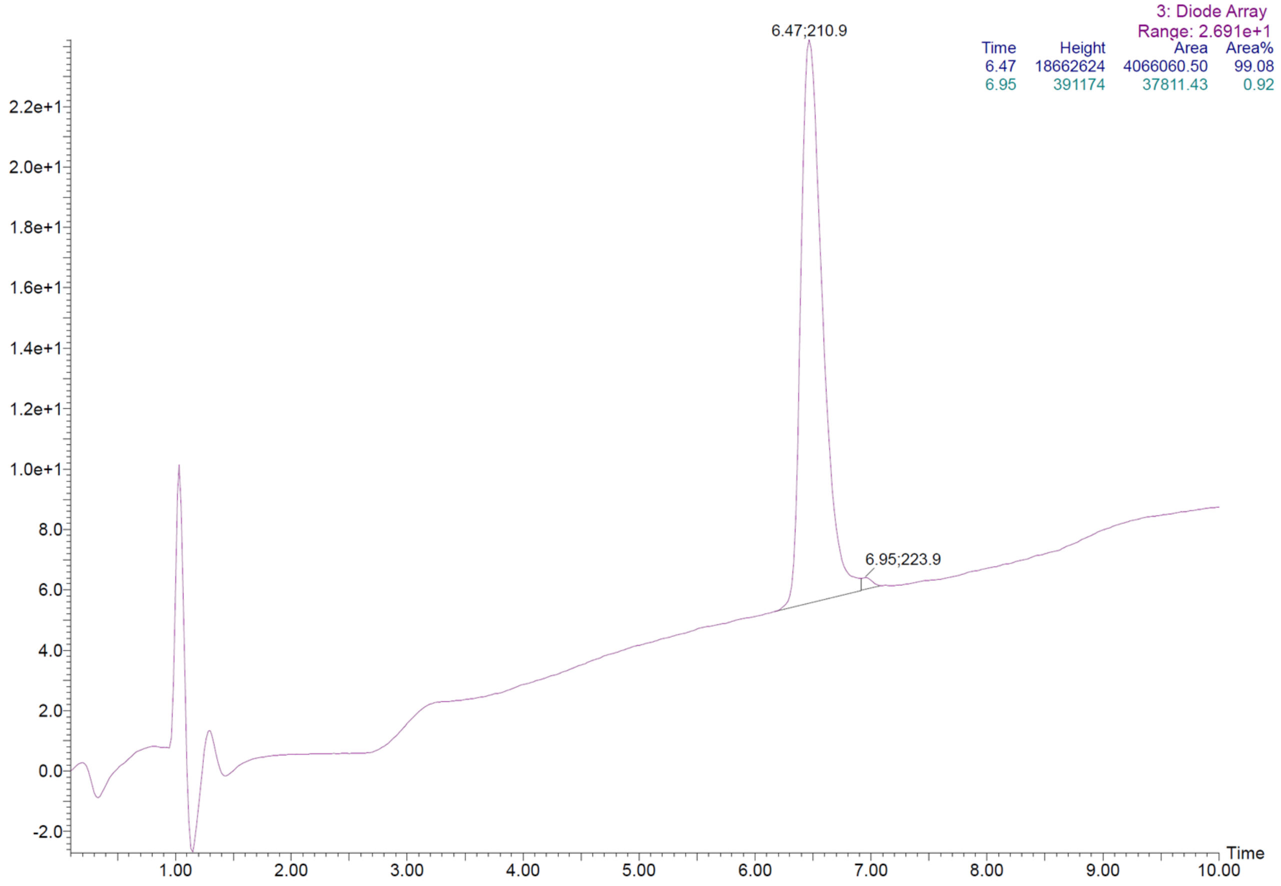


HPLC Chromatogram of compound C120.

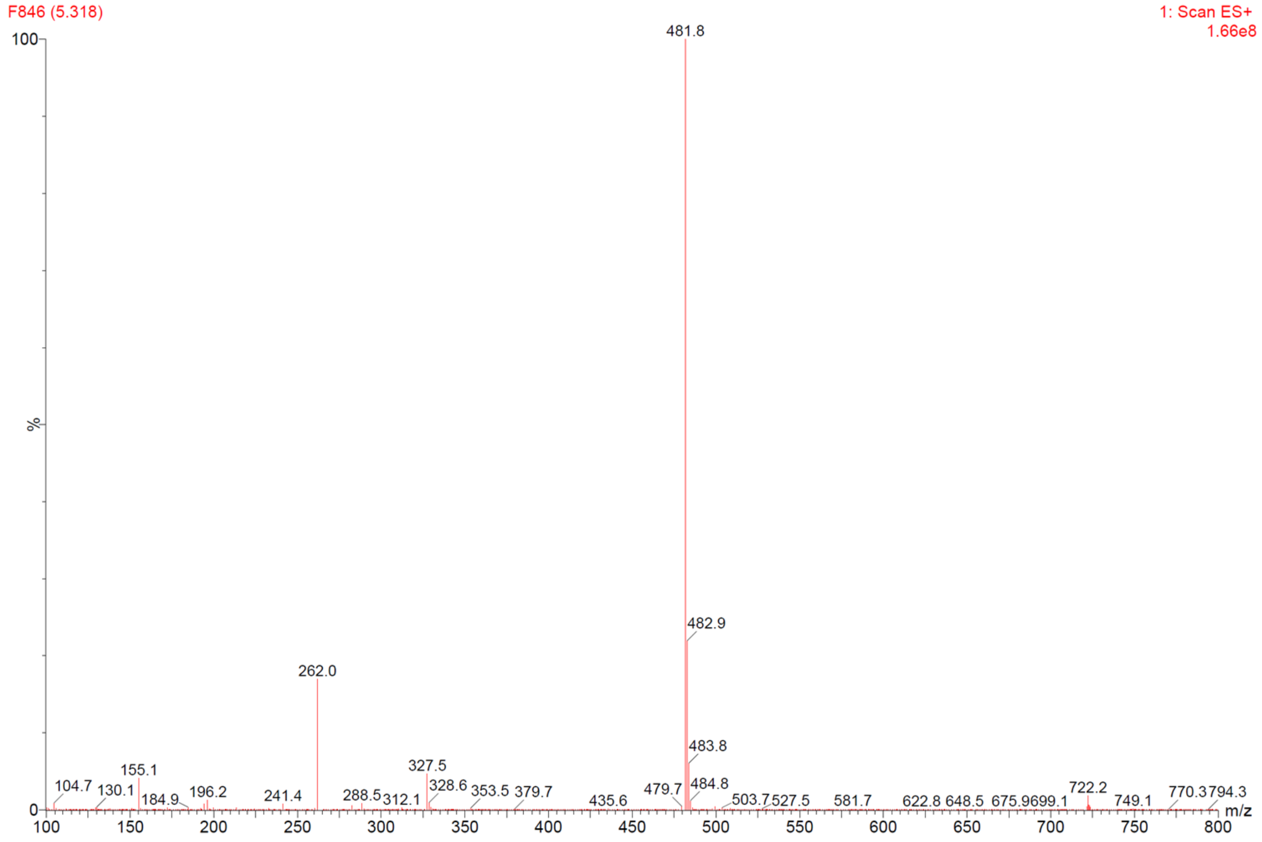


Mass spectrum of compound C120.

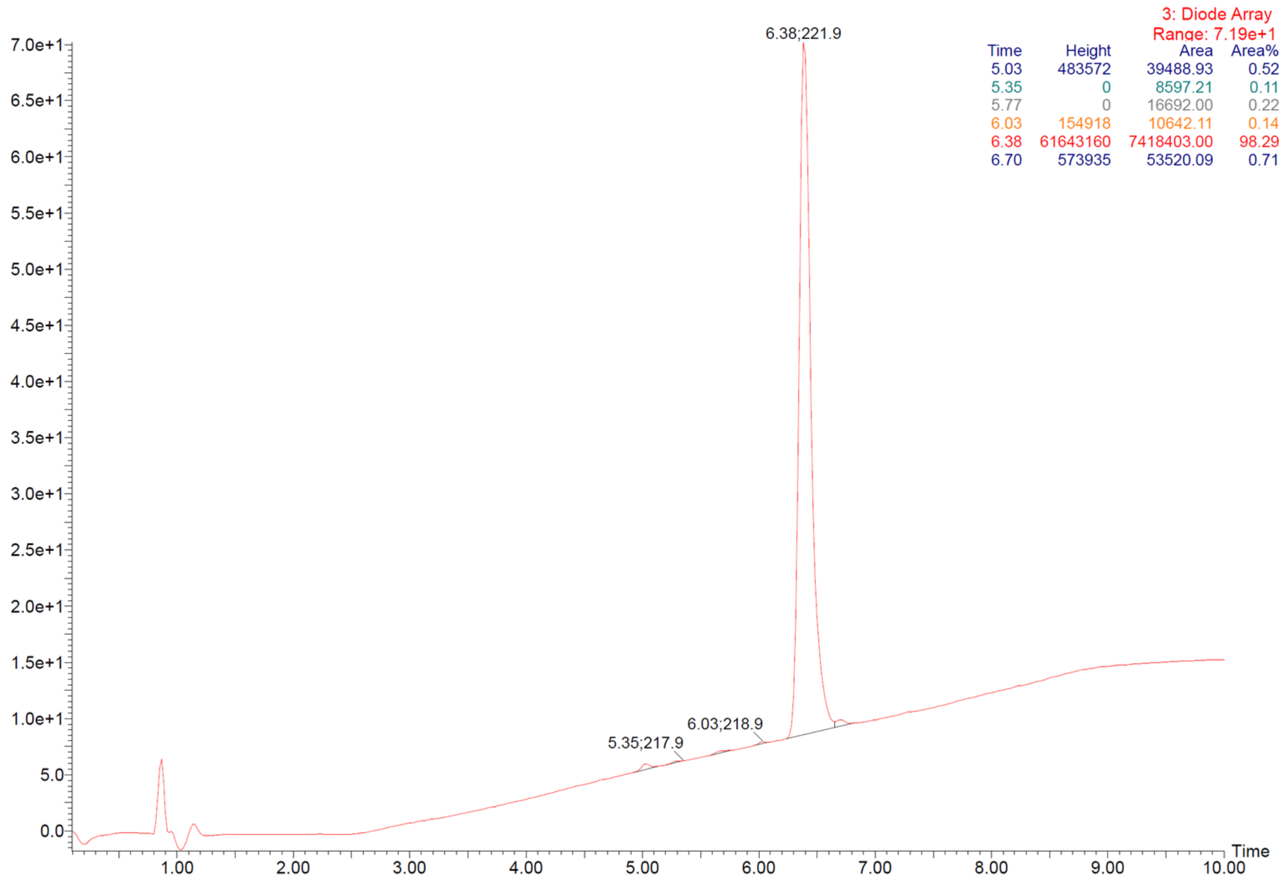


| Time | Height | Area | Area% |
|---|---|---|---|
| 5.03 | 483572 | 39488.93 | 0.52 |
| 5.35 | 0 | 8597.21 | 0.11 |
| 5.77 | 0 | 16692.00 | 0.22 |
| 6.03 | 154918 | 10642.11 | 0.14 |
| 6.38 | 61643160 | 7418403.00 | 98.29 |
| 6.70 | 573935 | 53520.09 | 0.71 |

HPLC Chromatogram of compound C123.

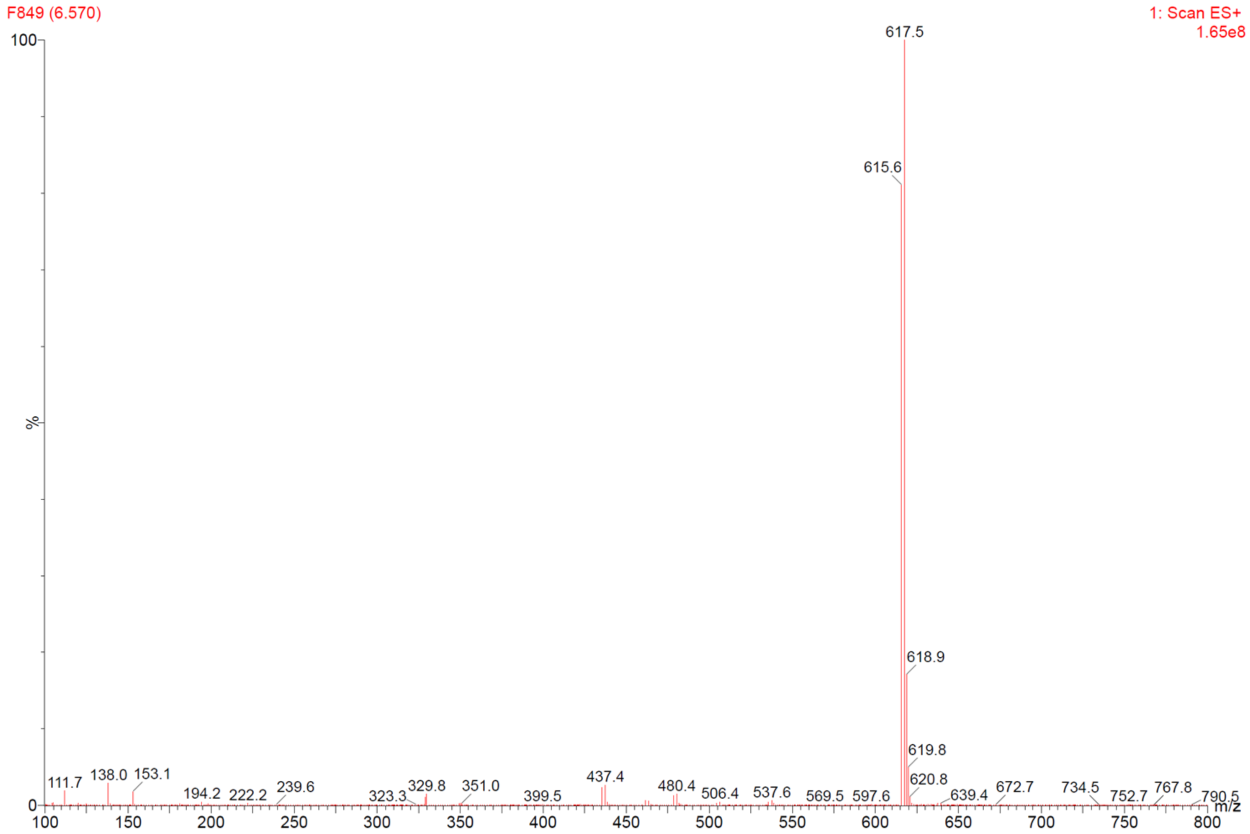


Mass spectrum of compound C123.

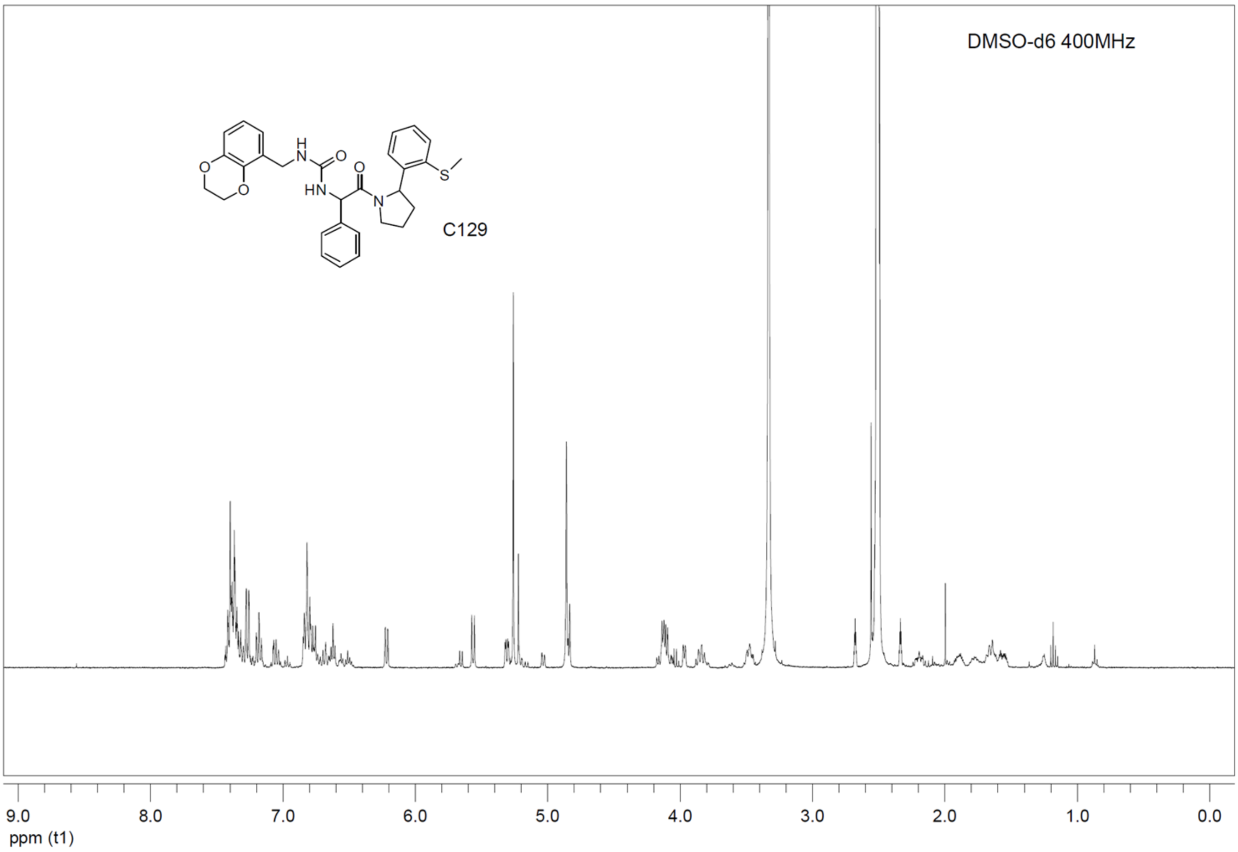


$^{1}$H NMR (400 MHz, DMSO-d6) spectrum of compound C129.

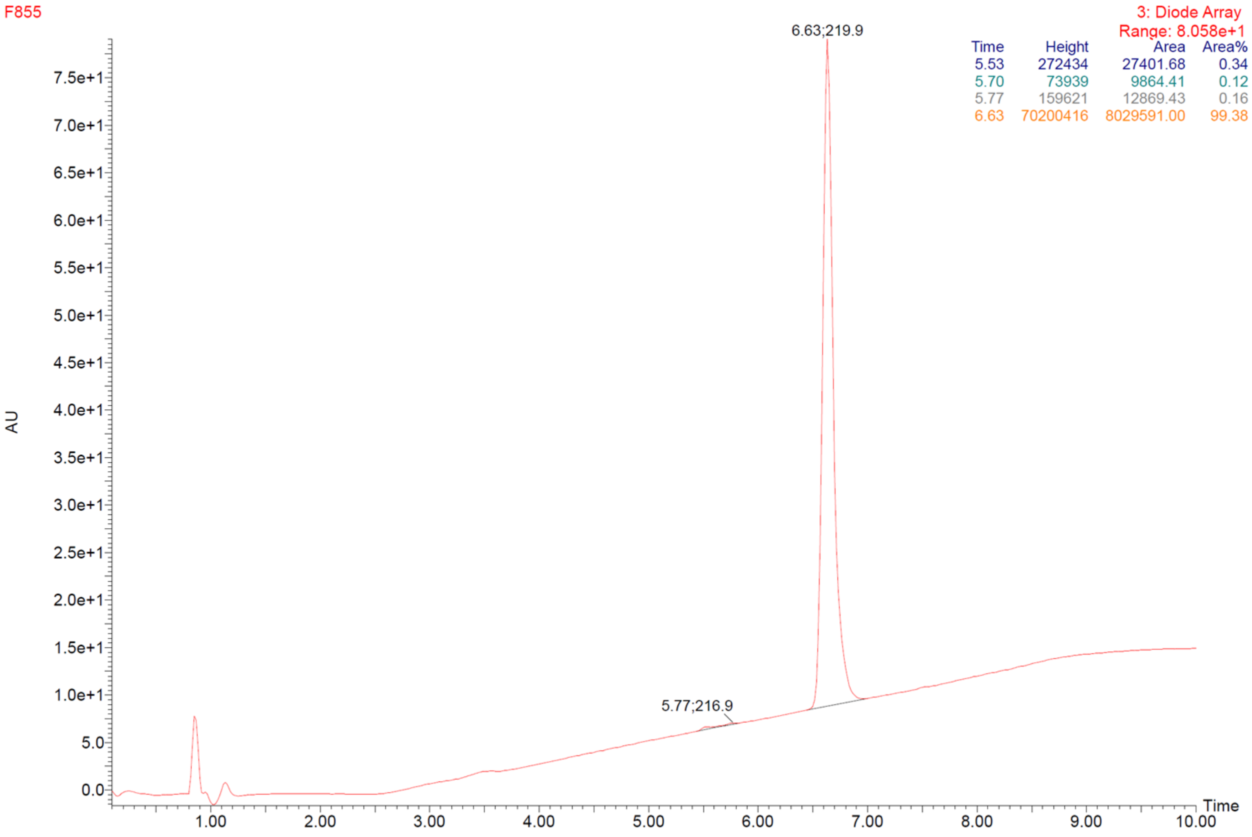


HPLC Chromatogram of compound C129.

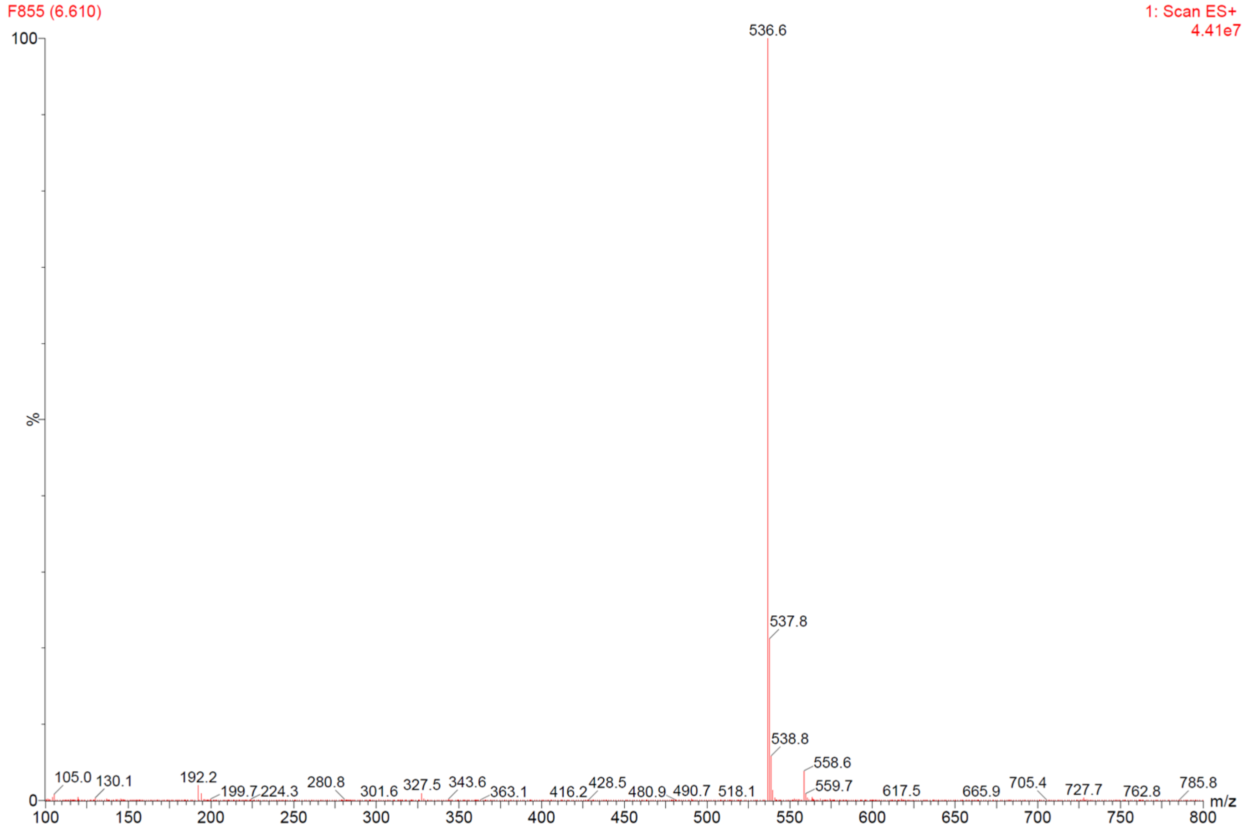


Mass spectrum of compound C129.

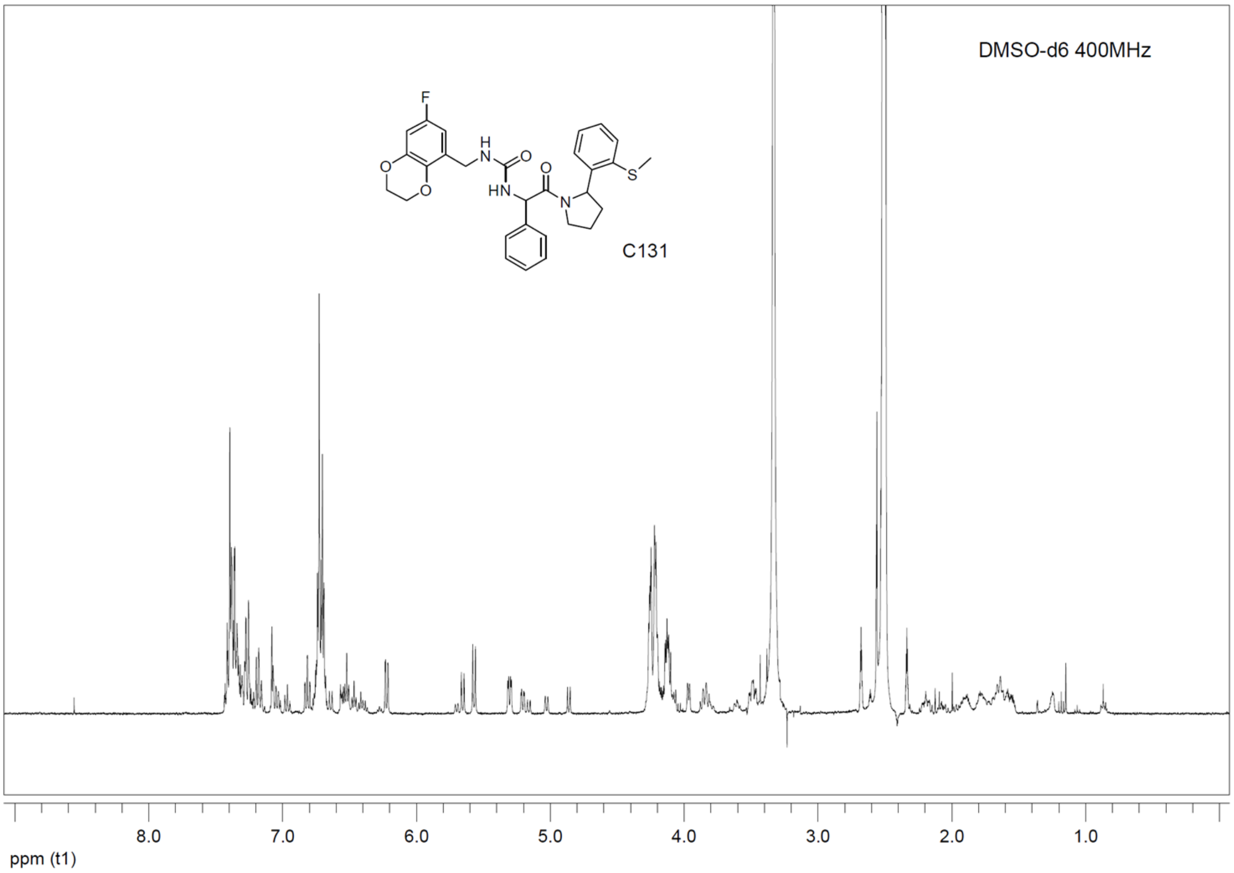


$^{1}$H NMR (400 MHz, DMSO-d6) spectrum of compound C131.

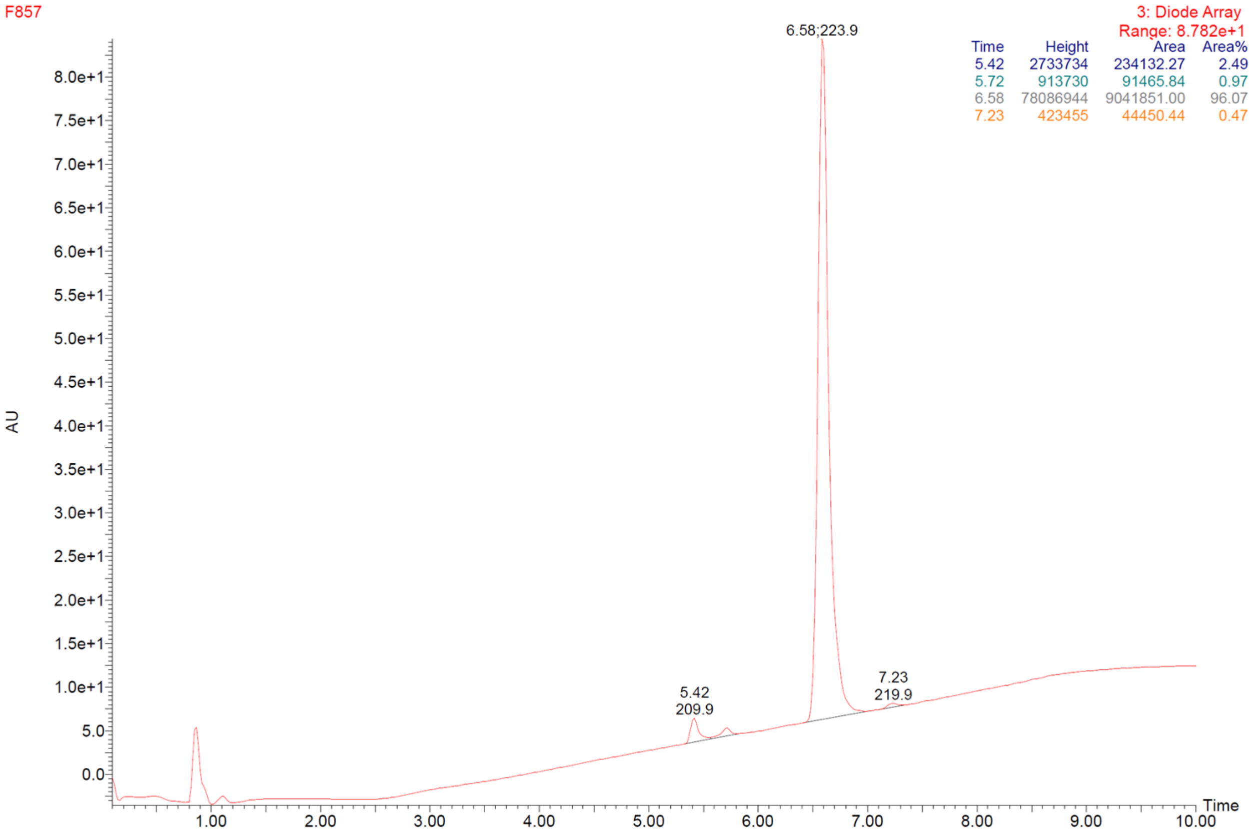


HPLC Chromatogram of compound C131.

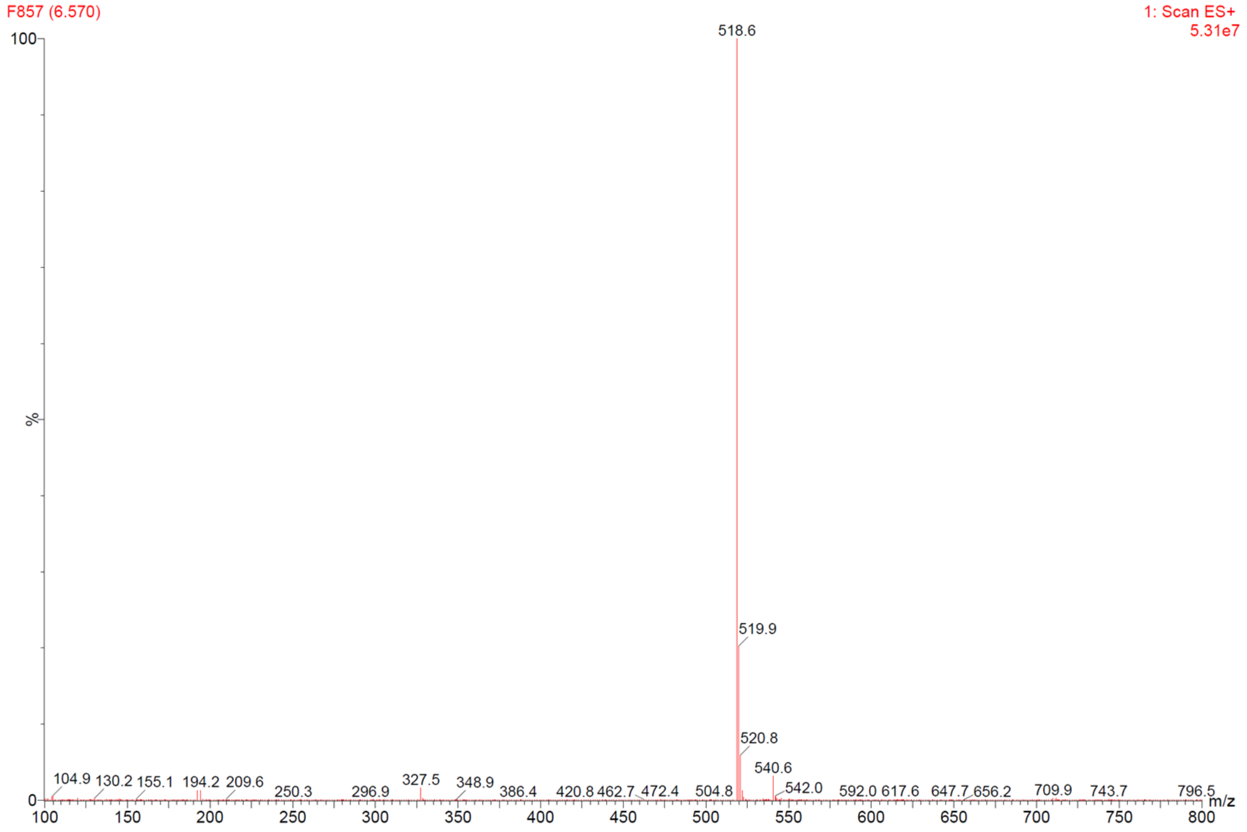


Mass spectrum of compound C131.

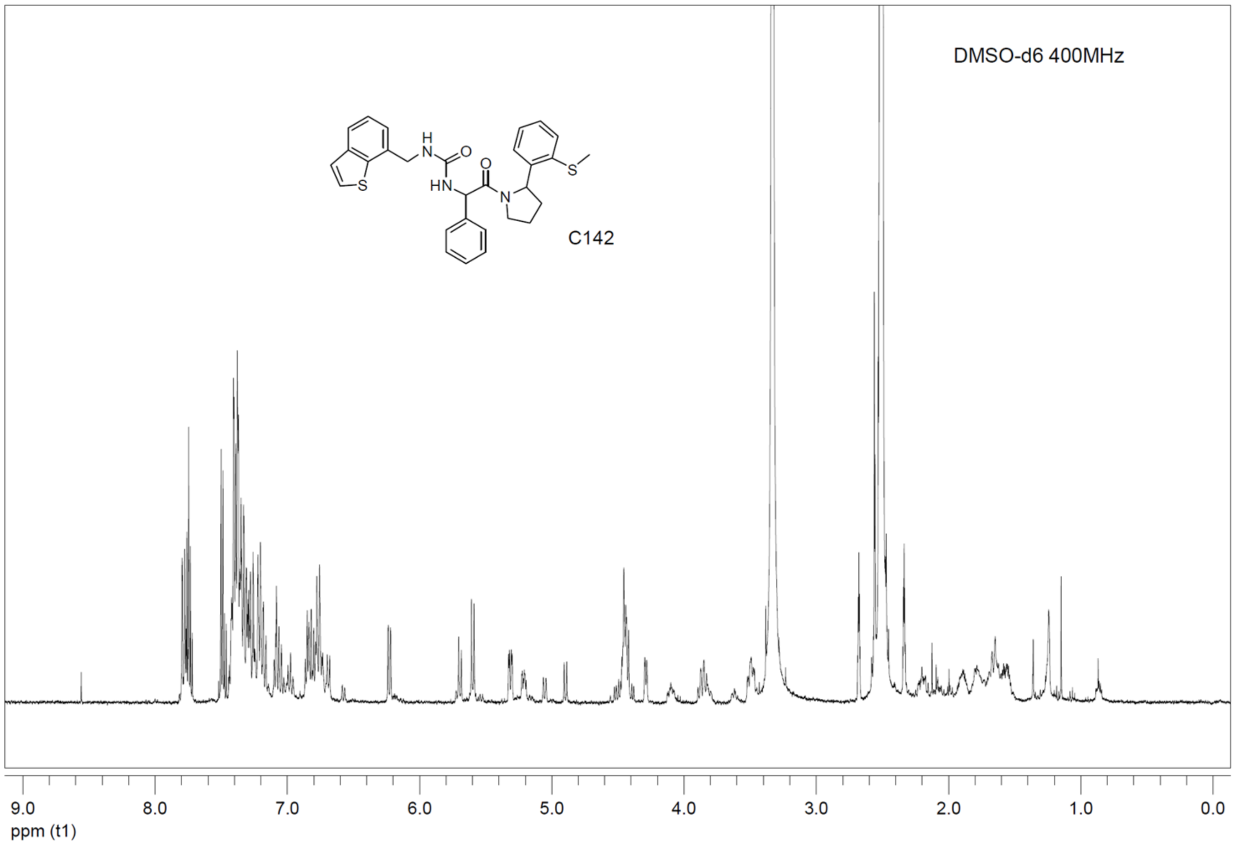


$^{1}$H NMR (400 MHz, DMSO-d6) spectrum of compound C142.

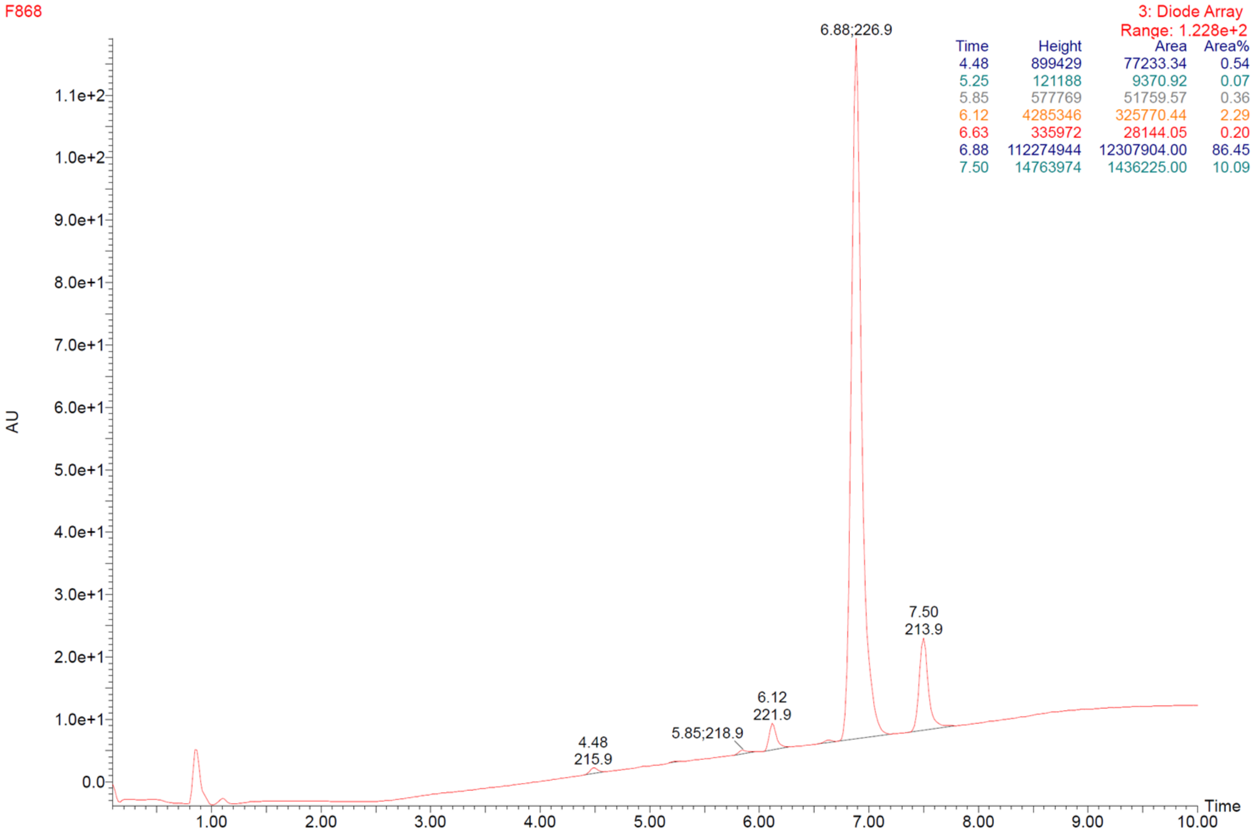


HPLC Chromatogram of compound C142.

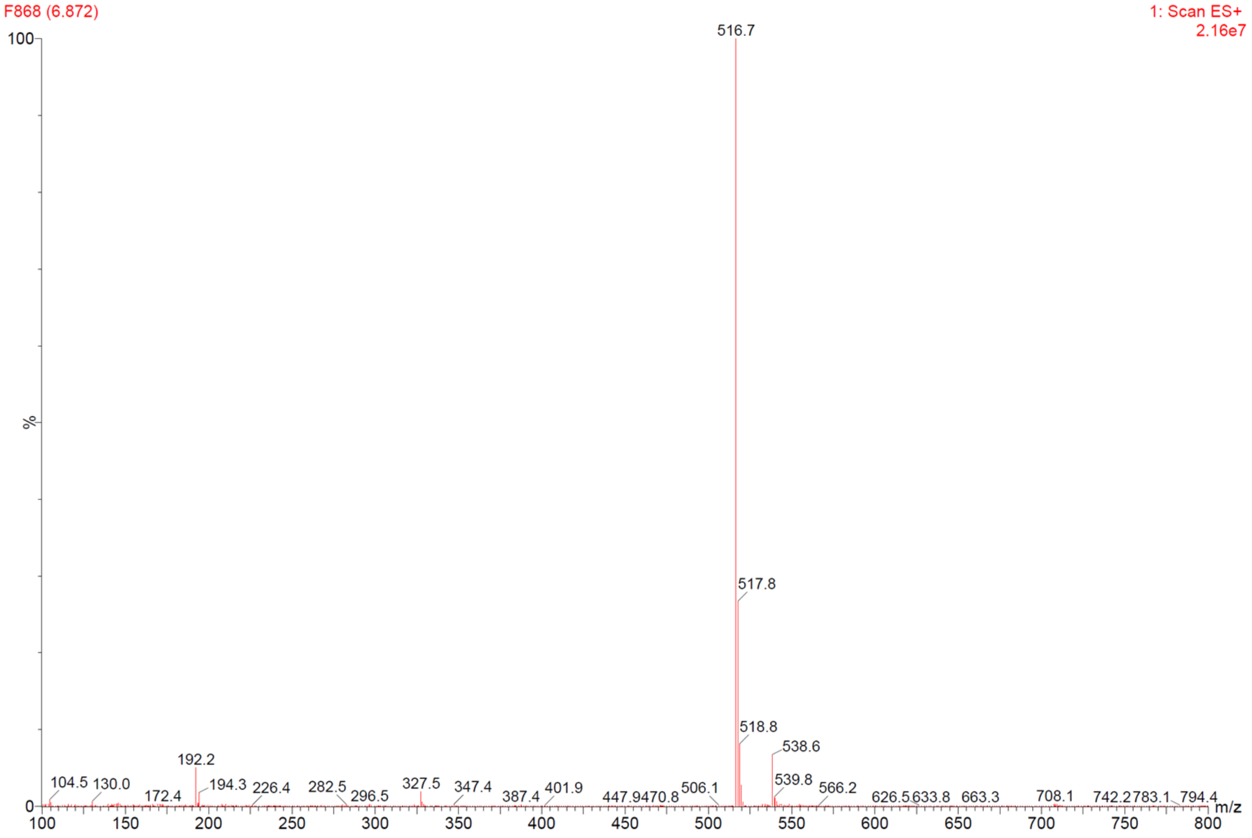


Mass spectrum of compound C142.

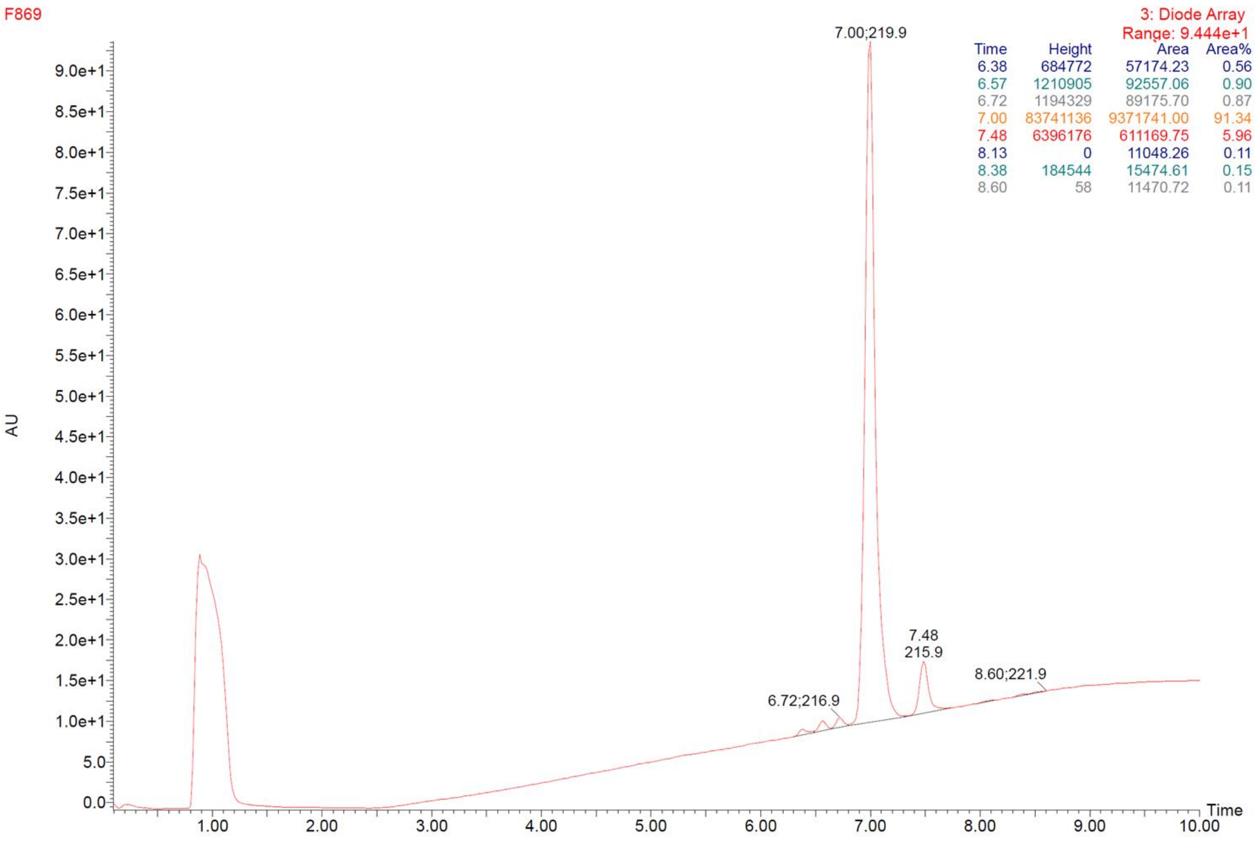


HPLC Chromatogram of compound C143.

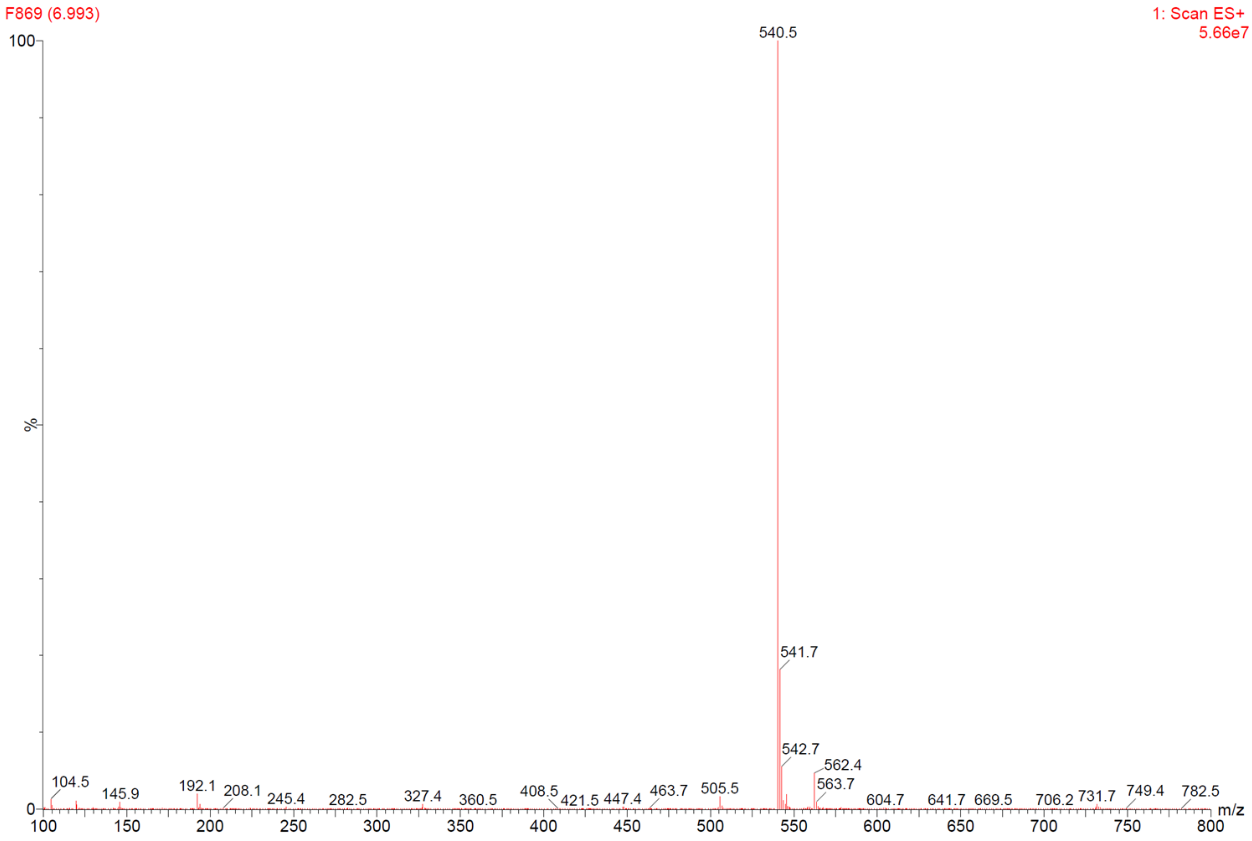


Mass spectrum of compound C143.

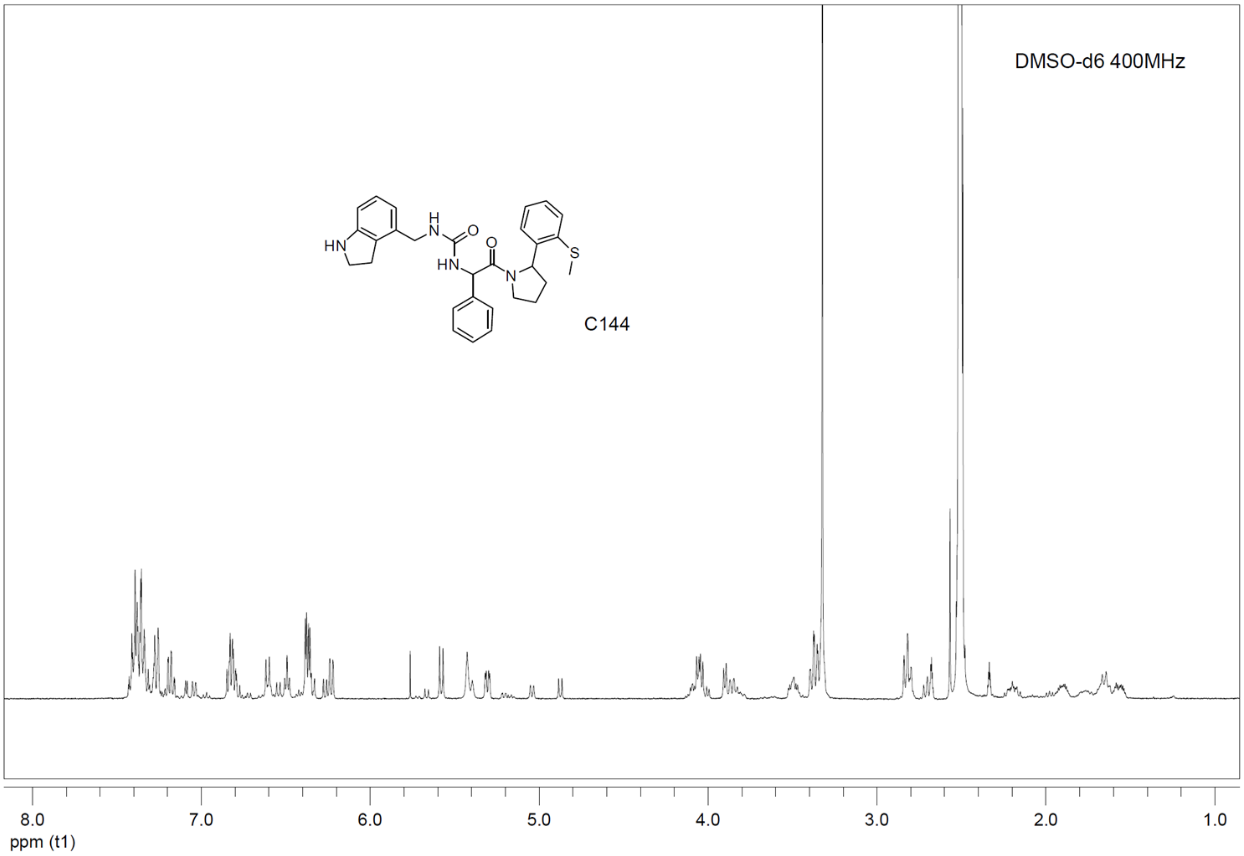


$^{1}$H NMR (400 MHz, DMSO-d6) spectrum of compound C144.

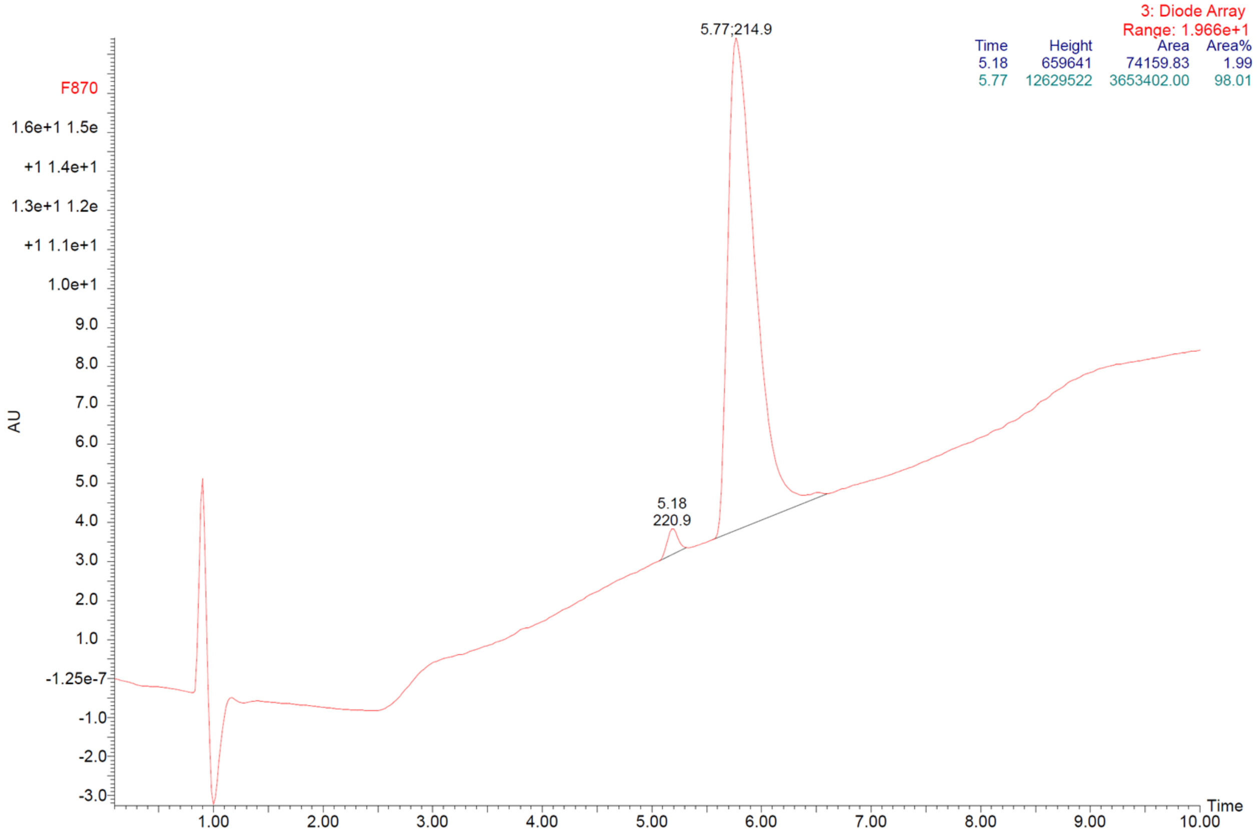


HPLC Chromatogram of compound C144.

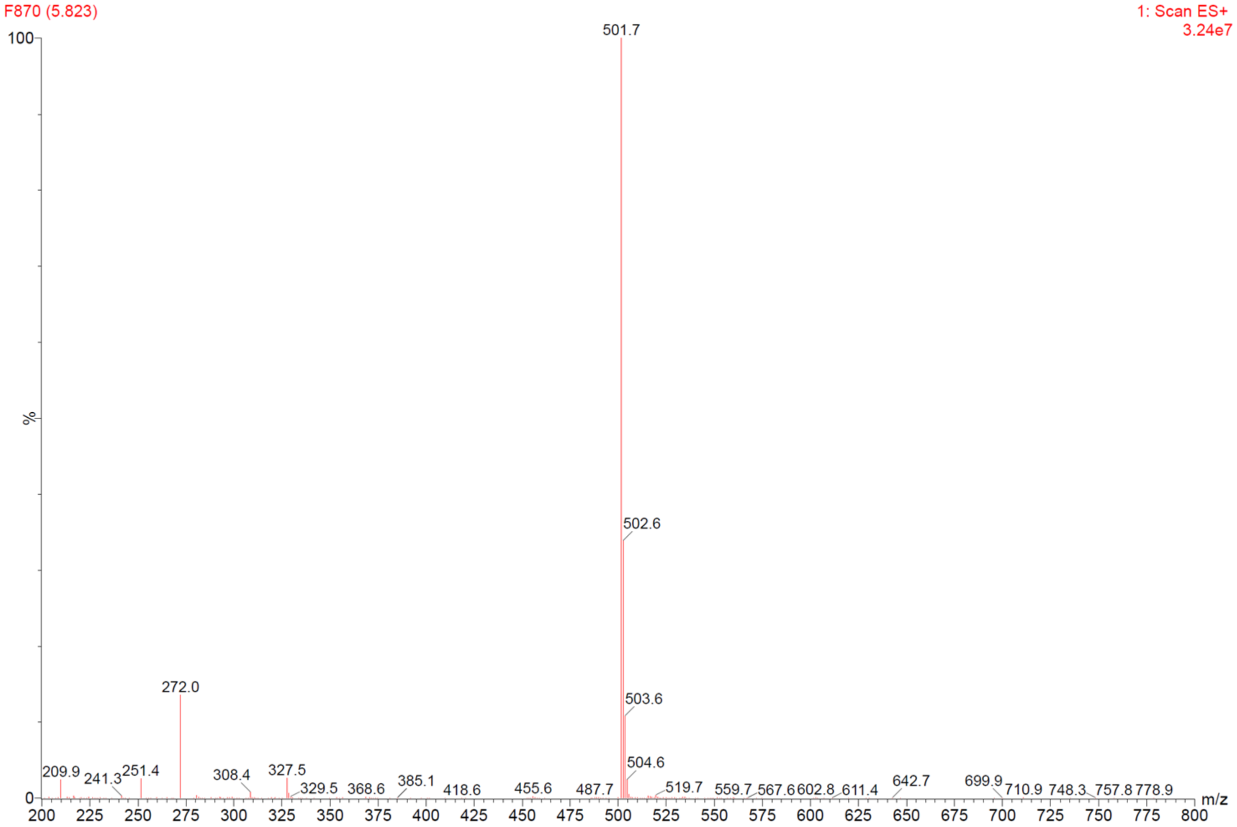


Mass spectrum of compound C144.

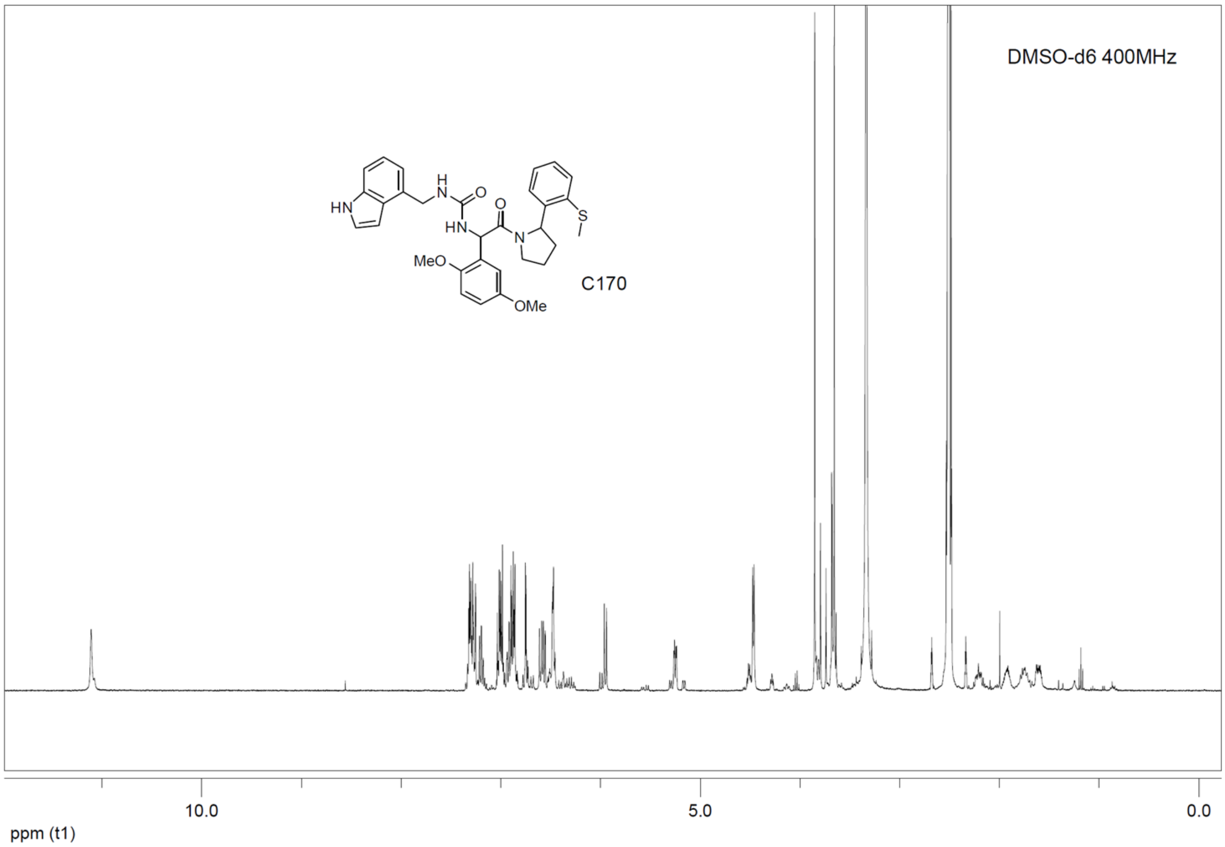


$^{1}$H NMR (400 MHz, DMSO-d6) spectrum of compound C170.

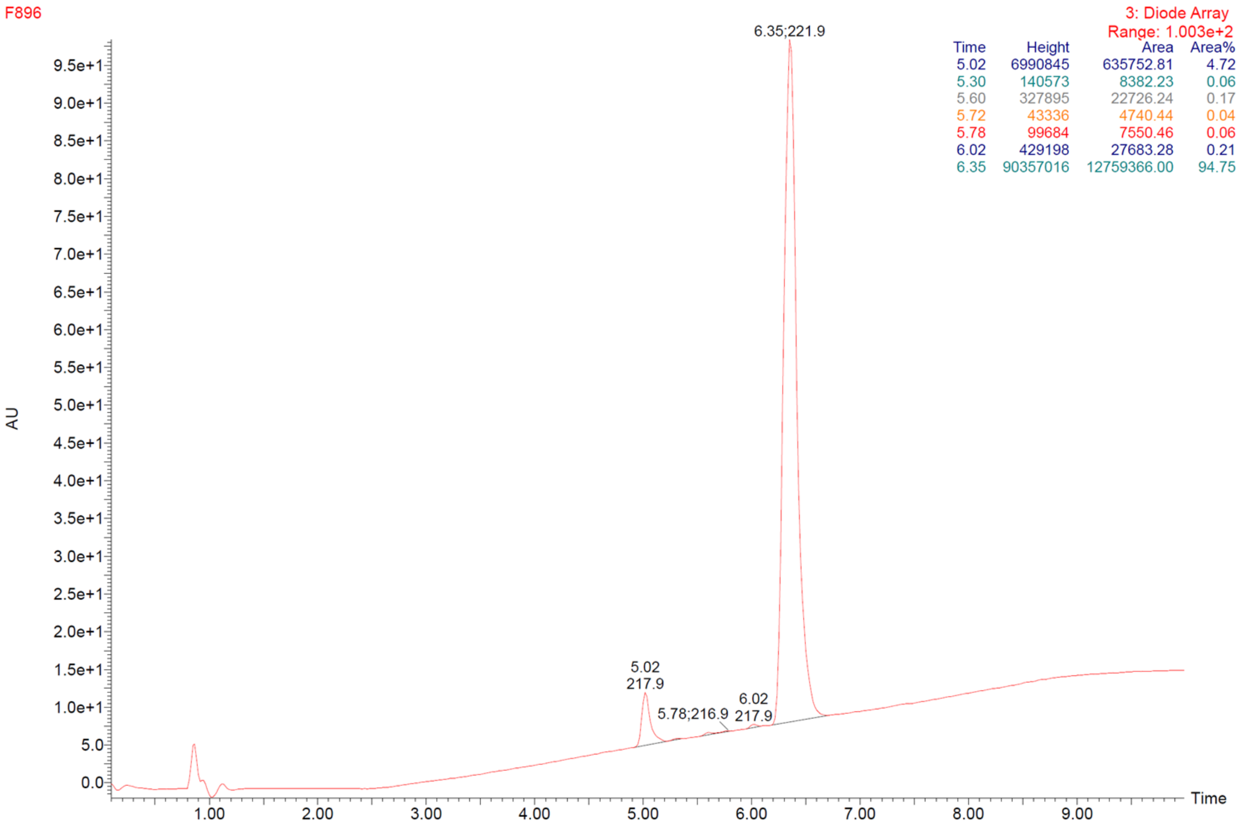


| Time | Height | Area | Area% |
|---|---|---|---|
| 5.02 | 6990845 | 635752.81 | 4.72 |
| 5.30 | 140573 | 8382.23 | 0.06 |
| 5.60 | 327895 | 22726.24 | 0.17 |
| 5.72 | 43336 | 4740.44 | 0.04 |
| 5.78 | 99684 | 7550.46 | 0.06 |
| 6.02 | 429198 | 27683.28 | 0.21 |
| 6.35 | 90357016 | 12759366.00 | 94.75 |

HPLC Chromatogram of compound C170.

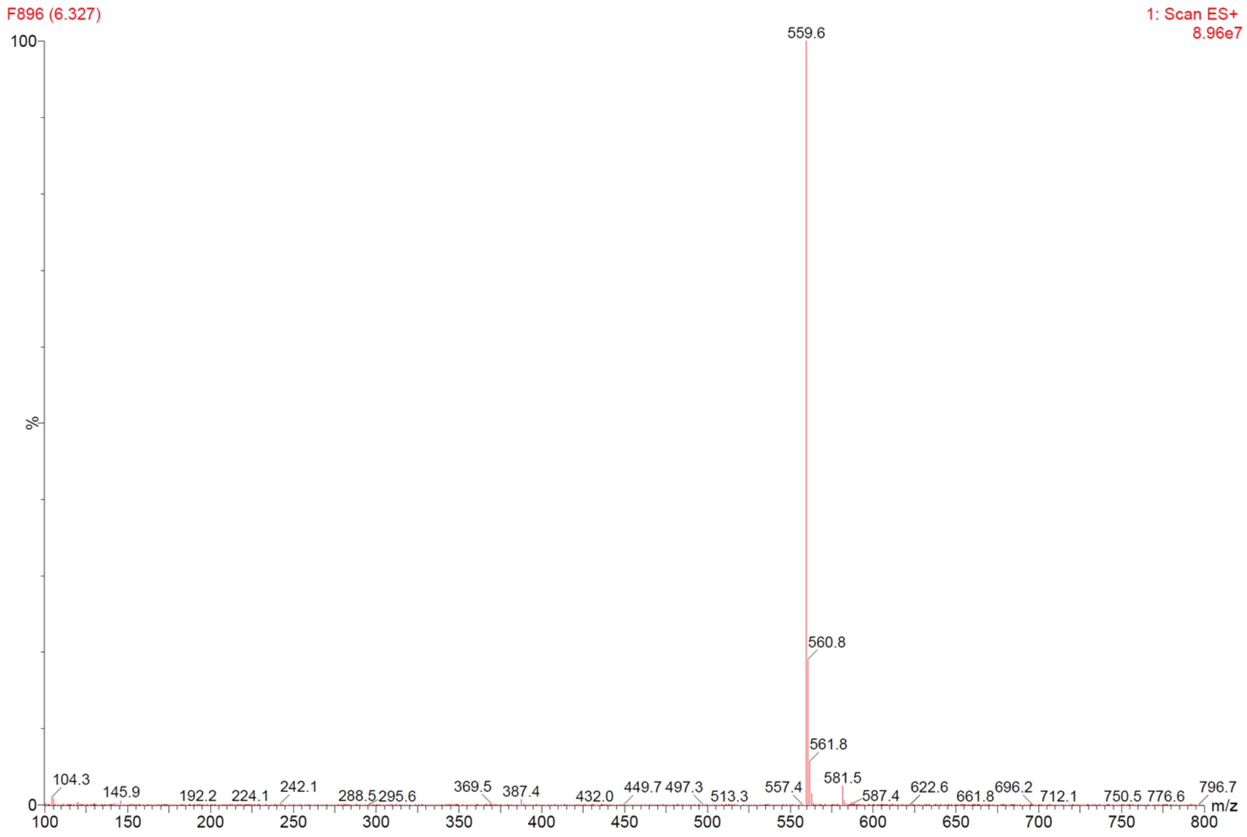


Mass spectrum of compound C170.

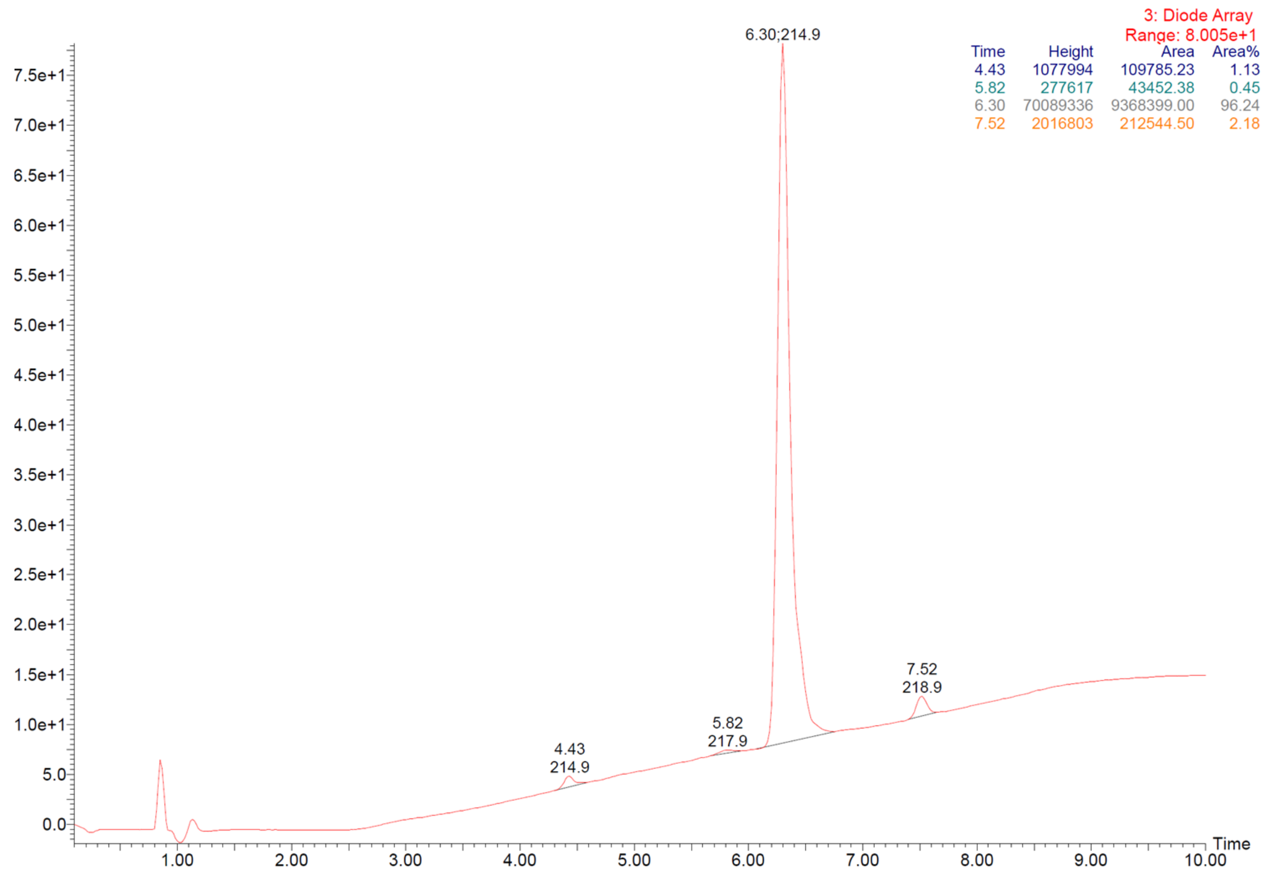


HPLC Chromatogram of compound C171.

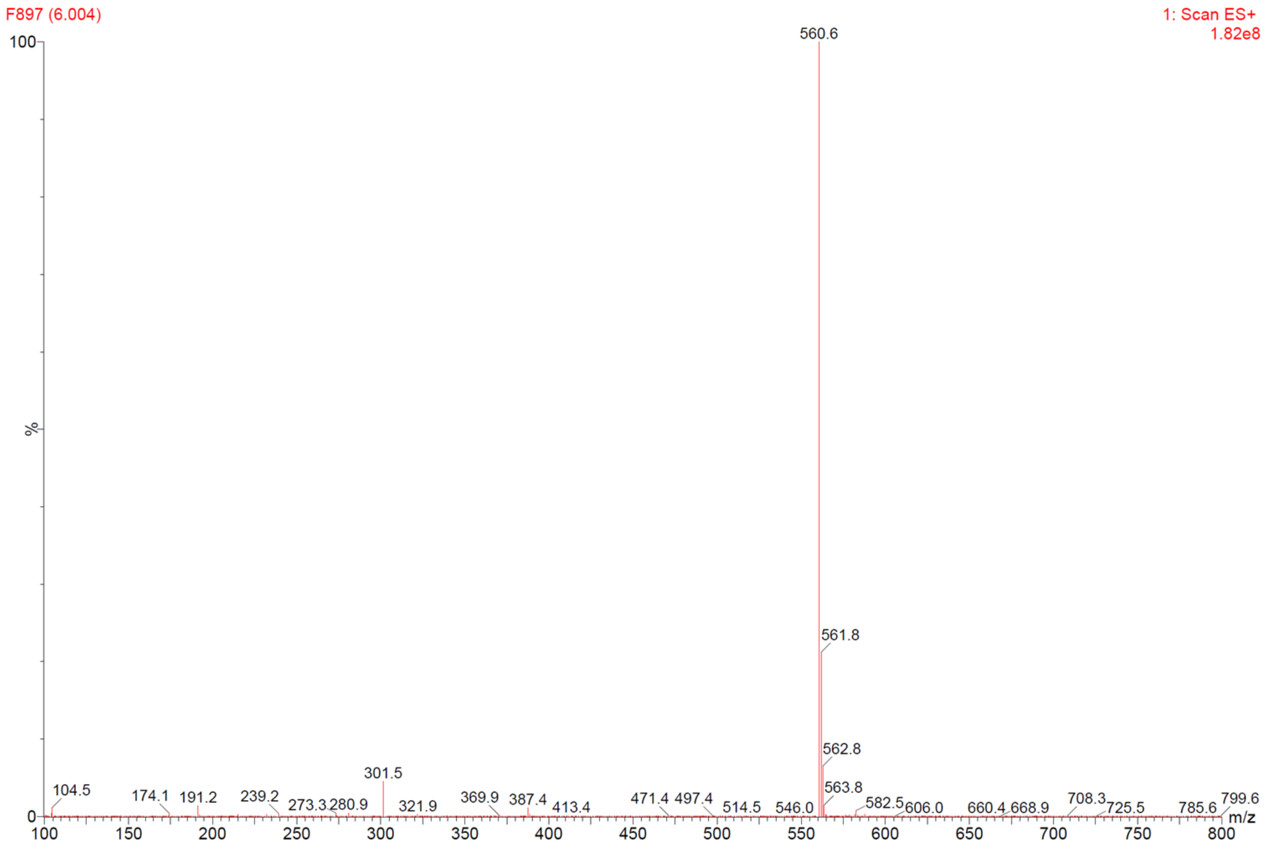


Mass spectrum of compound C171.

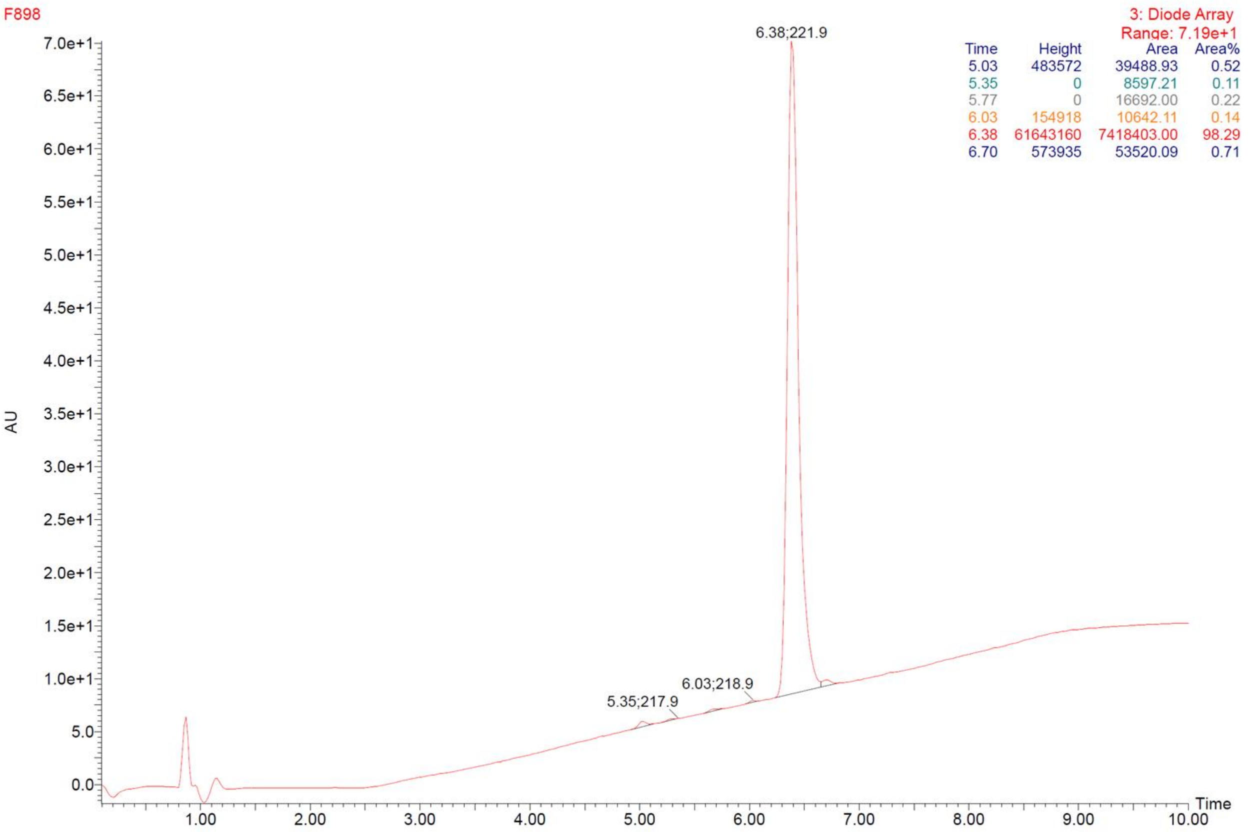


HPLC Chromatogram of compound C172.

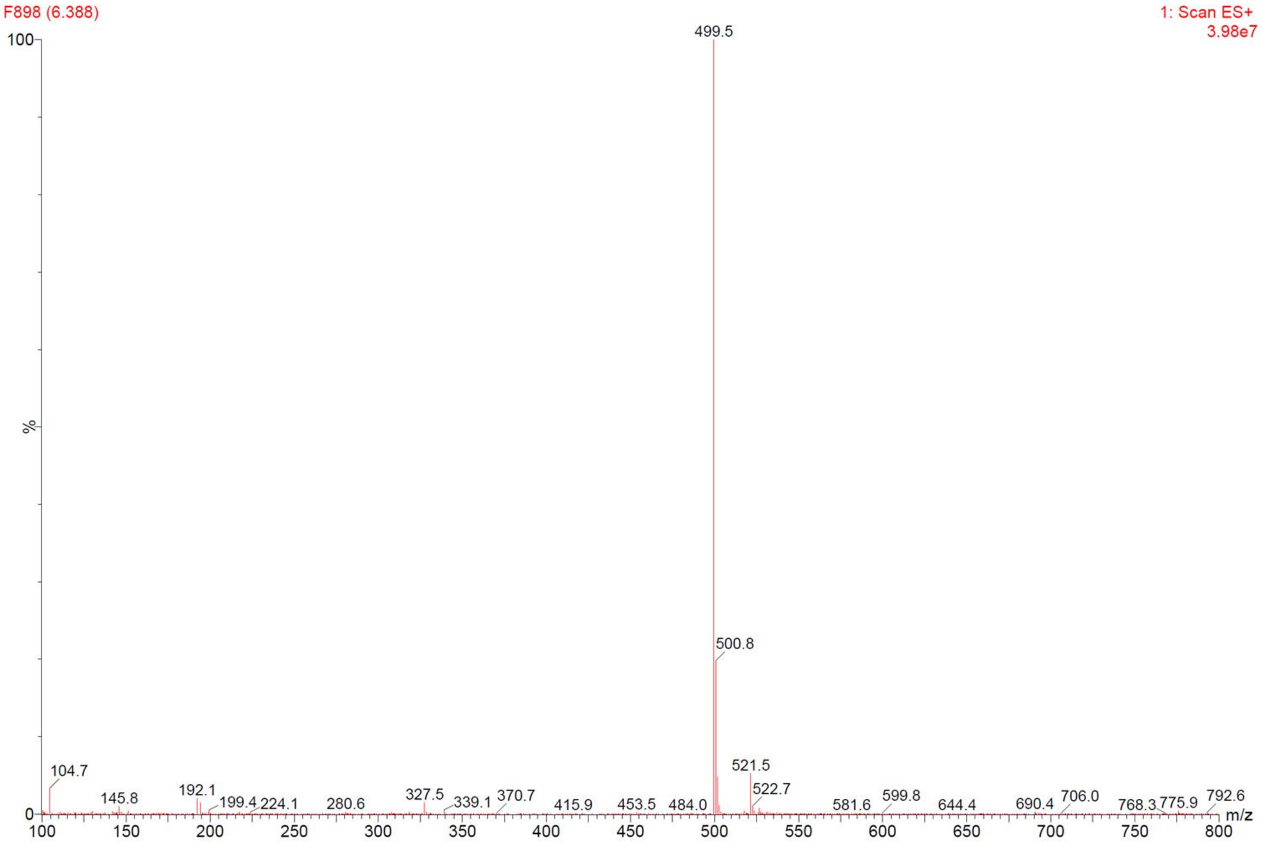


Mass spectrum of compound C172.

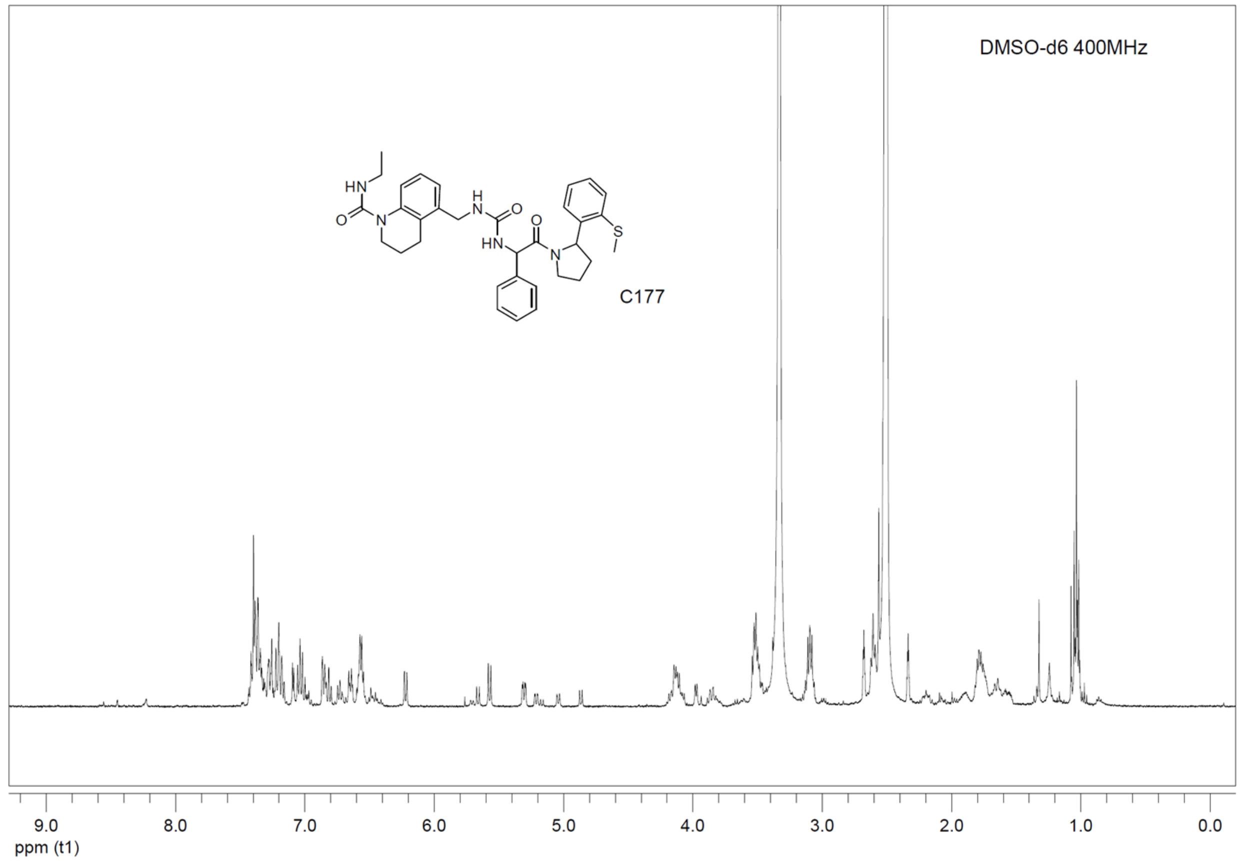


$^{1}$H NMR (400 MHz, DMSO-d6) spectrum of compound C177.

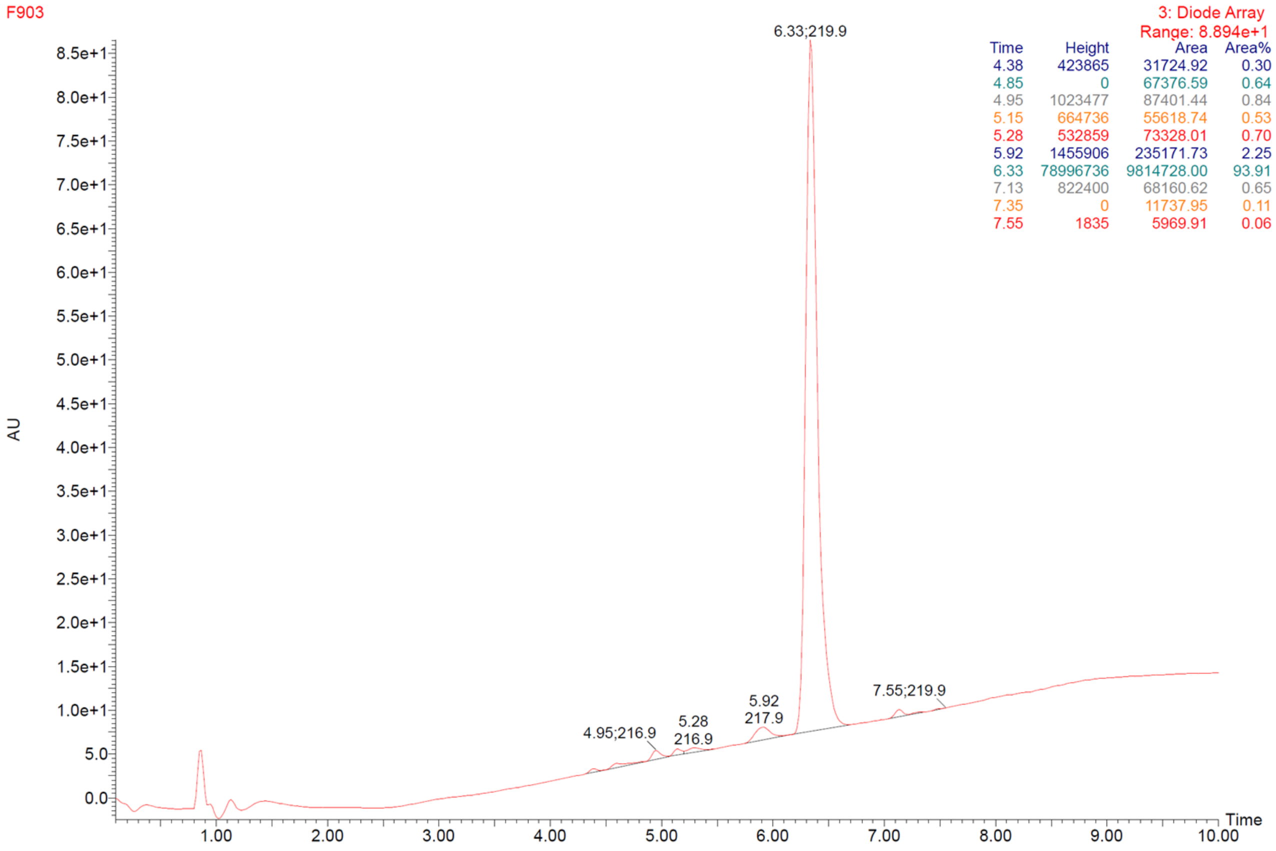


HPLC Chromatogram of compound C177.

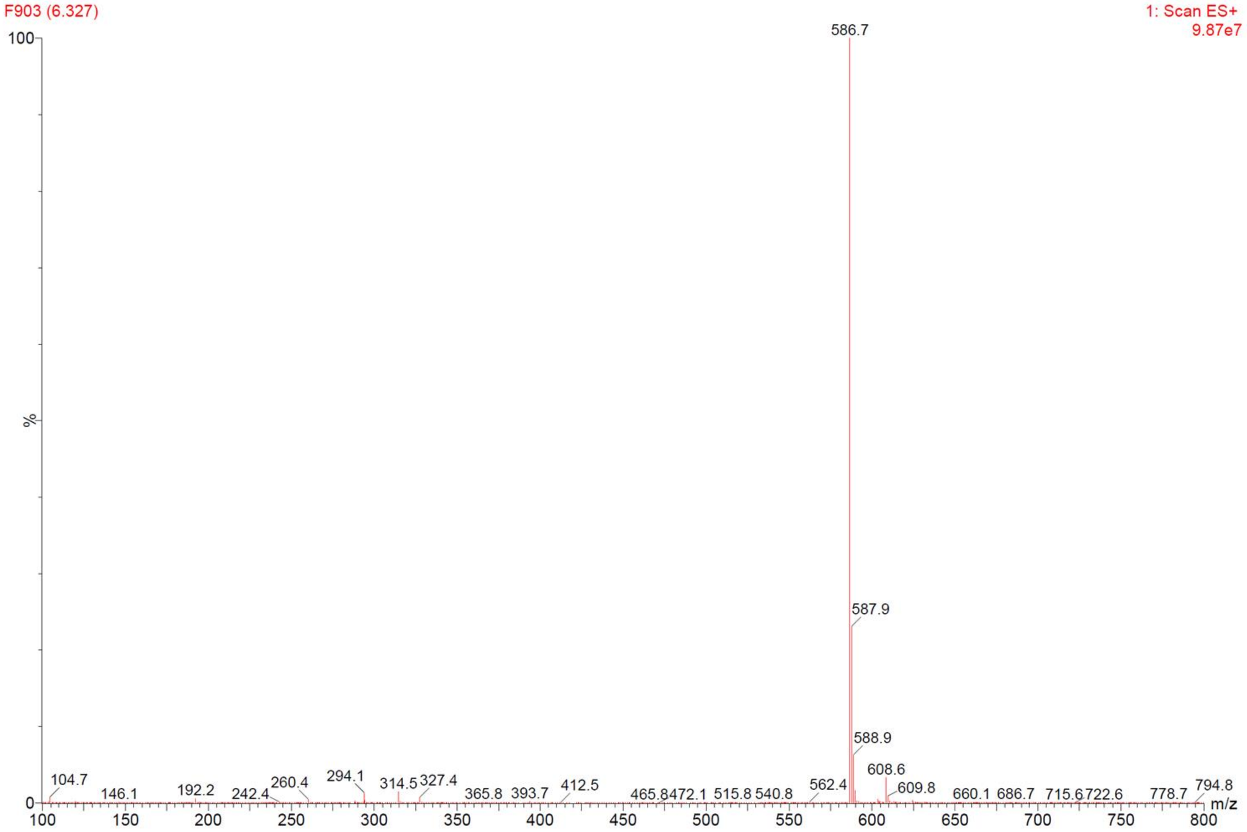


Mass spectrum of compound C177.

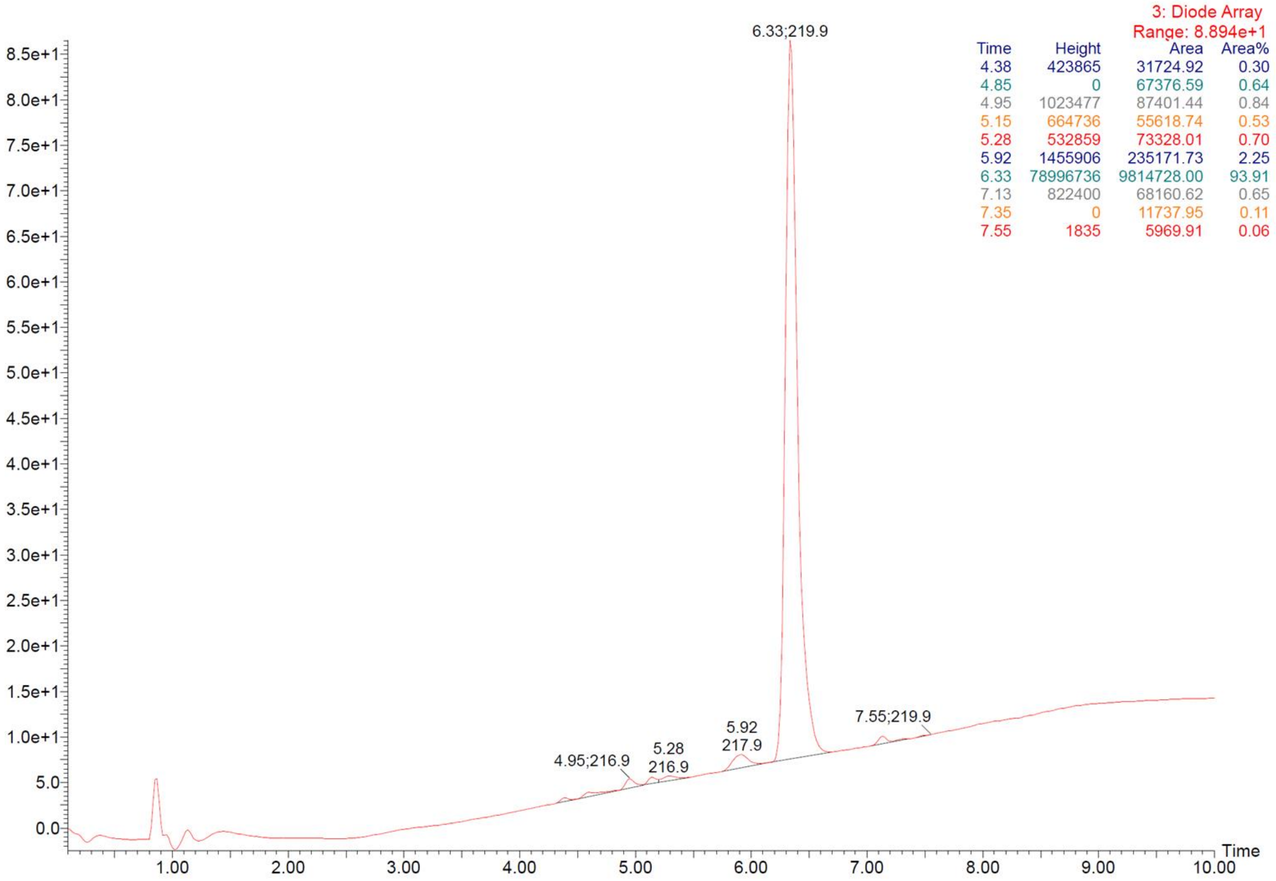


HPLC Chromatogram of compound C178.

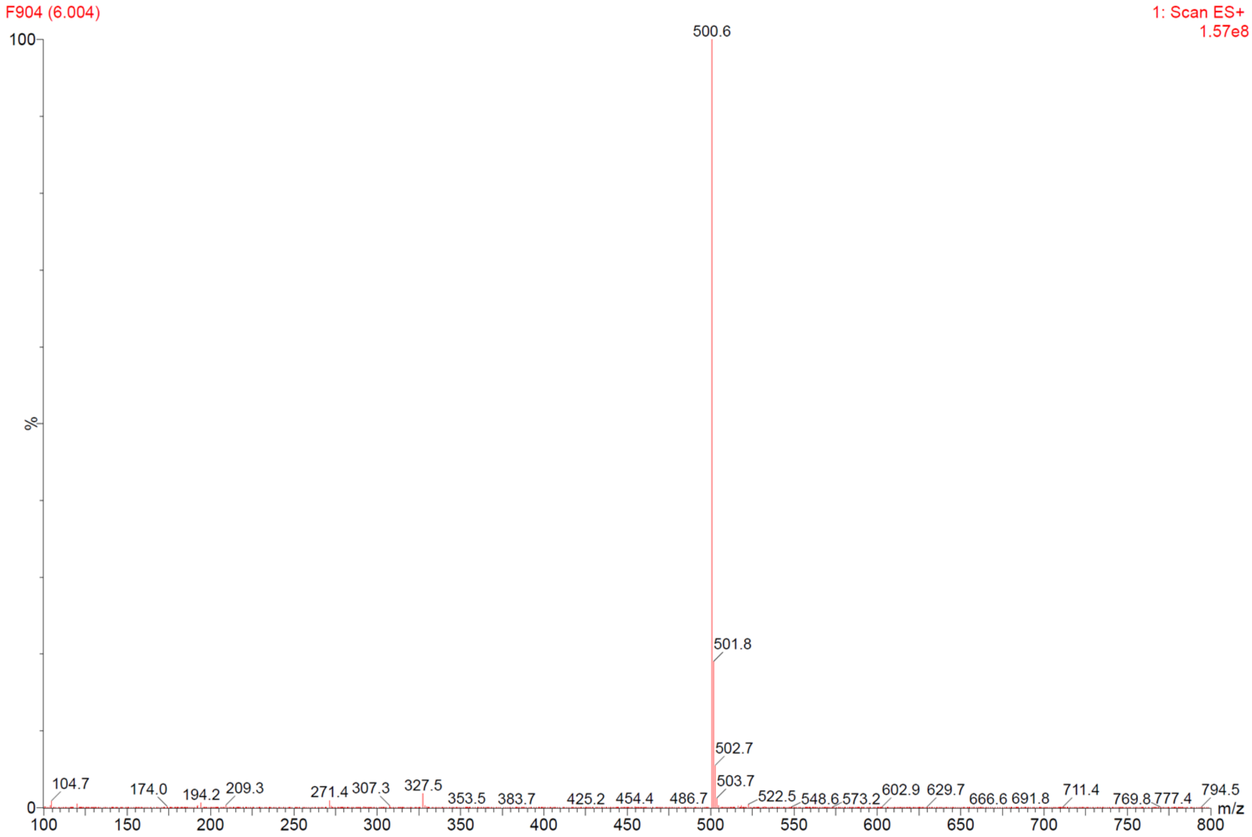


Mass spectrum of compound C178.

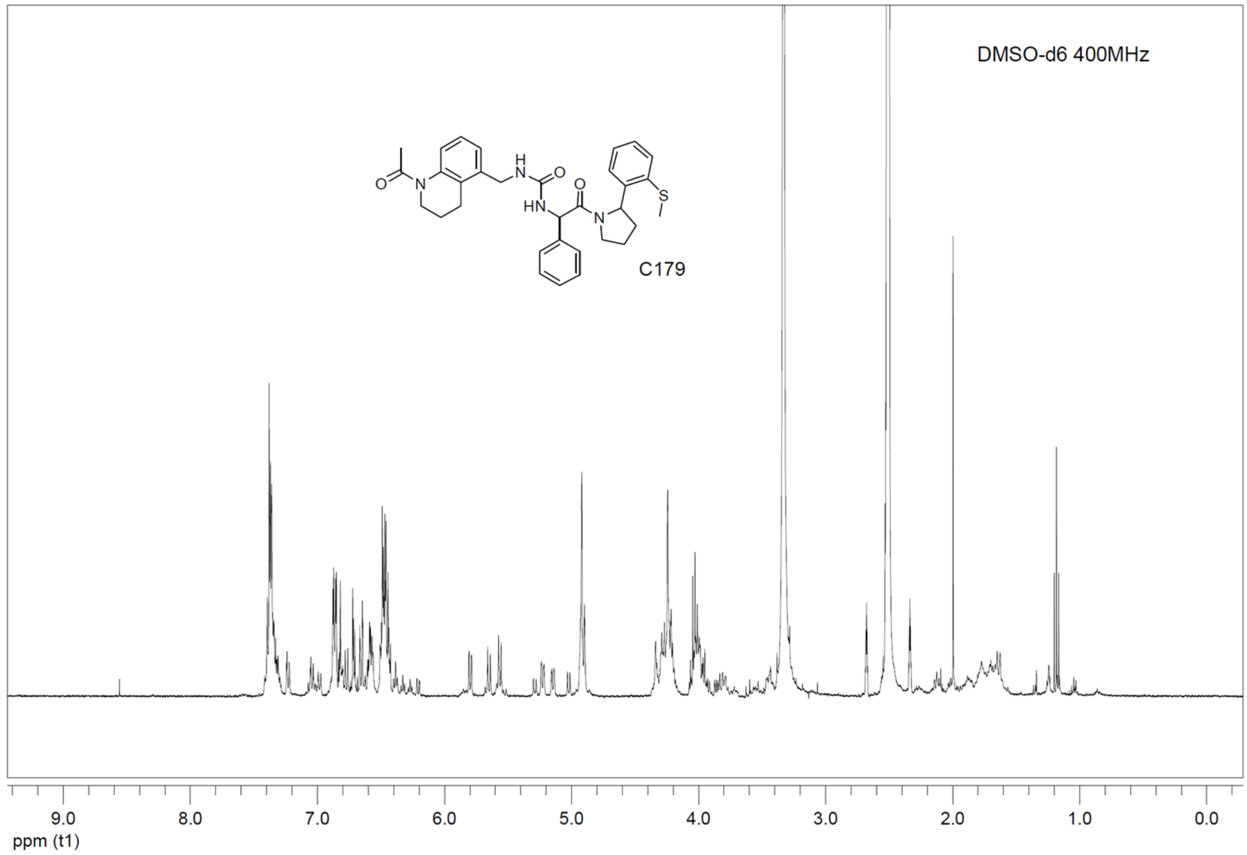


$^1$H NMR (400 MHz, DMSO-d6) spectrum of compound C179.

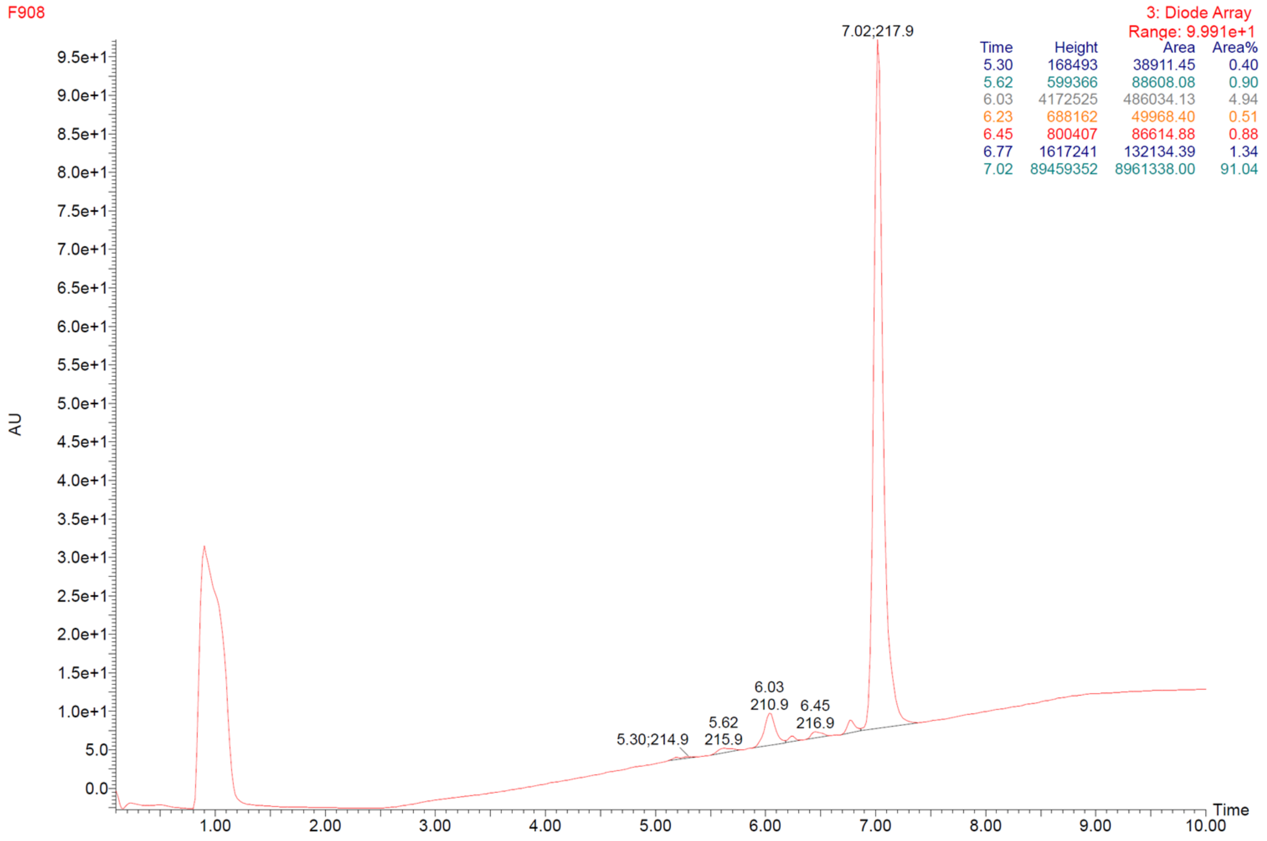


HPLC Chromatogram of compound C182.

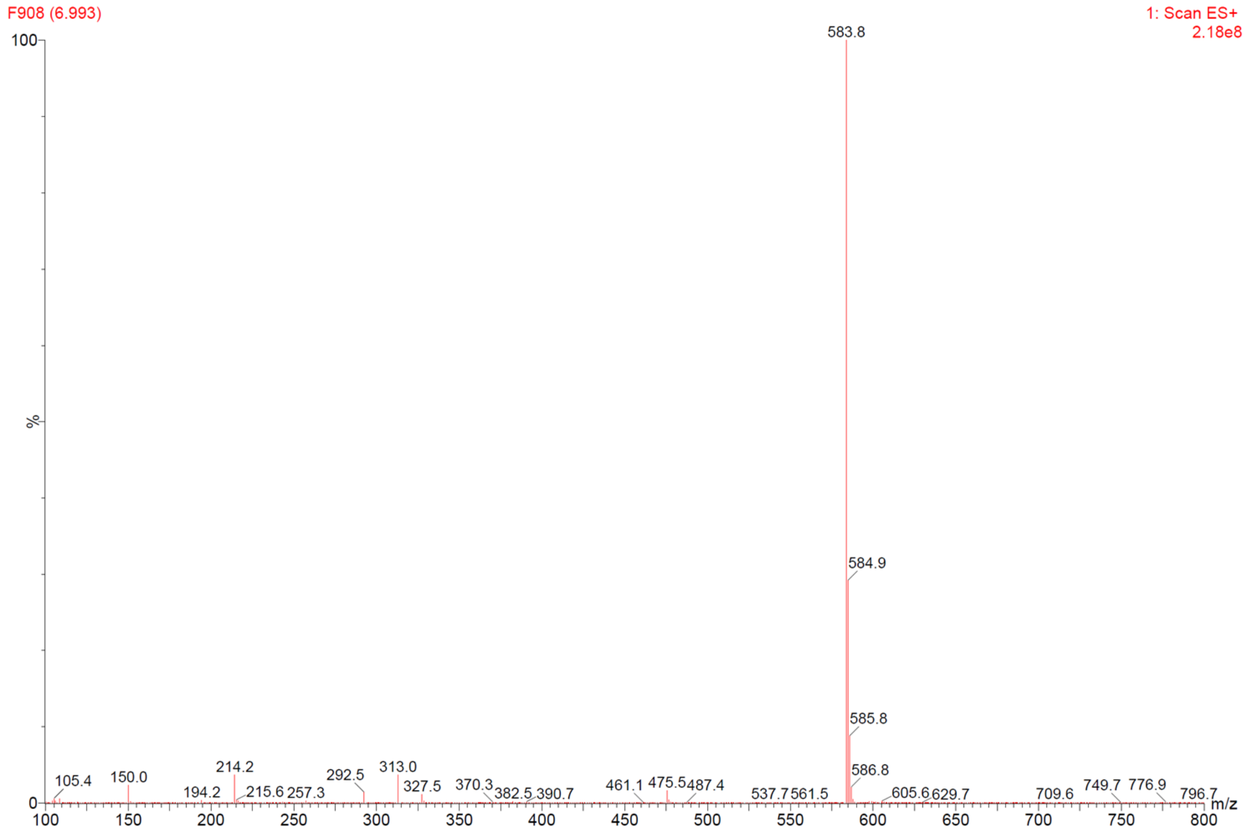


Mass spectrum of compound C182.

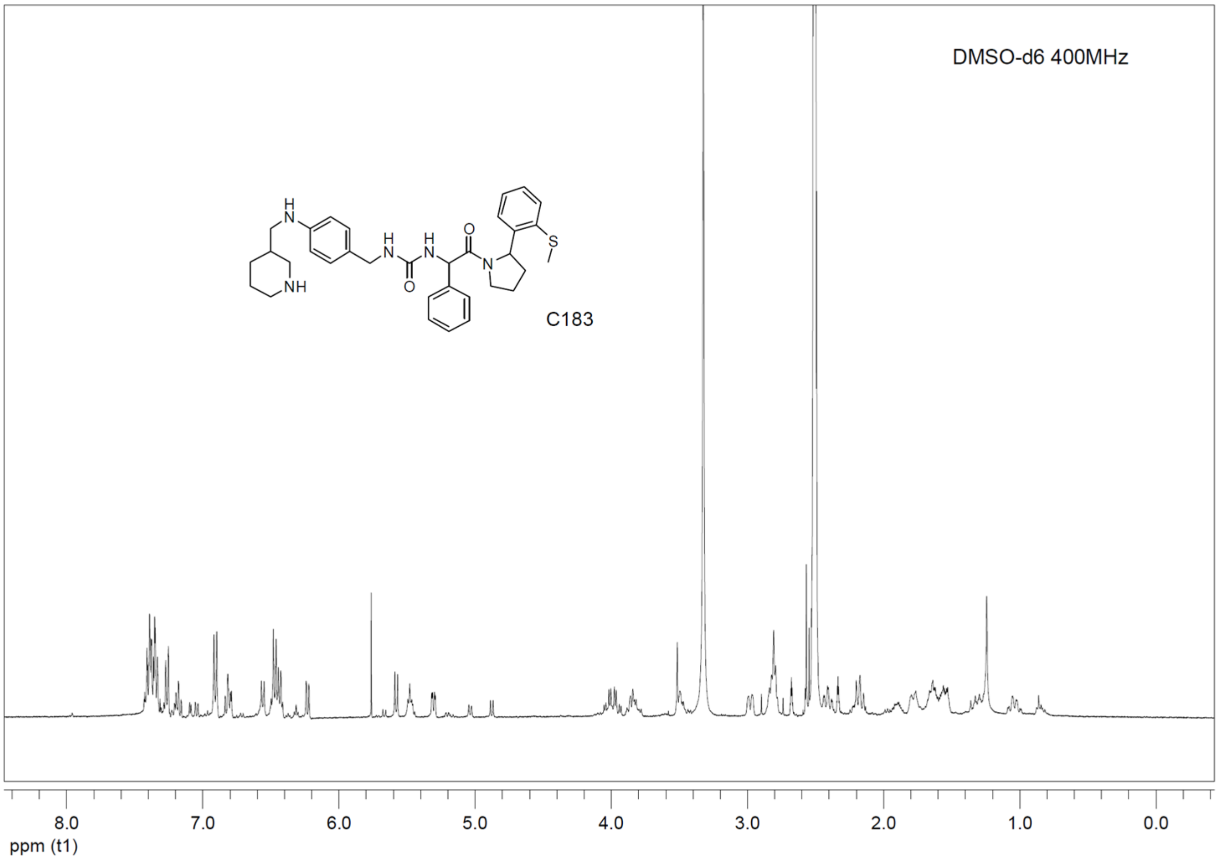


$^{1}$H NMR (400 MHz, DMSO-d6) spectrum of compound C183.

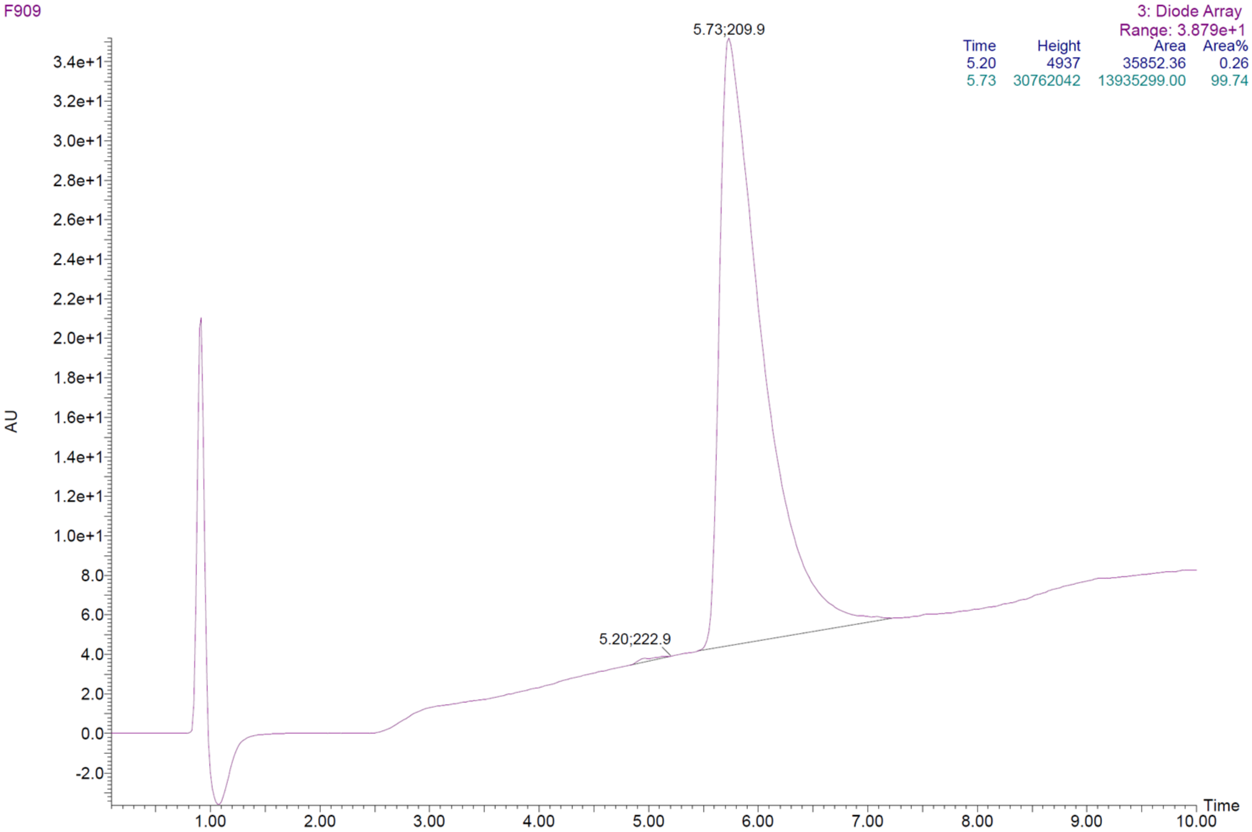


HPLC Chromatogram of compound C183.

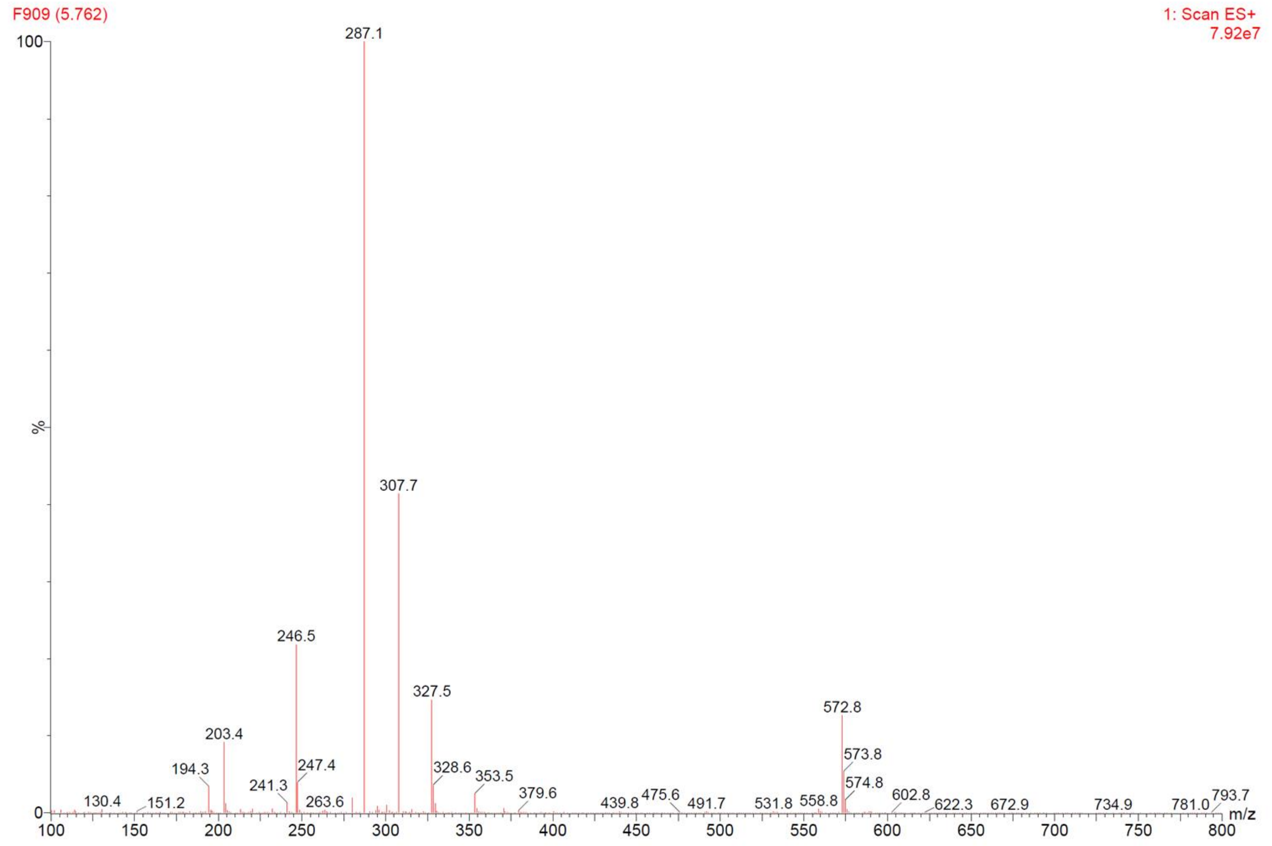


Mass spectrum of compound C183.

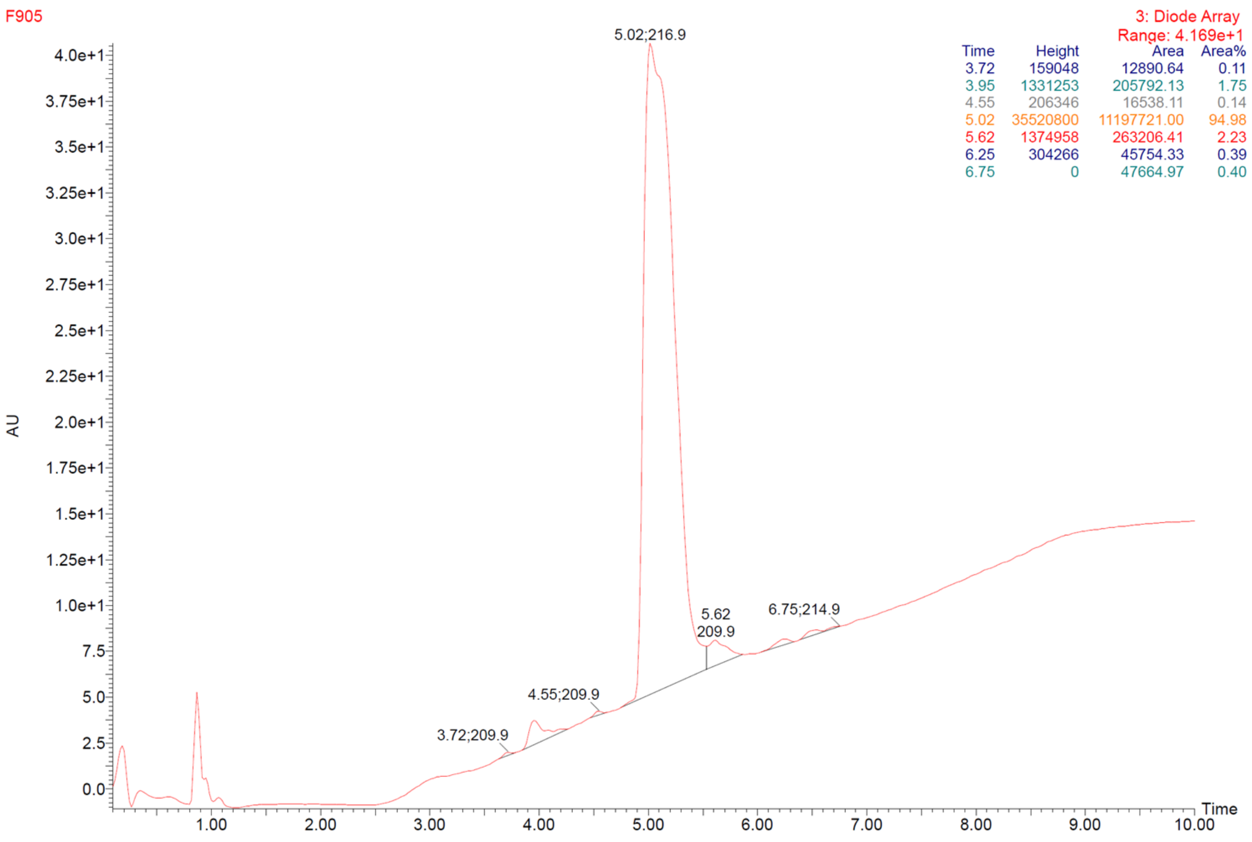


HPLC Chromatogram of compound C185.

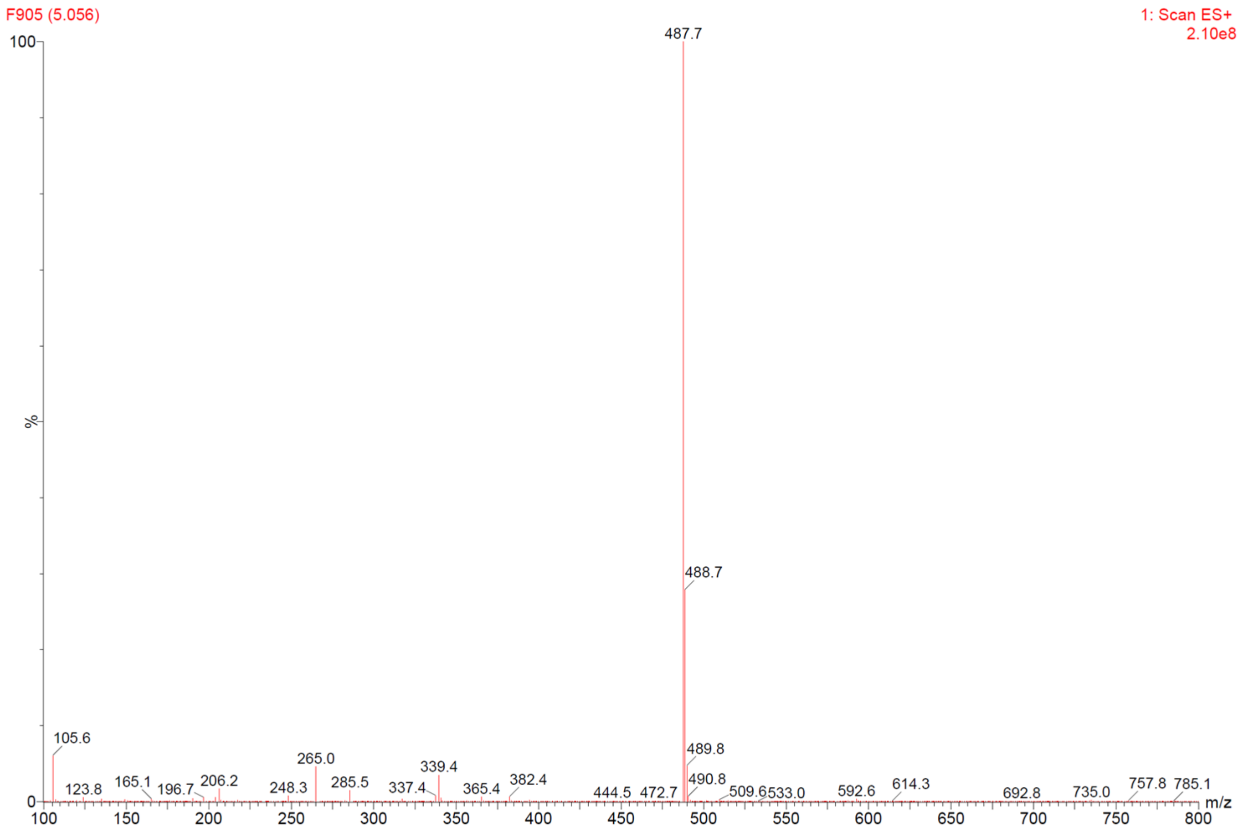


Mass spectrum of compound C185.

C32

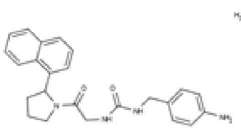

C71

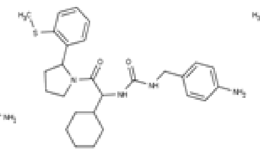

C90

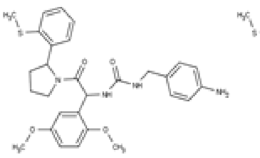

C99

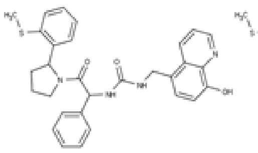

C101

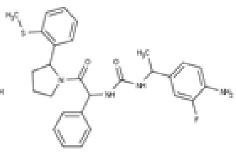

C104

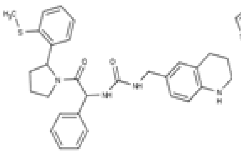

C105

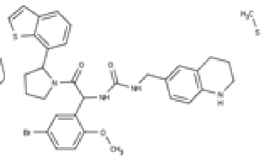

C106

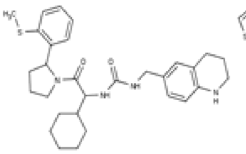

C107

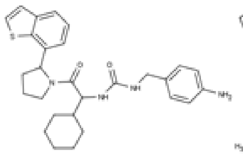

C108

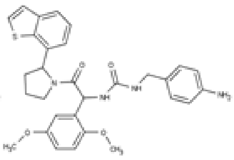

C110

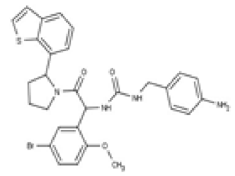

C112

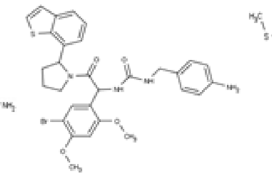

C115

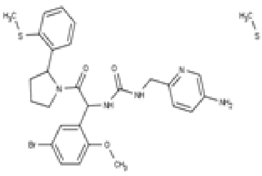

C119

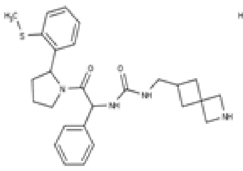

C120

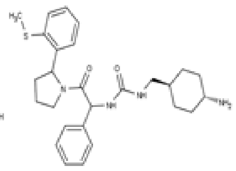

C123

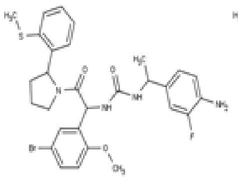

C129

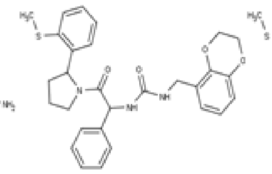

C131

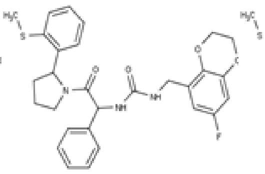

C142

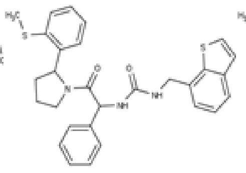

C143

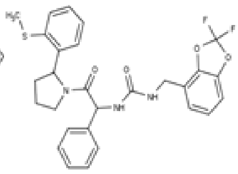

C144

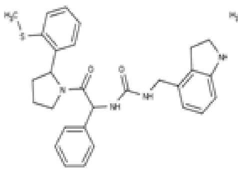

C170

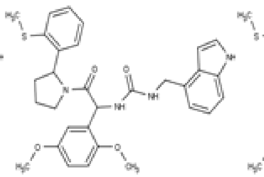

C171

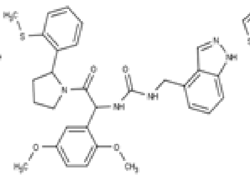

C172

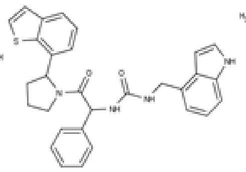

C173

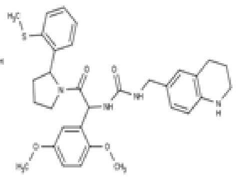

C177

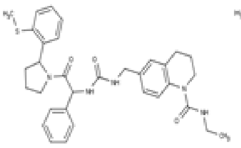

C178

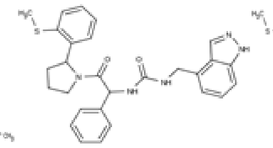

C179

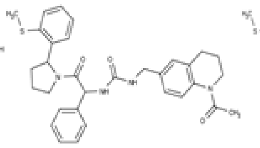

C182

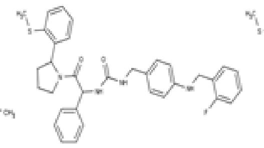

C183

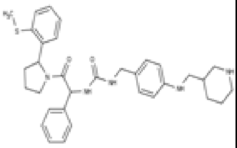

C185

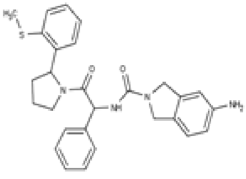

**Supplemental Figure 1:** Chemical structures of C31 derivatives used in this study.

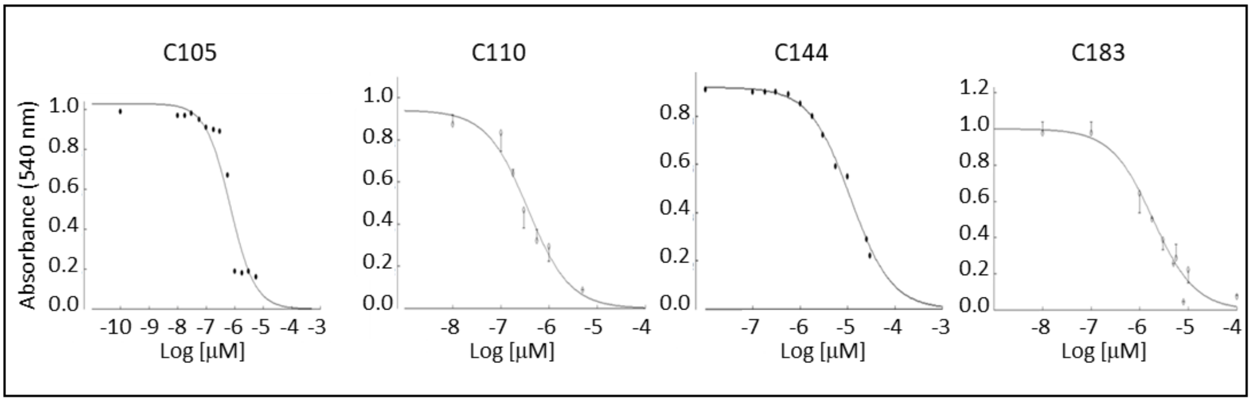


**Supplemental Figure 2:** Concentration-response curves of mitochondrial swelling inhibition by compounds C105, C110, C144 and C183.

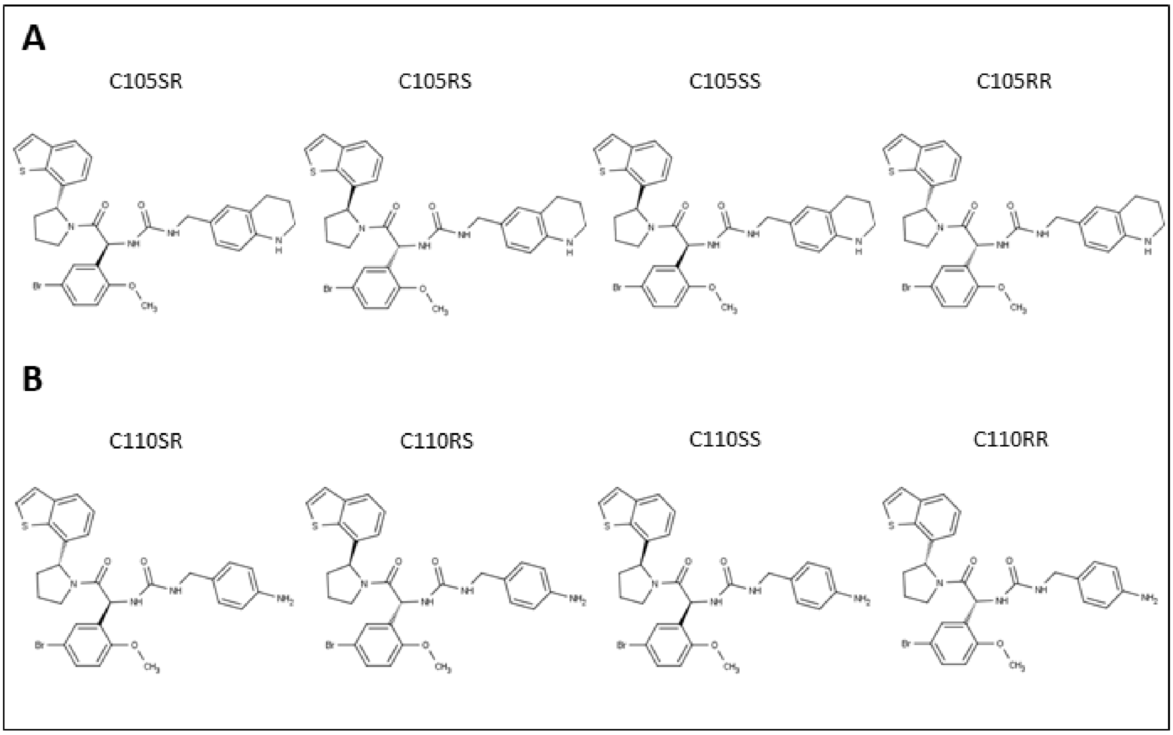


**Supplemental Figure 3:** Chemical structures of the four diastereoisomers of C105 (A) and C110 (B).

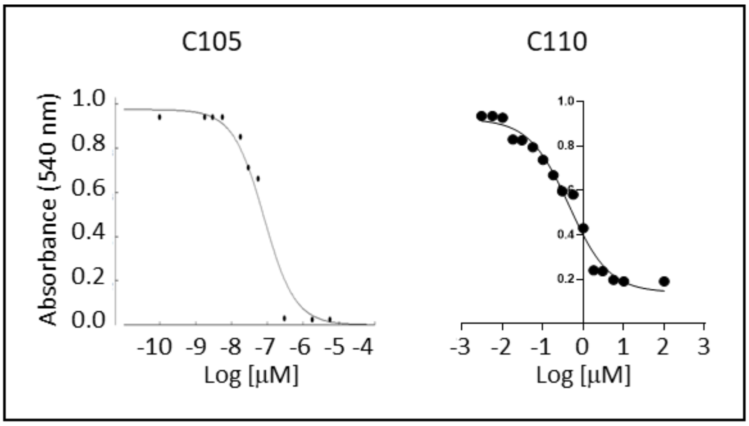


**Supplemental Figure 4:** Concentration-response curves of mitochondrial swelling inhibition by compounds C105SR and C110SR.

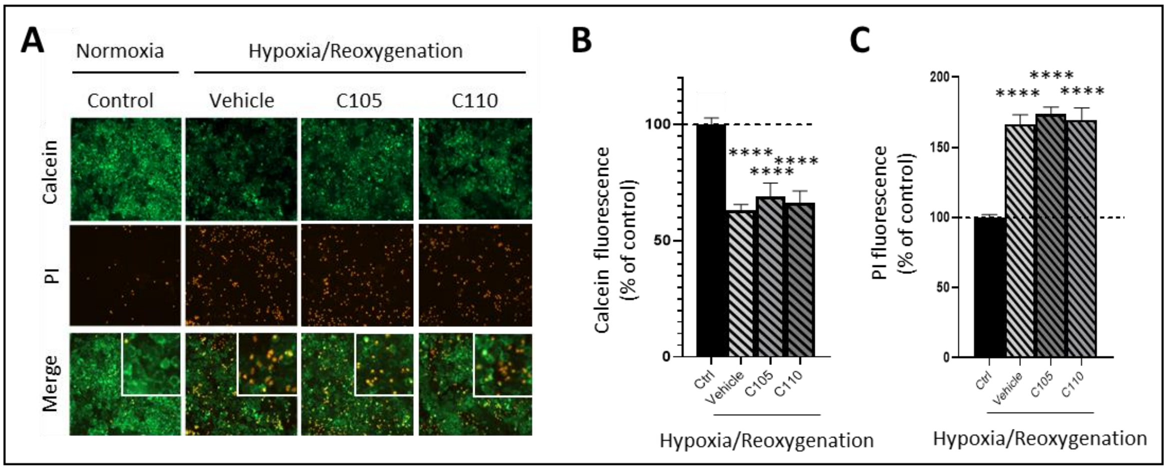


**Supplemental Figure 5:** Absence of inhibition of mPTP opening and reduction of necrosis by C105 and C110 at 1 µM *in vitro* in a model of hepatic hypoxia/reoxygenation. Cells were pretreated with 1 µM calcein and 1 mM CoCl2 for 30 min and 10 min, respectively, then subjected to 4 h of hypoxia (1% O2) followed by 1 h of reoxygenation (21% O2) in the presence of 3 µM propidium iodide (PI). C105 and C110 were added at 1 µM for the entire duration of hypoxia/reoxygenation. (A) Representative images of calcein (green) and PI (red) labeling in cells exposed to normoxia (control) or hypoxia/reoxygenation in the absence (vehicle) or in the presence of C105 or C110 (original magnification × 400). (B) Calcein fluorescence in cells exposed to normoxia (Ctrl) or hypoxia/reoxygenation in the absence (vehicle) or in the presence of C105 or C110. ****p < 0.0001 vs Ctrl. (C) PI fluorescence in cells exposed to normoxia (Ctrl) or hypoxia/reoxygenation in the absence (vehicle) or in the presence of C105 or C110. ****p < 0.0001 vs Ctrl.

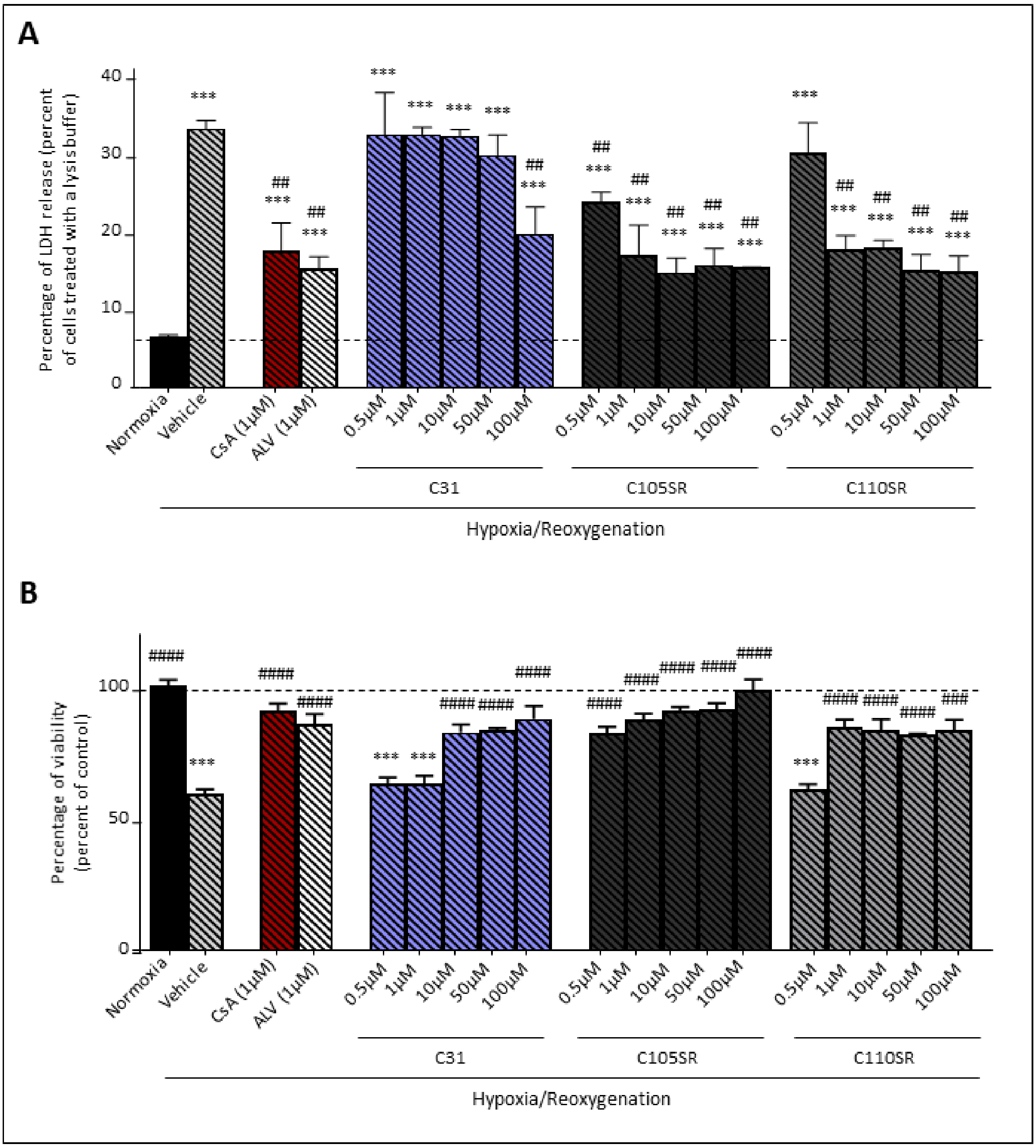


**Supplemental Figure 6**: Protection against cell death by pharmacological perconditioning with C105SR and C110SR. Cells were subjected to 4 h of hypoxia (1% $O_2$) followed by 2 h of reoxygenation (21% $O_2$). CsA and ALV were used as references. CsA and ALV were added at 1 µM while C31, C105SR and C110SR were added at increasing concentrations during the hypoxic phase. (A) LDH release from cells exposed to normoxia (Ctrl) or hypoxia/reoxygenation in the absence (vehicle) or in the presence of CsA, ALV or increasing concentrations of C31, C105SR or C110SR expressed as percentage of LDH release in cells treated with a lysis buffer (Ctrl). ***p < 0.001 vs Ctrl; ##p < 0.01 vs hypoxia/reoxygenation vehicle. (B) Cell viability measured by MTT assay in cells exposed to normoxia (Ctrl) or hypoxia/reoxygenation in the absence (vehicle) or in the presence of CsA, ALV or increasing concentrations of C31, C105SR or C110SR expressed as percentage of control (Ctrl). ***p < 0.001 vs Ctrl ; ##p < 0.05 vs hypoxia/reoxygenation vehicle.

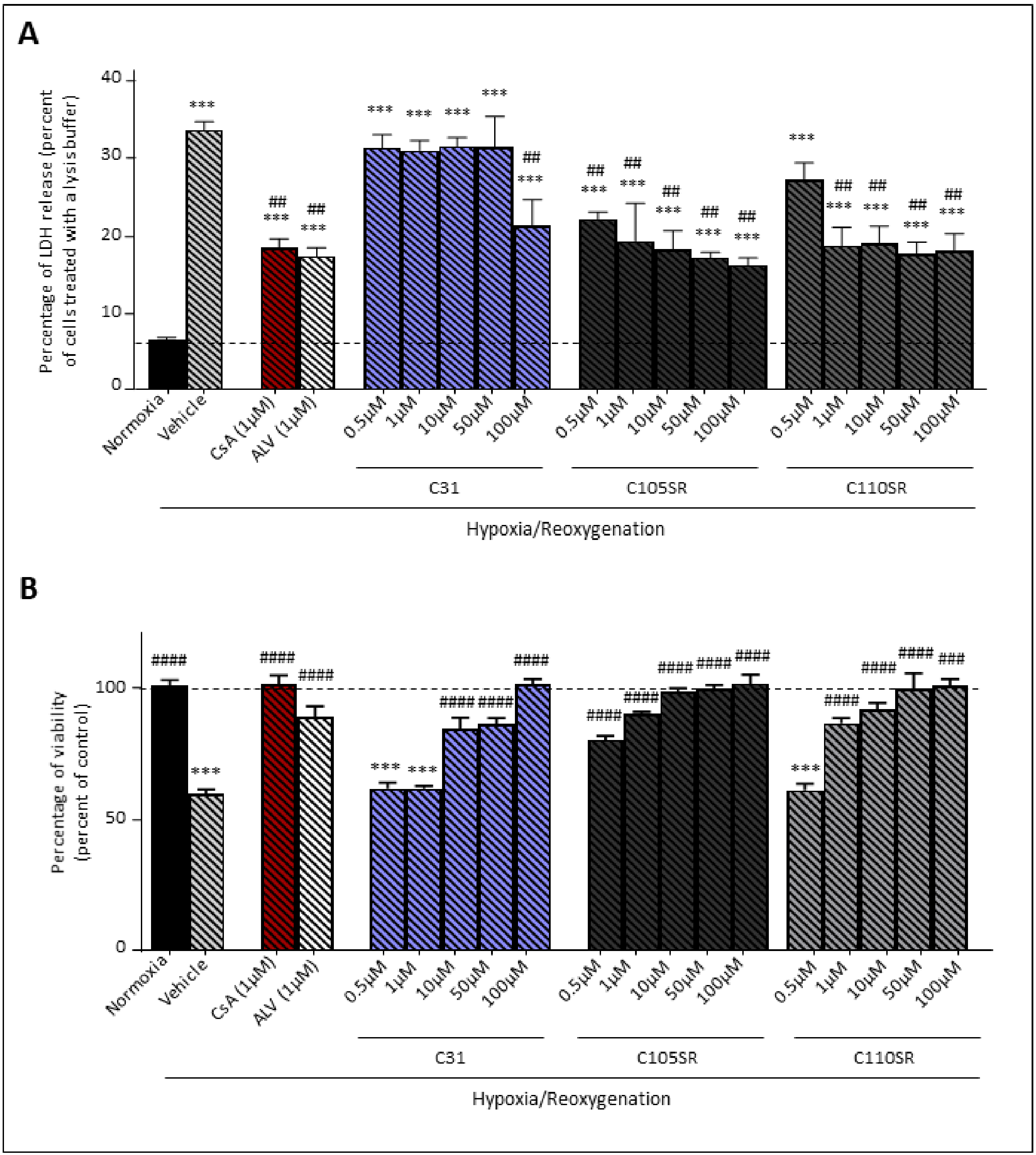


**Supplemental Figure 7**: Protection against cell death by pharmacological postconditioning with C105SR and C110SR. Cells were subjected to 4 h of hypoxia (1% $O_2$) followed by 2 h of reoxygenation (21% $O_2$). CsA and ALV were used as references. CsA and ALV were added at 1 µM while C31, C105SR or C110SR were added at increasing concentrations during the reoxygenation phase. (A) LDH release from cells exposed to normoxia or hypoxia/reoxygenation in the absence (vehicle) or in the presence of CsA, ALV or increasing concentrations of C31, C105SR or C110SR expressed as percentage of LDH release in control cells (Ctrl). ***$p < 0.001$ vs Ctrl; ##$p < 0.01$ vs hypoxia/reoxygenation vehicle. (B) Cell viability measured by MTT assay in cells exposed to normoxia (Ctrl) or hypoxia/reoxygenation in the absence (vehicle) or in the presence of CsA, ALV or increasing concentrations of C31, C105SR or C110SR expressed as percentage of control (normoxia). ***$p < 0.001$ vs Ctrl ; #$p < 0,05$ vs hypoxia/reoxygenation vehicle.

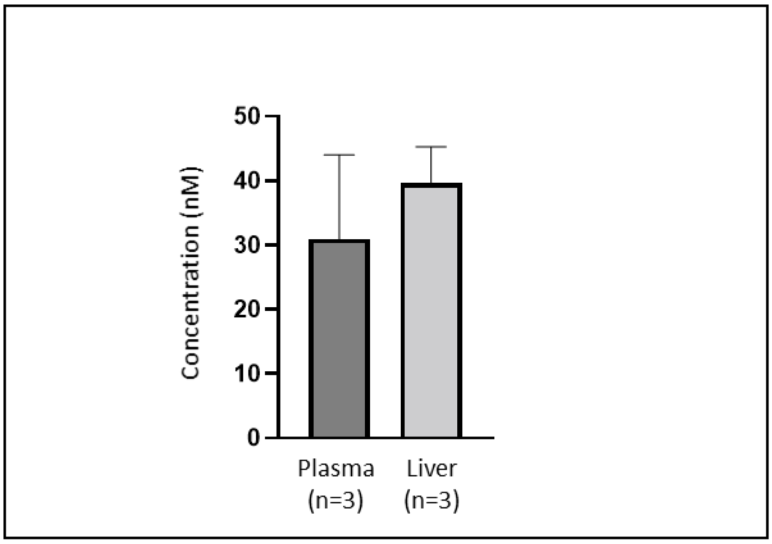


**Supplemental Figure 8:** Plasmatic and hepatic concentrations of C105SR 24 hours after osmotic pump implantation.
